# Validity of PROMIS^®^ Pediatric Physical Activity Parent Proxy Short Form Scale as a Physical Activity Measure for Children with Cerebral Palsy Who Are Non-Ambulatory

**DOI:** 10.3390/bs15081042

**Published:** 2025-07-31

**Authors:** Nia Toomer-Mensah, Margaret O’Neil, Lori Quinn

**Affiliations:** 1 Department of Biobehavioral Sciences, Teachers College, Columbia University, 520 W. 120th Street, Building 528 Room 1056A, New York, NY 10027, USA; lq2165@tc.columbia.edu; 2 Doctor of Physical Therapy Program, Department of Physical Medicine and Rehabilitation, University of Colorado Anschutz Medical Center, Aurora, CO 80045, USA; 3 Motor Development Laboratory, University of Southern California, Health Research Association, 1640 Marengo St. Suite 408, Los Angeles, CA 90033, USA; 4 Department of Physical Therapy and Kinesiology, Zuckerberg College of Health Sciences, University of Massachusetts, 113 Wilder Street, Suite 377, Lowell, MA 01854, USA; maggie_oneil@uml.edu

**Keywords:** physical activity, cerebral palsy, non-ambulatory, PROMIS^®^, wearable-monitors, heartrate, accelerometers, parents, physical therapy, Actigraph, FitBit

## Abstract

**Background**: Self-report physical activity (PA) scales, accelerometry, and heart rate (HR) monitoring are reliable tools for PA measurement for children with cerebral palsy (CP); however, there are limitations for those who are primary wheelchair users. The purpose of our study was to evaluate face and construct validity of the PROMIS^®^ Pediatric PA parent proxy short form 8a in measuring PA amount and intensity in children with CP who are non-ambulatory. **Methods**: Face validity: Semi-structured interviews with parents and pediatric physical therapists (PTs) were conducted about the appropriateness of each item on the PROMIS^®^ Pediatric PA short form. Construct validity: Children with CP who were non-ambulatory participated in a one-week observational study. PA amount and intensity were examined using PA monitors (Actigraph GT9X) and HR monitors (Fitbit Charge 4). Activity counts and time in sedentary and non-sedentary intensity zones were derived and compared to the PROMIS^®^ T-scaled score. **Results**: Twenty-two physical therapists (PTs) and fifteen parents participated in the interviews, and ten children completed 1-week PA observation. Eight and seven participants completed sufficient time of uninterrupted PA and HR monitor wear, respectively. Parents and PTs agreed that several questions were not appropriate for children with CP who were non-ambulatory. PA intensity via activity counts derived from wrist worn monitors showed a strong positive correlation with the PROMIS^®^ PA measure. **Conclusions**: Construct validity in our small sample was established between PROMIS^®^ scores and accelerometry activity counts when documenting PA amount and intensity; however, there were some differences on PROMIS^®^ face validity per parent and PT respondents. Despite some concerns regarding face validity, the PROMIS^®^ Pediatric PA parent proxy short form 8a shows promise as a valid measure of physical activity amount and intensity in non-ambulatory children with CP, warranting further investigation and refinement.

## 1. Introduction

Physical activity (PA) is defined as any bodily movement produced by skeletal muscle that requires energy expenditure for successful participation in everyday activities (2018 Physical Activity Guidelines Advisory Committee, 2018). Cerebral palsy (CP) is the most common motor disability in childhood and affects 1 in every 1000 children in the United States (Oskoui et al., 2013). Motor ability of children with CP is typically classified using the gross motor function classification system (GMFCS) (Palisano et al., 1997). Children with CP functioning at Level IV and V are primary wheelchair users; children functioning at level IV can take steps in a weight-supported gait trainer and those functioning at level V require a manual tilt-in-space wheelchair to maintain head and trunk alignment during sitting and community transportation (Palisano et al., 1997). Unfortunately, children with CP functioning at GMFCS levels IV and V have a greater risk of developing secondary impairments (such as reduced bone density, low muscle mass, and orthopedic contractures) associated with lack of PA (Orlin et al., 2010).

Few studies evaluate PA in children who are non-ambulatory, however there is evidence supporting reliable PA measures in children with CP who are ambulatory (Capio et al., 2010; Clanchy et al., 2011). Measuring VO_2 max_ (the maximum oxygen inspired during the most intense level of vigorous exercise) during activity is the gold standard to determine PA intensity (Hui & Chan, 2006). However, the use of direct and indirect calorimetry to determine VO_2_ max is not commonly feasible for children with CP who are non-ambulatory, given the severity of their motor impairments, a lack of expensive equipment for clinical use, and complications related to spinal and extremity contractures that can negatively influence respiration (i.e., use of face mask when utilizing indirect calorimetry). Furthermore, other direct measures of PA can be obtained using accelerometry (Clanchy et al., 2011; Trost, 2001) and heart rate (HR) monitoring (Balemans et al., 2014; Hui & Chan, 2006) from wearable monitors.

Accelerometers are reliable and valid measurement tools that can provide PA amount and intensity for children with CP functioning at levels GMFCS I–III (Mitchell et al., 2015; O’Neil et al., 2014), and may be a feasible and meaningful measure of daily activity in children functioning at GMFCS level IV (Gorter, 2017). HR monitoring has been shown to be a reliable measure of energy expenditure in children without disabilities (Livingstone et al., 1992; Rose et al., 1990), and ambulatory children with CP (Blanchard et al., 2016), and has been validated as a measure of PA intensity in ambulatory children with CP (Balemans et al., 2014; Dirienzo et al., 2007; Israeli-Mendlovic et al., 2014). HR changes reflect the body’s physiological response to movement, exercise, emotions, or stimuli, and are commonly used as a training tool for aerobic conditioning, providing a measure of exercise intensity (Fleming et al., 2011). HR monitoring with PA assessment improves accuracy in typically developing children, though predicting energy expenditure in children with disabilities remains difficult due to altered movement patterns and metabolic differences (Brazendale et al., 2019; Nightingale et al., 2017). Nevertheless, PA monitoring presents challenges for non-ambulatory children with CP due to the need for caregiver assistance for many activities.

While several reliable self-report measures of PA exist for children with CP aged six and older such as the Activity Scale for Kids–adolescence (ASK) (Young et al., 2009), the Children’s Assessment of Participation and Enjoyment (CAPE) (King et al., 2007), and Physical Activity Questionnaire–Adolescent (PAQ-A) (Clanchy et al., 2011; King et al., 2007), these tools have not been validated for non-ambulatory adolescents with CP (GMFCS levels IV and V). These measures often include many activities that are upright or ambulatory that many of these children cannot perform. Additionally, children with severe motor impairments may have limited communication and cognitive abilities, making self -report unreliable or infeasible. In these cases, a validated parent proxy PA report becomes essential to capture habitual behaviors. The Patient Reported Outcome Measurement Information System (PROMIS^®^) Pediatric Physical Activity Scale Parent Proxy Short Form (PPSF) 8a offers a structured tool to assess PA across intensity levels for ages 5–17 years when using a parent proxy (Tucker et al., 2020). However, its use and validity in non-ambulatory children with CP has not been explored, highlighting a critical gap in appropriate PA measurement in this population.

It is important for clinicians to value what parents believe is important as it relates to PA measurement (Terwiel et al., 2017). The use of parent and pediatric physical therapists (PTs) responses will provide stakeholder response correlating to habitual patterns of this population. Assessing the correlation between wearable sensors and PROMIS measures could offer clinicians a valuable framework for evaluation. Henceforth, if there is agreement between PTs and parents, this would support family centered coordination (Council on Children with Disabilities and Medical Home Implementation Project Advisory Committee, 2014) approaches to interventions, measures, and plans of care for children with CP at GFMCS levels IV and V.

The purpose of this study was to evaluate face and construct validity of the PROMIS^®^ Pediatric PA PPSF 8a in measuring PA amount and intensity in children with CP who are non-ambulatory. We selected the PROMIS PA scale because the question rankings yielded optimal thresholds that allow for parents and children of varied functional levels to engage (Tucker et al., 2020). We explored parent and pediatric physical therapists’ opinions about the PROMIS^®^ Pediatric PA PPSF 8a scale to provide evidence regarding the validity of this measure for children with CP functioning at GMFCS levels IV and V. To investigate construct validity, we compared the PROMIS^®^ PPSF 8a to direct methods of PA measures (HR monitors and accelerometers) over a one-week observation period in children with CP who were non-ambulatory.

## 2. Materials and Methods

This study consisted of two parts. First, we evaluated the face validity of the PROMIS^®^ Pediatric PA PPSF 8a scale by conducting 1:1 interviews with pediatric PTs and parents of children with CP at GMFCS levels IV and V. Second, we investigated the construct validity of the PROMIS^®^ Pediatric PA PPSF 8a as a measure of PA in children with CP who were non-ambulatory by comparing it to PA measures obtained through PA and HR monitors over a one-week period.

### 2.1. Participants

#### 2.1.1. Part 1: Parent and PT Interviews

Parents of children with CP who were non-ambulatory were recruited through emails sent to private schools for children with disabilities and local PT networks (word-of-mouth from therapist within American Academy of Pediatric Physical Therapy listserv). PTs were recruited via email to the department and program directors at specialized schools as well as local PT networks. PTs and parents who responded with interest were emailed a survey to identify if they were appropriate to participate in this study. The inclusion criteria for PTs had a minimum of 25% of their caseload with children who were primary wheelchair users either currently or in the past. Inclusion criteria for parents were to have a child with CP who is non-ambulatory (GMFCS levels IV and V). Once participants were deemed eligible a follow-up email was sent to offer an introductory phone call or teleconference meeting to review protocol along with a copy of the informed consent form. In this email, interested PTs and parents received a Research Electronic Data Capture (REDCap) version 12 (Vanderbilt University, 2021) link to provide electronic-consent (e-consent) with details about the study inclusion criteria and the expectations for participants. After the parent or PT completed the e-consent, the participant was contacted to schedule a time and date to meet with the principal investigator (PI) for the audio and video (optional) recorded interview. This study was approved by the Institutional Review Board at Teachers College, Columbia University (TC 23-093) and all participants signed informed e-consent prior to the interview.

#### 2.1.2. Part 2: PA and HR Monitoring

Families were recruited through emails sent to private schools for children with disabilities, and local PT networks to compare the PROMIS^®^ Pediatric PA PPSF 8a to PA and HR monitors during one-week of observation and wear time. Ten parent participants were recruited via an email circulated to specialized schools for children from 5 to 21 years of age with CP. Inclusion criteria were children with (1) CP diagnosis; (2) enrollment as full time K-12 student; (3) GMFCS IV or V; and (4) self-initiated movement, such as minimal voluntary use of their head and/or upper extremity to access and use a switch/communication device. Exclusion criteria were children who had the following: (1) surgery within the past six months; (2) uncontrollable seizures; or (3) any contraindications to participating in PA with or without a caregiver or assistive device. Once participants were deemed eligible, a follow-up email was sent with a copy of the informed consent form and a request to conduct an introductory phone call or teleconference meeting to review the informed consent form and protocol. During this introductory meeting, the parents were able to ask questions about the project and were given instructions on how to complete e-consent via REDCap online database via the link provided in email communication. This study was approved by the Institutional Review Board at Teachers College, Columbia University (TC 21-079) and all participants signed informed e-consent prior to data collection.

After e-consent, parents were asked to complete the PROMIS^®^ Pediatric PA PPSF 8a via Zoom teleconferencing, REDcap, or by hand (paper copies were included in mailed package with wearable monitors) before and after the child participated in 7 days of wearing the Actigraph GT9X (PA monitor; Actigraph, Pensacola, FL, USA) and Fitbit Charge 4 (HR monitor; Fitbit, San Francisco, CA, USA) to measure PA amount and intensity. All families were emailed REDcap surveys consisting of e-consent, and PROMIS PPSF 8a; however, some parents did not complete all surveys after consent was obtained.

#### 2.1.3. Pediatric Physical Activity Scale Parent Proxy Short Form (PPSF) 8a

The PROMIS^®^ is a set of person-centered measures developed with support from the U.S. National Institutes of Health (Cella et al., 2010) that were designed to be relevant across populations for the assessment of function and symptoms to enhance communication among clinicians and patients in research and clinical settings. The PROMIS^®^ PPSF 8a includes eight items, each asking how many days in the past week a child engaged in specific physical activities. Response options range from “No days” to “6–7 days,” scored from 1 to 5, respectively. The total raw score (ranging from 8 to 40) is converted into a standardized T-score using Item Response Theory (IRT) calibration. T-scores have a mean of 50 and a standard deviation of 10, with higher scores indicating greater physical activity levels. In this study, T-scores were calculated using REDCap automated scoring feature based on PROMIS scoring parameters (Tucker et al., 2020). A maximum score of 100 indicates the highest level of intensity/activity during the week.

### 2.2. Interview and Monitoring Methods

#### 2.2.1. Part 1: Interview Protocol

Individual semi-structured interviews were conducted via teleconferencing (Zoom^®^, San Jose, CA, USA) with both PTs and parents. The PTs or parents responded to questions about the relevance of each of the 8 items on the PROMIS^®^ PA PPSF 8a for children with CP (GMFCS IV and V) (Appendix A).

PTs and parents were asked to use a Likert scale (0 = not at all, 5 = highly appropriate) to rate the following question for each of the 8 items on the PROMIS^®^ PA PPSF 8a: “How appropriate is the question to addressing PA intensity in children with CP at GMFCS levels IV and V?”. A follow-up open-ended question about each item provided an opportunity for respondents to discuss their rating. There was no blinding of the assessor as the PI consented, contacted, and conducted all interviews.

#### 2.2.2. Part 2: Wearable Devices Protocol

The monitors, adjustable bands, instructions on monitor wear and use, and PROMIS^®^ forms were mailed or delivered via non-contact methods to each consented family’s home (this study was conducted during the COVID pandemic). The parents were instructed on where to place the monitors on their child (one HR and two PA monitors) for a 7-day period for 23 h per day. Based on family preference, a video or phone call was scheduled to review placement or any questions the family had prior to the start of the procedures. The PA monitors (Actigraph (GTX9) accelerometers, Actigraph Corporation Pensacola, Pensacola, FL, USA) were worn on the child’s dominant wrist and waist via adjustable belt strap with clips that snap for closure. The HR monitor (Fitbit^®^ Charge 4, San Francisco, CA, USA) was worn on the non-dominant wrist. The Actigraph GT3X has been validated to record PA in children with CP (O’Neil et al., 2016; Trost et al., 2016). The Fitbit has been shown to be a reliable tool for HR measurement in children with CP (Brazendale et al., 2019; Schmit & Miros, 2016).

### 2.3. Data Processing

#### 2.3.1. Transcript Verification

After the participant interviews were completed, the PI (NM) compared each Zoom teleconferencing generated transcript to the audio file for accuracy and transferred the text file to a Microsoft word file to deidentify and edit each interview transcription for accuracy based on the original audio file (See Supplementary Materials). These deidentified files were further reviewed by co-author (LQ) for accuracy.

#### 2.3.2. PA Monitor Analysis

Actigraph data was processed using ActiLife (Version 6) [Computer software] (Pensacola, FL, USA). PA amount was reported as raw activity counts per minute (cpm) that were collected in 15 sec epochs (60 Hz frequency). Wear time for each wrist worn Actigraph device was compiled and divided into sedentary and non-sedentary time over the 7-day observation period. The counts per minute values were analyzed for the hours of 7 a.m. to 7 p.m. to determine minutes per day the child was either sedentary or non-sedentary. Activity zones were classified using CP cut points (Trost et al., 2016). We applied these cut points in this study, though they were established for children and youth at GMFCS I–III. We used the cut points to determine non-sedentary (less than 288 counts per minute) versus sedentary behavior (greater than 288 counts per minute). We used these cut points because none exist for children and youth at GMFCS IV–V. CP cut points established by Trost et al. (2016) are a proxy for PA intensity derived from accelerometry (activity counts) and indirect calorimetry (oxygen uptake) data (Trost et al., 2016). Current research challenges normative cut points for children with CP as there is evidence for individualized norms for each GMFCS classification level with case specific investigation to validate PA measurement (Bianchim et al., 2020; Gorter et al., 2012; Keawutan et al., 2018; Oftedal et al., 2014; Orlando et al., 2019). Due to the small sample and limited observation time, we could not derive case specific PA measurements for the participants in this study. In the absence of CP-specific non-ambulatory cut points, we used Trost CP GMFCS level III values (288 counts per minute (cpm) = indicator for non-sedentary behavior) as a proxy due to lack of GMFCS IV–V specific norms (Trost et al., 2016). The research team opted to use only the wrist data for comparison as we found greater activity count in pilot data during comparison of PA in three children at GMFCS level IV–V when comparing school to home activity patterns when using an activity journal and wearable monitors (Toomer-Mensah, 2023). This has also been found in recent studies when evaluating PA in youth with CP (Alamoudi et al., 2024). The statistician did not know the GMFCS levels of each child.

#### 2.3.3. HR Monitor Analysis

Raw HR data retrieved from the Fitbit were averaged to produce HR beats per minute (bpm) values and further analyzed through RStudio (Version 1.4.1717, © 2009-2021 RStudio, PBC) [Computer software] for the hours of 7 a.m. to 7 p.m. to determine minutes per day the child was either sedentary or non-sedentary. The HR values corresponding to each intensity level for the children were defined as sedentary (HR < 111 bpm, <57% of HRmax) and non-sedentary (HR ≥ 111 bpm), based on guidelines from American College of Sports Medicine (ACSM) (American College of Sports Medicine, 2016).

#### 2.3.4. Sedentary vs. Non-Sedentary Analysis

The percentage of non-sedentary minutes during the observation period was calculated by dividing the non-sedentary minutes by the total number of minutes over four consecutive days. When less than four days of data were available, percentages of non-sedentary time were determined based on available minutes collected.

We compared the PROMIS^®^ T-scores of parent-reported PA intensity to the PA (activity counts) and HR (bpm) data to calculate a continuum of time the children were sedentary or non-sedentary as a measure of PA intensity. Higher PROMIS^®^ PA T-scores indicate higher PA levels (PA intensity) reported by the parents for their children. The PA intensity derivation methods and definitions are identified in Table 1 below.

### 2.4. Statistical Analysis

Interview analysis. Quantitative ratings (5-point Likert scale with 0 = not at all related, 5 = highly appropriate; e.g., “How appropriate is the question to address PA intensity in children with CP at GMFCS level IV and V”) were collected, and descriptive statistics were used to determine the PROMIS^®^ question that has the greatest face validity (highest level of appropriateness using mean and median scores) based on the participants’ ratings. Due to the small sample size and lack of normally distributed data, a Mann–Whitney U test was used to investigate group differences between parents and therapist related to their views about the appropriateness of each question on the PROMIS^®^ scale (Hart, 2001).

PA and HR monitor analysis. We used Kendall’s tau-b correlation and *p*-values to compare the PROMIS^®^ T-score to the percentages of sedentary and non-sedentary times as recorded via the PA and HR monitors. Kendall’s tau-b is a non-parametric statistic used to measure the strength and direction of association between two ranked ordinal variables.

## 3. Results

### 3.1. Participants

Twenty-two physical therapists participated in the interviews. Table 2 provides an overview of PT demographics including education and current work setting. The age range for the PTs was 46–50 years of age with an average of 21.1 years of practice experience. Fifteen parents participated in the interviews. Table 3 provides an overview of parent demographics including education, ethnicity, child’s age and gender, and the number of siblings in the home. There was one male parent participant (PA14), and the remainder of participants were female respondents. The average age of the children with CP was 13.2 years.

### 3.2. PROMIS^®^ PT and Parent Responses

The eight questions in the PROMIS^®^ PA PPSF 8a are found in Appendix A. Parents scored questions 7, 6, 2, and 4 as the most appropriate questions, respectively, for their child’s level of functioning. The PTs scored questions 7, 6, 3, 2 and 1, as the most appropriate questions, respectively.

The Mann–Whitney U test results indicated no significant difference (*p* > 0.05) between parents and PTs ratings on appropriateness of the PROMIS^®^ PA PPSF 8a questions. Ratings between parents and PTs were similar except for Q4 (*p* = 0.07) (Table 4).

Question 7 (How many days was your child physically active for 10 min or more?) received highest scores for appropriateness from both parents and PTs. Question 6 received the second highest rating from both groups (How many days did your child exercise or play so hard that he/she felt tired?). The most inappropriate question (lowest rating) for both parents and PTs was Question 8 (How many days did your child run for 10 min or more?), followed by Question 5 (How many days did your child exercise or play so hard that his/her muscles burned?).

### 3.3. PROMIS^®^ Comparison to PA and HR Monitors

Ten children with CP who were non-ambulatory participated in this one-week observational study. One child (participant 8) was deemed within GMFCS level III (part-time walker) after post hoc analysis and was excluded from group comparisons. All participants attended private specialized schools in different regions (states included NY, PA, and IL) for children with disabilities. Demographic information about each participant’s functional classification level, age, race, and PROMIS^®^ PA PPSF 8a T-scores are found in Table 5.

Three participants did not complete the post-observation PROMIS^®^ PA PPSF 8a and in those instances, we used the preliminary PROMIS^®^ PA PPSF 8a scores with the assumption that the prior weekly schedule was similar to the observation week. Due to malfunction or non-wear of the HR or PA monitor, eight and seven participants, respectively, completed sufficient uninterrupted monitor wear time on both devices to calculate intensity zones. Table 6 provides an overview of the calculated monitor wear per participant and percentage of time in non-sedentary activity per monitor.

There was a significant strong positive correlation between the PROMIS^®^ PA T-scores and the percentage of non-sedentary time based on PA count data (Kendall’s tau-b = 0.714, *p* = 0.024, *n* = 8) indicating a relationship between the PA monitors and the PROMIS scale. There was no significant relationship between the PROMIS^®^ PA PPSF 8a and HR data (bpm) (Kendall tau-b = 0.143, *p* = 0.624, *n* = 7). Kendall’s tau-b was used due to the ordinal nature of the data and small sample size.

## 4. Discussion

The results of this study suggest that the PROMIS^®^ PA PPSF 8a lacks face validity for assessing PA in children with CP who are non-ambulatory. PTs might consider utilizing a modified version of the PROMIS^®^ PA PPSF 8a for children with CP who are non-ambulatory, specifically the three questions with the highest parent mean ratings (Q7, Q6, and Q4), as parents are best able to identify levels of fatigue in their children (Guimarães et al., 2023). Nonetheless, PTs and parents agree on the level of appropriateness of each question within the PROMIS^®^ PA PPSF 8a. If the survey is used, it is recommended that clinicians provide qualifying commentary to aid the family in providing comparable measurement or omit questions 5 and 8.

Both parents and PTs did not support the use of Question 8 due to the obvious reference to running which may be offensive to a population of children who are not full-time walkers. Constructive critique from some parents included a recommendation to include language such as ‘with or without adaptive equipment or gait trainer’ to make question 8 more specific and relevant to their children’s mobility capabilities (i.e., running ability). This revision to Question 8 would improve the accuracy, precision, and generalizability of the item for use in children with different PA abilities. The parents interviewed in our study also felt the question about muscle soreness (Q5) represented pain for these children because of the difficulty for parents or children to discriminate muscle soreness due to PA or having pain due to other reasons. Pain is commonly reported in children with CP (Boyd et al., 2017; Ostojic et al., 2019) and therefore parents and PTs prefer not confuse respondents with this question on muscle soreness that may be construed as a question on pain.

Question 4 (“How many days was your child so physically active that he/she sweated?) was deemed more appropriate by parents as compared to PTs. Furthermore, PTs reported that most children with CP at GMFCS levels IV–V do not sweat often because of impaired thermoregulation. However, the PTs in our study reported that they do not often push these children to a point of exertion and sweating because they are concerned about the risk of medical complications, respiratory failure, and fatigue that would prevent engagement during the remainder of the day’s activities. Question 4 was the only question where there was some discrepancy among parents and PTs as parents felt this question was somewhat appropriate because sweating is evidence of physical exertion for their children.

When reviewing the original item data bank in the PROMIS PA scale formation (Tucker et al., 2020), the psychometric parameters and item response means correspond to the results of our study. The questions related to muscle pain and running (Q5 and Q8 in the PPSF 8a), both showed lower mean scores (‘muscles burning’ scored the lowest), higher thresholds, and moderate discrimination when compared in a sample of over 2000 typically developing children and adolescents (Tucker et al., 2020). This means fewer children were able to endorse a response at the higher frequency of 6–7 days a week, and that these questions are reflective of high-effort PA. These items are the most intensity-specific, and while they may be valuable for assessing vigorous PA, their contribution to assessing general activity patterns across a whole population (i.e., inclusive to children functioning at GMFCS IV–V) is limited. Furthermore, when the Early Childhood PA scale was developed for children aged 1 to 5 years, modifications were made to replace the ‘muscle soreness’ and ‘running’ items with a ‘fatigue’ item, which reduced the parent scale to seven items. Items were also reworded to include napping and falling asleep early (Lai et al., 2022). Similar item responses to our study were found in a validation study of the PROMIS^®^ Child PA scale when the lowest ranked questions were the questions related to muscle soreness and the ability of children running for 10 min a day in a sample of 681 Swedish youth aged 12–18 years (Carlberg Rindestig et al., 2021). This supports our observation that Q5 and Q8 are the most difficult to endorse (evidenced by higher threshold), and that these items fall at the upper end of the PA continuum that do not reflect the experience of less active children or those with movement limitation.

In order to determine construct validity, when compared to the accelerometers, the PROMIS^®^ PA PPSF 8a T-scores showed a significant and strong correlation indicating a positive relationship. Two categories of activity (sedentary and non-sedentary) were selected because the Trost CP cut points for multiple activity zones are not validated in children greater than GMFCS level III. There are validated cut points that have been used for children who are typically developing; however, these values tend to underestimate movement capabilities in children with disabilities (Bianchim et al., 2020; Clanchy et al., 2011; Oftedal et al., 2014). We were conservative in using these cut points (Trost et al., 2016) in children with CP due to the greater functional limitation of our population. Prior studies have shown construct validity of the PROMIS PA scale when comparing active disease status in children with idiopathic juvenile arthritis and lupus in 451 youths between the ages of 12 and 18 (Weitzman et al., 2023). In a sample of 81 children, a moderate correlation was found with the Fitbit utilizing step counts for PA measurement when compared to the self-reported responses on the survey, with a preference from the participants for 7-day recall versus frequent monitoring (Algheryahi et al., 2023).

### 4.1. Limitations

The PROMIS^®^ PA PPSF 8a relies on parental perception of their child’s exertion as a proxy for PA intensity, which may be influenced by education level, health and physical activity literacy, and caregiving experience (Østensjø et al., 2003). PA and HR monitoring in a population with hypokinesia may result in misleading or biased measures related to caregiver assistance in their child’s movement, and discrepancy related to HR changes. HR monitoring, while useful, does not solely reflect PA levels, as HR fluctuations can result from emotional, homeostatic, or other intrinsic factors. Understanding these limitations can help therapists avoid biases in PA assessment.

The study sample was predominantly Caucasian children, limiting diversity. The small sample size restricts the generalization of findings. We did not calculate sample size a priori; however, the authors used prior qualitative studies investigating parent perspectives to devise an appropriate sample (Peplow & Carpenter, 2013; Shields & Synnot, 2016). Parent and PT responses support revising the PROMIS^®^ scale, but without validation of the recommended scale, the authors could not compare the removal of questions 5 and 8. Furthermore, a selection bias may be present as many of the PT participants worked in similar networks (American Academy of Pediatric PT, school-based PT, and pediatric faculty at accredited DPT programs) as PI Mensah.

As mentioned earlier, Trost CP cut points have not been validated in children with CP who are non-ambulatory (Trost et al., 2016). Global normative values for this population would be highly difficult to ascertain due to the vast heterogeneity of cognitive and motor levels among this group; therefore, some investigators suggest using case-specific values to help quantify individualized PA counts utilizing raw accelerometry data (Bianchim et al., 2020; Orlando et al., 2025). Finally, our study was conducted during the COVID-19 pandemic which presented challenges in remote delivery of the project as indicated by missing data due to some malfunctions in technology (i.e., PA and HR monitors) for several participants.

### 4.2. Clinical Implications

When using PA monitors, PTs may find it best to collaborate with the family about software and ways they can optimize tracking their child’s behaviors in the home and community. Many parents and pediatric PTs identified the lack of PA in this population, which is also supported in the literature (Alamoudi et al., 2024; Bedell et al., 2013; Bjornson et al., 2019; Carlon et al., 2013; Gorter et al., 2012; Mitchell et al., 2015). The lack of PA engagement may be related to the difficulty that parents may experience in identifying characteristics of increased PA intensity in their children (i.e., sweating, muscle soreness, and fatigue). The definitions of the term’s “exercise”, “play”, and “run” as represented in the questions on the PROMIS^®^ PA PPSF 8a need to be modified with explanations/definitions of terms for families for children who are non-ambulatory. Use of adaptive equipment and communication devices may be cited when providing examples if using this tool as a PA measure. Instead of asking “How many days did your child run for 10 min?” (Question 8), the assessment may be modified to say, “How many days did your child take steps in a weight supportive gait trainer?”. Measuring time for the duration of PA-related questions was preferred by both parents and PTs in our study, and serves as a quantifiable measurement that can assist with dosing and program implementation.

To our knowledge, this is the first study attempting to establish construct validity of the PROMIS^®^ PA Pediatric scale in children with CP, with significant motor impairment. The use of PA monitors can be helpful to quantify breaks in sedentary behaviors for these children, which is a viable and important intervention and measurement approach to promote increased PA. However, when direct observation is a viable option, HR monitoring may be a suitable measure. Direct observation will allow the PT to visually differentiate active movement from other autonomic changes that affect HR. When data collection is performed post-intervention, use of a PA monitor and parent-reported PROMIS^®^ PA PPSF 8a may provide the most congruent measure to a child’s habitual PA and free-living behaviors.

### 4.3. Conclusions

PROMIS^®^ PA PPSF 8a scores were associated with objective PA measures for sedentary vs. non-sedentary time, making it a useful complement or alternative when objective measures are unavailable. However, the PROMIS^®^ PA PPSF 8a requires modification to assess PA in non-ambulatory children with CP. Terms such as “running” and “muscle soreness” are inappropriate, while Q7 and Q6 appear to be the most applicable for this population. By refining PA assessment tools, PTs can better examine the effectiveness of their interventions to support non-ambulatory children in engaging in meaningful PA, improving mobility, and enhancing the overall quality of life.

## Figures and Tables

**Table 1:** PA intensity parameters and definitions as measured by identified source.

Measurement Tool		PA Monitor	HR Monitor	Parent Reported— PROMIS Pediatric PA Parent Proxy Scale
Derivation Method		PA counts converted to PA intensity levels via proxy use of CP cut points (Trost et al., 2016)	HR in beats per minute categorized into PA intensity zones (ACSM) utilizing percentage of HRmax (194 bpm, Verschuren et al., 2011)	8-question parent proxy scale (i.e., “How many days in a 7-day period did the child engage in physical activities?”). Calculations for the week indicate a T-score (0–100)
Sedentary	<Light PA	Values of <288 cpm *	HR < 111 bpm (<57% of HRmax);	T-score
Non-sedentary	>/=Light PA	>/=288 cpm *	HR >/= 111 bpm (>/=57% of HRmax)	T-score

Definitions and classifications for PA monitor accelerometer (Actigraph 3GTX) and HR monitor (FitBit Charge 4) are identified here. Sedentary activity was classified as less than 288 counts per minute (cpm—PA monitors) and less than 111 beats per minute (bpm—HR monitors). Anything above this value was considered non-sedentary behavior. * Trost CP cut points * included in this table are not validated in children with CP at GMFCS levels IV and V. HR = heart rate; PA = physical activity; cpm = counts per minute.

**Table 2:** Physical therapist (PT) demographics.

ID	Settings as a Peds PT	Years as PT:	Age Range	Highest Level of Education Completed	Certifications
1	School	19	41–45	Transitional DPT degree	APTA-credentialed Clinical Instructor (CI)
2	Hospital/Homecare	33	56–60	Transitional DPT degree	ATP, C/NDT
3	Early intervention	35	56–60	Terminal research degree (PhD)	
4	Early intervention–home-based	27	46–50	Transitional DPT degree	PCS
5	Outpatient pediatric	12.5	36–40	Entry-level doctorate degree	
6	School	15	46–50	Entry-level doctorate degree	PCS, CPST
7	Hospital, clinic, academia	40	56–60	Terminal research degree	PCS
8	Rehab Hospital	43	>60	Transitional DPT degree	PCS
9	Hospital-based outpatient	5.5	26–30	Entry-level doctorate degree	PCS
10	School	14	31–35	Transitional DPT degree	CI
11	School	15	41–45	Not applicable	NDT, BSPTS-Schroth
12	School	20	41–45	Transitional DPT degree	Neuro developmental treatment-NDT
13	Hospital	12	36–40	Entry-level doctorate degree	
14	Hospital/Early intervention	25	46–50	Terminal research degree (PhD)	PCS, NDT
15	PT Home and Clinic	22	46–50	Terminal research degree (PhD)	PCS, EI, CPST
16	School	20	41–45	n/a	
17	Home and outpatient	20	41–45	Transitional DPT degree	C/NDT. PCS
18	Approved private school	42	>60	Transitional DPT degree	NDT trained
19	School	4.5	36–40	Entry-level doctorate degree	
20	Peds, school	9	31–35	Entry-level doctorate degree	APTA-credentialed CI
21	School	8	31–35	Entry-level doctorate degree	
22	Approved private school	42+	>60	Master’s degree	ATP

Pediatric PTs demographics including education and current work setting. Average age range for the PTs were between 46 and 50 years of age with mean and SD of 22.0 and 12.4 years of practice experience.

**Table 3:** Parent demographics.

Participant	Child’s Age	Child’s Gender	No. Siblings	Parent Ethnicity	Highest Level of Education Completed
PA1	12	Male	1	European descent	College diploma
PA2	16	Male	0	Hispanic	Some college
PA3	9	Male	2	European descent	Post-graduate diploma
PA4	25	Female	0	European descent	College diploma
PA5	13	Female	3	European descent	Post-graduate diploma
PA6	12	Male	0	Prefer not to answer	Graduate diploma
PA7	20	Male	1	Hispanic	Some college
PA8	10	Female	3	European descent	Graduate diploma
PA9	16	Female	0	Hispanic	Some college
PA10	15	Male	2	African American	Graduate diploma
PA11	4.5	Male	3	European descent	High school diploma
PA12	15	Female	0	Hispanic	Some college
PA13	10	Female	1	European descent	Post-graduate diploma
PA14 *	10	Female	1	African American	Post-graduate diploma
PA15	11	Male	1	Hispanic	Some college

Parent demographics including education, ethnicity, child’s gender, and number of siblings in the home. Mean and SD were 13.2 and 4.9 years, respectively. * PA14 was the only male parent participant of our study. All other parent participants were mothers of children with CP.

**Table 4:** Descriptive statistics PT and parent Likert score for individual PROMIS^®^ items.

Questions	Physical Therapist Mdn (IQR)	Parent Mdn (IQR)	MWU *p*-Value
1. How many days did your child exercise or play so hard that his/her body got tired?	3.5 (3.75)	3 (3.5)	0.680
2. How many days did your child exercise really hard for 10 min or more?	3 (3)	4 (3)	0.417
3. How many days did your child exercise so much that he/she breathed hard?	3.5 (1)	2 (4.5)	0.400
4. How many days was your child so physically active that he/she sweated?	2 (2.75)	4 (3)	0.070
5. How many days did your child exercise or play so hard that his/her muscles burned?	1 (2.25)	2 (3)	0.511
6. How many days did your child exercise or play so hard that he/she felt tired?	4.5 (1.75)	4 (1.5)	0.680
7. How many days was your child physically active for 10 min or more?	5 (1)	5 (0)	0.531
8. How many days did your child run for 10 min or more?	0 (0)	0 (0)	0.819

Median (Mdn) and interquartile range (IQR) of parent and PT responses about each question on the PROMIS^®^ scale. Mann–Whitney U (MWU) comparison *p*-value (alpha = 0.05): parents vs. PTs response per question. Mean rank for parent and PT responses is listed per each question on the PROMIS^®^ PA PPSF 8a.

**Table 5:** Child participant demographics.

Participant	Ethnicity	Gender	Age	GMFCS	PROMIS T-Score
1	Caucasian	F	15	5	36.5
2	Hispanic	M	19	5	47.7
3	Caucasian	M	11	4	57.3 *
4	Russian	M	15	5	56.7
5	Caucasian	F	10	4	52.9
6	Caucasian	M	11	5	39.8 *
7	Hispanic	M	14	5	28.3
8	Caucasian	M	12	3	52.4 *
9	Caucasian	M	9	4	49.2
10	Caucasian	F	10	4	41.2
Mean (SD)			12.6 (2.9)		46.2 (8.99)

Overview of demographic information as well as gross motor functional classification scale (GMFCS) and PROMIS PA Parent Proxy Short Form T-scores for all participants as well as functional classification scales * indicates when the PROMIS PA T-scores of the post-week scores were utilized.

**Table 6:** Wearable monitor data for each participant.

N	HR Monitor (days)	PA Monitor (days)	% Non-Sedentary (HR)	% Non-Sedentary (PA)
1	8	7	3.3%	6.5%
2	8	0	8.4%	Not applicable
3	7	0	11.9%	Not applicable
4	6	6	9.3%	16.6%
5	2	1	52.4%	38.7%
6	7	6	32.3%	10.6%
7	0	7	Not applicable	3.6%
8	0	1	Not applicable	Not applicable
9	7	7	9.5%	29.8%
10	8	8	18.7%	8.6%

Percentage of non-sedentary time for 12 h (7 a.m.–7 p.m.) per day. Most children had four full days of wear completed, and therefore we used the averages over this timeframe. Comparison of average minutes/week of sedentary and non-sedentary (light PA to moderate–vigorous PA) using Trost CP cut points and HR intensity zones based on HRmax (193 bpm) in children with CP. There were five instances where insufficient wear time due to device malfunction resulted in unreported sedentary/non-sedentary calculations.

## Data Availability

Due to privacy and ethical restrictions, all data is stored on encrypted hardware within the university’s HIPPA drive; therefore, data is unavailable.

## Supplementary Materials

The following supporting information can be downloaded at: https://www.mdpi.com/article/10.3390/bs15081042/s1, File S1: Transcripts: PT (22) and PA (15).

## Abbreviations

The following abbreviations are used in this manuscript:

PA	Physical Activity
CP	Cerebral Palsy
PROMIS^®^	Patient Reported Outcome Measurement Information System
PPSF	Parent Proxy Short Form
PT	Physical Therapist
PA	Parent (used in tables)
HR	Heartrate
ASK	Activity Scale for Kids–adolescence
CAPE	Children’s Assessment of Participation and Enjoyment
PAQ-A	Physical Activity Questionnaire–Adolescent

## Appendix A. PROMIS^®^ Physical Activity Parent and Physical Therapy Questionnaire

During the second half of this interview, each participant was asked to answer on a Likert scale (scale from 0 to 5, 0 = not at all related, 5 = highly appropriate). This interview question was asked about each of the eight questions on the PROMIS^®^ proxy scale (listed below). A follow-up to each item will include an opportunity to answer why the respondent scored the question as so.

PROMIS^®^ Parent Proxy Physical Activity–Short Form 8a (questions listed below are based on the past week 7 days):

Please respond to each question or statement by marking one box per row (scale indicates the following: no days (score = 1), 1 day (2), 2–3 days (3), 4–5 days (4), 6–7 days (5).

1. How many days did your child exercise or play so hard that his/her body got tired?
2. How many days did your child exercise really hard for 10 min or more?
3. How many days did your child exercise so much that he/she breathed hard?
4. How many days was your child so physically active that he/she sweated?
5. How many days did your child exercise or play so hard that his/her muscles burned?
6. How many days did your child exercise or play so hard that he/she felt tired?
7. How many days was your child physically active for 10 min or more?
8. How many days did your child run for 10 min or more?
