# Supplementary material for: Validity of PROMIS® Pediatric Physical Activity Parent Proxy Short Form Scale as a Physical Activity Measure for Children with Cerebral Palsy Who Are Non-Ambulatory
Source: Behav Sci (Basel). 2025 Jul 31;15(8):1042. doi: 10.3390/bs15081042 (PMC12382615; doi:10.3390/bs15081042)
Supplement: Supplementary file 1 [file behavsci-15-01042-s001.zip › Transcripts copy/Parent transcripts de-identified/Pa9.docx]

WEBVTT

1

00:00:00.680 --> 00:00:13.460

NM: All right. Good afternoon. Thank you so much for joining us today. We're going to talk a bit about physical activity for children that are not full time walkers. So when I ask you a few questions, if I sound scripted is because I am.

2

00:00:13.480 --> 00:00:20.020

NM: and then I have prompts after each question. And so there's no right or wrong answer, and I do look forward to hearing your responses.

3

00:00:20.030 --> 00:00:25.240

NM: So the first question is, how do you define physical activity for your child.

4

00:00:26.270 --> 00:00:27.090

Pa9: Okay.

5

00:00:28.950 --> 00:00:45.700

Pa9: for my child. Actually, I have high expectations for her. I would like to for her to be as an active as any other kid. of course, with the reality of her limitations, but with accommodations I

6

00:00:45.770 --> 00:00:52.930

Pa9: like for her to have physical activity doing. riding a bike. standing.

7

00:00:53.010 --> 00:01:01.070

Pa9: getting outside of the chair using a exercise goal. So for me, that is physical activity. Yeah.

8

00:01:01.300 --> 00:01:17.750

NM: Great, Thank you. The first prompt is the Department of Health defines physical activity as any activity that encompasses energy expended, and activation of skeletal muscles. Does this definition change your mind about how you define physical activity for your child? Why or why not?

9

00:01:19.470 --> 00:01:26.530

Pa9: Well, I think as it is to have physical activity for your health

10

00:01:27.810 --> 00:01:38.420

Pa9: that improves your You know your muscle strengtj, the your respiration for my daughter. She has aside of being cerebral palsy, so she has

11

00:01:38.770 --> 00:01:40.110

Pa9: other

12

00:01:40.140 --> 00:01:43.080

Pa9: medical diagnosis

13

00:01:43.230 --> 00:01:53.520

Pa9: which mainly a respiratory failure and uses a ventilator. So this is physical activity for her is very important to

14

00:01:53.900 --> 00:02:02.030

Pa9: help her with you know the respiratory issues that she has. So it doesn't change for me is actually improve her health.

15

00:02:02.850 --> 00:02:03.530

Pa9: Yes.

16

00:02:03.780 --> 00:02:05.540

NM: great, Thank you.

17

00:02:06.880 --> 00:02:10.960

NM: And the last prompt for this question is, how would you?

18

00:02:11.160 --> 00:02:15.880

NM: How do you think physical activity differs from rest for your child?

19

00:02:18.610 --> 00:02:36.310

Pa9: it's actually resting. Well, I think all need rest at certain point. I think this you know, day, time and night time, but I think physical activity give her energy energy to be able to have a strength throughout the day to be more alert.

20

00:02:36.520 --> 00:02:47.880

Pa9: and in her case she starts with physical therapy, her day, and she's been able to have been awake most of the day, like being alert

21

00:02:47.980 --> 00:02:56.220

Pa9: with this routine. So I think it's really important, having a physical activity for energy, energy wise

22

00:02:57.740 --> 00:03:11.470

NM: got it, and just a follow up. You're You're saying that it actually helps her to be. You're saying she gets her physical activity in the morning, and that helps keep her awake. That how does that affect her rest that she give better rest as a result?

23

00:03:11.620 --> 00:03:28.140

Pa9: Yeah, she yes, she actually been awake during the day, helps her rest in during the night, because she used to be wake up during the night a few times, but the day she has, you know, physical activity. She's.

24

00:03:28.140 --> 00:03:33.670

Pa9: you know, waste her energy to all the day. So this way she's she can rest better at night.

25

00:03:34.700 --> 00:03:44.920

NM: Thank you. All right. Question Number 2. What activities would you consider? Your child does his physical activity, and you gave me some already. So if you have any more to add that's great.

26

00:03:45.120 --> 00:03:56.870

Pa9: Yeah. Well, she loves aside of that. She loves being active. She uses a stander. She actually she now is using a gait trainer.

27

00:03:56.900 --> 00:04:07.110

Pa9: She has an adapted tricycle which she loves riding the bike. She also use the exercise ball that also loves

28

00:04:07.310 --> 00:04:16.959

Pa9: and stretching. She also does yoga. She likes doing yoga. So she's pretty active, I think, and she loved it. Yeah.

29

00:04:17.480 --> 00:04:18.810

NM: Oh, awesome.

30

00:04:19.019 --> 00:04:25.540

NM: And you gave me some really nice examples, so I guess I just had questions about.

31

00:04:26.170 --> 00:04:30.980

NM: Do you consider her getting in and out of the wheelchair? Physical activity?

32

00:04:31.790 --> 00:04:34.150

Pa9: Yes, because

33

00:04:34.790 --> 00:04:39.290

Pa9: is it she has to use For example, she helped me.

34

00:04:39.550 --> 00:04:43.440

Pa9: pushing herself up to transfer

35

00:04:43.530 --> 00:05:00.920

Pa9: is an exercise like trying, just you know, trying to get out of the chair. So I think In and out the chair is part of a exercising, I think, is part of our routine, because being in a same position is not good for her

36

00:05:01.050 --> 00:05:08.390

Pa9: for her lungs for her muscles. for anybody being in the same position. It bothers you so I think

37

00:05:08.450 --> 00:05:17.560

Pa9: I think it's it's it's part of an exercise, just, you know, like you sit on and Stand. it's, you know it's kind of an exercise.

38

00:05:18.610 --> 00:05:23.740

NM: And how about being on a playground swing? Does she enjoy that? like an adapted in a playground.

39

00:05:23.740 --> 00:05:39.710

Pa9: Yeah. Well, she has. She doesn't use the playground, I think she out grew the size, and there's unfortunately there's not many, you know, adapted playgrounds.

40

00:05:39.810 --> 00:05:50.000

Pa9: but but she loves all outdoors activities. She really loves outdoors. We we go to parks when you know weather permit.

41

00:05:50.080 --> 00:05:56.800

Pa9: and even put a mat, you know, up like a a blanket on the floor, and she actually has like a hammock.

42

00:05:57.100 --> 00:06:02.540

NM: We lay her down there and switch sides, you know. Feel the breeze

43

00:06:02.720 --> 00:06:07.070

Pa9: by the water, so she's really she really enjoys outdoors.

44

00:06:07.080 --> 00:06:08.300

Pa9: Yes.

45

00:06:09.250 --> 00:06:16.560

NM: and does she? How do? How about your child uses their arms during reaching, or a ball tasks? Do you consider this physical activity?

46

00:06:16.620 --> 00:06:37.790

Pa9: No, she can't reach. She's quadriplegic, but she does do a stretching as part of her daily routine. she enjoys stretching a lot like even when she in the morning. What for getting ready The first thing I do is like. Sit her up by the edge of the bed. And do some, you know.

47

00:06:38.130 --> 00:06:41.860

Pa9: stretching like and she, you know she

48

00:06:42.290 --> 00:06:50.020

Pa9: stretch herself and and take all the laziness out of her to start her day. So that's kind of a routine for her.

49

00:06:50.230 --> 00:07:00.690

Pa9: and midday same. Stretch her because she's been sitting, you know, when she's sitting a long right before an activity. I stretch her

50

00:07:00.690 --> 00:07:10.920

Pa9: so she'd be if she's gonna use on, you know, on an ipad or something for her to be focused. I do a stretching. So she's more alert, you know, more than just that thing.

51

00:07:10.940 --> 00:07:13.490

Pa9: Make her okay. Now i'm ready.

52

00:07:14.900 --> 00:07:19.910

Pa9: And yes, so she she also actually does.

53

00:07:20.270 --> 00:07:30.530

Pa9: She She loves water. So she did aquatic therapy, too, even though she has a ventilator. Now she's able to be for a period of time off.

54

00:07:30.720 --> 00:07:47.230

Pa9: and so she does a quite a bit, you know, just to her waist the what she goes into water. So she does movement inside the water, and she loves it so she's i'm trying to, because I know she. I know how important it is, and I know that she enjoys

55

00:07:47.290 --> 00:07:51.630

Pa9: Physical therapy. I try to get her involved in

56

00:07:51.730 --> 00:07:55.850

Pa9: as many things that she can enjoy, you know, and and get a bunch of

57

00:07:56.380 --> 00:07:57.030

Pa9: yeah.

58

00:07:57.320 --> 00:08:10.190

NM: That's awesome. So you kind of talked about therapy. So how do you feel related services such as you already talked about? P. T. O. T. Vision, hearing, education relate to her physical activity?

59

00:08:10.930 --> 00:08:20.610

Pa9: Yeah, I think, like like I said. not just because of her PT. She's she She has feeding schedule. So

60

00:08:20.750 --> 00:08:28.780

Pa9: that's why we kind of do the physical activity first before lunch, so she is not ready, but that give her also the

61

00:08:29.040 --> 00:08:34.100

Pa9: the energy to be engaged on the other related services.

62

00:08:35.039 --> 00:08:43.169

Pa9: Be, you know, alert for that, and and she's doing well with it. The other, you know, related services. She actually

63

00:08:43.330 --> 00:08:48.850

Pa9: I was working on on her to be able to communicate. She's non-verbal.

64

00:08:49.050 --> 00:09:01.740

Pa9: My concern was her communication, because only kind of me and her develop a a language because she's very expressive. But she's been doing really well with using, eye gaze

65

00:09:01.750 --> 00:09:05.220

NM: and now she's using a Toby.

66

00:09:05.350 --> 00:09:22.040

Pa9: She's learning to use it. She's been a little consistent as we're working on that. so she's able to. She goes twice a week You know, a part of the school. She goes after school to a speech, AT sessions to to

67

00:09:22.140 --> 00:09:32.780

Pa9: learn how to use the Toby and be able to have that as a communication tool. So she's doing pretty good. I'm very happy that you know that she is

68

00:09:33.030 --> 00:09:34.050

Pa9: doing well.

69

00:09:35.240 --> 00:09:39.050

NM: Well, that is really exciting. Let me just say I have to break script.

70

00:09:39.220 --> 00:09:50.610

NM: So would you consider that physical activity for her?

Pa9: Yes I try actually myself, and I was getting frustrated.

71

00:09:50.740 --> 00:09:57.400

Pa9: and I I told her how proud of us of her, because it's really hard.

72

00:09:57.540 --> 00:10:08.990

Pa9: and it takes a lot of energy. She. she, you can tell, you know, at the beginning she gets, you know, a little tired. and then she cannot rest. And then again she tries.

73

00:10:09.130 --> 00:10:25.600

Pa9: you can tell, and the therapies told me that that's normal, that until she gets used to it. But now she's much better. But yes, it takes a lot of energy and concentration, and I try myself, and it is really hard. So.

74

00:10:25.710 --> 00:10:29.980

Pa9: kudos to ‘child’

NM: that is wonderful.

75

00:10:30.010 --> 00:10:37.850

NM: And then does your child do some of these activities alone, or is she in a group with other peers? And then why or why not?

76

00:10:40.010 --> 00:10:49.840

Pa9: No, yes, she is very social. She likes to be around other people, but also. She doesn't like to, you know, to be to, in a place to is too crowded.

77

00:10:49.850 --> 00:10:53.630

Pa9: She yeah, she gets overwhelmed. But the

78

00:10:53.960 --> 00:11:02.120

Pa9: the physical therapy is one to one, but kind of the gym that she attend is open. So she's able to, you know, to be to interact with our kids.

79

00:11:02.350 --> 00:11:06.900

Pa9: and She has group sessions, and one to one.

80

00:11:07.490 --> 00:11:09.720

Pa9: but mostly

81

00:11:10.200 --> 00:11:21.960

Pa9: mostly is a one to one to be able to, you know. Have she has vision and hearing impairment as well. So you know, we try to accommodate the room

82

00:11:22.210 --> 00:11:30.970

Pa9: to be a little darker for her, so in, and things that need to more to be more concentrate for her.

83

00:11:31.110 --> 00:11:34.350

Pa9: We kind of do it like, in a secluded place. But

84

00:11:34.640 --> 00:11:39.170

Pa9: she enjoys like I said, outdoors being around people, so

85

00:11:39.390 --> 00:11:43.480

Pa9: she's really happy when she shows she likes. So

86

00:11:44.100 --> 00:11:46.300

Pa9: she has both

87

00:11:47.090 --> 00:11:51.670

NM: all right. All right. Last question before we get to the survey.

88

00:11:51.690 --> 00:11:59.320

NM: How many times a week. Does your child participate in these activities? And if you could time it, how long is she able to engage?

89

00:11:59.900 --> 00:12:06.480

Pa9: Yeah, she is. She has physical therapy 5 times a week for an hour.

90

00:12:08.060 --> 00:12:18.650

Pa9: depending on the activity and the energy she has that day she is able to do the full hour, or Sometimes, for example, the the stander

91

00:12:18.770 --> 00:12:25.920

Pa9: she's the longest she's been is an hour so 60 mins, so

92

00:12:25.950 --> 00:12:33.670

Pa9: you know. So there's sdays that she's only probably 45 min. There's you know. It depends. But she have daily physical activity.

93

00:12:34.090 --> 00:12:36.120

NM: Yeah, okay, great.

94

00:12:36.220 --> 00:12:54.250

NM: And then does she need assistance Once she's, I mean to set up. I can understand it. But once she's in a position, does she need assistance to perform any of the activities you mentioned standing so she can be so She needs.

Pa9: Yeah, she needs assistance for everything she does.

95

00:12:54.280 --> 00:13:08.040

Pa9: She needs assistance to be transferred to put her AFOs her leg braces on to the position in the equipment that she's gonna use.

96

00:13:08.310 --> 00:13:20.570

Pa9: So she needs assistance for everything. And then, you know, she gets the the ques, what she needs to do. And you know, and she loves to be in. Cheer up so she loves

97

00:13:20.680 --> 00:13:25.330

Pa9: so the the more that you cheer her up she will try harder.

98

00:13:25.520 --> 00:13:33.870

Pa9: So she does her part. but she needs assistance to to position or to put

99

00:13:33.910 --> 00:13:35.360

Pa9: things on for her.

100

00:13:36.590 --> 00:13:46.350

Pa9: But yeah, then she she will be able to do her part.

NM: And do you think she should participate in more or less of these activities and why?

101

00:13:46.940 --> 00:13:56.400

Pa9: I think she should be participating in more. I she. She enjoys it, she enjoys participating on physical activity. She

102

00:13:59.120 --> 00:14:07.280

Pa9: I always we, always she's always monitored. She has a pulse oximeter, and actually she

103

00:14:07.320 --> 00:14:16.560

Pa9: gets off the ventilator during physical activity, which is great before she was doing, using the support now she during that time

104

00:14:16.600 --> 00:14:25.600

Pa9: she's able to. We monitor her closely. She's able to do the physical thing without. So it's really really helped her to improve her

105

00:14:25.740 --> 00:14:36.770

Pa9: her health. and and she enjoys. in the past. She She used her adapted tricycles for race, you know, for walks for close for a short,

106

00:14:36.810 --> 00:14:49.980

Pa9: one and a half mile. I think she did, and I was really nervous, but she did it, and she like I said everybody was, you know, clapping and and and cheering, and she was trying. You can tell how hard she was trying.

107

00:14:50.090 --> 00:14:53.710

Pa9: so she really enjoys physical activity and us

108

00:14:53.800 --> 00:14:57.590

Pa9: as much as I can. I will. I will keep trying to

109

00:14:57.620 --> 00:15:01.460

Pa9: sign her up for as many activities as she can do.

110

00:15:01.760 --> 00:15:03.060

NM: That's awesome.

111

00:15:03.360 --> 00:15:10.680

NM: All right. So let me show you the survey. Yeah, that very helpful. Let's get this one going for you.

112

00:15:12.220 --> 00:15:15.800

NM: And so I again. This survey was developed.

113

00:15:16.260 --> 00:15:21.630

NM: I, the National Institute of Health, and ideally it was created for

114

00:15:21.860 --> 00:15:33.140

NM: parents to report on their child's physical activity, because they either the shop was going through some level of treatments. Ideally it was originally created for children that were going through chemotherapy, and they were regressing.

115

00:15:33.710 --> 00:15:46.300

NM: and so they realized they didn't have really a scale that maybe could ask the parents a parent proxy scale to answer for the child level of physical activity before before. Yeah, actually before Louisiana was

116

00:15:47.840 --> 00:15:53.540

Pa9: right before pandemic she actually was. She had a heart surgery, and she was in

117

00:15:54.870 --> 00:16:04.880

Pa9: in the hospital for 4 months, and she wasn't getting us this as much fiscal therapy or any kind of therapy

118

00:16:05.050 --> 00:16:08.200

Pa9: as she used to, and she really

119

00:16:10.230 --> 00:16:14.600

Pa9: was very the condition. It took a whole year

120

00:16:14.870 --> 00:16:21.260

Pa9: to get her back. And then pandemic came. And actually that year

121

00:16:21.570 --> 00:16:35.000

Pa9: opposite. I think other people that was, you know, that were it was really hard for her, she she was able to recover and be strong enough to be back to school when school reopens. We did all the physical activity here.

122

00:16:35.080 --> 00:16:47.130

Pa9: virtually, with her instructions of the physical therapies. I got all the exercises that I could, and she she recovered, and that's approval, that

123

00:16:47.300 --> 00:16:49.600

Pa9: physical activity really help her

124

00:16:51.320 --> 00:16:52.280

Pa9: improve.

125

00:16:52.330 --> 00:16:57.810

Pa9: She really was able to stand at all was so weak.

126

00:16:57.900 --> 00:17:01.650

Pa9: and you can say like a minimal thing. She was exhausted so.

127

00:17:02.140 --> 00:17:08.109

Pa9: and then she n you can you? It's unbelievable. The how she was and how she is now.

128

00:17:08.430 --> 00:17:26.770

NM: Yeah. okay, so that that's really great to hear. So this this this survey is only 8 questions, and i'm trying to get the parents perspective about how appropriate like, how would you feel about this question if it was asked to you like. Do you think it would be appropriate? And the skill is 0 not appropriate at all?

129

00:17:27.109 --> 00:17:45.300

NM: 5 highly appropriate for someone, a parent of a child that has Cp. Who is not ambulatory similar to your daughter? So the first question is, i'm going to i'm going to ask you to rate the question, and then give me a Tell me why. Okay? So the first question is, how many days your chat exercise your play so hard.

130

00:17:45.300 --> 00:17:51.830

NM: But his or her body got tired. How would you rate this question? 0? Not good at all. 5 highly appropriate

131

00:17:52.060 --> 00:17:53.280

NM: or in between.

132

00:17:54.500 --> 00:17:55.610

Pa9: Hmm.

133

00:17:56.900 --> 00:18:05.710

Pa9: How many days did your child exercise or play so hard that his or her body got tired. How would you rate that question?

134

00:18:05.770 --> 00:18:07.150

Pa9: They all because.

135

00:18:07.420 --> 00:18:08.350

NM: yeah.

136

00:18:08.990 --> 00:18:10.090

Pa9: yeah.

137

00:18:13.640 --> 00:18:22.480

Pa9: Why don't you like that question? I don't know I I don't think it's like so I mean I I I think in my daughter's case is

138

00:18:23.380 --> 00:18:28.380

Pa9: is like she does enough for her to be

139

00:18:28.620 --> 00:18:34.790

Pa9: not. I mean she. She exercise hard, but not as hard to get that so tired as she can.

140

00:18:35.080 --> 00:18:36.740

Pa9: that she can. You know

141

00:18:37.270 --> 00:18:55.490

Pa9: that her body, her or anything. She is hard to, you know she's numberable, but she's very expensive, and you can tell when something is, and then we see that she's that that is a data. She's not feeling well, but she's not. Ha! She doesn't have the strain or anything which is switched to something else less, you know.

142

00:18:55.530 --> 00:18:59.080

Pa9: So I don't. I don't think that yeah.

143

00:18:59.280 --> 00:19:09.800

NM: in her case she never never happened to her before. Got it. and K. That's great. And that next question, how would you rate the on the next number 2?

144

00:19:09.820 --> 00:19:16.270

NM: How many days did your child exercise really hard for 10 min or more? How would you rate that question, and why?

145

00:19:19.880 --> 00:19:20.850

Pa9: I think

146

00:19:21.110 --> 00:19:32.250

Pa9: 5. I think that's perfect. For example, a standard is, or a gate trainer I mean more than the standard, because she's really supported and just bearing her own way. But the

147

00:19:32.750 --> 00:19:39.020

Pa9: gate trainer, I think I i'm seeing her because I you know I see her how hard it is for her

148

00:19:39.120 --> 00:19:45.290

Pa9: just one step, or, you know, and she really had to try hard

149

00:19:45.640 --> 00:19:48.640

Pa9: to the to be, you know.

150

00:19:49.170 --> 00:19:53.390

Pa9: to get tired. But then, you know she rests and recovers.

151

00:19:53.440 --> 00:20:06.480

Pa9: But that is, that is something that you know she really works hard on achieve an exercise that is hard for her. Yeah. So I think that's appropriate. At 5. Let's say, okay.

152

00:20:06.520 --> 00:20:08.650

NM: Great. All right. Number 3.

153

00:20:08.680 --> 00:20:17.990

NM: How many days your child exercise so much that he or she breathed hard. How would you rate this 1? 0? Not applicable? 5 appropriate

154

00:20:18.120 --> 00:20:19.180

NM: we in between?

155

00:20:19.680 --> 00:20:28.340

Pa9: Yeah, maybe 4. Yeah. In her case, is she like I said, she's on a ventilator. So

156

00:20:28.580 --> 00:20:30.400

Pa9: we try to.

157

00:20:30.600 --> 00:20:39.230

Pa9: I mean, when you exercise, I think normally you start reading past there because you're exercising. So that's a I think.

158

00:20:39.320 --> 00:20:42.490

Pa9: but it's not that she's like, you know.

159

00:20:42.950 --> 00:20:46.970

Pa9: breath. It's just that she's agitated because she's exercising so all.

160

00:20:47.880 --> 00:20:55.410

Pa9: Maybe Why, in her case it might be a. Probably because she has a venture and we take it off to exercise. So maybe

161

00:20:55.480 --> 00:20:57.450

NM: yeah, okay.

162

00:20:58.180 --> 00:20:58.920

NM: right.

163

00:21:06.980 --> 00:21:14.080

NM: All right. Number 4. How would you write this question. How many days was your child so physically active that he or she sweated.

164

00:21:16.530 --> 00:21:22.670

Pa9: Hmm. I think for it. She's she sweats when she

165

00:21:22.710 --> 00:21:32.010

Pa9: I only the weather. I guess the the how the place the environment is. But it's a hot day. Yeah, she sweats. She sweats.

166

00:21:32.250 --> 00:21:37.790

Pa9: Yeah, if you if she exercise yeah, she will. You know she's

167

00:21:37.900 --> 00:21:41.860

Pa9: doing something hard as she will sweat, She will. Okay, yeah.

168

00:21:44.440 --> 00:21:45.220

NM: all right.

169

00:21:45.650 --> 00:21:48.400

NM: And number 5.

170

00:21:48.720 --> 00:21:54.310

NM: How many days your child exercise a place of heart that his or her muscles burned. How would you read that one?

171

00:21:59.090 --> 00:22:08.030

Pa9: I would say 2? I don't really. You know she's consistent and exercising all, you know, every day, so I think you mostly

172

00:22:08.720 --> 00:22:12.790

Pa9: burn your muscles when you are not exercising us

173

00:22:12.990 --> 00:22:16.330

Pa9: often, I guess.

174

00:22:16.480 --> 00:22:21.000

Pa9: Yeah. So she's. Yeah, she. I I never seen him like.

175

00:22:21.080 --> 00:22:25.220

Pa9: I never seen it before in pain because of exercise. So

176

00:22:25.360 --> 00:22:25.970

Pa9: yeah.

177

00:22:26.330 --> 00:22:28.130

NM: that's important to know. Yeah.

178

00:22:31.900 --> 00:22:39.370

NM: Number 6. How many days your child exercise a play so hard that he or she felt tired.

179

00:22:40.400 --> 00:22:46.750

Pa9: I was like for yeah, she like, I said, the gate plane, and it's something that really takes her

180

00:22:46.850 --> 00:22:59.940

Pa9: her energy out. It's hard for her. She wasn't using it before. So it's something that She' to do, and and he gets it. It takes her, you know. It makes her tire when she tries really hard.

181

00:23:00.030 --> 00:23:13.410

Pa9: but nothing that it will, you know. It will make her t the whole day she will rest and recover. It's like I said. It's during the day that I mean first thing in the morning. So this way she has time to rest a little bit and

182

00:23:13.630 --> 00:23:15.390

Pa9: get ready for the rest of the day.

183

00:23:15.600 --> 00:23:16.460

NM: Got it

184

00:23:17.690 --> 00:23:22.920

NM: all right. Good, and number 7 is how many days was your child physically active for 10 min or more.

185

00:23:23.180 --> 00:23:31.720

Pa9: How would you write this 1? 0. Not appropriate? 5 highly appropriate. And why? 5? Yeah. She's actually more like 5 days a week.

186

00:23:33.380 --> 00:23:38.020

Pa9: It's harder on the weekends because we don't have. You know, the all the equipment of

187

00:23:38.630 --> 00:23:46.260

Pa9: but you know she does she still that stretching during the weekends and and breaks so she's

188

00:23:46.570 --> 00:23:50.360

Pa9: mostly, if physically, up to every day.

189

00:23:50.740 --> 00:23:55.220

NM: No, I think my question, though. Do you think this is a question to ask other parents like you.

190

00:23:55.230 --> 00:23:56.360

Pa9: Oh, okay.

191

00:23:56.490 --> 00:24:04.450

NM: yeah, like. So I'm asked you to rate these questions like, what? The how would you rate these in terms of what's important to ask the parents.

192

00:24:04.780 --> 00:24:09.280

Pa9: Yeah, I think it's important to have the point, because because

193

00:24:09.510 --> 00:24:18.380

Pa9: it's important for the kids to be active. So if that if they, you know, they can question themselves if my kid is active enough.

194

00:24:18.960 --> 00:24:36.030

NM: Okay. So some questions may be better than others. So i'm trying to get an idea of which questions that are so, the one setting goal. Yeah, for example, this one is good because it makes you. You have the perspective of is my child active enough or not, you know. Okay, that's great.

195

00:24:36.420 --> 00:24:37.140

Pa9: Yeah.

196

00:24:42.460 --> 00:24:44.660

NM: And then the last one

197

00:24:45.510 --> 00:24:55.600

NM: Number 8 is how many days your child run for 10 min or more. How would you? How appropriate is this question for the No. I don't think so, probably because if this

198

00:24:56.630 --> 00:25:04.640

Pa9: we're in in specifically, my daughter. She's quite a plagic. So most of the see P

199

00:25:06.020 --> 00:25:15.730

Pa9: children are. Yeah, I can run, I think. There's equipment, but I think it's it's very limited. So I don't think it's appropriate.

200

00:25:15.790 --> 00:25:17.070

Pa9: Yeah.

201

00:25:19.650 --> 00:25:24.470

NM: Okay. So we are wrapping up. And what I like to do is ask the parents for any

202

00:25:24.600 --> 00:25:42.340

Pa9: final thoughts or comments related to physical activity for children with Cp. Who are not full time walkers. Anything you you would like to share, just to be, to not give up on advocating for physical therapy, I think, is really important. Makes

203

00:25:42.780 --> 00:25:53.170

Pa9: the child life easier, and the pine life easier because it helps you. You know They're not going to be a child. The whole life they grow, and the

204

00:25:54.960 --> 00:26:00.260

Pa9: you know we have to. We are caregivers and physical activity helps both the

205

00:26:00.510 --> 00:26:08.500

Pa9: your child, your child, and your and yourself as a parent. So I think it's really important, improved her their health.

206

00:26:10.250 --> 00:26:18.980

Pa9: and I think they mostly enjoy exercise. Well, there's people that that doesn't enjoy exercising. But I think in my particular I go to that.

207

00:26:19.160 --> 00:26:23.060

Pa9: and we have how many that enjoy physical activity. So

208

00:26:23.290 --> 00:26:27.420

Pa9: it keeps your mind fresh if you energy, and I think

209

00:26:29.020 --> 00:26:42.310

Pa9: it's really important. Physical care is very important. Part of the Cp. Child, children, so it's this: that's Don't Don't stop applicating for that.

210

00:26:43.910 --> 00:26:51.780

NM: That's awesome. Don't. Stop all right. Thank you so much. I'm going to stop the recording now.

211

00:26:53.170 --> 00:26:54.670

NM: and.
