# Supplementary material for: Validity of PROMIS® Pediatric Physical Activity Parent Proxy Short Form Scale as a Physical Activity Measure for Children with Cerebral Palsy Who Are Non-Ambulatory
Source: Behav Sci (Basel). 2025 Jul 31;15(8):1042. doi: 10.3390/bs15081042 (PMC12382615; doi:10.3390/bs15081042)
Supplement: Supplementary file 1 [file behavsci-15-01042-s001.zip › Transcripts copy/Parent transcripts de-identified/Pa12.docx]

WEBVTT

1

00:00:01.250 --> 00:00:16.840

NM: All right. So good evening. Thank you so much for meeting with me today to talk about physical activity for children that with Cp. We are not full time walkers. I do want to let you know that if I sound scripted is because I am because I have to be as consistent as possible

2

00:00:16.840 --> 00:00:27.910

with our interview questions. The first half of this interview will be basic questions about how you view physical activity as it relates to your child, and then the second app is only a survey

3

00:00:28.010 --> 00:00:40.030

NM: that was developed by the National Institute of Health for children that were not typically developing. And I really want to get parents feedback about each question. How appropriate would you feel? This question is

4

00:00:40.040 --> 00:00:50.860

NM: for a parent of a child with Cp. That does not walk. Okay and well, I have you great. I explain that in a moment. So for the first question, how do you define physical activity for

5

00:00:51.020 --> 00:00:52.120

NM: your child?

6

00:00:54.820 --> 00:00:57.620

Pa12: For my child. I define it as

7

00:00:57.720 --> 00:01:14.000

Pa12: whatever makes her comfortable. So I see it as where you know she's not she's not mobile. She She uses a wheelchair for most of her activities, whether it be wheelchair or any other equipment, we have to help her either walks, stand

8

00:01:14.090 --> 00:01:18.550

Pa12: transfer from the shower to bed.

9

00:01:18.640 --> 00:01:21.070

Pa12: But what I found is that with my child

10

00:01:21.120 --> 00:01:37.840

Pa12: she likes to move her legs a lot like whether she's in a lying position, sitting position, a standing position she constantly tries to mobilize her legs. So I've in any, on any equipment that she has been assigned to.

11

00:01:38.060 --> 00:01:42.750

Pa12: I try to make sure that her legs are not restricted, because that is where she mostly

12

00:01:42.960 --> 00:02:00.920

Pa12: finds comfort, I guess, to relieve, you know, strain of muscle tension, or, you know, just expressing herself. So I try my hardest to not restrict an areas that I know she expresses herself with. So, her leg her legs being that for for her

13

00:02:03.760 --> 00:02:18.620

NM: that's great. Thank you. And then the next question. It's more of a prompt related to the first question. The Department of Health defines physical activity as any activity that encompasses energy, expenditure, and activation of skeletal muscle.

14

00:02:18.650 --> 00:02:24.020

NM: Does that definition change your mind about how you define physical activity for your child? Why or why not?

Pa12: What was the first one?

15

00:02:25.340 --> 00:02:41.590

NM: I'm sorry the department of health defines physical activity as any activity that encompasses energy expended, and activation of her muscles. Does this definition. Change your mind is how you define physical activity for your child?

16

00:02:42.030 --> 00:02:42.650

No.

17

00:02:43.960 --> 00:02:47.540

NM: And then how do you think physical activity differs from rest?

18

00:03:00.020 --> 00:03:02.310

Pa12: I believe

19

00:03:04.160 --> 00:03:06.330

Pa12: physical activity differs from rest?

20

00:03:07.050 --> 00:03:10.390

Pa12: So for a child like mine.

21

00:03:10.690 --> 00:03:17.580

Pa12: whether she's in a resting state or no physical activity state, I feel like she she's worked hard because it

22

00:03:18.220 --> 00:03:37.660

Pa12: it's not. It's not. She's not highly physically active right? So whether she's resting or in a physical state, she's still using a lot of energy to do the minimum of movements, or even resting, being able to rest, and and actually have a a good rest meaning like

23

00:03:37.800 --> 00:03:52.190

Pa12: if she you know she's experiencing some type of pain and it and i'm not aware of that. She has to try to communicate that with me. So whether it be by trying to move her arm to show me, or you know

24

00:03:52.570 --> 00:03:56.890

Pa12: those, I find that you know she can be an arresting state, and still, like

25

00:03:58.390 --> 00:04:01.650

Pa12: exhaust her energy. If that makes sense.

26

00:04:02.100 --> 00:04:08.100

Pa12: that does. Thank you. Well, sir, I'm my own. Yeah.

27

00:04:08.390 --> 00:04:15.030

NM: So she may be in a resting state. I'm gonna make sure. Yeah. And still expending energy.

28

00:04:15.370 --> 00:04:16.370

Pa12: Yeah.

29

00:04:18.510 --> 00:04:31.130

NM: Okay. And now we're on to the second question. What activities would you consider your child does as physical activity? So you mentioned her leg movement. Is there anything else that you would say that you consider physical activity for your child?

30

00:04:32.300 --> 00:04:34.230

Pa12: Yeah.

31

00:04:35.100 --> 00:04:41.880

Pa12: She just recently started using an eye gaze machine. So I think that that is considered

32

00:04:42.300 --> 00:04:45.860

Pa12: physical activity for her, because she has to, you know.

33

00:04:45.970 --> 00:04:52.670

Pa12: move her eyes to, you know. Have the the machine track her, her

34

00:04:52.680 --> 00:04:55.060

Pa12: selections of whatever sort.

35

00:04:59.520 --> 00:05:02.060

Pa12: What are the I mean?

36

00:05:02.240 --> 00:05:03.710

Pa12: I don't know what other.

37

00:05:03.900 --> 00:05:20.240

NM: No, that's okay. I have a prompt for for the next part of this question. So for if unsure let's discuss some of her regular day or habitual activities, such as using adaptive equipment, would you consider the use of a stander or a gait trainer as physical activity.

38

00:05:20.790 --> 00:05:22.540

Pa12: Yes.

39

00:05:22.630 --> 00:05:29.400

Pa12: Yeah. So yes, her Toby device, her stander, her gait trainer.

40

00:05:30.590 --> 00:05:33.470

Pa12: her switches. She uses head switches

41

00:05:35.910 --> 00:05:45.550

Pa12: even when she's in her like AFOs or her hand brace. You know these are things that she's using her muscles, or even sometimes trying to get out of ‘cause

42

00:05:45.690 --> 00:06:00.050

Pa12: like my daughter doesn’t even like to have socks on. So she takes her socks off her feet, and all these things like again, if she's uncomfortable about something she tries. Oh, another thing that she does so she's sitting in her wheelchair

43

00:06:00.100 --> 00:06:06.560

Pa12: Many of times we've had to replace the like the neck part of her wheelchairs.

44

00:06:06.570 --> 00:06:18.170

Pa12: because she kicks to the point where she breaks that part of the wheelchair. So again she through to trying to communicate. She also is physical in trying to get her message across

45

00:06:19.630 --> 00:06:22.380

NM: What part of the wheelchair that you that she tries with the kick.

46

00:06:23.660 --> 00:06:27.850

Pa12: So she has the the foot rest

47

00:06:27.960 --> 00:06:32.970

Pa12: all the time that she's had to again whenever she's been restricted, whether it be by a belt.

48

00:06:33.260 --> 00:06:45.200

Pa12: the belt break, or you know. So it' be to the point that she has enough force to break the actual stand, or, like a piece of it, will chip off, and then I have to replace it, because

49

00:06:45.840 --> 00:06:48.190

Pa12: she just hates it.

50

00:06:48.420 --> 00:06:51.230

NM: She wants her legs free.

51

00:06:52.140 --> 00:06:52.740

NM: right

52

00:06:52.900 --> 00:06:58.140

NM: That's great. Does she enjoy? Does she still get on a playground swing on like adaptive playground?

53

00:07:01.170 --> 00:07:12.100

Pa12: She does. I I won't say a lot. She she she She has gotten better with the playground swings before we couldn't put her on because she she would her her

54

00:07:12.220 --> 00:07:24.080

Pa12: the balance, and she wouldn't. She wouldn't be able to focus. Now she's a little bit more where she can focus, and you know the movement of the swinging doesn't bother her or trigger her to go into a spastic state.

55

00:07:24.120 --> 00:07:34.020

Pa12: so she's gotten better with that with, and I feel that came with age, like as she got older, because when she was younger you couldn't. We couldn't even take her in the car because

56

00:07:34.030 --> 00:07:40.080

Pa12: she couldn't she No, she wouldn't be able to focus on certain things, and it would just blow her off completely.

57

00:07:41.400 --> 00:07:42.150

NM: Gotcha.

58

00:07:43.020 --> 00:08:00.760

Pa12: I just fount like repeating like repeating things. So a swing is something we Won't repeat, to do every day. So I find that certain certain activities that are repeated, or or you know, routines are repeated. You know she she'll get familiarized with them, and it won't be something new to her, as you know. A swing will do like

59

00:08:00.780 --> 00:08:10.090

Pa12: once a month, if that like. If it's us taking her to the park. But in school I know she does. She has used like the swings with therapist but

60

00:08:10.150 --> 00:08:14.670

Pa12: she doesn't like sudden movement that my daughter doesn't like setting movement. Yeah.

61

00:08:18.070 --> 00:08:26.090

NM: And then what about adaptive reaching or ball toss? Would you consider that physical activity?

62

00:08:26.530 --> 00:08:30.580

Pa12: Yes, hand over hand a lot, so

63

00:08:30.610 --> 00:08:49.390

Pa12: I believe her therapist work with her. As do us as her parents. You know it's a lot of handle hands, so you would think that she's not exhausting her energy, but she is because it's a stretch, and we have to put her, you know. There's times that she would retrack her arm due to her spasticity. So

64

00:08:49.470 --> 00:08:56.670

Pa12: just stretching on her arms sometimes just to release a ball to whether it's bowling, or you know any other

65

00:08:57.610 --> 00:08:59.850

Pa12: play playful ball activities.

66

00:09:00.580 --> 00:09:01.410

NM: I didn't

67

00:09:02.770 --> 00:09:13.920

NM: all right. And how does related services such as physical activity? I mean physical therapy, occupational therapy vision, hearing, or whatever she receives. Speech relates to physical activity for her.

68

00:09:15.210 --> 00:09:19.130

Pa12: How does how do they relate to physical activity for her?

NM: Yeah.

69

00:09:22.350 --> 00:09:31.000

Pa12: So for physical therapy, like we mentioned before, she uses the gait trainers, the standers, the you know

70

00:09:31.300 --> 00:09:44.420

Pa12: her AFOs you know the ball to stand, In vision, She uses her Toby device, or even when reading a book. You know she uses her switches

71

00:09:46.130 --> 00:09:54.610

Pa12: for speech the same thing. They use the switches for her to choose, you know, when she's communicating with the therapist

72

00:09:55.170 --> 00:09:58.920

Pa12: something as simple as reading a book to help turn a page, or.

73

00:09:58.970 --> 00:10:17.280

Pa12: you know, let them know she she's her. She went to the bathroom so her diaper might need to be changed. So it's all again. It's like brain to, you know, sending the message from the brain to actually have her, whether it be vocally like, just by a a, a discomfort, and and

74

00:10:17.390 --> 00:10:23.810

Pa12: a prompt from her mouth to something where she, might lift her bottom up off either her seat, or

75

00:10:23.850 --> 00:10:32.040

Pa12: if she's laying down on a on the mat or something, she doesn't like to be sitting in anything that makes her uncomfortable. So she's able to communicate that

76

00:10:32.370 --> 00:10:33.360

NM: that right. And so and does she get hearing? I don't think she does

77

00:10:34.000 --> 00:10:38.170

Pa12: No.

78

00:10:38.630 --> 00:10:48.100

Pa12: all right, and he just recently started and needs glasses, so she just recently got some glasses for the first time he actually so cute in them.

79

00:10:48.160 --> 00:10:51.470

NM: Oh, my God! It's seriously that I am sure she does

80

00:10:52.830 --> 00:10:58.370

NM: And does she do these activities in a group, or is it alone with the therapist

81

00:10:58.620 --> 00:11:14.630

Pa12: one to one when when for her in school a para is those are a para, and with the therapist, and then they also have push in sessions with other children, whether it be when they're having active in an academic section session.

82

00:11:14.630 --> 00:11:21.600

Pa12: or they'll bring them in for like music, therapy together, or physical like throwing ball to each other.

83

00:11:22.300 --> 00:11:32.130

Pa12: Those are things that I've witnessed when I've gone to her school and home at home. She has. We have relatives of cousins and stuff that come over, and

84

00:11:32.630 --> 00:11:44.880

Pa12: they don't even see my daughter's disability it's not. It's non-existent. So they treat her, and she gets annoyed. But I allow it because it is what it is.

85

00:11:46.080 --> 00:11:53.190

NM: And okay last question before survey. How many times a week does your child participate in these activities, and for how long

86

00:11:53.850 --> 00:11:55.440

Pa12: she

87

00:11:55.800 --> 00:12:00.510

Pa12: is supposed to receive an hour of each service 5 days a week.

88

00:12:00.590 --> 00:12:04.140

Pa12: So she received physical, occupational

89

00:12:04.380 --> 00:12:05.740

Pa11: vision.

90

00:12:05.860 --> 00:12:10.790

Pa12: speech and adaptive technology therapy.

91

00:12:11.210 --> 00:12:11.880

NM: Hmm.

92

00:12:13.280 --> 00:12:18.500

NM: And is she able to endure the whole hour? Would you say, or does she need breaks or…?

93

00:12:18.600 --> 00:12:34.220

Pa12: so? What's what's most for her is the actual like, maybe a half hour before therapy to kind of loop her up and get her ready. So she does use the whole hour, because it takes so much to get into therapies

94

00:12:34.220 --> 00:12:38.720

Pa12: to have her actually engage with each therapy she participates in

95

00:12:41.820 --> 00:12:42.620

NM: it's great.

96

00:12:42.850 --> 00:12:59.500

NM: And does she get assistance with these activities, and that is there part of any of the activity where they may set her up, and then she completes it, or does she? Does she get assistance for the entire activity? She needs assistance for the entirety of all activities?

97

00:12:59.800 --> 00:13:04.500

Pa12: She needs assistance for the entirety of all activities. She's fully dependent on either myself, her para or therapist

98

00:13:06.110 --> 00:13:11.420

NM: with the Toby though. how many commands is she able to to do on her own? With the eye gaze?

99

00:13:12.240 --> 00:13:16.170

Pa12: I have witnessed her. She does it. Oh.

100

00:13:16.330 --> 00:13:25.830

Pa12: you want the number you want the she she she she knows. Yes, no, that's hello, Goodbye, Good morning.

101

00:13:28.680 --> 00:13:30.010

Pa12: More

102

00:13:30.300 --> 00:13:31.820

Pa12: turn, Page.

103

00:13:34.760 --> 00:13:39.990

Pa12: I think those are the ones i'm familiar with. I'm not like I I have to. I haven't had full.

104

00:13:40.000 --> 00:13:47.260

Pa12: I haven't had a chance to sit and actually engage with her with the Toby with her. But when I do, she's gonna know it all.

105

00:13:47.550 --> 00:13:54.220

NM: That's awesome. It's just wonderful. And do you think she should participate in more or less of these activities. And why?

106

00:13:55.750 --> 00:13:59.470

Pa12: I think more because the more she gets, and

107

00:13:59.660 --> 00:14:13.020

Pa12: the more she gets, and how frequent she gets it, she retains it better. So I feel more is better. I rather her get more and be tired and not get it at all, and not have the ability to

108

00:14:13.790 --> 00:14:18.980

Pa12: fulfill any task that she may need to fulfill any tasks with those therapies that she receives.

109

00:14:20.930 --> 00:14:27.270

NM: That's all right. So now I'm going to share my screen and show you this survey. Now I didn't write the survey.

110

00:14:27.320 --> 00:14:44.470

NM: Let me say that. But it is a survey that was developed, I think specifically for children that were regressing. Due to cancer is what this survey was created for. So it was due to cancer. You said Yes, they would. They initially the first work they got this survey were for parents

111

00:14:44.470 --> 00:14:59.190

NM: to answer about physical activity for kids that were regressing. So they were losing their functioning and just wanting the parent to report on how active they were the week before. So the reason I I have this one is because it's really not that many options

112

00:14:59.310 --> 00:15:06.940

NM: for children to have. Cp. That are not full time. Walkers so really want to ask the parents to grade, not to answer the question for your child.

113

00:15:07.370 --> 00:15:22.100

NM: but for you to tell me how appropriate you think. This question is, as it relates to your family, your child, and give me a score of rating from 0, not appropriate at all. 5 highly appropriate. And then I want you to tell me why.

114

00:15:22.430 --> 00:15:33.390

NM: Okay? Alright, so let's start with the first question. How would you rank this question? How many days is your child exercise or play so hard that his or her body got tired?

115

00:15:33.620 --> 00:15:38.320

NM: 0? Not appropriate at all 5 highly appropriate. And why?

116

00:15:40.460 --> 00:15:44.430

Pa12: How many days? Your child?

117

00:15:45.450 --> 00:15:46.410

Pa12: I think

118

00:15:47.600 --> 00:15:53.800

Pa12: 5 highly appropriate. Okay.

119

00:15:57.480 --> 00:15:59.790

Pa12: Because you're identifying

120

00:16:00.950 --> 00:16:19.170

Pa12: the child. Play hard, and then you would also want to know why, right? The reason for the the by, why the body got tired and address that, because it might be something that is concerning you know, it could be something medical that you want to. You want to address before it becomes a long term issue.

121

00:16:19.190 --> 00:16:20.170

Pa12: I would

122

00:16:20.300 --> 00:16:21.000

think

123

00:16:21.080 --> 00:16:22.020

NM: that's good.

124

00:16:22.890 --> 00:16:25.620

NM: Thank you. Okay, Number 2.

125

00:16:26.340 --> 00:16:31.930

NM: How many days is your child exercise really hard for 10 min or more? How would you rate this question?

126

00:16:34.740 --> 00:16:36.460

Pa12: And

127

00:16:42.190 --> 00:16:44.190

Pa12: for 10,

128

00:16:46.030 --> 00:16:51.770

Pa12: I mean, I would say it's an appropriate question to again. It it's another way to assess.

129

00:16:52.860 --> 00:16:53.440

But

130

00:16:53.600 --> 00:16:54.750

Pa12: your child.

131

00:16:54.860 --> 00:16:58.320

Pa12: I mean.

132

00:17:00.870 --> 00:17:12.319

Pa12: how would you? What number would you give it. Oh, I said it 5. I'm sorry I said 5 I it is. It's just another tool to be able to assess your child. I would I would suggest.

133

00:17:14.130 --> 00:17:14.890

NM: hey, Great

134

00:17:15.140 --> 00:17:18.329

NM: number 3, and that in the language is appropriate.

135

00:17:19.930 --> 00:17:30.600

Pa12: So when you first read it to me, my first question came was, Why, Why, why is really hard like emphasize, I guess. But you know for me that's just

136

00:17:30.810 --> 00:17:40.020

Pa12: I could like when I was repeating it to myself, and when you read it the first time I was like, what does it say? Really hard? But that's the only part that kind of like had me like questioning the question

137

00:17:40.530 --> 00:17:54.580

NM: that that's that's important information. Thank you. Like, yeah, I just like to get that feedback she had to like, do a double take like I did I did a double take, and then I, when I read it back again, I I was like, what is it really hard like

138

00:17:54.680 --> 00:17:55.300

Pa12: this?

139

00:17:55.510 --> 00:17:57.570

NM: So what a definition be helpful.

140

00:17:58.030 --> 00:18:07.710

Pa12: How many busy your child as well like what really hard means? Maybe, yeah, like like maybe that. But then I guess I guess the purpose. For the person who

141

00:18:08.630 --> 00:18:11.770

Pa12: I had. This question was just a good

142

00:18:11.800 --> 00:18:13.830

Pa12: to the point, I guess. But

143

00:18:14.380 --> 00:18:24.710

Pa12: okay, cause you would question like, what is really hard like that's I. I had to repeat the question to myself for that that that that just it out to me, and not because it's underlying it's because

144

00:18:24.850 --> 00:18:27.810

Pa12: it's really hard. Yeah, yeah.

145

00:18:27.860 --> 00:18:30.260

NM: Got you all right. Number 3.

146

00:18:30.390 --> 00:18:39.720

NM: How many days your child exercise so much that he or she breathes hard? How appropriate or valid is this question for children that are not full time walkers in your opinion.

147

00:18:48.980 --> 00:18:49.600

Pa12: she

148

00:18:52.320 --> 00:19:00.040

NM: and remember, this is for physical activity, intensity. So we're looking at the level of intensity, they participate in activity over the week.

149

00:19:02.680 --> 00:19:10.790

Pa12: So again I it I I say 5, because again it's all. It all falls into assessing the child. So I don't see it as a negative.

150

00:19:13.840 --> 00:19:15.050

Pa12: Yeah, 5,

151

00:19:15.150 --> 00:19:22.780

NM: Okay. And number 4. How many days was your child so physically active that he or she sweated

152

00:19:30.550 --> 00:19:36.100

Pa12: You said the 0 is not important or not not not appropriate for for this population like

153

00:19:37.160 --> 00:19:52.310

Pa12: not really yeah, 0 0, just because I know it's the the amount of activity doesn't that like sweat is not something that's common for a child like mine like that's not gonna it's not gonna it's not gonna

154

00:19:53.410 --> 00:19:56.740

Pa12: scale anything for me like it's not gonna bring, you know.

155

00:19:57.480 --> 00:20:05.180

Pa12: I don't even think I've ever seen her split that crazy as well for me wouldn't apply. That's why I would give it a 0.

156

00:20:05.290 --> 00:20:06.290

NM: Yep. Got it

157

00:20:06.590 --> 00:20:08.030

that's helpful. Thank you.

158

00:20:08.220 --> 00:20:17.000

NM: Alright, bye. Bye. how many days is your child exercise a place so hard that his or her muscles bond. How would you rank that one?

159

00:20:17.220 --> 00:20:19.990

Pa12: 0? Because I wouldn't know how to answer that

160

00:20:20.350 --> 00:20:23.700

NM: because my child wouldn't be able to answer the question herself.

161

00:20:32.600 --> 00:20:40.210

Pa12: I guess that it would be 0 for a nonverbal child. I would. I don't know how it would be for a child who can speak, or, you know.

162

00:20:41.930 --> 00:20:42.680

NM: got it.

163

00:20:44.930 --> 00:20:53.210

NM: and number 6. How many days your child exercise a place so hard that he or she felt tired. How would you break that one?

164

00:20:53.830 --> 00:20:54.550

Pa12: Okay.

165

00:21:02.220 --> 00:21:12.080

Pa12: I can do that. A 5, because it's something I I would be able to answer. because she would fall as if she was exhausted. Yeah.

166

00:21:16.230 --> 00:21:21.190

NM: and number 7. How many days was your child physically active for 10 min or more?

167

00:21:21.410 --> 00:21:22.560

Pa12: Alright?

168

00:21:22.700 --> 00:21:23.480

NM: Yeah.

169

00:21:24.270 --> 00:21:35.310

Pa12: you need a reason for that as well or no. I mean, is it for the same reason you mentioned before? Yeah, because that it's just a simple ask and answer, type, question to take from it.

170

00:21:39.320 --> 00:21:40.140

NM: And

171

00:21:40.310 --> 00:21:46.490

NM: and the last one number 8. How many days did your child run for 10 min or more

172

00:21:48.470 --> 00:21:50.980

Pa12: because it doesn't apply.

173

00:21:51.220 --> 00:21:51.860

NM: Yeah.

174

00:21:52.680 --> 00:22:04.070

NM: And as we wrap up, I like to ask all my parents for their final thoughts and comments as it relates to physical activity in this population any that you want to share, as we wrap up

175

00:22:07.830 --> 00:22:14.260

Pa12: in this population when it comes to physical activity, I would say, is, you know.

176

00:22:14.670 --> 00:22:19.200

Pa12: I don't from for me and my child. I don't limit her to.

177

00:22:19.440 --> 00:22:28.720

Pa12: I don't put. I don't set any limitations for her like she she expresses herself her own way, and i'm here to facilitate, however way she is

178

00:22:28.840 --> 00:22:42.690

Pa12: capable of expressing herself. So if we're at a park, and there should, there are children running all around her. I'm not gonna have her feed it in her wheelchair, watching those children. I'm going to physically remove her from her chair.

179

00:22:42.850 --> 00:22:46.980

Pa12: and if as much as it may hurt me the next day or that evening

180

00:22:47.010 --> 00:22:57.800

Pa12: I'm gonna, you know, move her legs and run right with the children and have her, you know, feel included the same goals, for if we're at a

181

00:22:58.090 --> 00:23:16.650

Pa12: birthday party where it's like a Trampoline Park, you know, if kids are all around jumping around her. I'm not going to keep her, you know. I'm not going to limit her to her wheelchair. She's gonna get up, you know. I'm gonna move. Remove her. I'm gonna transfer it to the Trampoline, where whether it's her lane there or me sitting there with her, and we'll bounce on it. So just you know I don't

182

00:23:16.750 --> 00:23:21.370

Pa12: for me. It's more like not having her identify with

183

00:23:21.800 --> 00:23:25.120

Pa12: her equipment. That's not her. She is a a full

184

00:23:25.370 --> 00:23:35.000

Pa12: grown person. At this point. She's an older child now, and you know she's here to experience life the way everyone else is, and and i'm Here

185

00:23:35.280 --> 00:23:47.680

Pa12: I was chosen to to facilitate that for her. So that's what i'm going to consider. I've been doing it, and and her father and I are going to continue doing it. So yeah, just I don't again. No limitations. We're here to live life.

186

00:23:47.980 --> 00:23:48.950

Pa12: So the for this

187

00:23:51.020 --> 00:23:58.050

NM: sound like, an I like that. I'm just thank you so much. I must stop the recording. That was.
