# Supplementary material for: Validity of PROMIS® Pediatric Physical Activity Parent Proxy Short Form Scale as a Physical Activity Measure for Children with Cerebral Palsy Who Are Non-Ambulatory
Source: Behav Sci (Basel). 2025 Jul 31;15(8):1042. doi: 10.3390/bs15081042 (PMC12382615; doi:10.3390/bs15081042)
Supplement: Supplementary file 1 [file behavsci-15-01042-s001.zip › Transcripts copy/Parent transcripts de-identified/Pa5.docx]

WEBVTT

1

00:00:01.380 --> 00:00:16.400

NM: All right. Good morning. Thank you so much for joining us today. Today we're going to talk about physical activity for children with who are not full time walkers specifically in Gmf. Cs levels 4 and 5. I'm. Going to ask you a few questions, and then I have some prompts after each question.

2

00:00:16.400 --> 00:00:23.200

That's the first half of the interview, the second half of the interview. I'm going to share a survey that was created for parents to

3

00:00:23.490 --> 00:00:36.030

NM: report on the physical activity of their child for the week prior to assessing them. And so I will ask you questions about that survey for the second half of this time. So first question I ready to begin.

4

00:00:38.790 --> 00:00:44.730

NM: Yes, okay. First question. How do you define physical activity for your child?

5

00:00:48.460 --> 00:00:54.570

Pa5: I can define it as scooting around, crawling up on the couch.

6

00:00:55.080 --> 00:00:57.650

Pa5: rolling into her bed.

7

00:01:01.580 --> 00:01:07.860

Pa5: but mainly, you know, not using her legs and just a little bit of her arm.

8

00:01:08.510 --> 00:01:11.500

Pa5: But

9

00:01:12.020 --> 00:01:14.150

Pa5: Yeah, that's a tough one NM.

10

00:01:14.850 --> 00:01:19.570

Pa5: being pushed around in her in her wheelchair.

11

00:01:21.110 --> 00:01:24.670

NM: Now you're breaking up. I don't know if it's me, or if it's the phone

12

00:01:24.700 --> 00:01:29.520

Pa5: do you have Wi-fi at the house? Yeah, I don't know if it's me or you i'll move into

13

00:01:29.690 --> 00:01:38.290

Pa5: I do. I'll move into a different room. Let's see if that works. I I didn't hear a couple. I mean, I knew what you were saying, but I didn't hear a couple of words.

14

00:01:38.670 --> 00:01:39.450

Pa5: Okay.

15

00:01:39.760 --> 00:01:41.840

NM: Now, that sounds better actually, already.

16

00:01:41.900 --> 00:01:42.650

Pa5: Okay.

17

00:01:42.940 --> 00:01:46.150

NM: all right. So you said, scooting around, crawling around in and out of bed

18

00:01:46.280 --> 00:01:54.060

NM: But mainly, you said, but mainly not using her legs where I lost you in arms. So she, when you were you were saying, using the legs and arms.

19

00:01:54.600 --> 00:02:06.620

Pa5: Yeah, yeah, not so much Her legs but her arms, unless you know, like we have her, or if it's on. But still, then you know, she's walking with a lot of support. But if you're saying like, what's her physical activity? If she's just on her own.

20

00:02:06.650 --> 00:02:20.800

Pa5: it would be scooting around, maybe getting up to her knees, kind of walking on her knees, but you know that it creates a balance issue. So I mean, I I think most of our physical activity honestly is from scooting around, and then like

21

00:02:20.920 --> 00:02:23.860

Pa5: trying to get into bed or crawling up on the couch.

22

00:02:24.420 --> 00:02:26.290

NM: Okay. awesome

23

00:02:27.080 --> 00:02:30.520

NM: and first prompt is the Department of Health

24

00:02:30.810 --> 00:02:43.980

NM: defines physical activity as any activity that encompasses energy expended. and activation of skeletal muscles. Does this definition change your mind about how you define physical activity for your child? Why or why not?

25

00:02:45.060 --> 00:02:50.310

Pa5: it doesn't change my mind. I think I was looking at it as like. What type of exertion does she have on her own

26

00:02:50.640 --> 00:03:01.450

Pa5: And I think that's why I say, like scooting. I don't think you know, sitting still is in exertion, but that's certainly, you know, scooting between rooms and the house is exertion and getting from one place to another.

27

00:03:01.460 --> 00:03:06.060

Pa5: but always within the confines of an establishment or a home.

28

00:03:07.140 --> 00:03:14.380

NM: Okay, right? And then how do you think physical activity differs from rest for your child specifically.

29

00:03:18.510 --> 00:03:24.890

Pa5: You know what it goes back to exertion that she actually physically is using her muscles, and that if she you know

30

00:03:25.200 --> 00:03:30.480

Pa5: rest at least she's not wearing herself out. But you know, being tired.

31

00:03:30.920 --> 00:03:34.420

Pa5: and you know, having stamina, I think, is how I

32

00:03:34.860 --> 00:03:38.720

Pa5: find, you know differentiate between the 2 of those

33

00:03:41.100 --> 00:04:00.710

NM: great okay. Next question, what activities? What you consider your child does as physical activity. So you told me a little bit about that earlier. But specifically, if there's some specific activities mentioned schooling, is there anything else that you would feel? You feel that she does, whether at school at home

34

00:04:01.920 --> 00:04:13.840

Pa5: only at school or at home, or both.?

NM: It could be both.

Pa5: She stands in a stander, which I know takes a lot of energy to be upright and move around

35

00:04:13.840 --> 00:04:21.959

Pa5: you know, sometimes use a walker, but she'll have her orthotics on, so she's actively, you know, walking at school, but with assistance.

36

00:04:22.060 --> 00:04:28.990

Pa5: and at home. It's like being up under high knees or scooting to go after something.

37

00:04:30.220 --> 00:04:32.420

NM: And well.

38

00:04:32.510 --> 00:04:45.770

NM: your the prompt is, if i'm sure let's discuss some of your child's activities, such as engaging in the use of adaptive equipment. So you did talk about that already with the stander. Would you consider use of a stander and gait Trainer as physical activity?

39

00:04:47.160 --> 00:04:58.740

Pa5: You know it's really hard to someone who is actually mobile and can get around that just the thought of standing, I would say no, but in the capacity of looking at a child with cerebral palsy I would say yes, because.

40

00:04:58.930 --> 00:05:08.990

Pa5: you know, muscles are being activated. That haven't been activated before they're putting weight on the skeletal system, so I do see it. But I see it as a limited capacity.

41

00:05:09.090 --> 00:05:13.880

Pa5: Okay, in in the terms of ‘child’ in the terms of someone who

42

00:05:13.990 --> 00:05:14.620

Pa5: Yeah.

43

00:05:14.880 --> 00:05:17.480

NM: Absolutely. Yeah. It related to her. That's right.

44

00:05:19.640 --> 00:05:25.340

NM: And how about transition transitioning in and out of the wheelchair. Would you consider that physical activity?

45

00:05:27.700 --> 00:05:43.180

Pa5: You know she helps. I mean, she helps, because, as she's getting heavier, it's like ‘child’. Can you put some weight on your feet so that, and can you step into the chair? So it is, but it's a lot of exertion for her to move her whole body without any sort of adaptive equipment.

46

00:05:44.390 --> 00:05:44.960

NM: Okay.

47

00:05:46.180 --> 00:05:49.940

NM: And how about when does she enjoy time on the playground swing?

48

00:05:51.250 --> 00:05:55.900

NM: And Would you consider that physical activity like an outside of the community playground?

49

00:05:57.210 --> 00:06:15.450

Pa5: When ‘child’ was younger, she definitely enjoyed being on the on the swing, said I. Today, I would say she doesn't enjoy it. I'm not really. She's 13. Maybe it's an age thing. Do I consider that physical activity? Not so much. She's kind of it's very passive because she's sitting there. She's not pumping her legs, and she's not holding on.

50

00:06:15.530 --> 00:06:20.340

Pa5: So in Yup related to ‘child’. I don't see it as physical activity.

51

00:06:20.440 --> 00:06:21.070

NM: Okay.

52

00:06:21.330 --> 00:06:25.350

NM: it's great. And then the last one was: how about reaching or Ball toss? She participated in that, though you can?

53

00:06:25.840 --> 00:06:34.800

Pa5: Yeah, yeah, I do. You know what I do now? I wouldn't have before, because I think

54

00:06:35.350 --> 00:06:46.140

Pa5: I do know, because when she's reaching for the ball, and she's throwing it. She's really trying hard, and you can see, like there's a high level of exertion for her to catch the ball, and certainly throw the ball.

55

00:06:47.310 --> 00:06:56.910

NM: Great. Now, how do you, I mean, how does related services such as physical therapy or occupational therapy vision, hearing, education relates to physical activity. In your opinion.

56

00:06:58.640 --> 00:07:00.490

Pa5: In my opinion, I think.

57

00:07:03.200 --> 00:07:17.510

Pa5: How do they relate to… one: I think, without them ‘child’ wouldn't be getting the amount of physical therapy that she needs. I think they're always like engaging her, having her exert, whether she's bending from side to side, reaching for something, drawing

58

00:07:17.610 --> 00:07:29.060

Pa5: all the therapies to me. She wouldn't be where she is, if we didn't have the therapies, and and the only way she is where she is is because she's actually had to work her muscles and tire them out

59

00:07:33.580 --> 00:07:41.450

NM: And do you? Does your child do some of the activities that we mentioned alone, or in a group? And why why not?

60

00:07:42.200 --> 00:07:48.810

NM: So, as we gave examples with the gait trainer to Stander, we talked about playground and reach into ball to

61

00:07:49.500 --> 00:07:51.840

Pa5: sure, so she does

62

00:07:51.990 --> 00:08:02.040

Pa5: both by herself, certain when she does by herself in the gait trainer, but there is but in, and she also does them in a group. I think you know that part of it is, be having it like that social

63

00:08:02.730 --> 00:08:18.510

Pa5: side of it, and being with other friends, and not just doing it by yourself. I don't think anybody wants to go to the gym and constantly run by themselves. So I think sorry. But so yeah. So I think she doesn't by himself, but she also does them in a group, and I think both are very important.

64

00:08:20.060 --> 00:08:28.630

NM: great. And how many times a week this is next question: Does your child participate in these activities, and for how long? If you can put a timeframe on it?

65

00:08:30.390 --> 00:08:36.659

Pa5: So in a week I would say she's doing. Oh, gosh, yes.

66

00:08:36.720 --> 00:08:44.840

Pa5: that's like really hard, so she participates in the ot or in PT and physical therapy. She's, you know, active

67

00:08:45.230 --> 00:08:47.140

Pa5: God every single day.

68

00:08:47.170 --> 00:08:50.430

Pa5: and how many 8 h a day at least 8 h a day.

69

00:08:56.200 --> 00:09:01.880

NM: That's for the full day she's doing physical activity at school, and

70

00:09:02.210 --> 00:09:07.720

Pa5: I I feel like, yeah, we more so at school, you at home. Yeah, because it's a long day at school.

71

00:09:08.660 --> 00:09:15.450

Pa5: But even when I think about our weekend, I feel like she's still getting the 8 h worth of physical activity on Saturdays and Sundays.

72

00:09:15.490 --> 00:09:16.160

NM: Okay.

73

00:09:21.050 --> 00:09:22.070

NM: each day.

74

00:09:22.290 --> 00:09:23.890

Pa5: On Saturday and Sunday its collaborative.

75

00:09:24.110 --> 00:09:33.050

Pa5: I I think, on Saturday and Sunday it's collaborative. But you know, because I'm thinking of her like scooting around and having to stay in balance and reach and stretch. And

76

00:09:35.530 --> 00:09:45.080

NM: And is she doing this with assistance because the scooting she's not so. That would be independent right? And some of the other ones are what the can you help classify for me?

77

00:09:46.440 --> 00:10:01.850

Pa5: Sure, I mean, I would say the scooting, getting it. You know she sometimes requires assistance. So the gait trainer, you know she'll do that on her own. I mean. Somebody clearly has to get her in it the stander somebody gets her into it. She's doing that by herself. You know the scooting with the walking.

78

00:10:01.850 --> 00:10:12.040

Pa5: It's a bit of a mixed bag where she's doing it by herself. But she, I would say 25% of the time she does it by myself. And 75% of the time it's assisted.

79

00:10:17.950 --> 00:10:19.470

NM: Now.

80

00:10:19.950 --> 00:10:24.260

NM: do you think she should participate more or less in these activities? And why?

81

00:10:26.880 --> 00:10:33.340

Pa5: I think more, i'm. So i'm. Comparing one daughter who ‘child’ to a very typical girl, who

82

00:10:33.410 --> 00:10:47.470

Pa5: he has much more activity in her life, and I just think, be more active just you know it. It's not just firing the muscles and making her stronger overall. It's also like firing her brain and making her smarter and more aware of who she is, and

83

00:10:47.470 --> 00:11:01.880

Pa5: and there's consequences to stepping off the curb and not, you know, Looking where you're going, do I think she needs more activity? I definitely think she needs more activity. Yeah, she could to have more activity. There's a lot of sitting and being passive.

84

00:11:02.340 --> 00:11:03.280

NM: Hmm.

85

00:11:04.410 --> 00:11:05.440

NM: That's good.

86

00:11:06.620 --> 00:11:14.000

NM: Okay, those are. Make sure I got through. Those are my main questions for the first half, so the second half i'm going to pull in.

87

00:11:15.190 --> 00:11:24.040

NM: I'm a pull in the promise, so i'll pull it up so you can see it. You Probably you're familiar with this. As so

88

00:11:24.160 --> 00:11:29.380

NM: there are questions. This was actually created by an the National Institute of Health

89

00:11:30.380 --> 00:11:43.850

NM: for children that were not typically developing. So it was a parent proxy. The parent will report on how active or the physical activity intensity that child has performed the week before they did this assessment, and

90

00:11:43.850 --> 00:12:12.270

NM: it's really meant for a child that is, either having a progressive diagnosis, but it wasn't created for children with Cp. Specifically, but it has been validated in some kittens with Cp. So I saw that this, that I'm. Trying to find out what the parents believe about each question. So i'm going to ask you. I'll tell you the question. I'm going to ask you to rate it 0. This question is not applicable to children that are not walking full time. 5 up to 5, totally relatable.

91

00:12:12.270 --> 00:12:19.690

NM: totally appropriate, will give the would be appropriate for a parent to be asked this question, and then i'm asked you to tell me why. Okay?

92

00:12:21.030 --> 00:12:23.170

Pa5: First question.

93

00:12:25.840 --> 00:12:33.370

NM: How many days did your child exercise or play so hard that his or her body got tired. How would you rate this question, and why?

94

00:12:35.400 --> 00:12:45.570

Pa5: I think this is a 5, because I think it's also a self assessment for parents to realize our is the child moving around as a child not moving around. I think it also. You know

95

00:12:45.850 --> 00:12:55.120

Pa5: I find what I answered, and I I see the questions coming forward, that it's reflective; and as a parent it makes me work harder to do more with my child.

96

00:12:55.630 --> 00:13:03.540

Pa5: But I do think that this is good for, like a child like ‘child’, because I feel like she's getting stronger because she's playing until her body is tired.

97

00:13:03.630 --> 00:13:05.010

NM: Hmm. Okay.

98

00:13:07.720 --> 00:13:08.660

NM: right. Great

99

00:13:09.750 --> 00:13:18.020

NM: number 2. How many days did your chat exercise really hard for 10 min or more? How appropriate would you rate this question? 0? Not at all up to 5, and why

100

00:13:21.400 --> 00:13:33.280

Pa5: it was a tough one. I remember first seeing this question, I thought, how is this relatable to my child? Because when I think about exercise. I think about her doing it independently and independently. I don't see her, this being relatable. But

101

00:13:33.390 --> 00:13:48.500

Pa5: now, as she's older and she can scoot around, and I think, did she exercise really hard for 10 min more? No, I don't think this is relatable. I sorry Nia, this is a more like a 2 like, yeah, No, no. And and mainly because of the it because of the term.

102

00:13:48.500 --> 00:13:53.450

NM: Yeah, the term really hard. No, I think it's the really hard.

103

00:13:53.520 --> 00:13:54.590

NM: That's helpful.

104

00:13:56.620 --> 00:14:04.440

Pa5: And you say it's the term, really, because it's hard to quantify for the kids. Yes, yes, okay, incredibly hard to quantify. Got it.

105

00:14:05.970 --> 00:14:23.370

Pa5: And I say this because I think for ‘child’ in her early years, just sitting up was really hard for her. And then you know what I and you look at it. And you think, how can this be like exercise? Really hard like? It's hard for. But I don't think of it as exercise. So the 2 terms together. Don't correlate for me

106

00:14:23.460 --> 00:14:33.320

Pa5: right? It seems a little bit like an oxymoron when i'm thinking about a child with Cp. Exercise, with like the really hard, because what their exercise would be would be really different.

107

00:14:35.590 --> 00:14:36.960

NM: Excellent! Thank you.

108

00:14:37.840 --> 00:14:48.010

NM: And number 3. How many days is your child exercise so much that he or she breeds hard. How appropriate would this question be for you? I think it's a good question, because I think it makes the parent.

109

00:14:48.350 --> 00:14:50.980

Pa5: I think it makes the parent reflective on

110

00:14:51.220 --> 00:14:54.400

Pa5: how much activity their children their child is having.

111

00:14:56.970 --> 00:15:01.410

Pa5: It's hard to answer. I think it's a 4. It's a 4. Okay.

112

00:15:05.120 --> 00:15:07.330

NM: all right Number 4.

113

00:15:07.940 --> 00:15:15.060

Pa5: This was a 2 for me. I'm. I know. I know how many days was your child so physically active that he or she sweated. That's a tough one

114

00:15:15.290 --> 00:15:33.320

Pa5: for me. This is like a one. This is a to be my child Doesn't sweat like she just doesn't sweat, and and on top of that. She doesn't can really control her body temperature. So when it's really hot out she overheats. But doesn't sweat, and when it's really cold out she just gets really cold, and I don't this just wasn't applicable to

115

00:15:33.330 --> 00:15:35.510

Pa5: ‘child’, my child, in this sense.

116

00:15:39.790 --> 00:15:46.500

NM: Number 5. How many days your chat exercises play so hard that his or her muscle burn. How would you rate this one? And why

117

00:15:47.160 --> 00:15:49.010

that, you know that's really

118

00:15:49.180 --> 00:15:50.790

Pa5: that's a tough one, because

119

00:15:50.890 --> 00:16:01.320

Pa5: it's hard to again like quantify that their muscles hurt? Because are they just mentally tired of doing an exercise? Are they physically tired of doing the exercise?

120

00:16:02.570 --> 00:16:07.140

NM: Yeah, it's hard, so the parent would have to to make that determination.

121

00:16:07.360 --> 00:16:09.190

Pa5: Yeah, that's a hard one.

122

00:16:09.470 --> 00:16:12.840

Pa5: It sounds like it's 3 for me.

123

00:16:17.330 --> 00:16:22.190

NM: And you said mainly because of the the the ability to assess.

124

00:16:22.450 --> 00:16:24.980

Pa5: Yeah, I think it's really tough to assess that one.

125

00:16:25.020 --> 00:16:26.380

NM: Yeah, most of

126

00:16:26.510 --> 00:16:39.740

NM: all right. Number 6. How many days your chat, exercise, or play so hard that he or she felt tired. This is a I think this is a relevant one. I think this is I the up 5. This is an easy one, and it it's easy to like.

127

00:16:40.170 --> 00:16:43.270

Pa5: Understand how to answer that question.

128

00:16:47.810 --> 00:16:52.010

NM: And number 7. How many days was your child physically active for 10 min or more.

129

00:16:52.570 --> 00:16:55.960

Pa5: I think that's a 5. I think that's a easy one, too. Yeah.

130

00:16:59.030 --> 00:17:16.420

Pa5: And then the last one number 8. How many days your child run for 10 min or more 0 at you. Yeah, you know it's it's a 2, I you know, because i'm thinking about a child who's not really mobile it just doesn't seem reflective to me whether or not they have the orthotics on I I just

131

00:17:16.880 --> 00:17:20.780

Pa5: I didn't care for this question, because I was a little offended by it. That's

132

00:17:26.300 --> 00:17:27.790

NM: is very helpful.

133

00:17:32.430 --> 00:17:48.340

NM: all right. We are at the end of our talk. I always like to get the final thoughts from my interviewees about this topic related to their children in terms of activity for them any final thoughts or comments or things you would like to say as we wrap up

134

00:17:49.980 --> 00:18:07.150

Pa5: it so in in my own terms, and I may be going, you know, off base here. But I remember like doing this a number of years ago, and I was just so hard to answer these questions that I think I asked one of the caregivers to do it, because I really didn't know how to quantify how to answer these questions.

135

00:18:07.160 --> 00:18:19.360

Pa5: But now is my daughter's older? So, when she was young, it was really hard to answer these questions, but as she's older, I know it's so much better. And so, and I understand the importance of these questions, although I think these questions

136

00:18:19.950 --> 00:18:36.030

Pa5: the relevancy I gave you, but I think for parents. It makes them more reflective in this sense, and I know, I said, that a number of times, but it makes them step back and say, Wow! Am I really doing things with my child or Aren't? I doing it? Because every time after I took these because I was certainly much more involved with my child.

137

00:18:38.630 --> 00:18:50.850

NM: At can I ask the question out? So in those early years you mentioned being offended with question 8. What would be one of one of the most expensive things that you know. Maybe a therapist or

138

00:18:50.860 --> 00:18:59.160

NM: a caregiver? A medical provider may have said, related to physical activity that you hope you can kind of shed light on so it's not repeated.

139

00:18:59.830 --> 00:19:06.040

Pa5: I don't know I haven't. I've never had that happen. I've never had anyone say something to me

140

00:19:06.700 --> 00:19:07.950

NM: that's good to know.

141

00:19:09.480 --> 00:19:26.400

Pa5: They think their answers have been very calculated, and I can read between the lines, but i'm also not going to call them out on it to say, what do you mean by that? Because some things are just better left, You know you don't know, like I didn't think ‘child’ would ever walk or talk and communicate and look at where we are now

142

00:19:29.740 --> 00:19:33.450

NM: that is great, all right. We're done with it. But i'm gonna stop the recording.
