# Supplementary material for: Validity of PROMIS® Pediatric Physical Activity Parent Proxy Short Form Scale as a Physical Activity Measure for Children with Cerebral Palsy Who Are Non-Ambulatory
Source: Behav Sci (Basel). 2025 Jul 31;15(8):1042. doi: 10.3390/bs15081042 (PMC12382615; doi:10.3390/bs15081042)
Supplement: Supplementary file 1 [file behavsci-15-01042-s001.zip › Transcripts copy/Parent transcripts de-identified/Pa4.docx]

WEBVTT

1

00:00:01.350 --> 00:00:15.070

Good morning. Thank you for joining us today. We are going to have a little discussion about physical activity for children with Cp. Who are not full time. Walkers again. Thank you for joining us. We're going to start with some questions and some prompts.

2

00:00:15.070 --> 00:00:29.630

and so answer them to. The best of your Billy is no right or wrong Answer. I thank you for your time. The second half of this this talk is, we're going to look at a survey about physical activity, and it's a parent proxy survey. And I'm: going to ask your opinions about that. So are you ready to begin?

3

00:00:29.900 --> 00:00:30.710

Pa4: Sure.

4

00:00:31.120 --> 00:00:38.660

NM: Okay, great. Okay. So the first question is, how do you define physical activity for your child?

5

00:00:40.550 --> 00:00:42.930

Pa4: getting out of the wheelchair.

6

00:00:45.600 --> 00:00:49.250

Pa4: walking, standing.

7

00:00:50.500 --> 00:00:51.750

Pa4: biking

8

00:00:52.290 --> 00:00:55.110

Pa4: sports, adapted sports

9

00:00:59.430 --> 00:01:15.650

NM: great. So the first prompt I have is the Department of Health defines physical activity as any activity that encompasses energy extended, and activation of skeleton muscle. Does this definition. Change your mind about how you define physical activity for your child. Why or why not?

10

00:01:16.220 --> 00:01:17.630

Pa4: No, that sounds good.

11

00:01:22.930 --> 00:01:25.890

NM: How do you think physical activity differs from Rest?

12

00:01:28.330 --> 00:01:32.540

Pa4: Well, it's different. It helps

13

00:01:34.180 --> 00:01:35.630

Pa4: in what respect?

14

00:01:37.230 --> 00:01:40.670

NM: I guess i'm trying to get at

15

00:01:40.880 --> 00:01:45.060

NM: Can someone be sitting and still engage in physical activity?

16

00:01:45.280 --> 00:01:54.010

Pa4: Yeah, yeah, I mean. I didn't include that in what would be physical activity for my daughter when you just asked that question, but absolutely because they could, you know.

17

00:01:54.030 --> 00:02:07.980

Pa4: raise your arms. They could do upper body. They could probably do lower body, too. It's just with ‘child’ when we, When we think of physics, when when my daughter. When we think of physical activity, we typically get out of the wheelchair.

18

00:02:10.680 --> 00:02:15.550

NM: And can you a little bit more about what that looks like? Does she have assistance to get out of the wheelchair.

19

00:02:15.920 --> 00:02:20.300

Yeah, she needs assistance. She can't She's not able to get out independently.

20

00:02:24.880 --> 00:02:39.420

NM: Okay. Next question. Thank you. What activities. Would you consider, when you kind of answered this, what activities would you consider your child does as physical activity. You can be specific. So you get some examples of walking, but anything in adaptive sports. You have any a list of specific items.

21

00:02:39.800 --> 00:02:58.200

Pa4: Yeah. Well, first of all, the walking is facilitated. She can't walk by herself, and she's not really that she we haven't really been successful in in finding a walker that will help her, even though that would be really, that's like a goal NM I was thinking that would be good like

22

00:02:58.810 --> 00:03:09.390

Pa4: I don't like the rifton But yeah, so getting back to the question. She's done adapted skiing. which

23

00:03:10.660 --> 00:03:16.240

Pa4: isn't really I mean. She sits in an adaptive ski, so it's. I don't know if that's really physical like she's being like.

24

00:03:17.180 --> 00:03:21.640

Pa4: Push down a mountain. She's done a lot of horseback riding

25

00:03:22.380 --> 00:03:33.650

Pa4: that's definitely physical activity that that's core strength. She's done. Let's see what else swimming.

26

00:03:35.220 --> 00:03:48.970

Pa4: She's done walking on a treadmill where a physical therapist facilitates her legs, which is not easy. She's done

27

00:03:49.970 --> 00:03:53.950

Pa4: sailing, which isn't really physical activity. But

28

00:03:54.560 --> 00:04:02.260

Pa4: she's on a boat. She's done what else?

29

00:04:03.840 --> 00:04:05.770

Pa4: Ballet.

30

00:04:07.420 --> 00:04:10.600

Pa4: which is sort of. They stay in the wheelchair for that

31

00:04:10.770 --> 00:04:19.250

Pa4: activity. but for ‘child’ it's mostly facilitated walking and standing

32

00:04:24.530 --> 00:04:27.060

Pa4: and biking, biking also

33

00:04:28.080 --> 00:04:29.710

Pa4: on an adapted bike.

34

00:04:30.260 --> 00:04:31.070

NM: Great.

35

00:04:32.440 --> 00:04:44.170

NM: So you're pretty. I had a prompt, but you answered that pretty nicely. I was gonna say, if unsure. Let's to discuss some of your child's habitual activities, such as engaging in the use of adapting equipment which you shared.

36

00:04:44.360 --> 00:04:48.870

NM: Would you consider the use of a gate trainer, and it's for a stander.

37

00:04:49.180 --> 00:04:56.440

NM: It's physical Activity gait trainer, You already said Yes, To what would you consider the standard you at physical activity? You would.

Pa4: Yes.

38

00:05:00.270 --> 00:05:11.950

NM: does she? Well, she's kind of large now. She's a big. Did you ever use the adaptive sling at the playground?

Pa4: Yes.

NM: okay. Did you like that. Would you consider that physical activity?

39

00:05:15.960 --> 00:05:18.440

NM: And how about Ball toss?

40

00:05:18.730 --> 00:05:19.580

Pa4: Yes.

41

00:05:20.100 --> 00:05:20.660

NM: okay.

42

00:05:24.720 --> 00:05:32.120

NM: And how do related services such as physical therapy, occupational therapy vision, hearing education relates to physical activity

43

00:05:32.780 --> 00:05:34.120

Pa4: very helpful.

NM: Okay, this is how so?

44

00:05:34.290 --> 00:05:45.210

Pa4: Well, there's professionals who know what they're doing, getting your child to engage in physical activity, and and to

45

00:05:45.530 --> 00:05:50.000

Pa4: further there body awareness, strength

46

00:05:53.380 --> 00:05:54.150

NM: great.

47

00:05:55.280 --> 00:05:59.420

NM: And does your child do any of these activities alone, or in a group

48

00:06:00.800 --> 00:06:02.300

Pa4: which activities?

49

00:06:02.370 --> 00:06:13.610

NM: any of the ones you saw you mentioned the sailing, or the I mean. Is she in a group with other children, or it does she's? I mean she's with other facilitated, but like in terms of

50

00:06:13.900 --> 00:06:25.750

NM: these activities, you mentioned some of the adaptive, you know activities. And then why? Why not? What we know? She needs assistance? We talked about that, but in terms of like, maybe participation, she engaged with other children.

51

00:06:25.840 --> 00:06:28.880

NM: Are there other caregivers

52

00:06:32.210 --> 00:06:37.650

Pa4: in the adaptive sports, I would say. Generally, it's a group situation.

53

00:06:38.170 --> 00:06:40.530

Pa4: but it's also going to be one on one.

54

00:06:49.950 --> 00:06:52.280

NM: Do you prefer one or the other?

55

00:06:53.190 --> 00:07:05.320

Pa4: I think both have a place I mean regarding physical activity, I think it's definitely with with not just the adapted sports, but the physical activity. It's very important to have one on one.

56

00:07:05.570 --> 00:07:13.270

Pa4: And then a group is important, too, because you get to, you know. See, Have fun with your peers while you're working out.

57

00:07:24.940 --> 00:07:30.150

NM: And next question, how many times will we get

58

00:07:30.320 --> 00:07:31.310

NM: No problem

[Parent steps away]

59

00:07:38.770 --> 00:07:42.550

Pa4: Oh, okay.

60

00:07:46.560 --> 00:07:47.940

Okay.

61

00:07:48.080 --> 00:07:48.850

Pa4: Okay.

62

00:07:57.210 --> 00:07:57.780

Okay.

63

00:08:01.510 --> 00:08:02.160

Pa4: Okay.

64

00:08:06.770 --> 00:08:07.550

Pa4: Sorry.

65

00:08:11.230 --> 00:08:12.980

Pa4: Because maybe that's easier.

66

00:08:18.530 --> 00:08:19.960

[Parent returns]

Pa4: Hi: Sorry.

67

00:08:22.010 --> 00:08:33.740

NM: No problem. Thank you again. So last question before we get to the survey. How many times a week does your child participate in these activities? And as to specifics of physical activity, and for how long does she participate?

68

00:08:34.940 --> 00:08:38.950

Pa4: Well, I wish it was more. It used to be more when she was in school.

69

00:08:40.270 --> 00:08:43.049

Pa4: But I would say.

70

00:08:43.429 --> 00:08:44.900

Pa4: thinking.

71

00:08:47.460 --> 00:08:52.370

Pa4: I mean what we hope is that each day there's a little physical activity.

72

00:08:53.630 --> 00:09:04.950

Pa4: So she had she. Currently she's doing. She's not really doing horseback riding anymore, since she developed a little bit of a medical issue.

73

00:09:05.360 --> 00:09:11.760

Pa4: but she has ballet once a week, and she has personal training

74

00:09:12.860 --> 00:09:16.960

Pa4: once to twice a week. and

75

00:09:17.210 --> 00:09:25.400

Pa4: she goes on her stander Once to twice a week she goes in her bike once to twice a week.

76

00:09:25.990 --> 00:09:28.780

Pa4: She, what else is there?

77

00:09:30.070 --> 00:09:32.130

Pa4: So it all sort of adds up a little

78

00:09:34.220 --> 00:09:45.160

NM: great Do Does she need assistance to do these equip these activities? And does she need assistance for the entire task or part of the task depending on the activity?

79

00:09:45.880 --> 00:09:49.130

Pa4: I would say, part of the task part of the task.

80

00:09:55.150 --> 00:10:07.640

NM: What does she need Less assistance in. compared to something like, you know, we talk about standing and walking what she needs assistance to facilitate that. What are some tasks which she doesn't need the assistance, the entire time?

81

00:10:07.690 --> 00:10:09.440

Pa4: sure biking.

82

00:10:09.860 --> 00:10:12.940

Pa4: When she set up, she can take over

83

00:10:16.810 --> 00:10:19.780

Pa4: when she's in her stander, she stands.

84

00:10:28.610 --> 00:10:41.100

NM: That's great. Okay, Thank you so much. And do you think she should participate in more or less of these activities. And why so you said more. But why? Because

85

00:10:41.640 --> 00:10:47.230

Pa4: I think it. It's physically healthy for her the number One reason I think she number 2 reasons. She enjoys them.

86

00:10:50.470 --> 00:10:51.250

Pa4: Hmm.

87

00:10:53.810 --> 00:10:59.210

NM: hey? Thank you. All right. So now i'm going to share my screen and show you the survey.

88

00:11:03.380 --> 00:11:06.070

NM: and I will say this survey was.

89

00:11:06.090 --> 00:11:12.350

NM: Can you see it? Yeah, post loading Here we go. So this survey was created for parents to answer

90

00:11:12.620 --> 00:11:19.490

NM: specifically for children that we're not typically developing, not necessarily with to be a pause or a brain injury, but specifically

91

00:11:19.520 --> 00:11:39.410

NM: children that we're not functioning like typically developing children. And specifically, this is a parent would answer about the activity of the child the week before they answered this survey. Okay, and so i'm going to go through each question. You go and take a moment to look at them, and then I'm going to ask you to rate. How applicable

92

00:11:39.510 --> 00:11:52.350

NM: is this question to your daughter or to children that are not fully. They are not walking full time. Okay? And then I'm going to ask you why. So i'll give you a range of 0 not applicable at all

93

00:11:52.390 --> 00:12:06.080

NM: to measure this in this population 5 totally appropriate and valid to measure in this population. Okay, so first question about the last week you asked this you want me to answer regarding last week.

94

00:12:06.320 --> 00:12:15.080

NM: Well, you don't have to answer the questions like I don't want you to answer the questions. I just want you to tell me how applicable valid this question is for parent to be asked

95

00:12:15.100 --> 00:12:20.390

NM: related to this physical activity. Okay, so you don't have to answer the question. But yeah.

96

00:12:20.420 --> 00:12:30.940

NM: it's about how a valid is it? Okay? So the first question is, how many days did your child exercise or play so hard that his or her body got tired. Would you rate this a 0? Meaning?

97

00:12:30.950 --> 00:12:38.070

NM: It's not applicable at all for this population. Or would you say 5 highly appropriate, or somewhere in between. And why?

98

00:12:40.720 --> 00:12:50.580

Pa4: Well, I I just let me explain it first, and then I can, because, like there, there' only been like one or 2 things that would.

99

00:12:50.870 --> 00:12:51.810

Pa4: you know.

100

00:12:52.220 --> 00:12:56.310

Pa4: physical activity, things that would. My daughter would.

101

00:12:56.340 --> 00:13:08.480

Pa4: you know, feel really tired, have our muscles burned three's hard, and that was when Mika used to walk on the treadmill, which he doesn't do any more, and also when ‘child’ horseback road.

102

00:13:09.290 --> 00:13:22.660

Pa4: I think that all of the other exercises physical activity, nothing would fit into that cap into that category. So I guess it's a reasonable question, because

103

00:13:22.770 --> 00:13:27.860

Pa4: even with a child or a young adult who's in a wheelchair. There are

104

00:13:28.100 --> 00:13:31.640

Pa4: at physical activities that would produce

105

00:13:32.250 --> 00:13:36.660

Pa4: these results. It's just that. Sometimes they're not. You know

106

00:13:37.150 --> 00:13:39.190

Pa4: they're not done very often

107

00:13:39.780 --> 00:13:48.660

Pa4: I think the questions reasonable. How many days did your child exercise? I mean that you know they're just difficult to achieve

108

00:13:49.000 --> 00:13:52.470

Pa4: so. But it's it. It could be asked.

109

00:13:53.500 --> 00:13:59.390

Pa4: That's what you want to know how valid. But but to your point and thank you. This is very helpful.

110

00:14:00.500 --> 00:14:07.520

NM: It's not done often, is what you're what i'm gathering from you like to for a child that is not necessarily walking full time.

111

00:14:07.630 --> 00:14:14.730

NM: This is not something that you expect to see on a regular basis, and you gave me 2 examples of when you saw it. So that's how

112

00:14:15.340 --> 00:14:17.450

NM: so? Give me a number. How would you rate it.

113

00:14:18.820 --> 00:14:23.120

Pa4: 0. Not a applicable 5 highly appropriate like.

114

00:14:23.160 --> 00:14:24.800

Pa4: I guess. Live?

115

00:14:24.890 --> 00:14:25.570

NM: Okay.

116

00:14:29.080 --> 00:14:36.020

NM: Okay. Next question. But this is exactly what I need. Thank you. The the explanations are really the meet

117

00:14:36.170 --> 00:14:46.100

NM: bread and butter. Okay, Number 2. How many days did your child exercise really hard for 10 min or more? How would you rate this, and why?

118

00:14:47.940 --> 00:14:54.790

Well, I would rate that as a reasonable question. Because, like it's really asking me, how many days did your child have? Pt.

119

00:14:55.850 --> 00:15:03.470

Pa4: Because when I think about. You know what Nick is doing now in in or first or personal training, that

120

00:15:03.720 --> 00:15:15.060

Pa4: you know. During those days when that happened, I would say exercise was was very hard. So I think it's possible for a child who's.

121

00:15:15.110 --> 00:15:23.150

Pa4: you know, basically in a wheel like you know, the population you're studying to be able to. I I have an ability

122

00:15:23.170 --> 00:15:28.200

Pa4: to meet that criteria. so as I would say.

123

00:15:28.620 --> 00:15:33.360

Pa4: but it's it's difficult, you know. So like, if you're not getting personal training.

124

00:15:34.300 --> 00:15:42.240

Pa4: exercising really hard. It is very hard to exercise really hard. because you're also dependent on someone else.

125

00:15:42.330 --> 00:15:51.410

Pa4: You're not going to like it. You're not like a regular child. It's just going to go a running and play running and running. They're going to get exercise really hard every single day of their life.

126

00:15:51.830 --> 00:16:03.910

Pa4: So you're very dependent on others. and after you leave school you're even it's even less likely because you're you're not quite given the support that you used to be given.

127

00:16:04.440 --> 00:16:10.110

Pa4: So it's a reasonable question, though, especially if your population is still in school.

128

00:16:12.630 --> 00:16:15.440

Pa4: So did I say, how many date? Okay, is it?

129

00:16:15.470 --> 00:16:22.750

Pa4: What you want to know? Is the question reasonable? That's the that's what I'm: yeah like. Is it highly appropriate like? Will it measure physical activity

130

00:16:23.110 --> 00:16:28.600

NM: for this population like. Will that question give you a a good measurement. These kids.

131

00:16:28.660 --> 00:16:32.550

Pa4: in terms of their level of intensity. And you know it's it's

132

00:16:32.610 --> 00:16:40.440

NM: yeah. I mean you can rate it to some, make it be better like 5 is like a really appropriate test. Going to. Really, you get information for this population. And then.

133

00:16:40.500 --> 00:16:45.210

NM: you know, 0 is like this is not applicable at all.

134

00:16:45.350 --> 00:16:49.590

Pa4: Okay, I would say 5. Okay, this one's a 5. Okay, yeah.

135

00:16:50.030 --> 00:16:51.600

All right. Number 3.

136

00:16:51.990 --> 00:16:56.190

NM: How many days is your child exercise so much that he or she breathes hard?

137

00:16:56.630 --> 00:17:03.430

Pa4: Well, the only time they could ever breathe hard was when we were to it when you were doing that assistive walking with me on the treadmill.

138

00:17:04.150 --> 00:17:07.619

Pa4: for, like the 100 steps that she took, and I mean

139

00:17:07.829 --> 00:17:17.410

Pa4: that that she got out of breath for. But how many I how many. So I would say, this is like a 2, because how many kids in wheelchairs is going to achieve that.

140

00:17:20.819 --> 00:17:29.330

Pa4: That's great, okay to and that the the answer is 2 or 3, I mean. And I would say, sweated the same thing

141

00:17:29.400 --> 00:17:30.690

Pa4: 2 or 3.

142

00:17:31.270 --> 00:17:36.890

NM: Yeah, I mean, that's She's flat like that. She sweat like. Do you see that just in general when ‘child’

143

00:17:37.790 --> 00:17:40.130

No, I don't think she's a big sweater

144

00:17:46.430 --> 00:17:54.510

Pa4: muscles burned, I would say the same thing. I don't know you give me a number so, but for number 4, which is the sweating

145

00:17:54.550 --> 00:17:58.780

Pa4: you said you gave me a 2 or 3. I can't remember 2

146

00:17:58.830 --> 00:18:00.800

NM: to, and then it's because

147

00:18:01.150 --> 00:18:03.670

NM: I mean he sweats much.

148

00:18:05.200 --> 00:18:05.850

NM: Yeah.

149

00:18:06.380 --> 00:18:13.920

Pa4: muscle burned, and that's really like more like a Pt. Question, because i'm not sure I would know when her muscles were burning.

150

00:18:14.450 --> 00:18:24.250

Pa4: But I would say that one's probably more because, like, you know, she's working. She's standing for 4 min. Her muscles are going to start burning right? So 5

151

00:18:25.100 --> 00:18:26.830

NM: you give this one a 5. Okay.

152

00:18:27.080 --> 00:18:27.910

Pa4: Yeah.

153

00:18:29.450 --> 00:18:34.740

NM: now more actual because of the activity or more applicable.

154

00:18:37.030 --> 00:18:37.790

Okay.

155

00:18:38.690 --> 00:18:39.460

NM: Okay.

156

00:18:41.890 --> 00:18:45.750

Pa4: And so it's a presumed feeling that her muscles burn.

157

00:18:45.840 --> 00:18:46.550

NM: Okay.

158

00:18:54.560 --> 00:19:03.290

NM: Okay, that was Number 5 and Number 6 was. How many days did your child exercise a place so hard

159

00:19:03.610 --> 00:19:11.170

Pa4: that he or she felt tired

160

00:19:12.120 --> 00:19:15.830

Pa4: on things that you know like wouldn't make somebody else tired. So

161

00:19:15.910 --> 00:19:19.200

NM: some of the best question that's based. That's the best one you like. So far.

162

00:19:20.680 --> 00:19:28.720

Pa4: I like them all. I just think that they're I think they're all good questions. I think they're revealing of reality. you know, which is like.

163

00:19:31.400 --> 00:19:34.840

Pa4: you know, you're not gonna You're less physically active.

164

00:19:35.860 --> 00:19:37.560

NM: Got it. Yeah.

165

00:19:39.820 --> 00:19:49.780

NM: all right. So you gave that one a 5 like a 8 is what I have, and number 7 was how many days was your child physically active for 10 min or more.

166

00:19:49.900 --> 00:19:58.800

Pa4: Yeah, I think that's an excellent question. I'd give that a a 9 or 10, because because you want to know, I mean. That's a good question to ask yourself

167

00:20:01.780 --> 00:20:03.900

Pa4: to keep track of what's happening.

168

00:20:11.300 --> 00:20:13.780

NM: And then the last one, Number 8.

169

00:20:14.490 --> 00:20:23.910

Pa4: It depends on the situation in my situation. I would say 0 because she's never but some kids who are disabled

170

00:20:24.370 --> 00:20:25.710

Pa4: our wheelchair

171

00:20:26.010 --> 00:20:34.810

Pa4: I don't like the word wheelchair bound, but wheelchairs so it's possible they could run. So then I would. But you know, typically in my world.

172

00:20:35.290 --> 00:20:36.640

Pa4: nobody's running

173

00:20:39.380 --> 00:20:52.400

NM: morning. I was my last question we'd like to do before we include is, I always like to ask my interview. We for some final thoughts, for comments, as it relates to physical activity, and this is the opportunity for you to share.

174

00:20:52.420 --> 00:21:04.930

NM: You know anything that you feel that is really important, as it relates to physical activity, and children like your daughter and children that are not walking every day full time the users won't trip a primary mobility, anything you would like to share.

175

00:21:05.560 --> 00:21:11.390

Pa4: Yeah, I think it's really important to do it. I think it's really important to do it to whatever ability

176

00:21:11.470 --> 00:21:28.430

Pa4: a person in a wheelchair can do it, whatever that means, for that person is gonna look differently for depending on the person. Some people can't wait there right. I mean there's all ranges of ability, and you know, just to try to enter to try to get as much support as you can.

177

00:21:28.510 --> 00:21:38.100

Pa4: and the system is very difficult, especially after you graduate, because there seems to be no way to fund physical therapy. Because insurance companies don't

178

00:21:38.390 --> 00:21:43.680

Pa4: consider situation conditions like cerebral palsy.

179

00:21:43.690 --> 00:21:44.790

a

180

00:21:44.900 --> 00:21:48.980

Pa4: a applicable diagnosis for physical therapy

181

00:21:49.400 --> 00:22:06.380

Pa4: unless you're just on medicaid. And then maybe they do, i'm not sure. But but if you have like a commercial insurance that they, you know that they're not going to fund that. So what you got used to your whole life with school is now going to be thrown in the garbage, so

182

00:22:06.380 --> 00:22:18.800

Pa4: you know it's. And then, if the parents are aging and they have to to support the child. It's all like a recipe for not working out in the system, so I would say, the system is very failed.

183

00:22:18.910 --> 00:22:22.950

and you know, I think physical activity is important, so

184

00:22:23.230 --> 00:22:32.300

Pa4: i'm not sure. You know that there's some things as a society we have to be asking ourselves, and I guess we're just saying we don't care. Hmm.

185

00:22:36.730 --> 00:22:39.380

NM: Anything else that's very helpful.

186

00:22:40.800 --> 00:22:45.250

Pa4: No, thank you for looking into this and thank you for all you do

187

00:22:45.410 --> 00:22:50.960

Pa4: thank you for being part of our lives. I'm going to stop the recording. Thank you.
