# Supplementary material for: Validity of PROMIS® Pediatric Physical Activity Parent Proxy Short Form Scale as a Physical Activity Measure for Children with Cerebral Palsy Who Are Non-Ambulatory
Source: Behav Sci (Basel). 2025 Jul 31;15(8):1042. doi: 10.3390/bs15081042 (PMC12382615; doi:10.3390/bs15081042)
Supplement: Supplementary file 1 [file behavsci-15-01042-s001.zip › Transcripts copy/Parent transcripts de-identified/Pa13.docx]

WEBVTT

1

00:00:00.830 --> 00:00:29.050

NM: All right. Thank you so much for joining us today. I am going to be asking you questions about physical activity. It pertains to your child for children that specifically for children with CPU or nonambulatory. So if I sound scripted, it is because I am, and I do have 2 parts to this interview. The first interview will be questions related to kind of your opinion about physical activity as relates to your child, and then the second half is me sharing a survey that was created by the National Institute of Health.

2

00:00:29.130 --> 00:00:38.630

NM: and I want you to grade the questions on how appropriate you think each question is, and why? Okay? So that's pretty much everything we'll go through. So are you ready to begin?

3

00:00:38.710 --> 00:00:39.640

Pa13: Sure?

4

00:00:39.690 --> 00:00:41.790

Okay. So the first question is.

5

00:00:42.780 --> 00:00:45.670

NM: how do you define physical activity for your child?

6

00:00:46.930 --> 00:00:48.630

Pa13: movement.

7

00:00:48.640 --> 00:01:07.500

Pa13: Getting, you know. An ambulation is, you know. would be awesome, but she does that in in a very assisted manner. So a lot of her physical activity that she does independently is rolling back and forth.

8

00:01:07.650 --> 00:01:15.620

Pa13: We're stretching or just movement from a a floor position.

9

00:01:15.770 --> 00:01:19.050

Pa13: It seems as though, like she has less

10

00:01:20.430 --> 00:01:31.130

Pa13: independent movement when she's already seated. That seems to be more difficult. She does do some like bicycling but you know that's you know. That's that's basically it.

11

00:01:31.240 --> 00:01:45.490

NM: Okay, great. And the first prompt to that question is Department of health defines physical activity. Is any activity that encompasses energy extended, and activation of skeletal muscle. How does this definition change your mind about how you define physical activity?

12

00:01:46.490 --> 00:01:49.100

Pa13: I think it's similar. I I don't

13

00:01:52.790 --> 00:01:53.920

NM: Yes, thank you.

14

00:01:56.140 --> 00:02:00.060

NM: Next question. How do you think physical activity differs from rest?

15

00:02:03.060 --> 00:02:10.520

Pa13: Well, I mean physical activity is when you're moving and resting is when you're still.

16

00:02:14.650 --> 00:02:24.040

NM: Well, next question, what activities would you consider your child does as physical activity? So you kind of gave me some examples, so i'll take You mentioned use of

17

00:02:24.440 --> 00:02:29.370

NM: the gait trainer or assistant walking, and some work on the floor. Anything else you want to add?

18

00:02:30.300 --> 00:02:33.780

Pa13: as far as the swim she she does.

19

00:02:33.860 --> 00:02:46.830

Pa13: She's a aquatics through her school, and so she is actually very activated in the water, kicking her legs and moving her arms. But my daughter has a trach, so her head always has to be pretty far above water.

20

00:02:49.240 --> 00:02:50.060

NM: Great

21

00:02:51.900 --> 00:03:01.880

NM: And let's discuss some of your child's habitual activities for engaging in physical activity. So let's talk about it. Some of the adaptive equipment. Does she use a stander?

22

00:03:02.410 --> 00:03:13.460

Pa13: She does use a stander at school. We don't have one at home anymore, just because of like space.

NM: Okay, And would you consider that physical activity?

Pa13: Yes, it's the expenditure she's

23

00:03:14.420 --> 00:03:17.780

Pa13: weight bearing at the time. So she's expending energy?

24

00:03:19.860 --> 00:03:25.800

NM: And you mentioned. Yes, to the gait trainer. How about when she's transferred in and out of her wheelchair? Would you say that's physical activity

25

00:03:26.580 --> 00:03:29.430

Pa13: more for us than for her

26

00:03:31.490 --> 00:03:37.510

NM: the playground swing or using a swing. Does she? Would you consider that physical activity for her?

27

00:03:37.570 --> 00:03:40.790

Pa13: Yeah, I mean that it's it's

28

00:03:40.880 --> 00:03:52.180

Pa13: again, more expenditure of energy of us than her. But she is getting that motion, and so she's participating in. She loves it, she loves being on the swing.

29

00:03:54.100 --> 00:03:59.820

NM: And how about reaching for a ball, or like with the assisted ball toss and reaching. Would you consider that physical activity?

30

00:03:59.880 --> 00:04:00.650

Pa13: Yes.

31

00:04:01.840 --> 00:04:16.220

Pa13: she doesn't do a lot of that independently. But that's where we kind of come in and kind of help hold the ball, and move her arms to grab it and roll the ball down to like knock over things on the floor and stuff like that.

32

00:04:16.660 --> 00:04:17.649

NM: Okay.

33

00:04:17.850 --> 00:04:22.700

Pa13: yeah, yeah, we do, bowling with like an angled pillow.

34

00:04:24.000 --> 00:04:32.610

NM: And how we how does related services, such as physical therapy ot vision hearing, relate to physical physical activity for her? In your opinion.

35

00:04:33.570 --> 00:04:49.420

Pa13: I think they coordinate, like they work together. I think there essential to each other, because if she isn't physically comfortable or able, she can't participate in a number of those other services, so I I think that they

36

00:04:49.560 --> 00:04:50.370

Pa13: or

37

00:04:51.220 --> 00:04:55.410

Pa13: important, and also complementary.

38

00:04:57.400 --> 00:05:14.670

NM: And does your child do some of these activities alone, or she in a group? And i'm talking about like individual therapy, or in a group therapy, and then the activity, like when she's on the floor. For example, when she can move by herself, does she do that independently? And she needs some assistance when she's once she's on the floor?

39

00:05:16.000 --> 00:05:26.880

Pa13: Well, she needs transfer to the floor, and then once she is on the floor, she can move from, you know, laying on her back to her side, and then

40

00:05:27.870 --> 00:05:35.810

Pa13: sometimes back to midline. But most of the time she'll. If you put her in midline. She'll go either way to the side.

41

00:05:36.170 --> 00:05:37.270

NM: Okay, great.

42

00:05:38.500 --> 00:05:46.240

NM: And then she's in a group at school or at home, when she's doing some of these adaptive activities, or she is by herself with the therapist?

43

00:05:46.820 --> 00:05:59.430

Pa13: Oh, it's with a therapist. Yeah, definitely with the therapist and the kids And at school school she is with other kids. And it's nice, because you know, a lot of it is play, and it's interactive, and it's social for her, because.

44

00:05:59.540 --> 00:06:05.230

Pa13: you know. there's not a lot of social opportunities for children.

45

00:06:05.300 --> 00:06:08.070

Pa13: with significant medical needs and disabilities.

46

00:06:09.850 --> 00:06:16.160

NM: Awesome! All right. How many times a week does your child participate in these activities, and for how long can she endure it?

47

00:06:17.370 --> 00:06:42.430

Pa13: I don't think endurance is so much of the issue. It's access to health care. My daughter requires a nurse, and so she's only able to physically go to school 2 days a week, because we only have nursing 2 days a week, and then the other 3 days. We open our home to her aid coming in. And they do video services. Basically, so the aid will

48

00:06:42.550 --> 00:06:53.380

Pa13: help assist her. But then one of us always has to be available in case there's medical needs. So she does participate in things 5 days a week, typically, usually

49

00:06:54.280 --> 00:06:57.390

Pa13: probably for 6 or 7 h a day.

50

00:06:57.960 --> 00:06:58.650

NM: nice

51

00:06:59.870 --> 00:07:04.760

Pa13: because then she also has. like practitioners, come in

52

00:07:05.420 --> 00:07:10.250

Pa13: 2 times a week from an outside program to work with her independently.

53

00:07:12.480 --> 00:07:17.380

NM: That's great. And how long is she standing at school in the Stander?

54

00:07:18.390 --> 00:07:27.110

Pa13: Typically, I think it's like 45 min when they put her in there. But I wouldn't say that they're doing that every single time.

55

00:07:29.220 --> 00:07:33.460

NM: And do you think she should participate in more of these activities, or less

56

00:07:33.810 --> 00:07:50.930

Pa13: more, I would say.

NM: Why?

Pa13: yeah, I mean it's. It's more. I think the more challenging activities happen at school where there is a team of people who are able to help support those activities, whereas when it's just you know

57

00:07:51.050 --> 00:07:55.430

Pa13: her at home. It's a bit more challenging to like

58

00:07:56.590 --> 00:08:02.790

Pa13: be walking with her in the gait trainer, or to get her into the gait trainer, even, you know.

59

00:08:04.800 --> 00:08:06.020

NM: Yeah, I hear that.

60

00:08:07.170 --> 00:08:12.730

NM: And all right. So now we're at the survey. So go ahead. I'll show you the survey. and

61

00:08:12.910 --> 00:08:23.270

NM: I will remind you I did not write this survey. So this is the National Institute of Help. So this was actually created for children that were regressing, as it relates to cancer.

62

00:08:23.290 --> 00:08:39.809

NM: So they were regressing, due to chemotherapy and the just, the the effects of cancer. So they were trying to get create a survey that assess their physical activity. But the children couldn't answer so the the parents would report. And so we're trying to see how this could be related, or it's valid

63

00:08:39.809 --> 00:08:50.140

NM: for this population, children that are not ambulatory with cerebral palsy. So this is the way it's? Answer. The parent would answer, answer, how many days in the prior week. Did they, you know.

64

00:08:50.160 --> 00:09:01.220

NM: do these state questions? But you're not going to answer the question for your child? I'm going to ask you to rank rate the question okay. And this will be related more so, for you know how you feel

65

00:09:01.320 --> 00:09:07.440

NM: as it relates to you know your child and children similar to your child. Okay. So the first question is.

66

00:09:07.770 --> 00:09:19.190

NM: how many days your child exercise will play so hard that his or her body got tired. How would you rate this question 0. Not appropriate at all? 5 highly appropriate and valid for this population? And why?

67

00:09:20.390 --> 00:09:22.500

Pa13: Probably

68

00:09:22.780 --> 00:09:24.790

Pa13: 3, and why?

69

00:09:30.760 --> 00:09:31.760

I guess

70

00:09:33.550 --> 00:09:37.090

I guess it would be hard to tell, because our daughter does it

71

00:09:37.510 --> 00:09:41.980

Pa13: physically express tired, and so

72

00:09:42.200 --> 00:09:45.270

Pa13: she I i'm not sure whether, like

73

00:09:46.730 --> 00:10:02.180

Pa13: like she also doesn't usually fall asleep during things. You know what I mean. So she's not if she is active and activated and engaged, then she's down for it, and so I don't know how I would

74

00:10:04.220 --> 00:10:06.660

Pa13: know the answer.

75

00:10:06.790 --> 00:10:08.890

NM: That's that's great. Thank you.

76

00:10:09.180 --> 00:10:10.520

NM: All right. Number 2.

77

00:10:10.950 --> 00:10:20.540

NM: This is exactly what I want you to do. Perfect? Yeah, how many days did your child exercise really hard for 10 min or more? How would you rate this question, and why

78

00:10:21.370 --> 00:10:29.040

Pa13: this question doesn't really work also for Jonah, because I don't like

79

00:10:29.120 --> 00:10:41.390

Pa13: we're not doing like orange theory here. You know what I mean. Like. Her exercise is more over an extended period of time, and not really hard, because that would

80

00:10:42.530 --> 00:10:44.100

Pa13: potentially

81

00:10:44.160 --> 00:10:45.100

NM: Hmm.

82

00:10:49.000 --> 00:10:55.830

NM: So what number would you write that?

83

00:10:56.160 --> 00:11:04.650

NM: Thank you. Number 3. How many days your child exercise so much that he or she breathe hard. How would you rent this question away?

84

00:11:09.870 --> 00:11:12.940

Pa13: Well, I mean that that's a it.

85

00:11:13.140 --> 00:11:28.710

Pa13: What? What was the ranking again of heroes not appropriate or valid at all, I would say that's a for that is something that could evaluate like for for me like I could make a judgment on that. I don't know

86

00:11:30.610 --> 00:11:32.240

Pa13: how often

87

00:11:32.400 --> 00:11:44.560

Pa13: Jonas exercise would really lead to her breathing hard, because typically for medically fragile children the exercise is more

88

00:11:44.580 --> 00:11:46.860

Pa13: extended and

89

00:11:48.910 --> 00:12:00.220

Pa13: less vigorous than to bring a child to a position where they breathe hard, because, you know, they're medically fragile a lot of times, and so you wouldn't want to bring them to a position where they breathe part, you know

90

00:12:01.560 --> 00:12:05.050

Pa13: so. But but that is a question that

91

00:12:05.330 --> 00:12:07.660

Pa13: I could answer.

92

00:12:08.520 --> 00:12:09.640

Pa13: because then

93

00:12:10.180 --> 00:12:10.800

it's

94

00:12:11.070 --> 00:12:14.520

Pa13: so it's more relevant than the others, I think.

95

00:12:14.830 --> 00:12:15.680

NM: Okay.

96

00:12:17.560 --> 00:12:20.220

NM: perfect. Thank you. All right. Number 4.

97

00:12:20.990 --> 00:12:37.050

NM: How many days was your child so physically active that he or she sweated. How would you rate this question? 0. Not appropriate up to 5 when looking at the intensity for your your child physical activity, intensity, maybe like a 4. Also, because that's another. You know it's a

98

00:12:37.540 --> 00:12:41.820

Pa13: question that can be, answered John. It doesn't typically

99

00:12:43.340 --> 00:12:50.640

Pa13: she's not typically so physically active, but she's flat. But there are times that that has happened.

100

00:12:50.730 --> 00:12:53.200

Pa13: and so that could to be a measure.

101

00:12:53.330 --> 00:12:54.170

NM: Okay.

102

00:12:55.050 --> 00:12:55.700

Pa13: Okay.

103

00:12:56.770 --> 00:13:04.320

NM: all right, Wait a number of that. How many days your child exercise or play so hard that his or her muscles burned.

104

00:13:04.350 --> 00:13:08.310

Pa13: How would you rate that question? I I don't I wouldn't. Yeah.

105

00:13:09.080 --> 00:13:09.970

NM: And why?

106

00:13:10.620 --> 00:13:12.480

Pa13: Well, because I

107

00:13:12.630 --> 00:13:20.870

Pa13: I wouldn't. Jonah would not express whether her muscles burned or not, and I don't believe that any type of

108

00:13:21.030 --> 00:13:30.300

Pa13: activity that she would engage in given her medical conditions would be so vigorous that it would get to a point where the muscles

109

00:13:31.180 --> 00:13:34.750

NM: so you wouldn't want anything that would get to that point right?

110

00:13:40.800 --> 00:13:42.950

NM: And number 6.

111

00:13:43.110 --> 00:13:50.890

NM: How many days did your child exercise? Replace a heart that he or she felt tired? Do you feel? How would you answer. I mean.

112

00:13:50.930 --> 00:14:02.880

Pa13: we're rate this question in terms of again. This is just very similar to the first question about whether her body got tired, so I guess it would be a 3. Also

113

00:14:03.300 --> 00:14:03.930

Pa13: it

114

00:14:06.030 --> 00:14:07.000

Pa13: I think

115

00:14:09.020 --> 00:14:18.030

Pa13: I think you know. I I think that would be an important thing to know about physical activity for your child, I think just in our situation it would be a hard

116

00:14:18.050 --> 00:14:19.610

Pa13: thing to judge

117

00:14:22.920 --> 00:14:26.910

Pa13: You'll notice and express herself as tired. Do you know what I mean, yeah.

118

00:14:26.930 --> 00:14:34.810

Pa13: we we have her, Toby, and I actually tell you she's mad, but she does it usually choose tired.

119

00:14:36.380 --> 00:14:41.420

NM: And you didn't mention her Toby earlier. Would you consider her using her, Toby as physical activity for her?

120

00:14:41.740 --> 00:14:51.990

Pa13: Yeah, kind of maintain that I give. I mean, I always tell that there, because it's funny, because, like a lot of the people are like oh, you know, sometimes she doesn't

121

00:14:52.000 --> 00:15:06.070

Pa13: want to look at the Toby, or whatever like her teacher and I got it, you know. Kind of got into a conversation about this, and I was like. Listen, you do it. I was like you can use it, it'll pick up anybody's eyes. I'm like what you do it, and you know.

122

00:15:06.350 --> 00:15:21.920

Pa13: Sit there and and have to manage what the questions are and what your responses are, and keep your eyes on the screen and move to the page with your eyes. And all this other stuff i'm like it's exhausting. So, of course. Sometimes she doesn't want to

123

00:15:22.120 --> 00:15:30.400

Pa13: do it, or she's not in the moon to look at it, because it is It's a lot. It's a lot of it. It may not be

124

00:15:31.410 --> 00:15:40.320

Pa13: physical energy. Expenditure is not sweating over it, but it's it's a lot of mental energy expenditure.

125

00:15:41.850 --> 00:15:43.310

NM: Yeah, so.

126

00:15:47.250 --> 00:15:51.860

NM: And it is her eyes. You know what i'm saying like that. That is just that's

127

00:15:52.270 --> 00:16:12.020

Pa13: It's a cool yeah, exactly. And like the thing that's always amazing to me. With her with that is, you think about it, compounded with cortical visual impairment, where you know at times the the brain connections are not connecting to her vision. So like. Sometimes I think that

128

00:16:12.020 --> 00:16:26.830

Pa13: maybe like those connections Aren't just happening that day, so it's impossible for her to use it, but she also doesn't have a way of explaining. I'm not seeing. Do you know what I mean? I i'm not seeing correctly. She's, yelling inside right now.

129

00:16:27.050 --> 00:16:40.510

Pa13: She knows me talking about it. Yeah, Exactly. Number 7. How many days was your child physically active for 10 min or more? How would you rate that 1? 0? Not at all appropriate or 5.

130

00:16:40.510 --> 00:16:47.780

NM: I mean she's definitely been physically active for more than 10 min. I mean that that would be a readable question.

131

00:16:50.750 --> 00:16:57.180

NM: And then the last one is how many days your child run for 10 min. How would you rate this one for this population?

132

00:16:57.380 --> 00:16:59.870

Pa13: Run? Yes, 0.

133

00:16:59.900 --> 00:17:02.490

NM: Okay. Not applicable.

134

00:17:02.830 --> 00:17:07.089

Pa13: right? It's not applicable, I mean I i'm I'm not. Maybe other

135

00:17:08.240 --> 00:17:17.730

Pa13: more ambulatory children would would be able to like answer that. But like for her particularly, she's not running for 10 min, so it wouldn't.

136

00:17:19.430 --> 00:17:21.869

Pa13: It's not running. I mean, yeah.

137

00:17:21.880 --> 00:17:31.530

NM: And for the the final thoughts act as we wrap up. I like to ask the parent. You know any comments or final thoughts. Is it release the physical activity

138

00:17:31.700 --> 00:17:37.180

NM: for your children like your daughter? Anything you want to leave for us?

139

00:17:37.280 --> 00:17:38.360

NM: Last off.

140

00:17:38.470 --> 00:17:49.010

Pa13: Well, I think it's great that they're coming up with a scale for physical activity, because I know that a lot of like the standardized tasks Don't really

141

00:17:49.350 --> 00:18:02.160

Pa13: apply to children with several policy, or children who have multiple disabilities or a traumatic brain injury. And so it would be nice to have

142

00:18:03.230 --> 00:18:15.730

Pa13: like a reading scale that is answerable for my child, which is not the current situation. So I mean i'm i'm glad to hear that there's moving towards this Also, I just you know.

143

00:18:16.150 --> 00:18:18.800

Pa13: I hope that in general the the

144

00:18:19.980 --> 00:18:22.210

Pa13: the focus is on helping

145

00:18:24.120 --> 00:18:27.810

Pa13: children who are

146

00:18:28.240 --> 00:18:31.050

not able to independently

147

00:18:31.460 --> 00:18:42.110

Pa13: get involved in physical activity to find more and better ways to help them to do so, because I know currently a lot of the the

148

00:18:42.110 --> 00:18:51.510

Pa13: equipment and the pieces, and the amount of stuff that you have to do in order to get your child to be physically active

149

00:18:51.610 --> 00:18:57.140

Pa13: is all is is prohibitive from the action of it. You know what I mean, Like with all of

150

00:18:57.480 --> 00:19:14.570

Pa13: the technology. Now, you could make a gate trainer that is like aluminum alloy or lightweight, or something like that or foldable. Do you know what I mean? Right now? Everything is huge, and it's heavy, and it's bulky, and it's cumbersome, and

151

00:19:15.390 --> 00:19:26.640

Pa13: as much as you would want to have your child be more physically active. The the equipment that's available to help them to get there is not really functional.

152

00:19:31.220 --> 00:19:35.670

NM: That's excellent. Thank you. That's that's great. Thank you. I must stop for your

153

00:19:36.010 --> 00:19:36.680

Pa13: okay.
