# Supplementary material for: Validity of PROMIS® Pediatric Physical Activity Parent Proxy Short Form Scale as a Physical Activity Measure for Children with Cerebral Palsy Who Are Non-Ambulatory
Source: Behav Sci (Basel). 2025 Jul 31;15(8):1042. doi: 10.3390/bs15081042 (PMC12382615; doi:10.3390/bs15081042)
Supplement: Supplementary file 1 [file behavsci-15-01042-s001.zip › Transcripts copy/Parent transcripts de-identified/Pa8.docx]

WEBVTT

1

00:00:03.430 --> 00:00:24.200

NM: And so thank you so much. Good morning. Thank you for joining us today. We're going to talk about physical activity for children with that are not full time, walkers, and so I have a few questions for you. We're going to start with questions with prompts, and then the second half. We'll talk about an nih develop survey for parents to complete for their child. Are you ready to begin?

2

00:00:24.700 --> 00:00:30.580

NM: Okay, Great. So the first question I have for you is, how do you define physical activity for your child?

3

00:00:32.150 --> 00:00:34.380

Pa8: I think

4

00:00:35.540 --> 00:00:43.190

Pa8: I think it's her doing what she's able to do on her own, and even if it's using devices or or whatever it is.

5

00:00:44.630 --> 00:00:45.360

Pa8: Yeah.

6

00:00:46.540 --> 00:00:52.470

Pa8: yeah, as long as I personally think it's like. If she's not assisted, then that would be physical activity for her.

7

00:00:53.080 --> 00:00:53.680

NM: Okay.

8

00:00:55.900 --> 00:01:13.030

NM: right now, the first prompt is the Department of Health defines physical activity as any activity that encompasses energy extended, and activation of skeletal muscle. Does this definition change your mind about how you define physical activity for your child? Why or why not?

9

00:01:14.280 --> 00:01:15.820

I would think

10

00:01:16.830 --> 00:01:29.700

Pa8: I think, pretty much. She's always doing physical activity, because with Cp she always has that spasticity. So her skeletal muscle is always engaged, whether she is trying to or not.

11

00:01:30.130 --> 00:01:35.600

Pa8: So I guess she's always physically active, but in my mind.

12

00:01:35.960 --> 00:01:39.270

just everyday life, because she's always, I guess, quite a bit active.

13

00:01:40.070 --> 00:01:40.730

NM: Okay.

14

00:01:41.140 --> 00:01:44.040

NM: So it does change a little bit?

Pa8: A little bit

15

00:01:44.240 --> 00:01:50.670

NM: Yeah. And how do you think for her physical activity differs from rest?

16

00:01:54.800 --> 00:01:58.020

Pa8: It's a lot more difficult for her, I think.

17

00:01:59.710 --> 00:02:09.820

Pa8: at rest. I guess she's like one of the lucky ones at rest. She's truly at rest. She doesn't have any of that spasticity when she's when she's sleeping or contractures, or anything like that. So

18

00:02:09.900 --> 00:02:15.130

Pa8: it's. It's exactly opposite. I guess. She says she can be completely at rest.

19

00:02:19.100 --> 00:02:25.160

NM: Next question. What activities would you consider your child does Is physical activity?

20

00:02:25.530 --> 00:02:43.680

Pa8: So she crawls around the house. When she's not in her power chair. She crawls like like an army crawl, using her arms mostly to to pull her. She loves a gait trainer. She loves to be held up under her arms and walking. That's probably the most physically taxing physical activity that she does

21

00:02:43.680 --> 00:02:49.400

Pa8: like with the swash brace on, or even just us, you know, keeping her her legs separate. So she doesn't scissor

22

00:02:50.990 --> 00:03:05.830

Pa8: and then driving her power chair, does take a lot of effort for her, just for for trunk control, because we can see that she she fatigues after a while. That so? That would be, I mean, and she does. She gets exhausted from from driving her chair for too long, so that would be another physical activity for her

23

00:03:06.590 --> 00:03:07.820

NM: sounds good.

24

00:03:07.920 --> 00:03:22.090

NM: and you gave really great examples. But my prompt is, if unsure let's discuss some of the child's activities, and so you already mentioned some adaptive equipment. Would you consider the use of the stander as physical activity?

25

00:03:23.180 --> 00:03:37.020

Pa8: I guess it could be. She has a sit to stand stander, so like she will, she she actually will go halfway between the stand and sit just do a long squat, but you know, and

26

00:03:37.160 --> 00:03:43.760

Pa8: she thinks it's funny just because she can control it. So I guess. Yeah. Use of a stander

27

00:03:45.740 --> 00:03:49.550

when she's. You know she's she does. She goes up and down a lot. She she can

28

00:03:50.970 --> 00:03:52.030

NM: awesome.

29

00:03:54.380 --> 00:03:56.860

NM: Okay. I think I may lost you. Okay.

30

00:03:57.050 --> 00:04:07.510

NM: all right. And so you may have we. You already mentioned the gait trainer. How about transitions in and out of her wheelchair. Would you consider that sort of activity?

31

00:04:09.300 --> 00:04:25.820

Pa8: It depends on her mood, I I think, and also our time like how much time we have to dedicate to it because she is still, since she's tiny, she's still like a grab and go kid for us, so we should probably make her do more in transitions, but we're usually like running like we're on fire. So

32

00:04:25.820 --> 00:04:36.320

Pa8: just we're kind of scooping her up. But it it could be more of a physical activity for her if we have time for it. But she is capable of of helping.

33

00:04:36.540 --> 00:04:37.630

NM: Okay.

34

00:04:37.730 --> 00:04:47.030

NM: And how about the use of a a playground swing like an adaptive swing that you would you consider that physical activity if she's using those and on a playground, or at home?

35

00:04:47.110 --> 00:04:57.770

Pa8: No, she really is when she's in a in a swing, because she can't really sit up on her own. We're almost having to like. Lay her down to swing, and she's happy to rest and just kind of go for the ride. Yeah.

36

00:04:58.260 --> 00:05:02.510

NM: And then how about during a reaching or a ball toss activity?

37

00:05:03.610 --> 00:05:15.950

Pa8: Yeah, she's super like she's very excitable. So if she's she, if we pull out a ball or do anything like that, I know they use it a lot in Pt. Because it's motivating for her. She, her whole body is engaged. So yeah, so I would say for sure.

38

00:05:17.880 --> 00:05:28.980

NM: And then, how do you? How does related services such as Pt oT. Vision, hearing, relate to physical activity or speech for that manner. How does any of the related services

39

00:05:30.660 --> 00:05:34.300

NM: relate to physical activity in your in your opinion?

40

00:05:34.420 --> 00:05:52.950

Pa8: I think they all they all relate. because, especially at her school, where everything crosses over all of the disciplines, like even when she's in speech, like the work. She's not just like, you know, sitting in her chair. She's usually like actively driving around and and trying to like work on conversation skills

41

00:05:52.950 --> 00:06:03.470

Pa8: and engaging people, and like I said, she is excitable. So when she has a task, she's usually all in and like her by default, her whole body gets involved. So I think working on any of that is just

42

00:06:04.690 --> 00:06:06.750

Pa8: is activity for her for sure.

43

00:06:07.050 --> 00:06:13.920

NM: awesome. And does your child do some of these activities alone, or is she typically in a group? And why? Why? Why? Why not?

44

00:06:14.080 --> 00:06:16.110

Pa8: So She has everything alone.

45

00:06:16.120 --> 00:06:19.000

Pa8: and then she will do

46

00:06:19.340 --> 00:06:25.460

Pa8: speech at least one group, setting just obviously to work on like the social aspect of it. And then

47

00:06:26.480 --> 00:06:41.410

Pa8: occasionally they'll do OT like if they're working on like a craft or something like she's again. She's super motivated by other by their her peers, as well as they are by her, so like when they can get them together. But it doesn't take the place of her individual time. It's just usually in addition to

48

00:06:41.760 --> 00:06:42.430

NM: okay.

49

00:06:44.240 --> 00:06:45.150

NM: right

50

00:06:47.960 --> 00:06:57.340

NM: And on the last question before the survey, how many times a week. Does your child participate in these activities, and then…And for how long? So trying to get at endurance

51

00:06:59.020 --> 00:07:14.410

Pa8: So I mean, if if we're just going to go it and and call all of her therapies physical activity. So she is. She is actively engaged from the hours of 9 to 3 at school she's doing she's.

52

00:07:14.520 --> 00:07:20.660

Pa8: I mean, and she's exhausted when she gets home. So she does She doesn't do much or very little, when she comes home.

53

00:07:20.800 --> 00:07:34.140

Pa8: unless, like, if we're going out as a family, or doing something like that, she's up and ready for it. But if given the choice, she's ready to chill out when she comes home, so i'd say consistently from 9 to 3, and then on the weekends she she pretty much goes

54

00:07:34.760 --> 00:07:40.830

Pa8: at least all those hours, plus some. Whatever we do, she's doing it also. So

55

00:07:42.470 --> 00:07:48.940

NM: how long does she tolerate like the walking in the gait trainer like when you said underneath her arms before she really gets fatigued.

56

00:07:48.950 --> 00:08:03.720

Pa8: So underneath her arms she probably we used to be happy before she had hip surgery she was at like 45 min she could go. She was like walking like just just over a mile, was awesome.

57

00:08:03.720 --> 00:08:22.690

Pa8: But after her hip surgery she's probably more around, super fatigued and just exhausted, but the gait trainer, which is more supportive, and she can stop and take rest and do it. She'll stay in there for about 45 min moving around, taking breaks, but but moving as she as she wants.

58

00:08:22.740 --> 00:08:26.230

NM: Okay, Imagine now how long ago was her hip surgery?

59

00:08:26.390 --> 00:08:30.670

Pa8: She had that in 2020. So we're coming up on. Yeah, in 3 years.

60

00:08:30.980 --> 00:08:33.190

NM: She had osteotomies? I'm: just curious.

61

00:08:33.200 --> 00:08:39.900

Pa8: Yeah, She had a stadium on both sides. And then a de-rotation on one side as well. Yeah, there's the left side as well. Yeah.

62

00:08:40.190 --> 00:08:41.280

NM: Muscle Lengthening?

63

00:08:42.419 --> 00:08:47.470

Pa8: They did not do it at that surgery, but she has had something in the past. Yeah.

64

00:08:47.970 --> 00:08:49.700

NM: Okay. all right.

65

00:08:51.020 --> 00:09:02.400

NM: Well, she's doing quite well, since that was only 2. You have a Covid or before Covid.

Pa8: It was. It was during Covid. So it was. We had it scheduled, so it was hard. She stayed in the hospital for about a month.

66

00:09:02.430 --> 00:09:12.740

Pa8: and we stayed with her, and just kind of traded off every day. That was fun, but it was awesome, because, like that, she had that burst of therapy. I could not have made her do what

67

00:09:12.790 --> 00:09:26.090

Pa8: she needed to do, so I was happy to not have to be a therapist in that time. So it was nice,

NM: Gotcha, and the next prom will be. Does she need assistance with some of these activities? So I, of course, by giving in the gate trainer. But once she's in it.

68

00:09:26.250 --> 00:09:36.500

Pa8: she can kind of go. Yeah, she she can go. She has the kidwalk with the big wheels on the side, so she can use this to kind of turn herself if she needs to. But yeah, she does, she?

69

00:09:36.560 --> 00:09:38.980

Pa8: It's pretty independent in the in the gait trainer.

70

00:09:39.450 --> 00:09:51.140

NM: Okay, great. And so how about anything else that she needs assistance in or does not. What? What, what, what does she need? Part assistance, the whole assistance with some of the activities we already talked about.

71

00:09:51.390 --> 00:09:58.840

Pa8: So she is pretty good on that we have. She's. I guess it's called distant supervision for her power chair.

72

00:09:58.850 --> 00:10:03.210

Pa8: so we can. She can kind of go in and and explore on her own.

73

00:10:03.360 --> 00:10:12.400

Pa8: and we'll let her kind of have full reign when we know that there's no like height, differences, or anything like that. That's that's there. and

74

00:10:13.140 --> 00:10:21.080

Pa8: stander. Once we get her in, she's on her own she can control when she's up when she's down when she's you know, going through just doing some squats.

75

00:10:21.170 --> 00:10:22.570

Pa8: and then

76

00:10:22.930 --> 00:10:42.880

Pa8: once she's in any kind of positioning. So like when we're working on any goals or like. And in the classroom. Once outside of getting into her chair, she's she can be on her own to do like manipulatives and things like that. She will ask if she can't get the right grasp on something just to help me, you know. Reposition something. But other than that she's she's on her own

77

00:10:43.870 --> 00:10:45.200

sounds great.

78

00:10:45.320 --> 00:10:48.240

NM: How about the speech. What is she using for communication?

Pa8: She she speaks,

79

00:10:48.310 --> 00:10:55.310

NM: she so she's fully independent with that as well.

80

00:10:55.480 --> 00:11:09.480

Pa8: Yes, it's definitely easier to understand if you're if you are, if you hear her often. But she doesn't get frustrated like you ask her, Say that again. Say that she'll say it 100 times until you get it. Yeah, i'll tell you a great job, a great job. You figure it out.

81

00:11:09.760 --> 00:11:10.720

NM: That's awesome.

82

00:11:10.770 --> 00:11:11.470

Pa8: Yeah.

83

00:11:14.810 --> 00:11:19.130

NM: alright, and do you think she should participate in more and less of these activities, and why?

84

00:11:23.550 --> 00:11:33.950

Pa8: I think I guess we always try to push her to do the most that she possibly can, and and it's it's. We try pretty much everything first, and let it see that it didn't work out, and we have failed

85

00:11:34.470 --> 00:11:44.730

Pa8: amazingly, it and you know, trying to get her do some things, but like we have just, we've never told her. No, if she's ready to go or try it, then we've we've been able to figure out ways to do it, so I don't think that she needs.

86

00:11:44.880 --> 00:11:50.430

Pa8: We're always game for anything, but I don't think she could use more of it because we do as much as she kind of dictates.

87

00:11:52.520 --> 00:12:07.140

NM: That's good. Okay, alright. So let me go ahead and show you the promis scale which you may remember. And so this time i'm not going to ask you to do that to rate, ‘child’. But i'm going to ask you to create these questions. So there's a question.

88

00:12:07.180 --> 00:12:19.760

NM: parent proxy. So the parent does report on behalf of the child. And if you, as you remember, it was prior to the week, your your grading. And so what I'm. Going to ask you in terms of how this is

89

00:12:19.780 --> 00:12:23.010

NM: relative to a child that is not walking full time.

90

00:12:23.480 --> 00:12:40.710

NM: right? And so for each question I'm asked you to rank it 0 totally, not appleable. Does not a good question to ask a parent, and why, and then up to Number 5, which is like totally relevant. A good, a good people give you a good gauge on physical activity, intensity for this population

91

00:12:40.840 --> 00:12:48.840

NM: all right. So the first question is, how many days you chop, exercise, or place so hard that his or her body got tired? How would you rate this question, and why?

92

00:12:49.490 --> 00:12:54.880

Pa8: I think it's a terrible question, I think, because our kids bodies are always tired. I think that they they don't have.

93

00:12:55.040 --> 00:13:08.800

Pa8: whereas I can't think that's how we kind of talked about, whereas a typical kid can stop and take a rest like ‘child’ can be at rest. A lot of kids can't be at rest. They still have that tone and to a degree, unless she's actually asleep.

94

00:13:08.890 --> 00:13:13.640

Pa8: There's still tone present, so she's never really getting like true rest, I think, unless she's sleeping

95

00:13:13.820 --> 00:13:19.450

NM: alright. Well, how would you rate it? 0. Not at all up to 5. What number would you get it.

96

00:13:20.040 --> 00:13:21.120

Pa8: maybe a one.

97

00:13:21.800 --> 00:13:22.440

NM: Okay.

98

00:13:22.840 --> 00:13:32.980

NM: All right, Number 2. Thank you. Very helpful. How many days did your child exercise really hard for 10 min or more? How would you rate this in terms of giving it to a parent

99

00:13:33.000 --> 00:13:35.500

NM: of a child with Cp. Who is not a full time? Walker.

100

00:13:36.860 --> 00:13:41.190

Pa8: I also think this is like not applicable, because I feel like

101

00:13:41.850 --> 00:13:43.500

Pa8: I mean what's really hard.

102

00:13:43.620 --> 00:13:46.170

NM: It's it's so hard to figure like.

103

00:13:46.780 --> 00:13:51.260

Pa8: really hard. I I did the hardest thing for Mayor Grace to do would be to you know

104

00:13:52.070 --> 00:13:57.220

Pa8: us to be standing up and and holding her walking. That's probably her hardest task

105

00:13:58.860 --> 00:14:10.000

Pa8: on a day that we do that, but like otherwise, I mean, like every like this, I don't know. I just think it's. I think it's not applicable, because we're not really ever saying, okay, Go and do this for 10 min. It's. How can we. you know, fit in this

106

00:14:10.060 --> 00:14:11.500

Pa8: activity for you?

107

00:14:11.950 --> 00:14:13.030

NM: Yeah.

108

00:14:14.670 --> 00:14:17.590

NM: Great. Thank you. Did I get a number? I'm: sorry

109

00:14:17.760 --> 00:14:19.840

Pa8: I was 0.

110

00:14:20.040 --> 00:14:20.820

NM: Okay.

111

00:14:21.060 --> 00:14:25.400

NM: So yes, we don't use that. You don't you just like this even more than the first one

112

00:14:28.010 --> 00:14:36.090

NM: all right next one. Thank you. But 3. How many days your child exercise so much that he or she breathes hard.

113

00:14:38.490 --> 00:14:42.720

NM: Yeah, it's it's it's just silly.

114

00:14:42.910 --> 00:14:44.080

Pa8: Because.

115

00:14:45.720 --> 00:14:51.680

Pa8: yeah, I don't know. I I would say probably a 0 for this as well. I just I mean.

116

00:14:52.600 --> 00:15:07.410

Pa8: I don't think we've ever gotten. I mean there's there's times where she's been like breathing hard, but like it, it usually results in like body failure before I would say, oh, she's breathing hard. It would be like just a collapsing of like I'm just i'm done. I can't do it. I would never be like oh, she was breathing so hard.

117

00:15:07.850 --> 00:15:14.120

Pa8: but she's just. She's physically exhausted. So I would say 0 probably like not not not applicable.

118

00:15:14.160 --> 00:15:15.130

NM: Yeah.

119

00:15:16.060 --> 00:15:27.130

NM: because it's not necessarily related to exercise. All right. Number 4. How many days was your child due to a child so physical that he or she sweated.

120

00:15:29.080 --> 00:15:39.710

Pa8: I mean, that's fine. I would, I would say, because I could answer that question with any kind of like. If we had her up in the gate trainer I could say yeah, she maybe she's, maybe, she sweated, but I again

121

00:15:41.430 --> 00:15:47.800

Pa8: I don't like any. I don't like any of the questions, but I would say probably probably a one or 2. I mean it's like. If that was a

122

00:15:47.920 --> 00:15:53.170

Pa8: a strong marker, like an ease, it's an easier thing to mark, I think. Then other things.

123

00:15:53.290 --> 00:15:55.240

NM: Okay, this is one easier to grade.

124

00:15:55.280 --> 00:15:56.050

Pa8: Yeah.

125

00:15:56.600 --> 00:15:57.340

NM: okay.

126

00:15:58.200 --> 00:16:01.410

Pa8: And what number did you give it again? I'm: sorry that you can give it to

127

00:16:03.330 --> 00:16:11.670

NM: Number 5. How many days your child exercise or play so hard that his or her muscles burned. How would you rate this one?

128

00:16:12.370 --> 00:16:13.310

So

129

00:16:13.980 --> 00:16:19.220

Pa8: I think, if your kid can accurately report it, ‘child’ is very aware of her body, and she'll

130

00:16:19.240 --> 00:16:30.580

Pa8: she'll tell us sometimes, like you know. Her muscles are tired. She's never used the word burned. I don't know if we kind of just go by her. She'll be shaking. She'll just be.

131

00:16:30.980 --> 00:16:45.320

Pa8: and I would. I would imagine, at that moment her muscles are burning, but I don't know if it's an accurate question. If if kids have the language to express that so. and that that's kind of something that they have to tell you rather than You're judging that their muscles are burning.

132

00:16:49.390 --> 00:16:53.190

NM: That's all good points. What number would you give this one?

133

00:16:53.740 --> 00:16:55.200

Pa8: Probably one.

134

00:16:57.160 --> 00:16:59.600

NM: and then number 6?

135

00:16:59.900 --> 00:17:04.099

NM: How many days. You're trying to exercise a place for heart that he or she felt tired.

136

00:17:04.390 --> 00:17:10.920

Pa8: A 0. I feel like our kids like life is hard. I don't think we're we're saying that they're going out and playing hard

137

00:17:11.440 --> 00:17:12.329

NM: right?

138

00:17:16.130 --> 00:17:21.839

NM: And then number 7. How many days was your child physically active for 10 min a month.

139

00:17:23.450 --> 00:17:30.720

Pa8: I guess that goes back to your definition of what we're saying is physically active, because then you would say, you know, every day it's 10 min. But what are you saying? Are you?

140

00:17:30.970 --> 00:17:38.530

Pa8: Are we saying that they're breathing hard? Are we saying that their muscles are burning like then I have no idea, I can't tell you. But if we're saying that she's just active because she's

141

00:17:38.880 --> 00:17:43.500

Pa8: trying to participate in her life, then it's just a hard question like without more

142

00:17:44.030 --> 00:17:45.430

Pa8: direction to it.

143

00:17:45.540 --> 00:17:51.020

Pa8: like I wouldn't have a problem answering that for my typical kids. you know.

144

00:17:51.890 --> 00:17:57.540

Pa8: because they're always out running around. But like again, what are we defining as physically active for? For ‘child’?

145

00:17:58.350 --> 00:18:03.380

NM: Okay. So this one. So what? What? What language would you feel

146

00:18:03.460 --> 00:18:11.270

NM: with this question will be, you know, in terms of the definition to be added to that question to be more receptive from a parents perspective.

147

00:18:13.570 --> 00:18:28.580

Pa8: I almost it's. It's maybe more so. The the order of it we, if I had. This was a a question that I had first. I may have thought more about the definition that we had talked about about being physically active, but because we just talked about playing so hard that you were sweating or you were breathing hard.

148

00:18:28.580 --> 00:18:34.240

Pa8: Then it almost throws me off and going. What are we actually talking about anymore? Because I just kind of told you that they weren't doing all those things.

149

00:18:34.620 --> 00:18:38.980

Pa8: But now I can tell you. Yeah, for 6 days in a row that she was physically.

150

00:18:38.990 --> 00:18:42.420

Pa8: You know what I mean. Like it's almost like the order of it. It would have

151

00:18:42.810 --> 00:18:49.010

Pa8: kind of played differently in my mind if I had read it first, rather than after all, of the exercise, hard exercise questions.

152

00:18:50.770 --> 00:18:52.160

NM: some really good one.

153

00:18:54.170 --> 00:18:56.160

NM: Okay, give me a number for this one.

154

00:19:00.460 --> 00:19:03.680

Pa8: I guess you were one or 2 to go to, but 2 is fine.

155

00:19:05.040 --> 00:19:07.280

NM: Okay? And then the last one.

156

00:19:07.690 --> 00:19:16.030

Pa8: Number 8. How many days your child run for 10 min and more? How appropriate Would you rate this question?

157

00:19:20.380 --> 00:19:25.200

NM: Okay, i'm not going to ask you why I think I got it on.

158

00:19:25.330 --> 00:19:37.810

NM: So, as we near the end, I like to get like final comments and thoughts from the parents. Anything you want to share, like some last remarks about physical activity in this in this population.

159

00:19:39.200 --> 00:19:53.130

Pa8: Well question just a a a comment on the actual surveys. I think, when we get presented surveys so often they don't apply to our kids at all that we kinda. You gotta get like survey fatigue, and then, like, I feel like if you could get a survey

160

00:19:53.830 --> 00:20:11.820

Pa8: that ask questions that were so specific to your population, then you're more apt to like kind of think through things, but like when I was doing this it was like, I don't know, like you know, what do you say? Because, like that kind of stuff like, and I don't think that any information that's put down on these surveys are accurate at all, because you're at best. Guess you're trying to guess what the researcher wants.

161

00:20:11.820 --> 00:20:14.640

and then say, like it doesn't really apply. But I guess if you do, it.

162

00:20:15.070 --> 00:20:19.950

Pa8: does it apply to her in her own ability Level versus a typical kid.

163

00:20:20.160 --> 00:20:31.210

Pa8: because I don't think these these apply. If you're looking at typical kid, you know. But then you're trying to say, like, okay, Well, how much like, what is my definition versus yours? It's just like these surveys in general are just like

164

00:20:31.310 --> 00:20:34.890

Pa8: just so silly like it, and it like you read it, and you're going.

165

00:20:34.980 --> 00:20:45.900

Pa8: I think we've passed the point of going like oh, great! Another thing is pointing out what our kid can't do. I think we're past that point, but I know it's very real. When your child's younger they're just

166

00:20:46.190 --> 00:20:57.070

Pa8: they're hard to read all the things that your kid can't do, and and like kind of bring to light all those things. But yeah, just the need for a survey that more accurately reflects

167

00:20:57.640 --> 00:21:01.810

Pa8: what our kids abilities are. And then you don't have to. You know. Kind of you

168

00:21:03.820 --> 00:21:04.790

Pa8: I don't know.

169

00:21:04.900 --> 00:21:07.680

Pa8: Try to connect things that really aren't

170

00:21:08.840 --> 00:21:10.510

Pa8: applicable, I guess.

171

00:21:13.370 --> 00:21:17.160

NM: Yeah. Anything else you want to add.

172

00:21:21.850 --> 00:21:23.580

Pa8: I don't think so. I think

173

00:21:24.110 --> 00:21:32.600

Pa8: I think I think it's I'm. I'm so interested in in the research that you're doing just because I I feel like there's just such a range of kids that have Cp of like what

174

00:21:32.620 --> 00:21:42.040

Pa8: like I don't think ‘child’ is even like the typical, You know. power-chair. User She has so many more like kind of crossing over between, like Gmf. Cs levels. So

175

00:21:42.080 --> 00:21:42.800

I

176

00:21:42.840 --> 00:21:54.290

Pa8: it's it's really interesting. I'm excited. I'm thankful that you're doing this. I'm excited to to see what your research is. and I think it just was a really good perspective. I mean just this conversation, just saying that, like you know.

177

00:21:54.360 --> 00:22:10.780

Pa8: they say what it is like 3 to 5 times more energy than a typical typical kid to get through their day. And you're saying like, if you, if you're doing this at every second, you know for every activity that you want to do, you are technically working really hard. It is just a really interesting perspective to to put things in. So

178

00:22:10.970 --> 00:22:11.710

Pa8: yeah.

179

00:22:11.750 --> 00:22:12.620

NM: no.

180

00:22:12.700 --> 00:22:17.210

Pa8: Oh, well, that's great. I really appreciate that. Okay, I want to stop the recording.
