# Supplementary material for: Validity of PROMIS® Pediatric Physical Activity Parent Proxy Short Form Scale as a Physical Activity Measure for Children with Cerebral Palsy Who Are Non-Ambulatory
Source: Behav Sci (Basel). 2025 Jul 31;15(8):1042. doi: 10.3390/bs15081042 (PMC12382615; doi:10.3390/bs15081042)
Supplement: Supplementary file 1 [file behavsci-15-01042-s001.zip › Transcripts copy/Parent transcripts de-identified/Pa14.docx]

WEBVTT

1

00:00:01.320 --> 00:00:20.330

NM: All right. Good evening. Thank you so much for joining us to discuss physical activity for children with the quality we're not full time walkers, so I didn't get your consent yet. So i'm going to go and ask you if you would allow me to interview you about your child, as it relates to physical activity, definitions, and measurements.

2

00:00:20.330 --> 00:00:24.550

Pa14: Is that okay to record you for this interview?

3

00:00:24.590 --> 00:00:38.170

NM: Thank you so much, and we're going to continue with the interview. And so there's gonna be a few questions with some proms about how you define fiscal activity as a release your child. And then the second half is going to be about survey that was created by the National Institute of Health

4

00:00:38.260 --> 00:00:51.610

NM: for parents to report about the physical activity of their child. And we're going to talk about how applicable you feel that would be to children like your daughter. So the first question I have for you is, how do you define physical activity for your child?

5

00:00:53.420 --> 00:01:00.040

Pa14: Physical activity, I guess, would be anything that's requiring her to

6

00:01:00.550 --> 00:01:04.450

Pa14: use her muscles, her body

7

00:01:04.550 --> 00:01:11.120

Pa14: to, I guess, engage in whatever her goal or activity or need is at the moment.

8

00:01:11.150 --> 00:01:20.570

Pa14: Sometimes it could be just. Stretching is a physical activity. Her trying to sit up, her trying to, you know, Maintain her balance of her head.

9

00:01:20.600 --> 00:01:27.200

Pa14: using her, Toby. Just you know anything that requires to me, mental focus that

10

00:01:27.390 --> 00:01:30.590

Pa14: and relates to her physical activity

11

00:01:32.330 --> 00:01:44.370

NM: great. Thank you. So the Department of Health defines physical activity as any activity that encompasses energy expended and activation of skeletal muscles. Does this definition change your mind about how you define physical activity.

12

00:01:49.080 --> 00:02:06.240

Pa14: I mean, in a way, yeah, I mean, I mean, I I I don't know if i'd say change it. I just don't want 100% agree with it. I don't. I don't think it has to always do with the moving of skeletal muscles, although that is the definition of physical. So I don't know. I think there are some times at the the idea of

13

00:02:06.630 --> 00:02:17.130

Pa14: things that my daughter has to do. Aren't necessarily physically obvious. I guess the best way to put it, but I still consider it an activity for her to do

14

00:02:17.150 --> 00:02:22.590

Pa14: so. But yeah, I mean I that I mean. That sounds like a pretty accurate definition, I guess.

15

00:02:23.760 --> 00:02:28.640

NM: Thank you. And how do you think physical activity differs from rest?

16

00:02:31.010 --> 00:02:35.270

Pa14: from rest? I guess I would say is when you're not.

17

00:02:35.630 --> 00:02:42.840

Pa14: you know at all exerting energy or just relaxing. And you, you know whether you're sleeping or laying down, or

18

00:02:42.880 --> 00:02:47.010

Pa14: you know anything where you're not using your

19

00:02:47.110 --> 00:02:51.960

Pa14: conscious ability to do something like keeping your head upright.

20

00:02:52.040 --> 00:02:52.940

NM: Hmm.

21

00:02:56.890 --> 00:03:01.640

NM: And i'm just gonna throw an extra question in there. Does your child.

22

00:03:03.580 --> 00:03:14.530

NM: when she wants to maybe rest is able, to always rest. Do you know what I mean? Like i'm talking about like spasticity? I don't know tone. Does she have moments of that?

23

00:03:14.540 --> 00:03:30.840

Pa14: I mean, I don't know if I would say she's always able to rest. I know she is able to relax her body at times, which you know those times of somewhat concerning me, because she'll flop and like, lay her head down, and her head will be almost in her lap, which looks incredibly uncomfortable to me.

24

00:03:30.840 --> 00:03:40.140

Pa14: But maybe for her that's what she needs to. You know she needs a a minute, an hour, whatever it is to relax. And so that's how she relaxes.

25

00:03:40.240 --> 00:03:42.500

Pa14: So

26

00:03:42.620 --> 00:03:50.690

Pa14: I I think she's able to relax at times. I don't know if I would. You know. Say that she can relax whenever she wants.

27

00:03:51.070 --> 00:03:52.380

Pa14: you know, consistently.

28

00:03:53.000 --> 00:03:53.720

NM: Okay.

29

00:03:54.360 --> 00:04:03.050

Pa14: you know, like you said as spasticity in her tone. I i'm sure things that she's, you know, battling against constantly.

30

00:04:03.160 --> 00:04:08.090

Pa14: and you know I I would imagine she's just fine. She finds ways to adjust

31

00:04:08.240 --> 00:04:11.390

Pa14: and deal with it. You know

32

00:04:11.730 --> 00:04:22.860

NM: That's good. Thank you. What activities would you consider your child does as physical activity? You mentioned some earlier? You mentioned the Toby, and holding her head up right. Anything else you want to mention?

33

00:04:22.900 --> 00:04:35.530

Pa14: Yeah, I mean, she uses a stander. She uses a gait trainer. She uses an adaptive tricycle. She at times is in the pool, the swimming pool.

34

00:04:35.550 --> 00:04:40.900

Pa14: She kicks her legs quite a bit. Anytime she's near water. If it's the pool or at the beach.

35

00:04:40.920 --> 00:04:50.410

Pa14: There are times. If we're around animals or pets, you'll notice that she's trying to do more with our arms and hands like reaching out to them, and so on.

36

00:04:53.590 --> 00:05:07.480

Pa14: You know if you come anywhere near her face. She you know she does what she can to protect her eyes. And so I mean I don't know if I can say that a physical activity. But it is a reaction.

37

00:05:07.570 --> 00:05:12.940

Pa14: But yeah, I mean, i'm trying to think of anything else that she does in terms of physical activities.

38

00:05:15.930 --> 00:05:20.140

Pa14: Yeah, I think I mean, yeah, but I think that's about it.

39

00:05:20.450 --> 00:05:31.690

NM: Okay, that's great. I do have some prompts about some habitual activities, such as engaging in the use of adaptive equipment. So you mentioned biking. How does she use the adaptive tricycle?

40

00:05:32.200 --> 00:05:48.820

Pa14: with the adaptive tricycle as long as she's secured into the seat, and you know she has her feet, you know, strapped into the where the pedals are. She's able to get momentum going. She's able to maintain momentum. You have to steer at the bicycle, the tricycly, I should say so that she doesn't crash into anything

41

00:05:48.820 --> 00:06:06.500

Pa14: you can see. She enjoys it, and she she's able to pedal to the point where the idea of being put on a bicycle kind of triggered a sort of cyclic cycle motion with her legs in general, so she does that quite often, which looks like She's just kind of training, for.

42

00:06:06.500 --> 00:06:13.180

NM: you know, getting ready for the next time she's on her tricycle, but that seems like it's great exercise and something she enjoys doing

43

00:06:13.290 --> 00:06:19.570

NM: that's wonderful, and then is she able to help push to propel the bike forward with the sisters of somebody pushing her.

44

00:06:20.130 --> 00:06:26.890

Pa14: Yeah, I mean you. You could propel her. But if you're on a nice smooth surface she can get it going on her own.

45

00:06:27.070 --> 00:06:44.720

Pa14: And so there are times when we're out, whether you know, if it's like a park or the boardwalk, any kind of smooth surface. She can get it going on her own if she's on a more bumpy surface or something that's a little bit more rough. Then. Yeah, we might have to give her a little bit of a push, you know, because of the traction.

46

00:06:44.720 --> 00:06:48.230

Pa14: But then she's able to maintain that momentum until she stops peddling.

47

00:06:48.760 --> 00:06:55.180

NM: That's wonderful. So that's some independent mobility right there. That's really great.

48

00:06:55.230 --> 00:06:55.940

Pa14: Yeah.

49

00:06:56.180 --> 00:07:04.260

NM: that makes sense. And you mentioned the stander you mentioned the gate trainer. How about transitions in and out of the wheelchair? Would you say that that physical activity? or is it more passive for her?

50

00:07:04.290 --> 00:07:18.320

Pa14: Umm she stretches when you get her out of the wheelchair. For her is more of a passive activity,

51

00:07:19.300 --> 00:07:23.430

Pa14: kind of just being moved from one place to the other.

52

00:07:23.480 --> 00:07:42.680

Pa14: There are times, though. She will stretch in the middle of you, you know, transferring her, you know. I guess, just because she feels like she needs a stretch. There are times she'll do things in the middle of like diaper changing. We'll show arch her back you know. Lift her butt up, and I think, and it's her way of trying to either help or be a pain in the butt, one or the other.

53

00:07:42.680 --> 00:07:49.040

Pa14: But she does it consistently enough that I don't think it's just, you know, a flute.

54

00:07:51.520 --> 00:07:52.560

NM: Yeah, that's great.

55

00:07:53.640 --> 00:08:06.750

NM: And how about the playground swing? The adaptive swings at the playground?

Pa14: Oh, yeah, yeah, yeah. They adaptive swings in the playground. She enjoys, You know. We we get her in there. And again it's a matter of getting her secured in the seat.

56

00:08:06.810 --> 00:08:16.820

Pa14: But at at that point you know as much as it's a sort of quote unquote passive thing that she's just sitting there. She is obviously to me. I think the sort of

57

00:08:18.520 --> 00:08:25.810

Pa14: one of the things I've come to sort of recognize is the amount of energy and time and focus it must take for her to keep her head upright.

58

00:08:25.830 --> 00:08:41.830

Pa14: And so, being in a swing where you know she's going back and forth to sometimes a little bit, you know, swaying to the sides. She keeps her head up, and that's not by accident, I mean. I think she's focusing on doing that, and she's enjoying the swing. And so

59

00:08:41.830 --> 00:08:47.350

Pa14: I think there's some physical activity for her in that opportunity of being in this way.

60

00:08:49.320 --> 00:08:57.940

NM: And how about yeah, that's great. How about her use of the arms during reaching of a ball toss? Would you consider, even with the assistant, that physical activity for her?

61

00:08:58.580 --> 00:09:17.350

Pa14: Yeah, I mean it, you know. Again it really it's more obvious when it's she's around animals. She's around animals you can see she's in so to reach out. You know you can see she starts. She tries to like, just lay her hand on it, and like kinda I don't know if I would say wiggle her fingers, but she keeps her hands more open

62

00:09:17.380 --> 00:09:31.630

Pa14: when she's around some kind of like animal like, especially like, you know, something like with thick hair like a a sheep, or something like that, cats, or anything soft, she really you could tell she's she's reaching out to it, and she's trying to actually engage it with our end.

63

00:09:31.710 --> 00:09:50.950

Pa14: and there's not a lot of things that she really reaches out for to try to like, you know. Grab it, I mean she uses other adaptive devices like the wobble stick and things like that. But those are things where you really kind of have to like, situate her hand into it. So on. Now there are things that she does

64

00:09:50.950 --> 00:09:54.930

Pa14: use the limbs more actively and more independently, for, like if you put like

65

00:09:55.140 --> 00:10:11.560

Pa14: bells on her wrist and stuff like that, you know, because she loves music. You will she, She will start making music with her, you know. Whatever musical devices are, you know, within her her reach, or tied to our wrist or attached to this, I would say

66

00:10:11.590 --> 00:10:19.660

Pa14: so. Things like that, things that she seems to enjoy. Yes, you can see she makes a physical effort to do something to participate in that.

67

00:10:20.190 --> 00:10:21.420

NM: That's awesome.

68

00:10:22.600 --> 00:10:30.170

NM: all right. And how does related services such as PT, OT. Vision, or hearing even speech. How does that relate to her physical activity?

69

00:10:30.970 --> 00:10:48.670

Pa14: I think it's all played a part, I think, if she wasn't getting these related services, she wouldn't be able to do any of these things. I mean, I think, that that you know from the time she was, you know, first home, after having been diagnosed with cerebral palsy, she had the traumatic brain injury.

70

00:10:48.670 --> 00:11:08.000

Pa14: She was, you know, incredibly tight, you know, high tone. Her arms were stiff all day, elbows locked, fist clinched, and if it wasn't for the fact that she was getting services, especially ot and pt to directly loosen up those muscles and

71

00:11:08.000 --> 00:11:09.460

Pa14: get her to kind of

72

00:11:09.480 --> 00:11:28.760

Pa14: have a more functional, you know opportunity to use her arms and hands. She wouldn't be able to do these things, and so in the same with like her vision services and her speech services, I mean, I think her speech services have done a lot to kind of give her the opportunity to at least smile more, for instance.

73

00:11:28.760 --> 00:11:48.210

Pa14: and have more facial affect, and you know expression, and as far as a vision you know, the challenge of you know, of of having her. You be exposed to. The Toby has given her an opportunity to express herself, you know, and communicate. You know ways that she can't do verbally

74

00:11:48.210 --> 00:12:03.150

Pa14: so, and you know I think it's given our opportunity to be more, you know. You know, educated and things that are out there music, books, colors, you know, shapes, numbers, things that you know

75

00:12:03.590 --> 00:12:09.760

Pa14: that don't work so well with like, you know, paper and books and cards. I mean it's it's

76

00:12:09.810 --> 00:12:12.730

Pa14: It's really seem to give her an opportunity to be

77

00:12:12.760 --> 00:12:19.020

Pa14: more in control and more independent, and actually more engaging. Something she's, you know, seems to have a lot of fun with

78

00:12:19.250 --> 00:12:20.340

NM: that's awesome.

79

00:12:21.940 --> 00:12:41.920

NM: Yeah. Opportunity to express yourself. That's great. And I am doing that, you know. Y

Pa14: Yeah, I mean. And she's shown over the years like. You know her desire for things is clear. You know she the stander when that was really the one thing you know

80

00:12:41.920 --> 00:12:58.490

Pa14: that she could, or that she was exposed to, and she came exposed to the gait trainer, and you could see that the opportunity to now move around while being upright was different than the Stander, and that seemed to be something she was really, you know, motivated by.

81

00:12:58.520 --> 00:13:06.870

Pa14: And then she got the adaptive tricycle which led to her sort of not wanting to be in the gait trainer or the stander.

82

00:13:06.970 --> 00:13:22.380

Pa14: It's it's just more fun. She was really able to propel herself, you know, and again she's at the same height and eye level as her peers, you know, and her brother and and other kids, her age. And so

83

00:13:22.380 --> 00:13:29.810

Pa14: I think, as as we started to see her being exposed to these things, it was clear what she liked and preferred more.

84

00:13:33.140 --> 00:13:39.720

NM: It's great, and so you mentioned the therapy. She works individually with her therapist.

Pa14: Yes

85

00:13:40.370 --> 00:13:52.030

NM: even a group that there but okay, individual. And but she's in a she's in groups at school. What activities is she in groups when I I guess, with the participation at school.

86

00:13:52.030 --> 00:14:08.660

Pa14: Well, I think a lot of times they'll do some activities that might be in groups in terms of like ot and pt in adaptive training, like when they're They're having sessions, and they're having her and her classmates use their

87

00:14:09.120 --> 00:14:26.650

Pa14: I forgot the name of the button that it is, but it's like a it's got like the speaker, and it allows them to introduce themselves and say, hello, and you know, check in for attendance and stuff like that.

NM: Morning meeting

Pa14: yes, morning meeting

88

00:14:26.650 --> 00:14:42.680

Pa14: And then, you know, in times during Pt. They may have a group activity that they'll do together. Maybe they'll have like soccer, and they'll kind of kick the ball around, or something like that, or they'll be in the pool together, and you know they're not splashing each other. But again, they're all in the same area and enjoying.

89

00:14:42.680 --> 00:14:47.170

Pa14: you know, being in a you know, an environment that the kids are all kind of having fun with

90

00:14:47.910 --> 00:14:48.680

NM: right?

91

00:14:50.150 --> 00:14:56.820

NM: All right. Last question before our survey. How many times a week does she participate in these activities, and for how long?

92

00:14:59.120 --> 00:15:12.040

Pa14: Generally speaking, the ot pt speech. I believe it's 5 times a week for an hour each at school, and then at home. She's getting those same services for half an hour.

93

00:15:12.080 --> 00:15:14.170

Pa14: twice a week.

94

00:15:14.610 --> 00:15:15.360

NM: ok

95

00:15:15.560 --> 00:15:25.080

Pa14: And then the other services, like vision, so on, are less repetitive. But for the same hour length timeframe at school.

96

00:15:25.360 --> 00:15:27.060

NM: Okay, 5 times.?

97

00:15:27.330 --> 00:15:34.030

Pa14: No, I believe it's more like I don't know if the top of my head I don't want to get the wrong thing, but 2, maybe 3 times a week.

98

00:15:34.480 --> 00:15:35.130

NM: Perfect

99

00:15:37.120 --> 00:15:38.000

NM: is great.

100

00:15:39.180 --> 00:15:51.390

NM: and with some of the activities we talked about, you share that she sometimes needs assistance. You mentioned that once she gets the assistance on the bike she can take off. Is there anything else where she needs assistance for just part of the task?

101

00:15:55.150 --> 00:16:04.930

Pa14: Well, I mean, I think you know, setting up her, Toby, and getting it, you know, positioned in a in a in a place where she can use it. She needs assistance with that.

102

00:16:04.980 --> 00:16:10.090

Pa14: and you know I would say I mean, you know she really is.

103

00:16:11.350 --> 00:16:12.160

Pa14: I mean

104

00:16:12.190 --> 00:16:31.730

Pa14: you. You have to get her in the equipment, such as the gait trainer. You have to get her AFOs on, and so on. So, and all those activities you have to assist getting her prepared to be able to do those things. Yeah, yeah, you have to get her set up to do those things, you know, in the same with the music stuff. I mean again. It's just a matter of you. You You give her the

105

00:16:31.730 --> 00:16:48.010

Pa14: the instrument, you know all the the the the bells, or whatever it is, and you know. Once she's aware of what she's able to do in controlling the response she's able to sort of get. Then she is doing things more on her own. But

106

00:16:48.010 --> 00:16:54.060

Pa14: for the most part you you have to get her prepared. You have to assistant her with getting

107

00:16:54.100 --> 00:16:55.730

Pa14: prepared to do all these things

108

00:16:55.780 --> 00:17:00.460

NM: right, and that's also in the communication like sharing with her. What about to happen?

109

00:17:00.620 --> 00:17:19.329

Pa14: Right? Right? Right right Well, and in the same way with when she's using the Toby, if she's calling out to you, know her parents or her brother, or wherever it is, it's important to respond to her to let her know that her expression is heard and valid, and and this is cause and effect, you know.

110

00:17:19.480 --> 00:17:24.210

NM: right now. And do you think she should participate in more or less of these activities. And why?

111

00:17:24.980 --> 00:17:37.500

Pa14: More because I mean, I think she shouldn't be limited into as far as how much she is allowed to participate in how much she can participate. I think she's shown incredible growth with the things that she's been exposed to, and

112

00:17:37.540 --> 00:17:55.930

Pa14: you know the the pessimism of what she would be able to do. I think she's proven lots of people wrong. So you know we, you know, often trying to remind people don't pity her, don't baby her, you know. Push her, challenge her, and if ‘child’ or well, if my daughter gets tired

113

00:17:55.930 --> 00:17:58.040

Pa14: she'll she'll go to sleep.

114

00:17:58.210 --> 00:18:07.230

Pa14: So so she she'll. She'll disconnect from the dis from the activity on her own. One way or the other she'll stop moving. She'll stop peddling. If she's done, she's done, you know. You'll know it.

115

00:18:07.300 --> 00:18:07.950

NM: Yeah.

116

00:18:09.190 --> 00:18:10.280

NM: all right.

117

00:18:10.970 --> 00:18:17.750

NM: Now, I'm going to share my screen. I actually had it up for you earlier, so you can see the survey.

118

00:18:18.190 --> 00:18:35.850

NM: So this was the survey I was mentioning earlier, and so this was developed by the National Institute of Health. There's 8 questions. It is called the parent Proxy. Physical activity survey on it. They developed for children that were undergoing a chemotherapy, and regressing, as it relates to cancer treatments. So.

119

00:18:36.230 --> 00:18:50.320

NM: But the way it works is, a parent will be given the survey, and they would answer for the physical activity for the past 7 days. How many days the child do this but what i'm going to ask you is to grade each question how you feel like it would be appropriate or valid

120

00:18:50.320 --> 00:18:57.900

NM: in children with Cp. Or a traumatic brain injury that are non ambulatory or not walking full time. Okay, like your your child.

121

00:18:57.940 --> 00:19:03.910

NM: So it's really just giving us understanding. And then why? Right? What? Why, you, you think you would, you would feel that way?

122

00:19:04.160 --> 00:19:06.350

Alright, so the first question is.

123

00:19:07.070 --> 00:19:14.600

NM: how many days did your child exercise or play so hard that his or her body got tired. How would you rate this question? And why?

124

00:19:15.570 --> 00:19:29.950

Pa14: Okay? And I'm: sorry. So i'm reading as it pertains to my child or in general to children, or non ambulatory to your child. But then I also will like you like, in terms of any feedback, maybe a parent, that

125

00:19:30.070 --> 00:19:49.670

NM: what the parent that has a child that presents like your own, maybe give me some understanding of how it may be perceived would be helpful, you know, like if there's any words that maybe that it's one way, and can give me some information about that, or just any feedback, as it relates to your child or children like your child.

126

00:19:49.900 --> 00:20:03.270

NM: And what's the rating system again?

127

00:20:05.070 --> 00:20:13.790

Pa14: I'm gonna say i'm gonna i'm gonna give it a one, I mean. especially for my child. It's it's in really

128

00:20:13.860 --> 00:20:22.050

Pa14: impossible to get a response as to whether or not a particular activity, you know, has

129

00:20:22.400 --> 00:20:37.570

Pa14: exhausted my child from that day or the day before, you know, as I said, when she's sort of tired of doing something or not interested in doing something, then you will see her disengagement from it

130

00:20:37.580 --> 00:20:40.370

Pa14: or her reluctance to do it.

131

00:20:41.320 --> 00:20:46.430

Pa14: But I can't say. I know that she's tired.

132

00:20:46.590 --> 00:20:47.470

NM: Got it?

133

00:20:49.340 --> 00:20:50.530

Pa14: It could be boredom.

134

00:20:51.000 --> 00:21:06.840

NM: Yeah, I hear you. Okay. Okay, next question: how many days did you get out? Exercise really hard for 10 min or more? How appropriate with this question be to looking at physical activity and children, and not full time. Walkers 0 not appropriate at all up to a 5 highly appropriate.

135

00:21:09.260 --> 00:21:26.160

Pa14: I mean, I think it's the 5 I mean. I think she exercises. You know it's all about perspective. So I think she exercises really hard, I think, using her Toby devices not an easy thing to do when she uses it for a good portion of her day, especially school days.

136

00:21:26.260 --> 00:21:30.090

Pa14: You know I use it for 15 min, and i'm exhausted.

137

00:21:30.140 --> 00:21:47.190

Pa14: So I think that's all. That's a testimony of how hard she's worked to build up her endurance and stamina to use that machine. I can only imagine how hard it must be to, you know. Use the gate trainer to, you know, to move around to

138

00:21:47.190 --> 00:22:01.330

Pa14: the adaptive tricycle. I'm assuming is just a little bit easier because of the fact that she's just, I guess, able to concentrate on the leg muscles where the gay trainer probably is putting a lot more

139

00:22:01.390 --> 00:22:19.040

Pa14: stress on her trunk and her body to really kind of engage that activity. So I mean I I I mean, I would say pretty much every day that she's involved in her services. She's working really hard for way more than 10 min.

140

00:22:21.010 --> 00:22:34.190

NM: Yeah. all right. That's great Number 3. How many days did your child exercise so much that he or she breathed hard. 0 not appropriate at all up to 5. Highly appropriate. How would you ring 3? That?

141

00:22:37.540 --> 00:22:44.400

Pa14: Well, I mean, I guess from my child, that I I've not seen that ever be the case where the

142

00:22:45.510 --> 00:22:56.560

Pa14: exercise led to her breathing heavy, so I guess i'm going to rate that as a 0. It doesn't seem like I've ever seen

143

00:22:56.940 --> 00:23:00.130

Pa14: for feeling or looking like she was

144

00:23:00.210 --> 00:23:06.830

Pa14: exhausted or burned out from doing anything. If anything, again. When she's tired

145

00:23:07.040 --> 00:23:08.830

Pa14: she will just fall asleep.

146

00:23:09.070 --> 00:23:10.910

Pa14: Oh, yeah, so.

147

00:23:11.200 --> 00:23:22.530

Pa14: But it's also really one of those mixed bags, because I mean she, the medication that she takes could also play into that. But I don't know I I would say it's really hard to answer that question.

148

00:23:22.780 --> 00:23:23.560

NM: Okay.

149

00:23:24.010 --> 00:23:30.210

NM: very helpful. Number 4. How many days. Was your child so physically active that he or she sweated.

150

00:23:31.470 --> 00:23:32.340

Pa14: Hmm.

151

00:23:32.370 --> 00:23:37.150

Pa14: I would say, that happens from time to time. I mean, you know.

152

00:23:37.600 --> 00:23:40.990

Pa14: I mean, especially as you get into doing more like

153

00:23:41.350 --> 00:23:50.670

Pa14: like outdoor activities, or if it's warm out, I mean, I I would say from time to time I would say she's built up a little bit of perspiration.

154

00:23:53.720 --> 00:23:57.210

NM: It's a exercise or a physical activity.

155

00:23:57.750 --> 00:24:03.000

Pa14: you know. I mean, I I think it's just from the physical activity. I mean, I think again, I think the fact

156

00:24:03.030 --> 00:24:06.350

Pa14: that I think it's. It's been

157

00:24:06.410 --> 00:24:17.000

Pa14: easier to understand how easy it is for people without several policy or any of these sort of physical afflictions to take for granted how

158

00:24:17.050 --> 00:24:31.850

Pa14: we just do things, and we're doing them so easily and so smoothly. We're not exerting ourselves to do them, and for our her to do similar things takes a lot of focus and coordination and so on. So

159

00:24:31.850 --> 00:24:41.560

Pa14: yeah, I mean, there are times She may be sweating a you know, a bit, just simply from sitting up. because it takes so much more energy and focus for her to keep her head up right.

160

00:24:42.710 --> 00:24:46.010

Pa14: you know. So

161

00:24:46.070 --> 00:24:47.630

Pa14: I would say

162

00:24:48.260 --> 00:24:54.110

Pa14: it's a very appropriate question. How many you know? I I I would get a give it a 5.

163

00:24:54.310 --> 00:25:02.340

NM: Okay. Number 5. How many days is your child exercise a place so hard that his or her muscles burned

164

00:25:03.910 --> 00:25:12.560

Pa14: hard to say. because of the fact that she's not necessarily expressing any sort of particular

165

00:25:12.700 --> 00:25:14.340

Pa14: specific

166

00:25:14.350 --> 00:25:21.200

Pa14: pain or muscle being burned, or something like that. So for me, I would say it's a 0. It's it's it's impossible to say.

167

00:25:21.330 --> 00:25:22.160

NM: Okay.

168

00:25:24.540 --> 00:25:32.190

NM: And number 6. How many days did your child exercise or play so hard that he or she felt tired?

169

00:25:37.030 --> 00:25:47.820

Pa14: I would say in general that you know, especially when she's engaged in activities at school, especially because a lot of her services are are back to back at times

170

00:25:47.880 --> 00:25:51.550

Pa14: that it will lead to her eventually needing to take a nap.

171

00:25:51.650 --> 00:25:56.520

Pa14: And so I think she does, you know, become fatigued

172

00:25:56.610 --> 00:25:59.350

Pa14: for a bit where she'll need a, you know, a break.

173

00:25:59.450 --> 00:26:05.330

Pa14: and so I think it's an appropriate question. I guess I would give it a 5.

174

00:26:05.780 --> 00:26:06.470

NM: Oh.

175

00:26:08.010 --> 00:26:09.540

NM: Number

176

00:26:09.830 --> 00:26:15.390

NM: 7. How many days. Was your child physically active for 10 min or more? How would you rate this one?

177

00:26:15.950 --> 00:26:34.640

Pa14: I think it's a very appropriate question. I would give it a 5, I mean, especially again when she's getting her services on the day she's getting a services she's definitely getting certain physical activity for more than 10 min. But in general I think you know the the the things that she will do, just because she's playful and loves to kick and stuff like that.

178

00:26:34.640 --> 00:26:40.280

Pa14: whether or not she's getting services. She's definitely, physically active for more than 10 min a day.

179

00:26:43.280 --> 00:26:52.000

NM: right? And then the last one is how many days your child, run for 10 min for more. How appropriate for this population! Would you like this question?

180

00:26:54.420 --> 00:26:59.170

Pa14: Well, I mean I mean, I think it's some interesting question.

181

00:26:59.240 --> 00:27:16.710

Pa14: She's not running so hard to judge how many minutes a day she's running. But I think if you put a timer on, when, on the days when she is most active, with her bicycling and without in the air. She will sit in her tomato seat, and her legs would just be going.

182

00:27:16.710 --> 00:27:21.390

Pa14: And I think if you time that, and want to consider that to be running.

183

00:27:21.400 --> 00:27:29.430

Pa14: then that would be way more than 10 min. Right? We we've tried that time sitting in the park to keep up with her.

184

00:27:29.460 --> 00:27:33.720

Pa14: you know, just sitting on a mention elevating our feet, and no one keeps up with her.

185

00:27:34.710 --> 00:27:43.610

Pa14: I mean we. We start when she's already been into it for like 5 min, and we all stop, and she still continues for 1015, more minutes.

186

00:27:47.300 --> 00:27:48.880

NM: How would you rate this question.

187

00:27:48.990 --> 00:28:07.130

Pa14: Oh, i'm sorry, so I guess one For 10 min or more I I give it a 5. I think it's an appropriate question. I mean I you know I I I would imagine, for different parents. Their perspective of what they considered to be running is, you know, very. But to me

188

00:28:07.130 --> 00:28:25.300

Pa14: seeing her doing that bicycling, you know, peddling their feet or her legs, I should say again, that's her way of doing what I would consider to be a physical activity simulating, running, or cycling, or whatever

189

00:28:25.300 --> 00:28:33.410

NM: classifying what that really means for the person you're asking right, like, maybe, if someone was giving this to a parent, maybe

190

00:28:33.650 --> 00:28:38.860

NM: qualifying this question, because the term run can be

191

00:28:39.040 --> 00:28:47.230

Pa14: very depending on the motion that the child is able to achieve. Does that sound about right.

192

00:28:48.040 --> 00:28:55.180

Pa14: you know, upright, and you know, hitting the ground with her feet, which I guess is the definition of running.

193

00:28:55.270 --> 00:28:58.440

Pa14: But I would challenge most

194

00:28:58.450 --> 00:29:13.440

Pa14: anyone to do what she's doing. As for as long as she does it, and then tell me that's not. You know the similar sort of impact of running other than like, you know. Get the the the fact that you're not putting your feed on the ground just to keep that movement going

195

00:29:13.530 --> 00:29:23.240

Pa14: is very similar to basically running like as if you're on a treadmill. You're not going anywhere. So yeah, yeah, actually, you're actually making a really good point.

196

00:29:24.180 --> 00:29:27.550

NM: All right, Thank you so much all right. So, as we wrap up.

197

00:29:27.640 --> 00:29:47.640

NM: I just want to give you an opportunity, as I give all the parents, and this has been so helpful to just educating me and my growth as a clinician and researcher. What are your final thoughts as it relates to physical activity for children with through palsy or brain-based disorders who are not full time walkers. Any last and final thoughts or comments as it relates to this.

198

00:29:48.280 --> 00:29:57.190

Pa14: I would just, I guess, suggest to parents if you're child, was given the opportunity to

199

00:29:57.200 --> 00:29:59.240

Pa14: the exposed to services

200

00:29:59.360 --> 00:30:09.020

Pa14: that will give them an opportunity to do physical activities to do things like using the Toby. I mean, technology is

201

00:30:09.160 --> 00:30:14.830

Pa14: always changing. And so there are things that you know my daughter's been able to to use that

202

00:30:14.880 --> 00:30:19.240

Pa14: I would have never known was even out there, and so

203

00:30:19.250 --> 00:30:26.440

Pa14: I think it's helpful. I think it's all you know. Stimulating the brain. I think it's you know it it.

204

00:30:26.700 --> 00:30:42.830

Pa14: if nothing else is something that you know she enjoys, and without it I would think like anything else. I mean, you start to kind of, you know, feel not only left out, but, like, you know, just board and listless, and

205

00:30:42.830 --> 00:30:47.810

Pa14: I mean her health would be impacted greatly without these fixed physical activities that she's able to do

206

00:30:51.020 --> 00:30:53.330

NM: you so much. I'm going to stop the recording.
