# Supplementary material for: Validity of PROMIS® Pediatric Physical Activity Parent Proxy Short Form Scale as a Physical Activity Measure for Children with Cerebral Palsy Who Are Non-Ambulatory
Source: Behav Sci (Basel). 2025 Jul 31;15(8):1042. doi: 10.3390/bs15081042 (PMC12382615; doi:10.3390/bs15081042)
Supplement: Supplementary file 1 [file behavsci-15-01042-s001.zip › Transcripts copy/Parent transcripts de-identified/Pa3.docx]

WEBVTT

1

00:00:00.000 --> 00:00:00.820

Morning.

2

00:00:00.960 --> 00:00:15.840

NM: All right. Good morning. Thank you so much for joining us today. Today we're going to talk about fiscal activity as it pertains to children with Cp. Who are not ambulatory full time. And so I have a few questions, and if I sound scripted, it is because I am

3

00:00:15.840 --> 00:00:25.040

NM: so, i'm going to ask you a few questions with some prompts and follow ups just to get some more information as it relates to each item. All right. Are you ready to start?

4

00:00:25.440 --> 00:00:32.970

Pa3: Yes, awesome. Thank you. All right. So the first question is, how do you define physical activity for your child?

5

00:00:34.780 --> 00:00:43.370

Pa3: So for ‘Child’, I would say. anytime he is it

6

00:00:43.590 --> 00:00:59.960

Pa3: either up, and so he has like an activity chair. And I would say, if he's like dancing in his activity chair he's mostly using his upper extremities, but he does kind of uses like a little bit to like push up. I would consider that physical activity

7

00:00:59.960 --> 00:01:05.760

Pa3: anytime he's up and doing, supported walking, using a gate trainer.

8

00:01:05.800 --> 00:01:09.740

Pa3: using his like single arm drive manual wheelchair

9

00:01:11.480 --> 00:01:21.200

Pa3: anytime. He's on the floor, kind of rolling around, playing with toys on the floor, or playing with his brother and sister. That's kind of

10

00:01:22.560 --> 00:01:27.510

Pa3: anytime he's engaging and moving. I guess that's what I would consider physical activity for him

11

00:01:29.430 --> 00:01:44.820

NM: awesome, and the Department of Health defines physical activity as any activity that encompasses energy extended and activation of skeletal muscles. Does this definition change your mind? About how you define physical activity for your child. Why or why not?

12

00:01:45.730 --> 00:01:58.340

Pa3: If we're going to define it like that, then I would probably say, even when ‘child’ is sitting up statically in his activity chair. He probably is doing

13

00:01:58.530 --> 00:02:10.280

Pa3: kind of a low level of physical activity and thinking about it. He does fatigue in that in his activity chair. It does take a while, but he does fatigue.

14

00:02:11.150 --> 00:02:16.170

Pa3: So I would probably add that for him to if we're looking at that definition.

15

00:02:16.830 --> 00:02:18.370

NM: Okay, thanks.

16

00:02:19.180 --> 00:02:24.600

Pa3: And how do you think physical activity differs from rest specifically in this population?

17

00:02:26.080 --> 00:02:38.070

Pa3: So I would say that if my typically developing 3 or 5 year old were to sit down and take a break.

18

00:02:38.390 --> 00:02:45.030

Pa3: That would be a rest period for them. They're not expending a ton of energy.

19

00:02:45.220 --> 00:02:54.900

Pa3: but I know with ‘child’ when he does sit down. He's also using a lot of energy to kind of maintain his balance and to keep himself upright.

20

00:02:56.200 --> 00:03:08.740

Pa3: So with ‘child’, I would probably say, when he's like laying down. That's probably rest for him. But any other position for him I would anticipate that he is using a lot more

21

00:03:09.680 --> 00:03:14.170

Pa3: energy. Than my 2 typically developing kids.

22

00:03:15.540 --> 00:03:18.630

NM: Absolutely. Okay. Next question.

23

00:03:19.670 --> 00:03:32.920

NM: What activities would you consider your child does is physical activity, and you've paid me some. So you don't have to give me anything in addition, or you know you kind of want to summarize the the main activities that you consider

24

00:03:33.000 --> 00:03:34.650

NM: physical activity for him.

25

00:03:37.110 --> 00:03:48.670

Pa3: So I would say that the activities that we kind of make a point of doing on a regular basis to really get him up, and moving in to kind of get his whole body engaged would be the supported walking

26

00:03:48.960 --> 00:03:54.380

Pa3: time, and, like his power stander, his gate trainer.

27

00:03:56.050 --> 00:04:03.480

Pa3: And then, when he does, you know, like his Pt. And his ot in his clinic, his transitions between

28

00:04:05.840 --> 00:04:09.510

Pa3: equipment, I would say, would be physical activity, and then

29

00:04:09.630 --> 00:04:18.269

Pa3: the time he spends on the floor kind of rolling around, playing, transitioning in and out of W sitting, and all of that good stuff

30

00:04:19.970 --> 00:04:23.170

NM: question about the power stander. What is that? Exactly

31

00:04:23.660 --> 00:04:30.490

Pa3: so ‘child’ has a custom piece of equipment. It's a kid walk with like a

32

00:04:30.520 --> 00:04:35.700

Pa3: steel plate on the bottom, and we put an E fixed system on it so he could be in standing

33

00:04:35.730 --> 00:04:37.900

Pa3: and still drive around.

34

00:04:38.140 --> 00:04:41.360

Pa3: What I like about it is it's not like a

35

00:04:42.090 --> 00:04:54.760

Pa3: it's not like a zing stander, or like a rifton stander where he's really locked in, he can bend his knees and kind of sit and take a break or stand up. And the

36

00:04:55.760 --> 00:04:57.030

dynamic

37

00:04:59.420 --> 00:05:18.910

Pa3: Yes, the dynamic structure of the Kidwalk does allow him to do some weight shifting side to side, and he can do like a little bit of trunk rotation in it. So he he does work pretty hard in that. He does fatigue relatively quickly when he's in it. So I would say that that is a good example of like a physical activity for him, too.

38

00:05:19.720 --> 00:05:24.830

So question he's driving it with a joystick.

Pa3: Yeah, he drives me with a joystick.

39

00:05:25.460 --> 00:05:45.010

NM: That's great.

Pa3: I'll send you a video of it.

NM: Yeah, that's awesome. I i'm familiar with the kidwalks. I'm trying to think of the e-fix where the e-fix system is going on there.

Pa3: So we pop the, put the battery pack on the bottom, on the bottom. So we put a steel plate on it, so he's not on the ground. He's standing on the steel plate, and then we attached a joystick, and we popped

40

00:05:45.160 --> 00:05:49.510

Pa3: the Kidwalk off and we put the E fix system wheels on.

41

00:05:49.530 --> 00:05:59.460

NM: Hmm. Awesome. Yeah. I had a kid with a power chair. We we. We've used the E fixed wheel. They're really great. They're They're great for small.

42

00:05:59.890 --> 00:06:09.620

NM: you know, small power of power, dynamic and for portability. So that's

Pa3: 100 percent We've been very happy with it.

43

00:06:11.560 --> 00:06:28.970

NM: Wonderful, All right. Yeah. I would like to see it if you don't mind all right. So in in terms of a prompt Well, you're not unsure, but if you were unsure, you gave me some of the child habitual activities. So you talked about adaptive

44

00:06:29.220 --> 00:06:45.610

NM: equipment. So i'm just gonna pass this question because you answered all of all of all of the there was one piece in here that talked about using a playground swing or adaptive swing, and then does he engage in reaching or ball toss? Would you consider that physical activity

45

00:06:47.090 --> 00:06:49.670

Pa3: sorry? Does he reach involved? What

46

00:06:49.820 --> 00:06:59.990

NM: does he participate in my ball toss or reaching, and then he does he ever use an adaptive play playground swing? And would you consider those 2 things at physical activity?

47

00:07:01.030 --> 00:07:10.760

Pa3: So he does. So there he doesn't like the adaptive swings at the playgrounds anymore. I'm not…I mean he'll go in for a little bit.

48

00:07:11.930 --> 00:07:21.060

Pa3: He's usually holding on, so I would consider that probably a little bit of physical activity. We have a disc swing at our house

49

00:07:21.160 --> 00:07:30.390

Pa3: that he he'll lay on his stomach, and he'll play on that. And that's definitely physical activity, because he likes to touch the floor now, so he, like moving himself all around.

50

00:07:30.460 --> 00:07:41.620

Pa3: He does enjoy throwing balls. So we have a one year old Golden Doodle, who loves to play, fetch, and he'll come over and drop the ball in his lap, and he'll throw it.

51

00:07:41.680 --> 00:07:47.590

Pa3: He doesn't catch, but he does really enjoy throwing, and I would consider that physical activity for him.

52

00:07:48.200 --> 00:07:48.980

NM: Okay.

53

00:07:49.960 --> 00:07:55.400

NM: Now, how do you how do related services such as Pt. Ot.

54

00:07:55.450 --> 00:07:59.190

NM: I don't know if he gets vision hearing services relate to physical activity.

55

00:08:00.240 --> 00:08:05.570

Pa3: so he does have vision services. He does not have hearing services.

56

00:08:06.230 --> 00:08:12.820

Pa3: I know that. So he has a cortical visual impairment, and I know that

57

00:08:13.430 --> 00:08:28.360

Pa3: it is a lot more work, and it takes a lot more out of him to use his vision. So we'll see him when he starts to fatigue. We'll see him kind of shut his vision off, and there's definitely a correlation with him between

58

00:08:28.500 --> 00:08:38.480

Pa3: his physical fatigue and how successful he is that using his vision, especially when it comes to like depth perception. So when he.

59

00:08:48.520 --> 00:08:58.740

Pa3: or you know, cut a corner kind of short,

NM: you cut out for a second right when you were saying you can be a correlation to use of his use of his position.

60

00:08:58.770 --> 00:09:02.260

NM: and especially as it relates to when they you cut out.

61

00:09:02.520 --> 00:09:13.620

Pa3: Oh, especially as it relates to depth perception, we find that his depth, perception, as he fatigues physically. really decreases.

62

00:09:14.870 --> 00:09:15.800

Pa3: so

63

00:09:15.940 --> 00:09:18.260

Pa3: we know with

64

00:09:18.400 --> 00:09:23.800

Pa3: with his vision, even if he's in like an activity chair, and he's extremely well supported.

65

00:09:26.240 --> 00:09:34.390

Pa3: Vision is something that fatigues him, and i'm not sure. I mean, I guess, from a I guess we could call it a physical activity

66

00:09:34.510 --> 00:09:37.790

Pa3: just with the motor control the eyes, but

67

00:09:38.110 --> 00:09:43.790

Pa3: it's more of like the processing piece for him like that internal

68

00:09:43.960 --> 00:09:44.850

Pa3: kind of

69

00:09:46.080 --> 00:09:49.250

Pa3: what the brain is processing from that visual standpoint.

70

00:09:49.410 --> 00:09:55.900

Pa3: And then, with related services with Pt. And Ot, he definitely works

71

00:09:56.290 --> 00:10:14.370

Pa3: harder when they're challenging him. But I I feel like a lot of the support that he's getting from. Pt: and OT. Especially in school, is really trying to make his life easier. So I would hope that in school. His related services are reducing his energy expenditure.

72

00:10:14.610 --> 00:10:21.300

Pa3: but, like his outpatient clinical services, he I mean. He goes there, and he gets a great work out. He sweats every time.

73

00:10:21.380 --> 00:10:26.300

Pa3: so I think it depends on what their goals are. I don't know if they answer your question. Sorry

74

00:10:26.960 --> 00:10:37.310

NM: you touched on a few different things you touched on on me school basis, I mean, we we usually don't go there. But I say, why not? Okay, so comparing school-based therapy to

75

00:10:37.320 --> 00:10:41.890

NM: clinical or outpatient, medically specific

76

00:10:42.140 --> 00:10:45.750

NM: therapy, and your your point about in school

77

00:10:46.210 --> 00:10:53.340

NM: trying to make his life easier. Can you go a little bit more into that what you expect that to look like as it relates to the therapist

78

00:10:53.590 --> 00:10:54.940

NM: intervention

79

00:10:55.200 --> 00:11:04.480

Pa3: absolutely so in school. A lot of like that school base PT and OT is ideally kind of targeting

80

00:11:04.700 --> 00:11:10.380

Pa3: improving participation in access to that general education curriculum. So

81

00:11:10.440 --> 00:11:17.110

Pa3: when he's in the classroom, you know they're working on ways that ‘child’ can participate with his peers.

82

00:11:18.940 --> 00:11:20.460

Pa3: so that you.

83

00:11:20.950 --> 00:11:23.110

Pa3: looking for ways that he can participate

84

00:11:23.170 --> 00:11:30.960

Pa3: and not be super fatigue. So, for example, they work on a lot of seating modifications. So if they're doing circle time.

85

00:11:30.970 --> 00:11:45.110

Pa3: ‘child’ has a couple of options. He has, like a small Adirondack here, or bean bag depending on how tired he is, and what time of day it is, so that he can be there with his peers, and participate in whatever activity they're doing.

86

00:11:45.600 --> 00:11:52.380

Pa3: but be supported in a way that he's available to kind of pay attention.

87

00:11:52.910 --> 00:12:02.160

Pa3: And then, for example, in like gym, if they're doing scooter boards more recently, they've kind of

88

00:12:02.570 --> 00:12:14.670

Pa3: recently they put 3 scooter scooter boards together. So he doesn't have to hold up his head, and he doesn't have to worry about his legs. It kind of makes it a little bit more free rolling. So that's an adaptation that his Pt Made to make it

89

00:12:14.750 --> 00:12:20.450

Pa3: easier for him to participate in the actual activity, first

90

00:12:20.510 --> 00:12:24.060

Pa3: kind of just exhausting him by just trying to

91

00:12:24.170 --> 00:12:37.360

Pa3: keep himself on the scooter board. and then, in like as far as occupational therapy and stuff goes with his school. They're looking at ways for him to

92

00:12:37.680 --> 00:12:41.290

Pa3: again make things easier. So

93

00:12:41.710 --> 00:12:44.150

Pa3: in

94

00:12:44.310 --> 00:12:51.210

Pa3: his ot, and I collaborate pretty regularly, so he uses like a spoon that's a little bit weighted, and has like a bigger grip.

95

00:12:51.330 --> 00:13:00.140

Pa3: so that he has more control, and he doesn't have to try as hard to get bites in his mouth. He's more consistent getting bites in his mouth, so he's having to

96

00:13:00.250 --> 00:13:08.710

Pa3: kind of load that spoon less amount of time. He uses tabletop scissors

97

00:13:08.960 --> 00:13:16.700

Pa3: versus like even attempting like regular scissors, or even tearing paper, because that bimanual task was really hard for him

98

00:13:19.470 --> 00:13:29.240

Pa3: when he is doing coloring and writing. We know that he is more successful with like the thicker crayola markers or kind of the jumbo crayons

99

00:13:29.480 --> 00:13:35.800

Pa3: because it takes less of that fine motor control.

100

00:13:36.300 --> 00:13:42.940

Pa3: So I think, when he goes to like an outpatient clinical setting, like he's there for a workout.

101

00:13:43.130 --> 00:13:49.090

Pa3: And that's typically what he gets. But in school that that therapy

102

00:13:49.760 --> 00:13:52.620

Pa3: is kind of geared towards

103

00:13:52.660 --> 00:13:56.260

Pa3: him, being able to participate to the fullest extent.

104

00:13:56.470 --> 00:14:04.640

Pa3: and for him that usually means reducing the physical exertion in those activities

105

00:14:04.760 --> 00:14:05.530

Pa3: and

106

00:14:06.630 --> 00:14:09.030

Pa3: so I think that might can be…

107

00:14:10.210 --> 00:14:23.410

NM: that that is that awesome? Thank you. That's very helpful. And does your child do these activities alone like so once he's gotten the modification, Is he able to now engage without a person setting him up, or does somebody set him up

108

00:14:24.950 --> 00:14:33.340

NM: and still help him like hand over hand and get through some of the tasks. and that, and and why or why not? If he needs assistance with some of these activities, we talked about.

109

00:14:33.900 --> 00:14:39.610

Pa3: So that's funny. You brought that up. So that is something that we are pushing

110

00:14:39.690 --> 00:14:43.480

Pa3: very strongly in the school system.

111

00:14:44.420 --> 00:14:56.600

Pa3: I've worked in the school system before, and I think one of my biggest soap boxes is developing independent learners. A lot of our children who have special needs

112

00:14:56.840 --> 00:15:04.410

Pa3: tend to have a para or somebody, or some adult that's providing constant prompting for them to

113

00:15:04.510 --> 00:15:20.450

Pa3: start a project for them to do the next step for them to finish, and then redirecting them back to whatever activity it is. So we are in the process of really pushing to reduce the adult prompt and in increases independence. So

114

00:15:20.750 --> 00:15:24.230

Pa3: right now, the way that they have found.

115

00:15:24.440 --> 00:15:37.890

Pa3: or one way they've been successful is they have, like rubber made drawers like a tower of 3 of them, and they have broken it down into steps of like an assignment. So each

116

00:15:37.990 --> 00:15:42.920

Pa3: drawer has a different step of the project or the assignment that he's doing.

117

00:15:44.450 --> 00:15:50.270

Pa3: and they set it up for him, and then his para or his teacher steps back.

118

00:15:50.460 --> 00:15:54.000

Pa3: and we're just working on him completing it.

119

00:15:55.320 --> 00:16:20.870

Pa3: We don't care if it's this, I mean, we want it to be done correctly, but we don't necessarily care if it's done correctly, but we're working on building his independence, so that in the future, when he's giving a task, he doesn't need the adult prompting. So we are definitely working towards that. But I wouldn't say that we're there yet, where he's being set up with an activity and step in, and, like an adult, is completely stepping back

120

00:16:20.940 --> 00:16:22.220

Pa3: they are.

121

00:16:22.460 --> 00:16:28.770

Pa3: They'll help with parts of it, like if he's cutting paper. Somebody needs to hold the paper.

122

00:16:28.840 --> 00:16:31.520

Pa3: If he is the

123

00:16:31.550 --> 00:16:33.950

Pa3: you know we've got them to the point now, where

124

00:16:34.550 --> 00:16:46.170

Pa3: he will, you know, take his lunch box, and we've put like key rings, or we've extended the zippers using like paracord so that he can

125

00:16:46.870 --> 00:16:54.150

Pa3: get his lunch back, or somebody will put his lunch box in front of him, fill and zip it, and then we've tried to make a lot of

126

00:16:54.340 --> 00:17:08.609

Pa3: the snacks or food that's in his lunch box accessible for him. So we're building that independence kind of across routines. So we are not there where they can set them up and walk away completely. But we're building towards that.

127

00:17:09.119 --> 00:17:19.680

NM: Well, that's great, though, great goals. I like the rubbermaid idea to just to have it sequential. And so he learns to do like top 1, 2, and 3.

128

00:17:19.680 --> 00:17:34.240

NM: You can just put different things in the the bins is just my understanding depending on the task. Is that how you?

Pa3: Yeah, Absolutely. And they usually put the most motivating one in the bottom, so that he learned that he has to follow

129

00:17:34.300 --> 00:17:41.740

Pa3: that sequence. Because I I mean it's It's tricky for kids like he's very charismatic. He's got a great smile.

130

00:17:41.810 --> 00:17:47.890

Pa3: It's really good. I just being like I can't do it. Do it for me, and then like.

131

00:17:48.030 --> 00:17:56.060

Pa3: and then I talk to them, and i'm like no listen at home. He's pretty independent, you know, at home once he's in his power. Mobility, like

132

00:17:56.680 --> 00:18:07.310

Pa3: we set things up on. We have like a countertop, and then our dining room is no longer a dining room. It's like a kids craft area, so we have a table in there, and then we have a table in

133

00:18:07.980 --> 00:18:10.700

Pa3: like like a kitchen table.

134

00:18:10.810 --> 00:18:20.550

Pa3: and so we'll put activities out, because that that's the hard thing like he has a hard time getting things out. But we put things out, and then he kind of drives around and

135

00:18:20.570 --> 00:18:23.550

Pa3: does what he wants, and there, there, if he wants to.

136

00:18:25.380 --> 00:18:36.870

Pa3: and we've tried to make our pantry accessible, so that he can go and like, Go and get a snack if you want. So at home he does a lot more things independently. So we're trying to reinforce that

137

00:18:37.360 --> 00:18:38.400

Pa3: at school.

138

00:18:39.530 --> 00:18:54.920

NM: That's wonderful, all right. So how many times the last question before we get to the survey, how many times a week. Does your child participate in these activities? And do you have like a a framework, or how long he's able to endure some of these tasks.

139

00:18:58.210 --> 00:18:59.900

Pa3: So

140

00:19:01.000 --> 00:19:10.210

NM: let's do the gait training to start like I know you have a lot of

Pa3: the gate trainer…So between the gate trainer and like supported walking.

141

00:19:10.810 --> 00:19:16.550

Pa3: I would say he does that daily, either one of the other or both.

142

00:19:16.850 --> 00:19:30.980

Pa3: We try it like if he goes to the bathroom, you know if he. you know, if he wants to like change between equipment and stuff, we're not necessarily like carrying him anymore. So he does a lot of supported

143

00:19:31.390 --> 00:19:37.790

Pa3: walking or in his gait trainer with his gate trainer. He still needs a little bit of help.

144

00:19:38.160 --> 00:19:42.320

Pa3: kind of maneuvering it. But once we get them up, and these and we're going. It's kind of

145

00:19:42.400 --> 00:19:51.900

Pa3: so, I would probably say, between the gate trainer and supported walking. And this is like after school hours during the week, I would probably say, we're at like

146

00:19:52.290 --> 00:19:57.270

Pa3: 20 min to an hour, depending on how tired he is.

147

00:19:58.580 --> 00:20:08.250

Pa3: and then at school he has another version of the power stander, so he's in that, and then they do try to do some supported walking at school, so

148

00:20:08.460 --> 00:20:21.230

Pa3: I I wouldn't really be able to gauge how much he is doing in school, but I would probably say at least a half hour of, or maybe like, 20 min of supported walking transitions.

149

00:20:22.230 --> 00:20:23.800

NM: and then

150

00:20:24.460 --> 00:20:41.530

NM: at home, in terms of like the activities that we're getting to the upper extremity of you so like the crafting, or I mean, would you consider the the driving, just driving the power, mobility as a physical activity task, and if you do, how long does he do that?

151

00:20:41.890 --> 00:20:46.500

Pa3: Yeah, so if we were to combine so like eating.

152

00:20:47.710 --> 00:20:54.200

Pa3: He does like he likes to play with his tablet. He likes to play music. he does a lot of dancing.

153

00:20:54.680 --> 00:21:06.200

Pa3: He like, to help with like cooking and playing with toys and stuff. So I would say, the majority of his afternoon is probably physical activity for his up like upper extremities between the driving, and whatever else he's doing. So

154

00:21:06.770 --> 00:21:15.390

Pa3: it gets home from school about 4, or probably 6, a good chunk of like 2 and a half to 3 h where he's.

155

00:21:15.460 --> 00:21:19.280

Pa3: he's pretty actively engaged in an activity or driving.

156

00:21:20.040 --> 00:21:25.790

NM: Okay, that's great. So he's doing that for a couple of hours. Then now, if you count all of it.

Pa3: Yeah, he doesn't like he doesn't stop moving

157

00:21:26.330 --> 00:21:37.910

NM:. Yeah, he's he's he's pretty active, which is, which is awesome. Okay, Someone just put 60 to 90 min, because i'm sure it's more than that. But

158

00:21:38.440 --> 00:21:40.240

Pa3: yeah.

159

00:21:40.850 --> 00:21:42.780

NM: and then do you?

160

00:21:43.070 --> 00:21:53.200

NM: So we talked about this on that. So he does need some assistance. But you work at home, though you said he doesn't need it's just getting him in the equipment that's about it. Then he goes.

161

00:21:53.310 --> 00:22:04.160

Pa3: Yeah, so it's getting him in the equipment, helping him like, you know. If this tablet dies we have to plug it in from. If he has to go to the bathroom.

162

00:22:04.430 --> 00:22:17.360

Pa3: He can usually go in and into the pantry, and, like get himself a snack if he's in power mobility. But then we usually have to help him like, open it, and then, like I mean his whole.

163

00:22:17.420 --> 00:22:23.540

Pa3: He eats independently. Once we set him up. He drinks, you know, from

164

00:22:24.500 --> 00:22:28.950

Pa3: either, like a a small open cup, or like a cup of a straw.

165

00:22:31.230 --> 00:22:38.900

Pa3: and then, like his whole bedtime routine, he's dependent he'll do supported walking, but it's like on and off the toilet teeth brushing, dressing.

166

00:22:38.980 --> 00:22:45.260

Pa3: He'll participate in dressing by like pushing his arms and legs in. but that's mostly

167

00:22:45.520 --> 00:22:50.940

Pa3: I mean we dress him and stuff like that. So I would say that routine piece.

168

00:22:51.360 --> 00:22:53.320

Pa3: He he's completely dependent.

169

00:22:55.110 --> 00:23:01.560

NM: That's great. And then the the final question, I mean. And you touch on this. But i'm just gonna ask you kind of just, general.

170

00:23:01.610 --> 00:23:11.070

NM: Do you think he should participate more or less in these in these activities? And why Why do you want him to be more independent? What is it? What is the thing that you really

171

00:23:11.270 --> 00:23:17.180

NM: your your big? Why, as it relates to him, participating more or less in these physical activity tasks.

172

00:23:17.470 --> 00:23:26.740

Pa3: So I mean I would love for him to participate more. I think the biggest barrier for us is honestly, time and like hands.

173

00:23:26.790 --> 00:23:39.690

Pa3: My husband travels a lot, and our child-care ends about like 4 4:30. So if it were just ‘child’ and I, I think he would be like a lot more involved in a lot more

174

00:23:39.840 --> 00:23:50.620

Pa3: active because I would be able to, you know, supported walk him while he carries his dishes to the thing which we do sometimes, but not nearly as much as I would like to. You know. You know those little things that.

175

00:23:50.840 --> 00:23:55.900

Pa3: you know. I'm also attending to a 3 year old and a 6 year old. I'm taking dinner, and my life is crazy.

176

00:23:56.010 --> 00:24:02.610

Pa3: and you know I think my long term end goal is that I want him to have options when he's older.

177

00:24:03.220 --> 00:24:06.790

Pa3: you know, if he wants to live with my husband, and I

178

00:24:07.230 --> 00:24:11.040

Pa3: I think that that's wonderful. If he wants to live in something like

179

00:24:11.440 --> 00:24:24.850

Pa3: an in law suite, or an apartment off of wherever we live and be more independent. I think that that's great. If he doesn't want to live with us at all, and he wants to live in like a group home with other young adults. Then I want him to be able to.

180

00:24:25.450 --> 00:24:27.010

Pa3: I want him to be able to make choices

181

00:24:27.030 --> 00:24:31.100

PA3: when he gets older, and I want him to be able to have choices. So I think.

182

00:24:31.200 --> 00:24:34.450

Pa3: when we're looking at like a long term goal.

183

00:24:34.520 --> 00:24:41.830

Pa3: I think the more we do now when he's younger, and the more that we really push the independence, and we help him build

184

00:24:42.170 --> 00:24:44.420

Pa3: the skills to be independent.

185

00:24:44.470 --> 00:24:49.180

Pa3: It just opens more opportunities for him in the future. So he has.

186

00:24:49.260 --> 00:24:54.690

Pa3: Yeah, so that he can make choices about his future. And I think that's really important to my husband and I.

187

00:24:55.790 --> 00:24:56.880

NM: Well said.

188

00:24:56.890 --> 00:25:00.090

Pa3: first of all. I'm just gonna pause and say this you, are doing an amazing job.

189

00:25:00.610 --> 00:25:03.440

NM: you know. You should. You should know that.

190

00:25:03.560 --> 00:25:07.280

All right. Last thing i'm gonna pull up

191

00:25:07.760 --> 00:25:18.720

NM: the promise which you're not a stranger to. So i'm just gonna you know you don't have to read this. Don't worry about it. I'll just i'll. I'll say the questions as we go through them.

192

00:25:18.730 --> 00:25:27.550

NM: But what I'm going to do this is a this is an 8 question. Parent proxy, physical activity survey, which was developed by the Nih specifically for

193

00:25:27.850 --> 00:25:45.960

NM: families of children that were not typically developing or undergoing a a progressive neurological disorder, a cancer, or some kind of post surgery with the the children can't really answer for themselves. So this was the closest thing I felt like we could start with for this population. However.

194

00:25:45.960 --> 00:25:58.970

NM: I want to ask parents how appropriate or how valley would you say this assesses physical activity, intensity for a population with Cp. Who are non ambulatory? Okay. So i'm going to ask you to rate each question

195

00:25:59.510 --> 00:26:09.580

NM: on a scale from 0 to 5 0, not related at all. Not and not appropriate at all to 5 up to 5, which is highly appropriate for this population. And then i'm going to ask you to tell me why.

196

00:26:09.650 --> 00:26:13.020

NM: Okay? Alright, so for the first question.

197

00:26:13.590 --> 00:26:26.750

NM: The promise asks how many days a year it's out exercise or play so hard that his or her body got tired. How appropriate would you rate this question; for to a parent that has a child that was Cp. Who was not walking full time.

198

00:26:30.360 --> 00:26:33.640

Pa3: I would rate that probably at a

199

00:26:33.940 --> 00:26:39.370

Pa3: 4, and I would rate it as a for, because I think that

200

00:26:39.380 --> 00:26:50.930

Pa3: and kids who are non ambulatory, that physical fitness piece is lost. So I think it's a not lost, but a lot of times. I don't think it's the focus.

201

00:26:50.930 --> 00:27:04.890

Pa3: And it is really important for kids who have a physical disability to be engaged in physical physical fitness and physical activity, and I think that it's a good question because it kind of puts that on the parents radar.

202

00:27:06.270 --> 00:27:09.850

Pa3: The reason i'd give it a 4 verse of 5 is because

203

00:27:10.040 --> 00:27:13.600

Pa3: I think most parents would have a hard time

204

00:27:14.040 --> 00:27:24.880

Pa3: kind of conceptualizing what would constitute physical activity for their child. So i'm not sure that the answer would come back with a lot of.

205

00:27:25.420 --> 00:27:26.340

Pa3: I guess

206

00:27:26.440 --> 00:27:30.360

Pa3: accuracy across families.

207

00:27:34.380 --> 00:27:36.940

NM: Very good. The point.

208

00:27:39.520 --> 00:27:41.370

NM: Okay. So the next question

209

00:27:41.900 --> 00:27:53.040

NM: for number 2. How many days did your child exercise really hard for 10 min or more? How would you rate this question? 0? Not the appropriate up to 5 highly appropriate.

210

00:27:54.740 --> 00:28:10.350

Pa3: I would probably give it a 4 again, and for similar reasons, I think it's important for kids who have physical disabilities to have that kind of parents and caregivers radar that that physical fitness component is important.

211

00:28:10.850 --> 00:28:13.080

Pa3: But I also

212

00:28:13.740 --> 00:28:20.440

Pa3: really know about the accuracy, because. like really hard for

213

00:28:21.210 --> 00:28:33.710

Pa3: a parent might mean. They have to go to the gym, and they have to play in sweat. But really hard, for a child who has a physical disability might mean like I worked really hard today.

214

00:28:34.200 --> 00:28:38.720

Pa3: you know, sitting up at the table, or I worked really hard today, just

215

00:28:39.160 --> 00:28:41.980

Pa3: getting from like the kitchen to the living room.

216

00:28:43.760 --> 00:28:46.150

Pa3: So I I don't know.

217

00:28:46.810 --> 00:28:47.620

NM: Yeah.

218

00:28:48.830 --> 00:28:49.890

NM: Okay, that's all

219

00:28:50.000 --> 00:28:57.840

NM: all right for the third question: how many days is your child exercise so much that he or she breathes heart. How would you rate this one?

220

00:29:03.210 --> 00:29:15.100

Pa3: I'll i'll ship this one a little bit. I think that that one would be 5, because I think that's a little bit more tangental. And the fact that they'll be able to see their child breathing hard.

221

00:29:18.670 --> 00:29:20.200

NM: No, okay.

222

00:29:21.690 --> 00:29:22.460

Pa3: yeah.

223

00:29:22.870 --> 00:29:25.520

NM: that's good. Thank you. Number 4.

224

00:29:26.110 --> 00:29:35.990

NM: How many days was your child so physically active that he or she sweated. How would you rate this one for children that are not full time. Walkers, do you? Much as well as for

225

00:29:36.980 --> 00:29:37.760

Pa3: Okay.

226

00:29:40.750 --> 00:29:50.150

Pa3: Yeah, I would probably give that one a 5 to, because again, you can see that they're sweating, or feel that they're sweating first. Just kind of

227

00:29:50.720 --> 00:29:54.080

Pa3: guessing. I'm a physical exertion.

228

00:29:57.770 --> 00:30:07.620

NM: And number 5. How many days did your child exercise or play so hard that his or her muscles bound. How appropriate would you think this question would be for this population?

229

00:30:08.130 --> 00:30:09.520

Pa3: Hmm.

230

00:30:09.620 --> 00:30:14.770

Pa3: I would say I would put that one down further, probably about a 3, because you're not.

231

00:30:14.780 --> 00:30:20.740

Pa3: I don't know how you would know that and trade. ‘child’ like

232

00:30:22.800 --> 00:30:26.070

Pa3: I mean. I know that he's like he gets sore

233

00:30:26.620 --> 00:30:37.620

Pa3: when he's been sitting up right for a long time. He's been getting like tension headaches and stuff, so he'll say like his upper back and neck or sort, and that would kind of be my qualifier for him. But

234

00:30:37.870 --> 00:30:48.920

Pa3: is it because he exerted himself so intensely that it's there, or is that one of those like overuse types. muscle tension

235

00:30:51.480 --> 00:30:56.160

Pa3: issues where it's kind of compiling on each other from several days. But, like

236

00:30:56.220 --> 00:31:06.730

Pa3: I mean, he never. He never really says like, oh, my legs are sore, my arms are sore like he'll say my legs are tired, and I would probably take that from him as a

237

00:31:07.100 --> 00:31:11.360

Pa3: as that point where he's getting a little bit sore because he's done so much.

238

00:31:11.610 --> 00:31:13.580

Pa3: But I don't know.

239

00:31:13.820 --> 00:31:21.510

Pa3: unless the child was able to really are we assuming that the child is able to tell you that they're so? Are we assuming that the parents are facing?

240

00:31:22.000 --> 00:31:31.580

NM: No, the parent would have to understand, like just the the child can be verbal or can be to the point where they can articulate. But it's really from the parents perspective.

241

00:31:33.310 --> 00:31:39.270

Pa3: Yeah, I again. I think that these questions are good, because I think they increase awareness about physical fitness. But I,

242

00:31:39.640 --> 00:31:44.180

Pa3: as far as like accuracy and gathering data.

243

00:31:44.240 --> 00:31:48.410

Pa3: i'm not sure like between parents and between children, if

244

00:31:49.030 --> 00:31:54.100

Pa3: there would be a whole lot there. But I mean within the family it might be a good

245

00:31:54.640 --> 00:31:57.850

Pa3: I mean, it might be a good marker, because it's going to be based on

246

00:31:59.460 --> 00:32:03.410

Pa3: it will be, you know, we kind of based on their opinion over time.

247

00:32:03.820 --> 00:32:05.420

NM: Yeah.

248

00:32:05.620 --> 00:32:10.220

Pa3: I don't know. I feel like I'm not really answering your question. You are.

249

00:32:10.450 --> 00:32:14.440

NM: I think I think it's tough, right? I think

250

00:32:15.000 --> 00:32:27.100

NM: you don't know how parents perceive things, too, when you ask a question, I mean your parents, so you can see it from both lenses as a Pt. And a parent. So yeah, I think I I I can hear where you're coming from.

251

00:32:27.130 --> 00:32:37.120

NM: And so that was Number 5. So number 6. How many days your child exercise a place so far that he or she felt tired.

252

00:32:38.120 --> 00:32:41.230

Pa3: How did you read that

253

00:32:44.300 --> 00:32:54.960

Pa3: again? I think that fatigue ability factor with kids is a little bit more apparent because they like get tired. They get irritable. They get whiny.

254

00:32:55.400 --> 00:32:58.330

Pa3: So maybe like a for for that one. Okay.

255

00:32:59.560 --> 00:33:05.140

Pa3: I I feel like this is the pain rating scale right? Like it's like 0 to 10.

256

00:33:05.160 --> 00:33:11.350

Pa3: Yeah. And that scale drives me insane.

257

00:33:12.370 --> 00:33:18.310

NM: And number 7. How many days is your child physically active for 10 min or more.

258

00:33:23.730 --> 00:33:31.950

Pa3: I would. I would give that a 4 again, because I think you can tell when they're active. But then we're also looking at what the

259

00:33:32.460 --> 00:33:40.100

Pa3: what do the parents consider physical activity, and that physical exertion from the child. And I think that that's really hard to gauge.

260

00:33:41.400 --> 00:33:45.690

NM: Yeah. And then

261

00:33:46.300 --> 00:33:56.260

NM: number 8. How many days did your child run for 10 min or more? How would you rate that one in terms of appropriate in this population 0 not appropriate at all. 5 highly appropriate.

262

00:33:58.770 --> 00:34:05.860

Pa3: I would give that us. How long? How let me do that? I mean

263

00:34:07.860 --> 00:34:19.150

Pa3: I would probably give it a 3 only because I know how I would answer with ‘child’ I would look at, based on the exertion of what he's doing with either his gate trainer or

264

00:34:19.219 --> 00:34:20.730

Pa3: his

265

00:34:20.810 --> 00:34:27.090

Pa3: supported walking, or with like one of with his like single arm, drive manual wheelchair. And

266

00:34:27.260 --> 00:34:31.679

Pa3: how quickly he's moving, and what that exertion looks like for him.

267

00:34:32.480 --> 00:34:33.449

NM: Yeah.

268

00:34:33.830 --> 00:34:34.440

Pa3: Yeah.

269

00:34:35.300 --> 00:34:37.100

Pa3: Another Heck.

270

00:34:37.380 --> 00:34:48.280

NM: Okay. So we're at the end. But I like to ask everybody who our interview to share any last words, as it relates to physical activity in this population. Anything you would like any final thoughts.

271

00:34:52.230 --> 00:34:56.960

Pa3: No, I mean, I makes. I'm excited to see kind of what comes out of this like

272

00:34:57.400 --> 00:35:09.240

Pa3: big picture wise, because I know that. like when I was in grad school, we looked at the step test at the

273

00:35:10.540 --> 00:35:18.280

Pa3: as a like a determiner of like physical exertion, and kids with physical disabilities, and

274

00:35:18.910 --> 00:35:24.020

Pa3: you know their their heart rate and the

275

00:35:24.590 --> 00:35:36.110

Pa3: like. Their heart rate, the energy, expenditure, and like how quickly they recover. And everything was significantly more than kids without a physical disability. And I think

276

00:35:36.180 --> 00:35:44.320

Pa3: that a lot of this opens awareness as far as like expectations for kids with special with

277

00:35:44.610 --> 00:35:48.840

Pa3: special needs, and also kind of

278

00:35:50.230 --> 00:36:00.100

Pa3: reiterates that, like even though a child has a physical disability like they should still be physically active, and they should still be doing things that kind of promote

279

00:36:00.170 --> 00:36:10.050

Pa3: that general health and wellness that we do with with typically developing kids. but it kind of puts into perspective, like

280

00:36:11.360 --> 00:36:22.800

NM: what is physically exertion, what is physical exertion for them? How does it differ? And how do we kind of modify activities, so we're not over doing it or under doing it. I don't know if that makes sense, but

281

00:36:24.010 --> 00:36:25.130

NM: that's a good point.

282

00:36:27.450 --> 00:36:29.090

NM: all right. Anything else.

283

00:36:29.790 --> 00:36:33.640

Pa3: No, it was a pleasure chatting with you, and

284

00:36:34.250 --> 00:36:40.000

Pa3: sorry we were all over the place.

285

00:36:40.240 --> 00:36:44.110

NM: No need to apologize. Hold on, i'm just up the recording. Thank you so much. Hold on 1 s.
