# Supplementary material for: Validity of PROMIS® Pediatric Physical Activity Parent Proxy Short Form Scale as a Physical Activity Measure for Children with Cerebral Palsy Who Are Non-Ambulatory
Source: Behav Sci (Basel). 2025 Jul 31;15(8):1042. doi: 10.3390/bs15081042 (PMC12382615; doi:10.3390/bs15081042)
Supplement: Supplementary file 1 [file behavsci-15-01042-s001.zip › Transcripts copy/Parent transcripts de-identified/Pa2.docx]

WEBVTT

1

00:00:01.770 --> 00:00:20.280

NM: All right. Good evening. Thank you for joining me tonight, as we talk about physical activity and children with single palsy who are not full time Walkers. So I do have a couple of questions, and I have some follow up. So it it sounds like i'm scripted is because I am. Thank you for your time, and let me jump right int0 0ur survey and our interview questions.

2

00:00:20.280 --> 00:00:22.300

All right. So the first question is.

3

00:00:22.400 --> 00:00:25.960

NM: how do you define physical activity for your child.

4

00:00:29.080 --> 00:00:34.910

PA2: His physical activity umm right now is not as much as I want it to be

5

00:00:37.400 --> 00:00:39.280

PA2: due to the fact that

6

00:00:39.420 --> 00:00:45.780

PA2: he's in school most of the week, and by the time he gets home from school it's late, and then the weekend

7

00:00:45.840 --> 00:00:52.690

PA2: he's basically lazy and doesn't want to get out of bed. So it's really hard for us to get him out of bed.

8

00:00:52.780 --> 00:01:01.590

PA2: But it's been like we said we've come up with an idea that starting this year on the weekends. We're gonna get them out of the bed at least

9

00:01:01.600 --> 00:01:10.080

PA2: one day on the Saturday or Sunday, so he can get out of the bed, and because you got get him moving more than he is moving now.

10

00:01:11.180 --> 00:01:16.100

NM: Okay, great. How would you define his physical activity? What? What what do you consider physical activity for him.

11

00:01:16.310 --> 00:01:21.620

PA2: well For me physical activity for him would be getting him out of his bed

12

00:01:21.760 --> 00:01:26.680

PA2: and putting him in his chair and actually going out with him somewhere.

13

00:01:28.940 --> 00:01:41.170

PA2: That would be. That's like a physical activity, and he's not doing that much, only only the only the time that he goes to school, but like with us here at home, he's not doing much physical activity.

14

00:01:41.990 --> 00:01:44.470

PA2: and I want to. I want to start improving that.

15

00:01:46.080 --> 00:01:51.900

NM: and just to follow up when you say he does when he goes to school. Do you consider that physical activity as well?

16

00:01:52.000 --> 00:01:53.800

PA2: Yes.

17

00:01:55.250 --> 00:02:00.590

PA2: I definitely do, because he does a lot of stuff at school more stuff than he does at home.

18

00:02:04.490 --> 00:02:21.060

NM: And what would you say at school? You would consider physical activity like what type of activity?

PA2: all his therapies like when he goes to physical therapy when he goes to OT. when they take him out into the community

19

00:02:21.060 --> 00:02:28.170

Anything that you do with him that doesn't, have him in his chair or out of his chair

20

00:02:28.460 --> 00:02:29.430

NM: got it

21

00:02:33.010 --> 00:02:35.790

NM: awesome. And how do you think?

22

00:02:35.860 --> 00:02:50.990

NM: Oh, I'm: Sorry I haven't a prompt okay. First prompt. The Department of Health defines fiscal activity as any activity that encompasses energy expended and activation of skeletal muscles. Does this definition change your mind? About how you define physical activity for him? Why or why not?

23

00:02:51.370 --> 00:02:54.050

PA2: No, it doesn't Cause: yeah, I think

24

00:02:54.090 --> 00:02:57.430

PA2: you know, any any movement or anything is physical activity.

25

00:02:57.630 --> 00:02:58.840

NM: Got it.

26

00:02:58.880 --> 00:02:59.460

NM: Okay?

27

00:03:01.530 --> 00:03:05.260

NM: And how do you think physical activity differs from rest for him?

28

00:03:05.940 --> 00:03:07.370

PA2: From rest?

29

00:03:14.760 --> 00:03:15.820

PA2: I don't know.

30

00:03:19.210 --> 00:03:22.670

PA2: Well, he's pretty much resting right now.

31

00:03:23.860 --> 00:03:31.510

PA2: You know he's not really doing anything. He's just laying down and not not moving. He is just watching TV

32

00:03:31.520 --> 00:03:34.910

PA2: So that he’s resting

33

00:03:36.280 --> 00:03:38.200

NM: got it. Thank you.

34

00:03:38.580 --> 00:03:51.690

NM: What? Actually? Vide0 0kay, next question, what activities would you consider your child does as physical activity? You already gave us some. But if you can give me, let's say, 3 activities that you will consider physical activity.

35

00:03:57.370 --> 00:03:59.620

PA2: When we do arts stuff, Arts’and Crafts.

36

00:04:03.920 --> 00:04:07.040

PA2: We play this game. It's called head bangers

37

00:04:08.250 --> 00:04:16.370

PA2: that they sent from school, and that's I counted as a physical activity. and

38

00:04:19.459 --> 00:04:24.770

PA2: when we talk in his Toby, I try to have conversations back and forth for him on his toby.

39

00:04:24.800 --> 00:04:31.100

PA2: They don't they don't necessarily always have to be back and forth most of the time It's just me talking to him.

40

00:04:31.110 --> 00:04:31.980

PA2: but

41

00:04:33.730 --> 00:04:40.690

PA2: I sometimes I I feel like, if we do it more enough, he gets because I feel like he uses the toby more at school than he does on at home

42

00:04:41.180 --> 00:04:42.140

NM: Okay.

43

00:04:42.880 --> 00:04:48.510

PA2: So I want him to be able to start using it the same way uses it school him use at home.

44

00:04:52.090 --> 00:04:53.430

NM: All right. That's great.

45

00:04:54.110 --> 00:05:01.620

NM: and you didn't you weren't unsure, but my prompt is, if i'm sure let's discuss some of your child's visual activities.

46

00:05:01.740 --> 00:05:09.790

NM: and such as engaging in the use of objective equipment. would you consider the use of a standard or a gate trainer as physical activity.

47

00:05:09.950 --> 00:05:11.040

PA2: Yes.

48

00:05:11.220 --> 00:05:11.950

NM: okay.

49

00:05:12.740 --> 00:05:17.980

NM: And how about transitions in and out of a wheelchair?

PA2: Yes, definitely

50

00:05:18.070 --> 00:05:18.890

NM: Okay.

51

00:05:21.290 --> 00:05:25.650

NM: And does your child enjoy time on a playground swing.

52

00:05:26.870 --> 00:05:36.370

PA2: I he used to. I don't know. I don’t know if he can fit now, he used to love it.

53

00:05:36.510 --> 00:05:37.410

NM: Okay.

54

00:05:44.000 --> 00:05:50.900

NM: And how about his ability to use his arms during reaching or a ball toss. Would you consider that physical activity?

55

00:05:51.020 --> 00:05:52.210

PA2: Yes.

56

00:05:52.460 --> 00:05:53.250

NM: okay.

57

00:05:56.720 --> 00:06:06.750

NM: And how do you? How how do? How do related services such as physical, therapy, occupational therapy, vision. Hearing education relate to physical activity

58

00:06:08.080 --> 00:06:22.610

PA2: because he's always he's gonna be moving around. They're gonna be adjusting him. They have to prompt him or do something, so he's gonna be moving around. So any movement I consider physical activity

59

00:06:27.050 --> 00:06:33.070

NM: great. And does does your child do these activities alone, or in a group? Why or why not?

60

00:06:33.520 --> 00:06:42.930

PA2: No, he doesn't do them in the group. Yeah. Well. well, you know what I know. He doesn't. His related services is one on one, but he has the para

61

00:06:42.950 --> 00:06:44.180

PA2: that helps him

62

00:06:46.340 --> 00:06:49.420

PA2: So it's him the para and and the therapist.

63

00:06:49.630 --> 00:06:50.500

NM: Okay.

64

00:06:51.670 --> 00:06:53.820

NM: I just got it.

65

00:06:54.210 --> 00:06:55.530

NM: And next question

66

00:06:55.730 --> 00:07:01.660

NM: How many times a week. Does your child participate in these activities, and for how long?

67

00:07:03.080 --> 00:07:11.360

PA2: I believe he gets physical therapy 5 times a week for an hour.

68

00:07:11.480 --> 00:07:12.550

NM: Okay.

69

00:07:12.930 --> 00:07:15.020

PA2: OT

70

00:07:15.250 --> 00:07:17.470

PA2: I believe, is 4 times

71

00:07:17.600 --> 00:07:20.270

PA2: an hour, 4 times a week for an hour

72

00:07:23.310 --> 00:07:27.730

PA2: vision he gets, I believe it's 2 times a week

73

00:07:28.170 --> 00:07:32.910

PA2: for an hour, and he gets speech. And I think speeches for

74

00:07:33.360 --> 00:07:35.520

PA2: is 3 times a week for an hour.

75

00:07:35.790 --> 00:07:36.670

NM: Okay.

76

00:07:38.480 --> 00:07:39.870

NM: and

77

00:07:39.900 --> 00:07:57.340

NM: that's great. Do you? And some of the other activities you mentioned. Do you know how often we talked about the gait trainer, Stander and communication? How often does he do those things? In a week?

78

00:07:57.860 --> 00:08:16.790

PA2: The communication device he does use at school every day. I don't know how long. He has it all day at school, he’s not using a loaner on, he has his personal one, He also uses an ipad, because gave him one at school, he uses that and the toby.

79

00:08:18.330 --> 00:08:35.230

PA2: What was the other stuff that you said?

NM: You mentioned…I brought up the gait trainer, stander You said that considered transition in and out of the

80

00:08:39.140 --> 00:08:39.940

NM: Okay.

81

00:08:39.980 --> 00:08:47.670

PA2: I don't think they are putting him in the gait trainer. know how often, but they are standing him at school, and he also they use a They use the motomed, he pedals his legs, they use that for him also, .

82

00:08:48.330 --> 00:08:50.890

PA2: It's okay. There you go

83

00:08:51.000 --> 00:08:55.830

PA2: that he just has some petal pedal to pedal his legs

84

00:09:00.110 --> 00:09:06.010

PA2: so he's.

85

00:09:06.450 --> 00:09:09.310

NM: I think it's the motomed, if I remember what that right

86

00:09:15.050 --> 00:09:18.090

PA2: You can put it to work by itself, or you can put him to pedal.

87

00:09:18.670 --> 00:09:19.690

NM: Awesome.

88

00:09:22.180 --> 00:09:25.210

NM: Okay? And

89

00:09:25.310 --> 00:09:30.030

NM: And does he need assistance with all the things we talk about. And you mentioned arts and stuff, too.

90

00:09:30.260 --> 00:09:32.100

NM: Is that pretty regularly?

91

00:09:35.250 --> 00:09:38.660

NM: And how long can you do the arts. Our activity?

92

00:09:38.710 --> 00:09:42.310

PA2: Yes, We can probably do like, maybe like 30 min.

93

00:09:43.060 --> 00:09:44.050

NM: Okay.

94

00:09:44.470 --> 00:09:50.560

PA2: sometimes more depends on depending on the day it is. And if he's engaged, and if it's something that he really likes.

95

00:09:50.770 --> 00:09:51.720

NM: Gotcha

96

00:09:52.530 --> 00:10:04.700

NM: Hey? Okay, so does he need assistance to complete any of these activities

97

00:10:05.840 --> 00:10:08.310

PA2: for the entire thing

98

00:10:13.320 --> 00:10:17.860

NM: And do you think he should participate in more or less of these activity. Now why

99

00:10:18.320 --> 00:10:33.310

PA2: I think he should participate in more because he likes to do them. He it bring some pleasure, and he enjoys them. Why not?

100

00:10:36.080 --> 00:10:36.880

NM: Well.

101

00:10:39.620 --> 00:10:44.190

NM: all right now. We're the second part, all right. So this survey may look familiar.

102

00:10:44.470 --> 00:10:47.260

Let me share my screen.

103

00:10:48.640 --> 00:10:55.520

NM: So if You've done this every before. You're familiar with how the parent reports for the child right? And so this is

104

00:10:55.560 --> 00:10:59.910

a parent is given this survey. This was developed by the National Institute of Health.

105

00:11:00.030 --> 00:11:08.340

NM: It's a parent proxy. Physical activity Survey with a parent report how intense the activity of the child endured for the week before.

106

00:11:12.890 --> 00:11:23.570

NM: but for you to grade the quality of the question. So, for example, the first question is, how many days did your child exercise a place so hard that his or her body got tired.

107

00:11:23.610 --> 00:11:29.780

NM: I'm going to ask you as a parent how appropriate this question is for

108

00:11:29.810 --> 00:11:36.460

NM: for a parent with a child with Cp. Who is not a full time. Walker and 0 would be not appropriate.

109

00:11:36.660 --> 00:11:55.620

NM: 5 would be highly appropriate. And and you can give me

110

00:11:55.870 --> 00:12:09.290

NM: Yeah, and that's the information that's the kind of information I need from you like when things like what? For each question, grade it for me. How appropriate! And then give me the reasoning why. and you will probably have some of that reaction with some of these questions.

111

00:12:09.760 --> 00:12:11.720

So the first question.

112

00:12:14.200 --> 00:12:15.430

NM: which is.

113

00:12:15.780 --> 00:12:24.440

NM: How many days. Did your child exercise a place so hard that his or her body got tired? Would you say this is appropriate? Looking at the question. It is

114

00:12:24.510 --> 00:12:29.660

NM: Not at all. It's a 0 5 is highly appropriate, or anywhere in between. And why

115

00:12:30.240 --> 00:12:32.090

PA2: I don't think it's appropriate.

116

00:12:32.450 --> 00:12:37.120

NM: Okay. So what number would you rate it give me a reason. Why

117

00:12:37.190 --> 00:12:43.200

PA2: would it be a 0 like? Not at all. 0 t0 5 is what you

118

00:12:45.410 --> 00:12:49.260

9 0. I'm gonna i'm gonna say, maybe like here.

119

00:12:49.490 --> 00:12:51.020

NM: Okay? And why?

120

00:12:51.330 --> 00:13:03.860

PA2: Because maybe it's just not word. Maybe it's not worded correctly.

121

00:13:03.910 --> 00:13:21.810

PA2: That's none that doesn't walk. It's gonna you know, gonna be like Well, it doesn't relate to my child; but if they use, if they were in a different way, like, how, how, how many days is your child get physical therapy.

122

00:13:21.890 --> 00:13:33.470

PA2: and I that that is, you know, is that their body got tired. You know what i'm saying.

123

00:13:33.750 --> 00:13:40.070

PA2: but like I feel like if they telling me Excise, my son doesn't exercise a play to me. His exercise is physical therapy.

124

00:13:40.120 --> 00:13:43.290

NM: right.

125

00:13:43.590 --> 00:13:44.510

PA2: you know.

126

00:13:45.680 --> 00:13:47.450

NM: That is great feedback.

127

00:13:48.960 --> 00:13:56.160

NM: all right, perfect. You got you got the gist of what we're trying to do? That's exactly right. So grade it and tell me why. Okay. So number 2,

128

00:13:56.370 --> 00:14:05.870

NM: How many days your child exercise really hard for 10 min or more. How would you rate this question? 0 Not applicable? 5. It's highly appropriate, or somewhere between. And why.

129

00:14:12.320 --> 00:14:25.470

PA2: I would say the same thing. I gave to the first one. I said, I said 3 right or 2,

130

00:14:29.210 --> 00:14:31.820

PA2: and then maybe it would be okay.

131

00:14:32.070 --> 00:14:39.800

NM: So in terms of this one. Is it the exercise really hard? That's that's bothering me?

132

00:14:39.880 --> 00:14:42.400

NM: Okay. that's that's hopeful.

133

00:14:43.500 --> 00:14:44.460

NM: Thank you.

134

00:14:46.570 --> 00:14:54.310

NM: Awesome Number 3. How many days did your child exercise so much that he or she breathed hard. Brett, breathe hard.

135

00:14:56.020 --> 00:14:57.880

PA2: I'm going to give this on a 0.

136

00:14:57.920 --> 00:14:59.900

NM: Okay? And why?

137

00:15:00.090 --> 00:15:03.260

PA2: Because I it it's I

138

00:15:03.430 --> 00:15:09.860

PA2: First of all, I've never seen. No, I work out so so much that he's out of breath.

139

00:15:10.910 --> 00:15:13.930

PA2: and like you know I that's why

140

00:15:14.130 --> 00:15:18.610

NM: that's why that's that is very helpful. Thank you. Number 4.

141

00:15:18.720 --> 00:15:22.910

NM: How many days was your child so physically active? And he or she sweated.

142

00:15:25.160 --> 00:15:27.340

PA2: and that's not. That's also a 0.

143

00:15:27.350 --> 00:15:29.350

NM: Okay? And why?

144

00:15:29.380 --> 00:15:37.320

PA2: Because he, he, he, he's he doesn't work out or or physically, is physically that active to the sweat.

145

00:15:40.250 --> 00:15:41.980

PA2: So it doesn't apply to him.

146

00:15:42.150 --> 00:15:47.190

NM: That's what we want to hear from you. Thank you. Application

147

00:15:48.880 --> 00:15:55.660

NM: and Number 5. How many days did you chat, exercise to play so hard that his or her muscles burned.

148

00:15:57.050 --> 00:15:58.460

PA2: Same thing.

149

00:15:58.670 --> 00:16:00.000

NM: Okay, give me a number.

150

00:16:00.210 --> 00:16:10.500

PA2: I would say, See? But then this is this picky question, because I I mean he, his muscles could hurt and they burn. But how is he able to tell me? Because he can't verbalize?

151

00:16:11.830 --> 00:16:23.400

PA2: So you know. Hmm. I don't know

152

00:16:23.520 --> 00:16:25.500

PA2: you know this is hard.

153

00:16:25.800 --> 00:16:27.670

NM: This is yeah. This one is tough.

154

00:16:28.100 --> 00:16:33.850

NM: What number would you rate it? And it's totally. But you gave me some rash good rationale, so

155

00:16:34.200 --> 00:16:39.990

PA2: 0. Not at all. 5 highly applicable somewhere in between. I'm. Going to say 3.

156

00:16:40.230 --> 00:16:41.090

NM: Okay.

157

00:16:41.130 --> 00:16:48.720

PA2: Now I am in the middle somewhere, because I I mean, you know is is is. you know I could, you know.

158

00:16:49.110 --> 00:16:53.910

NM: So you kind of want to know. It seems like this is higher because you want to know if he does have this.

159

00:16:58.190 --> 00:17:01.910

PA2: and I think that he is able to tell me like he, he's able to

160

00:17:02.140 --> 00:17:08.369

PA2: show me, or it, or express himself, to let me know that he's in in. He's in pain. So

161

00:17:08.380 --> 00:17:09.410

PA2: so I want to see.

162

00:17:10.430 --> 00:17:17.490

NM: Okay. all right. Number 6. How many days did your child exercise a place so hard that he or she felt tired

163

00:17:24.880 --> 00:17:26.380

NM: or somewhere in between.

164

00:17:32.620 --> 00:17:34.430

I'm gonna say.

165

00:17:50.810 --> 00:17:58.280

PA2: what is one of the most important?

166

00:18:00.740 --> 00:18:02.250

PA2: I'm going to say 4,

167

00:18:02.280 --> 00:18:06.480

PA2: because there are things that he's gone to school and come back, and he's tired.

168

00:18:06.610 --> 00:18:07.470

NM: Okay.

169

00:18:08.170 --> 00:18:10.390

PA2: and I know it's because they've worked them out.

170

00:18:10.530 --> 00:18:11.440

NM: Okay.

171

00:18:14.940 --> 00:18:15.840

NM: all right

172

00:18:24.500 --> 00:18:33.620

PA2: when he has times all like there's there's days off in between, and then you go

173

00:18:33.990 --> 00:18:40.140

PA2: stuff like like this. Break that he's off on. Now when he goes back on Monday. He's gonna come back on Monday.

174

00:18:40.670 --> 00:18:41.570

PA2: Tired?

175

00:18:42.830 --> 00:18:45.840

PA2: Absolutely. Yeah.

176

00:18:46.000 --> 00:18:55.240

NM: All right. Number 7. How many days was your child physically active for 10 min or more. How would you rate this question? 0. Not a full appropriate 5 Highly.

177

00:18:55.420 --> 00:18:57.810

PA2: I will say 5. That's a

178

00:18:58.010 --> 00:18:58.830

NM: Okay.

179

00:18:58.880 --> 00:19:05.910

PA2: And why is it appropriate? Because he's in school, and he does all these physical stuff activities for more than 10 min at school.

180

00:19:06.120 --> 00:19:07.220

NM: Hmm.

181

00:19:07.710 --> 00:19:09.270

PA2: You know. So

182

00:19:10.410 --> 00:19:14.620

PA2: and we do. We do stuff here at home that takes more than 10 min.

183

00:19:15.190 --> 00:19:16.170

NM: So yeah.

184

00:19:16.950 --> 00:19:24.370

NM: so is it more s0 0f the wording or the way you can measure it like, what are the things that we make it more applicable compared to some of the other ones?

185

00:19:30.970 --> 00:19:37.790

PA2: I think because this one doesn't say exercise

186

00:19:38.300 --> 00:19:39.210

NM: got it.

187

00:19:40.700 --> 00:19:46.170

PA2: cause I'm: okay with this one. How many times you say? I tell you that I said that that one was fine, how many?

188

00:19:46.250 --> 00:19:53.600

PA2: I'm fine with that, because he is physical. but it's just that that exercise for it throws me off.

189

00:20:00.010 --> 00:20:00.820

PA2: Yeah.

190

00:20:01.100 --> 00:20:03.370

PA2: you know, we're

191

00:20:03.400 --> 00:20:04.310

PA2: you know.

192

00:20:04.410 --> 00:20:05.240

NM: Right?

193

00:20:05.430 --> 00:20:07.420

PA2: Absolutely all right.

194

00:20:10.080 --> 00:20:12.020

NM: all right. Number 8.

195

00:20:13.300 --> 00:20:19.620

NM: How many days you ch one for 10 min or more 0 not appropriate at all. 5 highly appropriate! How will you rate that one?

196

00:20:26.870 --> 00:20:34.520

NM: And as we wrap up. I'd like to get any final thoughts or comments from you about physical activity

197

00:20:34.610 --> 00:20:47.720

NM: for your son and this population anything you wanted to share about your

198

00:20:47.790 --> 00:20:50.490

PA2: these children are so forgotten, and it's so sad.

199

00:20:54.050 --> 00:20:55.550

NM: Thank you so much.

200

00:20:57.530 --> 00:20:58.580

NM: Anything else.

201

00:20:58.700 --> 00:21:00.060

PA2: No, that's it.

202

00:21:01.520 --> 00:21:04.970

NM: Thank you for your, your, your participation on the stop, the recording.
