# Supplementary material for: Validity of PROMIS® Pediatric Physical Activity Parent Proxy Short Form Scale as a Physical Activity Measure for Children with Cerebral Palsy Who Are Non-Ambulatory
Source: Behav Sci (Basel). 2025 Jul 31;15(8):1042. doi: 10.3390/bs15081042 (PMC12382615; doi:10.3390/bs15081042)
Supplement: Supplementary file 1 [file behavsci-15-01042-s001.zip › Transcripts copy/Parent transcripts de-identified/Pa15.docx]

WEBVTT

1

00:00:00.300 --> 00:00:19.060

NM: Hi! Good afternoon. Thank you so much for taking the time to meet with me today. We're going to be talking about physical activity for children who are not full time walkers at single palsy. So i'm going to ask you a few questions. The first half we're going to ask you questions about how you view physical activity. And then the second half I'm. Going to show you a survey that was developed by National Institute of Health

2

00:00:19.060 --> 00:00:35.730

NM: Specific. It was developed for children that were actually of regressing, as it relates, to cancer. So, just looking at a survey that was created for children that were not typically developing and looking how that applies to children with Cp. We are not full time walkers, and we'll do that for the second half. Are you ready to proceed.

3

00:00:35.820 --> 00:00:36.490

PA15: Yes.

4

00:00:36.600 --> 00:00:41.190

PA15: okay. First question is, how do you define physical activity for your child?

5

00:00:44.390 --> 00:00:50.830

PA15: That's when we're physically moving him into a gait trainer. his bicycle

6

00:00:51.070 --> 00:00:53.360

PA15: his stander.

7

00:00:54.290 --> 00:00:59.860

PA15: and even when he's on the mat you can roll over, sit up. But he needs help.

8

00:00:59.970 --> 00:01:01.020

PA15: but that's it.

9

00:01:01.130 --> 00:01:03.530

PA15: As long as he's not on the couch or in the wheelchair.

10

00:01:04.120 --> 00:01:07.680

PA15: That to me is physical activity.

11

00:01:08.120 --> 00:01:23.920

NM: The first prompt is the department of Help defines physical activity as any activity that encompasses energy extended, and activation of skeletal muscles. Does this change your? Does this definition change your mind about how you define physical activity for your child? Why or why not?

12

00:01:25.560 --> 00:01:37.880

PA15: No, I think i'd agree with that definition. Because he is moving. He is using his energy, it just. He probably uses more energy than a typical kid just by standing. but He's engaged. He he's working.

13

00:01:39.370 --> 00:01:44.540

NM: you know that's great. How do you think physical activity differs from rest?

14

00:01:46.770 --> 00:02:00.330

PA15: So rest is, you know, just a different ambience. Different different atmosphere. It's, you know, laying down in this room or on the couch being comforted, cud-, cuddling favorite toys.

15

00:02:00.580 --> 00:02:01.440

PA15: and

16

00:02:01.510 --> 00:02:06.130

PA15: there he's like at peace, you know, not being not being bothered

17

00:02:06.760 --> 00:02:07.660

NM: Ok

18

00:02:08.720 --> 00:02:10.680

NM: Perfect Okay. Second question.

19

00:02:10.720 --> 00:02:19.750

NM: Now, I mean, i'm sorry. What activities would you consider your child does is physical activity. You already gave me some. So you mentioned the gait trainer

20

00:02:20.360 --> 00:02:22.080

NM: and stander, or anything else?

21

00:02:22.560 --> 00:02:30.010

PA15: when the weather permits the bicycle, and then we have a hoyer lift in the house like it it.

22

00:02:30.140 --> 00:02:37.830

PA15: and they set it up so that they gave me a special harness, so he can stand in the hoyer lift in front of the TV.

23

00:02:38.040 --> 00:02:46.800

PA15: But even in the mat, like sitting up on the mat with a wedge behind him or him reaching for the toys like.

24

00:02:47.360 --> 00:02:52.770

PA15: I consider any movement that he does, because if you leave him on the couch, you don't engage he'll stay there.

25

00:02:52.780 --> 00:02:53.940

NM: Got you.

26

00:02:56.590 --> 00:03:15.200

NM: awesome, all right. And so one prompt is, if you're not sure what you gave me a great amount of examples. Let's discuss some of your child's habitual activities such as engaging in the use of activity as adaptive equipment. You already said, You consider stander and gait trainers as physical activity. How about transitions in and out of the wheelchair.

27

00:03:16.160 --> 00:03:20.350

PA15: It's physical activity for me.

28

00:03:20.380 --> 00:03:21.670

PA15: No, I don't that

29

00:03:22.320 --> 00:03:27.800

PA15: I don't consider that physical activity for him because he doesn't help any more

30

00:03:27.900 --> 00:03:30.350

PA15: when he was a little stronger.

31

00:03:30.470 --> 00:03:31.470

PA15: he would

32

00:03:31.540 --> 00:03:36.440

PA15: weight bear, and kind of try to set up a little bit all that's gone.

33

00:03:36.760 --> 00:03:37.640

PA15: So.

34

00:03:37.750 --> 00:03:42.340

PA15: transferring from the wheelchair to the bed or a couch, or Mat.

35

00:03:42.370 --> 00:03:43.720

PA15: that's all on me.

36

00:03:45.320 --> 00:03:56.030

NM: Got it. And how about you mentioned? Oh. how about a playground swing? Does he? Do you consider that physical activity. Does he like the adaptive swings? Does he still, you know, enjoy those?

37

00:03:56.800 --> 00:04:04.840

PA15: He does enjoy them. And I think for him. Yeah, because he has to work on keeping his head straight, you know, like

38

00:04:05.860 --> 00:04:08.760

PA15: he's not just laying there. you know. So

39

00:04:09.500 --> 00:04:16.200

PA15: maybe like light physical activity for him. Let's move his legs when he's excited. So yeah, he's moving.

40

00:04:16.470 --> 00:04:18.019

NM: Got it.

41

00:04:18.459 --> 00:04:19.380

NM: got it.

42

00:04:21.110 --> 00:04:26.450

NM: and reaching you mentioned reaching what kind of reaching or ball toss, would you. You consider that physical activity?

43

00:04:26.480 --> 00:04:31.660

PA15: Yes. definitely. we call it Geronimo

44

00:04:31.720 --> 00:04:35.200

PA15: I don't know if he's really playing around, because I don't know if he really

45

00:04:35.370 --> 00:04:46.470

PA15: it seems like it, though you throw it. You say Geronimo. He grabs it, and he throws it back. It's not a full throw, but he's. He throws it back on command when you say Geronimo

46

00:04:46.540 --> 00:04:56.600

PA15: for Easter. He got this cute little football. but it lights up, and he likes the material. It's easy for him to grab, so he's been throwing that.

47

00:04:57.650 --> 00:04:58.890

NM: That's awesome.

48

00:04:59.200 --> 00:05:01.700

NM: That's awesome. And

49

00:05:01.910 --> 00:05:11.840

NM: all right. So how do you? How does related services such as physical, therapy, occupational therapy vision hearing and even speech relate to his physical activity? When you say

50

00:05:12.890 --> 00:05:16.880

PA15: well definitely ot and pt, they always have him on the mat, or

51

00:05:17.070 --> 00:05:25.700

PA15: they'll they'll get him outside on the gait trainer. They do the gait trainer inside the house before they leave. They'll help me put him in the stander.

52

00:05:28.100 --> 00:05:35.950

PA15: They'll even like I said do the bike rides. and then, I guess with speech, there's physical activity, because most of the time he sits in a bean bag.

53

00:05:36.230 --> 00:05:41.610

PA15: and so you'll see him trying to sit up and reach for the button.

54

00:05:41.860 --> 00:05:42.960

PA15: Okay.

55

00:05:44.010 --> 00:05:47.510

PA15: vision, we don't get vision at home anymore. So

56

00:05:47.520 --> 00:05:54.120

PA15: what they do at school, I'm not there. But I would assume that she does have him like reaching.

57

00:05:58.200 --> 00:06:04.260

NM: Okay, great. And does your child do these activities alone, or in a group by or why not

58

00:06:08.120 --> 00:06:09.790

PA15: like. If you leave them on the mat.

59

00:06:09.850 --> 00:06:12.170

PA15: he will. Yeah, he'll play alone.

60

00:06:12.300 --> 00:06:13.210

NM: Yeah, okay.

61

00:06:13.220 --> 00:06:17.300

PA15: His way. He’ll play alone, and

62

00:06:17.500 --> 00:06:21.790

PA15: you know he’ll roll over to try to reach a toy or try to sit up.

63

00:06:23.890 --> 00:06:32.630

PA15: and then he please with ‘brother’ I mean him and ‘brother’ are constantly wrestling and grace and jumps all over, and he tries to

64

00:06:32.640 --> 00:06:33.960

PA15: grabbed him. So

65

00:06:34.120 --> 00:06:35.690

NM: okay, that's awesome

66

00:06:35.840 --> 00:06:40.320

PA15: with the other kids.

67

00:06:40.330 --> 00:06:43.080

PA15: He plays by himself, and then he plays with kids.

68

00:06:45.730 --> 00:06:52.510

NM: and then in the therapies he's with the therapist at all times it does he do group like therapy sessions, too.

69

00:06:54.370 --> 00:07:04.270

PA15: The only group therapy session, I think, would be when they do the what do they call it when they. when they do like Pt In the classroom.

70

00:07:04.400 --> 00:07:08.400

PA15: the push-in, I think it's called.

71

00:07:08.650 --> 00:07:13.730

PA15: and group other than that. It's like in the physical therapy room. There's other kids around, but

72

00:07:14.080 --> 00:07:18.200

PA15: she's focused on him, and he's supposed to be focused on her.

73

00:07:18.260 --> 00:07:19.670

NM: Got it Got it That's perfect.

74

00:07:20.280 --> 00:07:26.640

NM: all right. Your question. How many times a week does your child participate in these activities, and for how long?

75

00:07:28.110 --> 00:07:30.620

PA15: daily. Okay.

76

00:07:32.050 --> 00:07:35.960

PA15: it really depends on his mood.

77

00:07:36.550 --> 00:07:41.470

PA15: P,: Ot Sessions are half an hour at the house, but, like I said.

78

00:07:41.910 --> 00:07:56.600

PA15: let's say like Pt. Would like. Have him on the mat rolling and sitting, and I know that OTs coming. She'll come out for, and I know old he's coming like at 5 or 5 30 she'll help me put him in the stander, and he'll hang out in the stander

79

00:07:57.400 --> 00:08:12.080

PA15: while I cook. Yeah, so it's. Sometimes it's in spurts of half an hour, but sometimes it stretches out. and we try to keep him engaged in doing something until bath time.

80

00:08:12.470 --> 00:08:13.380

NM: Okay.

81

00:08:14.300 --> 00:08:20.950

PA15: So like maybe what? 3 from like 3, 30 to 6, 30, he's doing. He's doing some type of

82

00:08:20.970 --> 00:08:23.320

NM: Everyday? That's a lot, that's good.

83

00:08:23.540 --> 00:08:26.260

PA15: Yeah, he gets off the bus at 3, 10

84

00:08:26.270 --> 00:08:36.860

PA15: and 3 30. Yeah. You got speech right after speech. It's Ot. And then that's when he has a break, pt. That he has a little bit of a break type of change

85

00:08:37.840 --> 00:08:42.520

PA15: snack, and then Pt. Or he's in the stander waiting for Pt to come

86

00:08:43.210 --> 00:08:46.260

NM: that's awesome. He's busy

87

00:08:46.320 --> 00:08:47.330

NM: all right.

88

00:08:47.430 --> 00:08:52.410

NM: and then, do you do they need assistance to these activities

89

00:08:52.540 --> 00:08:56.750

NM: to get them in and out? I would, you know. Yes, he would need the assistance right? And then

90

00:08:57.080 --> 00:08:59.320

NM: you explain for me. Go ahead, please.

91

00:08:59.530 --> 00:09:03.400

PA15: So he's 49 inches tall. which isn't that tall for his age

92

00:09:03.630 --> 00:09:04.980

NM: or is it

93

00:09:05.000 --> 00:09:11.290

PA15: but he's 70. He's 79 pounds, and he barely helps

NM: got it.

94

00:09:12.840 --> 00:09:17.830

PA15: He can help with the upper body, but the lower body he can't help at all. So, like

95

00:09:19.020 --> 00:09:24.670

PA15: my husband, “husband”. Could pick him up and move him by himself. But me.

96

00:09:25.180 --> 00:09:27.690

PA15: I could do it if I had to, but

97

00:09:28.040 --> 00:09:30.940

PA15: I prefer help. and then

98

00:09:31.000 --> 00:09:36.750

PA15: Pt. Or the therapist they're just they won't do it by themselves. He's just too heavy. They're they need

99

00:09:36.780 --> 00:09:38.260

PA15: to man transfer.

100

00:09:38.370 --> 00:09:39.390

NM: Okay.

101

00:09:41.340 --> 00:09:42.240

NM: Got it.

102

00:09:44.600 --> 00:09:49.400

NM: And do you think he should participate in more or less of these activities, and why?

103

00:09:49.980 --> 00:09:51.800

PA15: I wish I could do more

104

00:09:51.880 --> 00:09:52.790

PA15: like I.

105

00:09:52.930 --> 00:10:03.100

PA15: Yeah, there's a lot of stuff around my neighborhood like there's he did it one year the baseball and there swimming, and there's horseback riding and

106

00:10:03.350 --> 00:10:10.320

PA15: I just don't have the bandwidth to add that into a schedule after school, because he's so busy with therapy.

107

00:10:10.910 --> 00:10:17.250

PA15: and he goes to school year around. So yeah. I wish he could do more.

108

00:10:17.550 --> 00:10:22.480

PA15: I wish I could take him to do more because he does what he does in his way. But

109

00:10:22.800 --> 00:10:24.540

PA15: I just wish I could take them to do more.

110

00:10:25.400 --> 00:10:26.400

NM: Gotcha.

111

00:10:26.870 --> 00:10:36.680

NM: Well, you're doing a great job. Okay, Thank you. You got him doing a lot that's awesome. All right. We're on the on to the second part. I'm gonna show you the survey. I'll pull it up so you can see it

112

00:10:43.590 --> 00:10:50.570

NM: sure. All right. So this is called the promise parent proxy, physical activity, or I put it in Spanish, you know

113

00:10:50.890 --> 00:10:59.030

NM: I mean, do you want it to Spanish or how you put it? They'll take me for I was gonna say, listen, I I have it so that I

114

00:10:59.540 --> 00:11:01.360

NM: so that I can help

115

00:11:01.410 --> 00:11:10.080

NM: translate. I speak the very minimal, but I can get through, and I do have a translator to in case. Yeah. Now, we're ready.

116

00:11:13.750 --> 00:11:19.730

NM: See that? Okay, make it look Well, that's a small that. Yeah, that's too small

117

00:11:19.760 --> 00:11:20.820

NM: as well. Okay.

118

00:11:20.950 --> 00:11:22.240

PA15: this is.

119

00:11:22.940 --> 00:11:35.080

NM: Can I? I like to zoom in. I can I can. I can scroll. I can do one at a time, because the truth is, you don't really need the the the the day's portion. So Just so. You know about this the survey. So

120

00:11:35.120 --> 00:11:42.730

NM: the service given to a parent they answer for the week before they're they. They're rating the week before they get the survey.

121

00:11:42.730 --> 00:12:12.540

NM: So if the survey was given on a Friday, we'd be right in the week before I miss about how many days the child did so and so, and it's about how intense the child is engaging in physical activity. And so again, this was traded, not for children with Cp. We're looking to see if this can be for this population. And I really need like your honest opinion about each question. So i'm gonna ask you to rate it like 0, not appropriate at all. I would not want to be asked this whatever, and give me a reason up to a score 5 that's appropriate. I could see myself answering that, and I can give a good answer about it

122

00:12:12.540 --> 00:12:29.080

NM: like it relates: okay. So we'll through these first One is how many days your child exercise a place so hard that his or her body got tired. How would you rate that question, and why 0 not would be not appropriate at all up to a 5 highly appropriate.

123

00:12:31.390 --> 00:12:35.040

PA15: maybe probably like a 2 or 3.

124

00:12:35.320 --> 00:12:38.050

NM: You guys give me one. That is

125

00:12:41.650 --> 00:12:47.900

PA15: some, maybe a 3. I Probably if I had this question, and I had to read it, I would probably get annoyed

126

00:12:47.910 --> 00:12:50.160

PA15: because. hey lur

127

00:12:51.750 --> 00:12:54.320

PA15: circumstance, he doesn't speak

128

00:12:54.540 --> 00:12:55.740

NM: so.

129

00:12:55.970 --> 00:13:06.060

PA15: I wouldn't know how tired he actually is! It's not like gracing. He's in those lacrosse to call Mom, You know my legs hurt mom my shoulder.

130

00:13:06.190 --> 00:13:12.880

PA15: Toby can't tell me that so? The the question might be. That's why i'm giving it like a 3. But then

131

00:13:13.390 --> 00:13:14.760

PA15: I could kind of like

132

00:13:16.430 --> 00:13:18.790

PA15: guess by.

133

00:13:19.450 --> 00:13:21.710

PA15: and

134

00:13:21.880 --> 00:13:23.310

PA15: if you didn't wake up.

135

00:13:23.400 --> 00:13:25.010

PA15: you know.

136

00:13:25.330 --> 00:13:32.260

PA15: if he was whining before 70'clock, then I would say, okay, he's tired because he did all this, so I could guess

137

00:13:33.170 --> 00:13:33.990

PA15: you get it.

138

00:13:34.320 --> 00:13:38.240

NM: Yeah, I get it. You know. That's actually really good feedback

139

00:13:38.390 --> 00:13:39.340

NM: about that.

140

00:13:39.370 --> 00:13:42.440

NM: Okay, All right, Number 2. You doing great. That's exactly what I want.

141

00:13:42.580 --> 00:13:47.530

All right. The second question is, how many days your child exercise really hard

142

00:13:47.630 --> 00:13:51.310

NM: for 10 min or more. How would you write this question, and why?

143

00:13:51.540 --> 00:13:53.030

PA15: Oh, one.

144

00:13:53.110 --> 00:13:54.030

NM: Okay.

145

00:13:56.400 --> 00:14:00.280

PA15: I get. Maybe i'm just sensitive, but everything's really hard for him. You know

146

00:14:00.390 --> 00:14:04.490

NM: that the words underlined really hard, like

147

00:14:04.790 --> 00:14:08.230

PA15: everything. It's. It's a question for him.

148

00:14:11.420 --> 00:14:13.740

NM: That's that's helpful.

149

00:14:15.450 --> 00:14:26.630

NM: all right. What would be a better term, you think, if it like, related to this kind of Congress? This question about exercising phone and quote really hard. What would you feel? Maybe a suggestion that could be replaced?

150

00:14:32.620 --> 00:14:36.180

PA15: I don't know. like.

151

00:14:40.250 --> 00:14:49.980

PA15: and Don't worry. I was just curious, maybe like like not the word strain, but something like that word like Not that he strained himself working, but that, like

152

00:14:51.750 --> 00:14:53.470

PA15: I don't know. I'm sorry.

153

00:14:53.560 --> 00:14:55.440

Oh, that's fine. No, that's fine.

154

00:14:55.480 --> 00:15:03.420

NM: That's what I I do appreciate you all right. Number 3. How many days your job ex so much that he or she breathe hard.

155

00:15:06.480 --> 00:15:11.210

PA15: It's just not appropriate. I for it. It would be a 0 for me, because I don't. I think.

156

00:15:11.520 --> 00:15:12.180

PA15: that

157

00:15:13.430 --> 00:15:17.040

PA15: he's limited into what he can do, so he's not gonna like, you know.

158

00:15:17.100 --> 00:15:27.780

PA15: dug around the block and come back and try to right. You know I don't think that the physical activity that he can do would cause that kind of reaction.

159

00:15:28.050 --> 00:15:28.770

Right?

160

00:15:31.450 --> 00:15:33.040

NM: That's good. Okay.

161

00:15:35.690 --> 00:15:37.460

NM: All right. Next one

162

00:15:38.280 --> 00:15:43.890

NM: Number 4. How many days was your child so active that he or she sweated.

163

00:15:46.330 --> 00:15:48.840

NM: And how appropriate then, would that be.

164

00:15:52.510 --> 00:15:54.430

PA15: I guess, maybe like a

165

00:15:55.560 --> 00:15:56.680

PA15: a 3.

166

00:15:57.670 --> 00:15:59.370

Okay. And why?

167

00:16:01.040 --> 00:16:07.480

PA15: Just to my situation, the only time he really swept his when he's doing the physical activity outside.

168

00:16:07.860 --> 00:16:08.750

PA15: Okay.

169

00:16:09.300 --> 00:16:13.310

PA15: I do know that that's not everybody's situation, you know. Kids do sweat. So

170

00:16:15.610 --> 00:16:23.020

PA15: So he does. What, though, like, you know, some kids don't sweat at all. Well, I've seen that many times. I don't know those sweat when he's outside.

171

00:16:23.220 --> 00:16:24.560

NM: Okay, okay.

172

00:16:25.170 --> 00:16:29.510

PA15: me or grammar won't let him like we start seeing that he starts listening

173

00:16:29.630 --> 00:16:35.300

PA15: that rag, and we cause then, if he gets too hot. gets red, it gets really red

174

00:16:36.940 --> 00:16:39.070

PA15: and overheating, you have a seizure

175

00:16:40.680 --> 00:16:42.000

NM: that's important to know.

176

00:16:42.340 --> 00:16:43.380

PA15: Yes.

177

00:16:46.400 --> 00:16:49.650

NM: that's important, and all. Thank you all right. No, i'm fine.

178

00:16:49.740 --> 00:16:55.160

NM: How many days in child exercise, or play so hard that his or her muscles are

179

00:16:56.040 --> 00:17:01.080

PA15: again. I would probably rate that just because of my situation, a one I wouldn't know

180

00:17:01.150 --> 00:17:03.980

PA15: what kind of you know muscles he's burning like.

181

00:17:04.579 --> 00:17:06.700

PA15: I don't know. I think, that even him

182

00:17:07.700 --> 00:17:10.619

PA15: just sitting up burns muscles. You know

183

00:17:10.630 --> 00:17:14.000

PA15: everything is

184

00:17:14.630 --> 00:17:16.510

PA15: much harder for him.

185

00:17:16.880 --> 00:17:25.180

PA15: but I wouldn't know. Oh, I played, and then not you, compared to Grace and Grace, and tell you. Oh, this hurts, that hurts like

186

00:17:25.290 --> 00:17:29.590

PA15: I wouldn't be able to answer that question for Jo B. I wouldn't be able to guess it

187

00:17:30.380 --> 00:17:40.220

PA15: right, and it's mostly mostly due for the number of these like you mentioned before, because he's not verbal. He can't communicate. I could guess when he's tired, you know.

188

00:17:40.400 --> 00:17:42.370

NM: Yeah, but there's even harder.

189

00:17:42.700 --> 00:17:46.880

PA15: I can't. If he's, you know, if he burned muscle or he's. So

190

00:17:48.550 --> 00:17:49.410

NM: yeah.

191

00:17:52.040 --> 00:17:58.150

NM: okay. And number 6. How many days is your child exercise a place so hard that he or she felt tired.

192

00:17:59.520 --> 00:18:01.320

PA15: I could I

193

00:18:01.510 --> 00:18:02.810

PA15: it for

194

00:18:02.960 --> 00:18:08.950

PA15: because I guess what? Oh, wow, I just say, wow, he's really tired. I'll get to.

195

00:18:09.270 --> 00:18:11.090

NM: So okay, this is a better one.

196

00:18:12.290 --> 00:18:13.140

NM: Okay.

197

00:18:14.090 --> 00:18:18.870

NM: All right. Now, what's up? How many days was your child physically active? For 10 min or more

198

00:18:20.440 --> 00:18:25.800

PA15: I could answer that with no problem. 4 5 5, 5. Okay. This was appropriate. And why?

199

00:18:26.630 --> 00:18:32.860

PA15: Just because I could easily answer that without having to rely on information from him, you know, like

200

00:18:32.980 --> 00:18:42.010

PA15: I I can monitor the the time intervals, and I'm not relying on information from him to question

201

00:18:42.400 --> 00:18:43.280

NM: Gotcha

202

00:18:44.380 --> 00:18:48.750

NM: all right, and the last one. How many days the child run for 10 min or more

203

00:18:49.550 --> 00:18:55.740

PA15: 0. It can't run. So i'd probably be a little fault you reading that.

204

00:18:57.040 --> 00:18:57.970

NM: Yeah.

205

00:19:00.890 --> 00:19:14.280

NM: all right. And then, as we wrap up Cynthia, thank you. I would love to give you a chance to give me your final thoughts about physical activity, as it relates to your child, and any thing you want to share, as it relates to this this topic.

206

00:19:14.810 --> 00:19:21.620

PA15: Well, I I think our whole family. especially my husband, pushes all of us to be

207

00:19:21.650 --> 00:19:26.500

PA15: a little bit more physically active, active.

208

00:19:27.340 --> 00:19:30.890

PA15: just for like the healthy lifestyle, and you know.

209

00:19:30.900 --> 00:19:35.200

PA15: And then with Joby, it's very important, because I feel like, if

210

00:19:35.650 --> 00:19:45.120

PA15: you don't use it, you're going to lose it, you know. So the little bit that he does do I've noticed, like, if we're in the hospital a week, and we're stuck in the bed, or we can't.

211

00:19:45.290 --> 00:19:55.460

PA15: There's there's a little bit of a question. I mean. Come back home and you're starting back from. So it to us is really important to keep Joey like engaged and doing something, and

212

00:19:56.070 --> 00:20:05.160

PA15: plus it's it's nice for him to we understand there, and his brother is playing music and twirling him around. And that's the way that he also.

213

00:20:05.310 --> 00:20:14.840

PA15: by keeping him physical, not in the bed, not in his room apart from the family. It keeps him with us, you know, and like we're all together.

214

00:20:15.380 --> 00:20:18.480

NM: Yeah, if that makes any sense. But

215

00:20:18.800 --> 00:20:20.530

NM: that makes perfect sense.

216

00:20:22.460 --> 00:20:26.280

NM: That's awesome. Thank you, Cynthia. I will stop the recording.

217

00:20:26.320 --> 00:20:27.740

PA15: Okay, you're very welcome.
