# Supplementary material for: Validity of PROMIS® Pediatric Physical Activity Parent Proxy Short Form Scale as a Physical Activity Measure for Children with Cerebral Palsy Who Are Non-Ambulatory
Source: Behav Sci (Basel). 2025 Jul 31;15(8):1042. doi: 10.3390/bs15081042 (PMC12382615; doi:10.3390/bs15081042)
Supplement: Supplementary file 1 [file behavsci-15-01042-s001.zip › Transcripts copy/Parent transcripts de-identified/Pa1.docx]

WEBVTT

1

00:00:00.790 --> 00:00:06.810

NM: All right. Good evening. Thank you so much for joining us today. We're going to be

2

00:00:07.010 --> 00:00:19.510

talking about physical activity specifically for children with several palsy who are not full time walkers. And so in the Cp. Classification scale. That's classification 4 5 0ut of the gross motor classification, functional scale.

3

00:00:19.510 --> 00:00:27.540

And so i'm going to ask you a few questions, feel free to answer as you see fit. There's no right or wrong Answer. We are so grateful for your feedback, and I do have a couple of prompts.

4

00:00:27.760 --> 00:00:37.460

NM: as it relates to each question. And so, if I sound script, it is because I am, and then we'll end well. In the second half of this interview, with viewing of a

5

00:00:37.730 --> 00:00:48.230

NM: a physical activity intensity scale that was created by the National Institute of health called the promise scale, and i'll show that i'll pull that up on the screen, and i'll ask you some questions in relates to that. Are you ready to start

6

00:00:48.380 --> 00:00:49.260

PA1: ready.

7

00:00:49.340 --> 00:00:55.330

NM: Okay, Great. So my first question is, how do you define physical activity for your child?

8

00:00:56.190 --> 00:00:58.420

PA1: Oh, my

9

00:00:58.500 --> 00:01:09.570

PA1: one of his biggest physical activities is the act of communication. So him interacting with his communication device can create

10

00:01:09.680 --> 00:01:14.290

PA1: a lot of activity for him to be able to manage his body.

11

00:01:18.130 --> 00:01:34.620

NM: Great. Thank you. My first follow up is the department of Health defines physical activity as any activity that encompasses energy extended, and activation of skeletal muscle. Does this definition. Change your mind about how you define physical activity for your child. Why or why not?

12

00:01:34.740 --> 00:01:37.490

PA1: No, it doesn't because

13

00:01:37.520 --> 00:01:54.290

PA1: that's that act of communicating he's having to engage his muscles, and you know, and really be active to just interact with his device. So that simple thing, you know.

14

00:01:54.720 --> 00:02:00.930

PA1: And how do you think physical activity differs from rest.

15

00:02:02.380 --> 00:02:10.389

PA1: so rest to me, for for him is when his body is calm.

16

00:02:10.620 --> 00:02:13.000

PA1: and that's not

17

00:02:13.160 --> 00:02:16.510

PA1: super often for him throughout the day.

18

00:02:16.600 --> 00:02:21.390

It may be, you know generally, if he's just.

19

00:02:21.700 --> 00:02:36.210

PA1: you know, the rest of like. The closest thing I would say is like, when a person is watching TV. You just kind of zone out, and then you can see everything be calm But if he's engaged in learning or thinking, or communicating, or anything.

20

00:02:36.600 --> 00:02:38.170

PA1: he is not at rest.

21

00:02:41.770 --> 00:02:43.050

NM: Thank you.

22

00:02:43.440 --> 00:02:57.390

NM: Next question what activities? And you, You. You gave us some examples already, but you could elaborate or continue with this the same examples. What activities would you consider your child does as physical activity

23

00:02:59.100 --> 00:03:06.280

PA1: so well. So when he's engaged with his communicator when he's playing a board game

24

00:03:06.790 --> 00:03:09.460

PA1: when he's playing Dj.

25

00:03:09.520 --> 00:03:11.180

PA1: With his sister.

26

00:03:11.370 --> 00:03:18.450

you know he also does activities outside of his chair like he likes to be out in a different position, and he'll kick.

27

00:03:18.490 --> 00:03:25.290

PA1: and he'll lay on the bed and kick his legs dance the way that he does.

28

00:03:25.480 --> 00:03:33.540

PA1: Let's see, he uses a gait trainer, you know. That's a big activity, but you know, and which is more typical.

29

00:03:33.970 --> 00:03:45.720

PA1: But, like I said, even you know, to for him to play, you know, engage with his sister and play, play, play a board game that gets his muscles moving and engaged and his heart rate up

30

00:03:46.920 --> 00:03:48.570

PA1: so that's activity to me.

31

00:03:50.490 --> 00:03:52.340

NM: That's great, Thank you

32

00:03:52.350 --> 00:04:02.620

NM: and what you were not unsure. But if unsure, let's discuss some of your child's habitual activity, such as engaging in the use of the adaptive equipment.

33

00:04:02.700 --> 00:04:06.440

You've mentioned gait trainers. So would you consider the use of a stander

34

00:04:06.450 --> 00:04:08.910

NM: or a gait trainer as physical activity?

35

00:04:09.390 --> 00:04:17.579

PA1: 0 0, yeah, yeah, Even a stander. The amount of effort he has to put in to be in a

36

00:04:17.930 --> 00:04:22.640

standing position, even though he's well supported.

37

00:04:22.920 --> 00:04:35.450

PA1: What isn't and that again. That's active for him to keep his trunk up, his head up us his arms if he can, to help make that support physical activity.

38

00:04:37.430 --> 00:04:43.670

NM: Thank you. Does your child enjoy time on a playground swing?

39

00:04:45.280 --> 00:04:49.260

PA1: Does he enjoy it, boy? That's

40

00:04:49.340 --> 00:04:55.120

PA1: some, if we ever take him. Sometimes he does.

41

00:04:56.700 --> 00:05:06.790

PA1: but it's not there Aren't. Many swings that he can be comfortable and feel safe in or that I can get him in an out of

42

00:05:10.000 --> 00:05:17.800

PA1: So a playground swing not often does it happen we don’t have that many opportunities

43

00:05:17.820 --> 00:05:21.890

PA1: a therapy swing in a therapy session, that feeling. Yes, he likes that and this is fine.

44

00:05:24.250 --> 00:05:37.280

NM: And how do you relate? How does related services such as physical, therapy, occupational therapy vision, hearing, or hearing education relate to physical activity for your child.

45

00:05:38.230 --> 00:05:41.940

PA1: So no no vision or hearing therapies but

46

00:05:42.630 --> 00:05:54.610

PA1: pt and ot. He has to have that baseline in order to have the stamina and strength to engage in an academic program to engage in his

47

00:05:54.620 --> 00:06:04.080

PA1: assistive technology program, because if he doesn't have the that core, that baseline of Pt. Of being able to.

48

00:06:04.290 --> 00:06:06.250

PA1: you know, uses his core.

49

00:06:06.390 --> 00:06:19.170

PA1: manage his dystonia. He's not going to be able to engage with the communication device or drive his power chair. or, you know, follow along with his academics.

50

00:06:22.280 --> 00:06:29.280

PA1: So they're They're very interrelated. But I take Pt. And ot to be the the center of the hub.

51

00:06:29.650 --> 00:06:30.370

NM: Okay.

52

00:06:31.420 --> 00:06:40.010

NM: thank you. And does your child do the activity that you mentioned alone or in a group? And why or why not?

53

00:06:40.710 --> 00:06:58.510

PA1: So he does a lot of one on one therapies. He has some group to help with his social, but mostly it's one on one, because that's his. His learning profile is better with that direct instruction for the the repetition, so that he's not

54

00:06:58.510 --> 00:07:04.660

PA1: confused by wrong answers from other students. He also has a one on one para that

55

00:07:04.690 --> 00:07:09.610

PA1: is engaged in, all of you know, along with the other therapies and and academics.

56

00:07:09.960 --> 00:07:10.930

NM: Gotcha.

57

00:07:14.480 --> 00:07:15.710

NM: And

58

00:07:15.880 --> 00:07:32.790

NM: how many times a week does your child participate in in these activities. So you, i'll. I'll pick some just to kind of help us hone in. Let's talk about the communication you mentioned the gait trainers, and you mentioned board games. How how many times a week would you say your child participates in these activities?

59

00:07:32.840 --> 00:07:43.320

PA1: So board games? I'm gonna say you know we do it over the weekend, and then maybe a couple of times. You know it is down time at school. S0 3 to 4 times

60

00:07:44.830 --> 00:07:50.110

PA1: gait trainer 2 to 3 times.

61

00:07:51.050 --> 00:08:05.880

PA1: and the social you said no communication piece communication communication, you know. That's all day every day. He has speech sessions every day at school

62

00:08:05.930 --> 00:08:15.990

PA1: but you know, being able to communicate at home. We were just at the Zoo, right. He used a a manual board.

63

00:08:16.330 --> 00:08:18.100

PA1: He interacted with me.

64

00:08:18.240 --> 00:08:24.550

PA1: you know. He engages with the people in the store

65

00:08:24.560 --> 00:08:33.070

PA1: to, you know, kind of flirt his way into into a discount. That's communication.

66

00:08:34.159 --> 00:08:38.320

NM: That's his own communication.

67

00:08:38.770 --> 00:08:54.190

NM: And does he need assistance typically for completing these activities. and if there are some that if you want to qualify with these, does he use, when does he need assistance, and how much assistance does he need to do some of these activities we talked about.

68

00:08:54.790 --> 00:09:01.300

PA1: So I mean, he's he needs assistance with everything.

69

00:09:01.950 --> 00:09:05.530

PA1: Oh, yeah, you can. You know he's so. He's a 12 year old

70

00:09:05.560 --> 00:09:10.240

PA1: boy, so he's not going to go out on his own

71

00:09:10.350 --> 00:09:23.420

PA1: in New York City. He can drive on his own, but I would not let him drive unsupervised, just like I wouldn’t let him ride his bike unsupervised, he can certainly

72

00:09:23.500 --> 00:09:26.650

PA1: navigate his communication device

73

00:09:27.150 --> 00:09:31.290

PA1: and be able to express himself with others independently.

74

00:09:32.620 --> 00:09:38.100

PA1: So so there's that. But to to get like into the gait trainer, right? He's not going to go 0ff on his own.

75

00:09:38.190 --> 00:09:38.900

NM: Okay.

76

00:09:44.300 --> 00:09:47.860

NM: And for the communication he's independent once it's all set up?

77

00:09:48.190 --> 00:09:57.210

PA1: Yes,

NM: how much setup is required for him with the eye gaze? Specifically, actually, it is, but

PA1: it's just mounting the device and turning it on.

78

00:09:57.410 --> 00:09:58.660

PA1: and from there

79

00:09:58.780 --> 00:10:00.620

PA1: he's on his own. Yeah.

80

00:10:02.460 --> 00:10:16.310

NM: And you mentioned a manual board. How much assistance does that require with the manual board of this communication device?

PA1: that just needs somebody to hold it, and

81

00:10:16.390 --> 00:10:19.460

PA1: He knows how to spell, and it's just a way for him to spell everything out.

82

00:10:22.250 --> 00:10:23.020

NM: Okay.

83

00:10:23.240 --> 00:10:28.070

PA1: but he requests it like he, and he knows. you know, when we're out, like

84

00:10:28.340 --> 00:10:32.410

PA1: if I, if he goes like this, and a big eye up that's like.

85

00:10:32.920 --> 00:10:37.530

PA1: Get behind me. Pull out the device so I can tell you what I need.

86

00:10:37.800 --> 00:10:41.820

NM: Great! He cues you to let you know when He wants to talk. That's great.

87

00:10:42.400 --> 00:10:47.250

Do you think he should participate in more or less of these activities? And why

88

00:10:48.090 --> 00:10:51.670

PA1: so more is always great.

89

00:10:51.740 --> 00:10:58.300

PA1: Right?

90

00:10:58.710 --> 00:11:06.520

PA1: Yeah. So the more opportunity that he has to use his device in different settings, which is what I would say would be the thing that

91

00:11:06.960 --> 00:11:23.370

PA1: maybe it's where we have a challenge, right because he's in school. And then to use it a communication device, an electronic device that's bulky and expensive, and needs to be plugged in, and only has can only work when you're using wi-fi.

92

00:11:24.770 --> 00:11:28.940

PA1: Yeah, I didn't carry it to the zoo I don't.

93

00:11:29.230 --> 00:11:34.800

PA1: And so then he's restricted from his very easy, very expressive

94

00:11:37.500 --> 00:11:38.540

PA1: avenue.

95

00:11:41.260 --> 00:11:42.460

NM: That's all good.

96

00:11:42.580 --> 00:11:48.440

NM: Okay, Great. So now we're at the second portion of our chat. I'm gonna share my screen.

97

00:11:49.470 --> 00:11:52.050

NM: So this is called the Promise

98

00:11:54.040 --> 00:12:05.120

NM: parent Parent Proxy. Physical activity will only make sure I can make it so you can see the whole thing, so i'll leave it up on the screen while we we talk. But

99

00:12:05.120 --> 00:12:15.820

NM: as we continue on for the second portion of our interview, i'm going to ask you questions for each of the 8 questions here. So this is a physical activity survey. So as the parent, you will report for your trial.

100

00:12:15.870 --> 00:12:24.640

NM: and you were gonna you would base your reporting based on the past the prior 7 days. But what I would like you to really think about is for each question.

101

00:12:25.480 --> 00:12:40.470

NM: how appropriate is the question to addressing physical activity intensity in a child with Cp. Who is not a full time, Walker. So specific to children like your your son, and give me a rating 0. It is not appropriate at all.

102

00:12:40.920 --> 00:12:52.620

NM: 5, up t0 5, highly appropriate, can give you a good understanding of where your chat is performing day to day. Okay, or over over the past week. Okay. So for the first question.

103

00:12:53.090 --> 00:12:58.900

NM: and i'll read, I'll i'll, I'll give you Qing, for each each time I ask a question. So the first question is.

104

00:12:59.060 --> 00:13:15.680

NM: How many days did your child exercise or place so hard that his or her body got tired. How would you rate this question and determine the level of physical activity, intensity for your child? 0 not related at all up t0 5 highly appropriate. How would you rate it? And why?

105

00:13:16.400 --> 00:13:25.110

PA1: So let let me ask right? Because I I get very. I get real specific with with words, and I think exercise or play.

106

00:13:25.240 --> 00:13:33.700

PA1: It's like, okay, you know. Now, i'm thinking about his sister. and she does an exercise bed, or she goes out she runs around the playground.

107

00:13:34.480 --> 00:13:38.600

PA1: He's not engaging in that activity. but

108

00:13:38.840 --> 00:13:41.840

PA1: he is engaging his body

109

00:13:42.760 --> 00:13:47.390

PA1: to the same kind of extreme in a different

110

00:13:47.930 --> 00:13:51.420

PA1: in something I wouldn't call exercise or play, I would say.

111

00:13:51.630 --> 00:13:56.360

PA1: when he's participating in his physical therapy when he's when he's

112

00:13:56.550 --> 00:14:03.170

PA1: trying to. You know, make his computer communicator, play the dies for his board game.

113

00:14:04.910 --> 00:14:11.250

PA1: You know he.

114

00:14:11.270 --> 00:14:15.430

PA1: How many does he work so hard that his body gets tired every day.

115

00:14:15.520 --> 00:14:16.390

NM: Got it

116

00:14:17.380 --> 00:14:22.750

NM: So do you? If it was the term work you would really get. How would you raise that effort?

117

00:14:22.910 --> 00:14:28.510

PA1: I would say

118

00:14:29.660 --> 00:14:36.770

PA1: right, how many days this is this? Does he expend him so much effort that his body gets tired every day?

119

00:14:37.060 --> 00:14:39.870

NM: Hmm, that's good. So

120

00:14:39.880 --> 00:14:45.830

NM: on his face. So this is where it gets tricky right? Because we can edit s0 0n a space. How is Ren?

121

00:14:45.990 --> 00:15:00.810

NM: And you already give me some rationale? Why, you would rate it on his face, looking at this question phrase Just how was raised? How appropriate would you think this will be for parents or children that are not full Time Walkers 0 you can do, and you can give me a range, and that would be the highest to the lowest.

122

00:15:03.800 --> 00:15:15.360

PA1: because I would probably put Pt. In there. You know that I would say as exercise. Then I would say it's applicable. It's kind of a of an applicable question. Then it's like a 5.

123

00:15:15.930 --> 00:15:35.830

NM: Okay, so you would get one day 10. It's like sort of applicable. Okay. So somewhere between. So Zeros No, not it. Accurate at all. 5 is highly appropriate like this will be golden. Good good question to go to. So somewhere in between is where you're thinking

124

00:15:35.830 --> 00:15:37.640

PA1: i'll just say it's a 5,

125

00:15:37.990 --> 00:15:45.940

NM: s0 5 is the highest. Oh, i'm sorry. That's a good 10. So not here. Okay. N0 0 is too low. Then i'm going to g0 0n the 2

126

00:15:45.970 --> 00:15:50.300

PA1: 2 0kay.

127

00:15:50.370 --> 00:15:53.280

NM: exercise. The term exercise is

128

00:15:53.320 --> 00:16:02.620

NM: really not as applicable, and with the true sense of the words, it's kind of what you said. I just want to make sure any other reasons why you think it. It's kind of middle of the road.

129

00:16:04.560 --> 00:16:09.430

PA1: That's really that that exercise or play. Okay, idea.

130

00:16:09.670 --> 00:16:18.350

NM: Got it all right. Perfect. Thank you. All right. Number 2. How many days it your child exercise really hard for 10 min of warm

131

00:16:19.740 --> 00:16:26.120

NM: 0. Not it applicable up t0 5, highly appropriate to ask a parent of a child.

132

00:16:26.250 --> 00:16:26.940

NM: Let's see

133

00:16:27.770 --> 00:16:29.100

PA1: that's not walking.

134

00:16:29.350 --> 00:16:46.980

PA1: Yeah, i'm sorry, because i'm looking at the like the days in trying and getting confused, I think. Oh, yes, that's the problem. Yeah, yeah, it's it being thinking of like I'm not even worried about how many days does he do? It is this likeable question? It's just the question, and and that's a really good feedback for me, because I usually leave it up.

135

00:16:47.010 --> 00:17:00.870

NM: But you're my first parent. So I and I think because it was a parent survey.

136

00:17:01.170 --> 00:17:09.980

NM: So don't worry. It's not about rating the question it's I mean rating the days on how you would measure your child, but in terms of a therapist with someone asking you this.

137

00:17:10.710 --> 00:17:14.890

NM: How appropriate would you feel? This question is from you to answer. You know what i'm saying.

138

00:17:14.970 --> 00:17:18.170

PA1: N0 0ne your child knowing his level of activity.

139

00:17:18.200 --> 00:17:29.210

PA1: right? S0 0kay. So i'm gonna forget. I'm gonna forget the days. And

140

00:17:29.280 --> 00:17:33.540

PA1: Okay, I need an exercise like clear exercises. Yes.

141

00:17:33.910 --> 00:17:37.920

PA1: Does he have Pt: and does he?

142

00:17:38.230 --> 00:17:43.230

PA1: Does he do a guess, maybe an exercise program like, okay?

143

00:17:43.320 --> 00:17:47.120

PA1: If I, if I thought of it as like, you know, working out with a trainer

144

00:17:48.500 --> 00:17:53.300

PA1: exercise, then Pt. Counts and ot counts

145

00:17:53.520 --> 00:17:58.770

PA1: right, and and then it feels more applicable.

146

00:18:12.450 --> 00:18:18.660

PA1: and and if I read it blank like that, then i'm like no that doesn't apply to that's a 0

147

00:18:18.960 --> 00:18:19.880

NM: right

148

00:18:19.910 --> 00:18:22.960

PA1: if I change it in my head a little bit. Then.

149

00:18:24.000 --> 00:18:29.440

PA1: if I have to. If I change it, then it becomes applicable. I like adapt. I adapt the question.

150

00:18:30.030 --> 00:18:34.320

NM: Okay. So we need to be adapted, but that on his on his face.

151

00:18:44.250 --> 00:18:51.390

PA1: But I as a as an interviewer, we would have to ask the same questions.

152

00:18:51.640 --> 00:18:55.210

PA1: What would you say, how relatable, applicable, would you say?

153

00:18:55.400 --> 00:18:59.920

PA1: Would this be from this?

154

00:19:00.050 --> 00:19:02.140

PA1: Okay, I like it. You know

155

00:19:02.390 --> 00:19:06.080

PA1: it. Doesn't terminology like we did. Okay, All right. That's great.

156

00:19:06.170 --> 00:19:17.990

NM: Thank you. That's really that's really helpful in terms of feedback. Number 3. How many days is your child exercise so much that he or she breathes hard 0 not applicable at all

157

00:19:18.070 --> 00:19:21.530

NM: up t0 5 highly appropriate. How would you rank this question?

158

00:19:22.060 --> 00:19:23.610

PA1: I'm going back t0 2.

159

00:19:23.670 --> 00:19:25.510

NM: Okay. And why

160

00:19:25.630 --> 00:19:27.980

PA1: with the it's that word. Exercise

161

00:19:30.910 --> 00:19:33.530

NM: it all right. Number 4.

162

00:19:34.190 --> 00:19:38.220

PA1: How many days was your child so physically active that he or she sweated?

163

00:19:38.430 --> 00:19:43.370

PA1: That's a good question. That

164

00:19:43.470 --> 00:19:47.360

NM: Okay? Why? Because it's not telling me. It's not. It's not

165

00:19:47.690 --> 00:19:49.550

PA1: boxing the

166

00:19:49.600 --> 00:19:53.320

PA1: activity. It's not

167

00:19:54.410 --> 00:19:55.320

PA1: it. Got it

168

00:19:56.310 --> 00:19:59.750

PA1: like you said. I think he's physically active sitting in his chair.

169

00:20:06.480 --> 00:20:20.000

NM: That's great. all right. And number 5. How many days did your child exercise our play so hard that his or her muscles are 0 not applicable at all up t0 5 highly appropriate.

170

00:20:20.170 --> 00:20:21.130

NM: And why

171

00:20:21.450 --> 00:20:26.710

I'll, i'm going back down to the 2, and I might change all these 2 t0 0ne.

172

00:20:27.040 --> 00:20:27.830

PA1: Okay.

173

00:20:27.860 --> 00:20:34.380

NM: you like, you know, wherever it says exercise. I'm like

174

00:20:34.680 --> 00:20:35.930

PA1: or play.

175

00:20:38.860 --> 00:20:49.660

PA1: Now, here we are, 5 questions in, and am I going to flip Flop? Does he plan.

176

00:20:52.680 --> 00:20:55.850

NM: But I think the key part of this one is the muscles.

177

00:21:01.420 --> 00:21:09.490

PA1: If they do.

178

00:21:10.050 --> 00:21:13.910

NM: Okay, that yeah. And the terminology. And I I think you're making it really good.

179

00:21:14.060 --> 00:21:15.440

PA1: Yeah.

180

00:21:15.740 --> 00:21:17.200

How many days?

181

00:21:17.510 --> 00:21:22.490

NM: Say it again. You know where I'm coming from?

182

00:21:22.540 --> 00:21:27.960

NM: Number 6. How many days your chat exercise a play so hard that he or she felt tired.

183

00:21:29.740 --> 00:21:31.500

2

184

00:21:31.650 --> 00:21:32.400

NM: Okay.

185

00:21:35.940 --> 00:21:45.560

NM: And number 7. How many days was your child physically active for 10 min or more? That one I can give a 5 for the

186

00:21:45.590 --> 00:21:46.970

PA1: applicability

187

00:21:47.040 --> 00:21:48.630

NM: because it's applicable.

188

00:21:48.760 --> 00:21:49.700

PA1: Yeah.

189

00:21:55.160 --> 00:22:01.820

PA1: how many days did your child run for 10 min or more? I'm. Okay, All right, I I know, and and bye

190

00:22:03.370 --> 00:22:06.830

PA1: 0 because they don't run. Okay

191

00:22:07.270 --> 00:22:12.790

NM: and and and this and I will tell you a little bit more about the this. This scale was.

192

00:22:13.120 --> 00:22:26.580

NM: I believe, initially developed for children that we're. We're losing, functioning as a release oncology and leukemia. So it was. It wasn't created for children that are, let's say, typically developing, however, just trying to find something

193

00:22:26.910 --> 00:22:37.730

NM: is where we're trying to go. And I really appreciate everything you said. So the last thing I like to do is always open up for final comments and thoughts, or anything. Joules you want to give us as we wrap up.

194

00:22:37.830 --> 00:22:44.180

NM: and it relates to physical activity in this population for your son, and what you would like to share from your experience.

195

00:22:44.340 --> 00:22:56.660

PA1: Sure, sure, I think you know, like we've I've filled out. I don't know how many surveys from it, and I don't think that there's one that's made for a kid

196

00:22:56.890 --> 00:23:00.270

PA1: like him. That's fully

197

00:23:00.660 --> 00:23:08.000

PA1: wheelchair dependent fully dependent on others for all aspects of his daily living activities.

198

00:23:08.010 --> 00:23:14.500

PA1: and yet super engaged mentally in his environment with his friends.

199

00:23:14.510 --> 00:23:18.810

PA1: So it's a challenge, because I know they're written from

200

00:23:18.930 --> 00:23:26.740

PA1: at whatever time they were written, for whatever purpose. But whenever you have a and not this survey at all, but there are ones that are like.

201

00:23:26.970 --> 00:23:28.880

PA1: you know.

202

00:23:28.930 --> 00:23:31.330

PA1: Do you worry about him running into the street?

203

00:23:32.250 --> 00:23:41.410

PA1: No, I've already answered that he uses it. He's not running anywhere right? I mean, that's just reality.

204

00:23:41.950 --> 00:23:50.930

PA1: mentally and behaviorally competent to know that he needs to look before he crosses the street.

205

00:23:51.840 --> 00:23:59.000

PA1: Yes.

206

00:23:59.370 --> 00:24:02.900

PA1: behavior and physical activity. But they don't fight, get it right.

207

00:24:07.830 --> 00:24:11.260

PA1: do it because it I'm asked.

208

00:24:11.270 --> 00:24:20.110

PA1: But I always just kind of expect that whoever's working with him knows that some of that's applicable, and some of it's not.

209

00:24:21.990 --> 00:24:29.100

PA1: It would be nice to have a a survey that's more beneficial for a population like him

210

00:24:29.230 --> 00:24:35.500

PA1: that tells a little more. Because I again i'm in a and maybe i'm a little too picky on the words.

211

00:24:35.540 --> 00:24:36.880

When I think

212

00:24:36.950 --> 00:24:41.330

PA1: exercise, I always kind of figure that everything's kind of coming from that baseline of

213

00:24:41.610 --> 00:24:49.570

PA1: typically developing.

214

00:24:50.470 --> 00:24:56.440

PA1: sits in his chair, but can move in his chair and can move his chair, but also

215

00:24:56.550 --> 00:24:59.200

PA1: has to manage

216

00:24:59.500 --> 00:25:06.890

PA1: dystonia and tone, and the exertion that that creates

217

00:25:07.890 --> 00:25:09.120

PA1: is immense.

218

00:25:11.370 --> 00:25:14.260

PA1: So and that's very helpful. Yeah.

219

00:25:14.870 --> 00:25:17.140

NM: And the

220

00:25:17.880 --> 00:25:21.010

PA1: thank you so much. We're gonna stop the recording by.
