# Supplementary material for: Validity of PROMIS® Pediatric Physical Activity Parent Proxy Short Form Scale as a Physical Activity Measure for Children with Cerebral Palsy Who Are Non-Ambulatory
Source: Behav Sci (Basel). 2025 Jul 31;15(8):1042. doi: 10.3390/bs15081042 (PMC12382615; doi:10.3390/bs15081042)
Supplement: Supplementary file 1 [file behavsci-15-01042-s001.zip › Transcripts copy/Parent transcripts de-identified/Pa7.docx]

WEBVTT

1

00:00:01.260 --> 00:00:18.090

NM: All right. Good afternoon. Thank you so much for joining us today. We are going to talk about physical activity for children with Cp. Who are non ambulatory. There's no right or wrong answer, and I I thank you for your time on the first half. We're going to ask you some questions, and then i'm going to

2

00:00:18.090 --> 00:00:37.830

NM: give you some. Follow up to each question the second half. I'm going to share a survey that that was developed by the National Institute of Health that looked at physical activity, intensity for children and parent proxy, physical activity survey. So we'll do that the second half. So first half we'll start with our questions. Are you ready to?

3

00:00:38.600 --> 00:00:43.670

NM: So the first question is, how do you define physical activity for your child?

4

00:00:44.960 --> 00:00:47.670

Pa7: How do I define it.

5

00:00:47.960 --> 00:00:52.420

Pa7: I mean physical activities, anything my son can actually do.

6

00:00:52.780 --> 00:01:11.630

Pa7: and it has changed over the years, because now it's it's been reduced to. You know he's a little bit contracted and stuff so it's been reduced to like his arm movements not so much legs before it was everything. He scooted around the bed and stuff, and move his neck around, and all of that. But over time it has changed a little bit.

7

00:01:11.730 --> 00:01:14.300

Pa7: and recently, you know it's been more

8

00:01:14.420 --> 00:01:31.070

Pa7: of him just looking to where the sounds are, and then moving his arms to whatever like what. When he's going to answer questions and things like that. But he's not moving as much as he used to. So that definition has changed a little bit.

9

00:01:32.420 --> 00:01:49.820

NM: Thank you, and the first follow up is the Department of health defines physical activity as any activity that encompasses energy expended, and activation of skeletal muscles. Does this definition change your mind about how you define physical activity for your child, why or why not?

10

00:01:50.210 --> 00:01:57.880

Pa7: No, it doesn't really change it. I think it it's. It's basically the same thing. It says, I'm, basically narrowing it down to what he can do.

11

00:02:01.270 --> 00:02:06.030

NM: And how would you say physical activity differs from rest in general?

12

00:02:06.880 --> 00:02:15.280

Pa7: Well, physical activity, I mean you're moving. You're using your energy. And when you're resting your

13

00:02:16.040 --> 00:02:18.470

Pa7: not using our energy.

14

00:02:19.250 --> 00:02:20.050

NM: Yeah.

15

00:02:20.340 --> 00:02:22.600

NM: awesome. Thank you.

16

00:02:22.740 --> 00:02:25.690

NM: The next question is, what activities

17

00:02:25.720 --> 00:02:33.400

NM: would you consider your child does as physical activity? So you gave some examples. Can you maybe share like in the functional capacity?

18

00:02:33.480 --> 00:02:35.310

Pa7: Sure

19

00:02:35.340 --> 00:02:48.800

Pa7: so, ‘child’, you know. Obviously I can transfer him to his chair. When he's in his chair he is able to move his upper body a little bit more than he does when he is laying down so like his neck movements are more

20

00:02:49.330 --> 00:03:07.000

Pa7: more, I guess, voluntary, as opposed to when he's laying down, because he can't really move as much, and then his hands move a lot more, I think, when he's excited they move a lot more when he is being asked questions. They move a lot more.

21

00:03:07.020 --> 00:03:14.410

Pa7: but when he's laying down. I guess. Gravity, you know it plays a part in his strength, and like being able to lift his arms all the way and stuff like that.

22

00:03:15.710 --> 00:03:16.330

Pa7: Yeah.

23

00:03:17.530 --> 00:03:30.940

NM:: And you mentioned when he gets excited, is it? Is it? In response to the questions like you said when he's communicating?

Pa7: yeah, when he's communicating, or Sometimes he just feels that way. Sometimes he could just see him. He's laughing. And you don't even know why he's just

24

00:03:31.090 --> 00:03:34.870

Pa7: having his own moments, so he'll respond to that with his body.

25

00:03:35.160 --> 00:03:36.010

NM: Okay.

26

00:03:37.760 --> 00:03:47.670

NM: And I know he's a big boy now. So I was gonna talk about some of his habitual activities, such as use of any adaptive equipment? Is he using the adaptive equipment right now?

27

00:03:47.860 --> 00:04:01.520

Pa7: So, besides the the wheel chair, like he's he hasn't been in school in a few months. So like when it comes to like what the stander and like gait training the good, the he hasn't been using any of that he's been at home.

28

00:04:01.520 --> 00:04:09.480

Pa7: So I think the most he has been doing is like range of motion. And then switching into the chair, going outside things like that.

29

00:04:09.570 --> 00:04:10.420

NM: Okay.

30

00:04:13.030 --> 00:04:23.870

[Pa7: let those audio on 50, and I mean he's ]-background noise]

NM: like I said he's a large boy now, so would you say he, going in and out of the wheelchair with that count as physical activity for him?

31

00:04:26.830 --> 00:04:32.870

Pa7: I I guess I think its more Physical activity for me

32

00:04:37.410 --> 00:04:49.940

NM: How about the the

NM: he's too large for the playground swings right like I mean adapitve swings?

Pa7: It depends. Yeah, Some parks have the wheelchair I mean, some parks have the the wheelchair where you can put the actual wheelchair on it.

33

00:04:50.110 --> 00:04:54.630

NM: Okay? Oh, really, that's awesome. Yeah. So does he enjoy that?

34

00:04:54.790 --> 00:05:05.900

Pa7: He does? He really does. It makes him like. I feel like he has like this exhilarating feeling like when he's he's going up and down because it's not something he's used to. I don't do it often. I don't like have

35

00:05:06.060 --> 00:05:16.780

Pa7: that like freedom to do it a lot. So when it does happen, he's like, whoo, you know his eyes open and he gets excited. Yeah, he I know he feels that rush in his belly.

36

00:05:16.890 --> 00:05:21.640

NM: So you got to tell me more about this. So it's a platform that you can put the wheelchair on.

Pa7: Yeah.

37

00:05:21.990 --> 00:05:22.970

NM: wow!

38

00:05:24.240 --> 00:05:26.580

NM: And then you the how do you push it

39

00:05:27.380 --> 00:05:38.660

Pa7: Um so?It has like a handle like you know, like the the baby swings you. You just hold the actual swing. It's the same way. But you the wheelchair’s just on it.

40

00:05:38.770 --> 00:05:40.370

NM: Wow, yeah. And would you consider that physical activity for him?

41

00:05:41.410 --> 00:05:45.920

Pa7: Yes.

42

00:05:47.860 --> 00:05:50.660

NM: and he smiles

Pa7: Yeah, he loves it.

43

00:05:52.310 --> 00:06:08.310

NM: And then the last one was reaching. Does he do any he does reaching with his hands? Is he reaching to for a switch? What is he reaching for when he does his recount?

Pa7: So he is not so much reaching, like, I think, when he uses like, like the devices to communicate. He he does more like

44

00:06:09.280 --> 00:06:15.510

Pa7: like we it because it's hand over him right. So we bring it up to him, and like at a safe distance, where we know he can

45

00:06:15.540 --> 00:06:24.360

Pa7: voluntarily touch it. But he doesn't actually reach for things. Yeah, like he uses. He moves his arms a lot like, you know, like

46

00:06:24.930 --> 00:06:30.100

Pa7: back and forth, you know, but not to grab anything.

47

00:06:30.160 --> 00:06:37.160

Pa7: It's more of a like a communication thing for him.

NM: And then would you consider that as physical Activity?

Pa7: Yes.

48

00:06:37.330 --> 00:06:37.960

NM: okay.

49

00:06:40.650 --> 00:06:42.470

NM: What kind of switch is he using now?

50

00:06:42.720 --> 00:06:47.900

Pa7: The jelly bean, He's still using the jelly bean, but he also now he's using the Toby.

51

00:06:48.140 --> 00:07:02.130

Pa7: So it's more more eye movement which I feel like it's it's a little strenuous activity for him like for us, it is super easy to just scan anything but for him. It's something that he's being trained to do so. I feel like that. That is, that's

52

00:07:02.260 --> 00:07:04.310

Pa7: heavy for him. So.

53

00:07:04.560 --> 00:07:06.870

NM: wow! So how long has he been using the eye gaze?

54

00:07:08.340 --> 00:07:10.300

Pa7: for since, like 2,016

55

00:07:10.420 --> 00:07:30.060

Pa7: or 17, I want to say. But he had a break in between because we were like having like a little bit confused with his medical diagnosis, with how much he can see. But now that he we can see, we know that he understands what's on the the screen. So yeah, it's pretty pretty cool. He's very good at it.

56

00:07:32.320 --> 00:07:35.800

NM: So you consider that physical activity, right?

Pa7: Yes, I do. Greatly.

57

00:07:35.980 --> 00:07:36.800

NM: Yeah.

58

00:07:40.390 --> 00:07:55.700

NM: that's wonderful. I didn't know you want to find it. It's great.

Pa7: Yeah, it's awesome. That is awesome.

NM: How do related services such as physical therapy, occupational therapy vision, hearing, or even speech related to physical activity for your child?

59

00:07:56.060 --> 00:08:04.610

Pa7: I mean they're the ones who help him enable most of his movements. I think everything that he is capable of doing. He got from getting these therapies.

60

00:08:04.640 --> 00:08:22.780

Pa7: which right now i'm trying to get therapies at home, because I feel like this is one of the most important things is like, if it for my son, I can just imagine him, you know, wanting to do things because I mean he's seen them and other people, you know he's been able to do more before.

61

00:08:22.780 --> 00:08:29.220

Pa7: so he has to miss it. So those services are super important for physical activity.

62

00:08:39.490 --> 00:08:44.870

NM: You said he has to. He hasn't been in therapy for, like he hasn't been able to do as much what it is.

63

00:08:45.190 --> 00:08:52.500

Pa7: Okay, so he used to, because he used to take some steps with the gait trainer he used to set up.

64

00:08:52.560 --> 00:08:55.320

Pa7: and like a bunch for, like.

65

00:08:55.410 --> 00:09:00.800

Pa7: I think he reached 60 s without assistance. You know he was

66

00:09:01.070 --> 00:09:08.780

Pa7: making more head movements. He was supporting his neck a little more, but you know, because he doesn't have that consistent physical therapy that he was getting

67

00:09:08.860 --> 00:09:17.400

Pa7: an occupational therapy. He he's a little. He's very different. He's more contracted. We do a range of motion, but it's just not enough, you know.

68

00:09:21.890 --> 00:09:28.640

NM: Thank you for sharing. And does he do do any of these activities alone or in a group?

69

00:09:28.900 --> 00:09:30.740

Pa7: He does them alone

70

00:09:32.290 --> 00:09:33.730

NM: arm movements.

71

00:09:33.890 --> 00:09:34.730

Pa7: Yes.

72

00:09:34.970 --> 00:09:36.760

NM: okay. In the chair

73

00:09:37.110 --> 00:09:39.960

Pa7: in the chair and laying down

74

00:09:40.130 --> 00:09:43.170

NM: Now, the other things we talked about. He's usually with a therapist

75

00:09:43.610 --> 00:09:52.630

NM: to get into the equipment?

Pa7: Yes that was in school, he was usually with a therapist, the Stander in school and the gait trainer.

76

00:10:01.510 --> 00:10:04.680

NM: And all right. Next question.

77

00:10:05.300 --> 00:10:19.720

NM: How many times a week does your child? And this is okay, if it's not in school, how many times a week does your child participate in the activities we talked about, and for how long? So it's getting at his his level of endurance like you've mentioned the eye gaze is he doing that at home, and how long?

78

00:10:19.720 --> 00:10:46.190

Pa7: So he, I just actually got it, because they they couldn’t find it But it's here now, and he uses it mostly every day, if I like it get a chance to put it on before I go to school and stuff it's on all day, but mostly when he's on the chair, so I tell the nurse, while I'm gone, to put him on the chair every couple of hours, and keep him in for at least an hour the most 2 h, but obviously give him relief in between.

79

00:10:46.190 --> 00:11:04.940

Pa7: But he loves being his chair, obviously, I mean who you know, Going from the bed to the chair, but then also he's wearing his his AFO's. He's wearing his hands splints at the same time. So that stays on

NM: oh really he's still wearing his braces, hand braces And AFOS

80

00:11:04.940 --> 00:11:20.770

Pa7: Yeah, okay, okay, yeah. the AFOs He can deal with them all day. The hands splints are a little more tricky, because you know it's he's super contracted in his hands so he can tolerate 2 h, 2 h.

81

00:11:21.400 --> 00:11:22.050

NM: Okay.

82

00:11:23.360 --> 00:11:35.260

NM: that's great. And then so the nurse puts on the braces? and then does he let you know when he to come out of them, or when he's tired on the eyegaze?

83

00:11:35.260 --> 00:11:57.150

Pa7: No, I think. Oh, well, he does like he makes these grimacing faces, or he'll aspirate more like, you know. ‘child’ is a mouth breather, and so he he he creates the secretions when he's uncomfortable. It's like that's this way. It's like. If if you notice that you're suctioning him more often, it's like all right, what's wrong? Let's start looking like at what's bothering you. Yeah.

84

00:11:58.980 --> 00:12:00.230

NM: that's important.

85

00:12:04.780 --> 00:12:05.850

Pa7: And then

86

00:12:06.330 --> 00:12:12.380

NM: and then do you think he should participate in more or less of these activities more and more and more and more.

87

00:12:12.770 --> 00:12:13.340

Pa7: More, more, more. Yeah.

88

00:12:13.450 --> 00:12:14.300

NM: And why

89

00:12:14.590 --> 00:12:37.240

Pa7: a a lot is affected by the lack of movement. You know he's so always super constipated, and stuff like there's no like actual movement in his body. I know that when I don't move for a certain period of time, I'm. In pain when I have to move, so I know that range of motion might be a little difficult for him sometimes to, and I feel that if he had more of it. He would be looser. He would be much happier. He would

90

00:12:37.270 --> 00:12:41.860

Pa7: move more, you know, and I think that you would have a better like health.

91

00:12:43.440 --> 00:12:54.290

Pa7: because, you know, we also have to go through a transition with like his nutrition, because when he has like growth spurts like, I don't really notice, because he’s kind of the same size and stuff.

92

00:12:54.290 --> 00:13:12.320

Pa7: But then things change, and it's like, okay? Well, you're you're kind of skinny what's happening, you know. But I feel like, you know, with movement. You know your bones grow, your muscles grow, you know, and it's like something that you kinda expect to happen, but because it's not, you know. ‘child’ fluctuates, like his weight, fluctuates a little bit.

93

00:13:12.320 --> 00:13:17.630

Pa7: and I think that it was much more steady when he was in school.

94

00:13:27.990 --> 00:13:32.400

NM: And then, All right, i'm going to show you the survey.

95

00:13:36.260 --> 00:13:55.990

NM: all right. So now i'm gonna ask you to look at these questions. And again this was created for children that were not typically developing. Actually, they were on the was created for our children that were starting to lose function due to cancer. And so the parents would report on their physical activity, and i'm looking to see how a pla app up

96

00:13:55.990 --> 00:14:00.550

NM: applicable if I get the word out, this is to children.

97

00:14:00.660 --> 00:14:07.940

NM: So parents with children that have Cp. That are not ambulatory. And so i'm going to ask you how appropriate on 0 is not appropriate at all

98

00:14:08.040 --> 00:14:24.100

NM: doesn't apply to our population, or to your to your child, and then up to 5, so it can write it from 0 to 5 5. Is it totally is relevant, and it can be. It will be a good question to ask, and and I want you to tell me why, whatever score you give it. Why, you gave it that score.

99

00:14:24.170 --> 00:14:26.810

Pa7: Okay, all right. So the first question is.

100

00:14:27.200 --> 00:14:33.410

NM: how many days did your child exercise or play so hard that his or her body got tired. How would you rank this question?

101

00:14:33.880 --> 00:14:46.370

Pa7: I think it's a fair question. I would give it a 4, I guess a 4, because, like he doesn't really what, when it comes to my child specifically he can't really do a lot.

102

00:14:46.370 --> 00:14:55.380

Pa7: However, he's been in situations where he does, and he does get tired, you know, he starts falling asleep on people and stuff. So yeah, it's pretty relevant.

103

00:15:00.680 --> 00:15:05.520

NM: Okay, Number 2. Thank you. How many days

104

00:15:05.920 --> 00:15:11.810

NM: did your child exercise really hard for 10 min or more. 0. Not appropriate at all. 5 highly appropriate.

105

00:15:11.980 --> 00:15:28.940

Pa7: I think it's highly appropriate, I think, when you have difficulty with like physical therapy and stuff like that. That that's it's I consider that really hard for my child, and it can be difficult, especially when you're you're already like limited in movement, and now you're getting therapy to move

106

00:15:28.940 --> 00:15:32.190

NM: It's it's a hard transition, so I I consider that very relevant.

107

00:15:32.610 --> 00:15:41.800

Pa7: And what's the number. You give it No, mom Sorry.

108

00:15:44.720 --> 00:15:50.320

Pa7: And number 3. How many days did your child exercise so much that he or she breathed hard?

109

00:15:55.520 --> 00:15:58.600

Pa7: I it's relevant to. I think

110

00:15:59.170 --> 00:16:05.550

Pa7: it. I'd give it a 5. I've seen ‘child’ actually like breathe out like

111

00:16:05.760 --> 00:16:11.380

when he doesn't want to do any more like he'll get excited for therapy. And then midway, he's like, All right. I'm done.

112

00:16:12.450 --> 00:16:14.060

Pa7: Okay.

113

00:16:18.530 --> 00:16:24.960

NM: all right. Number 4. How many days was your child so busy, active that he or she sweated.

114

00:16:26.670 --> 00:16:48.460

Pa7: I think that that's that's also fair, I mean. But then I don't know if i'm being biased because ‘child’ is a good sweater. Yeah, that's what it's just by being so. It it is highly possible, obviously, with your extent extenuating your abilities. It's going to make you. I think it's also wrong, and i'll give it a for

115

00:16:48.670 --> 00:16:49.480

NM: okay

116

00:16:51.600 --> 00:17:07.900

Pa7: now. But he sweats sometimes when he's not active. Exactly. That's what i'm saying he sweats just by being so. It's like when it comes to actually doing something. I'm: Pretty sure. He's flooding more because he's now at using his body. Yeah, okay, yeah.

117

00:17:12.510 --> 00:17:21.119

NM: okay. that's good Number 5. How many days did your chat exercise replace so hard that his or her muscle?

118

00:17:22.400 --> 00:17:34.720

Pa7: I'll give it a 3, I think, because it it really depends on your child's level of communication where you can. Actually, they can actually share. My muscles are solar.

119

00:17:34.740 --> 00:17:41.050

or whatever. There is no way for me to know ‘child’'s muscles are so. He doesn't express pain or things like that.

120

00:17:41.270 --> 00:17:48.020

Pa7: So for me is is for me. Specifically it it. It is relevant, but not as much in my situation.

121

00:17:48.140 --> 00:17:49.780

NM: Okay, okay.

122

00:17:49.880 --> 00:17:56.690

NM: What's that? Number 6? How many days your child exercise the play so hard that he or she felt time.

123

00:17:57.090 --> 00:17:58.100

Pa7: Yeah.

124

00:17:58.600 --> 00:18:05.330

Pa7: that's fair to. I mean it goes along with the the beginning questions, I think

125

00:18:05.520 --> 00:18:18.130

Pa7: ‘child’ has developed this thing where he knows when it's physical therapy. He's either in it or not. He's like, all right. Maybe I have 10 min for you. I don't know, but he does get very, very, very sleepy after Pt: yeah. Yeah.

126

00:18:18.540 --> 00:18:21.660

Pa7: So how would you write this one?

127

00:18:21.830 --> 00:18:22.560

NM: Okay.

128

00:18:28.950 --> 00:18:33.330

NM: Number 7? How many days was your child physically active for 10 min or more?

129

00:18:34.200 --> 00:18:35.090

Pa7: Oh, this

130

00:18:35.450 --> 00:18:38.980

Pa7: I give it a 5. It's important for your kids to be active. So

131

00:18:40.070 --> 00:18:42.590

NM: so you think it's a good question to ask a parent, because

132

00:18:42.830 --> 00:18:50.780

Pa7: because it it's good for you to know that you're actually helping your kid stay active. But it's a it's a part of being healthy. So

133

00:18:51.010 --> 00:18:52.210

Pa7: it's a good question.

134

00:18:56.310 --> 00:19:01.230

NM: And then the last one. How many days you shall run for 10 min or more?

135

00:19:01.530 --> 00:19:02.860

NM: How would you recap

136

00:19:03.840 --> 00:19:11.200

Pa7: for children with Cp? I mean, unless they are not able to. They're not ambulatory. Okay? So it's irrelevant.

137

00:19:11.390 --> 00:19:12.920

NM: Yeah, One Number

138

00:19:13.300 --> 00:19:14.150

Pa7: 0,

139

00:19:18.070 --> 00:19:20.280

NM: All right. We are near the end.

140

00:19:21.290 --> 00:19:37.010

NM: And so what? I've asked everybody at the last. It is to give me their final thoughts or comments about physical activity, as it pertains to being a parent of a child that is not inventory, but just in general like for anything you want to share

141

00:19:38.340 --> 00:19:49.080

Pa7: from. I mean being also that i'm like in nursing school learning a lot of how your body is posted function. I think that physical activity is like

142

00:19:49.250 --> 00:19:52.240

Pa7: It's super important along with like for me, because I always

143

00:19:52.320 --> 00:19:58.990

Pa7: put communication before physical activity, but I feel that it it goes hand in hand. Because

144

00:19:59.010 --> 00:20:11.420

Pa7: if you, if you can't communicate like how you want to move, and you know for a child like my my son. He can't tell me, you know. I want to get up, and I want to walk, or I want to just move my legs. Can you give me a range of motion? Can you?

145

00:20:11.420 --> 00:20:28.610

Pa7: You know, and these are things that you have to think for them? It it it's just as important. I don't know how to rate it, or to after communication that was hard to do in that survey, but it for me it's. It's important to have, like the be at your healthiest

146

00:20:29.640 --> 00:20:37.120

Pa7: it in with your body like you. You have to be able to move. You have to be able so when it comes to kids like ours it's

147

00:20:37.960 --> 00:20:57.050

Pa7: like it sucks for me, because, and also because we have to do the work for them, you know, like when we're gonna transfer them, or we're gonna do any activities with them. Or we're gonna implement therapy when there isn't a therapist to do it. It's more work on us. So you know it's it's it's like we're doing

148

00:20:57.050 --> 00:21:11.500

Pa7: what their body can't for them. and I feel that yeah, it might benefit us in a way, but it's not a 100% benefiting them, even though we're doing the best we can to help them. But physical activity is like

149

00:21:11.660 --> 00:21:13.810

Pa7: it's, and it's essential.

150

00:21:14.450 --> 00:21:15.330

NM: Hmm.

151

00:21:15.740 --> 00:21:16.780

Pa7: To be unhealthy

152

00:21:18.470 --> 00:21:19.860

NM: as well, said.

153

00:21:20.240 --> 00:21:22.860

NM: All right, Thank you. I must stop the recording. Okay.
