# Supplementary material for: Validity of PROMIS® Pediatric Physical Activity Parent Proxy Short Form Scale as a Physical Activity Measure for Children with Cerebral Palsy Who Are Non-Ambulatory
Source: Behav Sci (Basel). 2025 Jul 31;15(8):1042. doi: 10.3390/bs15081042 (PMC12382615; doi:10.3390/bs15081042)
Supplement: Supplementary file 1 [file behavsci-15-01042-s001.zip › Transcripts copy/Parent transcripts de-identified/Pa10.docx]

WEBVTT

1

00:00:01.160 --> 00:00:17.030

NM: All right. Well, thank you so much for joining us today. I have a few questions, as it relates to physical activity for children with cerebral palsy who are nonambulatory. First half is going to be some structured Questions with. Some follow ups about how you may define physical activity for your child.

2

00:00:17.030 --> 00:00:22.300

and some questions about your son's activity, and then the second half, I will ask you questions about

3

00:00:22.330 --> 00:00:37.000

NM: a survey that was created for parents to report on their child's physical activity, intensity. And if I sound scripted is because I am because I have to be as systematic as possible. So bare with me there. And are you ready to start?

4

00:00:41.340 --> 00:00:42.140

NM: Can you hear me?

5

00:00:42.730 --> 00:00:51.270

Pa10: Yeah. Okay. First question. How do you define physical activity for your child?

6

00:00:54.240 --> 00:01:09.840

Pa10: I think right now, just as much activity as we could do while using his power chair. I mean he's, you know he's not ambulatory, so all of the activities that he do. For the most part he uses, you know, using his motorized wheelchair.

7

00:01:09.840 --> 00:01:20.840

Pa10: You know he plays basketball at school. We go on walks. The most physical activity that he does outside of the power chair would be in the summer when he swims

8

00:01:20.840 --> 00:01:50.610

Pa10: with us at home. He also swims at school. They have an aquatic physical therapy program that he he's also a part of. So that's the most physical activity he does outside, and um stretching, you know, like we put him on the floor, so that he can kind of stretch out when he's like watching a show, or we're busy, so we'll like, Put him in the middle of the living room on, like, you know, like on on our carpet, and kind of like. Just roll around. So we try to make it. Make sure it's as clean as possible.

9

00:01:50.610 --> 00:02:01.360

Pa10: So that's the space, you know. So that's the most of the physical activity he does, you know. Maybe outside of the chair, but the rest of it would be in his power chair

10

00:02:01.590 --> 00:02:16.790

NM: alright. Thank you. And the department, is the first. Prompt. The Department of health defines physical activity as any activity that encompasses energy expended, and activation of skeletal muscles. Does this definition change how you view

11

00:02:16.880 --> 00:02:19.010

NM: physical activity for your child?

12

00:02:22.540 --> 00:02:41.210

Pa10: I mean I I guess not. I mean he does. I mean, I consider he also plays a lot of video games, and he only has full range of motion on his right arm, right hand. He can use the left, but it's a little bit more unstable.

13

00:02:41.600 --> 00:03:00.870

Pa10: you know, like something in it. He might throw it, but not voluntarily. It's like an involuntary kind of like spasm, almost, and he plays video games, but he uses his right hand to do everything like if the character needs to jump. He positions his hand in a way where he's still moving and jumping at the same time, like he's great at that.

14

00:03:00.870 --> 00:03:13.680

Pa10: you know. So he's able to, and we have a lot of. We designed a lot of assistant devices for him to be able to play different games, you know, like for all his games, he uses arcade stick.

15

00:03:13.680 --> 00:03:32.850

Pa10: you know, from back in a days from like, so he uses, you know, like the big art box, Arcade Stick with the joystick, and then the buttons are like right next to the joystick. So he command over his hands, and you can jump and move at the same time. So he does that excellently. And then we also, the Xbox has an an adaptive, remote control

16

00:03:32.850 --> 00:03:44.640

Pa10: that they designed for people with disabilities, and we've adapted that with switches and buttons. And we created this well, not we. I'm gonna say my husband created a huge

17

00:03:44.640 --> 00:04:00.880

Pa10: kind of system where now he's able to play even the games that require multi buttons, being pressed at the same time by creating switches where he just has to kind of like touch it, and multiple functions are done with one. But so he is able to buy all types of video games. So for me.

18

00:04:00.890 --> 00:04:19.230

Pa10: even though he's not able to ambulate He does a lot of physical activity based on his ability to play games, even when his lack of mobility may stop him. So I mean, yeah, he he's using his body. He's using his muscles, but maybe not in the same way that, like

19

00:04:19.230 --> 00:04:27.460

Pa10: others would be able to do it. So I I it still falls under the guideline of the definition of physical, you know activity. So I would say, Yes.

20

00:04:27.750 --> 00:04:34.470

NM: Okay. So now you now, with that definition, you're adding the video gaming because the truth is like you, said he's moving

21

00:04:34.520 --> 00:04:39.560

NM: so much just to activate. But now that this adapted, he's actually able to do everything

22

00:04:40.010 --> 00:04:43.110

NM: anyone can you do with the game which is awesome

Pa10: Yes

23

00:04:45.670 --> 00:04:51.050

NM: Oh, that is awesome. Okay? And how do you think physical activity differs from rest for your child specifically

24

00:04:51.830 --> 00:04:55.300

Pa10: physical activity, the first from rest.

25

00:04:58.310 --> 00:05:09.230

Pa10: Well, I I mean to be honest with you. I feel like the way that CP Is presenting in my son. He's always somehow exerting some kind of physical energy.

26

00:05:09.230 --> 00:05:38.170

Pa10: you know, even when he speaks, you know. And even though I I feel like speaking, is exerting some kind of energy. But with ‘child’, when he speaks sometimes his body stiffness stiffens up. He moves more of his body when he speaks, and we, you know, like sometimes like you know, being from Brooklyn. We speak with our hands a lot, being from Bronx people speak with their hands alot, I thinkg that’s a new York thing a lot,. but he kind of exerts his entire body when he's speaking, and like, let's say, if he really animated about something, his whole.

27

00:05:38.170 --> 00:05:47.270

Pa10: the whole body goes into kind of like a, you know it. It kind of stretches out somewhat like he. He He hyperextends his back and

28

00:05:47.270 --> 00:06:06.410

Pa10: his legs. You could tell that I was part of the Pt program because I use words that you guys…He does all the things that you know others wouldn't when they're like just having a kind of like a conversation, and even when he's resting.

29

00:06:06.470 --> 00:06:20.230

Pa10: you know we're constantly getting up in the middle of the night to make sure that he's not wrapped up in his sheets, because that's an issue because of the fact that he has limited mobility. He can move, but sometimes he gets himself into a a a position that he can't get himself out of.

30

00:06:20.400 --> 00:06:38.670

Pa10: you know. So even when he's sleeping, he's, you know he’s exerting energy. I I I think he's like one of the one of the only kids in the world with a six pack, and he's like 15 years old, and and literally, I mean, he has like muscles everywhere, you know. So you you you start to look at how, even when he's in

31

00:06:38.670 --> 00:06:41.930

Pa10: his downtime, let's say, or his rest time.

32

00:06:42.110 --> 00:06:59.930

Pa10: he's still exerting energy where maybe you and I, when once we hit rem, you know, body kind of stabilizes, you know. I like our breathing stabilizes, you know we're kind of like in a position, and we may wake up in that same position when we wake up in the morning. You know that kind of thing. That's not ‘child’.

33

00:06:59.930 --> 00:07:11.950

Pa10: you know ‘child’ constantly needs to be checked constantly needs to be positioned. You know better. His sheets need to be tucked in. He can't do a weighted sheet, because that's too heavy

34

00:07:11.950 --> 00:07:31.830

Pa10: and God forbid Something may happen to him, so we don't use them. He has railings at both sides of the bed, so that he doesn't roll off because he can't move. It's just once he gets into a bad spot. It's really hard to get out of it, so he's always exerting energy, so it's hard to determine what rest is happening. I I guess you know I've

35

00:07:31.830 --> 00:07:37.770

Pa10: quick answer to that question, and when he's not exerting some type of energy or a physical activity.

36

00:07:38.350 --> 00:07:41.850

NM: that's a great answer. Thank you.

37

00:07:44.530 --> 00:07:57.150

NM: And what would you say is the the optimal time when he you do feel like he lies his body. Is there something specific you can. He knows to do that, you know, to do, to try to get him to quiet as he as he can?

38

00:07:57.700 --> 00:08:12.300

Pa10: like, if he's like. Let's say you know he watches a lot of wrestling. He likes so so like Mondays, Tuesdays and Fridays are the days that wrestling comes on. So like if i'm home

39

00:08:12.300 --> 00:08:29.880

Pa10: during that time, like we'll eat dinner, and then wrestling is about 3 h long on Monday, so like after we eat dinner, i'll put him on the couch so he can lay out, and i'll sit next to him, and we'll watch it together. So one of the things that we like to do is to like position a pillow under his knees.

40

00:08:29.880 --> 00:08:37.360

Pa10: so that you know, so that he's not laying like he's not, cause he his leg is bent at the knee.

41

00:08:37.390 --> 00:08:42.250

Pa10: He doesn't. He can't fully extend his left leg

42

00:08:42.350 --> 00:09:01.160

Pa10: like his right, like we can, but his his left leg is very stiff. thankfully he doesn't have any like contractures as of yeah, like his hamstring, are very tight, which is why he gets stretched a lot to try to kind of alleviate that a little bit. So what we do is that we put a pull under his knees to kind of help him

43

00:09:01.260 --> 00:09:10.830

Pa10: relax, and a little bit more, relax his body, and not need to be like re-positioned too much, and also like if he wants a blanket or something. We tuck the blanket.

44

00:09:10.890 --> 00:09:18.410

Pa10: Tucking the blanket helps stabilize his body and keep him in the same place; and when he's on the couch he's sleeps

45

00:09:18.430 --> 00:09:20.280

Pa10: he sleeps on his stomach

46

00:09:20.660 --> 00:09:24.340

Pa10: or on his side, but he's on the couch he's on his back.

47

00:09:24.680 --> 00:09:29.310

Pa10: so there's less chance of him putting himself in a

48

00:09:29.360 --> 00:09:32.940

Pa10: not so great position where he needs to be repositioned.

49

00:09:33.950 --> 00:09:47.900

Pa10: If that answers the question, yeah. So like does those are things we do. We tuck the blanket, and when he goes to bed. We also tuck the blanket in. It helps him. It'll be better, and he also sleeps better because he likes. I think he likes the feeling of being.

50

00:09:48.530 --> 00:10:00.030

Pa10: you know, enclosed space, kind of like a cat. He likes feeling like he's in an enclosed space. So tucking a blanket helps yeah, and the and the pillow under his knees.

51

00:10:02.550 --> 00:10:17.760

NM: That's awesome. All right. So you then next question. Thank you. What activities would you consider your child does? Is physical activity. So you already gave me some good answers. Anything, maybe like the top 3, or the type of activities that you would consider your child does is physical activity?

52

00:10:19.190 --> 00:10:25.170

Pa10: Oh, I mean other than video games, I would say, like the the wheelchair basketball that he plays at school

53

00:10:25.580 --> 00:10:29.210

NM: and is that a that is a adaptive support, like after school?

54

00:10:29.830 --> 00:10:42.230

Pa10: No, no, no, he is. Well, I mean, yeah, like. Sometimes there's a after school activities for for the basketball team. He stays at school, but they also incorporated, I think, twice a week as part of gym.

55

00:10:42.390 --> 00:10:47.270

Pa10: because again he goes to a school where other children with special needs the majority of the children in the school

56

00:10:47.530 --> 00:10:57.700

Pa10: use a wheelchair for mobility. So you know they They've adapted every single thing in that school to, you know, accommodate the kids. So this is

57

00:10:57.700 --> 00:11:17.620

Pa10: part of his regular routine of school, and also and after school thing as well. And then in the summer, and, like I said, he swims, but he also swims in school, too. They have the aquatic program in school, so he does, swimming at school, home, the wheelchair, basketball team he's a part of, and then his video game activity at home.

58

00:11:17.620 --> 00:11:22.940

Pa10: And then, you know, I mean, we stretch him, and we do exercising with him, and he also gets in his adaptive equipment.

59

00:11:23.110 --> 00:11:33.590

Pa10: you know, like his stander, his gait trainer.

60

00:11:33.660 --> 00:11:39.970

Pa10: He does have a gait trainer, he he doesn't his gait, I mean he, ‘child’, can walk, but it needs to be assisted.

61

00:11:40.370 --> 00:11:51.600

Pa10: and also it's the gait. It's very unstable, like he, you know, like he he does almost like a hopping motion sometimes because he doesn't, he doesn't weight bear very much.

62

00:11:51.860 --> 00:12:10.920

Pa10: Yeah. And and some and his left leg sometimes is elevated like it's not not what he's, not bearing any weight on his left leg or his foot. So you know. So sometimes he uses his long leg braces, the ones that hinge at the pelvis. He uses those

63

00:12:11.030 --> 00:12:23.290

Pa10: yeah to stand up so he can tolerate that for a little while. Not much beyond that, the older. He got the less time he tolerates those, but he uses the the short AFOs of those every day

64

00:12:24.280 --> 00:12:31.400

NM: got it. And how long is he standing? And in this is using a standard as school, or is mostly at home.

65

00:12:31.760 --> 00:12:33.000

Pa10: Oh, I I

66

00:12:33.240 --> 00:12:47.870

Pa10: I want to say at home. If you get school they don't. I don't think they do it at school anymore, and he mostly does like, you know, like floor exercises and stretching. I mean, he's getting older. He's, you know. He's he's 16 at this point he's super tall, so it's it's it's

67

00:12:47.930 --> 00:13:01.480

Pa10: I I I think, for the most part I mean i'm not. You know we're not there when he's getting the Pts school. But when we have gone, the most thing we've seen is like them doing floor exercises with him, and a lot of stretching, because again it is hamstrings are very tight.

68

00:13:01.480 --> 00:13:20.210

Pa10: so they do. A lot of stretching with them, like the stander you mostly is that only tolerates it for a good like hour and a half, and we and since I work, you know, we can't do it every single day usually gets home from school like around. 5 o'clock, too, is in that. His school’s in Nassau, We live in Suffolk. It's about an hour and 10 min away.

69

00:13:20.300 --> 00:13:31.810

Pa10: you know, back and forth. So he's commuting for almost, you know, 2 and a half hours back and forth from school, so he gets in the stander. We have him on a schedule of Saturday and Sunday.

70

00:13:31.980 --> 00:13:35.430

Pa10: So Saturday you get on the standard for like an hour and a half Sunday as well.

71

00:13:40.850 --> 00:13:43.860

NM: Awesome? Okay. And so

72

00:13:43.960 --> 00:13:56.720

NM: I had a prompt, but you answered that so? Well, it was about using a depth of equipment. So you shared about that, and he's old. He's too old to probably go. Does he get on the playground swing. He probably did that when he was little like the

73

00:13:56.860 --> 00:14:16.580

Pa10: it. When he was little…There, I mean, you know. Sometimes we go upstate to visit my husband's family, and and they live near a playground that has an adaptive for swing where you can just put the entire powered chair. Yeah, but they don't have those around our neighborhood. So. No he hasn't been able to get into any.

74

00:14:16.580 --> 00:14:29.690

Pa10: you know. I used to get on a slide when he was little, but he's 16. Now, you know you know I do none of that. He want to play video games. He don't want to do that.

75

00:14:29.810 --> 00:14:36.780

NM: Gotcha Gotcha. And how do he has a joystick for the power chair? What hand is he using?

Pa10: The right hand?

76

00:14:37.550 --> 00:14:38.370

NM: Got it?

77

00:14:39.300 --> 00:14:53.270

NM: And so he's able to use with the he plays basketball, so I know he's reaching and doing the the ball toss. And how would you say his related services? P. T. Ot. I don't know if he gets vision or hearing. How would that relate to his physical activity?

78

00:14:53.560 --> 00:15:02.080

Pa10: He doesn't get the hearing he gets your services he does have. He uses hearing Aids and and

79

00:15:02.100 --> 00:15:09.570

Pa10: his teacher has the you know, the F Transmitter he uses for him. So he does get hearing services. He gets speech.

80

00:15:09.630 --> 00:15:31.630

Pa10: I think right now to be honest with you When we were when he was younger, and the diagnosis kind of hit us, we were more concerned about his ability. You know his mobility. Now we're less concerned. I mean. Now, what we want him to do is be able to socialize, to be able to, you know, be at, you know, active academically on point which you know which has been a struggle.

81

00:15:31.630 --> 00:15:49.500

Pa10: You know he is in the tenth grade, but his math and reading is like on a seventh grade level, you know. So there there There are challenges with with his academics, you know, I just. And this is social. Skills, really the mobility is something we hope

82

00:15:49.500 --> 00:15:53.650

Pa10: and we strive for. But we don't focus too much on.

83

00:15:53.760 --> 00:16:13.280

Pa10: We just want him to have a good quality of life, you know, as far as being happy, and at a at a certain point, we thought that that equated him, being able to ambulate him, be we.. That's not the case anymore. We understand that now. And so we just focusing on the other things.

84

00:16:14.090 --> 00:16:22.450

NM: Thank you. So does to a lot of these activities alone, or mostly in the group, and why?

85

00:16:22.780 --> 00:16:34.980

Pa10: it's mostly in a group. I mean when he's in when he's playing the wheelchair basketball, obviously with with his other classmates, and when he's swimming it's also with us, you know, because we're making sure he

86

00:16:35.110 --> 00:16:46.360

Pa10: you know it's just safe. So we're with him. You know he has like, maybe like a life preserver on, and he's, you know, trying to move around and it. But there is always somebody with him. He's never by himself.

87

00:16:46.430 --> 00:16:52.890

Pa10: So I I would say the I mean, the things he does independently are like when he's in a stander.

88

00:16:53.000 --> 00:17:11.329

Pa10: You know we give him something to entertain him, or he's watching something on TV. But he's in the stander. You don't by himself. That's not something that's done in a group when he plays video games. Sometimes it's by himself. Sometimes it's with someone else, you know, sometimes with all of us like we play family feud a lot, you know, or

89

00:17:11.329 --> 00:17:23.579

Pa10: you know we we play out of a multi-player games that require like 2 or 3 or 4 people. You know sometimes but a lot of times he plays alone. you know. He enjoys his alone time.

90

00:17:23.730 --> 00:17:32.450

Pa10: Sometimes you ask him if you want to. Hey do you want to play ‘I’m good, I want to play by myself’

91

00:17:33.570 --> 00:17:36.950

Pa10: but most of the things he does that requires, You know.

92

00:17:37.000 --> 00:17:45.340

Pa10: some type of assistance. Obviously he's with someone else. He does not have a para at school. Henry viscardi doesn’t do paras.

93

00:17:45.550 --> 00:17:52.070

Pa10: They have a teacher and a teaching assistant. But like if ‘child’ has to change classes, he goes by himself.

94

00:17:52.230 --> 00:18:09.060

Pa10: you know, like if he needs to go to pt, he goes by himself like he has his schedule. He looks at it. He knows where he has to go, so he just goes. That's they. They train the kids in school to be independent in that sense. So you know. So I I guess he does a mix of both independently and in a group.

95

00:18:12.720 --> 00:18:18.350

NM: I didn't know that they don't have Paras at

96

00:18:18.530 --> 00:18:30.680

Pa10: maybe like 4 or 5 years ago the State decided that the school did not need Paras, I I I They tried to fight it, but you know the only the only children that have

97

00:18:31.510 --> 00:18:45.190

Pa10: someone with them. While they have school as a children that are medically compromised like the children who have trachs and things of that nature. But a kid like ‘child’ Doesn't have any quote, unquote medical conditions.

98

00:18:46.640 --> 00:19:04.460

Pa10: He doesn't have anyone he does his own day. So yeah, they don't have Paras. They have like nursing assistance for those children who are medically compromised, but they will not have paras. They have. So in his classroom there's a teacher, and there's a teacher's assistant, and that's it. But again, Viscardi is is different, because their class sizes are super small.

99

00:19:04.460 --> 00:19:24.330

Pa10: They're not like district 75 schools where they can have, You know, a class of about you know. The average I believe, is 12, one to 2 or 12, one to one. Viscardi is not like that. His class. He got 6 in his class at any given day. It could be 4. Sometimes it's just him and someone else. Sometimes it's not even him, because it's just one other kid. So it's.

100

00:19:24.370 --> 00:19:35.910

Pa10: It depends, I mean, especially with Covid, because they still for a lot of the kids that are medically compromised, they still have Covid restrictions in for them. So some of the schools are Still, some of the kids are still streaming from home.

101

00:19:36.760 --> 00:19:38.000

Pa10: So it

102

00:19:38.580 --> 00:19:53.400

Pa10: you know they it. That school is is unique in that way, and I think that that's one of the reasons why we're happy. He got in there, you know, we avoided the district 75 catastrophe that could be the case Because.

103

00:19:54.150 --> 00:20:04.420

Pa10: yeah, that you know. I I I hand it off to people who have their kids in some of those districts 75 because I tell you we visited 3, and it was it was chaotic.

104

00:20:04.590 --> 00:20:11.050

and I I just. I didn't feel like my son would thrive in that environment, so i'm happy he got accepted to Viscardi.

105

00:20:13.490 --> 00:20:22.260

NM: I will stick to my script because I could totally go on a tangent with you on that. But I thank you for sharing. And so the last question.

106

00:20:22.260 --> 00:20:45.960

NM: I will just kind of focus in on specific things. So you you already shared. He. He stands for about an hour on an hour and a half on the weekends, and my last question is, how many times a week does your child participate in his activities? So you can include. You know. Pt: I know you make a hearing what other things that you know. If you have a frequency of, I can try to quantify how many times how many times a week these activities that for how long?

107

00:20:45.960 --> 00:20:54.820

Pa10: In school he does pt. Is 3 times a week for

108

00:20:55.000 --> 00:21:06.900

Pa10: 45 min sessions. The pt twice as individually, once is in a group, OT is twice a week, 45 min; that the period of 45 min long

109

00:21:07.880 --> 00:21:14.430

Pa10: hearing services twice a week. Speech is twice a week.

110

00:21:16.420 --> 00:21:19.450

Pa10: and you know he gets special instruction every day.

111

00:21:25.070 --> 00:21:44.740

NM: and for the swimming group is that daily in the summer at school, or is it like once a week? When is this so the swimming pool meet, and how long you know it's just,

Pa10: you know the swimming pool was like out of order for a while they just reopened it. But I think that they're you know they're waiting for it to get a new warmer outside before they start incorporating the kids

112

00:21:44.830 --> 00:21:55.620

Pa10: and the cool. Usually that's like once a week. It's, you know it's not something that's done every day, and I think it. you know, takes over one of the sessions for PT.

113

00:21:58.280 --> 00:21:59.640

NM: Got it? Okay?

114

00:22:00.450 --> 00:22:04.690

NM: And then you said OT was 2 times. You want to make sure you have that right? With 3. Okay.

115

00:22:05.070 --> 00:22:09.150

Pa10: Yeah. Yeah. PT: 3, OT: 2. Okay.

116

00:22:09.350 --> 00:22:17.100

Pa10: Speeches to hearing services to a special instructions is is every day every day.

NM: Does he give any assistive Tech?

117

00:22:18.480 --> 00:22:30.460

Pa10: No, he can. He he communicates, he used to, I mean, look at his hearing aids, I mean he does have hearing aids. But those are those are not the DOE doesn't Supply

118

00:22:30.570 --> 00:22:41.340

Pa10: the hearing aid that's through our insurance, you know. but he he does wear hearing aids but he doesn't use like a communicative like he doesn't

119

00:22:41.500 --> 00:22:45.400

Pa10: like a voice voice activated system. He doesn't use anything like that.

120

00:22:46.490 --> 00:22:48.070

NM: Got it. It's awesome.

121

00:22:48.600 --> 00:22:57.590

NM: all right. You mentioned that you do. Usually he gets some assistance, but there are the activities that he's independent in. And do you think he should participate in more or less of these activities, and why?

122

00:23:01.170 --> 00:23:09.460

Pa10: I mean, I would appreciate if he has more OT, because I want him to be able to use his right hand, and he'll be more in his left hand. I mean. I

123

00:23:09.460 --> 00:23:25.950

Pa10: I want him to be able to use that more, for, like, you know. Maybe he could use a little. I have the more stability, maybe not have to use it to actually like pick anything up or anything. But if he could stabilize himself with his left hand. It would open the door for him to be able to do much more stuff.

124

00:23:30.970 --> 00:23:32.180

NM: Hold on 1 s.

125

00:23:50.480 --> 00:24:03.140

NM: Sorry there's a class going on next door, and they just okay last. Okay, so yes, you can be asked for Walt and the right left hand awesome. So now we're gonna share the screen the last part of our chat.

126

00:24:03.660 --> 00:24:20.710

NM: And now again, just to give you a has up. This is the promise: physical activity, parent proxy, survey. So the parent will fill this out. There's 8 questions. And this was created for children that were not typically developing, not necessarily for Cp. But you know, as you may, as you know.

127

00:24:20.720 --> 00:24:37.370

NM: there's not really that many great skills for assessing physical activity, or looking at the so just trying to get a parents to give me their opinion about each question. So i'm going to ask you on a scale from 0 to 5. How appropriate this scale is 0 not appropriate at all for this population for your son.

128

00:24:37.430 --> 00:24:40.640

NM: 5 highly appropriate. This could be a question that

129

00:24:40.830 --> 00:24:50.160

NM: you you can see having some validity. And and why? Okay, so that'll be the last thing. And okay. So the first question

130

00:24:50.230 --> 00:25:01.090

NM: is, how many days did your child exercise or play so hard that it that his body got tired. How would you rate this question in terms of level appropriate? As for children with Cp. Who are not walking.

131

00:25:02.170 --> 00:25:03.010

NM: hold on.

132

00:25:07.040 --> 00:25:08.560

NM: and honest Don't

133

00:25:08.920 --> 00:25:10.520

NM: no hold back.

134

00:25:10.610 --> 00:25:21.560

I I just I I because yeah, there are some times where he's doing his exercising like Pt. Or we're stretching him, or he's in one of his adaptive devices, you know, and

135

00:25:21.860 --> 00:25:25.430

Pa10: he gets tired. I mean he does, I mean

136

00:25:29.180 --> 00:25:33.180

Pa10: I I get. I guess the play so hard

137

00:25:33.620 --> 00:25:35.890

Pa10: it's just not because it's.

138

00:25:36.330 --> 00:25:38.940

Pa10: You know it's I mean.

139

00:25:40.170 --> 00:25:48.700

Pa10: I don't know. I guess we'll we it differently. Yeah, I guess I would. You know just word it, and it will differently to you know

140

00:25:49.390 --> 00:25:59.900

Pa10: I mean to. But then, again, it depends on because Cp. Is such a broad range of of presentations. It's hard to Just look at this like just for my kid, like

141

00:25:59.930 --> 00:26:05.680

Pa10: I'm looking at it on my kid. Then, you know. Yeah, then the question should be worded a little differently, because

142

00:26:06.260 --> 00:26:18.360

Pa10: I mean the place so hard it's just not I. I I think that that I think that's what's bothering me the most. I guess the play so hard, because but it's a different type of play that maybe will not get someone

143

00:26:18.490 --> 00:26:21.120

Pa10: tired, for

144

00:26:21.780 --> 00:26:26.190

Pa10: I don't know if that makes any sense, I can. Yeah, go ahead for your child. Yep.

145

00:26:26.460 --> 00:26:32.000

Pa10: I guess for my kid, I would say, how many. how many days the child exercise

146

00:26:33.670 --> 00:26:34.980

Pa10: where

147

00:26:36.450 --> 00:26:39.430

Pa10: he expressed being tired.

148

00:26:40.000 --> 00:26:47.930

Pa10: like I would say something like that, because it it it just it the way that he is tired, and my tired is different.

149

00:26:48.870 --> 00:27:00.140

Pa10: So I I wouldn't so I would. I would. I guess I would word it that way as opposed to the way that it's worded

150

00:27:00.170 --> 00:27:04.450

NM: 5. Yeah for your bench.

151

00:27:09.990 --> 00:27:21.420

Pa10: What was the scales? 0? No, no, not not on there. It's 0 is not appropriate at all. 5 is highly appropriate and valid. So anywhere along that scale

152

00:27:21.760 --> 00:27:23.050

Pa10: 0,

153

00:27:23.210 --> 00:27:25.180

Pa10: maybe like a 2

154

00:27:25.810 --> 00:27:30.730

NM: awesome Thank you. And the feedback is very helpful. All right. Number 2.

155

00:27:30.900 --> 00:27:37.420

NM: How many days did your child exercise really hard for 10 min or more. How would you rate this question, and why?

156

00:27:37.800 --> 00:27:40.990

Pa10: Probably like a 2 again, because.

157

00:27:41.130 --> 00:27:46.220

Pa10: like when he's in it his devices, you know he's in there for like

158

00:27:46.610 --> 00:27:53.280

Pa10: hour hour and a half. It's more than 10 min, and I mean, I would think you know, with the way that

159

00:27:53.390 --> 00:28:03.740

Pa10: his body is presenting. It's hard for him, especially in the standard, because it's stretching our left leg, you know, really extending it. So I mean, I I I would

160

00:28:03.890 --> 00:28:07.640

Pa10: you know? Yeah, I I would say, like, maybe

161

00:28:08.770 --> 00:28:20.800

Pa10: it'd be like a like a 3 or 4. So you like this question better than the last one. Yeah, because like that, that's that's that's that's our interpretation. When he does as exercise.

162

00:28:22.220 --> 00:28:27.880

NM: So what i'm hearing and just correct what i'm wrong is that the interpretation has to be almost explained or

163

00:28:27.920 --> 00:28:29.880

understood by the parent.

164

00:28:30.070 --> 00:28:35.520

NM: When you ask this kind of question, because it it varies per child, is that.

165

00:28:35.840 --> 00:28:44.380

Pa10: you know, like like a child that maybe has. You know that the has Cp. But maybe also has, maybe is on a trade.

166

00:28:44.900 --> 00:28:50.720

Pa10: or maybe has absolutely no ability to wait there.

167

00:28:50.760 --> 00:28:51.610

NM: Yes.

168

00:28:51.690 --> 00:28:58.970

Pa10: their exercise will be different than ‘child’'s. So it's like, you know what I mean, so that word has to be explained.

169

00:28:59.060 --> 00:29:02.850

Pa10: and maybe even removed. Because, okay.

170

00:29:03.060 --> 00:29:16.240

Pa10: you know it. It has to be explained exactly what they mean, you know, like like you know, how many days did your child use any of the following assisted devices: Standard gay trainer.

171

00:29:16.370 --> 00:29:24.210

you know a a weight vest, you know, whatever those devices may be, and then arrange it as such.

172

00:29:24.840 --> 00:29:35.840

Pa10: I think that would be a better way to assess. You know how long that that child exercise and give an explanation of what the word exercise mean in that example.

173

00:29:38.040 --> 00:29:39.410

NM: This is so good.

174

00:29:39.430 --> 00:29:51.920

NM: Thank you. Alright, so give me a number for the question, though, as it is written, how would you? I mean, I could. I could like for like a 3 or 4. I would say it for okay. So this is a little better in terms of.

175

00:29:51.990 --> 00:30:10.930

Pa10: and that's that's because i'm interpreting what exercise means. You know what I mean. I don't know if every every parent would be, would do that, You know. Maybe they would take it. I just face badly. They would say, exercise. You can't wait there. You can't, you know. So this is not a good question, and then throw it out.

176

00:30:12.340 --> 00:30:14.970

NM: Yup. That's so good.

177

00:30:16.900 --> 00:30:27.800

NM: Okay, Next question. And this is exactly what i'm looking for. So thank you. Number 3. How many days your child exercise so much that he or she breathed hard. How would you rate this question?

178

00:30:28.570 --> 00:30:30.930

Pa10: It's it like a one.

179

00:30:32.030 --> 00:30:33.250

NM: Okay? And why?

180

00:30:36.600 --> 00:30:55.170

Pa10: I mean the green hard would be that the person is, you know, is is is using a lot a lot and an energy, you know, like when I think of breathing hard. I think of walking up a numerous slice of stairs. I think of Jumping Jack, or I think of running.

181

00:30:55.230 --> 00:30:56.420

You know

182

00:30:56.800 --> 00:31:02.550

Pa10: it's not the it's not the same. you know, for at least not for my kid.

183

00:31:02.570 --> 00:31:07.550

Pa10: So that's why I think like a one is just

184

00:31:08.280 --> 00:31:16.710

NM: not applicable. Really. Okay, that's one Number 4. How many days with your child so

185

00:31:16.740 --> 00:31:21.750

NM: physically active that he or she sweated. How would you rate this question?

186

00:31:24.220 --> 00:31:27.800

Pa10: Yeah, for my, I can actually let go. I mean

187

00:31:30.520 --> 00:31:31.960

Pa10: It's like a one.

188

00:31:32.010 --> 00:31:33.170

NM: Okay? And why

189

00:31:33.780 --> 00:31:50.610

Pa10: I I you know again, we you know my particular kid is not ambulatory, so everything that they do. They have to be in a, in, a, in a, in an environment, and in a position where he can use what he has to participate, and

190

00:31:50.750 --> 00:31:51.470

Pa10: it

191

00:31:51.620 --> 00:31:54.600

Pa10: it's it's usually not so.

192

00:31:54.910 --> 00:32:01.230

Pa10: Such a physically like in exerted activity that would cause him to like sweat.

193

00:32:01.570 --> 00:32:20.260

Pa10: you know, like some, it obviously like it depends on the environment again, like if he's playing wheelchair basketball. But the a/C is not running. He's gonna probably flatten it all if he's being stretched in the summer, in the house where we have like the windows open and the like, the door to backyard open.

194

00:32:20.260 --> 00:32:31.220

Pa10: and some reason is coming in, but he's it's not a/C he's gonna sweat. You know what I mean. So it's like, I don't know if maybe i'm reading too much into it. But for my particular kid that doesn't really apply. So i'm gonna go for one

195

00:32:31.440 --> 00:32:35.760

NM: 1 s. And I asked, because and i'll be honest some

196

00:32:36.150 --> 00:32:54.870

NM: some children or something, or, as I said, some of the truly just don't doesn't sweat, you know. Like it's just one of the things that okay, so he's that's when he's hot. He's West when he's for t. He's okay, all right. But even still it's not always related to his

197

00:32:54.870 --> 00:33:03.010

all right. Number. That was one of 4 Number 5. How many days did your child exercise a place so hard that his or her muscles bound?

198

00:33:07.950 --> 00:33:14.420

Pa10: I mean again, that probably would that about the Muslims burning would probably be a question I would have to ask him.

199

00:33:14.500 --> 00:33:17.190

Pa10: you know, because that would mean that he was sore.

200

00:33:17.470 --> 00:33:22.210

Pa10: and I would ask him, you know i'm stretching him

201

00:33:22.610 --> 00:33:28.830

Pa10: after judging him. Are you sure? Does anything hurt? If he says yes, then yes, but

202

00:33:30.350 --> 00:33:36.280

I mean I would have to ask him. So I I I mean, has there been times where he says that he feels a no sore?

203

00:33:37.180 --> 00:33:38.690

Pa10: I'm gonna go with you.

204

00:33:39.300 --> 00:33:42.000

Pa10: So I maybe a 3, or maybe a 3.

205

00:33:44.590 --> 00:33:47.800

NM: Okay, I got for 3. So

206

00:33:48.360 --> 00:33:58.920

NM: what I'm hearing and I don't want to pause the amount, but that you have to. They have to be able to explain it. The child has to be able to understand what soness is, or even express pain to do this.

207

00:33:59.080 --> 00:34:03.670

Yeah. And I would maybe change muscles, burn and stay.

208

00:34:04.190 --> 00:34:07.500

Pa10: I mean, you know, because I I don't.

209

00:34:08.630 --> 00:34:23.150

Pa10: I think if I was to say, ‘child’, do you do your muscles burn it to look at me like I'm crazy. I mean, this is not part of my You know what I mean. I don't know how many parents would say, hey, you know your muscles burning, You know I I I think that that language

210

00:34:23.250 --> 00:34:42.780

Pa10: it changed. I mean to say you know that they were so sure, or you know I don't know you. You feeling feeling a little bit of pain, because you know you stretch a kid who's tight. They're gonna feel a little. So after they be because you're forcing a stretch.

211

00:34:42.780 --> 00:35:02.230

Pa10: I mean you just literally what i'm doing when i'm pushing down on his leg. And i'm like, maybe stretching it out and like stabilizing his knee. I'm forcing a stretch. So you they're gonna feel so. But I don't know if it's burning so you know what I mean. That is it? Again, the exercise in my head, and that question is done being stretched.

212

00:35:02.930 --> 00:35:13.830

Pa10: But again, that may be just for my kid, because I know that there is, you know, Hypertonia. So there is a difference between the kids. My kid presents as a mix.

213

00:35:14.050 --> 00:35:26.880

NM: some some some areas he's a little bit more flexible than others, and in his life in particular he tends to be tight. So yeah, that's fair. Alright, Number 6.

214

00:35:26.990 --> 00:35:31.900

NM: How many days did your child exercise her play so hard that he or she felt tired?

215

00:35:32.460 --> 00:35:44.020

Pa10: I'd say 3 or 4. You know it's times it tells me. It comes home from too tired, especially after, you know, like wheelchair, basketball, or potentially strenuous Pt. Or ot session. So

216

00:35:44.270 --> 00:35:53.070

NM: so for parents give me give me one that is kind of hang your head on. Would you say this be a good question to ask parents in general? It's it's an appropriate

217

00:35:54.050 --> 00:35:57.480

Pa10: Yeah. I I'd say this is appropriate, but it yeah

218

00:35:57.670 --> 00:36:15.840

Pa10: again, boy, at the very beginning of this whole thing the word exercise needs to be explained. Exercise means physical therapy session stretching in assistant devices such as a trainer standard. You know the the the word exercise with these kids, how they identify needs to be.

219

00:36:15.840 --> 00:36:35.130

Pa10: It needs to be kind of dissected a little bit more, so that so that the parent isn't thinking, exercising the terms that we like, we would think of exercise, you know, in jail with the weight, you know. That's it. It's it's the way that exercise is presenting for these kids is different, and that needs to be explored.

220

00:36:35.130 --> 00:36:49.170

NM: Gotcha Gotcha. So 3 or 4 of you like this question. I think a little bit more is what it sounds like. Okay, Number 7. How many days was your child physically active, or 10 min or more.

221

00:36:52.750 --> 00:37:02.550

Pa10: I'd say for. But again, the physically active part needs to be explained. What Exactly. That means cause I I have to. I'll say right now for change up in my head. It's in playing video games.

222

00:37:14.270 --> 00:37:33.770

NM: Got you all right, and the last question, how many days is your child run for 10 min and more. How appropriate is this? 0? Not at all, you know. Yeah, yeah, if you're yeah, you know, if you're told that kid a non ambulance for you, the question is not even. You know. What does it sound to you? Right?

223

00:37:33.770 --> 00:37:53.980

NM: All right. So, as we wrap up, I would like to ask you for your final thoughts about physical activity in this population, any lasting comments. Everything you shared has been so helpful. But I always like to calls with the final fault from the parents and their input. So anything you would like to share about this activity, and it relates to your child and measures and definition.

224

00:37:54.280 --> 00:38:07.600

Pa10: Yeah, I just. I just think it just needs to be really defined. and it doesn't even have to be specifically defined for the the parent or the family that's going through the survey. It just needs to be like. What could physical activity need.

225

00:38:07.610 --> 00:38:17.710

Pa10: and there should be like a scale or some kind of explanation, so that when they're answering these questions, they understand how physical activity or exercise per change to their child.

226

00:38:18.300 --> 00:38:33.910

Pa10: and the way that it could, you know, because maybe to someone else, my son being in his power chair playing video games to them that does. That's not exercise. That's not physical activity, but because he's using his muscles.

227

00:38:33.910 --> 00:38:49.510

Pa10: We look at it as physical activity, maybe giving the definition that you gave earlier to the parents will probably also help them. But again, you know that's also explaining it's not because you're also you're dealing with families who are of

228

00:38:49.640 --> 00:38:56.860

Pa10: can I? You know they have different backgrounds and based on your background. You'll understand some of these studies more than others.

229

00:38:56.930 --> 00:39:12.650

Pa10: So these things have to be explained in in this terms, I would say very clearly, very detailed, so that they can effectively answer a survey like this, and, like the results. Don't get skewed in any kind of way, you know.

230

00:39:13.040 --> 00:39:18.630

Pa10: That's what I would say, you know, like exercise, physical activity playing hard.

231

00:39:19.670 --> 00:39:30.310

Pa10: I would take that out. I don't. I don't like that, because yes, my son plays, but you know, playing hard. I don't know I just. I don't like that for at least how I

232

00:39:30.350 --> 00:39:34.390

Pa10: and that is what you want for interpretation. I just think it's not.

233

00:39:34.610 --> 00:39:42.990

Pa10: You know. I don't think it's a good kind of like. I think exercise of physical activity works, you know, like my place. My son plays, you know.

234

00:39:43.240 --> 00:39:51.370

Pa10: You know he he he moves, you know. He he picks his card. He puts it down. That's physical activity. I guess that's playing

235

00:39:51.380 --> 00:39:54.610

Pa10: like you know. It's hard. I wouldn't say that.

236

00:39:54.800 --> 00:40:04.600

you know, so I I think I think we can leave this like a survey like this to to using the words either physical activity or exercise. But the play

237

00:40:06.360 --> 00:40:11.840

Pa10: I don't know. I guess I would remove that. I feel like playing falls on the more so social.

238

00:40:13.270 --> 00:40:16.680

Pa10: And I don't feel like this. Survey is asking about social

239

00:40:17.840 --> 00:40:22.060

Pa10: activities. You know what I mean. I I don't feel like it, so I I I would remove that.

240

00:40:23.980 --> 00:40:34.860

NM: That's all very helpful, Thank you, and I know you shared about the survey, but just in general about physical activity, as it relates to children with Cp. You are non ambulatory. Anything else you like to share. We wrap up.

241

00:40:35.100 --> 00:40:47.100

Pa10: just you know, defining what that means, you know, and and and and defining what it means to the particular. You know, family and child, that's in question. That's pretty much it I mean, everything else is is fine. It's just.

242

00:40:47.100 --> 00:41:02.770

Pa10: We can show that the family understands what that is, because you know that you that also could invoke a lot of feelings within the family. You know the word physical activity, you know. Let's say they. Child has has Cp. And is not ambulatory, but is also, let's say, you know, in a feeding tube and on a trade

243

00:41:03.090 --> 00:41:11.290

Pa10: you know, that really compromises their physical activity, and that just maybe something that evolves a lot of emotion in them.

244

00:41:11.470 --> 00:41:14.410

Pa10: And you know. So I I think that

245

00:41:14.640 --> 00:41:34.030

Pa10: really getting to know what the definitions of physical activity, exercise are for that particular family, and being able to describe that so that they could take this so so that they can understand it, and a very kind of a personal and individualistic way would help understand how to explain what that looks like for their kid.

246

00:41:39.870 --> 00:41:43.710

NM: That is wonderful. I must stop the recording. Thank you. Hold on 1 s.
