# Supplementary material for: Validity of PROMIS® Pediatric Physical Activity Parent Proxy Short Form Scale as a Physical Activity Measure for Children with Cerebral Palsy Who Are Non-Ambulatory
Source: Behav Sci (Basel). 2025 Jul 31;15(8):1042. doi: 10.3390/bs15081042 (PMC12382615; doi:10.3390/bs15081042)
Supplement: Supplementary file 1 [file behavsci-15-01042-s001.zip › Transcripts copy/Parent transcripts de-identified/Pa11.docx]

WEBVTT

1

00:00:01.650 --> 00:00:20.010

NM: Okay, good morning again. So thank you for meeting with me per our discussion. We're going to review the constant form so that I can. You gave me verbal approval to record this conversation. However, I do want to go through the entire constant form, for my.

2

00:00:20.010 --> 00:00:42.490

NM: So, as I mentioned, you were invited to participate in the research study call defining and measured physical activity in children with Cp. You may qualify to take part in this research study because you either are a parent of a child with Cp. Or or physical there who works with children. This interview will take approximately 1 h of your time. We've already been corresponding via phone conversation, and

3

00:00:42.570 --> 00:01:02.750

NM: we're using the zoom portal so that we can record the answer so that I can use it for research purposes Only this study is being done to investigate opinions of individuals who work and live closely with children, with Cp. To them, with Cp. May have limited level of selectivity for the more children with Cp. May not be able to process energy effectively, causing routine tasks more difficult

4

00:01:03.040 --> 00:01:05.730

compared to the age Ma match peers.

5

00:01:05.750 --> 00:01:33.610

NM: It's gonna go through what I'm asking you to do. Answer several questions as it relates to your education, family life and experiences answer questions as it relates to physical activity, definitions, measurements, and assessments, and children with Cp. Were not full walkers. There is minimal risk in this study, however you may feel about sharing, identifying information, to help reduce any concerns. I will inform you about the cost of taking the help, information, keep information confidential.

6

00:01:33.610 --> 00:01:51.900

NM: and to prevent anyone from discovering or guessing your identity. What possible benefits can I expect from taking part in a study? Well, there's no direct benefit for you to participate in a study. The results of this study may help to us to better understand physical activity. In Cp. We're not full time work, or there's no compensation for this study.

7

00:01:51.950 --> 00:01:54.730

NM: The study is over. After you complete the interview.

8

00:01:54.820 --> 00:02:14.580

NM: However, you can leave this interview any at any time, even if you have not finished in terms of protection, of your confidentiality. In instance of any electronic or computer malfunction, I will. I may. I need to collect handwriting materials, and Dr. M is still going to bring back the actual physical consent form to you.

9

00:02:14.640 --> 00:02:30.010

Pa11: Mrs. So that you can sign it and give it back to her. If that's okay, I do need to keep that. We don't sure. Sure I can send. I agree, and I will also do the writing one. Okay, thank you. So do you give your consent to be audio recorded.

10

00:02:30.250 --> 00:02:31.060

Pa11: Yes.

11

00:02:31.380 --> 00:02:35.440

NM: Do you give your consent to be video recorded? We're not doing that? So i'll just say no.

12

00:02:35.680 --> 00:02:36.990

Pa11: if that's okay.

13

00:02:37.420 --> 00:02:43.900

NM: Who may view your participation? You do you consent to allow written video and or recorded

14

00:02:43.930 --> 00:02:48.760

NM: materials to be viewed in the educational setting or at a conference outside of Teachers College.

15

00:02:50.050 --> 00:03:01.370

NM: That's okay. And can I contact you if there's any future study related to this.

16

00:03:01.580 --> 00:03:02.530

NM: and

17

00:03:04.070 --> 00:03:08.410

NM: and can I contact you? Related to this current study. Like to give you updated information about it.

18

00:03:08.530 --> 00:03:09.890

Pa11: of course. Okay.

19

00:03:11.390 --> 00:03:15.170

NM: All right, and you have part. You have rights as a participant.

20

00:03:15.180 --> 00:03:24.990

NM: and if you have read this or you you shared in terms of me sharing this information with you, Can I have your consent to sign the consent Form?

21

00:03:25.310 --> 00:03:26.040

Yes.

22

00:03:26.070 --> 00:03:28.360

NM: okay, Great. And how do you spell your first name?

23

00:03:28.810 --> 00:03:36.360

Pa11: V name is Ellie and G. S. Am.

24

00:03:37.030 --> 00:03:40.190

NM: Yes. and I have. Your first name is Bre

25

00:03:40.740 --> 00:03:44.130

NM: I and De. Why did I spell that out

26

00:03:44.300 --> 00:03:47.830

Pa11: without the ….

27

00:03:49.190 --> 00:03:50.600

NM: And

28

00:03:51.960 --> 00:03:53.560

NM: we are now?

29

00:03:54.730 --> 00:03:58.000

NM: Thank you. The next thing

30

00:04:03.020 --> 00:04:04.580

NM: I missed.

31

00:04:21.360 --> 00:04:25.130

NM: Oh, I know i'm sorry. I forgot to ask the scribble

32

00:04:26.090 --> 00:04:28.540

all your name. Hold on! Let me do that.

33

00:04:37.860 --> 00:04:40.710

Pa11: erm. You just said, it's gonna it will take an hour.

34

00:04:40.840 --> 00:04:45.350

NM: No, no, it shouldn't take longer than that. Now that we are here. Hold on.

35

00:04:49.270 --> 00:04:58.330

NM: Okay. And okay. Now that we're here. I just have to ask you this part. Is this about your

36

00:04:59.450 --> 00:05:01.760

NM: child? So how old is your child?

37

00:05:02.260 --> 00:05:06.040

Pa11: My child is 4 and a half now he will be 5 in August.

38

00:05:06.060 --> 00:05:07.260

NM: Okay.

39

00:05:07.960 --> 00:05:09.220

Pa11: little boy.

40

00:05:09.300 --> 00:05:12.070

Pa11: He's a boy

41

00:05:12.100 --> 00:05:18.840

Pa11: and the whole. Yes, he is the third in the family, and he has.

42

00:05:18.860 --> 00:05:21.730

NM: Were they? Okay? And the siblings ages.

43

00:05:22.570 --> 00:05:25.680

Pa11: So there's 9, 9 years, 11,

44

00:05:25.740 --> 00:05:26.710

Pa11: 7,

45

00:05:26.730 --> 00:05:34.410

Pa11: and then as himself, which is 4 and a half, and then we have a 2 and a half people

46

00:05:34.610 --> 00:05:36.700

NM: and your ethnicity.

47

00:05:38.470 --> 00:05:40.990

NM: or you don't have to. You don't have to answer if you don't want to.

48

00:05:41.620 --> 00:05:44.120

Pa11: It's okay. White. Non, hispanic.

49

00:05:44.450 --> 00:05:46.500

NM: Okay, got that

50

00:05:49.210 --> 00:05:59.190

NM: and age. If you don't mind, I have ranges. So are you between 20252030313536404140,

51

00:05:59.410 --> 00:06:03.630

NM: 26 to 30. Okay. And your highest at level of education.

52

00:06:04.710 --> 00:06:06.450

Pa11: I from college.

53

00:06:06.820 --> 00:06:08.940

NM: Yeah. And

54

00:06:08.960 --> 00:06:28.100

NM: your family goes on. Community Walks weekly. So you can indicate 0 is closer to to never 10 is most frequently so you can just give me a range from 0 to 10 for any of these questions. Okay, your family goes on walks, community walks week. You can range from 0 to 10.

55

00:06:28.510 --> 00:06:33.210

NM: How, on average, how frequently do you go? 10 would be all the time

56

00:06:33.620 --> 00:06:45.880

NM: right. You mean everybody in the family, or including your child. of course, with a job, I mean, you mean specifically with the father, mother, and all the kids or

57

00:06:45.930 --> 00:06:47.510

parts of the family with him.

58

00:06:47.930 --> 00:06:50.910

Pa11: parts of the family with him.

59

00:06:51.370 --> 00:06:52.170

Okay.

60

00:06:52.270 --> 00:06:55.480

NM: and you walk greater or equal to 5 blocks a day.

61

00:06:57.410 --> 00:06:58.960

Pa11: Well, I didn't. I'm sorry

62

00:06:59.010 --> 00:07:02.230

NM: of that. You want greater or equal to 5 blocks a day.

63

00:07:03.440 --> 00:07:04.640

Pa11: I'm not equal.

64

00:07:04.860 --> 00:07:11.050

NM: Oh, a 10. Okay. You walk greater or go to 20 blocks a day. You guys do that every day.

65

00:07:12.390 --> 00:07:13.940

NM: That's about a mile a day.

66

00:07:14.540 --> 00:07:16.860

Pa11: no less

67

00:07:17.320 --> 00:07:19.060

Pa11: right.

68

00:07:19.130 --> 00:07:22.480

NM: 50. I'll put a 5. That's a lot of walking.

69

00:07:23.630 --> 00:07:25.280

NM: I do that as far as

70

00:07:25.670 --> 00:07:27.850

NM: how would you rate that one?

71

00:07:30.250 --> 00:07:35.810

Pa11: I understand you. You asked me the first one like greater or equal to 5 right. So I said.

72

00:07:35.960 --> 00:07:48.190

NM: Right? Oh, you're asking each one how how often we do each one. Yes, yes, yes, yes. Oh, okay. So the 5 blocks is like all the time, and the 20 box is like, what would I say

73

00:07:48.370 --> 00:07:49.850

once in once in a

74

00:07:50.730 --> 00:07:54.180

Pa11: once a month and 50 black? Hmm.

75

00:07:56.340 --> 00:07:57.310

I don't know.

76

00:07:57.470 --> 00:08:05.170

NM: so should I range it like like in the middle of the road, because 0 is like never 10 is all the time. So maybe about 5

77

00:08:05.200 --> 00:08:07.930

NM: probably never

78

00:08:08.640 --> 00:08:10.870

Pa11: for 20, I would say, you know, like

79

00:08:12.380 --> 00:08:13.310

Pa11: So we

80

00:08:13.370 --> 00:08:17.450

NM: okay. And how often do you visit the community playground Park within the week?

81

00:08:18.940 --> 00:08:28.470

Pa11: So you know that's whether permitting now, in the winter 0 in the summer I do it often.

82

00:08:28.890 --> 00:08:30.950

NM: Okay. So we'll just go with the summer.

83

00:08:31.700 --> 00:08:33.100

Pa11: Okay.

84

00:08:33.549 --> 00:08:34.340

Pa11: 5.

85

00:08:34.480 --> 00:08:42.059

NM: Okay. And you spend equal or more than 30 min a day participating in moderate to vigorous exercise. Now this one is about you

86

00:08:47.740 --> 00:08:50.270

Pa11: one.

87

00:08:50.580 --> 00:08:57.350

NM: Okay, how about you spend more equal or more than 30 min per week engaging in.

88

00:08:58.400 --> 00:09:06.980

NM: That's the same question. The children in your household spend more than 30 min a day in moderate to vigorous

89

00:09:07.110 --> 00:09:09.780

NM: physical activity, whether in school or in the home

90

00:09:11.880 --> 00:09:12.900

5.

91

00:09:12.980 --> 00:09:18.540

NM: Okay. And on average, our our family spends 2 h and more a week watching television

92

00:09:19.060 --> 00:09:19.930

Pa11: 0.

93

00:09:20.060 --> 00:09:20.830

NM: Okay?

94

00:09:21.340 --> 00:09:28.600

NM: And and the last portion is I'm asking you to rank how important these areas are

95

00:09:29.980 --> 00:09:43.340

NM: for your rehabilitation goals for your child. Okay? So I'll give you the 4 categories for the first part, and then you tell me which is the most important is, for there's 4 options here. So the for I have academics.

96

00:09:43.450 --> 00:09:48.510

NM: communication. physical, well being and social participation. What's the most important?

97

00:09:49.960 --> 00:09:52.340

Pa11: Oh, hmm.

98

00:09:52.710 --> 00:09:54.420

Pa11: There is the

99

00:09:56.960 --> 00:10:03.230

Pa11: most important, I guess, would be physical

100

00:10:03.250 --> 00:10:08.270

Pa11: communication, and then social, and the last will be academic.

101

00:10:08.360 --> 00:10:10.620

NM: Okay? And then

102

00:10:10.910 --> 00:10:18.580

NM: the last one is which area of your life do you wish you had more free time for

103

00:10:18.580 --> 00:10:36.390

NM: okay. So this is specifically for you and the child with okay. So there's 7 options here, and you're going to rank them, based on which you had, which you had more time for. Okay. So I wish I wouldn't have had more time. Yes, exactly. Okay.

104

00:10:36.390 --> 00:10:42.310

Pa11: Yep. So i'll just list the 7. And this is really about your preferences. So we have self care.

105

00:10:42.420 --> 00:10:46.750

NM: We have career development, we have friends and time.

106

00:10:46.890 --> 00:10:52.810

NM: we have exercise and physical activity. We have hobbies. We have travel, and we have religion.

107

00:10:53.090 --> 00:10:59.570

NM: so I know that was a lot. So i'll say them again.

108

00:10:59.950 --> 00:11:04.060

Pa11: Which one would I have? I wished I would have had more time if what?

109

00:11:04.190 --> 00:11:07.490

NM: No, no, it's not. If what? Just in general for you?

110

00:11:07.670 --> 00:11:12.550

NM: Okay, no, actually not. I mean

111

00:11:13.160 --> 00:11:22.220

NM: as a parent. It's like from a parent's perspective, because it does relate to your family right? But at the end of the day this is really from a parents perspective for yourself. Yes.

112

00:11:26.110 --> 00:11:27.360

okay. So we did it again.

113

00:11:27.390 --> 00:11:34.510

NM: Which area of your life do you wish you had more free time for? Please write your selection one to 7

114

00:11:34.590 --> 00:11:38.120

NM: out of self- career, development.

115

00:11:38.330 --> 00:11:45.530

NM: friends and family time, exercise and physical activity, hobbies, travel, religion.

116

00:11:48.060 --> 00:11:51.070

NM: travel. Okay, One

117

00:12:00.730 --> 00:12:04.340

Pa11: should have written it down which one is before friends and family

118

00:12:04.870 --> 00:12:06.090

career development.

119

00:12:07.360 --> 00:12:08.860

Pa11: The first one was

120

00:12:08.950 --> 00:12:10.010

NM: self-care.

121

00:12:20.350 --> 00:12:24.100

Pa11: I'll read the the ones that are remaining. Okay.

122

00:12:24.530 --> 00:12:30.100

NM: Yeah. What's remaining is religion? Hobbies, exercise and physical activity. That's one

123

00:12:30.120 --> 00:12:36.400

NM: friends and family time, another career, development and self-care

124

00:12:36.630 --> 00:12:43.040

Pa11: exercise and physical activity. Okay. And then would be

125

00:12:45.510 --> 00:12:49.610

Pa11: religion. Okay.

126

00:12:50.810 --> 00:12:52.540

Pa11: my friends and family

127

00:12:53.330 --> 00:12:54.170

4,

128

00:12:54.500 --> 00:12:55.960

Pa11: and then

129

00:12:57.740 --> 00:12:59.320

Pa11: you mind me, the other ones

130

00:12:59.530 --> 00:13:03.220

NM: self-care career, development and track and hobbies are left

131

00:13:06.130 --> 00:13:09.250

Pa11: so. Career development self care and hobbies!

132

00:13:21.610 --> 00:13:22.760

Pa11: That was the order.

133

00:13:23.510 --> 00:13:31.740

NM: Oh, that was I'm: Sorry.

134

00:13:32.270 --> 00:13:33.170

Thank you. So much.

135

00:13:36.700 --> 00:13:39.500

NM: Awesome. Okay. So

136

00:13:44.770 --> 00:13:52.930

NM: we are at our interview. Okay, so thank you so much for taking the time. So if I sound scripted is because I am. And so I up. I

137

00:13:53.040 --> 00:14:06.600

NM: as you bear with me because I have to be as consistent as possible. I'm going to ask you 3 questions, and then I'm going to give you Some follow ups related to the initial question that the first half, the second half is the survey I was telling you about? All right. Are you ready to start?

138

00:14:06.940 --> 00:14:07.710

Pa11: Sure.

139

00:14:07.790 --> 00:14:11.700

NM: Great. How do you define physical activity for your child?

140

00:14:15.320 --> 00:14:16.990

Pa11: And what does he do?

141

00:14:18.080 --> 00:14:24.630

NM: Just how? Yeah, how you define it? We're gonna i'm gonna ask you what he does in a, in a moment. But how would you define it like? What would you?

142

00:14:25.210 --> 00:14:30.810

Pa11: How would you say physical activity for him? What it would mean? It would mean for him to

143

00:14:31.090 --> 00:14:33.720

Pa11: have his muscles

144

00:14:34.980 --> 00:14:37.080

Pa11 moved. And

145

00:14:39.180 --> 00:14:52.650

Pa11: yeah, I mean with help, you know, for now, but would be getting his feet flexed. Then his hands, you know. up and down, rotated and getting him all those physical inputs

146

00:14:52.940 --> 00:14:55.940

Pa11: so that he can stretch and

147

00:14:56.790 --> 00:14:59.060

Pa11: oh, pull his limbs and muscles

148

00:15:01.930 --> 00:15:19.590

NM: that's perfect. Thank you. And first prompt is the Department of Health defines physical activity as any activity that it comp. This energy extended, and activation of skeletal muscle. Does this definition change your mind about how you define physical activity for your child? Why or why not?

149

00:15:20.280 --> 00:15:25.340

Pa11: No, I think that really is a reflection of the definition

150

00:15:27.250 --> 00:15:31.830

NM: great. And how do you think physical activity differs from rest.

151

00:15:33.330 --> 00:15:34.930

Pa11: Sorry. How do I think

152

00:15:35.670 --> 00:15:38.510

NM: physical activity differs from rest?

153

00:15:40.310 --> 00:15:44.960

Pa11: Well, rest is when your muscles are not moving.

154

00:15:48.260 --> 00:15:50.180

NM: Thank you. Okay.

155

00:15:50.320 --> 00:15:57.410

NM: All right. Next question. What activities would you consider your child? Does physical activity?

156

00:16:02.140 --> 00:16:03.820

Pa11: Without assistance?

157

00:16:04.220 --> 00:16:05.790

NM: with or without assistance?

158

00:16:07.370 --> 00:16:13.180

Pa11: So, with the assistance it would be any time. We you know one of us or a therapist

159

00:16:16.110 --> 00:16:25.710

Pa11: do his routine exercise with him, and then for himself. It's when he is on his tummy, and he's.

160

00:16:26.080 --> 00:16:31.230

Pa11: and he learning, you know, trying to pick up his head and getting those.

161

00:16:32.200 --> 00:16:33.980

Pa11: or even when he's

162

00:16:35.230 --> 00:16:39.420

Pa11: and his stander. So he is being forced to

163

00:16:41.200 --> 00:16:50.280

Pa11: have his muscles, and and in being in alignment in certain ways, and then being able to be in the right position so that he can

164

00:16:50.330 --> 00:16:56.080

Pa11: use his arm. Let's say, and pick it up, and you know, try to get stuff

165

00:16:59.450 --> 00:17:14.119

NM: awesome. Thank you. And you did a great job giving me examples. I do have some habitual physical activities, such as use of that of equipment. So you mentioned the use of a stander? Does he use a gait trainer as physical activity.

166

00:17:14.450 --> 00:17:15.359

Pa11: No.

167

00:17:17.460 --> 00:17:21.990

NM: How about transitions in it out of his wheelchair? When you consider that physical activity?

168

00:17:26.119 --> 00:17:31.720

Pa11: Not much because he's being really, we have to just pick him up and place him

169

00:17:31.910 --> 00:17:32.940

Pa11: and to the next

170

00:17:33.120 --> 00:17:36.860

Pa11: place. So it's not much physical on his part.

171

00:17:37.080 --> 00:17:37.810

NM: Okay.

172

00:17:38.680 --> 00:17:43.750

NM: how about does he enjoy a playground swing? And would you consider that physical activity?

173

00:17:44.630 --> 00:17:54.690

Pa11: So he enjoys it a little bit more lately than he used to, Of course it has to be the right kind of swing with the right support.

174

00:17:55.410 --> 00:17:58.530

Pa11: just can’t be any of them, and then

175

00:17:58.550 --> 00:18:08.350

Pa11: and yes, it's physical activity when we give him that movement for his whole body right up and down.

176

00:18:08.450 --> 00:18:10.370

Pa11: and it's very

177

00:18:11.130 --> 00:18:14.070

Pa11: yeah. Of course it is a good input for him.

178

00:18:15.460 --> 00:18:16.250

NM: right?

179

00:18:16.800 --> 00:18:36.060

NM: And how about your child? I I don't know him. So how about his use of his arms. You mentioned that in the Stander..

Pa11: Right, so He has a very hard time using his arms. His right arm is stronger, though, and when in the right position.

180

00:18:36.060 --> 00:18:40.550

Pa11: we need to stay, either sitting or in the stander.

181

00:18:41.840 --> 00:18:48.290

Pa11: or if he's on like laying, but on the wedge, so that his arm is like available.

182

00:18:48.430 --> 00:18:50.880

Pa11: Then he can

183

00:18:51.240 --> 00:18:56.000

Pa11: the swipe or a press, or like, get it like a switch toy.

184

00:18:57.200 --> 00:19:03.940

Pa11: So he he but he has a hard time like to get to the right direction. He usually flexes at first back.

185

00:19:04.120 --> 00:19:09.530

Pa11: and you know, and then get this forward because he cant.

186

00:19:09.810 --> 00:19:12.670

Pa11: even though he knows where he wants to go with that arm.

187

00:19:14.100 --> 00:19:25.320

Pa11: You know his. His his brain has a hard, his brain, and the and the arm until they communicate where he needs to go, so he he goes like back first and then front, and then

188

00:19:25.550 --> 00:19:33.250

Pa11: it has the the item that he's trying to get at, and he's also. It needs to be in the right place in front of him, in his.

189

00:19:35.150 --> 00:19:41.100

Pa11: in his vision field first of all. and and in a place where he can.

190

00:19:42.160 --> 00:19:52.450

Pa11: We'll be able to get it. But then he can. He can swipe. He can press like switch toys.

191

00:19:54.980 --> 00:19:56.430

Pa11: He Can Do

192

00:19:56.550 --> 00:20:08.810

Pa11: you know, like, give me 5. Get your hand there. But yeah, so it needs to be a lot of things in place. He needs to be in the right position, and those, and the item needs to be in the right position

193

00:20:09.180 --> 00:20:22.910

Pa11: for him to be able to get at it. He can't isolate his fingers, though, like you wouldn't be able to just like press with a finger at a specific small spot. It needs to be a bigger thing that he can do it with his hand.

194

00:20:23.050 --> 00:20:28.850

Pa11: and sometimes he even uses either. His hand is sometimes uses even like the wrist part.

195

00:20:31.810 --> 00:20:33.070

Pa11: Do that stuff

196

00:20:36.250 --> 00:20:40.290

NM: that sounds awesome, great. And would you consider that physical activity?

197

00:20:40.470 --> 00:20:46.100

Pa11: Oh, Big time, Major physical activity for him. it takes so much so much

198

00:20:46.220 --> 00:20:48.090

Pa11: effort.

199

00:20:50.310 --> 00:20:52.320

NM: so much for sharing that.

200

00:20:53.060 --> 00:21:04.550

NM: And how would you relate? I mean, how does related services such as Pt. Ot. Vision, hearing, or speech relate to physical activity for your son?

201

00:21:07.190 --> 00:21:11.750

Pa11: Yeah. all of it really is connected in a lot of ways

202

00:21:13.740 --> 00:21:15.720

Pa11: right in order for his.

203

00:21:18.880 --> 00:21:23.690

Pa11: in order for him to be able to understand what we want from him

204

00:21:24.050 --> 00:21:28.600

Pa11: in therapies, in activities and physical.

205

00:21:28.850 --> 00:21:34.310

Pa11: For example, with a head control. He has a hard time. He doesn't have very good head head control.

206

00:21:34.410 --> 00:21:36.180

Pa11: So we're working on that a lot.

207

00:21:36.360 --> 00:21:48.030

Pa11: So of course we want him to understand what we're asking him to keep his head in place, or to, you know, go up, go down. So of course we need a lot of speech communication in that way.

208

00:21:48.130 --> 00:21:49.140

And

209

00:21:49.240 --> 00:21:51.720

Pa11: and then the OT and PT

210

00:21:51.740 --> 00:21:53.370

Pa11: work on different

211

00:21:53.650 --> 00:21:59.740

Pa11: areas of you know, getting him used to working and getting, am used to

212

00:22:06.450 --> 00:22:09.110

Pa11: exercising his muscles in different ways, which

213

00:22:09.200 --> 00:22:10.750

Pa11: adds up to really to the big picture.

214

00:22:13.890 --> 00:22:25.900

Pa11: of course, with vision and hearing in place, we need be able to know what he well, he does know what he doesn't know what he is seeing, and what he is hearing for us to be able to communicate with him, so that he understands and can

215

00:22:25.910 --> 00:22:28.590

Pa11: go forward in his physical activity.

216

00:22:32.010 --> 00:22:44.260

NM: That's great, Thank you. And does your child do some of these activities alone. I know you mentioned on the floor. He does do some land, and and but and most of it is in a group, and why or why not?

217

00:22:46.380 --> 00:22:50.320

Pa11: In a group? not necessarily in a group, but

218

00:22:50.330 --> 00:22:53.960

Pa11: it needs to be mostly with an adult prompting it.

219

00:22:55.140 --> 00:23:01.450

Pa11: Because so, A. Because he's just very social, and he wants that

220

00:23:01.580 --> 00:23:16.050

Pa11: he just, you know he just enjoys the company, and he doesn't enjoy being alone. As a matter of fact, most of the time when you just leave him alone. He starts crying, he's bored, and he wants that

221

00:23:16.310 --> 00:23:20.920

Pa11: he really wants to engage and and and enjoy a

222

00:23:21.060 --> 00:23:32.050

Pa11: social interaction. So that's just, I guess, his preference. But more than that. in order to be able to further and

223

00:23:32.160 --> 00:23:37.430

Pa11: develop his activities, we need to have an adult there to

224

00:23:37.470 --> 00:23:46.390

Pa11: prompt him first of all, to put him in the right position and have him in the right position the entire time so that he can

225

00:23:49.310 --> 00:23:55.340

Pa11: do the activities we want him to do, and he needs help with really everything.

226

00:23:55.370 --> 00:23:56.140

Pa11: So

227

00:23:58.320 --> 00:24:01.250

Pa11: So if we want him to

228

00:24:02.140 --> 00:24:10.090

Pa11: do any activity, we need to be there, hold him or position him, or make sure that he stays in the right position

229

00:24:12.630 --> 00:24:17.420

Pa11: and hope like, and also make sure that the item stays in the same position, or.

230

00:24:17.540 --> 00:24:24.350

Pa11: you know, really depending on what we're doing with him. But he very much is dependent on an adult.

231

00:24:26.290 --> 00:24:27.030

NM: I get it

232

00:24:28.540 --> 00:24:37.480

NM: all right and last question before the survey. How many times a week does your child participate in these activities, and for how long trying to get it his endurance.

233

00:24:38.090 --> 00:24:39.670

Pa11: So he is in school.

234

00:24:39.960 --> 00:24:40.620

NM: Okay.

235

00:24:40.970 --> 00:24:47.210

Pa11: So he gets everything there. He got Pt. And OT 3 times a week each.

236

00:24:47.470 --> 00:24:49.470

PA11: and

237

00:24:51.650 --> 00:24:53.340

NM: 3 times how long?

238

00:24:54.110 --> 00:24:58.260

Pa11: 3 times 30 min every, right. Every session is 30 min, so it's

239

00:24:58.320 --> 00:25:01.430

3 times Pt. 3 times OT, 3 times

240

00:25:04.320 --> 00:25:05.730

Pa11: speech

241

00:25:06.230 --> 00:25:10.820

vision therapy also comes, not as a set thing, but

242

00:25:10.830 --> 00:25:15.100

Pa11: here and there, probably once a week, or once in 2 weeks sometimes.

243

00:25:15.500 --> 00:25:21.040

Pa11: and then, besides, for that, he is in a classroom where they our 6

244

00:25:22.060 --> 00:25:25.300

Pa11: 6 of similar kind children to

245

00:25:25.540 --> 00:25:32.280

Pa11: four adults in the classroom. So they engage all the time, and they do all kinds of activities

246

00:25:32.860 --> 00:25:34.980

Pa11: that are appropriate for him

247

00:25:35.120 --> 00:25:35.800

Pa11: and

248

00:25:36.590 --> 00:25:41.690

Pa11: really furthering his goals and all these areas.

249

00:25:44.990 --> 00:25:55.180

NM: It's great. And you mentioned that he does need assistance during these activities. The reaching once he's set up.

250

00:25:55.510 --> 00:26:03.460

NM: he's able to actually do the reaching, or does he still need to queue occasionally to bring the hand forward because it's not working to do that and forward on his own

251

00:26:03.680 --> 00:26:05.510

Pa11: right, so he

252

00:26:07.680 --> 00:26:16.640

Pa11: he needs the stimulation. He need to be reminded that there's something there, and you know, like, reach at it again or

253

00:26:17.190 --> 00:26:21.010

NM: on his own. He'll just get bored like you he won't.

254

00:26:21.300 --> 00:26:22.150

Pa11: Yeah.

255

00:26:22.770 --> 00:26:30.070

NM: Got it. And do you think you should participate in more or less of these activities. And why?

256

00:26:32.100 --> 00:26:41.000

Pa11: the more is always better. The of course there's no limit as as much as we can do. It's never enough. He always needs more and more and more, because

257

00:26:41.180 --> 00:26:44.890

Pa11: that's what's just going to develop and cultivate his skills.

258

00:26:45.250 --> 00:26:56.440

Pa11: even right. There's no…we have a lot of a lot of things in mind, and it's never too much so I can't say that he's getting too little, but it's never enough so as much as we can.

259

00:26:57.150 --> 00:26:57.980

NM: Great

260

00:27:01.960 --> 00:27:29.730

NM: right, all right. So now I'm going to read some of the questions on the survey. This was the survey is called promise parent proxy, physical activity, scale. and it was developed for children that were not typically developing. Ideally it was originally creative children. They were going through cancer, and they were regressing, and so the parent was reporting for the child their level of physical activity, intensity. And so I wanted to get the feedback from

261

00:27:29.870 --> 00:27:38.300

NM: my participants to see how you would rate the question. So you're not going to grade your child with these questions. You're actually going to grade the question.

262

00:27:38.320 --> 00:27:43.070

NM: And so, and you're going to tell me how appropriate you think it is to ask a parent

263

00:27:43.570 --> 00:27:50.960

NM: how appropriate the question is to ask a parent, when a child with Cp. Who is not a full time work, Walker. So i'm going to ask you to rank it.

264

00:27:51.080 --> 00:27:54.780

NM: 0. Not appropriate at all.

265

00:27:54.880 --> 00:28:00.980

5 highly appropriate a good question to ask a parent, and then I'm going to ask you to tell me why. Okay?

266

00:28:01.210 --> 00:28:02.040

Pa11: Okay.

267

00:28:02.410 --> 00:28:04.240

NM: So the first question is.

268

00:28:05.400 --> 00:28:11.440

NM: how many days did your child exercise or play so hard that his or her body got tired?

269

00:28:12.050 --> 00:28:25.360

NM: So, just looking at that question, would you say this is an appropriate question to ask a parent with a child like yours, and why so 0? And how many Again, just repeat the question, how many days

270

00:28:25.440 --> 00:28:31.820

NM: did your child exercise or play so hard that his or her body got tired. How would you rate that question?

271

00:28:32.550 --> 00:28:37.560

Pa11: Okay, that's fine. I'm saying that's appropriate.

272

00:28:38.000 --> 00:28:41.540

Pa11: What 5 is appropriate 5 is the highest you can give it.

273

00:28:41.810 --> 00:28:46.610

Pa11: Yeah. And why? Because it's

274

00:28:47.700 --> 00:28:48.760

Pa11: It's so.

275

00:28:48.800 --> 00:28:49.490

So.

276

00:28:51.930 --> 00:28:52.850

Pa11: It like.

277

00:28:54.270 --> 00:28:55.490

Pa11: So

278

00:28:56.890 --> 00:28:58.330

so it is just not

279

00:29:00.270 --> 00:29:05.290

Pa11: so. What they do I'm saying, this is what they they keep doing, and this is exactly what

280

00:29:05.430 --> 00:29:08.110

keeps happening, and it's such a

281

00:29:09.000 --> 00:29:12.980

Pa11: you know, on the on the on target, but it's not

282

00:29:13.050 --> 00:29:15.650

Pa11: too sensitive, or

283

00:29:15.970 --> 00:29:24.590

NM: that's good enough. all right. So the next question is, how many days is your child exercise really hard for 10 min or more.

284

00:29:24.620 --> 00:29:26.440

NM: How would you rate that question?

285

00:29:27.250 --> 00:29:37.080

Pa11: 5. Okay. And why? I think 10 min is very so minimal. So I don't think it should be a problem.

286

00:29:39.840 --> 00:29:41.790

NM: not a problem for a parent to answer.

287

00:29:41.950 --> 00:29:43.580

Pa11: Right? Okay.

288

00:29:45.850 --> 00:29:55.010

NM: All right. 3. How many days did your child exercise so much that he or she breathed hard. How would you raise that question?

289

00:30:09.550 --> 00:30:21.160

Pa11: 5. I don't. I I don't know. I just don't. I don't have a problem with any of these questions. They're just about the you know, just the the logistics about their physical activity. And

290

00:30:21.220 --> 00:30:29.790

I think these children are so busy with physical activity, and this is what ties them, and it's interesting to see. You know the differences of

291

00:30:29.840 --> 00:30:31.900

Pa11: how they tell you why they tie your

292

00:30:31.930 --> 00:30:36.780

if they get better at it. You know with more physical activity they get used to it.

293

00:30:36.990 --> 00:30:40.440

Pa11: So I don't see that it should be appropriate.

294

00:30:40.620 --> 00:30:49.390

NM: I agree. Next question number 4. There's only 8 questions. Number 4. How many days was your child so physically active that he or she sweated.

295

00:30:50.160 --> 00:30:51.450

NM: How would you rate that one

296

00:30:55.530 --> 00:30:56.460

Pa11: bye?

297

00:30:56.950 --> 00:30:57.660

NM: Okay.

298

00:30:59.050 --> 00:31:01.860

NM: Does your son sweat. Are you right now?

299

00:31:02.750 --> 00:31:03.760

Pa11: So he

300

00:31:03.800 --> 00:31:19.790

he used to as a child like when he was even younger, I think. But no, not really. And I guess maybe maybe with the older child it would be a more personal. But with a young child I don't see that it should be too.

301

00:31:23.990 --> 00:31:26.520

NM: So you see this question more for an older channel.

302

00:31:27.380 --> 00:31:33.770

Pa11: No, I'm saying, maybe with an older child there would be a more personal question, but with a young child that wouldn't have a problem with it.

303

00:31:34.020 --> 00:31:35.720

NM: Okay, okay, Got it?

304

00:31:38.280 --> 00:31:48.310

NM: You said a personal question. Okay. all right. Number 5. How many days did your child exercise and play so hard that his or her muscles burned.

305

00:31:50.110 --> 00:31:54.980

Pa11: that his

306

00:31:55.040 --> 00:32:01.080

Pa11: so again, like I don't, I think it's appropriate. It would just be something I would.

307

00:32:02.080 --> 00:32:04.440

Pa11: because of the kind of child might

308

00:32:04.500 --> 00:32:10.040

child is, he's not expressive? I would have no way of knowing. So

309

00:32:11.080 --> 00:32:18.040

NM: So this is. This is a challenge with this survey, because I think you're looking at it from. you know.

310

00:32:18.740 --> 00:32:26.150

NM: just in terms of questions, you know, but in terms of like actually being engaged for our the kids that we're talking about.

311

00:32:26.360 --> 00:32:38.540

Pa11: So for me, this would be inappropriate. Because I was okay, I wouldn't know. Yeah, okay. So this one's inappropriate. Okay, got it okay, because of the communication piece.

312

00:32:42.030 --> 00:32:50.170

NM: And it's hard, because most of our kids will know that that feels like either. You know what i'm saying like how to? How would you rate this one?

313

00:32:50.250 --> 00:32:52.260

NM: How would you give me a number for it?

314

00:32:52.600 --> 00:32:53.450

Pa11: Okay.

315

00:32:55.960 --> 00:32:59.570

NM: What? How? What number would you rate that one about the muscles burning.

316

00:33:00.850 --> 00:33:05.300

Pa11: Oh, I didn't understand. That would be 0, I guess.

317

00:33:05.320 --> 00:33:07.250

NM: Okay, Not at all. Okay.

318

00:33:07.280 --> 00:33:22.780

Pa11: All right. Number 6. How many days your child exercise or play so hard that he or she felt tired. How would you rate this question for all the time? We just, you know they get sleepy right after therapy.

319

00:33:25.120 --> 00:33:31.210

NM: Okay, Great and Number 7 is how many days was your child physically active for 10 min or more?

320

00:33:33.150 --> 00:33:34.340

Pa11: Would you say it

321

00:33:34.480 --> 00:33:41.580

NM: that the other one was about exercise like the word was exercise, and this one is physically active. So that's the difference.

322

00:33:41.880 --> 00:33:42.710

Oh.

323

00:33:45.390 --> 00:33:46.410

Pa11: so

324

00:33:47.680 --> 00:33:51.310

Pa11: oh, is he? Yeah, I would be 5,

325

00:33:52.490 --> 00:33:58.830

Pa11: and, as you said before, with appropriate because of the 10 min timeframe easier than to.

326

00:34:05.110 --> 00:34:07.830

NM: And the last one question is

327

00:34:08.699 --> 00:34:14.770

NM: number 8: how many days is your child run for 10 min or more? How will you rate this question

328

00:34:14.840 --> 00:34:18.520

Pa11: run? Yes. Oh, I wish

329

00:34:20.270 --> 00:34:22.340

Pa11: that would be 0. Yeah.

330

00:34:22.860 --> 00:34:24.080

NM: it's not a.

331

00:34:30.480 --> 00:34:46.670

NM: And my last question as we wrap up is, if you have any final thoughts about physical activity in this population, any you know, suggestions concerns things you want to share as your final thoughts about how we define and measure physical activities for these children.

332

00:34:48.250 --> 00:34:50.679

Pa11: Oh, so I mean just i'm just

333

00:34:50.730 --> 00:34:56.350

Pa11: typing in Jennifer just texted me if she can drop it off at 1150.

334

00:34:56.909 --> 00:35:06.770

Pa11: So you want me so to do it right. You can just sign it and give it back to her, because that everything else you don't need to fill out because we did it together. Okay, fine.

335

00:35:07.040 --> 00:35:12.410

Pa11: So sorry I I I my final thoughts. So

336

00:35:14.950 --> 00:35:18.580

physical activity for these children is really like.

337

00:35:18.730 --> 00:35:39.240

Pa11: you know, like oxygen for us, it's something that they probably wish to have more for more more of. I see it with his whenever he gets physical activity. I see how he really got to enjoy it like maybe in the beginning it was hard for him, and he used to cry he was in, you know he's he's so stiff.

338

00:35:39.540 --> 00:35:48.250

and it was really work for him, and he was used to it. But the more he feels it, and the more he sees what it dust in, and

339

00:35:48.500 --> 00:35:50.610

Pa11: how it really makes him feel, he just

340

00:35:50.730 --> 00:35:55.060

he so enjoys it, and he loves it, and it makes me just think that I wish.

341

00:35:56.740 --> 00:36:05.980

Pa11: you know. I wish I would have more time and and and just be able to do it for him all day. But really our goal is to train him, and

342

00:36:06.200 --> 00:36:09.410

see as much as we can to

343

00:36:10.100 --> 00:36:14.680

Pa11: make him able to do as much as he can on his own independently.

344

00:36:16.510 --> 00:36:17.600

Pa11: because

345

00:36:18.600 --> 00:36:21.340

Pa11: that's that's where we want him to get it.

346

00:36:22.600 --> 00:36:24.730

and the more we can

347

00:36:25.780 --> 00:36:36.590

Pa11: up our understanding of how to help them in physical activity is just. Every every little bit makes such a huge difference and impact on their lives and on their

348

00:36:38.150 --> 00:36:41.040

Pa11: quality of life. So

349

00:36:43.300 --> 00:36:43.920

no

350

00:36:44.580 --> 00:36:45.610

NM: ask one.

351

00:36:47.290 --> 00:36:50.050

NM: thank you so much. I'm going to stop the recording.
