# Supplementary material for: Validity of PROMIS® Pediatric Physical Activity Parent Proxy Short Form Scale as a Physical Activity Measure for Children with Cerebral Palsy Who Are Non-Ambulatory
Source: Behav Sci (Basel). 2025 Jul 31;15(8):1042. doi: 10.3390/bs15081042 (PMC12382615; doi:10.3390/bs15081042)
Supplement: Supplementary file 1 [file behavsci-15-01042-s001.zip › Transcripts copy/Parent transcripts de-identified/Pa6.docx]

WEBVTT

1

00:00:01.210 --> 00:00:05.380

Pa6: Okay, all right. Good afternoon. Thank you so much for taking time

2

00:00:05.380 --> 00:00:24.330

NM: to chat with me. Today I am in the process of interviewing parents with children, with Cp. About physical activity, and how they view it as it relate to their child. It was potentially not a full time, Walker. So I'm going to ask you a set of questions for the first half, and there's no right or wrong Answer. If this.

3

00:00:24.330 --> 00:00:43.500

NM: if it sounds scripted, is because I am. I have to be as consistent as possible. Everyone I have follow ups after each question, and then the second half of our interview. I will show you a a parent proxy. Physical activity survey that was developed by the the the National Institute of Health.

4

00:00:43.500 --> 00:00:48.220

NM: and I'm. Going to ask you questions related to that survey. Are you ready to?

Pa6: I am ready

5

00:00:48.370 --> 00:00:57.790

NM: Awesome. Thank you so much for your time, All right. So the first question I have for you is, how do you define physical activity for your child?

6

00:01:03.450 --> 00:01:17.000

Pa6: Well, ‘child’ is not He's nonambulatory. So physical activity for him looks like a lot of stretching

7

00:01:22.000 --> 00:01:28.740

Pa6: a lot of practice standing. He does not practice walking.

8

00:01:30.660 --> 00:01:36.640

Pa6: We do. We do go swimming when we can.

9

00:01:40.350 --> 00:01:42.000

Pa6: And

10

00:01:43.330 --> 00:01:45.280

Pa6: yeah, that's that's probably about it.

11

00:01:45.450 --> 00:01:55.220

NM: That's great. So the first follow up is the department of Health defines physical activity as any activity that encompasses energy expended.

12

00:01:55.300 --> 00:02:03.090

NM: and activation of skeletal muscles. Does this definition change your mind about how you define physical activity for your child. Why or why not?

13

00:02:03.720 --> 00:02:11.060

Pa6: [In response to DOH definition] It does because, ‘child’ laughs a lot, and he laughs with his entire body.

14

00:02:11.460 --> 00:02:21.190

Pa6: and he's definitely expending energy. He also kicks his legs when he laughs. So there's a lot of energy expended there. So I think

15

00:02:21.230 --> 00:02:23.400

Pa6: you know, using that definition.

16

00:02:23.710 --> 00:02:33.240

Pa6: he definitely gets a lot more exercise than noted before, because he is a very happy child, and he's always laughing.

17

00:02:35.540 --> 00:02:36.960

NM: That's awesome.

18

00:02:37.360 --> 00:02:42.270

NM: And how do you think physical activity differs from rest?

19

00:02:44.280 --> 00:02:49.300

Pa6: Oh, well, when, ‘child’s resting, he's clearly just. You know he

20

00:02:52.550 --> 00:02:57.160

Pa6: he's relaxed. He's completely.

21

00:02:57.960 --> 00:03:05.460

Pa6: You know. I you know his body is relaxed. He's not using his tone to, to do anything

22

00:03:05.530 --> 00:03:10.240

Pa6: he's not. His tone is not acting up.

23

00:03:10.510 --> 00:03:18.880

Pa6: You know he's just has a very relaxed body, and he can be watching television. So his mind is active.

24

00:03:19.070 --> 00:03:24.260

Pa6: but his body is relaxed. And you know, I think that's different from

25

00:03:24.450 --> 00:03:31.650

Pa6: activity and just the fact that when, ‘child’ is active in any way.

26

00:03:32.420 --> 00:03:43.290

Pa6: you know, his, dystonia and his hyper- hyper[tonia] tone acts up

27

00:03:43.320 --> 00:03:44.170

Pa6: a lot.

28

00:03:46.120 --> 00:03:47.090

NM: Thank you

29

00:03:47.300 --> 00:03:55.390

NM: all right. Second question. You gave me some great examples already. Actually, what activities would you consider your child does as physical activity.

30

00:03:56.580 --> 00:04:07.660

Pa6: definitely swimming for sure. stretching. practicing standing. laughing, kicking.

31

00:04:07.930 --> 00:04:09.940

Pa6: horseback riding.

32

00:04:17.140 --> 00:04:25.200

Pa6: Yeah, occasionally he does arts and crafts which is using his hands. So that's definitely an activity.

33

00:04:28.390 --> 00:04:32.630

Pa6: Yeah, I I think that would probably be about most of them. Yeah.

34

00:04:32.700 --> 00:04:47.830

NM: okay, great. And I I do have some prompt. So if unsure we can discuss some of his habitual activities. So you already mentioned the stander he's not using the gait trainer. How about transitions in and out of the wheelchair? Would you consider that physical activity for him.

35

00:04:48.490 --> 00:05:06.610

Pa6: Yes, he does. He. He actually just to clarify. He's not using the stander right now, but he does stand with a a therapist, and most of the standing is transfer related. So he does sit to stand

36

00:05:06.610 --> 00:05:11.520

Pa6: to help with

37

00:05:11.640 --> 00:05:20.140

Pa6: wheelchair transfer. However, he still a little bit too small to do a wheelchair transfer. He can't actually

38

00:05:21.610 --> 00:05:27.550

Pa6: come to the edge of his seat and stand up. He's still in a stroller, so he can't really.

39

00:05:28.560 --> 00:05:30.000

Pa6: He can't

40

00:05:30.190 --> 00:05:33.400

Pa6: transfer from his wheelchair, but they do practice it.

41

00:05:33.740 --> 00:05:34.410

NM: Got it.

42

00:05:35.830 --> 00:05:42.570

NM: Okay. And so how about when he's being picked up in and out of a a chair or a bed.

43

00:05:42.850 --> 00:05:44.880

NM: Is he active during that period?

44

00:05:46.240 --> 00:05:54.060

Pa6: Well, he can be, but we try and quiet him down because it's dangerous for us to pick him up if if he's too active.

45

00:05:54.240 --> 00:06:05.020

Pa6: But if he's super excited about what's gonna happen, he does go into hyper extension and we'll kick his legs in excitement.

46

00:06:05.170 --> 00:06:13.960

Pa6: But we try to keep him as as still and quiet as possible, just not to hurt the person or people who are transferring him

47

00:06:14.110 --> 00:06:20.950

NM: great. And how about the playground swing or adaptive swing. Does he enjoy that? Would you question that physical activity?

48

00:06:21.140 --> 00:06:23.860

Pa6: He does enjoy that

49

00:06:24.610 --> 00:06:38.370

Pa6: And yeah, I would consider it. I would consider it more vestibular activity because he's often not active on the swing. He's just sitting on the swing, but it is definitely vestibular activity

50

00:06:38.680 --> 00:06:39.380

NM: Gotcha, awesome.

51

00:06:39.560 --> 00:06:51.880

NM: And then how about you Mentioned You saw his arms for arts and crafts. How about reaching or Ball Toss?

Pa6: Yes, he. He also, during he also reaches for

52

00:06:52.040 --> 00:06:58.180

Pa6: things pretty regularly, and he does

53

00:06:58.370 --> 00:07:05.910

Pa6: try to play using his hands and arms as much as he is capable of.

54

00:07:05.920 --> 00:07:17.370

Pa6: And I did forget to mention that he also plays musical instruments. So that's definitely activity, using his hands

55

00:07:17.520 --> 00:07:29.100

NM: all right. And then how do you relate? I mean, how does related services such as Pt. Ot. Vision, hearing, education relate to physical activity.

56

00:07:30.640 --> 00:07:41.090

Pa6: So after this is not a recipient of vision or hearing, but PT, Ot and academics. Was it that you said Academics.

57

00:07:43.100 --> 00:07:48.250

Pa6: They really. you know, physical therapy

58

00:07:48.590 --> 00:08:00.910

Pa6: is stretching mainly the lower extremities, putting on his AFOs, practicing, standing, doing, sit to stand sitting up right

59

00:08:00.960 --> 00:08:14.510

Pa6: strengthening core muscles and neck muscles. Occupational therapy is mostly stretching of the upper extremities, his hands, his fingers, his arms also

60

00:08:14.560 --> 00:08:19.320

Pa6: encouraging him to reach for things to roll

61

00:08:19.390 --> 00:08:21.680

Pa6: to

62

00:08:22.130 --> 00:08:26.900

Pa6: play with a ball or play with a toy to

63

00:08:29.380 --> 00:08:38.330

Pa6: basically just to to he uses hands. He also uses his functional hands splints to help him

64

00:08:39.799 --> 00:08:44.990

Pa6: be able to do these activities and academics kind of

65

00:08:46.180 --> 00:08:47.780

Pa6: helps in this.

66

00:08:48.630 --> 00:08:57.540

Pa6: and the fact that when he is practicing his core exercises or his or strengthening his neck muscles.

67

00:08:58.620 --> 00:09:08.450

Pa6: Academics keep keeps him engaged and keep them interested and doing these activities. Also, he uses an eye gaze computer

68

00:09:08.690 --> 00:09:26.810

Pa6: during academics and during his therapies. That helps him answer questions about how he's feeling, and whether he needs a break, and that this is part of his academic goals is to learn how to use his computer. So they all kind of work

69

00:09:26.860 --> 00:09:34.260

Pa6: together to further him in. In in all areas

70

00:09:35.080 --> 00:09:38.840

NM: you mentioned the eye gaze. Would you consider that physical activity for him?

71

00:09:38.920 --> 00:09:41.540

Pa6: Oh, definitely, it's.

72

00:09:42.710 --> 00:09:48.100

Pa6: It is a very strenuous activity.

73

00:09:49.770 --> 00:10:04.380

Pa6: He has to hold himself upright. He has to hold his head perfectly still and focus his eyes on a particular word on the screen. His dystonia is so severe that

74

00:10:04.380 --> 00:10:16.540

Pa6: it takes a lot of energy for him to hold himself still enough to be able to isolate a a word on the computer screen, and he often gets quite red in the face and sweaty.

75

00:10:16.570 --> 00:10:26.220

Pa6: and can get very frustrated when his Dystonia kicks in, and he's unable to maintain that upright posture to communicate.

76

00:10:26.660 --> 00:10:27.460

NM: Got it?

77

00:10:30.810 --> 00:10:34.850

NM: Come on, I'm. Just asking for myself now, how long has it been using the eye gaze? I think that was wonderful.

78

00:10:35.250 --> 00:10:41.390

Pa6: He's had the, eye gaze I think, for almost 3 years now he's getting very good with it.

79

00:10:46.130 --> 00:10:47.380

NM: And

80

00:10:47.420 --> 00:11:03.760

NM: does he do any of these activities alone, or is he typically in a group with other kids, or with, you know? And why? Why? Why, Why,

Pa6: He's he's as he's never alone unless he's sleeping. Because he does not like to sleep with other people near him.

81

00:11:03.880 --> 00:11:19.160

Pa6: He's never alone. He's always with someone for safety purposes. At school he's either with a therapist or a paraprofessional, or a teacher, or usually 2 of those at any given time

82

00:11:19.190 --> 00:11:30.020

Pa6: he's with myself or his dad or his nanny here at the house, but he's he is never alone, and never expected to do things on his own.

83

00:11:31.440 --> 00:11:40.610

Pa6: and mostly it's a safety reason, but also it's because his actions are so limited.

84

00:11:40.820 --> 00:11:48.600

Pa6: It's also a boredom issue for him as well. If you leave him in a room by himself without the ability to

85

00:11:49.830 --> 00:12:00.160

Pa6: to change his position. You know much. He will easily get bored, and also he could get himself into a compromising position.

86

00:12:02.750 --> 00:12:15.120

NM: Okay, all right. Next question before we go to the survey, how many times a week does your child participate in some of these activities, and how long and for how long. So I'm trying to tackle, maybe some endurance like. How long is he able to

87

00:12:15.560 --> 00:12:17.680

NM: you said

88

00:12:18.160 --> 00:12:28.310

Pa6: so as his IEP mandates that he gets 60 min sessions of physical therapy, occupational therapy and speech therapy

89

00:12:28.470 --> 00:12:41.110

Pa6: Monday through Friday, so that's 60 min. 5 days a week. He goes swimming not regularly, but I would say, once every 3 weeks or so.

90

00:12:41.280 --> 00:12:47.340

Pa6: He only goes horseback riding in the summer time, because it's the barn closes in the winter.

91

00:12:49.860 --> 00:12:56.870

Pa6: you know, using his, eye gaze as a daily function. Academics is a daily function.

92

00:12:58.990 --> 00:13:07.540

Pa6: a lot of this, a lot of the things that we've talked about are daily functions, but sporadic right.

93

00:13:07.780 --> 00:13:14.270

Pa6: you know. He could only sustain focus concentration, and

94

00:13:14.730 --> 00:13:25.440

Pa6: you know he gets fatigued very easily, so we can only sustain activities for a short amount of time unless he's specifically doing targeted therapy.

95

00:13:25.450 --> 00:13:37.850

Pa6: in which case they build in rest time and and stretching is not, it's it's more of a passive acti activity. Although it is exercise. It's passive exercise.

96

00:13:37.890 --> 00:13:44.650

Pa6: so you know he's not doing active exercise. The entire hour, but he is.

97

00:13:44.720 --> 00:13:47.450

Pa6: He has easily done an hour of

98

00:13:47.500 --> 00:13:59.430

Pa6: Pt. Ot. And speech every day of the week, for years now for years. so as far as his stamina, I would say, you know it's not compromised in in any way

99

00:13:59.470 --> 00:14:04.350

Pa6: by these you know therapies that he does in school.

100

00:14:04.970 --> 00:14:18.230

NM: and then the follow up to that is like how much assistance would you say he needs during like, in terms of prompting like, for some of the things that they may do an OT grab this setup, and then he's able to complete.

101

00:14:18.320 --> 00:14:25.280

NM: you know. Maybe the the access to the switch like, what kind of how, what things does he need part assistance, for.

102

00:14:25.720 --> 00:14:36.170

NM: compared to the other tasks which I would imagine eye gaze, he would need continual prompting, as he is continuing to learn

103

00:14:36.370 --> 00:14:51.350

Pa6: it's it's all day to day with ‘child’ depending on how his Dystonia is acting up. If he's having a good day and he's relaxed, he can do many activities with limited prompts, including his. Eye gaze computer

104

00:14:51.380 --> 00:14:56.010

Pa6: if he is having a bad day, and his dystonia is super bad.

105

00:14:56.090 --> 00:15:10.820

Pa6: and he is, and he easily slips into hyperflexion, or you know, extension. Then. you know, prompting will be a lot. But even with a lot of prompting

106

00:15:10.830 --> 00:15:13.740

Pa6: he may not be able to

107

00:15:13.760 --> 00:15:21.060

Pa6: complete the activity based on the fact that his body is working against him. So, after's one of the

108

00:15:21.390 --> 00:15:23.670

Pa6: it as you as one of the

109

00:15:24.410 --> 00:15:30.030

Pa6: it like one of these kids with cerebral palsy who really have no

110

00:15:30.130 --> 00:15:50.020

Pa6: like. Every day is different. It it. He can have a great day. He could have a bad day. He can have a middle of the road day he can have anywhere on the scale in between, and one day never looks like another day, and it all comes back to his dystonia and and the the loop that his mind gets stuck in.

111

00:15:50.020 --> 00:15:57.520

Pa6: you know, If he's, you can prompt him to reach for the ball, and he will be trying to reach for the ball. He just can't do it.

112

00:15:57.590 --> 00:16:03.600

Pa6: because his mind is stuck in a feedback loop where his muscles won't

113

00:16:03.710 --> 00:16:10.730

Pa6: respond to him. So it's not as if he doesn't understand the proper.

114

00:16:10.880 --> 00:16:23.640

Pa6: or He is actively not not paying attention or not trying. He is trying, and and it's obvious that he's trying. He just physically can't get the movement

115

00:16:23.760 --> 00:16:24.860

Pa6: to happen.

116

00:16:26.090 --> 00:16:26.890

NM: Got it?

117

00:16:27.740 --> 00:16:36.670

NM: And do you think, based on the things that he already has going on. Would you like him to participate in more of these activities or less? And why?

118

00:16:37.420 --> 00:16:39.150

Pa6: Definitely, More

119

00:16:39.160 --> 00:16:46.390

Pa6: Because I think exercise for ‘child’ is very important. He's non-ambulatory.

120

00:16:46.480 --> 00:16:54.320

Pa6: I believe that children are cerebral palsy who are nonambulatory. They have a

121

00:16:56.920 --> 00:17:01.120

Pa6: it's a they have a higher likelihood of

122

00:17:01.500 --> 00:17:14.750

Pa6: miss-shapened feet and leg bones, because they don't use them. You need to to stand to strengthen your bones so their bones can get very brittle. It's important to keep

123

00:17:14.760 --> 00:17:22.500

Pa6: these things as much as possible in the range of normality, so that the kids can remain as healthy as possible.

124

00:17:22.520 --> 00:17:35.490

Pa6: Also exercise engages the mind as much as the body. and you know, ‘child’ is a very bright child, and without these activities to keep his mind engaged.

125

00:17:35.560 --> 00:17:41.730

Pa6: You know we don't want his brain to atrophy any more than we want his body to atrophy.

126

00:17:42.180 --> 00:17:43.090

NM: That's good.

127

00:17:46.420 --> 00:17:49.690

NM: Absolutely all right. So now i'm going to share my screen.

128

00:17:49.760 --> 00:18:08.990

NM: So you the survey which I think you may already know. So i'm looking to ask parents about each question. Here again this survey was developed for children that actually had a a progressive dis disorder so initially for children that were having cancer, and that were regressing.

129

00:18:08.990 --> 00:18:11.120

Pa6: And so

130

00:18:11.210 --> 00:18:29.980

NM: the parents would answer for the child, and we're looking to see how parents really deem these questions. So i'm not going to ask you to. You know Rate your child. I'm actually going to ask you to grade the question like, how would you feel? This question is appropriate for a parent to answer of a child with Cp.

131

00:18:29.980 --> 00:18:33.050

NM: It was not the ambulatory like your

132

00:18:33.360 --> 00:18:37.550

NM: Okay? And then the so 0 is not appropriate at all.

133

00:18:37.590 --> 00:18:41.750

NM: 5 highly appropriate would be a good question. And then i'm gonna ask you to tell me why.

134

00:18:41.840 --> 00:18:42.800

NM: Okay?

135

00:18:42.830 --> 00:18:43.590

Pa6: Okay.

136

00:18:43.610 --> 00:18:57.110

NM: So the first question is, how many days did your child exercise a place so hard that his or her body got tired. How appropriate would it be for this population? 0? Not at all. 5 highly appropriate up to 5? And why?

137

00:19:02.640 --> 00:19:11.050

NM: I'll read the question again: how did your child exercise or play so hard that his or her body got tired?

138

00:19:11.610 --> 00:19:16.860

NM: I I i'm going to give that question a 0. Okay?

139

00:19:18.160 --> 00:19:25.460

Pa6: Because. being nonambulatory. Play. you know.

140

00:19:25.990 --> 00:19:34.480

Pa6: is very difficult, and their bodies are always tired because their bodies are working against them. So much so.

141

00:19:34.820 --> 00:19:39.090

you know it's it's a. It's kind of a trick question, because

142

00:19:39.380 --> 00:19:53.020

Pa6: children who are nonambulatory are always tired because of the hyper flexation, the hyper extension, the dytonia, You know this these things work muscles

143

00:19:53.080 --> 00:19:55.340

NM: very, very much.

144

00:19:55.360 --> 00:19:58.560

Pa6: and I think that's an inappropriate question, because

145

00:19:58.800 --> 00:20:05.110

Pa6: I think it's irrelevant to the population, and I think it's it's it it kind of

146

00:20:05.650 --> 00:20:14.620

Pa6: shows an ignorance on the part of the ask her, and not understanding how children with Cp's body works.

147

00:20:15.510 --> 00:20:16.610

NM: That's very help.

148

00:20:18.060 --> 00:20:21.470

NM: All right. Next question Number 2:

149

00:20:22.020 --> 00:20:26.780

NM: How many days did your child exercise really hard

150

00:20:27.470 --> 00:20:31.570

NM: for 10 min or more? How would you rate that one? And why?

151

00:20:35.560 --> 00:20:38.150

NM: So it does.

152

00:20:38.160 --> 00:20:46.080

Pa6: I mean, I I would give this question of 5, because a 10 min period is a

153

00:20:46.130 --> 00:20:56.260

Pa6: very relevant time period for children, and it is easily attainable by children with cerebral palsy to

154

00:20:56.360 --> 00:21:08.930

Pa6: play hard for a 10 min period before they need a rest. So I think this question would would be appropriate to ask.

155

00:21:08.990 --> 00:21:09.690

NM: Okay.

156

00:21:10.770 --> 00:21:12.540

NM: alright? Alright? Number 3.

157

00:21:13.650 --> 00:21:21.210

NM: How many days did your child exercise so much that he or she breathed hard. How would you rate that one?

158

00:21:29.540 --> 00:21:39.340

Pa6: I'm going to give this a one, and it's similar to the first question. I don't think it's as

159

00:21:39.820 --> 00:21:44.070

Pa6: kind of ignorant as the first question, but it

160

00:21:44.460 --> 00:21:47.260

Pa6: is bordering on it, because

161

00:21:48.060 --> 00:21:49.020

children

162

00:21:53.420 --> 00:21:55.690

Pa6: with cerebral palsy

163

00:21:57.380 --> 00:21:58.270

Pa6: can

164

00:21:58.600 --> 00:22:07.180

Pa6: breathe hard, not due to exercise, but just due to the exertion that it takes to actually just be in the world.

165

00:22:07.840 --> 00:22:25.370

Pa6: Not always, not all the time, but it can happen. And you know, I I think, to say that the only reason that a a child could breathe hard is because of exercise, I think is is is not really a a truthful statement.

166

00:22:26.360 --> 00:22:27.350

NM: Yeah.

167

00:22:31.370 --> 00:22:32.260

NM: I got it

168

00:22:32.490 --> 00:22:34.550

NM: all right. Number 4.

169

00:22:36.310 --> 00:22:43.100

NM: How many days. Was your child so physically active that he or she sweated. How would you rate that one?

170

00:22:46.210 --> 00:22:50.350

Pa6: I think that you know I would give this the 5,

171

00:22:50.840 --> 00:22:54.320

Pa6: because I think

172

00:22:55.370 --> 00:23:06.360

Pa6: it is relevant to being physically active. to sweat, because physical activity can also mean

173

00:23:08.690 --> 00:23:15.940

Pa6: how their body reacts. Sometimes, as your estonia and hyper extension is so bad he starts to sweat.

174

00:23:16.030 --> 00:23:21.370

Pa6: which is an activity. It's not exactly exercise, but it does

175

00:23:21.390 --> 00:23:27.250

Pa6: make him sweat. So you know, I I would say that this is a a relevant question.

176

00:23:27.500 --> 00:23:28.220

NM: Okay.

177

00:23:29.670 --> 00:23:38.240

NM: my number 5. How many days is your child exercise a place so hard that his or her muscles burned. How appropriate would that one be for you?

178

00:23:39.160 --> 00:23:50.490

Pa6: Well, for me this is in absolute 0, because, as your nonverbal and he could never tell us that the answer to that question.

179

00:23:51.320 --> 00:23:56.310

Pa6: So I would be highly offended by that question. And if a doctor asked asked me that question.

180

00:23:56.680 --> 00:23:57.350

NM: Yeah.

181

00:23:58.260 --> 00:24:06.890

NM: all right, Number 6. Thank you. That's good. How many days did your child exercise or play so hard that he or she felt tired

182

00:24:08.200 --> 00:24:10.340

again. I'm going to give this a

183

00:24:10.600 --> 00:24:20.970

Pa6: 0 because of the inability for him to tell me that I, as his parent. I'm very attuned to ‘child’, and I can guess

184

00:24:21.580 --> 00:24:32.190

Pa6: how he is doing, but I cannot say for 100% certain what he's dealing I can, I can guess, probably better than anyone in the world.

185

00:24:32.290 --> 00:24:36.690

Pa6: But I can't ask him and get a get an answer.

186

00:24:37.500 --> 00:24:38.370

NM: Got it

187

00:24:41.240 --> 00:24:47.270

NM: all right. Number 7. How many days was your child physically active for 10 min or more

188

00:24:47.760 --> 00:24:59.960

Pa6: I would give this a 5. I think it's relevant, I mean. Again, I think 10 min is a is an appropriate amount of time for a child with cerebral palsy to be active before needing a break

189

00:25:00.110 --> 00:25:17.210

Pa6: definitely as they get older, you know, I would say maybe not when he was 5 years old, but now that he's 12, almost 1310 min is definitely an appropriate amount of time. But as they get younger it would get more and more irrelevant

190

00:25:17.570 --> 00:25:24.850

Pa6: because younger kids just can't with cerebral palsy just can't sustain that amount of activity.

191

00:25:31.720 --> 00:25:37.180

NM: All right. Last, but not least. How many days your child run for 10 min or more.

192

00:25:37.210 --> 00:25:43.730

NM: How relevant would this be in this population.

193

00:25:45.370 --> 00:25:46.430

Pa6: and

194

00:25:46.580 --> 00:25:49.030

Pa6: just really insensitive?

195

00:25:55.030 --> 00:26:06.340

NM: All right. So we are near the end. I like to ask all my interviewees for their final words and thoughts, as we kind of wrap up about

196

00:26:06.350 --> 00:26:11.380

NM: physical activity for this population, anything that you would like to end with and share.

197

00:26:12.700 --> 00:26:17.630

Pa6: Yeah, I think you know, physical activity for

198

00:26:17.690 --> 00:26:28.050

Pa6: children who are nonambulatory with cerebral palsy is just as important as physical activity for quote unquote neurotypical, normal children who

199

00:26:28.080 --> 00:26:30.960

Pa6: have their entire bodily function.

200

00:26:31.040 --> 00:26:40.380

Pa6: For all the same reasons it's healthy. It gets them out in the world. It gets their muscles stretched.

201

00:26:40.960 --> 00:26:42.140

Pa6: and.

202

00:26:43.530 --> 00:26:51.990

Pa6: you know. having more activities available to children with herable palsy, I think, is also important.

203

00:26:52.120 --> 00:27:08.100

Pa6: You know there should be more teams that they can join more activities that they can do more dance classes, that they can go to more people who are willing to reach out and help them do these activities because they can get activities in school. But

204

00:27:08.230 --> 00:27:13.720

Pa6: you know, school and and getting out in the community are 2 different things, so you know.

205

00:27:13.740 --> 00:27:25.420

Pa6: taking as her to dance class, and having, you know, someone willing to help him stand and twirl around, and you know, raise his hands above his head with the music.

206

00:27:25.630 --> 00:27:29.410

Pa6: It. You know these are very important things like

207

00:27:29.600 --> 00:27:31.770

Pa6: playing, you know, baseball.

208

00:27:31.960 --> 00:27:33.850

Pa6: and just

209

00:27:33.970 --> 00:27:41.710

Pa6: anything that a quote on quote nor a typical child can do should be afforded to

210

00:27:41.810 --> 00:27:52.960

Pa6: children with disabilities; and the fact that we don't often have an avenue for that is it. I think it's irresponsible, and the

211

00:27:53.260 --> 00:27:55.920

Pa6: for society to allow that to happen.

212

00:27:56.570 --> 00:28:04.070

Pa6: and I think it's unconscionable that people don't don't think about it and Don't realize it and Don't include children with disabilities in these activities.

213

00:28:06.200 --> 00:28:07.080

NM: Okay.

214

00:28:09.180 --> 00:28:10.360

NM: very important.

215

00:28:10.880 --> 00:28:13.760

NM: Thank you so much, Pa6. Hold on.
