# Supplementary material for: Validity of PROMIS® Pediatric Physical Activity Parent Proxy Short Form Scale as a Physical Activity Measure for Children with Cerebral Palsy Who Are Non-Ambulatory
Source: Behav Sci (Basel). 2025 Jul 31;15(8):1042. doi: 10.3390/bs15081042 (PMC12382615; doi:10.3390/bs15081042)
Supplement: Supplementary file 1 [file behavsci-15-01042-s001.zip › Transcripts copy/PT transcripts - deidentified/PT20.docx]

WEBVTT

1

00:00:01.500 --> 00:00:23.839

NM: All right. Thank you so much for joining us today. We're going to be discuss physical activity in children with Cp. Who are not full time walkers. So I have my set quite a group of questions for you and follow up. So I asked you to answer them as you would basically your clinical expertise is you'll write a wrong answer. And again, thank you for your time. Second half. We'll look at a survey developed by the National institute of help.

2

00:00:23.850 --> 00:00:25.950

NM: and i'll ask you some questions pertaining to that.

3

00:00:26.030 --> 00:00:32.630

NM: So for our first question, how do you define physical activity for children with Cp. Who are not full-time walkers.

4

00:00:33.570 --> 00:00:46.720

PT20: Oh, that's a loaded one. I would say anything that requires increase, muscle involvement that can raise their heart rate or their respiratory rate. So that can be anything as simple as

5

00:00:46.980 --> 00:01:04.009

PT20: working on transitional movements, such as rolling on a mat to reaching for an object working on core balance, sitting balance, although we have to greater skills, such as assisted gait training and a weight supported Gait. Trainer

6

00:01:04.090 --> 00:01:09.320

PT20: all the way up to Bike riding, and again an adapted tricycle. So I would say anything that really

7

00:01:09.390 --> 00:01:14.120

PT20: works on muscle activation increases, heart rate increases respiratory rate.

8

00:01:16.140 --> 00:01:27.340

NM: Great. Thank you. The Department of Health defines physical activity as any activity that encompasses energy expanded in activation of skeletal muscle. Does this definition change your mind about how you define PA.

9

00:01:27.680 --> 00:01:31.159

PT20: I'm just gonna shut the door up as someone kind of peaked in. So hold on.

10

00:01:32.400 --> 00:01:34.200

PT20: Can you repeat that? I'm sorry.

11

00:01:35.720 --> 00:01:46.449

NM: Sure. The Department of help defines physical activity as any activity that encompasses energy expended, and activation of skeletal muscle. Does this definition change your mind about how you define PA.

12

00:01:46.920 --> 00:01:54.040

PT20: No I agree [NIH] with that energy, energy expenditure, and you can physiologically see that by increased heart rate increase respiratory rate. Yeah.

13

00:01:57.020 --> 00:02:01.629

NM: Great. How do you think physical activity differs from other types of fitness activities?

14

00:02:02.540 --> 00:02:22.039

PT20: I would say, because it When you think of fitness activities, you're thinking of doing a purposeful activity for the angle of being like cardiovascular training, strength, training, think of your typical going to the gym working out on a treadmill. bar bells weight training that kind of thing, physical activity, I think of more related to

15

00:02:22.050 --> 00:02:33.659

PT20: at least in this specific population, more mobility related activities, a daily living, so that is working on transfer training, sitting balance, reaching for functional activities.

16

00:02:34.010 --> 00:02:38.609

PT20: maneuvering in their wheelchair, whether that'd be manual or power.

17

00:02:39.190 --> 00:02:45.879

PT20: a more physical activity that way. But I also feel like you could do physical activity as working on strength training

18

00:02:46.250 --> 00:02:49.740

PT20: and cardiovascular endurance. So I feel like maybe

19

00:02:50.520 --> 00:02:53.699

PT20: physical activities, more of like an umbrella term. And then you can.

20

00:02:54.190 --> 00:02:56.960

PT20: you know, shoot off from there whether you're doing

21

00:02:57.450 --> 00:03:03.470

PT20: specifics, whether it's strength, training, cardiovascular training. Nerro-reed kind of that

22

00:03:04.840 --> 00:03:11.860

NM: great. Thank you. When do you witness your students participate in in physical activity during the school day.

23

00:03:13.970 --> 00:03:27.169

PT20: so they do have speech ot, pt, and a whole bunch of different specialties throughout the day, such as dance, movement, therapy, Rec, therapy, adapted PE. So I would say they're doing physical activity throughout all of those.

24

00:03:27.180 --> 00:03:50.999

PT20: even if they're just, you know, sitting in their chair, working on communication, seeing you using, an an eye gaze device. There has been some research out there that shows that this increased heart rate is associated with how eye gaze use and communication. So I would say, even that is considered physical activity in this patient population all the way up to you know what we do in Pt. Working on higher level gait training, mat, mobility skills transfer. So I would say, there's

25

00:03:51.350 --> 00:03:55.070

PT20: besides, maybe passively being transported between

26

00:03:55.920 --> 00:04:01.679

PT20: their schedules and their classes throughout the day. I would say that they are doing some sort of physical activity

27

00:04:02.090 --> 00:04:21.649

PT20: throughout the majority of the day, even though in the general population something may not be considered physical activity, such as like sitting in there like you or and I sitting at our desk and and writing down something that may not be considered physical activity, but asking someone GmfCs levels 4 and 5 to sit in their chair, their wheelchair, even though it has supported positioning aids

28

00:04:21.920 --> 00:04:37.149

PT20: But then ask them to use their communication device that is taxing for them. Whether that be working on postural control, head control, cervical rotation, eye gaze and visual fixation to me that is considered physical activity.

29

00:04:40.260 --> 00:04:41.190

NM: Thank you.

30

00:04:41.250 --> 00:04:42.210

NM: Number 2.

31

00:04:42.670 --> 00:04:53.309

NM: How do you measure physical activity, frequency, intensity, time and type? So i'm citing those thick principle here, and children with Cp. Who are not full time walkers.

32

00:04:55.110 --> 00:05:10.279

PT20: If in the moment we're able to access some methods such as like having a pulse ox available, we can take heart rate like you can do those general measures of bodily functions for pulse, respiratory rate, heart rate, all that stuff you could do that.

33

00:05:10.340 --> 00:05:12.389

PT20: But I think, looking at

34

00:05:12.430 --> 00:05:36.990

PT20: It's it's really hard, because it's all dependent on someone's personal endurance which completely can change, because, even though our kids may have that general diagnosis of Cp. Some of them may have been typically developing, but had a traumatic brain injury within the first 2 years, so they have a different skill set that they might have already developed. Some of them might have an underlying genetic condition. That may be progressive. So we're seeing difference in changes. So it's

35

00:05:37.000 --> 00:05:41.020

PT20: really hard to describe, because Cp. Is so generalized

36

00:05:41.030 --> 00:05:58.409

PT20: that it does make that more challenging. But I would say, looking at how long they can tolerate that activity in a certain piece of equipment. So time i'll measure distance, so say i'm measuring they can walk 100 feet. They can walk 50 feet, so distance would be a measure that I use a lot of times

37

00:05:58.530 --> 00:06:06.660

PT20: a lot of times. I'll say, how long can you hold this position? So say i'm working on sitting down so it's. Hold it for 10 s. Let's hold it for 15 s.

38

00:06:06.760 --> 00:06:08.160

I made

39

00:06:08.170 --> 00:06:27.570

PT20: graded off of how long they need their rest break for. So maybe on Monday I could do a gait training activity, and they only need to rest for maybe a minute. And before they begin stepping again compared to Friday. They might need a 5 min break in the hallway before we keep stepping, so that can be another way to measure how you know the amount of time that they need that rest in between.

40

00:06:28.700 --> 00:06:44.900

PT20: The only thing that I find challenging measuring is strength. Manual muscle Testing really is not accurate in this patient population. Whether that be because of they can't follow the commands because of, you know, some intellectual problems they might have. They may have limited range of motion. So we're really not getting an accurate

41

00:06:44.910 --> 00:06:55.660

PT20: manual muscle test response, because they can't even move their body actively through a full range because of joint contractures, so I struggle with, if grading

42

00:06:55.920 --> 00:06:56.950

PT20: strength

43

00:06:56.970 --> 00:07:00.480

PT20: but one way. I've kind of gone around, that is, by looking at

44

00:07:00.560 --> 00:07:24.580

PT20: the assistance I may give them, so it's more of like my intrarater or reliability with this is, you know, say i'm working on a sit to stand, transfer, and at the beginning of the school year. They're needing Max assist, and by the end of the school year they're meeting mod assist that lets me know that they're increasing that strength needed to do those to to stay in transfer. So it's a lot more subjective with this patient population.

45

00:07:28.660 --> 00:07:42.779

NM: Thank you. So you gave us a lot a good list of some activities? Do they need assistance to complete these activities, and during which activities will they need the assistance? And is it for a whole or just part of the task.

46

00:07:43.360 --> 00:08:02.039

PT20: Yeah. So almost all of our activities students will need assistance throughout the whole task. Whether that be verbal, queing, or physical assistance depends on the complexity of the task, and and you know whether there may be a GmfCs level. 4, 5 could also change that. So, for example.

47

00:08:02.050 --> 00:08:06.360

PT20: we do 2 outdoor power mobility groups to work on

48

00:08:06.370 --> 00:08:28.540

PT20: navigating the community, and one group is a little bit lower level, skill wise for power, mobility, and one is much more higher level, so the lower level one. I may need to use some hand over hand assistance with the joystick to, you know, for crossing the street, obviously significantly more verbal, queuing for safety awareness. Looking at, you know a red light versus a green light when it's safe to cross the street compared to that higher level

49

00:08:28.550 --> 00:08:37.220

PT20: group will only need verbal queuing for safety reasons, because we are in West Phil, you know, feel cut around traffic like crazy for for that. But

50

00:08:37.370 --> 00:08:42.269

PT20: that depends on the task with its you know, the level queuing there when it comes to

51

00:08:43.039 --> 00:08:47.330

PT20: more full body. Physical activity, such as gait training.

52

00:08:47.470 --> 00:08:50.040

PT20: Usually it requires

53

00:08:50.210 --> 00:09:09.509

PT20: physical assistance the entire time. Most of our kids will need maximum assistance just for that forward propulsion of the gait trainer. They may be able to do that stepping, but they're not getting that ground reaction force to really push off and move the gate trainer, so we'll be helping them that way. The same with steering. They may not have the ability to steer. To move the gait trainer.

54

00:09:09.520 --> 00:09:33.350

PT20: The same with the adapted bikes that you're going to need that assistance to. They may be able to help every once in a while, using maybe some gross extension t to help with that revolution, but they're going to need assistance to consistently pedal as well with steering. It's really hard, especially if you have any joint contractures that motor planning of trying to move, handle bars to steer like is complex, so they're going to need assistance with that and all of our transfers Our kids are.

55

00:09:33.620 --> 00:09:51.030

PT20: There's very few that can do independent transfers, and if they do, it's very close supervision, and usually some verbal queuing just for task sequencing like reminders of where I need to place my body, or or the sequence of of how to get, you know, a a seat, belt buckle undone so that can range from you know

56

00:09:51.160 --> 00:09:58.489

PT20: Max verbal queuing compared to some of our kids, our total dependence where we need to use a sling and a lift with them. So

57

00:09:58.810 --> 00:10:13.319

PT20: it's a little bit wider range. We try to do as many compensatory skills as possible to get them as independent. But usually I’m there i'm. Never stepping out of the Pt. Gym. And and tell you, kid, okay, can you get out of your chair and get on to the mat? It's always. I am right there with them

58

00:10:13.940 --> 00:10:15.550

PT20: long winded Answer.

59

00:10:15.710 --> 00:10:22.859

NM: That was great. Thank you. Do you think they should participate in more or less of each of these activities? And why

60

00:10:23.590 --> 00:10:30.120

PT20: I mean, i'd love more, the more that I can give them the opportunity to participate the better.

61

00:10:30.140 --> 00:10:34.250

PT20: But it's also looking at throughout their day. So I just got done with

62

00:10:34.420 --> 00:10:50.780

PT20: a little girl. We were working on the GMFM. With her, and it was nice to see some, some discrepancies from one year to another just because she has had some school uses progression. But she was really tired at the end of that, and typically I would have her drive to her back to her classroom.

63

00:10:51.010 --> 00:10:52.750

PT20: but I gave her the option. I was like

64

00:10:52.760 --> 00:11:17.559

PT20: you seem a little tight like. Do you want me to drive, or would you like to drive, and she selected that. I drive her so. It's tough, because I also don't want I could. I could have made her drive, but then I also have to consider her energy expenditure for the rest of the school day to make sure that she has enough energy to properly eat her lunch, so she's not aspirating. Make sure that she can participate in the rest of our educational programming. She does

65

00:11:17.570 --> 00:11:25.369

PT20: speak so i'm like. Is that going to then cause her to be exhausted, and she's not going to be able to have the breath control and diaphragmatic. You know

66

00:11:25.480 --> 00:11:34.099

PT20: strength and endurance for the rest of the day. So I would love to have kids contribute to as much as possible, because I do think it's so important to have them

67

00:11:34.110 --> 00:11:55.659

PT20: feel control of their environment, and have them feel that success of independence. But the same time i'm also thinking in my head all right if I have them for something in the morning. Am I going to want to exhaust them so much that they can't participate in the rest of their school day? So it's weighing those pros of cons of. I. I want them to get a well rounded day with all their activities without getting them exhausted just from Pt.

68

00:12:00.250 --> 00:12:02.169

NM: Thank you. Number 3.

69

00:12:02.360 --> 00:12:07.520

NM: Do you address promoting physical activity during physical therapy sessions?

70

00:12:08.540 --> 00:12:12.519

PT20: Yes, so wait. Can you explain that we see it again?

71

00:12:13.030 --> 00:12:19.239

NM: So do you address promoting physical activity during Pt. Sessions you've already gave and given some great example. So

72

00:12:19.560 --> 00:12:26.259

NM: yes, right. And then I I would go further and ask, how do you specifically do this in a in a session.

73

00:12:26.370 --> 00:12:27.820

NM: Excuse me.

74

00:12:28.090 --> 00:12:35.290

PT20: so we utilize so many pieces of equipment in here, and I think, being creative with the equipment available helps

75

00:12:35.340 --> 00:12:48.790

PT20: promote as much physical involvement as possible. So, for example, i'll use gait trainers in kind of an unconventional way, so may you think of it just as you know, supported stepping. I may then, instead

76

00:12:48.800 --> 00:13:10.579

PT20: have them use it as a trunk control, so I can get them sitting on a therapy ball, so I may put a a large therapy ball underneath them, but i'm using the trunk support, for example, like with a rifton pacer or a tram, and that way I can have my hands free and work on reaching, or I can do a little bit of trunk rotation while they're in there. So i'm using equipment not necessarily, and and

77

00:13:10.620 --> 00:13:15.690

PT20: the sense that it may have been designed for so that way they can continue with that that

78

00:13:15.880 --> 00:13:17.880

PT20: other movement that I may want them to do

79

00:13:18.040 --> 00:13:23.460

PT20: the same with some of the bikes and other equipment we're trying to incorporate more

80

00:13:23.470 --> 00:13:40.999

PT20: communication based stuff as well. So i'll always have like a big Mac switch with me, or a step by step, and I can record things so that they're involved in their movements. So, For example, if I have a student on a bike, I might record more bike, and then we'll stop, or on, you know, stopping, stop and go

81

00:13:41.010 --> 00:13:59.190

PT20: working on that language, and then, once we are stopped, they'll have to then use upper extremity, strength and range of motion to reach for a switch, to say more bike to reinstate that activity. So it's also using, like multimodal stuff, with equipment and communication, working on on their verbal language or their

82

00:13:59.330 --> 00:14:05.430

PT20: communication. Language the receptive and expressive language with that as well. It's.

83

00:14:05.750 --> 00:14:19.020

PT20: So I try to get as much movement as possible out of them, even if it's for kids like the GMFM. I keep going back to the GmFM because I just did it so it's kind of fresh in my head, but I like that. I can give partial credit, because it's really nice. And not

84

00:14:19.340 --> 00:14:20.480

PT20: only

85

00:14:20.490 --> 00:14:47.600

PT20: am I getting an objective score, but I feel like I can then realize. Oh, you know, what they were able to initiate some of this hip flexion when I asked on on this specific thing, maybe I can then look at this in a different area and use it towards. Oh, they have hip flexion. Well, maybe I can put a switch down at the top of their knee, and we can work on power, mobility going forward, using that hip election if they might not be able to access a joystick, or if they might not have the head control to access a header, a to move forward.

86

00:14:47.790 --> 00:14:56.909

PT20: So, looking at even the slightest movements that they can do, and trying to maximize that so that we can get independence in any way possible.

87

00:15:00.920 --> 00:15:08.550

NM: That's great. So what components physical activity do you address even in those examples? Let me give you some.

88

00:15:08.790 --> 00:15:18.890

NM: Let me give you some examples of components. Are you addressing most cardiovascular endurance, muscle, activation, energy, expenditure, mobility? I can go on. But what are some of the areas you feel like you are

89

00:15:19.090 --> 00:15:26.510

NM: really focusing on in terms of physical activity. What components of that are you using on in your session?

90

00:15:26.610 --> 00:15:40.100

PT20: for me, I really want to get as much self-initiated movement as possible. So a lot of the exercises kind of can be passive at times, whether that be passive range emotion. A lot of times in a gait trainer I will do passive stepping.

91

00:15:40.110 --> 00:16:00.450

PT20: They might be in a swing for sensory feedback, but that's me passively swinging them if they're in a bike, and i'm pushing them, and they're not assisting at all. That's passive range of motion. So for me i'm trying to look at. What can I get? Even just like the slightest bit of self initiated movement so that they could do so. Self initiated movement for me is a big one.

92

00:16:00.460 --> 00:16:13.810

PT20: Cardiovascular training is is important to me, because I feel like our kids have limited access to that, and it's tough because I feel like it's quickly. They reach a higher heart rate more quickly than a typical population, because

93

00:16:14.010 --> 00:16:15.619

PT20: they don't have

94

00:16:16.920 --> 00:16:33.830

PT20: as much experience with cardiovascular training. Like the smaller they don't. They spend more energy, more quickly. So working on on trying to increase endurance with activities is a big one. Whether that be muscular endurance or cardiovascular endurance. Postural controls. A big one core

95

00:16:33.840 --> 00:16:50.740

PT20: or strengthening is a huge one that I focus on, because our kids are positioned, and either a manual wheelchair or a power wheelchair with pretty significant positioning needs throughout the day. So i'm talking. We either will do. All of our kids have laterals on. Some have pelvic harnesses, seat belts. You know we have different types of headrests.

96

00:16:50.750 --> 00:16:59.149

PT20: So when I get them out on a mat or a piece of equipment working on engaging our core muscles and our cervical muscles and and head control and trunk control

97

00:16:59.420 --> 00:17:11.259

PT20: So that way. They're not expending as much energy when they're sitting in their wheelchair. They can participate in their educational programming, and you work on a lot of strengthening on you for the kids who are able to

98

00:17:11.270 --> 00:17:29.190

PT20: and understand the directions I will use. Thera-bands at times just to work on some basic strengthening for that, and then i'll use a lot of close chain weight bearing activities for strengthening. So, for example, working on our sit to stand transfers kind of making that into like a modified

99

00:17:29.200 --> 00:17:39.099

PT20: squat, or I've done wall…, wall slides with kids where I'm really giving them a lot of support at their pelvis and their trunk. But it's still that closed chain to activity.

100

00:17:40.240 --> 00:18:05.530

PT20: I've done things where they're on prone on a scooter or prone on a platform swing where they have to push off the floor. So you're getting that scapular retraction, but they're also getting tricep involvement. So a lot of closed chain activities for kids. I feel like open chain just doesn't work very well. They don't have the stability for it. A lot of them don't have the motor planning, or just the the cognition to kind of understand what that i'm asking of them

101

00:18:08.150 --> 00:18:09.410

NM: That's great.

102

00:18:09.480 --> 00:18:10.499

NM: All right.

103

00:18:10.650 --> 00:18:17.749

NM: You focus on pretty much everything. So is there ever a reason why you may not want to focus on any of those components for physical activity.

104

00:18:17.810 --> 00:18:25.810

PT20: So again, if they have something later on in the day that I know that they really need to have their energy for or

105

00:18:26.140 --> 00:18:36.820

PT20: medical reasons say, if they recently came back from a certain surgery or long prolonged hospitalization, or a respiratory compromise where they

106

00:18:36.830 --> 00:18:52.260

PT20: they are really struggling with certain things. It's it's really based off of how they're feeling when they come in. Some of our kids can be really affected by a poor night's sleep. Some of them have seizure activity when they're transitioning from sleep to wake.

107

00:18:52.270 --> 00:18:57.820

PT20: Even the slightest med change can really affect how how they're feeling that day so

108

00:18:57.990 --> 00:19:08.009

PT20: really gauging how they present to me that day dictates a lot of times what I do. We kind of joke around at my school like you have a plan ABCDEFG Like the whole thing, because

109

00:19:08.020 --> 00:19:27.289

PT20: our kids do really change day to day significantly on their physical presentation that you want to accommodate, for you know any fatigue that they may have any increase in spasticity we may see with, you know, if they're getting over an illness. So that would be reasons why i'm not doing something. Another reason, maybe, if they're having any

110

00:19:27.300 --> 00:19:42.190

PT20: issues with orthotiv=cs, so a lot of our when the majority of our kids will wear mathos or have body jackets, TlSO And if for some reason that is out for a repair, if they need a new one, or if they're getting some like a little bit of redness or skin compromise that may affect

111

00:19:42.200 --> 00:19:49.440

PT20: an activity, I do, because oh, they don't have their Mathos today, or oh, they don't have their body jacket today, so that could affect why I would or would not do something

112

00:19:50.460 --> 00:19:59.689

NM: great. Thank you all right. Last question before the survey. Do you address promoting physical activity that occurs outside of your physical therapy session?

113

00:20:01.010 --> 00:20:10.569

PT20: We try to, though I feel like that is a lot more challenging, especially with this patient population. Our families are already inundated with doing so much.

114

00:20:10.580 --> 00:20:22.960

PT20: The care surrounding our kids is extremely involved, and not every one of our parents has at home nursing. So it's tough for for parents to even find the time to do physical activity. So

115

00:20:23.090 --> 00:20:36.270

PT20: a lot of times we'll get equipment for the home that they can be positioned, such as standers standers are a really big one on, activity Chairs will order for the home date trainers we will order if they're up for the same with

116

00:20:36.580 --> 00:20:37.550

PT20: with

117

00:20:37.900 --> 00:20:39.120

PT20: adapted

118

00:20:39.890 --> 00:20:58.890

PT20: tricycles. If we have some alternative funding and I do make home exercise programs as deemed appropriate, and a lot of times i'll be more parent initiated because they might be like oh, you know, we have nursing at this hour, and they have some availability. Can you come up with a range of motion exercise, or we got this

119

00:20:58.900 --> 00:21:08.180

PT20: You know, I might be related to a sibling like, hey, If siblings playing soccer now like, do you have any activities that maybe like this, you know so and so can get involved with, because their siblings playing this.

120

00:21:08.210 --> 00:21:08.930

PT20: But

121

00:21:09.150 --> 00:21:14.770

PT20: and we don't do a ton of outreach to the home in that sense, because the the parents don't have a ton of

122

00:21:14.940 --> 00:21:16.470

PT20: time, and

123

00:21:16.490 --> 00:21:21.700

PT20: a lot of times parents will be like. Oh, my gosh! They were so exhausted when they got home, because they do have a long commute on the bus.

124

00:21:28.210 --> 00:21:46.639

PT20: traffic is really terrible. you know, and then they're getting up early, so they really have a 2 h commute plus a full day, plus when they get home. They need all their personal care, medication, all that stuff, whether it be 2 feedings as well it it. It really eats away with available time for family. So it's a challenge I would love ideally. It would love for all my kids to do physical activity at home.

125

00:21:46.650 --> 00:21:51.200

PT20: But I understand the practicality for families just may not be there

126

00:21:54.020 --> 00:22:00.079

NM: right. Have you recommended any community programs or events to your students to help increase PA?

127

00:22:01.770 --> 00:22:17.099

PT20: So I that's not necessarily my department's social work. It does a lot with that as well as recreational therapy that. But there are programs in our area. So, for example, we have an alumni who is a part of a power

128

00:22:17.110 --> 00:22:22.750

PT20: wheelchair hockey team that's sponsored by the Philadelphia flyers, alumni, and

129

00:22:22.990 --> 00:22:25.690

PT20: so like they'll they do that

130

00:22:25.750 --> 00:22:30.660

PT20: there is one student I have is involved in a adaptive

131

00:22:30.870 --> 00:22:36.950

PT20: basketball league. I have another student who is in part of an adaptive cheerleading squad. So a lot of it is more

132

00:22:37.140 --> 00:22:40.749

PT20: parent-based by like what they see to find

133

00:22:40.760 --> 00:23:07.489

PT20: outside of school as well as when we have their IEPs. We do discuss you know activities, especially the transition age. You know what's in the area, post 21 that they might want to be involved in. Whether that be, you know therapeutic hourse back riding, or you know, therapeutic swimming, or or anything like that, and then we try to assist them. Usually our social work department tries to system with getting them in contact with local organizations or things in the area.

134

00:23:07.500 --> 00:23:09.329

PT20: and we do have on

135

00:23:09.570 --> 00:23:17.739

PT20: staff doesn't see this, but they we do have a a Parent Facebook group where I know that they discuss. You know, things available in that area, and social work

136

00:23:17.950 --> 00:23:21.759

PT20: has been involved with creating some organization. So we do have like a

137

00:23:21.840 --> 00:23:30.829

PT20: a Girl Scout troop here, as well as a sibling shop, where they'll have, like the siblings, come in, and I know that may not be physically act related, but

138

00:23:31.260 --> 00:23:36.430

no way to yeah, I take that back. It is physically related, because if they're Selling Girl Scout Cookies and they have to reach without money.

139

00:23:36.520 --> 00:23:49.300

PT20: They're They're using their arms and activating their muscles that way. So I mean for our kids. We really can't count physical activity as just such small movements that you and I may take for granted as physical activity. But

140

00:23:49.700 --> 00:23:56.829

PT20: I wish I had more involvement in outside sports and activities, but usually social work will will assist when needed with the parents.

141

00:23:58.110 --> 00:24:04.229

NM: That's great, thank you. And you kind of cover this. But my last prompt was, what type of equipment.

142

00:24:04.250 --> 00:24:08.059

NM: Have you recommended to help improve home and or community engagement?

143

00:24:08.230 --> 00:24:14.270

NM: So I I believe you had already said the gait trainer standers anything else?

144

00:24:14.610 --> 00:24:18.250

PT20: Yeah, gait trainers, standards.

145

00:24:18.380 --> 00:24:20.430

PT20: activity chairs.

146

00:24:21.690 --> 00:24:26.040

PT20: We do run a seating clinic out of our school, so we order all of

147

00:24:27.130 --> 00:24:33.650

PT20: as if the parents choose to. We will order them power wheelchair, manual wheelchair as appropriate.

148

00:24:33.780 --> 00:24:41.600

PT20: We have very good repore with our orthotists in the area, so we will get them like their their mafos body jackets, you know.

149

00:24:41.700 --> 00:24:43.080

PT20: swashes

150

00:24:43.730 --> 00:24:46.950

PT20: any cervical collars they may need to anything like that.

151

00:24:47.140 --> 00:24:55.129

PT20: for at home ot really assist with, you know, bathing and hygiene chairs, bath, chairs, toileting chairs.

152

00:24:55.200 --> 00:25:01.570

PT20: other positioning aids that we have gotten versa -Form pillows

153

00:25:01.620 --> 00:25:05.020

PT20: in the past, tumble form

154

00:25:05.780 --> 00:25:12.310

PT20: floor, sitters on special tomato floor sitters side lyers like sidelying plinthes

155

00:25:14.590 --> 00:25:23.999

PT20: anything that insurance can cover, that we can justify as a medical need in the home that the parents are expressing. They need. We try to help with lift lifts and slings

156

00:25:24.080 --> 00:25:28.830

PT20: In the State of Pennsylvania. Medicare is now covering

157

00:25:30.000 --> 00:25:34.950

PT20: ceiling lifts and stair glides, so we're, starting to help with ordering that

158

00:25:35.730 --> 00:25:43.779

PT20: we used to order beds, but we got away from that because it was really hard for us to follow up with how they're tolerating that.

159

00:25:44.030 --> 00:25:57.449

PT20: So we got away from that. But whatever we can do in the home to try to make them as an as possible, and if we can't, I've no I've referred parents to Amazon. Bed bath and beyond being like, hey, this is the size wedge that they're on at school like this may be beneficial. You can look in.

160

00:25:57.520 --> 00:26:09.559

PT20: you know, for a cheaper option, because the second you put special needs stamp on something. The price goes through the roof. So if I can find alternatives that are not special needs, but can be used in a way that's therapeutic at home. I will always go that route

161

00:26:13.500 --> 00:26:17.710

NM: all right. That's great. I'm gonna share my screen. So you can look at the survey.

162

00:26:17.960 --> 00:26:19.220

NM: And

163

00:26:27.110 --> 00:26:35.710

NM: so this is called the promised parent, Active parent proxy, physical activity, skill. And again it was created for

164

00:26:35.950 --> 00:26:45.170

NM: children would have been going some kind of medical treatment on one typically developing or regressing. And so

165

00:26:45.480 --> 00:27:03.180

NM: it has been successfully used in many different populations. However, i'm. Looking to see how pt's first, and then i'm going to ask parents next how they believe this is valid in this population to specifically measure physical activity, intensity, and the kiddos that we're talking about. Okay, so i'll let you look at it for a second, and I'm going to ask you

166

00:27:03.660 --> 00:27:15.080

NM: to rate each question. So again, a caregiver would answer these questions about the activity in the past the previous week alright, alright, so based on hold on a second

167

00:27:19.560 --> 00:27:27.119

NM: based on your clinical expertise. I'm going to ask you to rate each question on a scale from 0, it not being related at all

168

00:27:27.330 --> 00:27:42.589

NM: up to 5. It's a highly appropriate question for this population, as it relates to physical activity, intensity, and then i'm gonna ask you Why? Okay? So the first question start here is, how many days is your child exercise or play so hard that his or her body got tired?

169

00:27:42.600 --> 00:27:56.049

NM: How appropriate would you believe this question is, as it relates to measuring physical activity, intensity, and children with with a Cp functioning at levels 4 and 5 0, not related, 5 highly appropriate and anywhere between

170

00:27:56.540 --> 00:27:57.349

and one

171

00:27:57.690 --> 00:27:58.520

PT20: Hmm.

172

00:27:58.810 --> 00:28:01.540

PT20: For the first one, I would probably say, like a 2.

173

00:28:01.730 --> 00:28:03.330

NM: Okay, I want.

174

00:28:03.800 --> 00:28:07.100

PT20: I feel like exercise or play

175

00:28:08.260 --> 00:28:19.239

PT20: may not be applicable applicable to our population like if I was a parent, I'm like well, my kids not really exercising like in the traditional sense that a parent may interpreting exercise.

176

00:28:20.440 --> 00:28:24.299

PT20: So that's what I would think of that. I do like

177

00:28:24.420 --> 00:28:41.490

PT20: about getting tired, because I do think that that affects a lot. What our kids can do, and we talk so much about energy, expenditure and energy conservation. I do like the keyword of tired. I just don't know if exercise or play so hard would be the right one. More, I would like to say, rather like.

178

00:28:41.830 --> 00:28:47.270

PT20: did your child move or assist with movement so much that they got tired.

179

00:28:47.880 --> 00:28:48.590

NM: Okay.

180

00:28:48.910 --> 00:28:50.670

NM: that's great feedback. Thank you.

181

00:28:50.950 --> 00:28:52.689

This is

182

00:28:53.600 --> 00:28:54.860

NM: number 2.

183

00:28:55.280 --> 00:29:03.629

NM: How many days is your child exercise really hard for 10 min or more? 0 not appropriate at all. 5 highly appropriate. How would you Re Graded? And why?

184

00:29:06.750 --> 00:29:08.700

PT20: Hmm. Probably like one.

185

00:29:09.480 --> 00:29:11.879

PT20: Just because, again, the exercise component

186

00:29:13.730 --> 00:29:18.299

PT20: Again, we're not interpreting exercise the same way that you would for a typical child.

187

00:29:19.160 --> 00:29:21.869

PT20: And it's hard to say like what is really hard

188

00:29:22.050 --> 00:29:25.129

PT20: like, really hard for a typical kid. I'd be like, okay. They're gonna.

189

00:29:25.840 --> 00:29:31.380

PT20: you know, like I remember running a mile in elementary school, and they timed you like that would be for me

190

00:29:31.420 --> 00:29:34.560

PT20: a hard exercise compared to our kids.

191

00:29:34.870 --> 00:29:36.439

PT20: a hard exercise.

192

00:29:36.760 --> 00:29:37.930

PT20: maybe.

193

00:29:38.280 --> 00:29:40.939

PT20: propelling their wheelchair in their home.

194

00:29:41.220 --> 00:29:47.319

PT20: So I guess just the wording like I like the idea of of objectively capturing what they're doing.

195

00:29:47.400 --> 00:29:50.630

PT20: But exercise to me is it's the wording of exercise

196

00:29:53.490 --> 00:29:55.319

NM: Great, Thank you. Number 3.

197

00:29:55.420 --> 00:30:00.080

NM: How many days did your child exercise so much that he or she breathe hard.

198

00:30:00.560 --> 00:30:06.240

NM: How would you rate that 0 not related at all up to 5, how to appropriate, or anywhere in between? And why

199

00:30:07.840 --> 00:30:19.809

PT20: I would say that a 3 or 4, I think the breathing hard is a really good way for parents to easily assess. If if their child is expending more energy. Again, it's just that exercise word. But actually, I I like the breathing hard part.

200

00:30:22.910 --> 00:30:24.940

NM: Okay, Final answer. Give me a number.

201

00:30:28.200 --> 00:30:29.770

PT20: Let's go with 4.

202

00:30:29.800 --> 00:30:30.510

NM: Okay.

203

00:30:37.460 --> 00:30:38.680

NM: Number 4,

204

00:30:40.140 --> 00:30:41.430

many days.

205

00:30:41.470 --> 00:30:49.729

NM: Your child so physically active, and he or she sweated 0, not related at all. 5 highly appropriate, and or somewhere between. And one.

206

00:30:52.020 --> 00:30:57.409

PT20: I give this one a 3, because I do think it's an easy physiological response that a parent can look at

207

00:30:57.430 --> 00:31:12.489

PT20: what's challenging with our kids is. They have issues with temperature regulation, and some of our kids will sweat just sitting there because they are having trouble regulating their temperature. But I do like that. It says physical, physically active over exercise.

208

00:31:14.690 --> 00:31:17.519

PT20: Right? You gave me a 3. I just wanna make sure that's right.

209

00:31:23.040 --> 00:31:25.350

NM: and Number 5 doing the right one.

210

00:31:25.650 --> 00:31:31.950

NM: How many days is your child exercise or play so hard that his or her muscles burn? How would you rate that 0 or not?

211

00:31:32.010 --> 00:31:35.519

NM: I hope you know, up to 5, and then it will be

212

00:31:36.230 --> 00:31:39.409

PT20: Hmm. Maybe like a one. I don't think our kids

213

00:31:40.260 --> 00:31:42.240

PT20: a lot of our kids cannot discern

214

00:31:42.470 --> 00:31:58.049

PT20: muscle burn. I don't think that they have the language for that. I don't think that they can express to us what they may be feeling when their muscles are fatigue and getting that burn some may be able to. I think I can think of maybe one or 2 off the top of my head at my school. They can.

215

00:31:58.120 --> 00:31:58.900

PT20: But

216

00:31:58.950 --> 00:32:03.050

PT20: some of our kids who are Gmf: Cs 4 and 5, I mean, we're working on

217

00:32:03.200 --> 00:32:04.530

PT20: trying to discern

218

00:32:05.440 --> 00:32:12.519

PT20: the difference between my head herding and my foot herding, so I don't think muscle burn would be something that they can differentiate something about one.

219

00:32:15.720 --> 00:32:18.510

NM: Thank you for your feedback. It's great Number 6.

220

00:32:18.860 --> 00:32:23.409

NM: How many days is your child exercise a place so hard that he or she felt tired?

221

00:32:26.220 --> 00:32:28.689

PT20: I think that could be a 4,

222

00:32:28.910 --> 00:32:36.160

PT20: because again the tired, I think kids can express when they feel tired. I think that's something that you can see when they're tired

223

00:32:36.290 --> 00:32:42.900

PT20: that a parent can observe their their physical reactions, and then that they're more fatigued. Again, it's that it's just the exercise word

224

00:32:43.910 --> 00:32:46.230

PT20: exercise and play. You know that freeze

225

00:32:53.010 --> 00:32:54.390

NM: number 7.

226

00:32:54.710 --> 00:33:03.559

NM: How many days it was your child physically active for 10 min or more? How would you rate this question, and why 0 not related 5 to highly applicable.

227

00:33:03.610 --> 00:33:05.110

NM: or anywhere in between.

228

00:33:06.560 --> 00:33:09.330

PT20: I think it's 4. I I would give that a 4. I think that

229

00:33:09.500 --> 00:33:22.400

PT20: 10 min is an easy thing a parent can look at. I think that the parameters just need to be explained to the parents defining physical activity. What that means for this patient population? And is it for 10 min

230

00:33:22.480 --> 00:33:32.640

PT20: in one consecutive setting, or can they be, you know, 2 min here? Oh, you know what there was 5 min there like. That would be another thing to is it all at once or not?

231

00:33:35.160 --> 00:33:37.139

NM: That's great. And number 8.

232

00:33:37.680 --> 00:33:44.919

NM: How many days is your child run for 10 min or more 0 not appropriate at all. 5 highly appropriate, and or or somewhere between. And why

233

00:33:45.380 --> 00:33:46.150

PT20: 0,

234

00:33:47.380 --> 00:33:50.579

PT20: that's Yep, that run not working.

235

00:33:51.650 --> 00:33:53.930

PT20: not appropriate for our patient population

236

00:33:57.710 --> 00:34:07.420

NM: all right. That's great. So I've been asking every Pt. If they have any final faults or last birds that they like to share about physical activities. Population as we close up.

237

00:34:08.449 --> 00:34:22.220

PT20: No, I think the work that you're doing is awesome. I look forward to seeing this because there is very little research that is applicable to Gmf. Cs. And very little outcome measures that I feel capture the

238

00:34:22.440 --> 00:34:31.589

PT20: small movements that they do do, and I think it's always good to have parent feedback, because I think that is one thing that is lacking

239

00:34:31.600 --> 00:34:50.449

PT20: sometimes between the plan of care, like we obviously do what we do in the Pt. Gem, but I think maybe some carry over at home, and maybe some communication between the therapist and the parent can be improved upon. So I think this would be actually a really nice addition to have when i'm creating a treatment plan.

240

00:34:54.000 --> 00:35:00.239

NM: I just want to thank you for your time. I'm going to stop the recording. Hold on 1 s. Thank you so much. The interview is now over.
