# Supplementary material for: Validity of PROMIS® Pediatric Physical Activity Parent Proxy Short Form Scale as a Physical Activity Measure for Children with Cerebral Palsy Who Are Non-Ambulatory
Source: Behav Sci (Basel). 2025 Jul 31;15(8):1042. doi: 10.3390/bs15081042 (PMC12382615; doi:10.3390/bs15081042)
Supplement: Supplementary file 1 [file behavsci-15-01042-s001.zip › Transcripts copy/PT transcripts - deidentified/PT6.docx]

WEBVTT

1

00:00:02.009 --> 00:00:09.360

NM: Good morning. Thank you, Dr.PT6, for joining us today. So we're going to begin our interview about

2

00:00:09.450 --> 00:00:15.700

NM: physical activity in children with Cp. They are not inventory. So I have a few questions, and then a few prompts to follow us up.

3

00:00:15.850 --> 00:00:22.380

NM: So the first question is, how do you define physical activity for children with Cp. Who are not full-time walkers.

4

00:00:24.890 --> 00:00:30.730

PT6: Well, it could be a range of things one could be supported sitting rolling

5

00:00:30.920 --> 00:00:33.080

PT6: even just positioning

6

00:00:33.200 --> 00:00:39.410

PT6: into different positions that they're not used to. It could be physical activity for them breathing, using different muscles

7

00:00:41.660 --> 00:00:43.630

PT6: or going on a stander as well.

8

00:00:43.740 --> 00:00:44.830

NM: Okay, Thank you.

9

00:00:44.940 --> 00:00:56.330

NM: a follow up to that is the department of Health defines physical activity as any activity that encompasses energy expended, and activation of skeletal muscle.

10

00:00:56.480 --> 00:01:00.830

NM: Does this definition change your mind about how you define physical activity?

11

00:01:02.970 --> 00:01:16.809

PT6: not really. I support like I think there maybe some of the kids are not actively voluntarily activating the skeletal muscle, but it's being activated by being put in different position.

12

00:01:17.550 --> 00:01:20.139

PT6: for example, breathing muscles.

13

00:01:20.490 --> 00:01:24.649

PT6: one doesn't have to think about using them for that, for that to be used

14

00:01:24.800 --> 00:01:28.070

PT6: that definition, I think, would apply.

15

00:01:30.020 --> 00:01:35.470

NM: And then, how do you think physical activity differs from other types of fitness activities.

16

00:01:40.780 --> 00:01:43.339

PT6: Well, that's a good question.

17

00:01:43.540 --> 00:01:47.969

PT6: I guess that really depends on your definition of fitness. Activities fitness. Kind of

18

00:01:48.320 --> 00:01:54.059

PT6: implies that you're actively, voluntarily trying to

19

00:01:54.200 --> 00:02:02.179

PT6: make your health better, whereas physical activity is just breath. It could be just breathing. It could be sitting. It could be looking.

20

00:02:03.430 --> 00:02:05.089

PT6: using your visual

21

00:02:05.210 --> 00:02:11.450

PT6: system so, and their ocular motor muscles. So I guess that would be the difference to me.

22

00:02:13.990 --> 00:02:19.639

NM: Thank you. And the last follow up, for this question is, when do you witness your students participate most

23

00:02:19.690 --> 00:02:22.570

NM: and physical activity during the school day?

24

00:02:23.560 --> 00:02:30.159

PT6: I don't actually see them during their school day. They I sometimes do if I go into their classroom, because I work in a facility where.

25

00:02:30.620 --> 00:02:41.589

PT6: like i'm their therapist upstairs in their home, and then they also get physical therapy at school downstairs. so sometimes I see them there. But, we do a lot of

26

00:02:42.100 --> 00:02:47.950

PT6: activities and group sessions that probably mimic similar school experiences, and

27

00:02:47.980 --> 00:02:51.749

PT6: I would say you definitely get the most from like a music group

28

00:02:51.770 --> 00:02:56.359

PT6: or any kind of group where it's lively around them, and people are laughing and

29

00:02:56.400 --> 00:02:57.910

PT6: interacting.

30

00:02:59.470 --> 00:03:02.010

PT6: You see a lot of smiling and laughing. And

31

00:03:04.430 --> 00:03:11.040

NM: And so would you consider that just the engagement of smiling and laughing part of their physical activity during the the the school day.

32

00:03:11.320 --> 00:03:13.050

PT6: I would. Yeah.

33

00:03:13.880 --> 00:03:16.030

NM: Okay, All right, Question Number 2.

34

00:03:16.270 --> 00:03:31.139

NM: How do you measure physical activity, frequency, intensity, time and type, and children with Cp. Who are not full time walkers. So that's like the fit principle so really trying to figure out how we could. How how would you measure that kind of looking at

35

00:03:31.920 --> 00:03:32.990

NM: the

36

00:03:33.030 --> 00:03:37.350

NM: defined stipulations of frequency, intensity, time and type in these kids?

37

00:03:39.090 --> 00:03:48.569

PT6: Well, most of the kids I work with are on pulse oximetry, so they can monitor their heart rate and their O2 saturation.

38

00:03:48.680 --> 00:03:53.110

PT6: you can. Now, if they're working a little harder based on their heartrate increase.

39

00:03:55.480 --> 00:03:57.160

PT6: I would say.

40

00:03:57.670 --> 00:03:58.900

PT6: Also.

41

00:03:59.100 --> 00:04:04.060

PT6: we just do it by the stander of like how many times a week they go on a stander.

42

00:04:04.530 --> 00:04:07.209

PT6: and how long they can tolerate the stander.

43

00:04:09.770 --> 00:04:13.120

PT6: or even being in a prone position or something like that

44

00:04:18.709 --> 00:04:36.769

NM: That's all great. Thank you. Do they need assistance to complete these activities. And you already mentioned Standard and some other activities. So if you have any additional activities, what they need, extra assistance, assistance, and do they need the assistance for the whole task, or just part of the task?

45

00:04:37.740 --> 00:04:39.859

PT6: I would say. Yes.

46

00:04:39.930 --> 00:04:44.050

PT6: So, children, I work with mostly need assistance.

47

00:04:44.780 --> 00:04:48.530

PT6: And not always the whole test, but a lot of times such as the whole task.

48

00:04:48.650 --> 00:04:58.950

PT6: But sometimes for rolling they just need to be put in a position where they can, you know, use the muscles they have to complete rolling, but usually they'll need some assistance.

49

00:05:03.000 --> 00:05:08.609

NM: And do you think they should participate more or less in each of these activities? And why?

50

00:05:10.350 --> 00:05:13.020

PT6: I think they should

51

00:05:13.610 --> 00:05:21.879

PT6: they should participate in these activities every day on part of their daily life, because it's what they can do, and you want to optimize whatever they can do

52

00:05:22.250 --> 00:05:23.870

PT6: and their quality of life.

53

00:05:29.930 --> 00:05:43.019

PT6: Can I add to that and say, I don't think it has to always be a physical therapist who does this like part of our role, is to train other staff like school staff and other caregivers, to also help them get into these

54

00:05:43.240 --> 00:05:45.389

PT6: physical activities.

55

00:05:49.700 --> 00:05:59.970

NM: Thank you so much. And okay. Now to our third question, moving right along. do you address promoting physical activity during your actual sessions, your Pt sessions?

56

00:06:01.240 --> 00:06:02.420

PT6: do I? Just

57

00:06:03.630 --> 00:06:04.290

okay.

58

00:06:04.540 --> 00:06:08.120

PT6: Well, in my setting. I

59

00:06:08.210 --> 00:06:09.170

PT6: so

60

00:06:09.290 --> 00:06:20.579

PT6:I don’t interact with the parents as much. So I would say that would probably be who i'd be promoting and pediatric. I'd probably be promoting it most to parents, so I don't have that experience.

61

00:06:21.710 --> 00:06:28.390

PT6: and with the children who are low functioning i'm probably not talking to them about physical activity. But

62

00:06:29.550 --> 00:06:34.120

PT6: I mean, if they're having a really hard time with it, then I might be talking them through it.

63

00:06:34.800 --> 00:06:35.990

PT6: But I guess

64

00:06:36.130 --> 00:06:48.230

NM: yeah, let me clarify, because I think we're going to get to that point of kind of engaging with the parents. But what like in your actual Pt session do you do to promote physical activity?

65

00:06:51.080 --> 00:06:57.720

PT6: Well, I guess I just I do. The hands on part of enabling them to be typically active.

66

00:06:59.020 --> 00:07:01.150

PT6: Well provide the opportunity

67

00:07:03.120 --> 00:07:04.800

NM: to do what specifically

68

00:07:05.310 --> 00:07:14.440

PT6: well one example could be. If we're having a music group instead of sitting in my chair, i'll get them out on the mat where they can move around with it

69

00:07:14.770 --> 00:07:18.569

PT6: or prop them up so they can see better and move their arms. And

70

00:07:19.120 --> 00:07:20.340

PT6: but that's helps with activity

71

00:07:20.650 --> 00:07:21.520

It's a

72

00:07:24.220 --> 00:07:46.300

NM: thank you. That's a great example now, and you said yes, so You mentioned how you do this, and you gave the music example. But what components of physical activity. Do you address, for example? Are you addressing cardiovascular endurance, muscle, activation, energy, expenditure, and it it could be an exhaustive list. So what things do you feel like you as a therapist, specifically address when you're working on physical activity in your sessions.

73

00:07:48.140 --> 00:07:51.610

PT6: I guess the freedom to move.

74

00:07:52.050 --> 00:07:53.890

PT6: So many of our kids are

75

00:07:53.960 --> 00:07:55.839

PT6: need to be positioned in

76

00:07:55.910 --> 00:08:00.729

PT6: wheelchairs that have a seat, belt, and a chest harness to keep them in a good position, and

77

00:08:00.940 --> 00:08:10.229

PT6: even if I don't get them out on the mat. I like to at least take off their laptray and take off their chest strap, and since there to make sure that they're safe.

78

00:08:10.360 --> 00:08:12.920

PT6: That they can have more freedom of movement.

79

00:08:14.360 --> 00:08:15.939

PT6: And I guess that

80

00:08:16.000 --> 00:08:18.769

PT6: activate whatever skeletal muscles they have.

81

00:08:18.820 --> 00:08:20.469

PT6: that they're able to use

82

00:08:21.540 --> 00:08:24.790

PT6: and move their limbs freely and safely.

83

00:08:33.159 --> 00:08:34.640

NM: Anything else you want to add?

84

00:08:35.070 --> 00:08:40.659

NM: no, I think that's that's my answer. Okay, Great. All right.

85

00:08:40.820 --> 00:08:57.849

NM: Fourth question, and then we have a second half an interview. We're going to look at actual tool. So do you address promoting physical activity that occurs outside of Pt. Session. So we talk about what you do with them. in your time with them. So what do you do outside like? How do you promote it outside of PT?.

86

00:08:58.830 --> 00:09:00.420

PT6: Well.

87

00:09:00.460 --> 00:09:15.849

PT6: we most the majority of probably what I do. If their parents are very involved and they're there, then I will work with them. But a lot of times it's more like the caregivers that work with them, and so what we do is inservicing.

88

00:09:16.000 --> 00:09:17.230

PT6: That's what we call it, like

89

00:09:17.480 --> 00:09:23.010

PT6: educational training on how to put on a TLso, or their bracing or

90

00:09:23.040 --> 00:09:29.120

PT6: change their position, and then we'll make a handout on a guide on different positions in bed.

91

00:09:29.330 --> 00:09:48.539

PT6: and one like excellent example. And I don't know if I think it is. I don't know if it's physical activity. But one child that I have. She's a teenage girl, and she goes on her stomach every day, twice a day now, and being on her stomach, she can move her head around, but she also

92

00:09:48.660 --> 00:10:01.649

PT6: passes a lot of gas and it actually makes her be able to take in food better afterwards. And she used to have to get a rectal tube to relieve, like, as

93

00:10:01.780 --> 00:10:06.899

PT6: because she was so distended. But now that she does this every day. She doesn't have to have that invasive

94

00:10:06.940 --> 00:10:17.160

PT6: procedure, and she doesn't have to take medication for gas. So I would say that physical activity. while kind of simple is really like important to her life.

95

00:10:20.930 --> 00:10:29.470

NM: That's a great example. All right. First prompt. Have you recommended any community programs or events to your students to help increase physical activity.

96

00:10:33.330 --> 00:10:35.769

PT6: yes, I have.

97

00:10:35.910 --> 00:10:39.370

PT6: that has been very limited because of Covid.

98

00:10:40.000 --> 00:10:42.610

PT6: and because we're like a nursing home.

99

00:10:43.960 --> 00:10:45.410

PT6: they don't

100

00:10:45.550 --> 00:10:49.769

PT6: really get to get out very much, but prior to that we

101

00:10:49.910 --> 00:11:01.049

PT6: with have take them out on trips to places. I know we were gonna come to Ihope once. But that got canceled but we did take them to like a special Olympics event in Long Island. And

102

00:11:02.460 --> 00:11:03.260

PT6: yeah.

103

00:11:04.790 --> 00:11:08.230

PT6: do simple things. It's just as much as going to a shoe store, too.

104

00:11:10.220 --> 00:11:10.940

Yeah.

105

00:11:15.910 --> 00:11:17.280

NM: And

106

00:11:18.420 --> 00:11:30.739

NM: what types of equipment have you recommended to help improve home and your case the nursing home or community engagement in physical activity outside of your clinical setting. What you know the gym, or you know your treatment setting.

107

00:11:32.590 --> 00:11:36.990

PT6: yeah, I would say, we do the stander

108

00:11:37.420 --> 00:11:39.330

PT6: outside of our session.

109

00:11:41.310 --> 00:11:45.890

PT6: So that's like we do have one rehab CNA.

110

00:11:46.100 --> 00:11:54.530

PT6: It's kind of similar to like a Pt aide. But it's a CNA, and she can be trained to put them on standers if you're not there.

111

00:11:54.620 --> 00:11:57.650

PT6: There has been like some cases where

112

00:11:57.710 --> 00:12:01.030

PT6: staff. I've also been trained to put a kid on a kid walk.

113

00:12:02.050 --> 00:12:03.909

PT6: but that's more rare

114

00:12:04.340 --> 00:12:05.510

PT6: because it

115

00:12:05.550 --> 00:12:07.489

PT6: it's not like very easy.

116

00:12:07.550 --> 00:12:08.400

PT6: So

117

00:12:08.560 --> 00:12:11.159

PT6: it depends on the kid and the caregivers.

118

00:12:12.450 --> 00:12:13.260

PT6: But

119

00:12:13.360 --> 00:12:14.740

PT6: outside of that

120

00:12:15.260 --> 00:12:17.289

PT6: we also

121

00:12:17.640 --> 00:12:20.870

PT6: might recommend a hydrotherapy spa, which is a nice

122

00:12:20.930 --> 00:12:25.419

PT6: a piece of equipment we have, which is like a a big tub with jets.

123

00:12:26.080 --> 00:12:31.970

PT6: and it has a stretcher that the child can sit on and go inside the spa, and like in.

124

00:12:32.320 --> 00:12:37.739

PT6: rather than just being washed. They also get like the benefits of the warm water and the jets, and

125

00:12:39.140 --> 00:12:44.849

PT6: but we mostly do that within our therapy sessions, not outside of them. But I would recommend it outside of that, too.

126

00:12:47.450 --> 00:12:49.450

PT6: Some parents have been able to do that.

127

00:12:50.310 --> 00:12:52.310

NM: Okay, Awesome. Okay. Great.

128

00:12:52.700 --> 00:12:57.570

NM: so equipment to hydrotherapy spa, and the stander are the most frequently recommended.

129

00:12:57.630 --> 00:12:58.580

NM: Thank you

130

00:12:58.610 --> 00:13:09.579

NM: all right now it's time to move into part 2. So i'm going to pull up a survey. We're almost near the end of our our talk today. let me pull this up for you.

131

00:13:09.740 --> 00:13:15.210

NM: So this is called the Promise Parent Proxy, physical activity, survey.

132

00:13:15.470 --> 00:13:17.050

NM: and

133

00:13:19.970 --> 00:13:21.470

NM: this

134

00:13:22.760 --> 00:13:26.290

NM: This survey was created by the National Institute

135

00:13:26.830 --> 00:13:27.800

NM: of Health.

136

00:13:28.240 --> 00:13:30.540

NM: and let me share it now.

137

00:13:34.990 --> 00:13:38.239

NM: So i'm going to let you look at these over these questions

138

00:13:38.510 --> 00:13:58.490

NM: because the parent will pick it Pick will answer these questions, but ideally it is to help give an idea of physical activity intensity over the past week. The scale. So it was. It was developed for children that had more neurologic impairment. Specifically, children that were progressing may may have been progressing due to cancer.

139

00:13:58.500 --> 00:14:18.489

NM: and so what i'm looking to see is how pts and eventually parents, how they would rate the level of this being valid in our population, the children that are non inventory. And I told you you pretty much work with that your facility. So i'm going to ask you when I go through each question, how would you rate the appropriateness of the question? 0 not related at all

140

00:14:18.900 --> 00:14:20.570

NM: 5 appropriate

141

00:14:20.740 --> 00:14:23.450

NM: for this population, and I'm going to ask you why.

142

00:14:23.610 --> 00:14:24.490

NM: All right.

143

00:14:24.840 --> 00:14:26.490

PT6: wait, am I? Answer it?

144

00:14:26.580 --> 00:14:36.620

NM: You're not answering the question. But you're going to. I'm going to ask you for each question. How would you rate of how appropriate it is? So how you? The question is not the number of days.

145

00:14:36.860 --> 00:14:42.140

NM: No, not the number of days. They're all the same like they're gonna all ask for each question. How many days with this child.

146

00:14:42.200 --> 00:14:55.819

NM: active or read hard, or what have you? And that's the parent? Answer this or the it could be any caregiver or therapist. But this is a scale for physical activity, intensity. And so looking to see how appropriate this is for children that are not inventory.

147

00:14:56.040 --> 00:15:10.650

PT6: Okay, and 5 is not appropriate, and one is 5 is highly appropriate. It is not appropriate at all, and you can give me a range if you think it's somewhere in the middle like somewhat appropriate. You just get in between

148

00:15:12.440 --> 00:15:17.129

NM: sound good again. I'll. I'll say that again for each question. So let's start with Question number one

149

00:15:17.790 --> 00:15:37.630

NM: how would you? I'm a re. I'll say it each time. So you have some timing. I'll keep it up there so you can see. So the first question is, how many days that your child exercise or play so hard that his or her body got tired 0, not related or appropriate at all; 5 being highly appropriate for children that are non ambulatory. How would you rate it?

150

00:15:37.910 --> 00:15:40.020

PT6: I would give that a

151

00:15:40.520 --> 00:15:41.590

PT6: 3.

152

00:15:41.780 --> 00:15:42.900

NM: Okay? And why?

153

00:15:43.750 --> 00:15:46.200

PT6: Because

154

00:15:48.670 --> 00:15:50.999

PT6: I don't know, I guess, because the

155

00:15:54.340 --> 00:15:59.180

PT6: my kids don't really like play or exercise per se.

156

00:16:07.540 --> 00:16:09.210

PT6: Maybe that's not true.

157

00:16:14.380 --> 00:16:17.630

PT6: I'll give it a 4. I take it back.

158

00:16:17.740 --> 00:16:31.089

NM: Okay. So you think it's more appropriate because the kids don't like that. I have. You know they don't like player exercise per se, or they don't like it. It's just that they're not necessarily really actively participating in play.

159

00:16:31.410 --> 00:16:32.840

PT6: but

160

00:16:32.950 --> 00:16:34.520

PT6: some of the kids do

161

00:16:35.310 --> 00:16:39.499

PT6: I? Really it? It depends on their cognitive awareness.

162

00:16:40.720 --> 00:16:48.000

PT6: So there are some children who are a little more aware. And then the others who aren't. So that's what the only thing.

163

00:16:49.680 --> 00:16:56.950

PT6: So I just to clarify. So it's more appropriate for kiddles. They have more cognitive abilities or what you're saying.

164

00:16:57.260 --> 00:16:58.040

NM: Okay.

165

00:17:02.460 --> 00:17:05.729

NM: and remember, the parents are answering this so they would be able

166

00:17:06.119 --> 00:17:07.149

NM: to infer

167

00:17:07.180 --> 00:17:08.550

NM: is kind of your thought.

168

00:17:09.000 --> 00:17:10.620

NM: I don't want to.

169

00:17:10.859 --> 00:17:15.570

PT6: Yeah. So this is wait. This is for parents to answer. Correct. Okay

170

00:17:15.790 --> 00:17:18.490

NM: about what they think their child did over the past week

171

00:17:22.420 --> 00:17:35.619

NM: alright, let's go on to number 2. If you have any other thoughts, you know, let me know. So number 2, how many days your child exercise really hard for 10 min or more. Is it not appropriate at all? 0, or somewhere along the scale, up to 5 highly appropriate?

172

00:17:37.690 --> 00:17:38.920

PT6: I'm doing this.

173

00:17:38.940 --> 00:17:43.369

PT6: Children who are not ambulatory like are in manual wheelchair.

174

00:17:44.050 --> 00:17:48.349

PT6: pushing themselves or well for can push themselves.

175

00:17:48.520 --> 00:17:49.530

PT6: Okay

176

00:17:54.560 --> 00:17:58.069

NM: like that. But it doesn't mean Prop: I'm: sorry.

177

00:17:58.280 --> 00:18:02.730

NM: 3 can mainly propel for are typically driving a power wheelchair.

178

00:18:02.940 --> 00:18:03.790

PT6: Okay.

179

00:18:03.920 --> 00:18:05.109

NM: a 5 cannot

180

00:18:06.470 --> 00:18:08.710

PT6: right. Let me correct. Yeah.

181

00:18:08.850 --> 00:18:15.669

PT6: Yeah. So I would say, I work mostly with children who are in level 5 and for level 4,

182

00:18:15.930 --> 00:18:20.679

PT6: I it probably is like a 3 or 4. As for appropriate enough.

183

00:18:21.270 --> 00:18:23.590

NM: Okay, give me a number, so I can just lock it in

184

00:18:23.880 --> 00:18:25.930

PT6: 3.

185

00:18:27.010 --> 00:18:28.060

NM: And why?

186

00:18:29.450 --> 00:18:35.929

PT6: because exercising really hard is probably not the appropriate term for

187

00:18:36.030 --> 00:18:39.670

PT6: the physical activity that I think these kids would do.

188

00:18:47.490 --> 00:18:48.320

NM: Okay.

189

00:18:48.970 --> 00:18:58.629

NM: Number 3. How many days your child exercise so much that he or she breathed hard. How appropriate is this one for a child? and levels 4 and 5.

190

00:19:03.520 --> 00:19:09.790

PT6: I would give this a for I just would probably change the word exercise. But

191

00:19:10.790 --> 00:19:14.340

PT6: to like engage in physical activity.

192

00:19:14.390 --> 00:19:17.080

PT6: but that one could be a 4.

193

00:19:18.220 --> 00:19:22.689

NM: Why, though, even if you change the word, why is this appropriate for this population.

194

00:19:23.140 --> 00:19:27.019

PT6: because I think that is something

195

00:19:27.070 --> 00:19:28.849

PT6: that you monitor

196

00:19:28.930 --> 00:19:33.709

PT6: like, even if sitting up in supported sitting, or

197

00:19:33.770 --> 00:19:38.680

PT6: just being out, is taxing for you. Then you can notice that that

198

00:19:38.700 --> 00:19:41.820

PT6: nasal flaring, or whatever I think this is appropriate.

199

00:19:46.500 --> 00:19:52.749

NM: Okay, Great Number 4. How many days was your child so physically active that he or she sweated.

200

00:19:52.880 --> 00:19:58.919

NM: How would you rate this one? for the 4 and 5 0 not related at all, 5 being highly appropriate.

201

00:19:59.540 --> 00:20:04.000

PT6: I think this one's less appropriate, like 2 or 3, but I have to pick one

202

00:20:05.850 --> 00:20:07.860

PT6: So i'm gonna say 3

203

00:20:10.220 --> 00:20:13.569

PT6: for me. Usually, if my, if the kids are sweating. I

204

00:20:13.960 --> 00:20:16.270

PT6: feel like that's an indicator that

205

00:20:16.340 --> 00:20:18.060

PT6: they need to cool down

206

00:20:18.160 --> 00:20:22.550

PT6: like they're usually there. Things are too taxing for them. If they're sweating

207

00:20:25.190 --> 00:20:26.680

PT6: and

208

00:20:27.470 --> 00:20:29.560

PT6: it's usually that, just like

209

00:20:29.830 --> 00:20:35.089

PT6: their own body system that's making them sweat. Not necessarily the activity, I think.

210

00:20:42.980 --> 00:20:44.999

NM: All right. Number 5.

211

00:20:45.280 --> 00:20:51.149

NM: How many days. Did your child exercise or play so hard that his or her muscle burned?

212

00:20:51.690 --> 00:20:54.909

PT6: They're all not related. 5 highly appropriate.

213

00:20:55.280 --> 00:21:00.699

PT6: I would say 2. I don't think that one's so appropriate? Because

214

00:21:01.640 --> 00:21:03.070

PT6: I

215

00:21:03.170 --> 00:21:05.749

PT6: necessarily think that

216

00:21:06.990 --> 00:21:13.579

PT6: we can, that they could say whether their muscles burn or not. And also I don't really think that's the

217

00:21:13.620 --> 00:21:18.120

PT6: extent of activity that we would be working towards in this population.

218

00:21:24.340 --> 00:21:27.029

PT6: I don't even know if I want my own kids muscles to burn.

219

00:21:28.940 --> 00:21:30.940

PT6: but okay.

220

00:21:33.770 --> 00:21:40.990

NM: and what I get. this is just a question. Why don't you want their muscles to burn this population specifically? Why wouldn't you want that?

221

00:21:41.430 --> 00:21:43.880

PT6: I guess I think like just

222

00:21:44.120 --> 00:21:47.230

PT6: sometimes there for them, like just

223

00:21:47.300 --> 00:21:49.779

PT6: grieving and sitting is

224

00:21:49.980 --> 00:22:00.660

PT6: taxing, and that's like a can be a workout. So if they're doing something that's gonna actually fatigue their muscles, I think that's a little beyond what's necessary.

225

00:22:03.380 --> 00:22:04.880

NM: Why, Why.

226

00:22:09.170 --> 00:22:11.450

PT6: you know I don't know why it just doesn't

227

00:22:11.690 --> 00:22:17.990

PT6: i'm not i'm not, I guess. I guess i'm thinking like in sm a how fatiguing the muscles actually

228

00:22:18.160 --> 00:22:26.790

PT6: negative aspect. But I would say in a higher functioning group of people children with Cp. That they could do like work out

229

00:22:26.920 --> 00:22:32.979

PT6: with weights. But I don't think levels. 4 and 5 are gonna get gains from that.

230

00:22:38.980 --> 00:22:40.840

NM: Thank you. Number 6.

231

00:22:41.510 --> 00:22:47.089

NM: How many days did your child exercise or place so hard that he or she felt tired.

232

00:22:49.610 --> 00:22:50.870

PT6: Hmm.

233

00:22:51.470 --> 00:22:53.400

PT6: Okay, that's a 3.

234

00:22:53.600 --> 00:22:54.570

NM: Okay? Why?

235

00:22:55.090 --> 00:22:58.970

PT6: because if

236

00:23:00.380 --> 00:23:08.250

PT6: like that's just it. A child in a power chair, it goes out with a group of kids and take that with them, and then they come back and they.

237

00:23:08.740 --> 00:23:11.709

PT6: But that was so fun I feel tired that seems appropriate.

238

00:23:11.830 --> 00:23:15.659

PT6: but that also kind of relies on him being verbal.

239

00:23:20.640 --> 00:23:24.610

PT6: So I don't have work with many kids that are verbal. But

240

00:23:25.150 --> 00:23:25.880

PT6: if

241

00:23:26.140 --> 00:23:29.970

PT6: they are, then I think this question is appropriate. If they're not, i'm not so sure.

242

00:23:30.580 --> 00:23:38.609

NM: Well, even if a parent the child's nonverbal, the parent would be the one to determine if they feel like their child is feeling tired.

243

00:23:38.740 --> 00:23:40.809

PT6: Okay? Then? Yes, that's a 3.

244

00:23:41.010 --> 00:23:42.550

PT6: Okay, yeah.

245

00:23:46.140 --> 00:23:51.399

NM: Number 7. How many days with your child physically active for 10 min or more.

246

00:23:51.460 --> 00:23:57.490

NM: How would you rate that 1 0 not related to this population at all? 5 being highly appropriate.

247

00:23:58.190 --> 00:24:02.190

PT6: I would give that a 5. I think that's a appropriate question.

248

00:24:02.510 --> 00:24:17.870

PT6: Okay, that's first 5. Why? Why, you might have to define what physically active means. to the parent. but it just seems like this Seems like a question a parent could answer even for a child who doesn't do very much.

249

00:24:19.600 --> 00:24:25.130

PT6: even if it's just like taking it back, or sitting in a chair

250

00:24:26.730 --> 00:24:30.139

PT6: and looking at things. I think that a question is appropriate.

251

00:24:32.200 --> 00:24:33.250

NM: Number 8.

252

00:24:33.760 --> 00:24:48.969

PT6: How many days your child run for 10 min or more. Well, that's a one, because there are non ambulatory. So well, the lowest 4 0. So you want to do one or 0, 0, because you know I would have one as a parent that if they're not ambulatory.

253

00:24:53.240 --> 00:25:07.280

NM: I hope I gave the right answers. No such thing as the right answers your opinion from your clinic or clinical expertise. But it's so. It's so helpful to get your thoughts about this category, especially with the population. You serve. You know.

254

00:25:11.570 --> 00:25:21.949

NM: All right. That is the last question. And so, before we end, I always like to ask the Pt. Is there any like last thoughts you want to share about physical activity in this population that you want to share.

255

00:25:27.790 --> 00:25:30.539

PT6: no. I think we covered it all

256

00:25:30.740 --> 00:25:32.550

PT6: things like, you know.

257

00:25:32.880 --> 00:25:36.729

PT6: in this population the people have to

258

00:25:37.040 --> 00:25:40.519

PT6: sort of change their mindset on what physical activity means.

259

00:25:40.890 --> 00:25:43.349

PT6: and I think, like

260

00:25:43.740 --> 00:25:46.619

PT6: a survey, should be sensitive to that.

261

00:25:48.200 --> 00:25:56.339

PT6: and appropriate for parents like, I think, sometimes standardized tests are not the best thing to give to parents when they are popular, when they have a very low functioning child.

262

00:25:56.400 --> 00:26:01.539

PT6: because they ask some questions that, like have never even been an option for their child; and

263

00:26:01.930 --> 00:26:03.180

PT6: I think that like

264

00:26:03.450 --> 00:26:09.470

PT6: could be crushing for them, whereas like a survey like this is good as long as it's geared towards the population.

265

00:26:12.240 --> 00:26:15.660

PT6: And maybe the term could be defined like physical activity.

266

00:26:21.040 --> 00:26:21.740

So i'm gonna

267

00:26:24.530 --> 00:26:29.470

NM: Well, thank you so much. I'm glad to hold on a second.
