# Supplementary material for: Validity of PROMIS® Pediatric Physical Activity Parent Proxy Short Form Scale as a Physical Activity Measure for Children with Cerebral Palsy Who Are Non-Ambulatory
Source: Behav Sci (Basel). 2025 Jul 31;15(8):1042. doi: 10.3390/bs15081042 (PMC12382615; doi:10.3390/bs15081042)
Supplement: Supplementary file 1 [file behavsci-15-01042-s001.zip › Transcripts copy/PT transcripts - deidentified/PT16.docx]

WEBVTT

1

00:00:02.460 --> 00:00:19.580

NM: Good evening. Thank you so much for joining us tonight as we do an interview about physical physical activity in children who are not full time, Walker. So the first half of this interview I'm going to ask you some questions related to physical activity, and then the second. I i'm going to show you a survey.

2

00:00:19.590 --> 00:00:23.529

NM: and I'm going to ask you to kind of look at it and see how valid

3

00:00:23.580 --> 00:00:42.870

NM: this survey kind of just on his face is, as it relates to physical activity in this population, so we'll get there soon. I do have a little bit of a script, so if it sounds like I'm scripted is because I am. There are no right or wrong answers, and so feel free to just share. Okay. So the first question is.

4

00:00:42.940 --> 00:00:49.130

NM: How do you define physical activity for children with CP who are not full time walkers?

5

00:00:49.950 --> 00:00:54.770

PT16: Well, physical activity for children who are not full Time walker? I think

6

00:00:54.840 --> 00:00:55.860

PT16: it's not

7

00:00:56.030 --> 00:01:00.729

PT16: how much, or you know it's not. It's not about the

8

00:01:00.960 --> 00:01:13.690

PT16: how much they can do like it is, how little or how you know even how little they can participate in the movement. You know we can do it on the mat, on the ball, or

9

00:01:13.820 --> 00:01:20.589

PT16: even when they are standing, you know, even when they are standing. I feel like the physical activity is present.

10

00:01:20.720 --> 00:01:30.710

PT16: you know, with them getting their head up. Everything is all encompassing like you know, even taking them out of their wheelchair. It's already like a therapy for them.

11

00:01:32.700 --> 00:01:33.910

NM: Thank you.

12

00:01:33.930 --> 00:01:46.840

NM: So the Department of Health defines physical activity as any activity that encompasses energy expended, and activation of skeletal muscle. Does this definition change your mind about how you define physical activity?

13

00:01:47.190 --> 00:01:48.320

PT16: No

14

00:01:49.510 --> 00:01:57.249

NM: great and how do you think physical activity differs from other types of fitness activity

15

00:01:58.080 --> 00:02:02.190

PT16: for the students with that are nonambulatory.

16

00:02:02.410 --> 00:02:03.860

PT16: Yeah. Well.

17

00:02:03.950 --> 00:02:12.399

PT16: of course, the way we're going to look at. It is so much different from like the exercises that's being done, and like the physical fitness exercises right.

18

00:02:12.500 --> 00:02:16.089

PT16: because we're not really looking at

19

00:02:16.670 --> 00:02:32.130

PT16: those. You know what we're working on is more on like postural control, you know improving mobility where us in physical fitness, I think it's more on, although strengthening is also a part of what our goals are.

20

00:02:32.140 --> 00:02:40.609

PT16: you know, but I think the focus is a little bit different than you know what we do in the gym with when we do physical fitness.

21

00:02:40.700 --> 00:02:41.430

NM: Gotcha.

22

00:02:44.460 --> 00:02:49.950

NM: And when do you witness your students participate most in physical activity during the school day?

23

00:02:51.400 --> 00:02:56.909

PT16: When is it like? Are you asking if it's in the morning or afternoon?

24

00:02:56.930 --> 00:02:59.600

NM: No, no! What When do you witness? Your students

25

00:02:59.790 --> 00:03:01.630

NM: participate the most

26

00:03:01.670 --> 00:03:06.030

NM: it? When do they participate in most in physical activity during their school day.

27

00:03:06.190 --> 00:03:09.520

NM: Like when do you feel like they're doing the most physical activity during the day?

28

00:03:09.690 --> 00:03:23.610

PT16: Well, of course, when they, when we we have the movement, you know, in school we now have like movement program. We like. That's the goal throughout the day. So the kids are supposed to be taken out of the chair, and they're supposed to participate in

29

00:03:23.620 --> 00:03:43.069

PT16: It could be anything like that. It's PT related so it could be yoga as long as they are taken out of the chair. But I feel like, when they are in physical therapy this most of time, that they are participating in physical activity in school, because other than physical therapy most of the time they are in their chair.

30

00:03:45.210 --> 00:03:46.379

NM: Why is there in the

31

00:03:47.510 --> 00:03:48.730

NM: all right? Great

PT16: that question. I'm not sure if I answered that question.

32

00:03:48.940 --> 00:04:07.680

NM: No, you did. No, you did all right. So second question, how do you measure physical activity, frequency, intensity, time and type, so quoting the fit principle in children with Cp. Who are not full time. Walker and I'll say it again.

33

00:04:07.930 --> 00:04:11.339

NM: How do you measure physical activity, frequency.

34

00:04:12.080 --> 00:04:18.140

NM: intensity, time and type, and children with Cp. Who are not full time walkers

35

00:04:18.820 --> 00:04:26.580

PT16: all right in our school. The International Academy of Hope for children who are not ambulatory. We usually have

36

00:04:26.710 --> 00:04:35.540

PT16: the high intensity for the students high intensity Therapy. So it's 5 times a week, 60 min of session.

37

00:04:37.200 --> 00:04:38.400

PT16: And

38

00:04:39.420 --> 00:04:47.690

PT16: yeah, so that's the the the the therapy that we provide. So it's mostly 4 to 5 times a week of therapy

39

00:04:50.410 --> 00:04:53.800

NM: Okay, great. And how would you measure

40

00:04:54.440 --> 00:05:01.639

NM: the type like, so that that would be kind of You're talking more about frequency. How about how you measuring their intensity

41

00:05:02.750 --> 00:05:05.310

PT16: like how off, often, how

42

00:05:05.500 --> 00:05:11.570

NM: how like, how they get. You know how, when they're going different levels like, is it easy when they go from easy medium

43

00:05:11.590 --> 00:05:12.790

NM: or high.

44

00:05:12.850 --> 00:05:20.519

PT16: You know, I think it all depends on the child. We listen to like what they will. Let us do like Sometime we

45

00:05:20.580 --> 00:05:32.060

PT16: each day is different, like some of the kids will let us do more for the day, and some of the kids like in the beginning of the session, they will already say, like I wanted to do like a spa day today, you know.

46

00:05:32.440 --> 00:05:37.879

PT16: You know it will listen to that to our kids to like during therapy like what they wanted us to do.

47

00:05:39.600 --> 00:05:46.019

NM: How do you know they go from different levels like when it becomes vigorous for them like once? You know. How do you? How can you see?

48

00:05:46.160 --> 00:05:52.370

NM: How do you measure their physical activity, intensity like when they get from one zone to the next

49

00:05:52.380 --> 00:06:06.910

PT16: like Yeah. Well, usually they will express it like facial expression is one thing that you know really tells us like, okay, this is too much for me. I need a break. They use their communication device or they vocalize.

50

00:06:07.910 --> 00:06:09.470

NM: That is great. Thank you.

51

00:06:13.950 --> 00:06:16.739

NM: And do they need assistance to complete

52

00:06:16.940 --> 00:06:24.410

NM: these activities that we talked about in physical therapy or do? And during what activities. Do they need assistance

53

00:06:25.130 --> 00:06:27.830

PT16: For the most part.

54

00:06:28.120 --> 00:06:32.549

PT16: for the most part they do need assistance to complete

55

00:06:33.440 --> 00:06:37.489

PT16: the activities that are, you know, being asked of them in therapy

56

00:06:38.010 --> 00:06:41.170

NM: for the entire task? Or is it part of the task? Can you give some examples?

57

00:06:41.250 --> 00:06:57.169

PT16: Well, usually we'll start off with providing them like activities on the mat on it, you know, activating their muscle, so they will be able to participate better when they're sitting or when they're rolling. It depends on

58

00:06:57.260 --> 00:06:58.460

PT16: how

59

00:06:58.560 --> 00:07:11.660

PT16: they respond to that handling. So sometimes, you know, there are times that they won't need as much assistance. But day to day it's different. It's usually difference in a day to day basis.

60

00:07:14.960 --> 00:07:20.319

NM: And do you think they should participate in more or less of these activities, and why?

61

00:07:20.750 --> 00:07:28.360

PT16: I think they should participate more. I feel like if they're given the opportunity to most of them, they just don't have the the experience.

62

00:07:28.410 --> 00:07:31.030

PT16: You know. We've had students that

63

00:07:32.260 --> 00:07:41.359

PT16: they just got into our school. They're already like in their teen, like teenage years. So hasn't gotten any, you know any therapy in the past.

64

00:07:41.700 --> 00:07:53.410

PT16: you know. But even though they're in school, they don't get that much therapy, so I feel like as long as they're given the opportunity, and they experience to do all this things I feel like the more the better.

65

00:07:57.760 --> 00:07:58.710

NM: Alright.

66

00:07:59.370 --> 00:08:00.400

NM: And

67

00:08:00.970 --> 00:08:09.500

NM: okay. Next question actually, do you address promoting physical activity during your Pt sessions? And if you ask, how do you do this?

68

00:08:09.690 --> 00:08:16.710

PT16: promoting activity. Of course. Yes, all of our sessions are geared towards promoting activities for our kids.

69

00:08:16.720 --> 00:08:30.010

PT16: That's why we have like the 60 min of session. So we can start with preparing the students preparing their muscles so can they participate in functional activities, like, you know, sitting standing or walking.

70

00:08:30.660 --> 00:08:33.799

PT16: Yeah, Always promoted during our sessions.

71

00:08:34.700 --> 00:08:38.330

PT16: I always tell the the PTs to like if the kids are

72

00:08:38.600 --> 00:08:40.600

PT16: sleeping during the session.

73

00:08:41.020 --> 00:08:50.919

PT16: even if we're just working on positioning the kids, or as long as they're taken out of the chair. It's like therapeutic for them, like, even if it's just putting on their

74

00:08:51.050 --> 00:08:55.070

PT16: you know their orthotics, or getting their

75

00:08:55.230 --> 00:08:58.060

PT16: getting them in a good position, its therapeutic

76

00:08:58.080 --> 00:08:58.880

PT16: for them.

77

00:09:02.780 --> 00:09:08.509

NM: And what components of physical activity are you addressing, for example, Are you addressing

78

00:09:08.850 --> 00:09:17.379

NM: cardiovascular endurance, muscle, activation, mobility, energy, expenditure. What are you primarily addressing during your physical activity? What components?

79

00:09:17.980 --> 00:09:27.169

PT16: Well, I think it's mostly like promoting their postural control for for nonambulatory kids. And then after that we promote that mobility

80

00:09:27.200 --> 00:09:27.970

PT16: part.

81

00:09:29.020 --> 00:09:29.830

NM: Okay.

82

00:09:30.530 --> 00:09:34.469

PT16: yeah, and that goes with my muscle activation and all that. But

83

00:09:36.480 --> 00:09:37.390

NM: got it.

84

00:09:39.630 --> 00:09:41.050

NM: And

85

00:09:41.340 --> 00:09:46.820

NM: if you don't work on something, why wouldn't you like there? Is there ever a reason why you would not work on

86

00:09:47.330 --> 00:09:49.010

NM: muscle activation?

87

00:09:49.070 --> 00:09:55.260

PT16: If they are in pain, it's contraindicated. They had just surgery.

88

00:09:55.350 --> 00:10:06.720

PT16: or if they had seizure, usually that's one thing. Like most of the students that we have at school have seizure disorder if they recently had the seizure, and you know

89

00:10:07.050 --> 00:10:16.159

PT16: their medication, and they're falling asleep during this during our session. So that is something that, you know, warrents us to change the

90

00:10:16.250 --> 00:10:20.089

PT16: the plan for the day. But following day is different, you know.

91

00:10:20.370 --> 00:10:22.250

NM: Gotcha. Okay.

92

00:10:22.390 --> 00:10:30.330

NM: Number 4. Do you address promoting physical activity that occurs outside of your physical, Your Pt: session.

93

00:10:30.860 --> 00:10:31.650

PT16: Yeah.

94

00:10:31.850 --> 00:10:37.689

PT16: Yeah. Physical activity outside of PT Session, so we have adjunctive therapy and school like

95

00:10:37.740 --> 00:10:42.380

PT16: the walking program. So for kids who are already

96

00:10:42.400 --> 00:11:00.180

PT16: starting to walk, using the gait trainer. So the paraprofessionals are trained to use the gait trainer so that they can walk with with the kids or standing program, or it could be a lying program, just taking them out of their wheelchair, getting them on the Mat. They can practice.

97

00:11:00.190 --> 00:11:08.940

PT16: you know, rolling on the mat, or just, you know, getting them or their belly working on head control. So that's one we're promoting that

98

00:11:09.110 --> 00:11:12.259

PT16: mobility outside of their physical therapy.

99

00:11:14.060 --> 00:11:21.610

NM: What types I'm sorry. Have you recommended any community programs or events to your students to help increase physical activity?

100

00:11:22.220 --> 00:11:42.370

PT16: Yes, so currently. We have, like the mobility movement program in school, which is like embedded throughout their day. Like it's a movement program. But this is now happening like once a week, so the goal is to get it incorporated throughout the week, like on a daily schedule. So it's like once a week every 1'clock

101

00:11:42.380 --> 00:12:00.260

PT16: there will be like movement blocks. So the kids, while they are in the classroom. So they are all moving. They're moving with their classmates. It's either there, you know, transitioning to the bathroom, let's say, like, just like lining up, or whatever it's just promoting movement, and the teachers are

102

00:12:00.270 --> 00:12:15.649

PT16: are taught how to do it and the paraprofessionals. So it's also like a push in sessions from other related service providers, so they also know how to move with their kids. It's a time for them to learn how their kids move.

103

00:12:16.340 --> 00:12:27.200

NM: It's great. How about any community programs outside of the school? Okay, or it could be something in their own community.

104

00:12:27.390 --> 00:12:30.610

NM: I'll include the trips. Those trips are great. What trips to where

105

00:12:30.990 --> 00:12:44.309

PT16: field trips to the library. Well, recently we just moved so like we're exploring the different places. Last time we went to…to see the Christmas tree.

106

00:12:44.730 --> 00:12:51.399

PT16: Yeah. So those are the things we're trying to learn about the community where we're currently at

107

00:12:51.440 --> 00:12:53.430

we have.

108

00:12:53.800 --> 00:12:56.689

PT16: We used to bring the kids out like just

109

00:12:56.770 --> 00:13:00.020

PT16: using their tricycle or walking around the block.

110

00:13:00.220 --> 00:13:09.999

PT16: What I think, when the weather permits. That was. That is something that we'll be able to do again. You know, in our current location

111

00:13:10.230 --> 00:13:14.469

NM: Great, how about for the families outside of like at home.

112

00:13:15.700 --> 00:13:19.420

NM: You have any like programs that you may recommend for parents to go to

113

00:13:20.810 --> 00:13:28.739

NM: programs outside of school. Yeah. But no, i'm not like school. I'm not like another school program like any community programs or advance.

114

00:13:28.870 --> 00:13:33.220

PT16: so that the parents could get the kids active.

115

00:13:33.230 --> 00:13:50.279

PT16: Yeah. So during the pandemic. The New York City ballet actually have been providing zoom zoom workshops for the students or they're sharing it with. Ihope community. So they used to come to our schools, and now they're doing it through zoom, too.

116

00:13:50.290 --> 00:13:54.560

PT16: to so that they can reach out to. You know more families.

117

00:13:54.880 --> 00:13:57.789

NM: Yeah. So do you guys. Still.

118

00:13:58.660 --> 00:14:05.739

NM: what was the other thing? It was a community event. Yeah, I used to. I can't think of the name of it. Okay. that we said that the families could be invited to, so that the central part challenge we do,

119

00:14:06.060 --> 00:14:18.849

PT16: we do have the central part. Challenge is once a year every June. And oh, yeah, the disability walk.

120

00:14:19.400 --> 00:14:26.949

PT16: the disability walk, the disability pride Walk, the Central Park Challenge.

121

00:14:27.180 --> 00:14:33.269

PT16: Those are the things. Yeah, I forgot about that.

NM: Those are events. Right? Yeah, those are events that

122

00:14:33.420 --> 00:14:36.580

NM: I remember hearing about that before. Yeah.

123

00:14:36.890 --> 00:14:38.709

NM: I shared with the families.

124

00:14:38.960 --> 00:14:39.650

NM: hey?

125

00:14:40.600 --> 00:14:47.849

NM: All right. And what types of equipment have you recommended to help improve home and or community engagement

126

00:14:48.630 --> 00:14:51.769

PT16: Gait trainers.

127

00:14:51.940 --> 00:14:54.160

PT16: wheelchairs

128

00:14:55.000 --> 00:14:55.710

Yeah.

129

00:14:56.070 --> 00:15:01.940

PT16: those are the things most of the time strollers. But most of the time we do recommend

130

00:15:02.050 --> 00:15:04.100

PT16: gait trainers and the standers.

131

00:15:05.090 --> 00:15:06.090

NM: Gotcha.

132

00:15:06.130 --> 00:15:06.980

NM: All right.

133

00:15:07.140 --> 00:15:14.180

NM: all right. Part 2. This is the part about this that I was telling you about. Let me pull that up.

134

00:15:16.800 --> 00:15:17.610

NM: Cool.

135

00:15:32.640 --> 00:15:35.179

NM: Okay, great. Here we go. So

136

00:15:36.330 --> 00:15:39.249

NM: do you see it? Okay. So that's the this is the

137

00:15:39.830 --> 00:15:49.400

NM: promise Parent proxy, physical Activity survey, and i'll read the questions when we talk, start talking about it. So for you to look at it. So basically the parents would

138

00:15:49.670 --> 00:15:55.480

NM: answer each question about how many days their child would have has engaged in

139

00:15:55.510 --> 00:16:01.940

NM: the the the question at hand. Okay. And so what? This was developed for

140

00:16:02.240 --> 00:16:19.110

NM: a population of children that were not typically developing or they were had some kind of disability. So i'm looking to see if this would be a good scale for children with that are not ambulatory with CD. The level. This are questionnaires for the parents right. The parents will fill it out

141

00:16:19.120 --> 00:16:30.709

NM: for the child, and and it'll be about their activity. Okay, so what I'm going to ask you, though, about each question is, how appropriate would this be for this population? 0 not related at all.

142

00:16:31.120 --> 00:16:36.630

NM: 5 highly appropriate. Okay? And again, it's on that scale. So, for example, the first question.

143

00:16:37.180 --> 00:16:42.790

NM: How many days your child exercise your place so hard that his or her body got tired.

144

00:16:42.820 --> 00:16:50.679

NM: How would you rate this question as it pertains to children with Cp. And levels 4 and 5 0, not related at all.

145

00:16:50.700 --> 00:16:54.419

NM: 4, 5 highly appropriate. And then why.

146

00:16:54.970 --> 00:17:13.219

PT16: I think it's highly appropriate for us to know like that's why I said, like, since we see it on a daily basis, and it changes from day to day. So I feel like it's highly appropriate to know, like, how many times you know, if if they can participate that whole time for therapy.

147

00:17:13.230 --> 00:17:21.850

NM: So how would how would you write Number one? How many days did your child exercise a place so hard that his or her body got tired. What number would you give that one? And why

148

00:17:23.520 --> 00:17:25.719

PT16: for this one like if I were the parent?

149

00:17:26.060 --> 00:17:39.259

PT16: Oh, no, you as a as a physical therapist, I you will give it a 5. It's a good question. Basically yeah, yeah, 5. I think it's a highly appropriate question for you know, I think it's good to know like

150

00:17:39.360 --> 00:17:42.210

PT16: how often, how often they can participate in therapy.

151

00:17:43.210 --> 00:17:44.060

NM: Okay.

152

00:17:44.380 --> 00:17:45.740

NM: And

153

00:17:46.020 --> 00:17:48.290

NM: this one. This question was about

154

00:17:48.330 --> 00:17:50.909

NM: how, if they played so hard

155

00:17:51.090 --> 00:17:54.390

NM: that his or her body got tired. This is the first question.

156

00:17:54.420 --> 00:18:04.529

NM: How many days did your child exercise a place so hard that his or her body got quite tired. So you give that question of 5? You think that question is a very appropriate.

157

00:18:04.610 --> 00:18:07.869

PT16: I think it's appropriate to know, like how many days. Yep.

158

00:18:08.280 --> 00:18:15.909

NM: Well, they're all they Each question is about how many days in the week up. Okay, so i'm going to ask you for each question i'm asked you to rate each question.

159

00:18:16.120 --> 00:18:28.160

NM: Right? So like is like some one question, maybe better than the next, or they all may be great right. But i'm gonna ask you, how do you think this question is appropriate for the kids

160

00:18:28.350 --> 00:18:30.989

NM: that you have at your school? Okay.

161

00:18:31.120 --> 00:18:37.419

NM: so. And the parent has to answer this, so it's not. We don't answer as a therapist. The parent would have an answer. Okay.

162

00:18:37.460 --> 00:18:50.319

NM: So the first question was, how many days. Did your child exercise or play so hard that his or her body got tired? How appropriate would this be for a parent of a child at level 4 and 5 to answer

163

00:18:51.270 --> 00:18:55.660

NM: a 5. Good. Okay? And why did you rate it at 5?

164

00:18:56.030 --> 00:19:11.409

PT16: Yeah. Because I think it is important for you know, for both the parents and the therapists to know. Like if the if the if the child who is, you know, participating in the physical activity, is able to participate like for the full

165

00:19:11.560 --> 00:19:13.680

PT16: 5 days of therapy.

166

00:19:13.840 --> 00:19:20.900

NM: right? But the question is different, because it's asking about that. They have to play so hard that their body got tired.

167

00:19:22.190 --> 00:19:24.909

PT16: I I don't think i'm getting it right. So

168

00:19:25.260 --> 00:19:30.319

PT16: how many days did your child exercise or play so hard that his or mighty?

169

00:19:30.780 --> 00:19:37.920

PT16: The the question is, if it is appropriate for this to be in that good in that, in this survey. Yes.

170

00:19:38.110 --> 00:19:40.220

NM: like how? How appropriate is it

171

00:19:41.130 --> 00:19:43.310

NM: so? The parent would have to know

172

00:19:43.860 --> 00:19:48.439

NM: that his or her child were played so hard that his body got tired.

173

00:19:48.600 --> 00:19:49.440

PT16: Yeah.

174

00:19:49.700 --> 00:19:59.300

NM: Yeah, I think it is appropriate for the yeah to know that. Okay. I just want to make you. You know, each question is different. Okay, okay, that's what I want. All right. Great. So that's a 5 number 2.

175

00:19:59.310 --> 00:20:13.929

NM: How many days is your child exercise really hard for 10 min or more? How appropriate is asking that a parent that question? And why 0 not related or appropriate, or 5 highly related, or somewhere along that scale?

176

00:20:18.680 --> 00:20:23.549

PT16: How many days did your child exercise really hard for 10 min or more.

177

00:20:23.930 --> 00:20:32.179

PT16: That's something we can ask the parents like 10 min or more. I think it's appropriate, too. Okay, good. How would you rate it? What what score. Would you give it?

178

00:20:32.520 --> 00:20:35.590

PT16: I think it could be a 5, 2, okay?

179

00:20:35.850 --> 00:20:36.810

NM: And why?

180

00:20:37.650 --> 00:20:41.780

PT16: Well, I feel like for most parents they're not.

181

00:20:44.430 --> 00:20:47.550

PT16: I think it is important that the parents know

182

00:20:48.100 --> 00:21:06.579

PT16: their child like how how much potential their child can actually do. So I think this is something that when they're asked, then they will be able to actually look at it. Oh, wait a minute to do this, my child really get tired in 10 min, so I feel like. It's important that they're asked so they can

183

00:21:07.050 --> 00:21:08.000

PT16: check.

184

00:21:08.150 --> 00:21:09.640

PT16: Got it

185

00:21:13.870 --> 00:21:15.349

NM: all right. Number 3.

186

00:21:15.890 --> 00:21:20.759

NM: How many days did your child exercise so much that he or she breathed her.

187

00:21:21.320 --> 00:21:28.709

NM: How appropriate to assess physical activity in this population! 0 not related at all, or 5 highly appropriate.

188

00:21:28.810 --> 00:21:42.759

PT16: It's highly appropriate for us to that's breathing for the children. Would you give that? I think this will be a 5 to I feel like this is also very appropriate. I feel like breathing is something that you know.

189

00:21:42.810 --> 00:21:49.329

PT16: It's something that we can gauge whether the child is actually working hard or, you know. Same with heart rate.

190

00:21:49.670 --> 00:21:50.350

NM: Good.

191

00:21:53.420 --> 00:21:55.000

NM: Okay, Number 4,

192

00:21:57.240 --> 00:21:59.979

NM: a number 4. Okay. How many days

193

00:22:00.010 --> 00:22:04.979

NM: was your child so physically active that he or she sweated. How would you rate this one?

194

00:22:07.240 --> 00:22:10.449

PT16: All right, that he sweated.

195

00:22:10.540 --> 00:22:12.160

PT16: All right, we have.

196

00:22:14.600 --> 00:22:18.539

PT16: Well, actually, we have some kids in school actually sweats without

197

00:22:18.990 --> 00:22:28.870

PT16: being physically active. I feel like temperature. Regulation is always one problem for students with cerebral policy.

198

00:22:29.470 --> 00:22:32.189

PT16: I feel like physical activity. It

199

00:22:32.920 --> 00:22:39.729

PT16: sweating may or may not be like a gauge for it. It could be for me. It's like, maybe in between

200

00:22:40.130 --> 00:22:42.370

PT16: for me. Okay, give me a number

201

00:22:43.530 --> 00:22:45.979

PT16: a of 3 or 4.

202

00:22:46.150 --> 00:22:52.250

NM: Yeah. Final answer. All right. 4 4. Okay.

203

00:22:52.930 --> 00:22:54.760

NM: All right. Number 5.

204

00:22:57.220 --> 00:23:02.899

PT16: How many days did your child exercise a place so hard that his or her muscles burned

205

00:23:06.500 --> 00:23:10.659

PT16: for most of our students who are not ambulatory? They are also non-verbal.

206

00:23:11.000 --> 00:23:15.780

PT16: I'm not sure if they will be able to

207

00:23:17.390 --> 00:23:21.059

express that their muscles burned, or

208

00:23:21.980 --> 00:23:23.570

PT16: I don't know. If this is

209

00:23:24.350 --> 00:23:28.989

PT16: appropriate question, I probably will give this a 3.

210

00:23:29.060 --> 00:23:30.000

NM: Okay.

211

00:23:34.020 --> 00:23:35.320

Okay, 3,

212

00:23:37.110 --> 00:23:38.860

NM: Number 6.

213

00:23:38.910 --> 00:23:50.850

NM: How many days did your child exercise a place so hard that he or she felt tired? How appropriate. Would this one be

214

00:23:50.960 --> 00:23:54.119

PT16: like the numbers of days that they are? They got to? I think

215

00:23:54.520 --> 00:23:59.809

PT16: we they can. They can communicate or express whether they are tired or not. The child

216

00:23:59.890 --> 00:24:00.680

NM: Yeah.

217

00:24:02.420 --> 00:24:07.420

NM: Number 7. How many days was your chat physically active for 10 min or more?

218

00:24:12.510 --> 00:24:16.730

PT16: I think it's good, for I think it's appropriate. I think

219

00:24:16.840 --> 00:24:22.299

PT16: this one say will be appropriate, appropriate to be in this questionnaire as well.

220

00:24:22.470 --> 00:24:24.530

PT16: Okay? Why.

221

00:24:24.710 --> 00:24:38.170

PT16: as we are promoting physical activity for the kids. I feel like it's important to know whether you know how long the child can actually participate in physical activity throughout the day, and

222

00:24:38.930 --> 00:24:41.870

PT16: how long and how many days are they going to be able to do it?

223

00:24:44.240 --> 00:24:45.859

NM: What number did you give me?

224

00:24:46.230 --> 00:24:47.600

PT16: I set a 5?

225

00:24:47.640 --> 00:24:48.550

NM: Thank you.

226

00:24:49.030 --> 00:24:53.229

NM: Number 8. How many days did your child

227

00:24:53.330 --> 00:24:58.399

NM: run for 10 min or more? How would you rate that week? 0 Not appropriate at all?

228

00:24:58.560 --> 00:25:02.279

NM: 5 highly appropriate for this population?

229

00:25:02.630 --> 00:25:09.129

PT16: Well for this population, I feel like this is really appropriate, as you know.

230

00:25:09.860 --> 00:25:11.609

PT16: they're not going to be running.

231

00:25:11.830 --> 00:25:13.399

NM: so what number would you give it?

232

00:25:14.190 --> 00:25:15.530

PT16: I feel like

233

00:25:15.710 --> 00:25:21.619

PT16: I won't Ask it. Some parents might feel like oh, well, why am I being asked this? If they are not running so

234

00:25:23.120 --> 00:25:27.259

PT16: final? Answer is, I give it a

235

00:25:28.050 --> 00:25:31.010

PT16: 0. Okay.

236

00:25:32.500 --> 00:25:34.700

PT16: all right, there's more.

237

00:25:35.010 --> 00:25:39.600

NM: The last thing is your final comments and thoughts.

238

00:25:40.160 --> 00:25:46.140

PT16: What about physical activity as population? Anything you would like to add, as we come to a close.

239

00:25:47.490 --> 00:25:59.169

NM: anything I wanted to ask in this one. No, anything you want to add any last comments we're at the end anything you want to do. Last week you want to share all physical activity in these kids.

240

00:25:59.500 --> 00:26:07.080

PT16: I think physical activity for our students or for the children in Gmf. Cs. 4 and 5 very is very important.

241

00:26:07.270 --> 00:26:11.280

PT16: Feel like it's giving them a different perspective.

242

00:26:13.190 --> 00:26:30.440

PT16: It, you know. I feel like, if even the parents the way they see it is also different, like the way they see their kids at home is different with the way you know. They see their kids in school when they're participating in all this physical activities, getting them on the standard getting them on the gate trainer.

243

00:26:30.450 --> 00:26:43.279

PT16: I feel like it excites the parents as well as the kids, you know. That's why I feel like this is very fulfilling for physical therapist being in this working with this population.

244

00:26:43.340 --> 00:26:45.550

PT16: it's very fulfilling and

245

00:26:45.720 --> 00:26:46.840

PT16: part one.

246

00:26:46.960 --> 00:26:47.610

Yeah.

247

00:26:50.850 --> 00:26:55.710

NM: Well, thank you so much. I mean, stop our recording. We're at the end.
