# Supplementary material for: Validity of PROMIS® Pediatric Physical Activity Parent Proxy Short Form Scale as a Physical Activity Measure for Children with Cerebral Palsy Who Are Non-Ambulatory
Source: Behav Sci (Basel). 2025 Jul 31;15(8):1042. doi: 10.3390/bs15081042 (PMC12382615; doi:10.3390/bs15081042)
Supplement: Supplementary file 1 [file behavsci-15-01042-s001.zip › Transcripts copy/PT transcripts - deidentified/PT1-edited.docx]

NM

we are now recording thank you again for joining us today and we talked about physical activity for children with cerebral palsy and have a few questions so I'm going to start with the questions and again take as much time as you need there is no rush and there's no perfect answer or correct answer this is really from your experience and we value everything that you will share today okay so the first question that I have for you is how would you define physical activity for children with CP who are not full-time Walker so this is gmfcs levels four and five how would you define physical activity for these kids

PT1

so in my perspective I Define them as like they must be able to do something functional and also with assistance with or without assistive devices but they should be able to do something functional based on whatever they can do so that those levels of participation is more important, we want them to do participate, it is very less and also like to something functional that makes them not rest in their seated equipment, they should be out of their chairs doing something functional outside of the chair.

I can’t hear you you're muted

NM

okay I'll see how you did so how do you think physical activity differs from other types of Fitness activity

PT1

I think the grades of 4 and 5 they are very less Fit- fitness in Fitness perspective they are not that they because they don't do much activities like what other grade levels do so I think it is very less and they need more like more repetitions or more frequency or activities so that like they can maintain their level of Fitness whatever they are at this point, it varies upon the age group too if they are like towards the adolescent they are very less participant mobile, compared to the other age groups who are on the same grade level, age is the main factor, motivation, participation that adds to the fitness level to

NM

absolutely thank you and when when do you witness your students participate most and physical activity during the school day?

PT1

of course during the PT session, that’s only time they do more things comparatively because they need more assistance. caregivers like if they are proactive and helping the child to helping the students to participate then they get extra level of mobility or functional activity or it's only during the PT session

NM

Awesome. Okay. So that was our first question. The next one I have for you. How do you measure, physical activity, frequency. intensity, time and type. I'm. I'm not sure if you're familiar with the fit principle, but it's kind of tied to that acronym right frequency, intensity, time and type. So how would you measure that in these children that are not full time Walkers.

PT1

I was saying as far as like the intensity into intensity is usually moderate to maximum for these kids like I'm sorry I'm in the not the moderate activities like whatever they can do you like but not like fully physical activities. How do you define the intensity more it's the maximum thing like, and not minimum, at least moderate activity they try to do. Do you have any like less of activities that goes under more?

NM

I think it depends on the child is kind of how I would and I didn't want to give like an example because I didn't want to skew the …

PT1

The mod is with assistance and also they are able to do not to vigorous activities like not like other activities which they cannot tolerate so because they don't we have to think about the fatigue levels, the endurance to keep up the endurance activities maybe I would like to do that so that told me that intensity like for them

NM

And that’s how you measure, you measure by Endurance?

PT1

by endurance and fatigue level They can rate it like using the visual analog scale. They can rate it like how much day per day they are or on Pain, or even pain, is also a major factor for these kids because so they don't do a lot and they also have some contractures and some tightness and some deformity is already there, so that may be preventing them to participate more, so that we can also have a pain scale to measure them, to see if they are fully able to do it to that actively which was given I would say, like frequency, like you mean for that session, or for that week

NM

Just in general… how would you measure it for you could be for a session or it can be like something maybe if it's tied to a goal I don't know you tell me how you would how would you measure it ?

PT1

in this matter of life or it's not going to start working with a student on the stationary bike or the adaptive tricycle using the heart rate oxygen levels also like the fatigue levels these are the three things by trying to measure them

NM

And yeah, that's actually very helpful. Thank you.

Yeah. Oh, you said heart rate, fatigue level and pain.

PT1:

pain with my oxygen saturation make us some kids have their they are very tired and the oxygen drops off most of them have like CPAP or some kind of ventilation support for them so that's why I like I use heart rate and also saturation levels

Okay. Yeah. And but if i'm doing like strengthening, that's the hard part we don't know we don't have higher hand dynamometer or something to measure the strength. But i'll see, like how many try how many repetitions there it if i'm doing like some sort of strengthening like sit ups from the mat like with the hand held support. If they're able to do. Sit up, then I see the repetitions. How many times they are able to do like, sit to sit, to stand transfers. If that is a strengthening activity, then I will measure like, okay. They were able to do like 10. The session next session, if they increase to 15, that's the second trial. So then I will measure that the number of repetitions they did.

If i'm doing like, for example, say a standing like sit, to stand and stand there for like with models or whatever. Then I measure the time. How many seconds, or how many minutes they're able to maintain the stance if i'm working on sitting balance. Then again, I use the time by how many minutes they can sit with the min muscles and amount of assist. that's the time is I don't like you can use it. Stop watch or a timer, or like whatever. Yeah.

NM

thank you do these children need assistance you gave me examples of biking, sit to stand and you said they do need assistance so is there any other activities that you want to include and what kind of assistance do you do they really need to do some of these tasks

PT1

I would also add gait training like gait training or like we have tricycle and a pool at school we have like we have you're not fully into pool right now those activities like in based on them I came if I'm using those activities I go over there like how much distance they can walk using the trainer like how many steps pick up making a minute or like how many steps did I take take does much are faster than a normal level of consistency like every time I use their heart rate and how how far they are like endure in the pool activities compared to land activities, like stander is not a functional activity is just like an Adaptive positioning system so that if they are like going incrementally

Like five minutes because of the contractures they may not tolerate like standing for the whole one hour but sometimes and if that reach like 30 minutes or 40 minutes tolerance level again like I use that as a progression for them so I can use the timer like how many minutes they can stand in the stander and also I use the heart rate and oxygen because sometimes they may have like a drop in the heart rate and they were in an upright position so use that and the bone density we cannot measure it but I know so we can make that weight on both feet , pressure measurement ) and standing we can measure that too but we don't do another school that that's an option to do

NM

thank you do you think these children should participate in more or less of the activities that you mentioned

PT1

I would say more and we also have to keep in mind that energy expenditure I gave in when you're measuring energy expenditure you go over the oxygen saturation levels and heart rate I get over there and also their fatigue level they are very fragile because of low bone density if you're not too much of activity at the time but they should be participating in more sessions with sufficient amount of break time or rest time like at least they twice a day in the week, we can do some kind of activity for this much of time so that they are not like doing at a stretch 60 Minutes in one session and making them more fatigue

if it's like a functional task

NM

thank you alright another question do you address promoting physical activity during PT sessions?

PT1

Yes, I do. So I i'll. I help them like with bed mobility activities which they need. That's the main thing, because it's hard for the caregiver to change them. So we always encourage some kind of bed mobility the activities, at least they maintain that level. and then sitting so they can participate with their peers like the alternative seating position during the classroom are with the family at home like sitting and doing some activities today on different levels of surfaces and also standers which may encourage a lot and then reaching like sit to stand transfers

whatever they are doing in the rest of the day. So, we promote every day the same thing, and also training depends again how fatigue they are at that time. If they're too fatigued, we reduce the number of activities, and we now we increase. I encourage more a functional like reaching, standing and sitting balance and everything.

NM

thank you and you said yes what components of physical activity are you addressing during these task. Yeah you mentioned cardiovascular endurance, muscle activation and energy expenditure so those are some things that you've already mentioned

PT1

I would I would like to say more endurance because that will keep them active more during the day, they’ll energy levels are also less they may not participate during all those other things the rest of the day so the endurance is the main thing which I want to address in these kids, so they can be fit because they are so at some point, than strengthening them.

NM

so you're saying I just want to repeat you said endurance keeps them Fit versus versus strengthening them or more than strengthening them?

PT1

Versus because strengthening is important but if they're not endurance like if they cannot say if they're feeling like a participating, we cannot do the strengthening activities, so I figured go first with endurance. Then I go to the strengthening activities, so I got a couple have a balance of activities they don't get fatigued again addressing something else pain and everything

NM

thank you alright do you address a promoting physical activity that occurs outside of your physical therapy session?

PT1

Yeah depends on the caregiver because these kids always needs maximum assistance to do that why we teach the family, caregiver to do some of the activities like if they can go like on a bike but everybody doesn't have a bike so we teach them some kind of bed Mobility activities or like some transfers if they can do it that they have otherwise like I don't think it's not it's not feasible for the caregiver like a fully dependent. So that's what I feel that's my challenge too, to make them to do all sort of outside of the school like what they can do

NM

have you recommended any Community programs or events the students to help increase physical activity?

PT1

Oh, yes, like actually some of my kids they go for like a one of them after school they go to. They get extra physical therapy sessions, but also they go to the park. We take them to the park. They can, we tell them to use the adaptive swings which are in the park, and then, like again, the the child, the caregiver has to support them

in the parks and some of the students they go for like an adaptive dance classes. No, they take up that, and the pool activities. And if this, if the that's what we we hear from the caregivers, so it's not through school, but they are already enrolled in there.

NM

Okay? So the parents, the families are already enrolled in.

PT1

Yeah, they have like adaptive dances. Adaptive like pool aquatic therapy. They take, and also they have like fit. Some of the adult students like who are like about 18. They already have an extra PT. To at home like to get some kind of strengthening or keep them functionally mobile. And while they are within the home

get some kind of threatening or keep them through their insurance I'm thinking through skip through New York so they can do extra activities apart from what they get

NM

its called skip New York?

PT1

Yeah Skip New York yeah

NM

have you share these Resources with any family?

PT1

I have shared many resources like whatever I know who like a like a adaptive dance school so it should be know and we encourage them to go… and if we have like or something like walks like they can take their adaptive equipment from school like a generous or like gait trainer it might be hard for this but they can use on a tricycle so the parents would love to do that take those challenges like we take the equipment from school and then they use it during that so that's what I like to do with them when I am there because parents feel less of a burden so be ready to have a therapist and so during those activities

NM

thank you what type of equipment have you recommended to help improve home or Community engagement or physical outside physical activity outside of the clinical setting?

PT1

So first thing I encourage them to use the standers first, because, even if they are not doing anything, they can go on stander for 60 minutes and the day and we train the parents for that if they are able to do it. And hoyer lift, or hoyer lift Is again the main major equipment. First, before I go for stander, the hoyer lift helps them to transfer. They must be comfortable in transferring the child, because each age group has different weight and parents cannot be flexible and cannot transfer them all by themselves.

The hoyer lift would be the first one then I'll tell them to use the Stander if they have if they have are able affordable to buy the adaptive tricycle and we teach them how to position in the tricycle then they have one at home. We also have a motorized stationary tricycle. Apart from that

that I don't recommend gait trainers because it's hard for the families to use it at home. So.. if the student is having more like cognitive ability to use their power wheelchairs that would be one thing I would also tell them, train the student at school and if they are good and they can use the power wheelchair within home or if they want to go outdoors and it should be accessible for them if the home and if its not accessible for them and there's no point getting on the family It depends on the family and their storage space and also how flexible the parents are ready to spend time to do this, do these activities if if they don't have an extra caregiver to support them I don't think they get enough training after the school

especially I was say during the Covid we had virtual sessions that worsen them a lot being at home than compared to being at school

NM

thank you all right so now we're in the second part of our interview and this is all

this is about a scale it's called The Promise parent proxy physical activity scale are you familiar with this scale yes I have heard about it because I was using a pain scale for my project promise Camp to I know like it has been used for Sleepy Kids yes okay great so I have a question so this one is a question I'm going to ask you how appropriate is the question to addressing physical and political activity intensity in children with CP level 45 okay but I'm going to ask you to write the question first so

on a scale from zero to 500 is not at all related five is highly appropriate for what I'm a go ahead and do is show on the screen so you can get a chance to look at it that will help

okay so this is the scale and so have to take a moment to look at the questions and then the parent or the caregiver

will answer basically on how many days they believe that's family for this child does the the the item right all right so that's how it gets killed, so that's the first thing I want to go over and then I'm going to ask you and I'll agree each question and then I'm going to ask you how really yeah so our population right so is you know 025 + 0 is not a it's not at all related to our population 5 is that is highly appropriate you feel like this is a question and then I'm going to ask you why you why you answered how you answered okay so the first question is how many days did you sell exercise or play so hard that his or her body got tired of zero is again not at all appropriate five is highly appropriate

comforting because they give me a number for can I have a I have a question before I answer you so I mean like the question means like is a child playing Independence Day in the pastures or without a systems like it can be with or without I think this is not at all appropriate

and why

friend staying at home environment parents should be available to bathe them to stop to a system to play or do an exercise time if they have the time to tattoo to be involved with the child then I think the child can participate so most of the time I hear from your parents they are not having a tough time because of all the issues and that's why I think it's not relevant and the other caregiver is not motivated enough to do so most of that must have thought I might stay there on the bed rather than doing some kind of exercise

yes it does. But this is a question a parent or caregiver at their biscuit answer to so how many days if a child exercise or play so hard that his or her body got tired you don't think this is a good question because you think the parents

they have to support they have to ask this time all the time so it's like you're still not I mean

PT1

it all depends on the family too and it depends I like that you know depends and a parent depends on the parents okay so the second question

NM

how many days did your child exercise really hard for 10 minutes or more zero is not at all related for this population of five you can you feel like this is appropriate it can be any of the number 0 1 2 3 4 5 5

PT1

I please play some kind of putting them two sitting on the bed so they can do that it's not that they have to do it this is a scale that they write they just a great their child so like they don't have to do these things to grade it this is like if I gave you a survey like you not leave the PD it out till de parent would fill it out and say how many days during the week typically does my child

read exercise so that body is tired or exercise for 10 minutes how many days in a week so it's just that I'm writing you down based on what they think their child does in a week from the parent I would say this is appropriate you can do you think this is a good question to a parent yes I would say a question to the bedroom light intensity yes thank you how many days did your child exercise so much that he or she breathes hard

it's appropriate or is it a 01 because of

PT1

we need to know like how a different level as like to do the activity so I think we should know why it's good to ask that question to the parents so they know the level of the entrance to participate in an activity at home

NM

great all right number four is how many days was your child so physically active that he or she sweated

PT1

I mean this is something tricky question because

they're not doing activities rigorously first of all so I mean this mine does not mean I would say right like I said three and a difference on the activity because the kids who cannot maintain the temperature they even suspect even for like just transferring from the back to stick my feet out Masons like the sweat on the forehead so I don't know actually might be a difference in their body than and Recreation

NM

thank you alright number 5 how many days did your chop exercise a place so hard that his or her muscles burned

let's keep one has like a Freddy word means fatty you know likes or Doms delayed onset muscle soreness

PT1

so I mean that's not I would rate that game 3 because we don't know how vigorously they will do with that home to get fatty it might be activated are doing there they may not do like a strengthening activities like sit to stand transfers to do so I might be like 3 because if they're having a doctor been like a dad to try cycling the truck so that may be a fatty if they can feel fatigued but again if I should not make them sore like I was at 3

Bankrate has a little light gray gray 4Runner

NM

got you okay all right number 6 how many days did your child you know you said this was number three number 5 was 33 how many days did your child exercise or play so hard that he or she felt tired the same as the first question okay 0 yeah I mean I see the same question the first question is the same as much like the first question in the survey looks like the same thing I'm not sure

PT1

thank you for that you're bringing the other Outsiders interesting they are similar how many will how many days and you're trying to find a place to hide that has everybody got tired

his and her got tired of the first one and then the other one was tired I think so I mean I'm sorry to change my answer for this question so tired I see what you're saying it's got tired place are they got tied out of the different you're right it's relevant to them because they say I cannot do this even if they do a little activity because of fatigue

NM

so what number would you give this one I would say like

PT1

five oldest ones highly appropriate child if they feel fatigued. We actually lie to me she was a different one to make them

NM

got it okay thank you I'll number 7 how many days

was your child physically active or 10 minutes or more

I think it's relevant because we have the parent has to keep them moving so that they don't lose what they already have

NM

what number would you give this one and then the last one is how many days is your child run for 10 minutes or more

PT1

I think it's Sarah why I mean it's not appropriate based on the physical level and greater grade four and five right anything else you would like to ask about the physical activity in this population I would like the eyes like the pain to express them if they're doing something I mean I'm not sure if muscle soreness sometimes they may have stress fractures happens during the activities are like the hip pain was just saying because they're not doing more of like an astute sitting and standing activities and we already know

already have an issue and should be able to say that like better their child is feeling any pain all so I maybe already have the 50 question yeah I like house that has she found the tire so that's a problem maybe we should have a pain question to address exactly where things frequency after that too late

NM

well that concludes our interview thank you thank you thank you I am so glad that we were able to do this today and I'm going to conclude our wedding
