# Supplementary material for: Validity of PROMIS® Pediatric Physical Activity Parent Proxy Short Form Scale as a Physical Activity Measure for Children with Cerebral Palsy Who Are Non-Ambulatory
Source: Behav Sci (Basel). 2025 Jul 31;15(8):1042. doi: 10.3390/bs15081042 (PMC12382615; doi:10.3390/bs15081042)
Supplement: Supplementary file 1 [file behavsci-15-01042-s001.zip › Transcripts copy/PT transcripts - deidentified/PT17.docx]

WEBVTT

1

00:00:01.710 --> 00:00:02.500

Oh.

2

00:00:04.790 --> 00:00:10.069

NM: all right, Thank you so much for joining us today. we are going to

3

00:00:10.460 --> 00:00:20.759

NM: talk about physical activity for children with Cp. Who are at Gmfcs levels 4 and 5, meaning they're not full time walkers. So I do have a couple of questions for you.

4

00:00:20.770 --> 00:00:38.120

NM: and we're gonna start with a little information about physical activity and your opinions and and clinical expertise, and there's no right or wrong Answer. So just feel free to answer freely on the second half of the interview. I'm going to share the promise parent proxy, survey with you, which was

5

00:00:38.130 --> 00:00:57.780

NM: specifically this survey was developed for children that were not typically developing or going through, or had to acquire some disability. And so, we'll talk about that for the second half, If it sounds like I scripted is because I am to be as soon as possible. Each question will have a few prompts or follow up questions.

6

00:00:57.790 --> 00:00:59.779

NM: and we'll start with Number one.

7

00:01:00.250 --> 00:01:06.630

NM: How do you define physical activity for children with Cp. Who are not full Time Walkers?

8

00:01:07.160 --> 00:01:12.230

PT 17: That is a great question. How do I define physical activity?

9

00:01:12.960 --> 00:01:23.889

PT 17: I think it's any type of movement or purposeful interaction that our patients can participate in, and I I define it.

10

00:01:24.160 --> 00:01:31.339

PT 17: I believe it's with assistance or without assistance. It's what's meaningful to them and their family and their caregivers. What's

11

00:01:31.480 --> 00:01:36.720

PT 17: you know, I think, and also part of that definition has to include what

12

00:01:37.840 --> 00:01:51.070

PT 17: is like what their day to day looks like. so you know it's gonna be different for every child. But I think just the encouragement of movement, and what, at whatever level that the child is able to to do.

13

00:01:53.880 --> 00:02:05.579

NM: the department of Health defines physical activity as any activity that encompasses energy expended, and activation of skeletal muscle. Does this definition change your mind about how you define PA?

14

00:02:06.540 --> 00:02:11.920

PT 17: No, I think you know that definitely adds to it gets a little bit more to that. The

15

00:02:12.970 --> 00:02:25.640

PT 17: that skeletal muscle part of it. But I think it's incorporating. What I was saying is that just them being able to move in whatever way they can. that's not. You know, more than just every sign.

16

00:02:26.890 --> 00:02:33.430

NM: Great thanks. How do you think physical activity differs from other types of fitness activities.

17

00:02:34.300 --> 00:02:34.980

PT 17: Oh.

18

00:02:36.850 --> 00:02:45.119

PT 17: i'm not sure that I really thought about that. How does physical activity the differ than fitness? I think fitness might be more of like

19

00:02:45.280 --> 00:02:56.409

PT 17: higher exertion Rates, maybe increased heart rate for fitness, but for physical activity is just really moving throughout the day. Meaningful movement.

20

00:03:02.610 --> 00:03:03.390

NM: Great.

21

00:03:03.440 --> 00:03:09.770

NM: When do you witness your students? participate most in physical activity during the school day?

22

00:03:10.160 --> 00:03:14.079

NM: Or if they're not your students? When would you expect these children to participate

23

00:03:14.200 --> 00:03:16.789

NM: most in physical activity during the day.

24

00:03:18.100 --> 00:03:20.230

PT 17: I find where it's

25

00:03:21.190 --> 00:03:49.569

PT 17: it depends, but primarily where there is some type of social component. So where you know, unless you just have a intrinsically motivated or just a child with high movement that will choose to move on their own. If you have a child that's more involved physically. when they will be more engaged, as when they have caregiver family, member, interaction, peer, interaction, and just that encourage and and encouragement for that movement.

26

00:03:53.730 --> 00:03:54.570

NM: You

27

00:03:54.960 --> 00:03:59.090

NM: Okay. Next question: how do you measure physical activity.

28

00:03:59.170 --> 00:04:08.750

NM: frequency, intensity, time and type? So i'm citing this fitt principle here in children with Cp. Who are not full time walkers.

29

00:04:09.520 --> 00:04:11.200

PT 17: Can you repeat that one more time?

30

00:04:12.040 --> 00:04:12.890

NM: Not a

31

00:04:13.160 --> 00:04:16.130

NM: How do you measure physical activity, frequency.

32

00:04:16.209 --> 00:04:18.759

NM: intensity, time and type.

33

00:04:19.060 --> 00:04:22.109

NM: and children with Cp. Who are not full time Walkers

34

00:04:22.230 --> 00:04:24.320

PT 17: got it.

35

00:04:24.560 --> 00:04:37.379

PT 17: It's looking at what they can do. So what are their transitional moments? So what are their? Can they roll? Can they sit? For then, looking at for how long? Even while at

36

00:04:37.440 --> 00:04:48.320

PT 17: identify what tasks they're doing when they are doing those things. So if they're rolling to play or rolling to get someone or rolling for transfers in the bed, so

37

00:04:48.670 --> 00:04:58.840

PT 17: identifying how often they have to do that throughout their day. How often they do it, and for what purpose, and if it's something that has longer endurance.

38

00:05:00.460 --> 00:05:11.569

PT 17: incorporated it in it like sitting with support or something like that. Then just kind of timing it to say, You know they can do this for 5 min 30 s. Whatever that number is

39

00:05:15.020 --> 00:05:18.609

NM: great. Do they need assistance to complete these activities?

40

00:05:19.640 --> 00:05:21.020

PT 17: Physical activity?

41

00:05:21.520 --> 00:05:27.869

NM: The yeah, Even some of the you mentioned rolling transfer do they need assistance to complete some of these activities is physical activity.

42

00:05:28.000 --> 00:05:32.790

PT 17: Yeah, sometimes they do, sometimes they do for full

43

00:05:33.340 --> 00:05:34.840

PT 17: implementation. Yeah.

44

00:05:35.990 --> 00:05:38.679

PT17 : And you know, identifying that, too.

45

00:05:40.300 --> 00:05:42.320

NM: So would you say,

46

00:05:42.400 --> 00:05:45.609

NM: Which activities do they need the assistance?

47

00:05:47.240 --> 00:05:50.960

PT 17: What do you mean? Like fitness or activity?

48

00:05:51.070 --> 00:05:57.770

NM: Physical activity. Where would you. When would you say they need assistance with some of the things you mentioned? Or even if you want to give some examples.

49

00:05:58.340 --> 00:06:10.180

PT 17: I mean, it really just depends on the kid. But a lot of times the assistance is needed,in more of the upright position so supported Sitting, supported standing.

50

00:06:10.300 --> 00:06:13.009

PT 17: depending on our age. It could be rolling

51

00:06:13.030 --> 00:06:17.460

PT 17: any any movement from a static position to a

52

00:06:17.610 --> 00:06:34.980

PT 17: the next static position, you know, like those dynamic components so primarily depending on the age of the child. It could really just be, you know, guarantee that they'll need help in those upright standing position, but they may or may not be help in sitting but then the transition from sitting into a quadruped, or something like that.

53

00:06:36.000 --> 00:06:39.039

PT 17: But then, if they're young, then they might need to help with rolling.

54

00:06:39.530 --> 00:06:48.069

PT 17: or the guide, like the support to reach and play. So kind of show them what we're asking them to do.

55

00:06:50.100 --> 00:06:54.739

NM: And do they need assistance during the entire task? Or would you say part of the task?

56

00:06:55.810 --> 00:07:14.089

PT 17: the way I like to look at it is, I don't want to give assistance, you know, all the time, maybe the first couple of times, and there's something that they're just not familiar with, and I guide them through it. But my expectation for physical activity is that maybe they need some support.

57

00:07:14.360 --> 00:07:15.040

PT 17: but

58

00:07:15.120 --> 00:07:17.169

PT 17: that if it's something that they need

59

00:07:17.340 --> 00:07:19.310

PT 17: support the whole time that I don't.

60

00:07:19.550 --> 00:07:37.789

PT 17: I'm not sure that that's physical activity. that might be more of a like a passive range of motion kind of thing, or modeling and moving them and showing them what you want. But for it to be true physical activity, I think there needs to be, you know, as least support as possible, but not a total assist

61

00:07:42.270 --> 00:07:48.699

NM: great. Do you think they should participate more in more or less of these activities, and why?

62

00:07:49.510 --> 00:07:51.419

PT 17: I think whatever’s meaningful.

63

00:07:51.470 --> 00:08:05.099

PT 17: sometimes you put a quantity on something, and it doesn't mean anything, then they may not truly be engaging. So I think, meaningful activities whatever that quantity is, or whatever that quantity is, that they are

64

00:08:05.150 --> 00:08:10.410

PT 17: parts of that they're doing and then hopefully that they're initiating as well.

65

00:08:11.670 --> 00:08:13.630

PT 17: even if they need support and help.

66

00:08:16.740 --> 00:08:17.470

NM: Great.

67

00:08:17.710 --> 00:08:22.889

NM: All right. Next question. Do you address promoting physical activity during your PT sessions?

68

00:08:23.210 --> 00:08:24.410

PT 17: Absolutely.

69

00:08:24.870 --> 00:08:30.660

PT 17: I always, you know, from the beginning first interaction, just figuring out what their day looks like. What are the

70

00:08:30.670 --> 00:09:00.659

PT 17: things that the family likes to do or engage in? What is the things that the child likes to do. And as we work on moving, just reminding and highlighting and sharing with families where they can incorporate the things that we're able to do, or what this child can do by themselves. Do more of that, you know. Yeah, i'm working on some of these higher level things, because I want to start working on the you know the the strength and alignment and and exposure to it. But, hey, family, if they're able to do this rolling.

71

00:09:00.670 --> 00:09:18.920

PT 17: and they'll roll around the floor all day. Let them let them get down there and set up an environment where they're more, you know they they can go and get to the things that they want, the toys that they like the remote to tablet the tv. Whatever it is, it's gonna get them to move then. Yes, more of that. And

72

00:09:18.930 --> 00:09:22.839

PT 17: when it's meaningful, it has a

73

00:09:23.790 --> 00:09:26.109

PT 17: it helps with that neuroplasticity

74

00:09:29.860 --> 00:09:46.329

NM: great. Now you said, Yes, which is great. How do you do this? in your actual session? So you told me how you have with the parents, and find what they do in your day and day. but when you're actually doing physical therapy with this child, what components physical activity do you address

75

00:09:48.520 --> 00:09:51.710

PT 17: when we're

76

00:09:51.900 --> 00:09:54.299

PT 17: It's probably like in between

77

00:09:54.790 --> 00:10:04.050

PT 17: the therapeutic interventions, when the child might be doing some active rest, just trying to continue to encourage them to move, observing movement when i'm

78

00:10:04.350 --> 00:10:09.349

PT 17: in between the in the intervention that i'm doing

79

00:10:09.470 --> 00:10:11.790

PT 17: and just kind of seeing what they're

80

00:10:11.940 --> 00:10:14.300

PT 17: willing and able to do on their own

81

00:10:15.350 --> 00:10:18.920

NM: Umm let me see what you say between intervention. So

82

00:10:19.000 --> 00:10:22.810

NM: when in your session, are you actually promoting physical activity.

83

00:10:24.750 --> 00:10:35.450

PT 17: I would say throughout. So if I have so, ---, the way I try to plan out. My intervention is i'll see a kid For an hour, and

84

00:10:35.680 --> 00:10:52.139

PT 17: I try to chunk, you know, for 15 min I might work on this, or, you know, between 5 and 15 min, depending on what the child can tolerate. So where I might be working on some facilitated rolling with the Kid to just work on their ability to do that independently, or going from

85

00:10:52.150 --> 00:11:17.139

PT 17: supine and prone into sitting position, working on weight bearing to the upper extremities. When I take a break from that, and the child just needs a moment. Then, just trying to encourage that active rest of okay, you can still play with this toy. You can move as you want to move Until we then transition to the next thing, so probably more in transitions between the

86

00:11:17.190 --> 00:11:20.730

PT 17: the activities that i'm working on to help them with their goals.

87

00:11:23.530 --> 00:11:24.320

NM: Okay.

88

00:11:24.430 --> 00:11:28.890

NM: so and I do have some examples, for example. So if you're working on

89

00:11:28.910 --> 00:11:48.009

NM: physical activity, as you mentioned, which will be in the during these transitions, what are you? What components? So some of the examples I give the therapist are like cardiovascular endurance, muscle activation, energy, expenditure. What components of physical activity are you addressing when you're doing it when you're addressing it in your therapy session.

90

00:11:49.650 --> 00:11:51.410

PT 17: Give me those examples again.

91

00:11:51.940 --> 00:11:53.620

NM: Cardiovascular endurance.

92

00:11:53.650 --> 00:12:12.439

NM: muscle activation, energy, expenditure, and mobility. There's so many, you know.

PT17: Yeah. Yeah. So probably not so much cardiovascular, indirect. I feel like that might be more of what i'm doing in my treatment. Intervention for fitness, I would say, but more like muscle activation, mobility.

93

00:12:14.320 --> 00:12:25.430

PT 17: there was something else you said. Tell me those options again,

NM: and energy expenditure,

PT17: Energy expenditure, just to kind of see what it takes them to do to be able to move on their own

94

00:12:27.350 --> 00:12:28.850

NM: Gotcha.

95

00:12:33.500 --> 00:12:35.290

NM: And

96

00:12:36.060 --> 00:12:40.810

NM: no, that's what I was gonna say. If you don't work on, let's say one of those components, why would

97

00:12:43.320 --> 00:12:48.299

PT 17: it would probably just be that the child couldn't handle it. but

98

00:12:48.420 --> 00:13:05.570

PT 17: always knowing the value of the multi-systems assessment, and how, though the all the systems when they're functioning at their best, the child will function at their best. So if, for for example, they're having a hard time, just

99

00:13:05.630 --> 00:13:15.769

PT 17: you know they have increase heart rate or respiratory rate with these activities. Then maybe I don't push that but trying to incrementally work toward that.

100

00:13:17.140 --> 00:13:17.810

PT 17: Yeah.

101

00:13:18.240 --> 00:13:24.650

NM: okay, great. All right. Do you address promoting physical activity that occurs outside of your Pt. Session?

102

00:13:24.970 --> 00:13:36.090

PT 17: Yes, because that's part of their home exercise program where we'll have better results if they can incorporate this and figure out a way to incorporate it throughout their day to day.

103

00:13:39.120 --> 00:13:44.370

NM: And have you recommended any community programs or events to your students to help increase PA

104

00:13:44.480 --> 00:13:47.410

PT 17: absolutely. so I look at

105

00:13:47.670 --> 00:13:59.460

PT 17: what the offerings are with our local rec center. listening and always thing trying to stay connected with family groups and local community offerings, because

106

00:13:59.630 --> 00:14:04.560

PT 17: when there are other, you know, if it's diagnosis specific or

107

00:14:04.760 --> 00:14:05.780

PT 17: just that, it

108

00:14:05.850 --> 00:14:07.920

PT 17: addresses

109

00:14:09.070 --> 00:14:12.459

PT 17: interaction from 4 children.

110

00:14:12.470 --> 00:14:37.199

PT 17: It helps families to see that as valuable, and then it helps to make the connection for them. What i'm doing in my Pt session for it to hopefully continue so. I'm always looking. I don't care what the level of my child is like what they're if they have Cp. And if you have GMFCS levels it it doesn't matter that they might be a 4 or 5 or one like one through 5. If I can find out like we have a local Rec center that

111

00:14:37.210 --> 00:14:40.409

PT 17: meets the need, that they serve

112

00:14:40.800 --> 00:14:49.459

PT 17: people with disabilities so always sharing that with families like listen, they have programming, they might have some things, and

113

00:14:49.890 --> 00:14:59.910

PT 17: continuing to encourage that mindset for them to that it's not just, you know for what whoever you think this is for is where your kids who is for everybody.

114

00:15:01.080 --> 00:15:02.150

NM: Awesome.

115

00:15:02.480 --> 00:15:06.380

NM: Okay, now it's time to second half show you the

116

00:15:06.550 --> 00:15:08.320

NM: survey.

117

00:15:14.690 --> 00:15:18.130

NM: It's too small. Let me know I can make you a little bit perjure

118

00:15:21.280 --> 00:15:23.790

NM: again. So this promise

119

00:15:24.000 --> 00:15:35.089

NM: parent, proxy. Physical activity surveys this out, and, as you can see, they'll answer for the past 7 days. I'm going to leave this up so you can look at it. What i'm going to do is

120

00:15:35.200 --> 00:15:36.450

NM: each question

121

00:15:36.540 --> 00:15:55.489

NM: how you would rate the appropriateness for children at levels. G. E. G. Functioning at G message Level 4 and 5, and so again a caregiver panel. Fill it out, and they'll report how many days it's. So it's a physical activity, intensity, measure, and so I will go through each question, and i'll tell you

122

00:15:55.860 --> 00:16:14.689

NM: the rating so from 0 to 5 0 being not related at all to this population. Doesn't that you don't think it's appropriate for them, 5 being highly appropriate. Okay, this is a question that makes sense, and that it is applicable to a child at level 4 and 5. Who is not a full time Walker, and then i'm going to ask you Why?

123

00:16:14.700 --> 00:16:20.750

NM: Okay? And so my face validity from Pt's parents. So

124

00:16:21.220 --> 00:16:28.739

NM: first question, how many days is your child exercise or place so hard that his or her body got tired. How would you rate that 0 not related at all?

125

00:16:28.820 --> 00:16:33.519

NM: 5 being highly appropriate or somewhere? And that's that ranking on a scale, and why.

126

00:16:33.960 --> 00:16:39.589

PT 17: I think 5 it's appropriate because it asks the

127

00:16:39.620 --> 00:16:41.970

PT 17: individuals how

128

00:16:43.760 --> 00:16:47.110

PT 17: many like, how many days of this child exercise

129

00:16:47.210 --> 00:16:49.759

PT 17: so far that they got tired.

130

00:16:50.130 --> 00:16:52.099

PT 17: without too many

131

00:16:52.170 --> 00:16:55.200

PT 17: qualifiers.

132

00:16:56.140 --> 00:16:57.150

PT 17: for them

133

00:16:57.890 --> 00:16:59.010

PT 17: like, I think

134

00:16:59.220 --> 00:17:00.690

PT 17: I think it's applicable.

135

00:17:01.590 --> 00:17:02.330

PT 17: Yeah.

136

00:17:02.800 --> 00:17:04.060

NM: all right. Number 2.

137

00:17:04.390 --> 00:17:09.950

NM: How many days. Is your child exercise really hard for 10 min or more? 0 not related at all?

138

00:17:10.670 --> 00:17:14.560

NM: Up to 5. Highly appropriate. How would you write this one, and why?

139

00:17:14.910 --> 00:17:30.569

PT 17: I think this is kind of speaking to the point of why I gave the first one to 5. I think this one might be 2 because of then these qualifiers of 10 min. I think that then starts to limit the

140

00:17:30.660 --> 00:17:34.919

PT 17: who this is eligible for

141

00:17:36.670 --> 00:17:39.329

PT 17: I think that number. And then

142

00:17:39.360 --> 00:17:42.660

PT 17: you know, really hard. What what does that mean? That

143

00:17:42.750 --> 00:17:44.410

PT 17: I think that

144

00:17:45.410 --> 00:17:46.670

PT 17: it like

145

00:17:46.720 --> 00:17:55.070

PT 17: if we're comparing it to the first one, it says so hard that your body got tired. Well, this one just says really hard. For 10 min it still is up for interpretation.

146

00:17:55.320 --> 00:17:56.020

NM: Okay.

147

00:17:56.890 --> 00:17:59.009

NM: alright, thank you. Number 3.

148

00:17:59.150 --> 00:18:03.510

NM: How many days is your child exercise so much that he or she breathe hard?

149

00:18:03.650 --> 00:18:11.200

NM: How would you rate this as it relates to children at levels one of 5 0 not related up to 5 highly appropriate

150

00:18:12.050 --> 00:18:12.790

NM: one.

151

00:18:14.300 --> 00:18:15.730

PT 17: I think it's.

152

00:18:16.000 --> 00:18:17.620

PT 17: I would say,

153

00:18:19.070 --> 00:18:20.090

PT 17: Hi, there.

154

00:18:21.160 --> 00:18:22.580

PT 17: hold 1 s

155

00:18:25.640 --> 00:18:27.739

PT 17: Okay, okay, sorry.

156

00:18:29.060 --> 00:18:32.990

PT 17: I would say. Probably a 4.

157

00:18:33.300 --> 00:18:34.150

NM: Okay.

158

00:18:34.570 --> 00:18:40.080

PT 17: And just knowing the population. That breathing hard

159

00:18:40.160 --> 00:18:54.490

PT 17: sometimes may or may not indicate that they are exercising but if it is that they were reading, if they were really hard because of movement, then I think it's appropriate. Sometimes our patients just have a hard time breathing because of postural

160

00:18:56.470 --> 00:18:57.650

PT 17: and what

161

00:18:58.360 --> 00:18:59.240

PT 17: and strength

162

00:19:01.700 --> 00:19:03.280

NM: All right. Number 4.

163

00:19:03.430 --> 00:19:11.460

NM: How many days was your child so physically active that he or she sweated 0 not related at all. 5 highly appropriate, and why

164

00:19:16.630 --> 00:19:26.409

PT 17: i'm gonna have a hard time with that one. I would say I one or 2, because i'm trying to think about how often I see my Ca patients sweat and pt

165

00:19:26.440 --> 00:19:27.160

PT 17: like

166

00:19:31.600 --> 00:19:34.150

PT 17: So yeah, and I don't know if that's just.

167

00:19:34.410 --> 00:19:35.320

PT 17: I don't know

168

00:19:36.300 --> 00:19:45.089

PT 17: the sweating got me like, do one? Do we work that hard, and then could, with a child do that much working on their own? I don't know that that

169

00:19:45.210 --> 00:19:46.270

PT 17: applies.

170

00:19:48.310 --> 00:19:51.049

NM: And so what was your final answer?

171

00:19:51.070 --> 00:19:53.160

PT 17: I must say a 2.

172

00:19:53.340 --> 00:19:54.070

NM: Okay.

173

00:19:54.390 --> 00:19:55.780

NM: Number 5.

174

00:19:56.150 --> 00:19:57.720

NM: How many days

175

00:20:00.730 --> 00:20:04.779

NM: did your child exercise or play so hard that his or her muscles burned

176

00:20:05.010 --> 00:20:09.550

NM: 0, not related at all. 5 highly appropriate. How would you bring this one?

177

00:20:10.740 --> 00:20:16.610

PT 17: I would say a 0, because I don't know what muscle burn means. and how

178

00:20:16.910 --> 00:20:20.210

PT 17: a caregiver could identify that within

179

00:20:20.510 --> 00:20:21.770

PT 17: a child.

180

00:20:26.590 --> 00:20:32.970

NM: Number 6. How many days did your child exercise a facial heart that he or she felt tired.

181

00:20:34.410 --> 00:20:35.950

PT 17: I think a 5.

182

00:20:36.070 --> 00:20:37.480

NM: Okay, you are

183

00:20:38.610 --> 00:20:50.770

PT 17: because caregivers can identify when you know a child is fatigued, and so they could think back to what what you know. What was this child doing when they

184

00:20:50.880 --> 00:20:52.740

PT 17: got tired?

185

00:20:53.000 --> 00:20:53.660

Okay.

186

00:20:53.700 --> 00:20:59.169

NM: how many Number 7? How many days was your child physically active for 10 min or more.

187

00:21:02.040 --> 00:21:05.000

PT 17: I think probably a one.

188

00:21:05.400 --> 00:21:09.669

PT 17: 10 min is a long time for a child.

189

00:21:10.010 --> 00:21:17.430

PT 17: 4 or 5 to be physically active, without some support and in tension around activity.

190

00:21:17.960 --> 00:21:20.769

NM: What number did you give this? You said 0. Sorry

191

00:21:20.870 --> 00:21:25.769

PT 17: does say a 0 or one

192

00:21:26.860 --> 00:21:28.509

NM: We call it a 10 min. Okay.

193

00:21:32.750 --> 00:21:34.189

NM: Number 8.

194

00:21:35.450 --> 00:21:43.469

NM: How many days is your child Run 10 min or more? 0 not related at all up to 5 highly appropriate. How would you rate this one, and why

195

00:21:43.960 --> 00:21:52.399

PT 17: 0 not appropriate to ask about a child running if they're at that Gmf Cs level, 4 or 5

196

00:21:54.090 --> 00:21:57.619

PT 17: running, or then running for 10 min.

197

00:21:58.540 --> 00:22:04.189

NM: All right. We are at the end. I like to open up for final comments or thoughts, as it relates to this

198

00:22:04.250 --> 00:22:08.499

NM: topic physical activity. Anything you like to share

199

00:22:08.640 --> 00:22:09.550

NM: as we can.

200

00:22:09.950 --> 00:22:11.999

PT 17: Yeah, I think.

201

00:22:13.740 --> 00:22:20.219

PT 17: Holding space for the individual needs of children on these surveys, so like

202

00:22:20.830 --> 00:22:26.559

PT 17: where it was not too specific. It's helpful for families to see them.

203

00:22:27.580 --> 00:22:42.529

PT 17: You know themselves, their child. to truly be able to honor. Answer it honestly. and I think sometimes surveys, especially if you're giving them to families like they're already grieving and dealing with the disability that their child has.

204

00:22:42.540 --> 00:22:55.910

PT 17: and so sometimes these can be hard for them to say, like you know what know my child is not doing these things instead of, you know, really asking like what their child is able to do, but not comparing it to like a

205

00:22:56.020 --> 00:23:10.480

PT 17: the same HP. Or without a disability. and just the knowledge of that population to then be able to ask appropriate questions. If you're going to ask for a comparison.

206

00:23:10.560 --> 00:23:13.930

PT 17: so maybe it's not 10 min. Maybe it's

207

00:23:14.360 --> 00:23:19.829

PT 17: at intervals, or you know less time, because that's more than likely what you're going to see.

208

00:23:21.940 --> 00:23:23.020

NM: Gotcha.

209

00:23:24.130 --> 00:23:27.219

NM: Thank you so much. I'm gonna stop our recording.
