# Supplementary material for: Validity of PROMIS® Pediatric Physical Activity Parent Proxy Short Form Scale as a Physical Activity Measure for Children with Cerebral Palsy Who Are Non-Ambulatory
Source: Behav Sci (Basel). 2025 Jul 31;15(8):1042. doi: 10.3390/bs15081042 (PMC12382615; doi:10.3390/bs15081042)
Supplement: Supplementary file 1 [file behavsci-15-01042-s001.zip › Transcripts copy/PT transcripts - deidentified/PT7.docx]

WEBVTT

1

00:00:00.000 --> 00:00:01.150

In a moment.

2

00:00:03.880 --> 00:00:15.920

NM: Good morning, Dr. EC7 Thank you for joining us today, as we are going to discuss physical activity and children with Cp: and we'll also want to look at a specific tool that looks like physical activity.

3

00:00:15.960 --> 00:00:30.939

NM: and so the first question I have, and i'm gonna ask a few questions for specifically, but I have some prompts for each question, and then we'll do the second half. We'll be looking at the instrument. I I'm: I shared about. Okay. So first question

4

00:00:31.010 --> 00:00:36.510

NM: is, how do you define physical activity for children with Cp Who are not full Time Walkers.

5

00:00:39.530 --> 00:00:41.890

PT7: Okay, I mean.

6

00:00:42.170 --> 00:00:46.190

PT7: The the issue is, you know. Can you… You have to try to

7

00:00:46.320 --> 00:01:16.300

PT7: develop something that gets them to move somehow or another, expend energy, you know, burn calories. That's the biggest struggle. You know what I mean with the you know. 4 and 5, you know, nonambulatory population. So anything that can that can get them moving on their own When I was thinking about this when I was waiting to start the call way back, when I first started treating. We used to use this little device that was made by tumble forms called the jet mobile.

8

00:01:16.310 --> 00:01:35.320

PT7: and the kids would be down in prone, You know in this thing that kind of look like a car, and like the even, the lowest functioning kids could really get some activity in. You know what I mean with that. But you know to me it's got to get them moving on their own, you know, and burning some kind of energy expenditure

9

00:01:37.000 --> 00:01:48.189

NM: awesome. And so the first prompt which is already addressed is the Department of Health defines physical activity is activity that it encompasses energy, expenditure, and activation of skeletal muscle.

10

00:01:48.260 --> 00:01:52.300

NM: Does this definition change your mind about how you define physical activity?

11

00:01:52.470 --> 00:02:02.639

PT7: No

NM: And then the next follow up would be how do you think physical activity differs from other types of fitness activities.

12

00:02:06.710 --> 00:02:08.500

PT7: fitness not therapy

13

00:02:09.320 --> 00:02:23.280

NM: fitness. I guess, when we the fine fitness we talk about more structured exercise kind of goals. Right? So how would physical activity relate, or how does how does it differ from other types of quote, end quote fitness, activity?

14

00:02:24.780 --> 00:02:27.280

PT7: Yeah. Well, it it could

15

00:02:27.290 --> 00:02:45.080

PT7: the more on a one on one kind of thing, you know, like maybe a parent or a caretaker has figured out what motivates their kid to try to move around, even if they're just rolling around on the floor. and so it could be less structured, you know. I think and I think it could

16

00:02:45.200 --> 00:02:54.879

PT7: have a fairly broad encompassing, you know, like just getting a kid in a pool without a structured, you know, aquatic session is physical activity.

17

00:02:57.840 --> 00:03:05.559

NM: Thank you. When do you witness your students for the children you've treated participate most in physical activity during the day.

18

00:03:06.910 --> 00:03:11.969

PT7: So there's been some interesting studies of that as well for actually kids that are

19

00:03:12.110 --> 00:03:21.440

PT7: ambulatory, and they wear up timers and stuff like that. But I think school honestly, it's school. That's where the kids get their physical activity.

20

00:03:21.570 --> 00:03:23.579

PT7: I I would say, by and large

21

00:03:24.560 --> 00:03:35.260

NM: like going to school. So what I what if what what's this? What's this? What if we just be going to school, or is there something specifically at school that you would consider physical activity?

22

00:03:35.680 --> 00:03:37.979

PT7: Yeah. yeah, I

23

00:03:38.320 --> 00:03:47.950

PT7: kind of both. You know what I mean. The process of getting up dressed out of the house in your chair, and off you go, and then you're when you're in school, your level of participation

24

00:03:48.110 --> 00:03:55.140

PT7: it could be of any type is going to be higher, you know. Unfortunately, a lot of the kids listen. If they're home.

25

00:03:55.280 --> 00:04:04.649

PT7: Mom and Dad are probably working, you know. Maybe there's just a sitter, caretaker, type of person who may not be the most motivated person to get them moving.

26

00:04:04.660 --> 00:04:15.819

PT7: and unfortunately most of my kids are that I've treated in recent times is really, you know, Inner City, Brooklyn. Those kids are housebound. They're not going out to the park. It's just not safe.

27

00:04:17.720 --> 00:04:32.949

NM: Thank you. That's a very helpful, all right. Second question. How do you measure physical activity, frequency, intensity, time and type. So i'm holding a fit principle there in with Cp. Who are not full time Walkers

28

00:04:34.930 --> 00:04:37.360

PT7: difficult, difficult

29

00:04:37.380 --> 00:04:38.990

so

30

00:04:39.500 --> 00:04:57.389

PT7: could time it. You know what I mean, you know. Can they stay in an activity for 5 min? 10 min, you know, whatever that may be, for, like an older like in, let's say, an adolescent child who is maybe cognitively more intact like you. Could

31

00:04:57.400 --> 00:05:15.670

PT7: you have a much easier job, right? Because you could put them on an upper body or a ergonometer on an exercise bike. You know what I mean Things like that. So that's easy to measure. It's your kids that are very low functioning both motor and cognitive, wise that it's really quite difficult. I would have to say it would be

32

00:05:15.680 --> 00:05:22.159

PT7: time that they can stay engaged in the activity and potentially numbers of

33

00:05:22.260 --> 00:05:37.919

PT7: skills, things they can do, you know, like, let's say you're just doing a reaching task right trying to get them to move a little bit, you know. Can they only do it for 5 min, you know, or can you get eventually build them up to have a longer period of time with activity.

34

00:05:39.590 --> 00:05:40.670

NM: Thank you.

35

00:05:40.700 --> 00:05:43.400

NM: And based on some of those examples you gave.

36

00:05:43.440 --> 00:05:48.460

NM: Do they need assistance to complete these activities in terms of

37

00:05:48.630 --> 00:05:59.169

NM: Measuring this physical activity, and in you could. You'll give me more examples of activities, and then you can tell me, do they need, if they need a assistance? Do they need it for a part of the task or the inquire task.

38

00:06:00.210 --> 00:06:01.880

PT7: Yeah,

39

00:06:03.820 --> 00:06:14.149

PT7: Oftentimes the kids need assistance to be set up for the activity, you know. So let's the example I just gave of an adolescent who can do a you know ergonometer

40

00:06:14.160 --> 00:06:32.819

PT7: More than likely she's not climbing on it or herself right. So someone's gonna have to assist to get her. You know what I mean. Same thing in that out of the pool, you know, in a seating arrangement, you know. So in getting the child set up, I would say, probably 99% of the time the kids will need that assistance.

41

00:06:32.830 --> 00:06:41.289

PT7: Yeah, unless they're doing something that is just very floor based. You know what I mean where they can, you know, on crawl around, do whatever they can to get moving.

42

00:06:43.510 --> 00:06:55.330

PT7: Great. Thank you. Yeah. And I think that, you know, if you wanted to try to document progress or demonstrate intensity, you know the the fit parameters

43

00:06:55.440 --> 00:07:13.710

PT7: level of assistance would have to definitely be recorded. You know what I mean. Let's say the the child is doing a a cycle, but they get stuck. You know what I mean. They can't make a a smooth rotation with the pedals, and if they need assistance, you could document the level of assistance. How frequently and that kind of stuff.

44

00:07:16.550 --> 00:07:17.540

NM: That's good.

45

00:07:17.880 --> 00:07:24.479

NM: Thank you. okay. And then do you think they should participate in more or less of each of these activities, and why

46

00:07:26.090 --> 00:07:28.030

PT7: more?

47

00:07:28.120 --> 00:07:39.010

PT7: you know, in the AACPDM. it's really all about, you know, activity and participation, you know. So the more the kids can be active more, they can participate

48

00:07:39.310 --> 00:07:51.389

PT7: in their life experience wherever they are. I mean. So I I think it's it's. It's it's it's vital and they need more, and that's the big push, you know, is to figure out

49

00:07:51.510 --> 00:07:58.699

PT7: what to do for children like this outside of therapy to get them. The activity that they need

50

00:08:01.910 --> 00:08:09.679

NM: great. You just you're leading me right into my next question. But before I continue you said the acronym, you said, AACPDM:

51

00:08:09.810 --> 00:08:19.040

PT7: Yeah. The American Academy for Cerebral Palsy and Development Medicine, a. AACPDM: that's what I thought. You may. I just want to confirm. Okay, Thank you

52

00:08:19.150 --> 00:08:21.169

NM: All right next question.

53

00:08:21.410 --> 00:08:27.729

NM: and you let us right here. Do you address promoting physical activity during physical

54

00:08:28.000 --> 00:08:32.499

NM: therapy sessions like within this session, and then we're going to talk about outside session in a moment.

55

00:08:33.400 --> 00:08:40.459

PT7: absolutely because the therapist is going to be the one who's going to be able to hopefully figure out

56

00:08:40.580 --> 00:08:49.390

PT7: what this child can do, and to what capacity you know. So hopefully, the treating therapist will have enough knowledge.

57

00:08:49.570 --> 00:09:07.100

PT7: experience, or energy, you know, to find the way in. There's always a way in with these kids to figure it out. And so that's the job, you know. And if the therapist can do that and get the activity incorporated in therapy. To start, then it can easily

58

00:09:07.400 --> 00:09:15.680

PT7: carry over to outside of therapy, you know, instructing the parents, giving them assistance to say we do this in therapy. You can do this, too.

59

00:09:20.270 --> 00:09:25.300

NM: You said so many golden nuggets. I just want to get some of these and

60

00:09:25.590 --> 00:09:27.410

NM: So that's all good.

61

00:09:27.560 --> 00:09:40.350

NM: If you said well, you said yes, and how you do this? what components, though of the physical activity are you addressing? So you mentioned, you know, finding out what you can do, and really a therapist taking the time

62

00:09:40.360 --> 00:09:48.639

NM: to figure that out and then help translate back to the family. What components of the activity that you figured out that you mentioned above?

63

00:09:48.650 --> 00:10:01.089

NM: What components of that physical activity would you be addressing in therapy? Let me give you some examples. Cardiovascular endurance, muscle, activation, energy. What is? What are the key things that you were looking to address when you're talking about physical activity in a session.

64

00:10:01.350 --> 00:10:16.700

PT7: Hmm. Yeah. Cardiovascular is way up on the top. It's a you know. It's a significant concern, you know, with this population, and it does nothing but get worse as they get older. Just being able to expend energy.

65

00:10:16.710 --> 00:10:26.760

PT7: Calorie burning. We many of our nonambulatory kids get really ‘chubba’ [overweight], which then makes it more difficult for them.

66

00:10:26.770 --> 00:10:39.550

PT7: and and getting the kids to explore, to figure out what they can do, you know, like you know, let them figure out how to experiment with their body. You know, no matter how much or how little it can do.

67

00:10:45.540 --> 00:10:51.669

PT7: and or what they like. You know what I mean, like you may think an activity is just fabulous, and the kid hates it.

68

00:10:53.970 --> 00:10:54.960

NM: That's great.

69

00:10:55.120 --> 00:10:57.370

NM: All right. Next question.

70

00:10:57.450 --> 00:11:04.690

NM: Do you address promoting physical activity that occurs now outside of the Pt session. And how how do you do that?

71

00:11:07.220 --> 00:11:20.770

PT7: if yes, for sure. it's easiest for the ambulatory kids. It's very easy to set up a program for the parents to the to follow, but I think for those, the the lower functioning, the fours and 5 kids.

72

00:11:23.290 --> 00:11:28.499

PT7: I had a thought in my head, but it left me, but I I guess what i'm having is to try to

73

00:11:32.680 --> 00:11:49.760

PT7: have. Try to if you've got to get to the parent to figure out what they have in their resources, you know, to try to get the kid, you know, physically active. So I have fun working with the wii

74

00:11:49.770 --> 00:11:57.199

PT7: and so a a nonambulatory kid can really do the Wii [Nintendo set] You know they sit on it.

75

00:11:57.220 --> 00:12:09.370

PT7: you know. And so that's I mean it's not popular now. But that would be a easy thing for a parent to incorporate at home, you know, to get physical activity, you know, going.

76

00:12:10.380 --> 00:12:14.010

PT7: It's a really a knowledge, sharing, and

77

00:12:14.090 --> 00:12:21.650

PT7: resources they have either in their home or in their community. That's the other option is that you know the

78

00:12:21.740 --> 00:12:36.779

PT7: the push with these kids is to get them involved in community activities, not just special therapeutic kinds of activities. And so here, even in New York City. We have a swimming program, for.

79

00:12:36.830 --> 00:12:50.530

PT7: you know, kids with impairments, you know, and so probably a level 5 kid wouldn't work in that environment. Not enough. But it's the therapist. It's our responsibility to be knowledgeable and get them to the right place.

80

00:12:50.760 --> 00:12:51.680

Okay.

81

00:12:53.660 --> 00:13:00.339

PT7: Yeah. For years Ucp United Cerebral palsy ran a Saturday program, and

82

00:13:00.380 --> 00:13:10.079

PT7: you know they they they loved it, you know, folks that were involved with it. They did. We had this one guy. He was diplegic, but severe. Non ambulatory.

83

00:13:10.280 --> 00:13:12.429

PT7: He used to bench Press 400 lbs.

84

00:13:14.240 --> 00:13:19.260

PT7: Okay, and went on to special Olympics. Yada Yada, but it's things like that that you can find.

85

00:13:21.920 --> 00:13:29.800

NM: So that leaves me right into my next prompt. So have you recommended any community programs or events specifically to help increase

86

00:13:29.820 --> 00:13:32.640

NM: physical activity in the families you work with.

87

00:13:33.070 --> 00:13:42.060

PT7: Yeah, Absolutely. Yeah, for sure. The New Year program you mentioned Ucp had programs. yeah. Yeah.

88

00:13:42.610 --> 00:13:44.620

NM: Any events that you recommend?

89

00:13:47.700 --> 00:13:48.480

PT7: i'm

90

00:13:49.380 --> 00:13:55.260

PT7: i'm i'm i'm drawing a blank. You know what I mean at the moment. but

91

00:13:55.650 --> 00:13:57.219

PT7: I know that

92

00:13:57.580 --> 00:14:09.180

PT7: I maybe I don't know. I'm. I'm. Not a 100% sure of, you know, you know, like events, you know. One thing, though I have done a lot is refer parents to the

93

00:14:09.440 --> 00:14:12.169

PT7: equipment. Expos

94

00:14:12.240 --> 00:14:16.220

PT7: because in the equipment exposed they can maybe find

95

00:14:16.480 --> 00:14:20.050

PT7: something that will help to get their kid moving. You know.

96

00:14:21.370 --> 00:14:35.050

NM: Wow, it sounds like, you know, my script that went so. The next question is, what type of equipment have you recommended to help improve home? Not just community now home and community engagement of physical activity outside of

97

00:14:35.140 --> 00:14:37.559

NM: your actual Pt session. You know

98

00:14:37.710 --> 00:14:39.009

PT7: our clinical setting.

99

00:14:39.250 --> 00:14:49.380

PT7: Yeah, I mean, you'd love to put a body weight support device in everybody's house. But it's not happening. but you know.

100

00:14:49.980 --> 00:14:54.539

PT7: getting kids upright in standers. You know what I mean, I think is really

101

00:14:55.320 --> 00:15:12.310

PT7: really vital. You know what I mean. It's good for their general health. And well being, you know what I mean, all their you know systems. And so yeah, figuring out a way to get kids to be upright, you know, I think is really important. And whatever type of equipment you can do, you know to do that.

102

00:15:12.400 --> 00:15:30.719

PT7: you know. Unfortunately, we don't have usually enough resources to get like really cool stuff like a nice power chair that will, you know. Take the kid from sit to stand and stuff like that. But yeah, I I think any kind of thing that you can do to get the kid to be upright, I think, is really important.

103

00:15:34.290 --> 00:15:36.030

NM: Great Any other equipment?

104

00:15:36.400 --> 00:15:49.579

PT7: What we go to the last portion you mentioned how wheelchair you mean the power, the the to stand feature.

PT7: Okay, Great: yeah, yeah. And you know, you can also think, really, basically you know, like.

105

00:15:49.600 --> 00:16:01.169

PT7: how about you put a cuff weight on the kid's arms, you know, and they throw up all around. You know what I mean. You're going to create their work output even as something as simple as that, you know.

106

00:16:01.180 --> 00:16:18.699

PT7: I've had the good fortune, though, when I was at downstate, to be on the other end of the spectrum, and had the opportunity to use the altar. G [treadmill], you know. And so that was it's tough. It's the same problem is getting the kid in the equipment is the hard part.

107

00:16:18.710 --> 00:16:24.660

PT7: but you know, if the kid was big enough and heavy enough, it worked, you know you could at least get them moving a little bit

108

00:16:26.950 --> 00:16:42.290

NM: that's great. So i'm gonna pull up the next part. a survey that we're gonna look at is called the Promise scale. Are you familiar with the promise scale. You think so? Okay. So let me show you this one, because this is there's so many I've got promise scales. But this one is the physical activity.

109

00:16:42.310 --> 00:16:57.699

NM: the parent proxy, physical activity, scale that was developed for children. I think believe it was oncology kittles where they were trying to look children. They were more involved, right? And so and now it has been used in other populations. So

110

00:16:57.860 --> 00:17:01.650

NM: what i'm looking to see from Pt. Is first, and then I'm going to go to parents

111

00:17:01.700 --> 00:17:16.579

NM: just looking at the scale. How appropriate is it for the kids that i'm studying right is for kids that are non inventory at Gms. Of the 4 and 5. So i'm going to ask you about each question, and then i'm going to ask you how appropriate so 0 not related at all.

112

00:17:16.619 --> 00:17:28.000

NM: Right? And then on the scale, any number from 0 to 5 5 being highly appropriate for this population. This will be an appropriate question to ask a parent for a child with this

113

00:17:28.520 --> 00:17:32.310

NM: this disorder. Okay, so let me go ahead and give you here.

114

00:17:32.720 --> 00:17:34.820

NM: And so first question is.

115

00:17:36.900 --> 00:17:49.029

NM: how many days did your child exercise or place so hard that his or her body got tired? How would you rate that? Would it be 0 or not appropriate at all? 5 highly appropriate anywhere in between?

116

00:17:50.020 --> 00:17:56.650

PT7: Yeah, of the group, Just kind of scanning what's on the screen. But of of the group that one is.

117

00:17:57.430 --> 00:18:00.269

PT7: I think, appropriate. You know what I mean, because

118

00:18:02.020 --> 00:18:06.060

NM: give me a number. Oh, I would say 4. Okay.

119

00:18:06.120 --> 00:18:09.349

NM: And because it's appropriate, as you were saying.

120

00:18:09.770 --> 00:18:19.459

PT7: Yeah, because you know, you can. A a child that's physically impaired will still show being tired, you know, and a parent would be able to read that.

121

00:18:19.930 --> 00:18:20.690

PT7: you know.

122

00:18:21.900 --> 00:18:23.310

Yeah, right?

123

00:18:23.550 --> 00:18:33.509

NM: Alright? Second question. How many days is your child exercise really hard for 10 min or more. Is this appropriate? 0. Not at all. 5 highly inappropriate. How would you rate it?

124

00:18:38.370 --> 00:18:52.109

PT7: The devil is in the definitions, you know that's okay. You can tell me what things you what what change. And I would I would say 3 as a response for this one, you know. and

125

00:18:52.450 --> 00:18:56.419

PT7: you know, how do you define? Really hard? That's okay.

126

00:19:00.390 --> 00:19:02.140

NM: So it's more the definition.

127

00:19:02.320 --> 00:19:05.199

NM: Yeah, it's not it's not. It's not as clear. Okay.

128

00:19:05.310 --> 00:19:08.440

PT7: Yeah, you know what's really hard for me is.

129

00:19:08.900 --> 00:19:11.750

PT7: you know, easy for somebody else, you know.

130

00:19:12.170 --> 00:19:31.330

PT7: Right? Right? You know, I was. Yeah, I was out with my sister in law this weekend, and I'm, you know i'm a walker. You know what I mean, and you know she had a hard time keeping up, and I had to consciously say like, stop. So anyway, going on

131

00:19:32.330 --> 00:19:34.190

NM: all right, all right. Number 3.

132

00:19:34.360 --> 00:19:38.450

NM: How many did your child exercise so much that he or she breathed heart.

133

00:19:38.920 --> 00:19:45.130

NM: Would that be related? Highly appropriate to 5 or somewhere lower to 0? Not appropriate at all.

134

00:19:45.330 --> 00:19:49.220

PT7: I I think it's appropriate, I would say for

135

00:19:49.340 --> 00:19:55.449

PT7: it's hard to get this level of a kid to that point, but

136

00:19:55.540 --> 00:19:57.829

PT7: you could. It's potential, you could.

137

00:20:02.950 --> 00:20:04.340

NM: And number 4.

138

00:20:04.660 --> 00:20:13.679

NM: How many days was your child so physically active that he or she sweat it? Would you say 0 not related at all 5 highly appropriate. And why.

139

00:20:16.770 --> 00:20:17.900

PT7: Yeah, I would.

140

00:20:18.410 --> 00:20:21.819

PT7: I would say, maybe 2. You know it's

141

00:20:21.910 --> 00:20:23.200

PT7: it's I mean.

142

00:20:24.220 --> 00:20:31.540

PT7: yeah, the kids don't really. They don't get to a high enough level, you know, I don't think to to demonstrate enough sweating. And then

143

00:20:31.810 --> 00:20:35.959

PT7: or the kids just sweaty cause they're sweaty kids.

144

00:20:36.190 --> 00:20:39.450

PT7: Yeah, okay,

145

00:20:43.220 --> 00:20:49.619

NM: It may be due to some other physiological reason. Yeah, that's what I mean.

146

00:20:56.540 --> 00:21:04.389

NM: Number 5. How many days did your child exercise or play so hard that his or her muscles burned?

147

00:21:04.690 --> 00:21:19.920

PT7: Yeah, I. This one, I think, is a 0. I you're not gonna get to that level of exercise in these kids like, I said. Maybe for the exceptional, you know, adolescent. You know who's gonna burn out on an organometer or something. But

148

00:21:20.740 --> 00:21:21.350

yeah

149

00:21:23.590 --> 00:21:33.370

NM: and I asked the last person to interview this. They they something similar? That was said. I think I asked. So why why wouldn't you want to burn their muscles?

150

00:21:33.990 --> 00:21:35.429

PT7: I would want to.

151

00:21:35.650 --> 00:21:39.950

PT7: Okay, I I would definitely want to. But I don't

152

00:21:40.090 --> 00:21:49.110

PT7: in this collection of questions. That's the most appropriate for this population, because the majority of the population is not going to get there.

153

00:21:49.810 --> 00:21:50.669

NM: Got it.

154

00:21:52.250 --> 00:21:58.210

PT7: But that's important that you would want to help get them to that level. Absolutely. Yeah, but

155

00:21:58.300 --> 00:21:59.140

PT7: you know

156

00:21:59.230 --> 00:22:01.599

PT7: on a on a you know.

157

00:22:02.120 --> 00:22:05.229

PT7: 95% of kids are not going to get there

158

00:22:17.970 --> 00:22:24.710

NM: all right. Number 6. How many days are your child exercise or play so hard that he will keep cell type.

159

00:22:25.860 --> 00:22:29.459

PT7: Yeah, I think that's appropriate. 5,

160

00:22:29.520 --> 00:22:32.020

PT7: I think a parent would be able to

161

00:22:32.120 --> 00:22:45.039

PT7: even discern that in a child who is nonverbal. Right? So let's say you on a thing. And then, like all of a sudden, they're like, I can't hold themselves up. They're tired, you know.

162

00:22:45.900 --> 00:22:52.470

NM: That's good Number 7. How many days was your child physically active for 10 min or more?

163

00:22:52.520 --> 00:22:57.300

PT7: Yep, that's a 5 very appropriate. That's just an activity log. That's easy.

164

00:22:57.390 --> 00:22:58.380

NM: Okay?

165

00:23:02.710 --> 00:23:07.079

NM: And number 8. How many days is your child run for 10 min or more?

166

00:23:07.280 --> 00:23:11.699

PT7: Yeah. So that's a no right. What number would you give it.

167

00:23:11.720 --> 00:23:20.459

PT7: I would give it a 0, because they're not going to run, so the only caveat is is.

168

00:23:21.090 --> 00:23:22.550

PT7: would you

169

00:23:23.710 --> 00:23:30.040

PT7: maintain mobility for 10 min or more like. Could a kid, you know, drive his chair for 10 min.

170

00:23:30.780 --> 00:23:33.279

PT7: but that probably alters the

171

00:23:33.460 --> 00:23:39.459

PT7: questionnaire too much. That would make it invalid. But in general that's a no.

172

00:23:40.330 --> 00:23:48.530

NM: and that's my last question. I always ask everyone. pt was come on. Is there any last thoughts or or final time, as if you

173

00:23:48.650 --> 00:23:50.520

NM: any last calls at

174

00:23:51.230 --> 00:23:52.090

anything?

175

00:24:00.470 --> 00:24:08.799

NM: Sorry. All right. Final thoughts and comments before I let you go about physical activity in this population.

176

00:24:09.360 --> 00:24:21.909

PT7: Yeah, this is kind of like the last frontier, you know. I mean there's never a last frontier, you know what I mean, but this is where we, as a profession, really have to push. You know what I mean. This is what

177

00:24:22.070 --> 00:24:23.230

PT7: these kids

178

00:24:23.270 --> 00:24:29.089

PT7: need as well as the ambulatory kids as well. You know what I mean. the whole

179

00:24:29.110 --> 00:24:36.490

PT7: therapy world has to step aside. You know what I mean, and we have to figure it out and develop

180

00:24:38.070 --> 00:24:47.419

PT7: what we can. You know community based, whatever it is to help, you know. But this is a really really vital and important topic, and it's great that you're studying it.

181

00:24:53.790 --> 00:25:01.979

PT7: Now, we just have to develop something and invent something. Really, I know you're so right. It's like it. And this is on. This is the

182

00:25:02.260 --> 00:25:15.910

NM: the beginning of just understanding how we measure and define it, you know. And so we got a long road ahead of us, but I I do appreciate everything you shared so far. Hold on, let me just go, and we're gonna wrap this recording up. Thank you.
