# Supplementary material for: Validity of PROMIS® Pediatric Physical Activity Parent Proxy Short Form Scale as a Physical Activity Measure for Children with Cerebral Palsy Who Are Non-Ambulatory
Source: Behav Sci (Basel). 2025 Jul 31;15(8):1042. doi: 10.3390/bs15081042 (PMC12382615; doi:10.3390/bs15081042)
Supplement: Supplementary file 1 [file behavsci-15-01042-s001.zip › Transcripts copy/PT transcripts - deidentified/PT21.docx]

WEBVTT

1

00:00:01.150 --> 00:00:24.440

NM: All right. Good afternoon. Thank you so much for joining us today. We're going to talk a bit about physical activity and children with Cp. Functioning at Levels, Gmf: Cs levels 4 and 5. So I do have a couple of questions for the first half and some prompt. So if I sound scripted is because I am the second half. I will show a survey developed by National Institute of Help, the Promise survey

2

00:00:24.450 --> 00:00:33.080

NM: that is, gear for parents to answers a parent proxy report on physical activity. And I ask you specific questions as it relates to that.

3

00:00:33.120 --> 00:00:34.559

Now we'll start

4

00:00:34.620 --> 00:00:40.840

NM: first question: how do you define physical activity for children with Cp. Who are not full time Walkers.

5

00:00:41.340 --> 00:00:56.999

PT21: Yeah. So I would say, for most of the students on my case load, I would consider any active movement that seems intentional would be considered physical activity for me. Some of my students, who

6

00:00:57.010 --> 00:01:12.880

PT21: are a little bit more active and able to kind of control their movements. I would look at more of their like respiratory rate if they're getting out of breath while we're moving. If they seem like They're fatiguing after a certain movement. But I would say, overall any

7

00:01:12.890 --> 00:01:17.030

PT21: active movement on on their end would be physical activity.

8

00:01:19.010 --> 00:01:32.369

NM: Thank you. Great. So the Department of Health defines physical activity as any activity that encompasses energy expended and activation of skeletal muscle. Does this definition? Does this definition change your mind about how you define

9

00:01:32.530 --> 00:01:33.729

NM: physical activity.

10

00:01:34.640 --> 00:01:37.880

PT21: No, I think it's pretty much in line with

11

00:01:38.590 --> 00:01:42.230

PT21: with kind of how I I view it, at least in terms of like

12

00:01:42.760 --> 00:01:46.390

PT21: when i'm thinking about like specific pt sessions. Yeah.

13

00:01:46.800 --> 00:01:47.470

okay.

14

00:01:47.560 --> 00:01:52.700

NM: And how do you think physical activity differs from other types of fitness activities?

15

00:01:54.760 --> 00:02:05.969

PT21: I would say physical activity in my mind is probably less strenuous than fitness activity. I would consider physical activity really like

16

00:02:06.010 --> 00:02:17.390

PT21: any movement that the student can do in their body, whereas, like fitness activities more geared towards like a specific exercise, or having them do like repetitions of an exercise.

17

00:02:18.810 --> 00:02:24.040

PT21: maybe in like a more structured like exercise program type of format.

18

00:02:27.130 --> 00:02:28.390

NM: Great. Thank you.

19

00:02:28.840 --> 00:02:35.020

NM: And when do you witness your students participate the most in physical activity during this today.

20

00:02:36.420 --> 00:02:47.219

PT21: during the school day, like in like a Pt. Session, or just like throughout the in general throughout the day. Like when do you feel like you witness them, participate the most in physical activity.

21

00:02:47.790 --> 00:03:05.850

PT21: I would say definitely, probably during physical therapy, like if they have a Pt. Session. We also have a sensorium that some of our classes are able to use throughout the day. So they have a day where they're in sensorium. I would say that's probably up there for

22

00:03:05.880 --> 00:03:08.509

PT21: physical activity, but those would be the top 2.

23

00:03:12.140 --> 00:03:16.700

NM: When do you? All right? And i'm sorry I about to say the same question number 2:

24

00:03:17.400 --> 00:03:28.150

NM: How do you measure physical activity, frequency, intensity, time and type quoting a fitt principle there in children with Cp. Who are not full time walkers.

25

00:03:28.490 --> 00:03:31.329

NM: So i'll say it Again.

26

00:03:31.530 --> 00:03:34.540

NM: How do you measure, physical activity, frequency.

27

00:03:34.640 --> 00:03:40.450

NM: intensity, time and type in children with Cp who are not full-time walkers.

28

00:03:41.310 --> 00:03:51.029

PT21: Yeah. So I would consider like most of the time that our students are in a therapy session. We have a 45 min therapy session, but

29

00:03:51.140 --> 00:03:59.219

PT21: it's mostly like 30 min of activity, I would say, for most of our students anytime they're out of their chair. Whether or not they're

30

00:03:59.270 --> 00:04:00.650

PT21: doing something that

31

00:04:00.920 --> 00:04:18.630

PT21: maybe perceive this physical activity or not. It is pretty physical for them just the demands that it's placed on them. To be able to maintain an upright head, even in a position that you would think is, you know, relatively inactive it. It takes a lot for some of our students, so

32

00:04:18.640 --> 00:04:24.129

I would consider them to be physically active throughout the entire session that we have them

33

00:04:24.400 --> 00:04:28.759

PT21: the what was the other one? It was frequency, intensity, time.

34

00:04:30.070 --> 00:04:31.650

PT21: yeah, and type

35

00:04:31.960 --> 00:04:40.160

PT21: and type. So I would say, intensity would depend on the activity. But I would say, most of the times we reach

36

00:04:40.670 --> 00:04:41.950

PT21: a moderate

37

00:04:42.030 --> 00:04:44.170

PT21: intensity throughout a session.

38

00:04:45.390 --> 00:05:03.549

PT21: trying to think of like a full session. It it doesn't. We can't sustain that through an entire session, just because of like the levels of endurance, most of our students. But I would say, like at the peak in a session we are able to reach moderate, even if it's only for, like a very brief period of time, like a minute or 2,

39

00:05:03.850 --> 00:05:09.980

PT21: and then type of physical activity, I would say that would probably depend

40

00:05:10.050 --> 00:05:14.290

PT21: on the session. Do they mean like specific activity for that or

41

00:05:14.350 --> 00:05:16.269

NM: It's just. However you may look at it.

42

00:05:16.700 --> 00:05:28.400

PT21: Yeah, so I would say, type would be. We kind of depend on the physical activity. So if it was like 8 training or working on more like head trunk control.

43

00:05:28.620 --> 00:05:32.480

PT21: I would like expect for us to have like higher

44

00:05:32.600 --> 00:05:39.690

PT21: energy demands when we're upright in any pieces of equipment versus being like down on the map, but that's not

45

00:05:39.800 --> 00:05:46.010

PT21: always feasible for some of our students who have like physical limitations for that.

46

00:05:46.040 --> 00:05:50.259

PT21: So I don't know if that was a good answer. Oh, that's great. Thank you.

47

00:05:50.310 --> 00:05:55.039

NM: Do they need assistance to complete the activities you mentioned?

48

00:05:55.430 --> 00:05:56.350

PT21: Yes.

49

00:05:56.440 --> 00:06:03.280

NM: yeah. And is it? During during which activities and do they need it for a or whole, the whole task.

50

00:06:03.640 --> 00:06:18.290

PT21: Yeah. So I would say, any activity. They definitely that is, weight, bearing activity and uptight activity. They would need assistance, at least from a gait trainer, and at most like physical hands on, like

51

00:06:18.550 --> 00:06:21.270

PT21: Max, assist from a therapist

52

00:06:21.300 --> 00:06:31.960

PT21: activities that are more like mat based activities like sitting balance or working on transfers depends on the student. But we.

53

00:06:32.120 --> 00:06:36.649

PT21: i'm trying to think, like from personally, from my case load. I have

54

00:06:37.160 --> 00:06:46.019

PT21: probably 80% of my students. I would need to have like physical hands on their trunk in order to help keep them up right, and then

55

00:06:46.670 --> 00:06:50.950

PT21: the other 20%. It's like very close contact guard.

56

00:06:51.530 --> 00:06:52.150

Okay.

57

00:06:56.000 --> 00:07:02.870

NM: that's great. Thank you. And do you think they should participate in more or less of each of these activities? And why

58

00:07:03.020 --> 00:07:09.180

PT21: definitely more, I think that for a lot of our students I see

59

00:07:10.040 --> 00:07:17.199

PT21: when we're like in session and session like school session is rolling, and you know, we're getting like consistent

60

00:07:18.130 --> 00:07:34.790

PT21: therapy throughout the week, I definitely notice, increases in their endurance and their ability to tolerate physical activity. Once we hit like summer break or winter break even that, like 2 weeks that we have off for winter break. Our kids come back, and I find that a lot of them are

61

00:07:34.800 --> 00:07:39.289

PT21: kind of back to where we started in the beginning of the year.

62

00:07:39.360 --> 00:07:49.749

PT21: just a little bit more deep condition. It takes us a little bit longer to kind of get into the rhythm of an activity, whereas right before those breaks normally, we can like, jump in and go.

63

00:07:49.840 --> 00:08:06.550

PT21: So I think more would definitely kind of help with like keeping that consistent level, and then being able to build on it. Then, having, like these little minor regressions, they usually are able to like build their endurance back up within like a week or 2. But it does take

64

00:08:06.820 --> 00:08:10.609

PT21: like that initial period to kind of get them back to where we left off.

65

00:08:13.050 --> 00:08:14.040

NM: Yeah.

66

00:08:14.860 --> 00:08:22.030

NM: thank you. All right. So third question, do you address promoting physical activity during your Pt sessions?

67

00:08:23.620 --> 00:08:28.030

PT21: Yes, I think that we have tried to do

68

00:08:28.420 --> 00:08:32.960

PT21: a good job of making sure that families have equipment at home to promote.

69

00:08:33.020 --> 00:08:41.959

PT21: you know, getting out as much as possible throughout like evenings and weekends and things like that, and students that i'm.

70

00:08:41.970 --> 00:08:59.199

PT21: you know, able to kind of have that discussion with. We like stress the importance of getting into the stander and getting into positions that will help to kind of keep them active throughout the day. Even in classrooms we did have classroom consults

71

00:08:59.210 --> 00:09:16.009

PT21: every Friday, for, like 45 min in the morning, where Pt. Would go into each classroom and discuss what positions we can get students to add in what throughout the day we can use to kind of break up their day to kind of promote different positions, different.

72

00:09:16.480 --> 00:09:19.279

PT21: and also still be able to access, like

73

00:09:19.460 --> 00:09:32.249

PT21: their environment in their classroom. So it's not like you're totally taking them out and making what should be an educational setting like a pt setting. But just to try to help keep active throughout the day.

74

00:09:32.730 --> 00:09:35.949

NM: No, that's great, but it but it for

75

00:09:36.170 --> 00:09:42.619

NM: but specifically in your in your Pt. Session, how do you address physical activity

76

00:09:44.320 --> 00:09:53.880

NM: like with the with your child like your child you're treating. How do you address physical activity? And how do you address promoting PA in your actual setting.

77

00:09:54.450 --> 00:09:57.440

PT21: So your session I'm: sorry in your actual session.

78

00:09:57.610 --> 00:10:10.530

PT21: Yeah. So I would say, every student knows that when they come in Pt. They're going to get out of their chair, and there's going to be some sort of physical activity we usually. I like to try to give choices

79

00:10:10.540 --> 00:10:22.850

PT21: for an activity. So kind of letting the student know that, like Yes, we are going to get out. We're going to do something active. You have the choice between this and this, and then

80

00:10:23.000 --> 00:10:26.489

PT21: you know, if it doesn't go well like giving them the option to

81

00:10:26.640 --> 00:10:31.289

PT21: choose something different. I think that sometimes like with our kids.

82

00:10:31.830 --> 00:10:49.749

PT21: letting them know that we can be flexible in a session. And like, if we decide we're gonna do the gait trainer in the beginning of a session, and they're just not feeling it, or it's not going as well as what we both know they can do. Then, giving them the option to to try something different. So I think that

83

00:10:50.150 --> 00:10:54.509

PT21: they trust that whatever physical activity they're going to do is going to be

84

00:10:54.530 --> 00:10:58.469

PT21: safe. It's going to be something that they can enjoy. And

85

00:10:59.410 --> 00:11:01.510

PT21: yeah, it's going to be active. But

86

00:11:01.630 --> 00:11:04.120

PT21: they at least have the choice of what it's going to be.

87

00:11:05.700 --> 00:11:23.729

NM: Yeah, and what components of physical activity. Do you address in your session, for example, cardiovascular endurance, muscle, activation, energy, expenditure, and mobility? So many things you could work on. But what are the components. Do you feel like you address the most when you're when you're working on physical activity?

88

00:11:24.040 --> 00:11:33.950

PT21: Yeah, I would say with my specific case load right now, I've been trying to do a lot of just strengthening and endurance work.

89

00:11:34.010 --> 00:11:35.250

PT21: We do

90

00:11:35.770 --> 00:11:43.379

PT21: a lot of the mobility work that we that I do is like power, mobility, work. So

91

00:11:44.440 --> 00:11:46.220

PT21: not necessarily like

92

00:11:46.410 --> 00:11:57.069

PT21: functional ambulation. But I would say that when I think of mobility, I think more power, mobility, wheelchair, training than like gait training

93

00:11:57.760 --> 00:12:16.160

PT21: and overall. Yeah, just I would say, all of my students, regardless of like their physical ability. It's. We focus a lot on just trying to strengthen whether it's head control or actually like weight, bearing in order to improve things like transfers. Even the transition from like

94

00:12:16.170 --> 00:12:20.589

PT21: lying into sitting, being able to control your head. Things like that.

95

00:12:20.870 --> 00:12:23.469

PT21: So I I guess number one would be strength.

96

00:12:23.720 --> 00:12:28.890

NM: Okay, thank you. And are there ever reasons why you may not focus on this

97

00:12:29.430 --> 00:12:31.289

NM: or any component. Ca.

98

00:12:31.580 --> 00:12:44.430

PT21: Yeah. So I mean, every time a student comes in we kind of do a quick assessment make sure that they're feeling up for the activity. I have had students recently who

99

00:12:44.510 --> 00:13:00.739

PT21: they're on like a G-tube feed, and if their feed is continuous it makes it difficult for them to tolerate certain positions like lying flat or being upright so in my mind. If I was thinking about working on getting up into like standing that day, and

100

00:13:00.750 --> 00:13:07.829

PT21: they had a change in that status. Then we kind of would switch gears to something a little less strenuous.

101

00:13:09.070 --> 00:13:21.249

PT21: And then, also, like there, the availability of orthotics. So if they're having like an issue with a mafo or they're getting redness, we would kind of change gears in order to be able to assess that.

102

00:13:21.690 --> 00:13:22.640

NM: Yeah.

103

00:13:22.980 --> 00:13:25.479

all good points. Thank you.

104

00:13:26.020 --> 00:13:34.239

NM: All right. Do you address promoting you already? Kind of answered this actually. But do you address promoting physical activity that occurs outside of your Pt sessions.

105

00:13:34.410 --> 00:13:37.950

PT21: Yes, yeah. So we definitely try

106

00:13:37.990 --> 00:13:44.580

PT21: to partner with families and make sure that they have what they need to be. It's like so challenging for

107

00:13:44.970 --> 00:14:01.040

PT21: our families to be able to do like physical activity in terms of like gait trainers and standers and things like that at home. But we try to make sure that if they, even if they have like a half hour, and they want to put the time to do it that they're set up at home to be able to do that.

108

00:14:01.050 --> 00:14:08.099

PT21: So we try to do like instructions. Videos. Get them the equipment that they need

109

00:14:08.830 --> 00:14:17.850

PT21: they send equipment in so we can resize it, making sure that everything is like fitting appropriately, and then in the classrooms we've been trying to.

110

00:14:18.200 --> 00:14:21.440

PT21: It's not perfect. Our school's gotten better at

111

00:14:21.600 --> 00:14:26.299

PT21: kind of understanding and recognizing the importance of getting students out.

112

00:14:26.440 --> 00:14:40.749

PT21: we do have a challenge with students who are like a little bit bigger, getting them out, and just being able to space-wise find the space in a classroom to do that. But I think that PT has really made an emphasis this year on

113

00:14:40.810 --> 00:14:47.989

PT21: at least making people aware that their options for their students to be able to use throughout the day.

114

00:14:48.130 --> 00:14:53.079

PT21: And we're down the hall, so we're always available for assistance, as people need it.

115

00:14:54.310 --> 00:15:00.580

NM: Gotcha. So have you ever recommended any community programs or events to your students to help

116

00:15:00.660 --> 00:15:02.330

NM: increase physical activity.

117

00:15:03.640 --> 00:15:06.220

PT21: No, I would say.

118

00:15:06.510 --> 00:15:11.669

PT21: one of the challenges that Hms. Has with community events is that not

119

00:15:11.800 --> 00:15:16.229

PT21: many of our students live in this area. So since we

120

00:15:16.390 --> 00:15:19.029

PT21: or an approved private school we pull from

121

00:15:19.240 --> 00:15:34.350

PT21: I I don't even know the number. It's like 40 different school districts, or something like that so a lot of our students have, like an over an hour bus ride to get here. So events that are like within our immediate community are not always

122

00:15:34.770 --> 00:15:38.230

PT21: feasible. There's not that, many of them. And then.

123

00:15:38.320 --> 00:15:43.020

PT21: when there are, it's just not always that easy to to get to

124

00:15:43.340 --> 00:15:44.230

NM: got it.

125

00:15:44.520 --> 00:15:45.600

Okay.

126

00:15:45.720 --> 00:15:55.189

NM: And what type of equipment you give some examples earlier. But what type of equipment have you recommended to help improve home and or community engagement

127

00:15:55.250 --> 00:15:57.899

NM: and physical activity outside of the clinical setting.

128

00:15:58.820 --> 00:16:03.710

PT21: Yeah, so definitely gait trainers, we you one like specific brands.

129

00:16:03.780 --> 00:16:09.389

NM: No, it doesn't it. That's fine. You can give me a brand if you want to, or you don't have to, whatever you have you you recommend.

130

00:16:09.540 --> 00:16:15.300

PT21: Yeah, we use Rifton products a lot like the tram and the E Pacer.

131

00:16:15.540 --> 00:16:26.750

PT21: the kid walk we've recommended for families to use at home that we, the nice thing about the kidwalk in the Epacer. So that we have started to kind of

132

00:16:26.790 --> 00:16:45.699

PT21: use. More of is that the student can face out without having a gait trainer like the actual equipment in front of them, so you could pull them right up to a counter or a table, and be able to use their hands or help in the kitchen, or whatever it is like hit a switch to turn on the blender. We've had a lot of families be very creative about

133

00:16:45.710 --> 00:16:49.820

PT21: how they incorporate equipment into like a daily routine.

134

00:16:51.510 --> 00:16:58.219

PT21: The standards that we recommend are typically like easystand type of sit to stand standards

135

00:16:58.380 --> 00:16:59.840

PT21: that students

136

00:17:00.060 --> 00:17:07.139

PT21: to our students tend to do pretty well on them. So we always like evaluate them before we recommend them for home. But

137

00:17:07.800 --> 00:17:09.379

PT21: we do

138

00:17:09.720 --> 00:17:12.879

PT21: like bikes and stuff recommend bikes, but

139

00:17:13.000 --> 00:17:26.379

PT21: they're a little bit more challenging space wise just like it's a very big commitment for a family to have bike. But we have worked with organizations to get funding for bikes because they're not funded by insurance.

140

00:17:31.510 --> 00:17:32.530

NM: That's fine.

141

00:17:32.650 --> 00:17:36.709

NM: And okay. So now this time, look at the survey. So i'll pull it up so you can

142

00:17:36.830 --> 00:17:39.219

NM: for a second.

143

00:17:39.260 --> 00:17:40.010

PT21: Okay?

144

00:17:49.570 --> 00:17:52.419

NM: And again, this is called the promise

145

00:17:53.120 --> 00:18:05.319

NM: Parent Proxy. Physical activity survey. So they have 8 questions as a short form, and a parent would ask, or carrier would ask in the past 7 days they would answer, what? How many days their child

146

00:18:05.400 --> 00:18:13.330

NM: exhibited some level of intensity according to the scale. And so i'm going to ask you in a moment

147

00:18:13.420 --> 00:18:16.450

NM: to rate each question on how appropriate

148

00:18:16.620 --> 00:18:30.020

NM: one is, face, how valid is it for this population? Children, with Cp, that are non-emulatory at functioning at level Gms. As well as 4 and 5, and then I'm gonna ask Why, okay, look at that for a second.

149

00:18:31.650 --> 00:18:34.519

NM: and then we'll go through the the ranking.

150

00:18:34.850 --> 00:18:41.819

NM: all right. So i'm gonna ask you to rank the the each question from 0 not related at all up to 5

151

00:18:41.840 --> 00:18:45.709

NM: highly appropriate. And then why okay? So the first question

152

00:18:46.650 --> 00:18:56.220

NM: as how many days your child exercise or play so hard that his or her body got tired. How appropriate is this question, as it relates to physical activity, intensity

153

00:18:56.270 --> 00:18:59.739

NM: for children functioning, and GM. Fcs. Levels 4 and 5

154

00:19:00.530 --> 00:19:07.000

NM: 0 is not related at all. 5 is highly appropriate. How would you rate rank it from 0 between 5?

155

00:19:07.330 --> 00:19:12.369

PT21: Yeah, I would probably give it like a one. I think that it's.

156

00:19:12.740 --> 00:19:15.139

PT21: I think it's hard for our

157

00:19:16.070 --> 00:19:17.890

PT21: students to

158

00:19:18.480 --> 00:19:29.999

PT21: give a good assessment of whether or not their body is tired from physical activity. I think they have a lot of challenges with their physical state. Just baseline, so to say that they were

159

00:19:30.060 --> 00:19:34.489

PT21: tired specifically from an exercise or play

160

00:19:34.600 --> 00:19:37.159

PT21: would be hard. I think that they.

161

00:19:37.570 --> 00:19:41.189

PT21: I think it'd be easier for them to assess whether or not they were fatigued.

162

00:19:41.460 --> 00:19:50.030

PT21: Fine that activity, then it would be to say that their like body muscles were tired from the activity.

163

00:19:50.610 --> 00:19:51.350

Right?

164

00:19:51.880 --> 00:19:53.450

NM: All right. Next question

165

00:19:53.890 --> 00:19:55.150

NM: number 2.

166

00:19:55.840 --> 00:20:13.009

NM: How many days is your child exercise really hard for 10 min or more? How appropriate. Would you believe this is for a parent to answer a parent of a child with Gms at functioning at Gmail Level 4 and 5 0, not related at all up to 5 highly appropriate.

167

00:20:14.570 --> 00:20:17.520

PT21: I would say, probably a 3.

168

00:20:17.560 --> 00:20:18.820

NM: Okay in one.

169

00:20:19.470 --> 00:20:25.759

PT21: Yeah, I feel like it's the the really hard part of that question that's

170

00:20:26.670 --> 00:20:31.010

PT21: challenging for me, just because I I think that it's hard

171

00:20:31.330 --> 00:20:37.700

PT21: even for Pt. To assess sometimes what is really hard for some of our students.

172

00:20:37.870 --> 00:20:49.750

PT21: I think on certain days it's really hard for them to keep their heads up. And then on other days it could be really hard for them to do like another type of activity that might not seem like

173

00:20:49.910 --> 00:20:51.320

PT21: exercise.

174

00:20:51.620 --> 00:20:54.310

PT21: I don't know if that makes sense.

175

00:20:55.370 --> 00:20:57.180

NM: No, that's great. Thank you.

176

00:20:57.300 --> 00:20:58.580

Hello, Your standard.

177

00:20:58.900 --> 00:21:00.199

NM: all right. Number 3.

178

00:21:00.580 --> 00:21:05.979

NM: How many days did your child child exercise so much that he or she breathe hard?

179

00:21:06.070 --> 00:21:10.150

NM: 0 not related at all. 5 highly appropriate for this population.

180

00:21:12.380 --> 00:21:14.730

PT21: Yeah, I would. I would say 4.

181

00:21:15.320 --> 00:21:16.299

NM: Okay. And why

182

00:21:17.250 --> 00:21:23.389

PT21: I I do think that our kids, as they exercise like in Pt: I've seen them

183

00:21:23.730 --> 00:21:27.629

PT21: like. I think that's a good measure of whether or not they're

184

00:21:28.060 --> 00:21:30.590

PT21: actually like exercising, or like

185

00:21:30.690 --> 00:21:37.020

PT21: reaching a level where they would be working hard enough that it would challenge them.

186

00:21:38.490 --> 00:21:41.930

PT21: I think that's probably an easier measure, because it's

187

00:21:42.630 --> 00:21:50.090

PT21: physical. It's something that the parent can see rather than assessing whether or not they feel like their body is hard, because parent can't really

188

00:21:50.470 --> 00:21:54.630

PT21: always get a good determination of that from their from their child.

189

00:21:55.720 --> 00:21:58.409

NM: Okay, thank you. Number 4.

190

00:21:58.600 --> 00:22:02.619

NM: How many days was your child so physically active that he or she sweated.

191

00:22:05.320 --> 00:22:07.189

PT21: Yeah, I would probably say

192

00:22:08.030 --> 00:22:08.670

PT21: like

193

00:22:08.980 --> 00:22:12.299

PT21: 0, I think.

194

00:22:12.550 --> 00:22:21.269

PT21: Yeah, I don't know our kids, I in all my time working with the students here. I've never gotten like a physical response. So

195

00:22:21.350 --> 00:22:24.150

PT21: them sweating from activity

196

00:22:25.190 --> 00:22:26.930

PT21: I don't know.

197

00:22:27.220 --> 00:22:28.259

PT21: I think, like

198

00:22:28.360 --> 00:22:35.300

PT21: baseline. Some of our kids get sweaty just because they're wearing like orthodox, and they have like.

199

00:22:35.550 --> 00:22:39.589

PT21: Usually they're in some sort of equipment. I don't know if it's a good assessment.

200

00:22:39.960 --> 00:22:48.700

PT21: just because I feel like there's too many external factors, like pieces of equipment that make them sweaty rather than like actually

201

00:22:48.750 --> 00:22:50.360

PT21: the work that they've done.

202

00:22:51.520 --> 00:22:53.780

NM: All right. Thank you. Number 5.

203

00:22:54.080 --> 00:23:01.450

NM: How many days your child exercise a place so hard that his or her muscles burn? 0 not related at all up to 5 highly.

204

00:23:02.410 --> 00:23:09.830

PT21: Yeah, I would say, probably 0. I think it's similar to like the body getting tired. Question where it I just

205

00:23:10.880 --> 00:23:14.970

PT21: I think that's too hard to assess, for most of our students.

206

00:23:16.760 --> 00:23:18.399

NM: Right Number 6.

207

00:23:19.010 --> 00:23:23.259

NM: How many days your child exercise a place so hard that he or she felt tired.

208

00:23:24.030 --> 00:23:27.350

NM: 0 now related. Well, up to 5.

209

00:23:28.700 --> 00:23:33.669

PT21: Yeah, I would say 4, I think 4 or 5. Yeah. I think that that's

210

00:23:34.330 --> 00:23:38.910

PT21: an appropriate. I think that that's like easier to assess for a parent.

211

00:23:39.080 --> 00:23:40.309

PT21: and it's.

212

00:23:40.340 --> 00:23:44.350

PT21: I think, accurate. I do think that some of our kids like tap out

213

00:23:44.860 --> 00:23:49.610

PT21: with an exercise due to fatigue, more than like muscle, burn

214

00:23:49.630 --> 00:23:52.020

PT21: where, like their body, feeling tired.

215

00:23:53.600 --> 00:23:57.660

NM: so final? Answer You said 405, which one would you like to say.

216

00:23:57.930 --> 00:23:59.659

let's go for

217

00:23:59.990 --> 00:24:00.850

Okay.

218

00:24:02.580 --> 00:24:03.360

NM: All right.

219

00:24:03.680 --> 00:24:05.280

NM: Number 7.

220

00:24:06.320 --> 00:24:13.889

NM: How many days was your child physically active for 10 min or more? 0 not related at all? 5 highly appropriate. Why.

221

00:24:14.630 --> 00:24:17.919

PT21: I think 5. I think that that's

222

00:24:18.360 --> 00:24:20.870

PT21: something that a pair would be what, said

223

00:24:22.020 --> 00:24:33.710

PT21: Assess, and it's not really measuring like their level of activity, just looking at whether or not they were physical, physically active. So I think that that's an an appropriate question.

224

00:24:36.810 --> 00:24:37.660

NM: Great

225

00:24:37.820 --> 00:24:45.280

NM: and number 8. How many days. Did your child run for 10 min or more? How will you relate that? How valid

226

00:24:45.370 --> 00:24:52.529

NM: would you? So this is a programs population 0 not related all 5 highly appropriate somewhere between. And why?

227

00:24:52.720 --> 00:24:56.130

PT21: Yeah, I would say, 0, None of our none of

228

00:24:56.270 --> 00:24:58.550

PT21: the students that we work with here are

229

00:24:58.890 --> 00:25:00.779

able to run.

230

00:25:04.280 --> 00:25:11.409

NM: and as we close, I like to ask about some final comments or thoughts as it relates to physical activity in this population.

231

00:25:11.540 --> 00:25:12.300

PT21: Okay.

232

00:25:13.520 --> 00:25:14.770

NM: anything like to share.

233

00:25:15.230 --> 00:25:26.409

NM: Oh, this is if you want to say any final comments or thoughts, or your thing is about just physical activity in this population.

234

00:25:26.670 --> 00:25:30.970

PT21: Yeah, I I think it's really exciting that you were trying to look

235

00:25:31.110 --> 00:25:35.690

PT21: closer at this. I do think it's challenging for

236

00:25:36.230 --> 00:25:44.270

PT21: us to be able to really get a good measure and a good assessment of physical activity for our students, I think

237

00:25:44.420 --> 00:26:01.109

PT21: part of it is that each one of them is so different with their physical ability, and then their response to physical activity, just because of all the underlying conditions that they have, that it does make it challenging to have, like a single assessment that

238

00:26:01.120 --> 00:26:05.109

PT21: can capture that. But I think that looking at it and

239

00:26:05.250 --> 00:26:07.140

PT21: looking at.

240

00:26:07.480 --> 00:26:10.160

PT21: basically especially for

241

00:26:10.270 --> 00:26:27.719

PT21: students who are level 4 and 5, I think, just acknowledging that they can be physically active, and that that's something that we want to promote and really kind of encourage. I think that's definitely we're moving in the right direction, so I appreciate all the work that you're putting into it.

242

00:26:28.520 --> 00:26:35.340

NM: Oh, thank you so much, and I appreciate that I agree totally with that, and we don't want to interview. I'm going to stop through

243

00:26:35.490 --> 00:26:38.669

NM: click our recording. Thank you for your time. Okay.
