# Supplementary material for: Validity of PROMIS® Pediatric Physical Activity Parent Proxy Short Form Scale as a Physical Activity Measure for Children with Cerebral Palsy Who Are Non-Ambulatory
Source: Behav Sci (Basel). 2025 Jul 31;15(8):1042. doi: 10.3390/bs15081042 (PMC12382615; doi:10.3390/bs15081042)
Supplement: Supplementary file 1 [file behavsci-15-01042-s001.zip › Transcripts copy/PT transcripts - deidentified/PT10.docx]

WEBVTT

1

00:00:02.840 --> 00:00:18.020

NM: good afternoon, PT10. Thank you for joining us today. We're going to talk about physical activity for children with Cp. Who are not full time, Walkers. I have a few questions for you, and in a couple of prompts for each question, and then the second half we're going to look at a a survey.

2

00:00:18.150 --> 00:00:26.689

NM: so for the first question, how do you define physical activity for children with through a policy who are not full time walkers.

3

00:00:28.660 --> 00:00:33.620

PT10: well, functionally, if I say, then

4

00:00:34.000 --> 00:00:38.850

PT10: that's the most challenging thing. Like if locomotive piece is missing.

5

00:00:39.020 --> 00:00:50.329

PT10: that is something which we are here for right physical therapy. So we try to work with whatever modalities or new techniques which we can help them

6

00:00:50.500 --> 00:00:52.330

PT10: to get online

7

00:00:52.690 --> 00:00:54.650

PT10: that the to start the locomotive move.

8

00:00:57.340 --> 00:01:08.499

NM: and so the first prompt is, the Department of Health defines physical activity as any activity that encompasses energy expended, and activation of skeletal muscle.

9

00:01:08.580 --> 00:01:13.580

NM: Does this definition change your mind about how you define physical activity.

10

00:01:15.680 --> 00:01:19.689

PT10: [In response to NIH definition] I feel like it should be more than what is being

11

00:01:19.890 --> 00:01:20.969

PT10: said in it.

12

00:01:21.540 --> 00:01:25.779

PT10: because these kids are totally dependent.

13

00:01:26.380 --> 00:01:27.140

PT10: So

14

00:01:27.810 --> 00:01:28.940

PT10: maybe

15

00:01:29.310 --> 00:01:32.779

PT10: maybe the functional aspect should be added to that definition.

16

00:01:41.490 --> 00:01:44.970

NM: And how do you think physical activity differs

17

00:01:45.190 --> 00:01:48.279

NM: from other types of fitness activities?

18

00:01:51.870 --> 00:01:53.089

PT10: I I

19

00:01:53.630 --> 00:01:59.310

PT10: I i'm not sure how to do that like whats... What's the context of the question

20

00:01:59.650 --> 00:02:17.250

NM: so like physical activity? If we go kind of with the definition from the Department of Health is any any any activity that encompasses energy, expenditure, or activation of skeletal muscles. And so fitness typically maybe something a little bit more exercise driven.

21

00:02:17.320 --> 00:02:20.250

NM: you know. Not just a routine.

22

00:02:20.700 --> 00:02:24.180

NM: you know, and that routine activity, right? And so

23

00:02:24.330 --> 00:02:26.130

NM: I think, with these children

24

00:02:26.240 --> 00:02:31.789

NM: like you said they're more dependent. So what can we count as physical activity for them?

25

00:02:32.170 --> 00:02:33.480

NM: If it's not gait?

26

00:02:35.320 --> 00:02:40.919

PT10: If it's not gait, then what else would be considered as physical activity? For

27

00:02:41.500 --> 00:02:42.180

oh.

28

00:02:43.930 --> 00:02:46.210

PT10: like this level of children that cannot walk

29

00:02:46.650 --> 00:02:49.690

PT10: So then children that cannot walk right.

30

00:02:56.300 --> 00:03:03.850

NM: and the and then you know it. Just thinking about that question I asked you. But how do you think physical activity for these kids? If you come up with another

31

00:03:04.370 --> 00:03:05.140

NM: kind of

32

00:03:05.270 --> 00:03:11.299

NM: way of looking at it? When you think about the Department of Health. How does that compare with fitness? Right?

33

00:03:11.370 --> 00:03:13.899

NM: So physical activity could just be?

34

00:03:14.060 --> 00:03:15.300

NM: let's say.

35

00:03:15.590 --> 00:03:25.240

NM: you know, walking around walking in the neighborhood park like just walking to the park, you know. That could be useful activity. But fitness may be more of.

36

00:03:25.310 --> 00:03:32.280

NM: you know, speed walking right because it has more of a goal to increase my heart rate.

37

00:03:32.620 --> 00:03:51.759

NM: It's not a leisure walk. So that's so. That's the question: how do you think physical activity is different, and I gave you example from other types of fitness activities. So how do we look at that in these case? So how would physical activity differ from fitness for these children. So I would consider the cardiovascular

38

00:03:51.770 --> 00:03:56.590

PT10: or cardi pulmonary fitness, fitness, as part of you know, to check

39

00:03:56.910 --> 00:04:08.710

PT10: for parameters for these kids like how how their posture is, if they are able to do the full diaphragmatic breathing or not, that could be considered one of the parameters

40

00:04:08.910 --> 00:04:10.999

NM: for physical activity, for fitness.

41

00:04:12.020 --> 00:04:16.610

PT10: I would consider that for both. Actually, Yes, yes.

42

00:04:17.990 --> 00:04:22.970

NM: so you would think these are kind of the same for these kids. Fitness and physical activity.

43

00:04:23.030 --> 00:04:28.170

PT10: Would you say it's like the same for these kids,

PT10: Yes because I feel like there's

44

00:04:28.220 --> 00:04:32.430

PT10: so limited factors where you can work with them.

45

00:04:32.970 --> 00:04:42.400

PT10: since the you know, like the generic field of locomotion, is missing. So you figure out what other systems can you focus on?

46

00:04:42.900 --> 00:04:48.379

PT10: To You know to engage them or to improve their physical

47

00:04:49.430 --> 00:04:55.460

PT10: activity or fitness. Yeah. So I consider. Yeah, it would be a factor for both the terms.

48

00:04:55.610 --> 00:04:56.390

NM: Okay.

49

00:04:56.720 --> 00:04:57.380

PT10: Yeah.

50

00:04:57.580 --> 00:05:05.700

NM: And the last prompt would be. When do you witness? Your students participate most in physical activity during the school day.

51

00:05:07.430 --> 00:05:14.419

PT10: like the time period of the day?

NM: Yeah, like when what activity? Or when do you see them participate most in physical activity during the day.

52

00:05:14.440 --> 00:05:18.259

PT10: when when there's music and

53

00:05:18.410 --> 00:05:20.150

PT10: when they are not hungry

54

00:05:20.170 --> 00:05:21.660

NM: Hmm. Okay.

55

00:05:21.890 --> 00:05:25.590

PT10: And usually the morning times, I would say.

56

00:05:32.120 --> 00:05:38.380

NM: Can you tell me a little bit more about what they're doing during those times when there's music when they're not hungry, and when it is the morning.

57

00:05:39.980 --> 00:05:47.970

PT10: so say, for example, like, if I go in a class, and if that classroom is just done with their circle. Time.

58

00:05:48.210 --> 00:05:53.310

PT10: Their system is, you know, like they are in that zone, and they want to do more.

59

00:05:53.330 --> 00:06:01.200

PT10: So I put up a yoga session or a movement break kind of thing to get them going for a physical activity.

60

00:06:04.900 --> 00:06:10.210

NM: Right. So that is what is these times always with you? Or this is just in the classroom in general.

61

00:06:12.880 --> 00:06:14.210

PT10: I would say

62

00:06:14.350 --> 00:06:15.419

PT10: I

63

00:06:15.540 --> 00:06:18.819

PT10: sometimes I do push-ins, and sometimes I pull out.

64

00:06:19.080 --> 00:06:20.510

PT10: It applies for both.

65

00:06:20.590 --> 00:06:24.070

PT10: It depends on the kind of you know motivation

66

00:06:24.600 --> 00:06:31.210

PT10: that that child needs. If I think that there's too much distraction in in the class, then I would do one on one.

67

00:06:31.470 --> 00:06:35.739

PT10: But if they are happy and I have a group, I go with the group.

68

00:06:37.880 --> 00:06:41.830

NM: So everything you decide to describe earlier on your therapy session.

69

00:06:42.000 --> 00:06:42.920

PT10: Yes.

70

00:06:45.160 --> 00:06:46.829

NM: and question number 2:

71

00:06:46.960 --> 00:06:50.610

NM: How do you measure physical activity, frequency.

72

00:06:50.810 --> 00:06:55.559

NM: intensity, time and type? So have you heard of the fiTT principle?

73

00:06:55.860 --> 00:06:57.090

NM: And

74

00:06:57.440 --> 00:07:05.109

NM: kinda like exercise physiology, right. So this is kind of where I was going with this question. So how would you measure that

75

00:07:05.370 --> 00:07:16.960

NM: with children in children with Cp. Who are not full time, Walkers, or would you not measure it? You could tell me how you would measure it if you do, and if you don't, that's fine, too. Just tell me your thoughts on that.

76

00:07:18.780 --> 00:07:21.129

PT10: It's it's actually really difficult.

77

00:07:21.270 --> 00:07:26.479

PT10: specific, parameter in this particular case, load.

78

00:07:27.410 --> 00:07:34.059

PT10: But all I would say is that, you know it should be consistently every day.

79

00:07:34.410 --> 00:07:36.540

PT10: at least 3 min for

80

00:07:36.700 --> 00:07:38.729

PT10: this range of population.

81

00:07:41.410 --> 00:07:52.510

NM: Okay, great. And do they need assistance to complete these activities, and and and if so, during which activities and do they need assistance for our part or the whole task?

82

00:07:54.150 --> 00:07:54.929

This.

83

00:07:55.060 --> 00:07:56.490

PT10: I feel like

84

00:07:56.620 --> 00:07:59.960

PT10: it just leads to. It depends

85

00:08:00.810 --> 00:08:11.170

PT10: because you never know what's going to hit right, and they might participate. But most of the time for the initiation piece they definitely need

86

00:08:11.190 --> 00:08:13.410

PT10: physical assistance from someone else.

87

00:08:22.620 --> 00:08:33.809

NM: and do you think they should perform more or less in each of these activities? Give me some examples of you gave me some. You talked about initiation. Can you give me an example of activity at first.

88

00:08:33.830 --> 00:08:36.999

PT10: like? Say, for example, if i'm

89

00:08:37.559 --> 00:08:40.320

PT10: taking them for the adaptive bike ride

90

00:08:40.350 --> 00:08:51.950

PT10: you know, like you did. You did the Prep. You. You stretch them out a little. You made made made them comfortable. They are wearing their braces. They have their Thera Togs, whatever it's all on.

91

00:08:52.130 --> 00:08:55.610

PT10: and you put them on. You keep the music on, and

92

00:08:55.720 --> 00:09:08.450

PT10: when they put them in that position. You need to make sure that they know the movement. So you initiate the first peddling for them, and then they they just, you know.

93

00:09:08.480 --> 00:09:11.170

PT10: hopefully they process it, and then

94

00:09:11.350 --> 00:09:12.780

PT10: they engage in it

95

00:09:13.150 --> 00:09:14.930

PT10: to participate in that.

96

00:09:15.590 --> 00:09:17.010

NM: Got it. Okay.

97

00:09:17.040 --> 00:09:18.689

NM: Thank you for that example.

98

00:09:19.330 --> 00:09:21.390

NM: And do you think

99

00:09:22.280 --> 00:09:26.939

NM: they should participate in more or less of these types of activities.

100

00:09:27.420 --> 00:09:28.830

PT10: absolutely more

101

00:09:28.910 --> 00:09:30.870

PT10: the more they get the better

102

00:09:31.200 --> 00:09:35.139

PT10: like based on the neuro-plasticity principle it's like

103

00:09:35.230 --> 00:09:37.070

PT10: it's. It's a

104

00:09:37.280 --> 00:09:39.840

PT10: you know, like it's a no brainer like they should get

105

00:09:39.970 --> 00:09:43.079

PT10: as much time as they can

106

00:09:43.480 --> 00:09:47.550

PT10: in physical activity or any kind of activity.

107

00:09:47.730 --> 00:09:57.849

PT10: Engagement is the basic principle, like once they get more time doing that, there are better chances of, you know, no regeneration and

108

00:09:58.220 --> 00:10:00.120

PT10: helping them to get better.

109

00:10:01.280 --> 00:10:08.790

NM: Great? Thank you all right. Next question. Do you address promoting physical activity during your physical therapy sessions?

110

00:10:09.690 --> 00:10:10.640

PT10: Yes.

111

00:10:12.660 --> 00:10:14.719

NM: how do you do this?

112

00:10:15.880 --> 00:10:23.309

PT10: So the structure which i'm currently working in has, someone called as APE (adaptive physical education teacher)

113

00:10:23.460 --> 00:10:25.859

PT10: adaptive physical education teacher.

114

00:10:25.960 --> 00:10:30.769

PT10: So I, read with apes to work on certain

115

00:10:31.230 --> 00:10:36.390

PT10: kids where I see that, you know. Say, for example, if

116

00:10:37.230 --> 00:10:39.579

PT10: if I'm also training them, for

117

00:10:40.050 --> 00:10:50.369

PT10: you know, hitting with the baseball and working on their lower extremity strength, then once I prep them with the exercises, I would set them up

118

00:10:50.520 --> 00:10:51.919

PT10: and

119

00:10:52.670 --> 00:11:03.459

PT10: me and APE would work together to figure out like what would be the best position for that child to you know, focus on that repetitive motion of hitting with the bat

120

00:11:15.310 --> 00:11:27.200

NM: great. And even with this example, what components of physical activity would you say you address? So let me give you an example. it can be mobility. It can be cardiovascular endurance.

121

00:11:27.240 --> 00:11:32.810

NM: muscle activation. What what are the the areas of physical activity. Do you feel like you're addressing?

122

00:11:34.740 --> 00:11:37.339

PT10: Okay, that would be, you know.

123

00:11:37.460 --> 00:11:39.379

PT10: muscle activation

124

00:11:39.480 --> 00:11:42.320

PT10: and bilateral coordination.

125

00:11:46.230 --> 00:11:53.030

PT10: Those both pieces, I would say, like, based on the example which I gave. Those are the both things which I would focus on

126

00:11:53.080 --> 00:12:04.890

NM: right and on, and that and so on that example. But what would you say You focus on most when you're doing this in your therapy? Is it always those 2, or there in general? Do you have like a favorite area you're working on in terms of the components for

127

00:12:05.030 --> 00:12:12.399

PT10: for specific that population, I would say muscle activation is something we work the most

128

00:12:12.630 --> 00:12:18.640

PT10: that if I see that okay, this is this is definitely giving this specific muscle group

129

00:12:18.810 --> 00:12:22.460

PT10: that activation piece. Then I would definitely work on it more

130

00:12:24.050 --> 00:12:24.889

NM: like, great.

131

00:12:25.570 --> 00:12:37.580

NM: Do you address promoting physical activity? This is the fourth question and the last one before we get to the survey, do you address promoting physical activity that occurs outside of your physical therapy sessions.

132

00:12:38.120 --> 00:12:40.310

PT10: Yes, absolutely.

133

00:12:40.500 --> 00:12:42.400

NM: And how do you do that?

134

00:12:44.170 --> 00:12:54.099

PT10: Okay. So in this scenario it's like APE. But I have in the past worked with the conductive educators. And so there's like different ways of you know.

135

00:12:54.250 --> 00:13:05.569

PT10: showing them like it. It. It doesn't need to be structured in the form of only exercises by physical therapist there are prescribed set of excises which we do.

136

00:13:05.680 --> 00:13:12.770

PT10: But if it is with some recreational activity, there are higher chances of kids getting

137

00:13:13.200 --> 00:13:18.070

PT10: like getting more participation from them If it is engaging. And you know

138

00:13:18.950 --> 00:13:25.409

PT10: more fun, I would say the prescribed set of like, okay, this is the exercise which you gotta do.

139

00:13:26.070 --> 00:13:27.140

PT10: That's what I feel.

140

00:13:27.600 --> 00:13:29.249

NM: that's good.

141

00:13:31.290 --> 00:13:37.979

NM: And have you recommended any community programs or events to your students to help increase physical activity.

142

00:13:39.340 --> 00:13:40.970

PT10: Yes.

143

00:13:41.950 --> 00:13:46.129

PT10: well, it based on my current setting, I would say, like I,

144

00:13:46.220 --> 00:13:49.760

PT10: I do encourage my kids to go for adaptive sports

145

00:13:50.120 --> 00:13:53.790

PT10: and ballet class, like swimming.

146

00:13:55.160 --> 00:14:01.509

PT10: So these all things like. If if I know the resources, I would definitely encourage the parents to go, for

147

00:14:01.600 --> 00:14:03.530

PT10: you know additional activities.

148

00:14:05.230 --> 00:14:07.029

NM: And what type of equipment

149

00:14:07.200 --> 00:14:15.729

NM: have you recommended to help improve home or community engagement in physical activity outside of the clinical setting?

150

00:14:19.950 --> 00:14:23.480

PT10: It's like adaptive bikes first of all.

151

00:14:23.640 --> 00:14:27.250

PT10: then walkers, but like based on

152

00:14:27.810 --> 00:14:30.390

PT10: at at what level the child is

153

00:14:33.260 --> 00:14:40.210

PT10: standing. I I don't know nowadays. I'm not so much inclined for the passive standing.

154

00:14:40.390 --> 00:14:47.039

PT10: So if the parents are, you know they are willing to do it then. Yes, otherwise it's usually done it

155

00:14:47.120 --> 00:14:48.410

PT10: at school

156

00:14:48.530 --> 00:14:54.369

PT10: where they could, you know, focus and work on if some curriculum thing is going on, and they could just be in stander.

157

00:14:56.550 --> 00:14:58.270

PT10: apart from that, I

158

00:14:58.470 --> 00:14:59.520

I Haven't.

159

00:15:00.230 --> 00:15:08.910

PT10: Not other equipment, because everything is insurance based. So you You've got to make sure that it's covered. You don't want to put extra burden on parents

160

00:15:18.470 --> 00:15:21.469

NM: all right. Great. So now i'm going to share my screen.

161

00:15:21.520 --> 00:15:23.510

NM: and i'll show you the tool

162

00:15:23.930 --> 00:15:25.800

PT10: that I want us to talk about.

163

00:15:26.730 --> 00:15:33.260

NM: So i'll put these questions up, can you? I'm going to try to get all 8 questions on there, so you can see it together

164

00:15:33.440 --> 00:15:51.900

NM: all right. So this is called the a promise, parent, proxy, physical activity, short form, survey. So when given to a caregiver or parent, we ask them to respond to each question or statement by marking one box per row. So they're going to say how many days in the past 7 days that this criteria

165

00:15:51.910 --> 00:15:57.609

NM: was appropriate or related to that child's level of physical activity. So what I'm going to ask you

166

00:15:57.940 --> 00:15:58.550

PT10: Hmm.

167

00:15:58.630 --> 00:16:03.870

NM: Is I want you to give me a rating for each question.

168

00:16:04.130 --> 00:16:07.040

and 0 being not related at all

169

00:16:07.080 --> 00:16:10.500

NM: for this population 5 being highly appropriate.

170

00:16:10.700 --> 00:16:18.140

NM: and then it could be along that scale. How would you rate this question? Do you think it's a good one for a parent that has a child with

171

00:16:18.490 --> 00:16:20.509

NM: Cp. Who is not walking

172

00:16:20.720 --> 00:16:28.500

NM: really related or 0 not at all. And or somewhere along the scale. Okay, so we'll go through each question. So the first question is.

173

00:16:29.000 --> 00:16:34.440

NM: How many days is your child exercise or place so hard that his or her body got tired.

174

00:16:34.530 --> 00:16:42.899

NM: Would you rate this 0 not related to all, or up to 5 highly appropriate. How would you rate this question, and why.

175

00:16:46.470 --> 00:16:48.560

PT10: so I will see.

176

00:16:51.850 --> 00:16:58.250

PT10: So it's just for the Pms. Cs: 4 and 5, right? Correct.

177

00:16:58.560 --> 00:17:04.310

PT10: And this survey is basically like for the parents. Yes.

178

00:17:05.880 --> 00:17:13.269

PT10: and I need to read like how it applies. I want you like. So there's not many tools for this population.

179

00:17:13.319 --> 00:17:28.089

NM: right? So this was actually created by the National Institute of Health to be for children that were more involved physically right, not necessarily made for children. With Cp. However, it has been used in that population. Now, what I want to see is

180

00:17:28.109 --> 00:17:33.029

NM: how physical therapy the believe, how valid is this scale

181

00:17:33.450 --> 00:17:36.600

NM: as it relates to measuring physical activity.

182

00:17:36.720 --> 00:17:46.829

NM: intensity for these kids. Now, this survey is asked to parents. So based on what my pts come up back with? We're going to see how appropriate is this tool

183

00:17:47.410 --> 00:17:52.570

NM: for these kids. Okay, but you do have to think about these questions are going to be asked to the parent.

184

00:17:53.180 --> 00:17:56.229

NM: And do you think this question is appropriate

185

00:17:57.040 --> 00:18:00.729

NM: to a talk about physical activity in the kids that are not walking.

186

00:18:01.290 --> 00:18:02.110

NM: Okay.

187

00:18:02.360 --> 00:18:07.530

NM: So the first question is, how many days did your child exercise

188

00:18:08.120 --> 00:18:14.160

NM: or play so hard that his or her body got tired. The parent would have to answer that, based on their understanding

189

00:18:14.200 --> 00:18:19.300

NM: of physical activity in their child. How appropriate do you think that question is for them?

190

00:18:22.520 --> 00:18:24.020

PT10: It's it's one

191

00:18:24.110 --> 00:18:25.889

NM: one, not okay. And why.

192

00:18:27.480 --> 00:18:34.590

PT10: I mean, like, if I go by the number of kids I've seen with this condition. Their parents are half of the time

193

00:18:34.750 --> 00:18:38.489

PT10: they don't see them doing any active.

194

00:18:38.960 --> 00:18:39.780

NM: Okay.

195

00:18:51.170 --> 00:18:52.559

NM: All right. Next question

196

00:18:52.780 --> 00:18:54.210

NM: number 2.

197

00:18:54.240 --> 00:19:00.089

NM: How many days. Did your child exercise really hard for 10 min or more?

198

00:19:00.940 --> 00:19:03.770

NM: How would you rate this 1? 0? Not appropriate

199

00:19:04.140 --> 00:19:05.130

NM: at all?

200

00:19:05.420 --> 00:19:08.030

NM: Up to 5 highly appropriate.

201

00:19:10.130 --> 00:19:10.700

PT10: Hmm.

202

00:19:12.950 --> 00:19:15.550

PT10: I'm. Just trying to see this through

203

00:19:15.640 --> 00:19:19.519

PT10: parents lens. And then i'm thinking me I probably might not.

204

00:19:22.410 --> 00:19:26.740

PT10: I'm not so sure if this scale actually measures anything for these kids.

205

00:19:26.900 --> 00:19:39.170

NM: And that's why i'm asking these questions. So that yeah, that's okay. So I guess i'm trying to see which questions are better than others. Right like you know, we we wanna throw. We don't want to go all the way right? What can what can be used? And then.

206

00:19:39.180 --> 00:19:49.460

NM: and also your reasoning is even more important than it's just as important as your grade. Right? So give me a score. But then let me tell me why you think it's appropriate. And why not

207

00:19:51.420 --> 00:19:52.670

PT10: so?

208

00:19:53.080 --> 00:19:55.130

PT10: Even this like it's

209

00:19:55.490 --> 00:20:01.429

PT10: I. I feel like the first and second question, like it, excise or play really really hard and all that

210

00:20:02.500 --> 00:20:08.179

PT10: with the parents of these kids that just basically doesn't apply because

211

00:20:08.310 --> 00:20:10.910

PT10: they they see no physical like they. Just.

212

00:20:11.010 --> 00:20:12.140

PT10: I would say, like

213

00:20:12.270 --> 00:20:15.179

PT10: only time when I actually got to

214

00:20:15.290 --> 00:20:22.799

PT10: make the parents understand more during the Zoom Meetings that you know your child can actually do this.

215

00:20:22.850 --> 00:20:27.469

PT10: So you better work on it, and you would see that they are doing it.

216

00:20:27.560 --> 00:20:30.209

PT10: So I would say nowadays.

217

00:20:30.680 --> 00:20:37.430

NM: So what you say that's a one like the first one, or because 0 is lower than that, too. Okay.

218

00:20:37.790 --> 00:20:39.740

NM: All right. So

219

00:20:40.900 --> 00:20:55.929

NM: and you I mean just to rephrase. I want to make sure understood. You said, you gave it a one because parents don't see how much their child is able to do physical activity. They needed instruction, or for you as a therapist to help explain it. Right? Yes.

220

00:20:57.580 --> 00:21:01.980

PT10: Well, if 0 is the rating, then you can go for 0 for both the

221

00:21:02.090 --> 00:21:09.179

NM: so you that you wanted to give them the lowest. That's what I was trying to remember. Okay, okay, so lowest is 0. That's not a problem.

222

00:21:10.300 --> 00:21:11.620

NM: 0 to 5.

223

00:21:13.070 --> 00:21:16.240

NM: All right. Now we go to number 3.

224

00:21:18.500 --> 00:21:21.149

NM: Now this one may be interesting. Number 3.

225

00:21:21.180 --> 00:21:26.679

NM: How many days of your child exercise so much that he or she breathe heart breathe hard.

226

00:21:27.230 --> 00:21:28.569

NM: How would you rate this one

227

00:21:31.080 --> 00:21:33.120

PT10: just based on excise

228

00:21:35.180 --> 00:21:40.059

NM: or in? And not necessarily. It could be just physical activity, anything they do that's physical activity.

229

00:21:40.760 --> 00:21:42.239

PT10: But I could say

230

00:21:42.840 --> 00:21:44.690

PT10: 2 or 3 days 3.

231

00:21:45.060 --> 00:21:49.710

PT10: So you give them one number no one number. Would you rate this?

232

00:21:49.760 --> 00:21:50.680

PT10: Yeah.

233

00:21:50.740 --> 00:21:51.660

NM: Because

234

00:21:51.740 --> 00:21:58.979

PT10: the fatigue in this kids can vary, and if they are really involved, then they do tend to breathe hard.

235

00:21:59.220 --> 00:22:03.089

PT10: Okay, that you of the amount of excise they do.

236

00:22:03.490 --> 00:22:04.240

NM: Okay.

237

00:22:04.480 --> 00:22:07.160

NM: and it can be assessed by the Pam, the parent.

238

00:22:08.880 --> 00:22:09.970

PT10: Yeah.

239

00:22:10.410 --> 00:22:14.279

PT10: I mean, like as far as the parents which I have dealt with.

240

00:22:14.390 --> 00:22:15.240

PT10: I feel like

241

00:22:15.590 --> 00:22:17.029

PT10: they cannot assess it.

242

00:22:17.700 --> 00:22:19.890

NM: Okay. So you don't think they can assess it. Okay.

243

00:22:24.080 --> 00:22:26.869

NM: So may or may not be able to be assessed.

244

00:22:28.130 --> 00:22:29.839

NM: Yeah, they can't. Okay.

245

00:22:31.670 --> 00:22:37.969

PT10: maybe. Yeah, I would say, yeah, May or may not. Because if the parent is really involved and

246

00:22:38.290 --> 00:22:42.839

PT10: is aware of its condition, then yeah, why not? So may or may not

247

00:22:43.150 --> 00:22:45.010

PT10: is a better approach. Yeah.

248

00:22:46.040 --> 00:22:47.550

PT10: okay, Number 4.

249

00:22:47.920 --> 00:22:53.439

NM: How many days was your child so physically active that he or she sweated. How would you rate this question

250

00:23:02.240 --> 00:23:03.090

PT10: to you.

251

00:23:03.230 --> 00:23:04.590

NM: 0. Why

252

00:23:08.770 --> 00:23:15.630

PT10: so, Nia? This is based on their you know, like this question. I would be asked to parents when they are at home.

253

00:23:15.940 --> 00:23:20.030

PT10: or at any setting. What is the setting.

254

00:23:21.270 --> 00:23:28.960

NM: But we can just go with your setting and make it easier right? So you know how we used to put sometimes, you know you put a assessment in a bag

255

00:23:29.190 --> 00:23:30.530

NM: and sitting home

256

00:23:30.560 --> 00:23:38.030

NM: so it could be like that, or you can actually sit there and give it to a parent and ask them to give you the score

257

00:23:38.260 --> 00:23:47.649

NM: Right? What is their opinion on these things? And I think what i'm trying to get at for the therapist is, you guys, just look at the question. And how appropriate is this question

258

00:23:47.860 --> 00:23:55.869

NM: to assess intensity in the kids that are not able to emulate the for the the Gms. Is all formed with Cp.

259

00:23:56.060 --> 00:24:05.760

NM: So i'll be specific children with some real quality. So how would you rate this question? So yeah, I understand why. Why you may say 4 is a 0. But why do you think so?

260

00:24:06.220 --> 00:24:07.970

NM: As it relates.

261

00:24:10.090 --> 00:24:15.059

NM: I mean, I got. I got a lot of different answers so it could be. It. Can, you know, just curious to know what your thoughts are.

262

00:24:15.970 --> 00:24:17.790

PT10: Yeah, I just feel like

263

00:24:19.070 --> 00:24:22.109

PT10: so wedding with physical activity like

264

00:24:22.680 --> 00:24:32.490

PT10: it. It also depends on the physiological function that I don't know if i'm thinking in the right way or not. But if you

265

00:24:32.550 --> 00:24:38.140

PT10: and it could be a physiological response, yeah, some some kids, some kids don't

266

00:24:38.970 --> 00:24:39.730

PT10: like.

267

00:24:43.380 --> 00:24:50.470

PT10: but I would. I would put a 0, because, like my perception for those parents is like they don't

268

00:24:51.160 --> 00:24:56.360

PT10: in like, you know, if they're aware, and they're already educated

269

00:24:56.480 --> 00:25:01.739

PT10: about the child's condition, and they are working actively with the 10%, the team

270

00:25:02.040 --> 00:25:09.130

PT10: to promote the physical activity, then it applies. But if you like, that, is there, then this is 0.

271

00:25:11.610 --> 00:25:16.729

NM: So in that situation where it it applies. How would you rate this question

272

00:25:19.270 --> 00:25:21.190

PT10: if it applies? Yes.

273

00:25:22.250 --> 00:25:25.930

PT10: the flies, then I would say

274

00:25:27.110 --> 00:25:29.490

PT10: these 2 to 3, not a 3.

275

00:25:30.300 --> 00:25:31.080

NM: Okay.

276

00:25:31.500 --> 00:25:35.919

NM: So. And why would you give it a 3 if it applies in the most ideal circumstance?

277

00:25:37.270 --> 00:25:43.679

PT10: So if the parent is aware of the activity, then they would see sweating, or whatever

278

00:25:43.920 --> 00:25:44.870

PT10: or

279

00:25:45.550 --> 00:25:52.260

PT10: the child. Whenever, like, say, for example, they have an active bike at home, or they have stationary biker.

280

00:25:52.290 --> 00:25:55.239

PT10: They are trying to engage the kids and

281

00:25:55.320 --> 00:25:58.459

PT10: make it work for the physical activity part.

282

00:25:58.490 --> 00:25:59.540

PT10: Then

283

00:26:00.300 --> 00:26:01.949

PT10: it it would be

284

00:26:01.990 --> 00:26:03.859

approximately 2 or 3 days.

285

00:26:04.710 --> 00:26:08.020

NM: No, it's not about the day, so i'm not asking you rate the days.

286

00:26:09.140 --> 00:26:11.959

NM: It's more so like, how would you write this question

287

00:26:14.490 --> 00:26:24.810

NM: if I read it then. That's fine. No, no, there's no wrong answer that that's what I want you to know, because you you have the experience. You are the wealth of knowledge. So I want to get what

288

00:26:25.210 --> 00:26:27.300

NM: what you have experience.

289

00:26:27.500 --> 00:26:38.109

NM: you know Why? Because then maybe I can give this information to the developers of this test, right? Or maybe they can make a new test as appropriate, you know. So it's okay

290

00:26:38.170 --> 00:26:40.809

NM: to just tell me what you're thinking. Okay.

291

00:26:42.000 --> 00:26:46.260

NM: And so if you're saying that this just doesn't apply, because most of the time time

292

00:26:46.510 --> 00:26:55.250

NM: it could be physiological or some of these kids just don't sweat because of their dysfunction. Right? That's what I hear you saying. I just don't want to put words.

293

00:26:55.770 --> 00:27:00.640

PT10: Yeah, no, that that's what my initial thought process was. That

294

00:27:00.830 --> 00:27:04.859

PT10: it's. If it's a physiological response it that just does not apply

295

00:27:04.900 --> 00:27:14.380

NM: that that's no problem. We're gonna stick with your original answer. Because I understand what you're saying, alright, so Number 5 is only it's only going to get better. The question is getting

296

00:27:15.020 --> 00:27:17.489

NM: no

297

00:27:17.670 --> 00:27:19.780

NM: Number 5 is

298

00:27:21.250 --> 00:27:34.030

NM: How many days is your child exercise or play so hard that his or her muscle burn. How appropriate 0? Not at all. 5 highly appropriate to ask a parent, as it relates to a child that has

299

00:27:34.600 --> 00:27:40.020

NM: it's at double 5,

300

00:27:43.410 --> 00:27:51.440

NM: I mean, like this is not applicable for these children.

301

00:27:51.920 --> 00:27:57.729

PT10: because muscle activation first of all is so difficult when you work with.

302

00:27:57.770 --> 00:28:06.379

PT10: Once you get that, you go to work on the repetition and asking a parent that playing so hard, exciting, so hard like they.

303

00:28:07.280 --> 00:28:09.400

PT10: No, it's it's a 0

304

00:28:09.750 --> 00:28:12.020

PT10: like. Yeah.

305

00:28:13.490 --> 00:28:17.130

PT10: I would put that like. If if the parents are

306

00:28:17.500 --> 00:28:21.559

PT10: in general, there's so less amount of information about this.

307

00:28:22.190 --> 00:28:28.709

PT10: and when parent faces it. They are like they are like, I would say they. They don't know what to do.

308

00:28:29.020 --> 00:28:34.590

PT10: so they totally rely on. The information is given by their doctors or

309

00:28:34.690 --> 00:28:39.999

PT10: whoever is in their team. They'll go one by one to each each of them, and then

310

00:28:40.160 --> 00:28:44.429

PT10: then also they wouldn't have that much pool of information

311

00:28:44.500 --> 00:28:46.889

PT10: to understand what's going on with the chat.

312

00:28:47.620 --> 00:28:50.759

PT10: They are participating regularly.

313

00:28:51.230 --> 00:28:52.340

PT10: You see.

314

00:28:53.020 --> 00:28:58.159

PT10: if at all, the progress is being made, because progress takes time in this case.

315

00:29:00.250 --> 00:29:02.590

PT10: So this question.

316

00:29:03.010 --> 00:29:05.610

PT10: Yeah, it's it's going to be 0 for

317

00:29:05.630 --> 00:29:06.700

PT10: more stuff

318

00:29:06.760 --> 00:29:08.469

PT10: like even this, like

319

00:29:08.560 --> 00:29:15.909

NM: Number 6. How many days did your child exercise or play so hard that he or she felt tired.

320

00:29:18.460 --> 00:29:19.680

PT10: It's a 0.

321

00:29:19.900 --> 00:29:20.520

NM: Okay?

322

00:29:21.510 --> 00:29:26.939

PT10: because, like I I I just feel like this is this is a

323

00:29:27.130 --> 00:29:30.619

PT10: I don't know why this question that is even made for these kids.

324

00:29:31.000 --> 00:29:34.679

PT10: I mean like, can it? It's just not applicable for this population.

325

00:29:34.820 --> 00:29:35.970

PT10: It's that simple.

326

00:29:37.470 --> 00:29:40.179

NM: And for this question, though anything specific

327

00:29:40.750 --> 00:29:58.430

NM: cause this one is about Number 6 was about a parent being able to tell you how many days that they feel their child exercise, or played so hard that he or she felt tired, like a parent being able to say, oh, they got! They were really tied on 2 or 3 days a week, we read. But this question is number 6.

328

00:29:58.440 --> 00:30:01.130

PT10: First question. No, it's the first question. See

329

00:30:01.210 --> 00:30:14.750

NM: how many of these different Number 6 is a little to the number. One is they're close. They're close to what I will agree with you. So number One was, how many days. You're talking about anybody that tired. And this one is, if your child.

330

00:30:14.960 --> 00:30:17.659

PT10: they that your child

331

00:30:18.550 --> 00:30:23.540

PT10: Yeah, they're very close. So you know one is about

332

00:30:23.880 --> 00:30:29.170

NM: feeling if the parent can determine if their body was tied versus if they still the child felt tired.

333

00:30:30.370 --> 00:30:31.289

PT10: it's a 0.

334

00:30:31.550 --> 00:30:32.350

NM: Okay.

335

00:30:32.690 --> 00:30:34.320

NM: And number 7.

336

00:30:34.830 --> 00:30:38.600

NM: How many days. Was your child physically active for 10 min or more.

337

00:30:47.730 --> 00:30:48.690

PT10: Hmm.

338

00:30:49.870 --> 00:30:58.820

NM: It's like the 0, not at all related, or 5 highly appropriate, like

339

00:30:59.070 --> 00:31:03.200

NM: Number 8. I Number 8 is tough. So that's the answer.

340

00:31:03.390 --> 00:31:11.100

PT10: Yeah, that that's that's not applicable. The last I got you for Number 8, but number 7. What number would you get it?

341

00:31:11.290 --> 00:31:12.080

NM: Give it

342

00:31:12.430 --> 00:31:13.440

PT10: 0.

343

00:31:14.110 --> 00:31:15.570

NM: Okay. And why?

344

00:31:15.860 --> 00:31:17.390

PT10: Because,

345

00:31:17.810 --> 00:31:26.790

PT10: to understand that, you know, like Cardio, pulmonary fitness is also a thing you need to make sure, and is aware that their child is not breathing proper.

346

00:31:27.260 --> 00:31:31.770

PT10: and it's a whole set of different training which we need to make sure that

347

00:31:31.900 --> 00:31:37.490

PT10: they are aware that this is something which the child is doing physically, actively.

348

00:31:37.930 --> 00:31:38.700

PT10: So

349

00:31:38.890 --> 00:31:44.410

PT10: I I consider this.

350

00:31:44.510 --> 00:31:45.630

PT10: The

351

00:31:45.930 --> 00:31:47.800

PT10: Yeah, Gmf: C. Is 4 and 5.

352

00:31:48.430 --> 00:31:49.380

NM: Okay.

353

00:31:52.060 --> 00:31:56.290

NM: got it all right. So that is it. You gave me the answer for Number 8,

354

00:31:56.310 --> 00:32:01.040

NM: and the way I like to end is, do you have any final thoughts that you'd like to add about

355

00:32:01.080 --> 00:32:04.900

NM: physical activity in this population any last faults and comments?

356

00:32:06.450 --> 00:32:09.499

PT10: Are you going to make a new scale for these kids?

357

00:32:09.750 --> 00:32:17.730

NM: You know what you're not the first person to ask me that I don't have not to finish my degree, but this is the start.

358

00:32:17.800 --> 00:32:28.490

NM: So this is how we start seeing what their, if it's believe, and the next part will be interviewing parents and seeing what they believe, and I think it's going to be a real interesting

359

00:32:28.560 --> 00:32:31.520

NM: bunch of interviews bringing this to parents.

360

00:32:31.720 --> 00:32:33.930

PT10: Yeah. So yeah.

361

00:32:34.280 --> 00:32:40.279

PT10: Because I I think like, if if at all, something could be measured

362

00:32:40.850 --> 00:32:51.920

PT10: in this population would definitely be, You know, Cardio, pulmonary fitness. We can parameters. We can find out. You know how they are engaging in it.

363

00:32:52.040 --> 00:33:00.080

PT10: how much improvement we can get with that because part of Cardio pulmonary aspect is involuntary. So if

364

00:33:00.120 --> 00:33:05.040

PT10: this is working in proper manner, you'll see changes faster

365

00:33:05.150 --> 00:33:07.520

PT10: of this

366

00:33:07.550 --> 00:33:12.810

PT10: criteria, which actually is just. For you know, normal kids, you cannot apply this person.

367

00:33:13.320 --> 00:33:14.020

PT10: Yeah.

368

00:33:17.720 --> 00:33:20.649

NM: thank you so much. I will stop the recording.

369

00:33:24.080 --> 00:33:25.450

PT10: It is.
