# Supplementary material for: Validity of PROMIS® Pediatric Physical Activity Parent Proxy Short Form Scale as a Physical Activity Measure for Children with Cerebral Palsy Who Are Non-Ambulatory
Source: Behav Sci (Basel). 2025 Jul 31;15(8):1042. doi: 10.3390/bs15081042 (PMC12382615; doi:10.3390/bs15081042)
Supplement: Supplementary file 1 [file behavsci-15-01042-s001.zip › Transcripts copy/PT transcripts - deidentified/PT11.docx]

WEBVTT

1

00:00:01.270 --> 00:00:23.530

NM: All right. Thank you so much for joining us today, PT11. Today we're going to talk a bit about physical activity for children with Cp. Who are not full time workers walkers. So I have a couple of questions for you. and then I have some prompts for each question, so bear with me. I will ask you a question if some of this repetitive. it's just kind of me following the script. Okay.

2

00:00:23.540 --> 00:00:29.610

NM: all right. So the first question is, how do you define physical activity for children with Cp.

3

00:00:29.790 --> 00:00:31.969

NM: Who are not full-time walkers?

4

00:00:34.620 --> 00:00:39.610

PT11: I would define any movement outside of their chairs.

5

00:00:41.120 --> 00:00:48.480

PT11: That lets them get to like a desired object or desired toy. Let's say they're on the

6

00:00:48.850 --> 00:00:49.770

PT11: Mat.

7

00:00:49.790 --> 00:00:51.810

PT11: and the

8

00:00:51.900 --> 00:00:59.170

PT11: child is able to roll to one side just to get to a toy. It's I wouldn't classify that as a physical activity.

9

00:01:01.400 --> 00:01:17.610

NM: Thank you. As a first follow up the Department of Health defines physical activity as any activity that encompasses energy, expenditure, and activation of skeletal muscles. Does Does this definition change your mind about how you define physical activity.

10

00:01:18.110 --> 00:01:21.440

PT11: Not at all. I think that goes very well with

11

00:01:21.700 --> 00:01:22.969

PT11: like a specific

12

00:01:22.990 --> 00:01:29.259

PT11: movement that I describe, but, like the overall or general description, would be

13

00:01:30.740 --> 00:01:32.179

PT11: totally agreeable.

14

00:01:34.290 --> 00:01:39.810

NM: great. And how do you think physical activity differs from other types of fitness? Activity?

15

00:01:42.120 --> 00:01:43.730

PT11: hmm.

16

00:01:46.790 --> 00:01:55.230

PT11: So Physical activity. Is it encompass like any movement, let's say, even within just within

17

00:01:55.320 --> 00:02:03.639

PT11: students sitting in their wheelchair and reaching for a communication device some compasses and activity.

18

00:02:03.660 --> 00:02:06.870

PT11: a physical activity, I would say, physical fitness

19

00:02:07.180 --> 00:02:08.940

PT11: probably is like more

20

00:02:09.030 --> 00:02:13.159

PT11: like higher intensity exercises and higher energy

21

00:02:13.530 --> 00:02:14.969

PT11: requirement as well.

22

00:02:17.980 --> 00:02:24.449

NM: Thank you. When do you witness your students participate most in physical activity during the school day.

23

00:02:29.270 --> 00:02:32.499

PT11: During usually, I would say, there's

24

00:02:32.530 --> 00:02:37.870

PT11: engaged most of the time, so during classroom activities they use their communication device.

25

00:02:37.890 --> 00:02:51.639

PT11: So they use switches. They use their hands. They use their heads. I would say, mostly in pt ot sessions is when they're out of their wheelchairs. So that's when I see

26

00:02:51.790 --> 00:02:54.350

PT11: more bigger movement. It's like

27

00:02:54.370 --> 00:02:57.039

PT11: walking, using a gait trainer or

28

00:02:57.320 --> 00:02:59.909

PT11: biking, or even crawling on the mat.

29

00:03:06.930 --> 00:03:08.630

NM: All right. Next question.

30

00:03:08.690 --> 00:03:15.019

NM: How do you measure physical activity, frequency, intensity, time and type?

31

00:03:15.150 --> 00:03:25.600

NM: And that is the fitt principle. I'm quoting there, and children with Cp. Who are not full time. Walkers. I'll say it again. How do you measure physical activity, frequency.

32

00:03:25.790 --> 00:03:27.180

NM: intensity.

33

00:03:27.500 --> 00:03:29.290

NM: time and type.

34

00:03:29.510 --> 00:03:32.950

NM: and children with Cp. That are at level 4 or 5.

35

00:03:33.130 --> 00:03:34.560

NM: Not full time Walkers

36

00:03:35.930 --> 00:03:37.870

PT11: frequency.

37

00:03:38.120 --> 00:03:45.890

PT11: so we, when we like, set a goal for them. So we write like, how often let's say

38

00:03:45.930 --> 00:03:49.550

PT11: we want to see 20 min of standing

39

00:03:49.960 --> 00:03:52.680

PT11: for 5 opportunities.

40

00:03:54.700 --> 00:04:05.140

PT11: So it can be. We can do as a measurement of those goals on a weekly basis, or it could be measuring that single

41

00:04:05.210 --> 00:04:07.840

PT11: during quarterly assessments.

42

00:04:08.230 --> 00:04:11.120

PT11: I think I missed two.

43

00:04:11.680 --> 00:04:12.400

PT11: Or

44

00:04:13.520 --> 00:04:14.990

PT11: did I answer all of this?

45

00:04:15.980 --> 00:04:25.500

NM: Yeah, no, that's great. How about intensity. So that was the perfect example of frequency. But in terms of like, how how hard they're working. How do you measure that? And these kids?

46

00:04:25.800 --> 00:04:41.210

PT11: So intensity is changing like the duration. Let's say we're doing prone on hands just seeing, if doing prone on hands. Can the students do, shifting to one hand and reaching with the other.

47

00:04:41.410 --> 00:04:44.719

PT11: or just sustaining weight, shifting

48

00:04:44.870 --> 00:04:49.499

PT11: or even walking on hands, if that's something that they can do

49

00:04:50.890 --> 00:04:58.309

NM: great. So I just wanna make Sure, i'm clear. So like maybe changing the level of the task you said you mentioned prone on hands.

50

00:04:58.400 --> 00:05:00.420

PT11: Yeah, let's say prone on hands

51

00:05:00.510 --> 00:05:07.730

PT11: shifting shape to one hand and reaching with the other, or even walking on hands like a wheelbarrel walk.

52

00:05:09.610 --> 00:05:10.669

NM: Okay, great.

53

00:05:10.990 --> 00:05:23.670

NM: Now do they need assistance? You gave some good examples of some tasks complete these tasks and which activities do they need assistance, and do they need assistance for just part of the task, or for the entire task.

54

00:05:24.710 --> 00:05:31.220

PT11: Hmm. Most of my students in my case, so would need assistance for most of it.

55

00:05:31.400 --> 00:05:33.420

PT11: That could be

56

00:05:36.140 --> 00:05:39.890

PT11: I have some students that would require maximum assistance the whole time.

57

00:05:40.300 --> 00:05:41.840

PT11: but

58

00:05:42.220 --> 00:05:47.079

PT11: there's also I also have students that can do contact, guard, assist.

59

00:05:47.300 --> 00:05:48.200

PT11: or

60

00:05:48.340 --> 00:05:49.950

PT11: just close supervision.

61

00:05:56.200 --> 00:06:02.999

NM: Great do they? I mean, do you think they should perform more or less In each of the activities you mentioned? And why?

62

00:06:05.380 --> 00:06:10.540

PT11: Hmm, I think it would really depend, because

63

00:06:10.930 --> 00:06:18.020

PT11: the endurance is really low. So if we, if I see that the student is getting tired easily, then I

64

00:06:18.510 --> 00:06:27.329

PT11: try to do a different exercise. Maybe just do some vestibular, just bouncing on the ball, or just.

65

00:06:27.410 --> 00:06:31.999

PT11: But if I see that, let's say after one set, then they can do it again.

66

00:06:32.020 --> 00:06:36.410

PT11: Then we go back to the same activity and challenge it a little more

67

00:06:44.420 --> 00:06:47.230

NM: great. Thank you. All right. Next question.

68

00:06:47.480 --> 00:06:52.409

NM: Do you address promoting physical activity during your physical therapy sessions.

69

00:06:52.460 --> 00:06:55.160

NM: And if yes, how do you do this?

70

00:06:56.390 --> 00:07:00.779

PT11: Yes, definitely. so most of our students are.

71

00:07:01.340 --> 00:07:08.500

PT11: So they're mostly in their chairs throughout the day, with the even in the classroom activities.

72

00:07:08.520 --> 00:07:11.010

PT11: in classroom meetings. So

73

00:07:11.050 --> 00:07:12.970

PT11: it is mostly during PT

74

00:07:13.110 --> 00:07:15.810

PT11: that they come out of their chairs.

75

00:07:16.040 --> 00:07:18.730

PT11: so even just

76

00:07:19.110 --> 00:07:28.740

PT11: coming out of their chair and sitting on a bench and doing some trunk mobility stretching or just overhead. Reaching

77

00:07:28.780 --> 00:07:32.130

PT11: is definitely something that we focus on.

78

00:07:39.600 --> 00:07:53.089

NM: and what components of physical activity would you say you're addressing? You can use the tasks that you gave as an example to Ben setting, and when I what I mean like components of physical activity. Some examples are cardiovascular endurance.

79

00:07:53.100 --> 00:08:00.590

NM: muscle, activation, energy, expenditure. There's so many things you could be working on. What components do you feel like you're really addressing in your sessions?

80

00:08:01.520 --> 00:08:05.370

PT11: I would say, mostly muscle activation, because I want to see

81

00:08:05.510 --> 00:08:09.059

PT11: like more trunk stability

82

00:08:09.120 --> 00:08:12.809

PT11: if they are not supported by a chest harness.

83

00:08:13.030 --> 00:08:14.860

PT11: or

84

00:08:17.160 --> 00:08:18.840

PT11: the laterals, too.

85

00:08:20.910 --> 00:08:24.429

PT11: just really seeing more upright trunk with

86

00:08:25.510 --> 00:08:28.900

PT11: hopefully. A good, fair to good head control.

87

00:08:34.669 --> 00:08:35.970

NM: Great, Thank you.

88

00:08:36.100 --> 00:08:43.660

NM: And Well, you said yes, so I don't like that. So. this is the last question before we get to the survey.

89

00:08:43.830 --> 00:08:55.169

NM: do you? Physical activity that occurs now outside of your Pt session? We talked about what you do in a pt session. What do you? How do you address this outside of Pt. Sessions?

90

00:08:55.800 --> 00:09:05.720

PT11: yeah. So outside of Pt sessions the students will have either a standing program or a walking program. So we

91

00:09:06.480 --> 00:09:10.000

PT11: train the Paras that are assigned to the students

92

00:09:10.100 --> 00:09:13.410

PT11: to use the equipment on top of the Pt. Session.

93

00:09:13.430 --> 00:09:19.459

PT11: or, if the time allows. Then we set up after the Pt. Session. We set up a student

94

00:09:19.540 --> 00:09:22.949

PT11: on the Stander for another 45 min an hour.

95

00:09:23.080 --> 00:09:26.969

PT11: aiming 3 times to 5 times a week.

96

00:09:27.310 --> 00:09:29.890

PT11: and the other things. If also the student

97

00:09:30.060 --> 00:09:37.340

PT11: is able to, then they can also go on the bike and just bike around the school hallways.

98

00:09:46.390 --> 00:09:47.669

NM: Great, Thank you.

99

00:09:47.780 --> 00:09:53.979

NM: Have you recommended any community programs or events to your students to help increase physical activity?

100

00:09:57.490 --> 00:10:15.359

PT11: Not directly community program. But we send out recommendations to parents on what to work on, just to carry over what we've been working on at school. So let's say just positioning a student in

101

00:10:15.370 --> 00:10:21.330

PT11: quadruped, or just going back to the same example of sitting, maybe just finding a seat without

102

00:10:21.480 --> 00:10:23.329

PT11: any back support at home.

103

00:10:24.030 --> 00:10:28.860

PT11: and just practice the same skills that we we are working on during the session, too.

104

00:10:33.760 --> 00:10:42.569

NM: And what types of equipment have you recommended to help improve home and community engagement and of physical activity outside of the clinical setting.

105

00:10:45.960 --> 00:10:52.209

PT11: mainly, I record. I usually recommend a therapy ball for home, but it can get

106

00:10:52.260 --> 00:10:58.959

PT11: challenging for parents. But other equipment that I was able to get to

107

00:10:59.050 --> 00:11:03.800

PT11: students at home is I was able to get a bike for them.

108

00:11:04.120 --> 00:11:07.260

PT11: and it's mostly standers

109

00:11:07.740 --> 00:11:09.790

PT11: and gait trainers to.

110

00:11:09.850 --> 00:11:12.709

PT11: just because the bike is like it just takes

111

00:11:12.730 --> 00:11:14.500

PT11: too much space at home.

112

00:11:14.520 --> 00:11:19.530

PT11: although most parents are interested in it. They are also like.

113

00:11:19.580 --> 00:11:23.009

PT11: can work more on a stander or a trainer at home.

114

00:11:30.930 --> 00:11:40.350

NM: And so you were saying so. Bikes usually take up more space. You meet specifically because we're in this this demographic like in New York.

115

00:11:40.670 --> 00:11:45.299

PT11: Yes, but I have I have one specific student that

116

00:11:46.020 --> 00:11:52.130

PT11: They're not in an …they are in a house, so it's. It's easier for them to

117

00:11:52.450 --> 00:11:55.340

PT11: kind of follow through on that

118

00:11:55.640 --> 00:11:58.410

PT11: process, because the students learn

119

00:11:58.600 --> 00:12:07.269

PT11: cycling in school, and Mom was really interested in in in carrying that over, and she really find

120

00:12:08.110 --> 00:12:11.650

PT11: like. What do you call that NM like

121

00:12:11.970 --> 00:12:18.209

NM: on funding or like on current.

PT11: Yes, like thing, they were able to find it. So I just have to submit all the

122

00:12:18.370 --> 00:12:21.530

PT11: letters that they need at that time.

123

00:12:21.740 --> 00:12:22.610

NM: Okay.

124

00:12:23.000 --> 00:12:28.689

PT11: but most mostly they're interested. But just because of the space it doesn't really

125

00:12:29.020 --> 00:12:30.749

PT11: it doesn't really work.

126

00:12:31.090 --> 00:12:31.950

NM: But

127

00:12:32.590 --> 00:12:38.430

NM: yeah, okay, thank you. So now we are in the second half i'm gonna pull up

128

00:12:40.410 --> 00:12:43.000

NM: the survey I was telling you about.

129

00:12:43.580 --> 00:12:45.130

NM: and

130

00:12:45.820 --> 00:12:48.459

NM: take a moment to look at the questions.

131

00:12:49.140 --> 00:12:50.660

NM: And then I ask you.

132

00:12:52.000 --> 00:12:57.800

NM: Okay, there you go. So there's 8 questions here. This is called the Promise

133

00:12:58.480 --> 00:13:03.779

NM: Parent proxy physical activity scale. So this was a scale created by the National Institute of Health.

134

00:13:03.920 --> 00:13:09.379

NM: this one specifically, was created for children that were had.

135

00:13:10.350 --> 00:13:11.820

NM: and more involvement.

136

00:13:12.050 --> 00:13:22.920

NM: it physical involvement. So that's why the parent can answer, and it's supposed to really determine or just give a good understanding about the level of intensity a child has

137

00:13:22.990 --> 00:13:29.539

NM: engaged in that physical activity over the week. So the parent or the caregiver would fill this out. And

138

00:13:29.660 --> 00:13:34.930

NM: what we need to talk about is how appropriate. Do we really think this is for children

139

00:13:35.120 --> 00:13:37.110

NM: that are not walkers.

140

00:13:37.240 --> 00:13:41.639

NM: or is the Cp. That are at level? 4 and 5?

141

00:13:41.890 --> 00:13:50.050

NM: So my first for each question. I'm going to ask you to, grade it on a scale of 0 to 5 0, meaning

142

00:13:50.140 --> 00:14:00.909

NM: this is not appropriate at all, not at all related to this population, and Number 5 would be highly appropriate. So you can give me a scale a number along that scale.

143

00:14:01.020 --> 00:14:01.990

NM: All right.

144

00:14:02.830 --> 00:14:04.660

PT11: Sound good.

145

00:14:05.370 --> 00:14:17.650

NM: Is there a way to make this a little bigger? Oh, yeah, absolutely no. I just I try to get it. I could. Yeah, because I can scroll. I just want to use you all we can go through them outside. Now. Yeah.

146

00:14:17.780 --> 00:14:22.460

NM: is that good? So that's the first 1, 4, 5, 6, and then i'll scroll down for the last 2.

147

00:14:23.190 --> 00:14:31.779

NM: All right. So for the first question, how many days did your child exercise or place so hard that his or her body got tired. Would you rate that

148

00:14:31.920 --> 00:14:36.409

NM: as a 0? It's not related for this population 5 would be highly appropriate

149

00:14:36.930 --> 00:14:40.579

NM: anything along the scale. What would you give it? What number would you rate it? Why.

150

00:14:45.080 --> 00:14:47.360

PT11: hmm, I would say a 3.

151

00:14:50.550 --> 00:14:52.100

NM: Okay? And why? Thank you.

152

00:14:55.560 --> 00:15:03.530

PT11: I what I typically notice with my student you can only say, for my student is

153

00:15:04.500 --> 00:15:05.740

PT11: when they get

154

00:15:05.850 --> 00:15:09.419

PT11: this we're talking about home right now. This is home setting

155

00:15:09.760 --> 00:15:19.290

NM: the parent could, would fill it out, but they it would be from the week, so it include school. If they went to school or they were home it can include. It depends on the child in the setting.

156

00:15:19.330 --> 00:15:22.870

NM: but it ideally. This is just a parent reporting on what they think.

157

00:15:23.030 --> 00:15:26.729

NM: If their child is in school for a week, it would include the full time. Yes.

158

00:15:26.820 --> 00:15:28.920

PT11: yeah, I would put 5.

159

00:15:30.900 --> 00:15:39.940

PT11: Okay, so 5, if it includes the school if it includes the school. If it's not. If i'll do just home, I would say a 3.

160

00:15:41.310 --> 00:15:46.979

PT11: I notice if my students has a sitting system at

161

00:15:47.220 --> 00:15:49.569

PT11: home other than the wheelchair.

162

00:15:50.150 --> 00:15:59.230

PT11: They also just stay. They stay in the in their sitting system. I also have a student that goes on the math right away.

163

00:15:59.430 --> 00:16:03.909

PT11: and rolling is his primary mobility. But no

164

00:16:04.430 --> 00:16:07.409

PT11: like after school there's

165

00:16:07.870 --> 00:16:11.320

PT11: no additional physical activity provided.

166

00:16:13.950 --> 00:16:22.110

NM: so i'll clarify the survey. so you think it's a good question to ask the parent whether they're a score or not. Do you think it's an appropriate

167

00:16:22.170 --> 00:16:24.200

NM: question that they are able to answer

168

00:16:24.420 --> 00:16:25.740

NM: for these kittles.

169

00:16:26.460 --> 00:16:27.300

PT11: Yes.

170

00:16:27.350 --> 00:16:28.660

NM: okay. But

171

00:16:28.790 --> 00:16:30.770

NM: because the parent doesn't go to school.

172

00:16:30.840 --> 00:16:32.980

NM: So I guess the question is

173

00:16:37.070 --> 00:16:45.930

NM: so. This is tough that your first person asked me about like comparing it to school at home. So I guess, in general for the whole survey.

174

00:16:45.990 --> 00:16:50.610

NM: I want you to look at it like if the parent was somebody you knew

175

00:16:50.690 --> 00:16:56.080

NM: like one of your parents, one of your kids. When you're study the parent of one of your kids that you, I mean

176

00:16:56.390 --> 00:16:58.390

NM: if you were to hand them this survey

177

00:16:58.970 --> 00:17:00.250

PT11: right

178

00:17:00.270 --> 00:17:01.889

NM: of these questions.

179

00:17:02.010 --> 00:17:03.730

NM: how appropriate would it be

180

00:17:04.130 --> 00:17:07.119

NM: right, the the how confident would you be in these questions.

181

00:17:07.180 --> 00:17:13.500

NM: So let me let me that help kind of clarify it. So if it was a parent you gave this survey to

182

00:17:14.349 --> 00:17:16.480

NM: how about that first question?

183

00:17:16.740 --> 00:17:20.960

NM: How many days your child exercise a play so hard that his or her body got tired?

184

00:17:24.000 --> 00:17:25.069

PT11: Hmm.

185

00:17:26.089 --> 00:17:28.960

PT11: I would say 5 okay is

186

00:17:29.400 --> 00:17:36.289

PT11: when they get all their sessions at school, because the parents would know that, too, and what they usually report to us is

187

00:17:36.390 --> 00:17:40.949

PT11: they get. They get home, they eat, and there's they just sleep right away.

188

00:17:41.960 --> 00:17:44.150

PT11: I think that would be my gauge there.

189

00:17:45.180 --> 00:17:49.830

NM: Okay, so we'll go with 5. A final answer.

190

00:17:51.330 --> 00:17:56.169

NM: I I hear what you're saying with if they were just home, let's say, all week.

191

00:17:56.580 --> 00:18:00.760

NM: but the parents should, may be able either way the parent is able to gauge

192

00:18:00.780 --> 00:18:03.669

NM: how many days, or how how that child

193

00:18:03.700 --> 00:18:13.820

NM: played so hard at their body got tired. So you think of it, a parent will be able to gauge that. That's a 5. Okay, that that's how we're going to rate the rest of them like the parent kind of day each

194

00:18:13.840 --> 00:18:16.690

NM: The answer to this question, okay. So the second one.

195

00:18:17.010 --> 00:18:25.100

NM: How many days your child exercise really hard for 10 min or more. Is this appropriate in this population? Would you say 0? Not at all

196

00:18:25.220 --> 00:18:28.099

NM: 5 highly appropriate, or somewhere along the middle.

197

00:18:31.940 --> 00:18:34.360

PT11: Hmm. Yeah, that will be really hard.

198

00:18:46.900 --> 00:18:48.720

PT11: hmm.

199

00:18:49.350 --> 00:18:55.339

PT11: If they don't see the report. I wouldn't say they would know, so I would. I wouldn't put this.

200

00:18:56.220 --> 00:19:09.679

NM: So what? What? Okay? So you wouldn't include this question in your survey You gave to the parents right? I guess so. Rate it for me, would you? What is it like? Really 0? Not at all appropriate, or is it somewhere on the lower scale or in the middle.

201

00:19:10.030 --> 00:19:11.779

NM: What number would you create it

202

00:19:11.830 --> 00:19:14.059

NM: and tell me why you wouldn't include this one?

203

00:19:15.660 --> 00:19:18.269

PT11: So i'll probably do 2

204

00:19:21.640 --> 00:19:24.359

PT11: just because the

205

00:19:26.010 --> 00:19:31.079

PT11: they can tell that the student is really tired, but just to really

206

00:19:31.400 --> 00:19:34.700

PT11: see or tell that

207

00:19:34.840 --> 00:19:38.000

PT11: the students worked hard for 10 min is

208

00:19:38.190 --> 00:19:43.969

PT11: really difficult to gauge for them. Parents are being sent reports.

209

00:19:44.580 --> 00:19:51.139

PT11: but it's also like daily activities for students. But it doesn't really say that. Oh, this he did

210

00:19:51.280 --> 00:19:56.899

PT11: 10 min on the standard, or but it just say that the student goes on the standard.

211

00:19:57.040 --> 00:20:00.160

PT11: The more detailed reports comes from the therapist.

212

00:20:00.430 --> 00:20:03.629

PT11: so I don't think the parents will really know

213

00:20:03.720 --> 00:20:09.890

PT11: they would know that the students worked like, or did their exercise. But like, if it's really hard or

214

00:20:10.090 --> 00:20:12.969

PT11: just to gauge, the intensity is not.

215

00:20:13.090 --> 00:20:15.320

PT11: I wouldn't say that would be

216

00:20:16.420 --> 00:20:18.470

PT11: easy for parents to gauge.

217

00:20:19.410 --> 00:20:22.049

NM: All right, Number 3. Thank you.

218

00:20:22.260 --> 00:20:30.269

NM: How many did your child exercise so much that he or she breathed hard. How appropriate it is this question

219

00:20:30.500 --> 00:20:35.530

NM: for a child, a family of a child with Cp. Who is not ambulatory?

220

00:20:36.440 --> 00:20:37.220

PT11: Hmm.

221

00:20:37.720 --> 00:20:40.029

PT11: That's more on the higher.

222

00:20:46.000 --> 00:20:48.550

PT11: I'll put that on a 4.

223

00:20:49.660 --> 00:20:53.680

PT11: It's very significant. I think I would relate that to

224

00:20:54.360 --> 00:20:57.360

PT11: like them getting really tired when they get home.

225

00:20:59.090 --> 00:21:05.860

PT11: or like even just reporting from the travel Paris or travel nurse that the student

226

00:21:06.150 --> 00:21:07.289

PT11: fell asleep.

227

00:21:07.730 --> 00:21:12.139

PT11: and the parents would know Students will eat and just go back to sleep right away.

228

00:21:16.150 --> 00:21:19.750

NM: and just to clarify. So if the parent did have the child for the whole week.

229

00:21:19.780 --> 00:21:26.779

NM: let's say in the summer they were off all summer. Do you think a parent would be able to under to identify how many days in the week

230

00:21:27.700 --> 00:21:32.090

NM: that the child exercise so so much that he or she breathe hard

231

00:21:34.070 --> 00:21:38.019

PT11: just in general. Are they able to determine when they're breathing hard

232

00:21:38.180 --> 00:21:38.990

PT11: there? So

233

00:21:39.680 --> 00:21:46.330

NM: okay. So you gave it a 4. So it's it's so. That's appropriate for this population. Okay, great. So

234

00:21:46.350 --> 00:21:51.290

NM: number 4. How many days was your child so physically active that he or she sweated.

235

00:22:04.410 --> 00:22:07.240

PT11: I would go more towards.

236

00:22:14.520 --> 00:22:17.489

PT11: I probably do a 3 on this one.

237

00:22:20.530 --> 00:22:22.030

PT11: because

238

00:22:22.160 --> 00:22:25.109

PT11: at home they see how much movements

239

00:22:25.560 --> 00:22:26.460

PT11: this

240

00:22:26.590 --> 00:22:28.090

PT11: their their child

241

00:22:28.350 --> 00:22:29.350

PT11: does.

242

00:22:29.680 --> 00:22:36.219

PT11: and what is like exerting more for the students, and they know that in school

243

00:22:36.240 --> 00:22:42.940

PT11: they actually do their exercises just to gauge like, what is the intensity really is hard, but

244

00:22:43.780 --> 00:22:46.630

PT11: seeing physically active that they sweat.

245

00:22:47.080 --> 00:22:51.029

PT11: I would say it the 3. It's not easy to to gauge, but

246

00:22:51.510 --> 00:22:53.839

PT11: I think parents will be able to

247

00:22:54.860 --> 00:22:55.810

PT11: to tell.

248

00:22:56.080 --> 00:22:58.250

NM: Okay, in this population. All right. Great

249

00:22:58.540 --> 00:23:04.559

NM: Number 5. How many days is your child exercise a place so hard that his or her muscles burned.

250

00:23:04.840 --> 00:23:09.339

NM: Is that a 0 not related at all, or highly appropriate in this population?

251

00:23:19.420 --> 00:23:22.309

PT11: This this is hard

252

00:23:27.140 --> 00:23:32.359

PT11: because they can't. The student can't actually tell where is bothering them.

253

00:23:33.080 --> 00:23:34.700

PT11: but they will be.

254

00:23:35.430 --> 00:23:37.299

PT11: and most of them doesn't really

255

00:23:37.910 --> 00:23:40.129

PT11: cry or show frustration.

256

00:23:43.030 --> 00:23:47.259

PT11: My only gauge here is like they're just really tired.

257

00:23:47.800 --> 00:23:48.750

PT11: Oh.

258

00:23:56.140 --> 00:23:58.089

PT11: this is really hard to gauge.

259

00:23:58.610 --> 00:24:00.189

PT11: I'm going to put 0

260

00:24:06.020 --> 00:24:07.260

NM: all right. Thank you.

261

00:24:07.470 --> 00:24:12.929

NM: How many days is your child exercise, or place so hard that he or she felt tired.

262

00:24:13.140 --> 00:24:15.380

PT11: So that's a little bit different. That?

263

00:24:15.750 --> 00:24:16.450

PT11: Yeah.

264

00:24:17.560 --> 00:24:19.389

PT11: I would put that to 5.

265

00:24:19.560 --> 00:24:20.870

NM: Okay? Why?

266

00:24:22.570 --> 00:24:27.480

PT11: because this is the most feedback that I get from parents

267

00:24:27.780 --> 00:24:29.339

PT11: really is like just

268

00:24:29.610 --> 00:24:32.729

PT11: this exact word that they are tired.

269

00:24:33.450 --> 00:24:38.900

PT11: so they know that there's lots of physical activities

270

00:24:38.960 --> 00:24:40.210

PT11: in school

271

00:24:40.430 --> 00:24:44.660

PT11: that even though the daily report that

272

00:24:44.810 --> 00:24:48.079

PT11: is sent to them is very

273

00:24:50.630 --> 00:24:52.220

PT11: like generalized.

274

00:24:53.480 --> 00:24:59.870

PT11: But they can. Yeah, the student will just be you up here really tired that after

275

00:25:01.370 --> 00:25:03.080

PT11: after

276

00:25:03.620 --> 00:25:10.230

PT11: all their like activities in school, just reaching for their device and

277

00:25:10.640 --> 00:25:14.879

PT11: for us, it's just doing their walking on the trainer.

278

00:25:15.190 --> 00:25:20.510

PT11: or even just doing truck stability on the on the exercise Call

279

00:25:24.870 --> 00:25:27.310

NM: great, all right, Number 7.

280

00:25:27.800 --> 00:25:31.930

NM: How many days was your child physically active for 10 min or more.

281

00:25:31.990 --> 00:25:33.340

NM: How would you rate that one

282

00:25:34.210 --> 00:25:37.259

NM: 0 not appropriate, or 5 highly appropriate.

283

00:25:42.160 --> 00:25:45.160

PT11: physically active?

284

00:25:45.990 --> 00:25:47.709

PT11: That is highly appropriate.

285

00:25:49.100 --> 00:25:55.450

PT11: because at all times Parents are aware that students are always engaged

286

00:25:56.350 --> 00:25:57.789

PT11: so

287

00:25:59.410 --> 00:26:05.460

PT11: like everything, as I said, just reaching for a communication device, or even dream feeding

288

00:26:07.100 --> 00:26:09.000

PT11: just trying to sell feed

289

00:26:09.710 --> 00:26:11.820

PT11: during

290

00:26:12.040 --> 00:26:14.330

PT11: the diaper change.

291

00:26:14.580 --> 00:26:15.180

PT11: And

292

00:26:15.230 --> 00:26:18.030

PT11: for us this is when they're really

293

00:26:18.240 --> 00:26:26.769

PT11: mostly out of their chair. So if it's like easier for parents to kind of know that. Oh, they're active

294

00:26:27.500 --> 00:26:32.689

PT11: because they know that they have a student has Pt. Session. Then they would know that what

295

00:26:32.820 --> 00:26:37.310

PT11: what we are working on during the session. I think that that's very appropriate.

296

00:26:39.060 --> 00:26:40.599

NM: Okay. So give me a number.

297

00:26:40.800 --> 00:26:42.850

NM: I think I know. Okay.

298

00:26:43.370 --> 00:26:44.199

NM: All right. Great

299

00:26:44.560 --> 00:26:47.370

NM: and last one Number 8.

300

00:26:47.530 --> 00:26:50.790

NM: How many days is your chat run for 10 min or more?

301

00:26:50.830 --> 00:26:55.820

NM: Would you say 0 not related at all, or 5 highly appropriate

302

00:27:00.930 --> 00:27:03.829

PT11: not related at all.

303

00:27:04.790 --> 00:27:05.920

NM: So what number?

304

00:27:06.380 --> 00:27:07.450

PT11: Yeah up

305

00:27:08.600 --> 00:27:09.730

PT11: 0.

306

00:27:11.580 --> 00:27:20.369

PT11: They They know that students are working on their. The their child is working on standing

307

00:27:20.540 --> 00:27:23.689

PT11: or working on walking, taking steps.

308

00:27:24.270 --> 00:27:26.950

PT11: But running will be

309

00:27:27.090 --> 00:27:28.540

PT11: difficult

310

00:27:30.800 --> 00:27:32.629

PT11: to gauge for parents to.

311

00:27:35.400 --> 00:27:44.429

NM: All right. That was my last question. I always ask everybody, do you have any final thoughts about physical activity in this population that you would like to close out with?

312

00:27:48.390 --> 00:27:51.519

PT11: Yeah, no, it's actually more enlightening

313

00:27:51.820 --> 00:27:56.800

PT11: like with the definition that you gave it physical activity, because we, like

314

00:27:57.730 --> 00:28:05.640

PT11: most of the time, Even for myself, I would think that oh, physical activity! We have to be walking. We have to be climbing stairs.

315

00:28:05.680 --> 00:28:10.769

PT11: but as simple as reaching for their device or for the spoon, is also

316

00:28:10.890 --> 00:28:15.339

PT11: physical activity for for all of the students.

317

00:28:19.220 --> 00:28:22.730

NM: Thank you so much. All right. I'm going to stop the recording.
