# Supplementary material for: Validity of PROMIS® Pediatric Physical Activity Parent Proxy Short Form Scale as a Physical Activity Measure for Children with Cerebral Palsy Who Are Non-Ambulatory
Source: Behav Sci (Basel). 2025 Jul 31;15(8):1042. doi: 10.3390/bs15081042 (PMC12382615; doi:10.3390/bs15081042)
Supplement: Supplementary file 1 [file behavsci-15-01042-s001.zip › Transcripts copy/PT transcripts - deidentified/PT12.docx]

WEBVTT

1

00:00:00.000 --> 00:00:00.920

Let's go.

2

00:00:01.980 --> 00:00:19.809

NM: Good evening, Dr. PT12. You guys, it is nice to have you here, so we're going to get started today. We're going to talk about a physical activity in children with Cp. But these children that are non inventory specifically grow smaller. Functional classification scale

3

00:00:19.820 --> 00:00:29.060

NM: levels 4 and 5. So I have a few questions, and then some follow up prompts. So bear with me as I go through my my my questions for for you you have any

4

00:00:29.130 --> 00:00:42.150

NM: a reason for me to re repeat, I'm happy to do so if you need further clarification, i'm happy to do so as well. So first question, how do you define physical activity for children with Cp who are not full time walkers?

5

00:00:46.470 --> 00:00:47.960

PT12: I would say

6

00:00:49.410 --> 00:00:52.649

PT12: any activity that they are actively

7

00:00:52.750 --> 00:00:53.860

PT12: moving

8

00:00:56.460 --> 00:01:03.639

PT12: during a certain amount of time, not just like a spontaneous movement, but like having us a certain amount of time

9

00:01:03.780 --> 00:01:06.850

PT12: that the dedicated to active movement.

10

00:01:07.120 --> 00:01:08.030

PT12: Let's say that

11

00:01:10.060 --> 00:01:11.400

NM: great. Thank you.

12

00:01:12.610 --> 00:01:24.830

NM: First follow up the department of Health defines physical activity as any activity that encompasses energy expended, and activation of skeletal muscles. Does this definition change your mind how you define physical activity.

13

00:01:26.470 --> 00:01:27.490

PT12: That's good.

14

00:01:28.620 --> 00:01:33.689

PT12: It would just change [from NIH definition intro] the the part that I said for a specific amount of time.

15

00:01:33.780 --> 00:01:39.119

PT12: Maybe it's this spontaneous movement with would fall into that classification as well.

16

00:01:43.660 --> 00:01:44.420

PT12: Yeah.

17

00:01:45.900 --> 00:01:51.800

NM: And how do you think physical activity differs from other types of fitness activities?

18

00:01:58.120 --> 00:01:59.480

PT12: I think

19

00:01:59.710 --> 00:02:01.149

PT12: fitness.

20

00:02:04.960 --> 00:02:06.039

PT12: Fitness

21

00:02:07.940 --> 00:02:09.390

PT12: never thought about it.

22

00:02:09.620 --> 00:02:11.460

PT12: But I think

23

00:02:12.270 --> 00:02:14.959

PT12: fitness, maybe something very like

24

00:02:15.260 --> 00:02:17.890

PT12: directed into

25

00:02:19.360 --> 00:02:20.500

PT12: specific

26

00:02:21.470 --> 00:02:22.180

PT12: like

27

00:02:22.290 --> 00:02:24.810

PT12: body goals

28

00:02:25.760 --> 00:02:31.170

PT12: and defining it this way that you just described to me, I think of

29

00:02:31.500 --> 00:02:33.149

PT12: just be able to move

30

00:02:33.200 --> 00:02:38.300

PT12: actively, having, like, as you said, muscle activation, like in general.

31

00:02:39.300 --> 00:02:45.940

PT12: even without having a set call of like getting stronger in this way, or getting

32

00:02:46.040 --> 00:02:50.669

PT12: like a muscle mass in another way. But just being able to

33

00:02:50.940 --> 00:02:52.580

PT12: physically activate it.

34

00:02:55.060 --> 00:03:05.920

NM: So you're you're saying like physical activity versus fitness. Fitness would have the goals. Physical activity would just be the activation more so. Not necessarily for the body goals like you mentioned.

35

00:03:06.020 --> 00:03:09.009

PT12: Yeah, that's how I understood it. Yeah.

36

00:03:09.220 --> 00:03:10.640

NM: thank you.

37

00:03:10.770 --> 00:03:11.890

NM: And

38

00:03:12.240 --> 00:03:18.140

NM: next, when do you witness? Your students participate most in physical activity during the day

39

00:03:19.140 --> 00:03:21.119

PT12: when they're in physical therapy.

40

00:03:24.470 --> 00:03:28.880

NM: And what specifically do you witness them doing? And well, you know what? Nope.

41

00:03:28.930 --> 00:03:31.720

PT12: that that that comes later. Okay.

42

00:03:31.770 --> 00:03:42.660

NM: let's go there. So the second question, how do you measure physical activity, frequency, intensity, time, and type? So if you recall the fit principle

43

00:03:42.910 --> 00:03:58.239

NM: in children with Cp. Who are not full time work work walkers. So let me repeat that I was no one. How do you measure physical activity, frequency, intensity, time and type in children with Cp who are not full time Walkers.

44

00:04:01.860 --> 00:04:03.460

PT12: We would use

45

00:04:04.260 --> 00:04:07.779

PT12: like some standardized test. That's what you mean. Like

46

00:04:07.900 --> 00:04:10.030

PT12: using the

47

00:04:11.900 --> 00:04:20.719

NM: it's. However, you do it. It it doesn't have to be standardized. I mean, I'm, I'm gonna You could say a standardized test, but it's really just kind of what you would think or how you would do this.

48

00:04:21.089 --> 00:04:29.369

NM: you know. How would you, as a therapist measure that measured the amount of activities that frequency, intensity.

49

00:04:29.820 --> 00:04:35.209

NM: time, and type in these children? How would you measure it.

50

00:04:37.670 --> 00:04:39.570

PT12: I think, defining as like an

51

00:04:39.850 --> 00:04:46.780

PT12: active movement it would be very difficult to measure, because throughout the day we're not with this children.

52

00:04:48.760 --> 00:04:52.040

PT12: but just with them for a little bit in their day.

53

00:04:52.190 --> 00:04:58.599

PT12: but measuring at least the part that we are with them in this session.

54

00:05:00.690 --> 00:05:02.130

PT12: We can

55

00:05:02.330 --> 00:05:08.469

PT12: like count repetitions of the activity we we are trying to have them perform.

56

00:05:08.710 --> 00:05:11.349

PT12: We measure

57

00:05:11.570 --> 00:05:13.750

PT12: the intensity by

58

00:05:14.990 --> 00:05:18.550

PT12: decreasing or increasing resistance

59

00:05:18.910 --> 00:05:21.960

PT12: by the amount of assistance, they need

60

00:05:22.160 --> 00:05:28.569

PT12: to perform a certain activity, progressing to like, not needing assistance

61

00:05:30.790 --> 00:05:31.650

PT12: about

62

00:05:32.180 --> 00:05:36.340

PT12: how much they need to use an assistive device or not

63

00:05:36.440 --> 00:05:39.840

PT12: to perform the like a specific activity.

64

00:05:40.130 --> 00:05:41.819

PT12: But what's the other ones?

65

00:05:43.240 --> 00:05:46.519

NM: It was frequency, intensity, time and type

66

00:05:48.800 --> 00:05:49.770

PT12: type.

67

00:05:55.320 --> 00:06:03.640

PT12: I don't know how to measure, type, like maybe divide by body segments. I I don't understand that. Well, I would say just

68

00:06:05.180 --> 00:06:13.649

PT12: usually in in the session, that the type is kind of guided by what we are performing, so

69

00:06:15.470 --> 00:06:20.880

PT12: it's selected, pre selected. But if i'm just analyzing how much they are moving.

70

00:06:21.160 --> 00:06:30.590

PT12: I would separate by body segments like the type of like physical activity, more of like an upper body, lower body.

71

00:06:34.020 --> 00:06:40.610

PT12: Or now I I thought to I could measure as like type of endurance activity more than

72

00:06:40.930 --> 00:06:41.820

PT12: and

73

00:06:43.040 --> 00:06:46.040

PT12: intensity, activity

74

00:06:46.680 --> 00:06:53.470

PT12: that doesn't make sense?

75

00:06:53.510 --> 00:06:56.359

NM: with some of the examples you gave?

76

00:06:56.410 --> 00:07:04.389

NM: Do you? Do they need assistance when you, when they complete some of these types of physical activity like in your sessions, and

77

00:07:04.870 --> 00:07:15.090

NM: and do, and when they need assistance, do they need it for the whole activity When you're doing physical activity in your sessions, which is part of the activity.

78

00:07:15.850 --> 00:07:20.250

PT12: I would say, for the level 5

79

00:07:21.330 --> 00:07:24.070

possibly probably yes.

80

00:07:24.190 --> 00:07:28.799

PT12: for most of it, but not necessarily max assistance.

81

00:07:29.030 --> 00:07:29.890

PT12: but

82

00:07:29.980 --> 00:07:32.840

they will probably need assistance to

83

00:07:33.290 --> 00:07:36.350

PT12: to complete the activity that we are performing.

84

00:07:37.730 --> 00:07:42.709

PT12: either from a like a physical assistance or a device assistance.

85

00:07:47.760 --> 00:07:56.099

NM: And do you think they should participate in more or less physical activity, or these activities that you work on, and why.

86

00:07:57.810 --> 00:08:01.030

PT12: yes, I think they need to participate more

87

00:08:02.830 --> 00:08:06.879

PT12: to not be just performed during it.

88

00:08:07.220 --> 00:08:14.699

PT12: Pt. Session, for example, if they have more opportunities throughout the day to participate, to engage in fiscal activities.

89

00:08:14.770 --> 00:08:16.200

PT12: they can

90

00:08:16.250 --> 00:08:24.360

PT12: be overall more mobile, stronger, prevent deviations to happen.

91

00:08:24.890 --> 00:08:27.760

PT12: have better endurance.

92

00:08:28.270 --> 00:08:32.600

PT12: and maybe be more participative in the community because of

93

00:08:32.750 --> 00:08:36.029

the the consistency, the frequency

94

00:08:36.659 --> 00:08:38.609

PT12: that they will be participating in it.

95

00:08:40.030 --> 00:08:41.340

NM: Great. Thank you.

96

00:08:41.590 --> 00:08:47.289

NM: Number 3. Do you address promoting physical activity during your Pt sessions?

97

00:08:48.360 --> 00:08:49.600

PT12: Yes.

98

00:08:50.470 --> 00:08:54.279

NM: and how do you do this in your Pt. Session. You can give examples.

99

00:08:56.340 --> 00:08:57.440

PT12: I

100

00:08:57.890 --> 00:08:59.610

PT12: I may initially

101

00:08:59.650 --> 00:09:02.480

PT12: guide a child to

102

00:09:02.690 --> 00:09:11.550

PT12: perform an active, a specific activity. And then, towards the end of the session, I try to target them to

103

00:09:11.650 --> 00:09:12.730

PT12: the

104

00:09:12.810 --> 00:09:15.329

PT12: be as independent as they can

105

00:09:15.380 --> 00:09:17.819

PT12: in that activity.

106

00:09:18.010 --> 00:09:25.139

PT12: So using it an initial guidance and repetition of the activities so.

107

00:09:25.440 --> 00:09:26.769

PT12: and they can

108

00:09:27.030 --> 00:09:37.430

PT12: gain better skills, as they repeat, get stronger, get more control, and do, have a better learning overall of the activity. So

109

00:09:37.830 --> 00:09:45.099

NM: can you give me an example of type of activities or things you do to really work on physical activities in your sessions.

110

00:09:45.360 --> 00:09:49.689

PT12: But because we're talking about children, i'm gonna talk about like

111

00:09:51.340 --> 00:09:53.690

PT12: a game. I'm gonna

112

00:09:53.800 --> 00:09:56.230

PT12: use a game like

113

00:09:57.170 --> 00:10:02.400

PT12: like basketball. So an activity where they have to

114

00:10:02.450 --> 00:10:18.200

PT12: band forward to reach for the ball and then lift the ball, hold the ball with both hands, then transition into a standing position, and then reaching until they get to a basket to be able to throw the ball.

115

00:10:18.770 --> 00:10:22.510

PT12: and then going back to sitting, or to whatever position they were.

116

00:10:24.810 --> 00:10:26.200

NM: That's a great example.

117

00:10:26.380 --> 00:10:27.840

NM: and

118

00:10:28.260 --> 00:10:41.600

NM: that what components of physical activity do you, or are you addressing even in that example? Let me give you example what I mean when I say components of physical physical activity? I'm talking about like cardiovascular endurance.

119

00:10:41.700 --> 00:10:46.690

NM: maybe, or muscle activation or energy expenditure. What are the things you feel like you Target.

120

00:10:46.840 --> 00:10:50.830

NM: that's related to the components of physical activity in your sessions.

121

00:10:52.220 --> 00:10:55.090

PT12: I would say.

122

00:10:55.820 --> 00:10:59.319

PT12: proper mobility. Muscle activation

123

00:11:01.370 --> 00:11:03.130

PT12: postural control.

124

00:11:06.250 --> 00:11:19.510

PT12: and in this specific one we may target, depending on how many repetitions. Yes, we may target endurance, not necessarily by measuring cardiovascular activity, but by

125

00:11:19.970 --> 00:11:24.410

PT12: counting the repetitions, maybe, how many they were able to do in the beginning, and how

126

00:11:24.440 --> 00:11:30.059

PT12: how many they're able to do. Maybe in the next session, or in the week or

127

00:11:30.140 --> 00:11:32.109

so. Just

128

00:11:32.830 --> 00:11:35.840

PT12: I personally measure

129

00:11:35.970 --> 00:11:44.949

PT12: more by seeing how my, how much more they are performing than just counting specific cardiovascular activity.

130

00:11:47.990 --> 00:11:48.880

NM: Thank you.

131

00:11:49.000 --> 00:11:49.700

It's great.

132

00:11:52.240 --> 00:12:00.349

NM: moving right along. Do you address promoting physical activity that occurs outside of your physical therapy session?

133

00:12:02.570 --> 00:12:05.779

PT12: So far we

134

00:12:06.020 --> 00:12:13.299

PT12: try to assign the kids to field programs outside of the session which include

135

00:12:13.320 --> 00:12:22.179

PT12: have them peddling around the school. I'm. In the school setting. So, besides what they already do in Pt.

136

00:12:22.190 --> 00:12:34.859

PT12: They have their time outside of the classroom, where they come out to pedal. This is one activity that we we perform outside of it. Another one would be for them to

137

00:12:35.460 --> 00:12:37.180

PT12: walk between

138

00:12:37.490 --> 00:12:45.849

PT12: one side on the other side of the school, either using an assistive device, or with any kind of support that they may need.

139

00:12:47.970 --> 00:12:50.020

PT12: We also

140

00:12:50.620 --> 00:13:01.170

PT12: encourage the children who are more in the classroom, who have, like fewer abilities to at least be taken out of

141

00:13:01.210 --> 00:13:09.440

PT12: their chairs throughout the day, and be placed on the mat to be able to practice some rolling around, and

142

00:13:09.460 --> 00:13:12.969

PT12: or be placed in different positions where they can

143

00:13:13.030 --> 00:13:22.330

PT12: access their educational materials like in a different position. Let's say on their belly’s that they need to lift their head differently. So

144

00:13:22.500 --> 00:13:32.209

PT12: they this all all like different opportunities to be continue to be activating, but not in specifically, in the session.

145

00:13:39.870 --> 00:13:47.180

NM: Great. Thank you. Have you recommended any community programs or events to your students to help increase physical activity.

146

00:13:47.580 --> 00:13:50.880

PT12: Yes, I have recommended

147

00:13:51.550 --> 00:13:53.869

PT12: both hippotherapy

148

00:13:54.280 --> 00:13:55.840

PT12: that

149

00:13:56.140 --> 00:14:12.189

PT12: a lot of people will find more in the suburbs. It's hard to find that, like close in the city, and I have recommended like aquatic therapy in in general, before we had our own centers like starting to be developed.

150

00:14:12.200 --> 00:14:16.659

PT12: We would recommend that children. We go find that outside, too.

151

00:14:19.510 --> 00:14:24.190

NM: And what type of equipment have you recommended to help improve home

152

00:14:24.220 --> 00:14:29.890

NM: and or community engagement and physical activity outside of the clinical setting.

153

00:14:30.560 --> 00:14:40.959

PT12: The first one would be the adaptive tricycle that a lot of the families can have at home, and then just go community outing in the park.

154

00:14:40.990 --> 00:14:44.179

PT12: taking their kids out. And

155

00:14:44.430 --> 00:14:53.219

PT12: the other one also used is like a regular gait trainer. It's supportive for them that some are able to be taken outside.

156

00:14:53.310 --> 00:15:00.200

PT12: like from short distance still, but still, like being able to take them to

157

00:15:00.300 --> 00:15:04.849

PT12: participate more in the community than if they didn't have that

158

00:15:07.780 --> 00:15:13.270

PT12: couple of our families have now the ‘trekxorobotics’ to

159

00:15:13.500 --> 00:15:21.149

PT12: that they they can even go a little further with within the community with the

160

00:15:21.640 --> 00:15:25.840

PT12: adapted to the gait trainer. So also promoting.

161

00:15:26.070 --> 00:15:27.689

PT12: when walking outside

162

00:15:31.140 --> 00:15:42.400

PT12: it's the trexo. Remind me of how do you know how to spell that trek. So T. R. E. X. O, yeah excel robotics. It's

163

00:15:42.620 --> 00:15:50.569

PT12: they partnered with Rifton. Use their day trainer and put a for the robotics

164

00:15:51.250 --> 00:15:54.300

PT12: equipment all around the the pacer

165

00:15:54.610 --> 00:15:57.460

PT12: controlled by a tablet

166

00:15:57.540 --> 00:16:06.050

PT12: that's that are able to count. I forgot to mention that before, but we do use that in therapy sessions to.

167

00:16:06.280 --> 00:16:10.069

PT12: because we have like one.

168

00:16:10.120 --> 00:16:18.819

PT12: You don't have as many. We can't do that as frequent with the kids like every day, because we have to alternate between the users.

169

00:16:18.940 --> 00:16:23.319

PT12: But that's a very good way of measuring

170

00:16:23.580 --> 00:16:34.290

PT12: the number of steps they took, and the amount of assistance that they are in need for it for this step taking, because it shows you.

171

00:16:34.380 --> 00:16:44.889

PT12: if how much force the machine is making to work, how much of they have to fight their tone. So that's a better

172

00:16:45.050 --> 00:16:48.169

PT12: system to actually to

173

00:16:48.330 --> 00:16:50.230

PT12: more

174

00:16:50.570 --> 00:16:53.189

PT12: accurate measurements of That

175

00:16:57.320 --> 00:16:59.850

NM: Hmm. That's good. Okay.

176

00:17:01.300 --> 00:17:09.140

NM: all right. All right. So now we're at the part we'll look at the survey. Let me share my screen. So this is called

177

00:17:11.520 --> 00:17:18.510

PT12: the now it's raining a lot. If you hear there's a lot of rain.

178

00:17:19.180 --> 00:17:22.479

NM: So this is called the App

179

00:17:22.530 --> 00:17:24.389

NM: pro the parent Proxy.

180

00:17:24.450 --> 00:17:29.759

NM: Physical activity, Promise Survey was developed by the National Institute of Health for children that have.

181

00:17:29.850 --> 00:17:32.969

NM: you know, progressive disorders or

182

00:17:33.170 --> 00:17:37.930

NM: children, that we're not typically developing. So there are 8 questions.

183

00:17:38.240 --> 00:17:54.679

NM: and, as you know, there's few measures. They for children with Cp that are non ambulatory in terms of some standardized measures. So i'm looking to get pts, and then eventually parents feedback on these 8 questions. And so what i'm going to ask you for each question is.

184

00:17:54.690 --> 00:18:00.770

NM: How do you think how appropriate is this question as it relates to children that are not ambulatory?

185

00:18:01.050 --> 00:18:07.089

NM: The children that we been talking about today. And so 0 means not related at all.

186

00:18:07.670 --> 00:18:10.800

NM: you know. And then 5 with on the scale from 0 to 5

187

00:18:10.920 --> 00:18:16.279

NM: 5 would be highly appropriate. You think this question will be good to determine physical activity, intensity

188

00:18:16.470 --> 00:18:20.069

NM: for a child at the level she 1 5.

189

00:18:20.530 --> 00:18:22.350

NM: Okay. So the first question.

190

00:18:22.890 --> 00:18:29.690

NM: How many days did your child exercise or place so hard that his or her body got tired? How would you rate that

191

00:18:29.710 --> 00:18:33.029

NM: 0 one scale from 0 to 5 0, not related at all.

192

00:18:33.050 --> 00:18:39.650

NM: or 5. It is highly appropriate to have a parent Answer. Answer. As it relates to physical activity, intensity.

193

00:18:40.390 --> 00:18:42.880

PT12: This is specific, you said, for for

194

00:18:43.230 --> 00:18:50.890

PT12: Gmfc. Levels for yeah. So I would say this place so hard. It's

195

00:18:52.070 --> 00:18:54.949

PT12: it's very subjective, and it's

196

00:18:56.660 --> 00:19:07.599

PT12: it's May, maybe something that the parents don't even see it like, oh, they're not able to do it. So the question does not apply, so I don't think I would.

197

00:19:07.990 --> 00:19:12.480

PT12: I would say one at 2 kind of, you know. Yeah.

198

00:19:16.470 --> 00:19:19.549

NM: All right. That's good. Number

199

00:19:19.840 --> 00:19:20.610

NM: 2.

200

00:19:21.100 --> 00:19:34.119

NM: How many days did your child exercise really hard for 10 min or more, and they would right. How many days in the past 7 days? That's how they answer it. So do you think this question is appropriate on the scale from 0 to 5. How would you rate it?

201

00:19:42.290 --> 00:19:46.629

PT12: I think this one is a little more appropriate. I can see the parents

202

00:19:47.680 --> 00:19:53.200

PT12: like seeing their kids, let's say, participate in a session.

203

00:19:53.990 --> 00:20:01.900

PT12: And then, seeing that they practice walking for 10 min and see them actively engaging, so I would say, for for this one.

204

00:20:01.930 --> 00:20:02.520

Okay.

205

00:20:09.250 --> 00:20:10.780

NM: my number 3.

206

00:20:13.940 --> 00:20:22.819

NM: How many days your child exercise so much that he or she breathed heart? How would you rate this one in this population 4 and 5.

207

00:20:23.400 --> 00:20:25.780

PT12: I don't think that would

208

00:20:26.690 --> 00:20:27.260

PT12: up

209

00:20:28.000 --> 00:20:32.929

PT12: it's. It's rare that we see a child like breathing so heavily.

210

00:20:33.140 --> 00:20:34.890

PT12: After

211

00:20:35.420 --> 00:20:38.169

PT12: participating in the Next we see them

212

00:20:38.300 --> 00:20:40.859

PT12: collapsing. We see them

213

00:20:42.300 --> 00:20:45.080

PT12: like falling asleep after.

214

00:20:45.290 --> 00:20:48.730

PT12: but it's hard that we see them breathing heavily.

215

00:20:48.790 --> 00:20:53.770

PT12: you know, after an activity. So I don't think this one would be

216

00:20:53.800 --> 00:20:54.540

PT12: out.

217

00:20:55.070 --> 00:20:56.990

PT12: I would give it a 2 as well.

218

00:20:57.550 --> 00:20:58.400

NM: Okay.

219

00:20:59.200 --> 00:21:04.330

NM: So a 2 is almost in the middle. Just so, you know. I'm gonna leave it at zoom. But just so you

220

00:21:04.600 --> 00:21:08.989

NM: Alright, because we're going to get there'll still be some more questions for you to see

221

00:21:09.190 --> 00:21:13.799

NM: how many days. Was your child so physically active that he or she sweated.

222

00:21:15.080 --> 00:21:26.089

PT12: Okay, that that I see more possibility because we do see some of them sweating after walking or trying to stay standing.

223

00:21:30.380 --> 00:21:37.990

PT12: even though not necessarily that measures that amount of activity more of like a metabolism thing, too. But

224

00:21:38.120 --> 00:21:39.809

PT12: I think

225

00:21:40.580 --> 00:21:43.730

PT12: let's yeah. Let's go to 4 again for this one

226

00:21:48.140 --> 00:21:50.190

NM: alright? Number 5.

227

00:21:52.870 --> 00:21:58.089

NM: How many days your child exercise or play so hard that his or her muscles burn?

228

00:21:58.440 --> 00:22:02.480

PT12: Yeah, what do you think that one is for a parent?

229

00:22:02.550 --> 00:22:05.960

PT12: I don't think that's appropriate. They cannot answer that.

230

00:22:05.990 --> 00:22:07.250

PT12: in my opinion.

231

00:22:07.660 --> 00:22:09.020

NM: So how would you re that one

232

00:22:10.350 --> 00:22:20.390

NM: one is the is the first one right? 0, 0, 0, 0. And what? Why? Do you think the parents can't measure that one?

233

00:22:24.720 --> 00:22:30.590

PT12: I think it's? It's more of a subjective thing. If it's for me, it's it's

234

00:22:31.140 --> 00:22:33.120

PT12: difficult to to

235

00:22:33.410 --> 00:22:37.400

PT12: make a parent understand what their child would be feeling

236

00:22:38.670 --> 00:22:41.619

PT12: like if the muscle is burning.

237

00:22:41.930 --> 00:22:52.270

PT12: I think it's. It's complicated enough for them to feel in their own bodies, I think, to try to understand if the child's muscle is really burning.

238

00:22:52.900 --> 00:22:59.270

PT12: We can. They can be like tired, but not necessarily burning. It's I don't. I don't.

239

00:23:00.970 --> 00:23:02.349

PT12: They can measure the

240

00:23:02.490 --> 00:23:03.700

PT12: in my opinion.

241

00:23:03.740 --> 00:23:04.480

Yeah.

242

00:23:04.580 --> 00:23:11.000

NM: all right, Number 6. How many days did your child exercise or play so hard there, he that he or she felt tired.

243

00:23:11.340 --> 00:23:13.970

PT12: Okay, that is appropriate. Yeah.

244

00:23:14.040 --> 00:23:18.109

PT12: What number would you give that one? I will give it a 5 then.

245

00:23:18.300 --> 00:23:19.110

NM: And why?

246

00:23:20.970 --> 00:23:28.289

PT12: Because then they know their children enough to know when they're tired when they're not. And so

247

00:23:28.700 --> 00:23:35.849

PT12: after, and they they know they're like tired signs, and so they can.

248

00:23:36.340 --> 00:23:40.029

PT12: They can definitely say, like, oh! After doing this.

249

00:23:40.280 --> 00:23:52.619

PT12: I see that they are very tired because they are performing, or they are falling asleep, or they are not like holding their heads up, or they are not participating. After you know they they know their children for that

250

00:23:54.330 --> 00:23:55.160

great

251

00:23:55.730 --> 00:24:02.340

NM: 7. How many days has your sorry, how many days was your child physically active? For 10 min or more.

252

00:24:02.380 --> 00:24:05.489

NM: However, we 0 not at all related, or 5

253

00:24:05.650 --> 00:24:06.800

NM: highly appropriate.

254

00:24:07.150 --> 00:24:08.540

NM: or somewhere between.

255

00:24:09.150 --> 00:24:13.450

PT12: I think this one I will put in the middle, because

256

00:24:13.540 --> 00:24:15.390

PT12: I think

257

00:24:15.760 --> 00:24:17.739

PT12: you, if you are

258

00:24:19.000 --> 00:24:21.080

PT12: for 2 reasons, one

259

00:24:21.180 --> 00:24:25.420

PT12: depending on the activity, if it's like an activity that is

260

00:24:26.850 --> 00:24:34.279

PT12: more, very like lower functioning activity. And you still see the child engaging. You can still measure that. But at the same time

261

00:24:34.390 --> 00:24:38.749

PT12: a lot of the kids, Gmf. Cs 4 and 5

262

00:24:39.450 --> 00:24:51.859

PT12: may not be able to assist in 10 min of activity, they may need a lot of breaks. So that's why I would put in the middle. Because, even though there's a component that

263

00:24:52.040 --> 00:24:55.790

PT12: is measurable. I feel like realistically

264

00:24:55.840 --> 00:24:59.439

PT12: depending on. If you put that for most activities.

265

00:25:00.260 --> 00:25:02.949

PT12: they really may not be able to reach 10 min.

266

00:25:05.320 --> 00:25:07.330

NM: Okay, so give me a good a number for that.

267

00:25:09.450 --> 00:25:10.799

PT12: So be a 3,

268

00:25:10.910 --> 00:25:11.890

NM: 3. Okay.

269

00:25:13.130 --> 00:25:21.150

NM: And I I just want to make sure I I understood you so because, depending on the activity, low functioning children, they can be measured.

270

00:25:21.480 --> 00:25:22.850

However.

271

00:25:23.890 --> 00:25:31.249

NM: we we can measure. I guess, I, that you were saying you can measure a little bit about. You know how far how active they are. However.

272

00:25:31.420 --> 00:25:48.339

PT12: you're saying those that are more involved. They can't. They typically can't go for a whole 10 min. That's that. What I is that what you said? I don't make sure. I understand that. Yeah, yeah, I said it that even though, like, if you are, let's say in a Pt. Session, and then they're working on. I'm gonna

273

00:25:48.350 --> 00:25:51.779

PT12: given an an example of

274

00:25:52.390 --> 00:25:59.160

PT12: like rolling, so they they may be able to be practicing rolling for 10 min.

275

00:25:59.230 --> 00:26:17.799

PT12: But if we put that for most activities, they may not be able to just sustain that, like, be doing that for 10 min, let's say just sometimes it's holding their heads up, and then they will need to like, Get a lot of breaks in between to be able to reach those 10 min. So

276

00:26:17.810 --> 00:26:19.930

PT12: I think that maybe

277

00:26:20.400 --> 00:26:23.039

PT12: too much depending on the activity.

278

00:26:23.160 --> 00:26:24.530

NM: Got it? Okay?

279

00:26:24.640 --> 00:26:25.580

NM: Hello.

280

00:26:32.270 --> 00:26:40.520

NM: Understood that perfect? Okay. Number 8 last one. How many days did your child run for 10 min or more? 0

281

00:26:40.610 --> 00:26:46.909

NM: not related at all? 5 highly appropriate! How would you rate this one

282

00:26:47.200 --> 00:26:50.850

PT12: for this population, I would give it a 0

283

00:26:51.280 --> 00:26:57.300

PT12: 4 and 5. Yes, no running gonna offend the parents kind of.

284

00:26:58.420 --> 00:27:00.430

PT12: in my opinion. Yeah.

285

00:27:07.530 --> 00:27:12.299

NM: right? And now that we're at the end. I always ask everybody for any final

286

00:27:12.370 --> 00:27:17.969

NM: words or comments about physical activity in this population that you'd like to share.

287

00:27:20.580 --> 00:27:27.099

PT12: I do believe it's so important to establish this continuity of

288

00:27:27.400 --> 00:27:28.919

PT12: giving them

289

00:27:29.080 --> 00:27:32.289

PT12: accessible opportunities

290

00:27:32.390 --> 00:27:35.549

PT12: for physical activities throughout their days.

291

00:27:36.240 --> 00:27:41.929

PT12: Of course they need the the accessibility. They need, the supervision. They need

292

00:27:42.260 --> 00:27:46.069

PT12: the scheduling of all the

293

00:27:47.230 --> 00:27:49.869

PT12: and there's so many things in their days

294

00:27:50.210 --> 00:27:53.370

PT12: like between feeding and

295

00:27:55.940 --> 00:28:02.139

PT12: proper naps and academics, and so many things that are important that take time to

296

00:28:02.430 --> 00:28:06.250

PT12: that are understandably hard to

297

00:28:06.410 --> 00:28:08.919

PT12: fit in those activities. But

298

00:28:09.150 --> 00:28:11.360

PT12: I do believe, even though

299

00:28:12.020 --> 00:28:15.930

PT12: that may not be as frequent as we Pts. Wish

300

00:28:16.060 --> 00:28:19.289

PT12: that they can be more frequent than

301

00:28:19.360 --> 00:28:22.789

PT12: what we see overall this kids performing.

302

00:28:23.100 --> 00:28:24.680

PT12: So I

303

00:28:24.930 --> 00:28:28.330

PT12: would strongly like defend the

304

00:28:28.790 --> 00:28:38.169

PT12: the promotion of more frequent physical activities for it, for this kids, mostly at this population of Gmf. Cs. 4 and 5.

305

00:28:41.250 --> 00:28:46.129

NM: Thank you so much. Well, said i'm going to stop our recording.
