# Supplementary material for: Validity of PROMIS® Pediatric Physical Activity Parent Proxy Short Form Scale as a Physical Activity Measure for Children with Cerebral Palsy Who Are Non-Ambulatory
Source: Behav Sci (Basel). 2025 Jul 31;15(8):1042. doi: 10.3390/bs15081042 (PMC12382615; doi:10.3390/bs15081042)
Supplement: Supplementary file 1 [file behavsci-15-01042-s001.zip › Transcripts copy/PT transcripts - deidentified/PT2.docx]

WEBVTT

1

00:00:01.650 --> 00:00:16.249

NM: Alright, Good morning. Thank you so much for joining us today. Today we're going to talk about physical activity, and I have a few questions for you, so just answer them as as completely and comfortably as you feel. I do have some follow ups; but

2

00:00:16.260 --> 00:00:29.699

NM: as you continue, we may not need to go more into detail. I do at 1 point want to share a survey with you, and then i'm going to ask you some questions about that, and that'll be the second half of this interview. So the first question I have for you

3

00:00:29.920 --> 00:00:33.390

NM: is, how do you define physical activity

4

00:00:33.470 --> 00:00:40.399

NM: for children with who are not full time walkers specifically gmfcs levels 4 and 5.

5

00:00:44.830 --> 00:00:47.149

PT2: I think I have to think about that.

6

00:00:47.300 --> 00:00:50.419

PT2: because I think it's very hard for them

7

00:00:50.500 --> 00:01:03.699

PT2: to move in general, and walking is so laborious for them. But physical activity could be anything from actually even

8

00:01:03.920 --> 00:01:17.370

PT2: just getting from one place to the next, whether they're using a walker, whether they're using a bicycle, whether they're crawling on their hands and knees. That's what physical activity that's

9

00:01:17.380 --> 00:01:28.570

PT2: That's enough of physical activity. What I would like for them to be physical with is active in like, maybe playing a game of catch, or, you know.

10

00:01:28.650 --> 00:01:30.200

PT2: even just.

11

00:01:30.710 --> 00:01:36.919

PT2: you know, doing something very simple, as just maybe moving their arms and dancing.

12

00:01:39.550 --> 00:01:42.490

NM: Thank you for that. How do you think

13

00:01:42.550 --> 00:01:48.099

NM: physical activity differs from other types of fitness activities

14

00:01:49.100 --> 00:02:04.440

PT2: for that level of GM: Yes, I think movement in general for those clients with with that level is very difficult and very laborious, even, you know, just even stuff that.

15

00:02:04.450 --> 00:02:09.899

PT2: and just daily tasks are very difficult. So I think activities for them.

16

00:02:10.199 --> 00:02:12.070

PT2: even just sitting up

17

00:02:12.240 --> 00:02:14.950

PT2: and trying to even read a book

18

00:02:15.280 --> 00:02:16.990

PT2: is difficult for them.

19

00:02:19.440 --> 00:02:26.200

NM: Thank you. And when do you witness your students that you have that? Or that you treated at this level?

20

00:02:26.830 --> 00:02:35.189

NM: When do you witness them? Participate most in physical activity. Let's say, during the school day or during your your work hours.

21

00:02:36.130 --> 00:02:53.519

PT2: because Blythedale Children's Hospital is connected to a school. We see our kids go to the school during the day and our kids um I see some of them being active when they go out in the playground in the back

22

00:02:53.530 --> 00:03:13.279

PT2: the one to ones will actually take them out of their chair and let them, you know, move around on the playground a little bit, and then Some of the other kids, unfortunately, are just active when it is like, maybe like an adjunct program where a PT aide is putting them on a bicycle.

23

00:03:18.200 --> 00:03:19.680

NM: Excellent. Thank you.

24

00:03:19.740 --> 00:03:38.070

NM: Okay. So now I want to move a little bit into the next question. How do you measure physical activity, frequency, intensity, time and type? That's the fitt principle just to give you where I'm going with this in children with Cp Who are not full time walkers.

25

00:03:42.050 --> 00:03:50.070

NM: I can say, r

PT2 repeat the question again. I think I have to think about it, you know, and no problem. Take as long as you need.

NM

How do you measure physical activity, frequency.

26

00:03:50.390 --> 00:03:51.720

NM: intensity.

27

00:03:51.930 --> 00:03:53.710

NM: time and type.

28

00:03:54.430 --> 00:03:58.460

NM: and children with Cp. Who are not full time walkers.

29

00:04:00.260 --> 00:04:05.769

PT2: I don't know that I even have a measure for that. I mean, I think, some part of me

30

00:04:05.790 --> 00:04:19.669

PT2: is actually in the process of actually looking for a measure of some of how our kids spend their free time as well. And what makes them, you know, enjoy what they enjoy to do

31

00:04:22.760 --> 00:04:25.590

NM: Do they need assistance to complete

32

00:04:26.290 --> 00:04:29.919

physical activity. And so and then

33

00:04:29.950 --> 00:04:47.360

NM: you gave some examples before. So I do have some. If you want some additional, so do they need a assistance to do some of the things you mentioned, For example, the the the bike riding or the moving from one place to the we.

PT2: Unfortunately, yes, they need assistance to get on the bike.

34

00:04:47.370 --> 00:05:01.889

PT2: If they want to move on their own. Someone may have to help them get out of their wheelchair, so they can get on the the floor and crawl, or they may need their wheelchair or their power wheelchair to get them from Point a to Point B,

35

00:05:02.760 --> 00:05:19.089

PT2: or they may use different forms of assistant technology, as well as whether they're going to use a computer, or they're going to use an eye gaze system, so that they can read a book, or, you know, participate in maybe a video game.

36

00:05:22.100 --> 00:05:23.600

NM: And

37

00:05:23.890 --> 00:05:31.769

NM: will they just need assistance to say you mentioned to get in and out of a chair, went on a bike Or do they need it sometimes for the whole task.

38

00:05:34.220 --> 00:05:42.140

PT2: I'm thinking about the kids that i'm treating, and they probably just need it to get in and out and on and off.

39

00:05:48.290 --> 00:05:50.140

NM: And why would you say that

40

00:05:51.350 --> 00:06:07.059

PT2: so once they get in and on, once they once once she gets out of her wheelchair, she is unable to ambulate, but she can crawl around on the floor to get where she needs to go. Once

41

00:06:07.070 --> 00:06:14.970

PT2: he's put on the bicycle, he can ride the bicycle and steer the bicycle, but he can't get on by himself because his balance is so poor.

42

00:06:19.930 --> 00:06:22.379

NM: Do you think they should participate

43

00:06:22.600 --> 00:06:27.040

NM: more or less? And each of these activities you mentioned, and why?

44

00:06:28.530 --> 00:06:37.220

PT2: Oh, I think they should participate more in all these activities. Because that's what typical children are doing. They need to be more like typical children.

45

00:06:37.630 --> 00:06:48.779

PT2: So yes, they should be be participating in more. And if there's any way that I think we can adapt something to make it so that they can participate more. We should try to do that as well.

46

00:06:50.490 --> 00:06:51.660

NM: Thank you.

47

00:06:52.080 --> 00:06:53.890

NM: Okay. Next question.

48

00:06:54.290 --> 00:07:00.200

NM: Do you address promoting physical activity during your actual physical therapy sessions.

49

00:07:01.550 --> 00:07:12.950

PT2: Yes, yes, because there are sometimes when you know I will put them additionally on the bike, or we will do something like um

50

00:07:12.960 --> 00:07:25.580

PT2: I'll be assisting walking, and we'll walk and go get a ball, and then try to do some hoops through the ball, and then we have to kind of chase the ball a little bit, so we can get it back in to the the the basketball hoop

51

00:07:26.250 --> 00:07:27.060

NM: right.

52

00:07:27.190 --> 00:07:33.950

NM: And so you gave me some examples which is great. What components of physical activity do you feel like you are addressing?

53

00:07:34.400 --> 00:07:40.940

NM: So, for example, cardiovascular endurance, muscle, activation, energy, expenditure, what things do you feel like you

54

00:07:41.240 --> 00:07:45.030

NM: address when you're doing physical activity in your pt session.

55

00:07:45.090 --> 00:07:49.230

PT2: I think we're ,what was the second one again?

56

00:07:49.610 --> 00:07:59.470

NM: I said cardiovascular endurance, muscle, activation, energy, expenditure

PT2: Yes both of those, Cardiovascular and muscle activation are what I am addressing the most.

57

00:08:00.650 --> 00:08:06.169

NM: And there could be more things. I just gave you some example, so you don't have to be limited to my My list

58

00:08:06.600 --> 00:08:12.239

NM: Oh, you want me to think about a few more. Some of the things

59

00:08:12.380 --> 00:08:32.240

PT2: i'm thinking about like postal control on, probably tapping into some of that as well. And when they their physical and they're moving, it's just a general, you know a psychomotor i'm tapping into that as well, so that they're feeling like their sibling. You know that they can participate with their sibling

60

00:08:34.510 --> 00:08:35.380

NM: Excellent.

61

00:08:36.140 --> 00:08:37.560

NM: And

62

00:08:39.190 --> 00:08:40.390

NM: so you didn't

63

00:08:41.390 --> 00:08:45.730

NM: Nope, you. Okay, that's great. No, thank you. And

64

00:08:46.300 --> 00:08:54.549

NM: next question do you address promoting physical activity that occurs outside of Pt. Sessions? Now we talked about inside your session.

65

00:08:54.620 --> 00:08:59.280

NM: Do you address promoting physical activity that occurs outside of Pt. Sessions.

66

00:08:59.810 --> 00:09:05.010

PT2: Yes, I do there a couple of parents, Unfortunately, with

67

00:09:05.100 --> 00:09:17.670

PT2: this gmfc. Scale, these children don't have access to like, I said a bicycle, so I've helped Some parents

68

00:09:17.680 --> 00:09:31.639

PT2: be able to get an adaptive tricycle so they can use that so they can go biking with their sibling. Some parents have also asked about different things in the Home for some of my younger kids

69

00:09:31.650 --> 00:09:52.690

PT2: they were looking at, you know. There's I. I forgot what they call them is this: this is snug system or something where you put everything together, and you crawl up and down, and you're moving around. And they they asked about some of that, and we've discussed it. It's been new to me, but I thought it was a good idea, for you know you can, you know.

70

00:09:52.700 --> 00:09:58.470

PT2: promote some of that crawling in a safe pattern with all these foam pads. So yes.

71

00:10:00.190 --> 00:10:01.169

that's great.

72

00:10:01.640 --> 00:10:03.899

NM: And have you recommended

73

00:10:04.060 --> 00:10:08.319

NM: any community programs or events to your students to help increase.

74

00:10:08.350 --> 00:10:09.740

PT2: This is where everybody

75

00:10:10.490 --> 00:10:25.770

PT2: I don't think I've done that recently. But, prior I've been. I've done some of that where I've gone through green chimeny’s, or I forgot the other one for horseback riding therapeutic horseback riding.

76

00:10:25.780 --> 00:10:35.570

PT2: because I think that's also very important, and that's a nice physical activity out because it can really get into the postal control system.

77

00:10:35.670 --> 00:10:38.890

PT2: I've also promoted

78

00:10:38.980 --> 00:10:52.360

PT2: There's the one in the city. I forgot what it's called Asphalt green. It's used to be in New York City, and it would do a lot of basketball, wheelchair, basketball, and it would do some swimming as well.

79

00:10:54.670 --> 00:10:57.250

PT2: And then Usually I tell parents

80

00:10:57.420 --> 00:11:07.070

PT2: if they can go to their local Y to see if there's something for them, and then, if not, I refer them for swimming to angel fish.

81

00:11:11.980 --> 00:11:24.580

NM: and you answered this question. But i'll say i'll ask it just to kind of give you a chance. You want to add any more. What type of equipment have you recommended to help improve home or community engagement and PA outside of clinical settings?

82

00:11:25.200 --> 00:11:31.779

PT2: Oh, for home, like you, said I, we have done bicycles. They've there's all kinds of adapted

83

00:11:33.070 --> 00:11:46.870

PT2: like. We said to these little adapted foam things where you can make them into steps, and you can make ramps, and you can crawl up and you can crawl down, and it's a safe environment anytime, even if a parent see something.

84

00:11:46.880 --> 00:12:01.689

PT2: And they tell me. You know, PT2, I was thinking of getting this. What do you think we can do? Can we make a change to it? I'm glad to have them, you know. Bring it in and discuss if we can really make it work. So again we can promote participation.

85

00:12:02.280 --> 00:12:03.080

NM: Great.

86

00:12:06.160 --> 00:12:10.829

NM: awesome, Thank you. And so now I'm going to go ahead and pull up

----------------------------------------------------------------------------------------------------------------------------

87

00:12:11.400 --> 00:12:14.980

NM: the promise survey. Are you familiar with this survey?

88

00:12:15.620 --> 00:12:19.180

PT2: No, I just somebody just mentioned it.

89

00:12:19.280 --> 00:12:30.350

PT2: and I've been Look! I looked into it, but I've never really got like I didn't. I didn't have time. I pulled it up, and then I get directed, and when it wasn't able to to look at it truly.

90

00:12:30.790 --> 00:12:38.290

NM: Okay. So i'll pull it up for you, and so you can just add it on the screen for a second, and then what i'll do is

91

00:12:39.130 --> 00:12:44.730

NM: ask you a few questions. The first thing I want to ask you is, how relevant

92

00:12:45.250 --> 00:12:48.540

NM: Would you think this question is to children

93

00:12:48.930 --> 00:12:58.650

NM: at Gmf Cs level 4 and 5, so that would be, and i'll give you a scale so like 0 I mean one not relevant at all. 5 highly relevant

94

00:12:58.750 --> 00:13:05.990

NM: right? So that will be the first thing to talk about, and then and i'm going to ask you why, you know, if you if you think is relevant.

95

00:13:06.040 --> 00:13:18.340

NM: and you'll see great. So this is a just to give you a little bit about the tool. So this was created by Nih, and ideally it's it's a parent proxy form, so it's not for the child to answer it for a caregiver

96

00:13:18.430 --> 00:13:31.200

NM: for a professional that works with the child to answer how active that chat had been in the past 7 days prior to doing this. So that's the first thing. And so it's 8 questions nice and quick.

97

00:13:31.660 --> 00:13:33.340

NM: and

98

00:13:33.670 --> 00:13:41.909

NM: and these are the quite and trying to make it so I can. You can see all 8 questions there you go. So take a moment to look at that, and then i'll go ahead with the next case to see it.

99

00:13:42.390 --> 00:13:44.850

PT2: Okay, Great? Okay, yeah.

100

00:13:45.380 --> 00:13:46.240

NM: Okay.

101

00:13:46.610 --> 00:13:54.629

NM: So i'm gonna do is, ask you a few questions about. So the first question again, I'm gonna ask you to tell me

102

00:13:55.750 --> 00:13:57.390

NM: on a scale from one.

103

00:13:57.900 --> 00:13:59.150

NM: 2, 5,

104

00:13:59.490 --> 00:14:00.900

NM: how appropriate?

105

00:14:00.920 --> 00:14:07.850

NM: And i'm sorry 0, we'll start a 0. So by not at all 0. Okay. So not if it is related or not.

106

00:14:07.870 --> 00:14:09.469

NM: And then

107

00:14:10.640 --> 00:14:16.279

NM: you gonna tell me how appropriate or not it is. And then I'm gonna ask you Why? Okay? And so the first question is.

108

00:14:16.340 --> 00:14:32.559

NM: How many days did your child exercise or play so hard that his or her body got tired. So with that first question, would you say it's 0 not relevant all or 5 highly appropriate, and it could be anywhere along that spectrum.

109

00:14:34.130 --> 00:14:48.870

PT2: I would probably say it's within to the 4 to 5 range, and i'm going to tell you why. Because if you're doing extra things with your child, and then you're coming into therapy, and they're having difficulty.

110

00:14:48.960 --> 00:14:51.769

PT2: you know, doing stuff like they're too tired

111

00:14:52.320 --> 00:14:54.340

PT2: like I'm, i'm worried about over.

112

00:14:54.370 --> 00:15:14.190

PT2: I'm like over exhausting the child. Yes, I do believe that you should do it, and maybe you should do it in like a moderation, where, if you're doing it, you know 2 or 3 days, and you know, then they're getting therapy during the week. Then they're not so fatigued that they can participate in everything.

113

00:15:14.410 --> 00:15:28.450

PT2: And also, if they're working so hard that they get so tired, can they really participate like i'm thinking the I think what's got me. Is that the hard that they got so tired? I think that's got me in there like

114

00:15:28.790 --> 00:15:29.979

PT2: i'm worried about

115

00:15:30.240 --> 00:15:41.770

PT2: knowing that level. Is it? Is it going to be too much for them, because I want them to participate and enjoy it. But I don't want them to be exhausted that they're like. Oh.

116

00:15:42.410 --> 00:15:50.499

NM: do you know what i'm trying to say? Yes, I do. I absolutely so. Would you say? 4 or 5 I get I i'm a child

117

00:15:51.070 --> 00:15:56.040

PT2: all right, I'll I'll narrow it down to 4. Okay. Alright, so yeah, I I hear you good to know.

118

00:15:56.120 --> 00:15:59.449

NM: Okay, so. And the next question.

119

00:15:59.720 --> 00:16:09.410

NM: How many days do you think this is appropriate? How many days did your chop exercise really hard for 10 min or more? How appropriate would you say? That is

120

00:16:10.230 --> 00:16:18.390

PT2: again? I still think that's a for, because for me to know and to gauge what i'm doing in therapy.

121

00:16:18.770 --> 00:16:21.589

PT2: I need to be able to know that.

122

00:16:21.710 --> 00:16:27.780

PT2: and I also be able to know that if i'm making a recommendation of what you're doing.

123

00:16:28.020 --> 00:16:47.650

PT2: I need to know how many days you're doing it, and and how hard it is. And if you know, is is it 10 min that they're doing it? And 10 min, you know, 3 times a week? Okay, that that could be good but 10 min every day. I don't know. That may be too fatiguing again for that level of

124

00:16:47.900 --> 00:16:49.400

PT2: of GM.

125

00:16:50.540 --> 00:16:52.870

NM: And just a question. So. What? How much

126

00:16:54.120 --> 00:16:58.060

NM: would you want a child at this level to be exercising? Let's say.

127

00:16:58.120 --> 00:16:59.460

NM: for 10 min.

128

00:17:00.110 --> 00:17:17.820

PT2: How much would I want them to be exercising for 10 min in if they're receiving daily therapy. Because i'm thinking about the program that's up life sale. So now, if you're a blight out, if you're coming, and you're getting a half hour of me.

129

00:17:17.829 --> 00:17:23.490

PT2: and i'm putting you on a standard, and i'm putting you on a biking program, and maybe i'm also putting you in pool

130

00:17:23.829 --> 00:17:30.979

PT2: by the time you get home. And now you've been in school all day by the time you get home i'm feeling you're going to be fatigued

131

00:17:31.130 --> 00:17:33.509

PT2: like you're going to be exhausted from your day.

132

00:17:34.080 --> 00:17:46.110

PT2: So i'm thinking if you're on that kind of tight schedule, i'm thinking maybe 3 times a week should be a Max or 2 2 times a week, you know, just to supplement the therapy.

133

00:17:47.180 --> 00:18:00.499

PT2: and if you were getting less therapy like, say you were getting 33 times a week, and you weren't getting so many of those adjunct programs. Then I could say, oh, you know daily would be good, or or even 5 to 5 to 7 days would be good.

134

00:18:01.130 --> 00:18:07.329

NM: Got it? And just to be clear, this can include therapy. So if a parent is answering this about their child.

135

00:18:07.530 --> 00:18:24.440

NM: I didn't know that it included therapy. Okay, so just so, you clear that's why why that's why I asked the follow up, because I want to make sure. You know, this is like a parent making a a a a judgment about whatever their child is in. Okay, okay, all right. So then, that makes sense. Yes, Daily is definitely. You definitely need that daily.

136

00:18:24.460 --> 00:18:26.520

NM: So you do want. Yes, that would be good for the

137

00:18:26.690 --> 00:18:28.439

PT2: in terms of that answer to no far away.

138

00:18:28.860 --> 00:18:29.570

PT2: Okay.

139

00:18:35.070 --> 00:18:36.759

NM: all right. Third question.

140

00:18:36.950 --> 00:18:43.329

NM: How many days did your child exercise so much that he or she breathed hard. How relevant

141

00:18:43.980 --> 00:18:48.230

NM: is that question to assessing physical activity in our in this problem

142

00:18:48.320 --> 00:18:48.900

that I

143

00:18:50.520 --> 00:18:51.770

PT2: Oh, well.

144

00:18:51.850 --> 00:19:04.100

PT2: again, some of our kids also have respiratory issues. So I have to pay attention to that as well. So I want to know if they're breathing hard, and if they're breathing hard

145

00:19:04.350 --> 00:19:06.290

PT2: I want to make sure that

146

00:19:06.570 --> 00:19:22.160

PT2: i'm not one over taxing loans again, and i'm promoting, you know, therapy and a promoting exercise at a therapeutic level, whereas i'm just making them breathe hard in their their level of exercise, is not

147

00:19:22.170 --> 00:19:29.030

PT2: like. I don't know that I can gauge exercise by how hard you breathe, especially if you have an underlying respiratory condition.

148

00:19:29.460 --> 00:19:38.859

NM: right? This is specific to physical activity. So I want to be clear. So if they do ask about exercise. But they have made this a physical activity survey.

149

00:19:38.920 --> 00:19:39.540

PT2: Yeah.

150

00:19:39.630 --> 00:19:42.829

NM: which means it doesn't have the same parameters

151

00:19:43.370 --> 00:19:45.059

NM: as fitness.

152

00:19:45.270 --> 00:19:49.489

NM: You know what i'm saying like so it. And then it's supposed to be for children that are not

153

00:19:49.680 --> 00:19:52.249

NM: typically developing. So i'll just pull that in there.

154

00:19:52.750 --> 00:19:57.270

NM: Okay, so. But this number Would you do this? I would give this it

155

00:19:57.390 --> 00:20:01.640

PT2: well again. It's I i'm going with 4

156

00:20:01.680 --> 00:20:04.519

PT2: of going before I think it's important to know.

157

00:20:05.250 --> 00:20:12.309

NM: because you know I don't, and I don't know you're ongoing respiratory issues, right? If there's with this population

158

00:20:12.340 --> 00:20:15.609

NM: really okay? Number 4

159

00:20:15.720 --> 00:20:16.650

NM: is

160

00:20:16.900 --> 00:20:27.420

NM: how appropriate? Do you think this question is measuring physical activity in 4 and 5. How many days was your child so physically active that he or she sweated.

161

00:20:28.210 --> 00:20:39.009

PT2: would you say? Not relevant at all, or 5 highly? I would put it down on the low scale on the one or 2 again. All right, so I would put it down on the to the one I know I can't.

162

00:20:39.070 --> 00:20:44.479

PT2: I'm having trouble with that. I I would put it at the 2, because some of the again have

163

00:20:44.570 --> 00:20:52.580

PT2: have. Yes, because some of them have difficulties with that auto regulation, and some of them don't really sweat so

164

00:20:52.620 --> 00:20:54.829

PT2: I could be working you

165

00:20:54.870 --> 00:20:56.470

PT2: really hard.

166

00:20:56.570 --> 00:20:59.389

PT2: and you're not going to sweat it all on. So

167

00:21:02.530 --> 00:21:10.729

NM: I know I'm always giving you the outlier. Sorry. No, I love it, because this is what I think. This is what we see, you know. And

168

00:21:11.020 --> 00:21:15.650

NM: yeah, so i'll give you my so 5.

169

00:21:15.930 --> 00:21:17.830

How relevant is this question?

170

00:21:17.880 --> 00:21:19.769

NM: Okay. So 0 Not at all.

171

00:21:20.140 --> 00:21:22.180

NM: 5 highly appropriate.

172

00:21:22.300 --> 00:21:28.880

NM: How many days did your child exercise or play so hard that his or her muscles burned.

173

00:21:29.340 --> 00:21:40.050

PT2: That's a 5. I want to know that that's very important, because i'm worried that I overworked you, and again I don't want to over fatigue you. There's a good

174

00:21:40.180 --> 00:21:49.550

PT2: there's you have to find that balance between working you with, not for teaching you, and as therapist, I think sometimes

175

00:21:49.670 --> 00:22:07.549

PT2: we push the limit and we see something happening, and we're going to push it, and we push it, and we push it, and I have some very cognitive and commutative kids, and they tell me, oh, PT2, my legs were very shake. You and I got homeless night. They were really tired, or

176

00:22:07.560 --> 00:22:13.460

PT2: you know I can. You stretch me a little more today because i'm, i'm feeling it from yesterday. So

177

00:22:13.540 --> 00:22:16.060

PT2: I think it's definitely a 5.

178

00:22:16.270 --> 00:22:20.769

NM: Yeah, because it's appropriate. Number 6.

179

00:22:21.020 --> 00:22:30.959

NM: How appropriate to physical activity assessment is this one? How many days did your child exercise or play so hard that he or she felt tired

180

00:22:36.870 --> 00:22:43.779

PT2: again. I put that almost that I I let's go for, because because I need to know

181

00:22:43.880 --> 00:22:57.010

PT2: that what? Especially if you're coming to therapy. I need to know that what I'm doing is not making you so fatigued that you can't participate in other things in your life like education.

182

00:22:57.020 --> 00:23:13.850

PT2: and even pushing your wheelchair to go outside and play with your friends, or get on that bicycle to ride with your friends something that you enjoy to do so. I think that's important to know. So you can make that moderation. So again, you could promote participation.

183

00:23:14.800 --> 00:23:15.970

That's good.

184

00:23:17.720 --> 00:23:19.400

NM: all right. Number 7.

185

00:23:19.460 --> 00:23:25.049

NM: How many days. Was your child physically active for 10 min or more? How appropriate

186

00:23:25.090 --> 00:23:27.420

NM: would you write that 0? Not at all.

187

00:23:27.830 --> 00:23:31.009

NM: or a 5 highly appropriate.

188

00:23:31.170 --> 00:23:34.479

NM: as it relates to physical activity intensity.

189

00:23:35.430 --> 00:23:44.259

PT2: I again I think that's a 5, because again I I like to know what you're doing like. I may see you 5 days a week, and again I have you

190

00:23:44.270 --> 00:24:01.530

PT2: on a a standing program, a biking program, a walking program, your pool, and then your parents take you on the weekend, and they have, you, you know, walking in the park, or riding your bike in the park, and then you come in, and you're totally exhausted. So we we need to know how to balance your activity.

191

00:24:02.960 --> 00:24:07.649

NM: And this is specific for measuring physical activity intensity. So this gives you a measure.

192

00:24:08.180 --> 00:24:16.900

PT2: So I would want to know. Yes, how many days that you did this, because maybe there's going to be a day when i'm going to say, you know, maybe today should be a You'll die.

193

00:24:17.000 --> 00:24:17.830

PT2: you know.

194

00:24:18.330 --> 00:24:21.770

PT2: You know. Maybe today we should go to the library and meet a book day.

195

00:24:26.980 --> 00:24:31.099

NM: and the last question is, how appropriate

196

00:24:31.160 --> 00:24:35.349

NM: would this be for a child at Gmfs level? 4, 5,

197

00:24:35.370 --> 00:24:42.819

NM: Not at all. 5 0 5 is highly appropriate. How many days did your child run for 10 min or more.

198

00:24:44.440 --> 00:24:46.360

PT2: I don't know that that's appropriate

199

00:24:46.760 --> 00:24:48.260

PT2: due to that level.

200

00:24:48.380 --> 00:24:59.209

PT2: you know. Can you modify it to say, okay? Can were they able to propel their wheelchair? Would you consider propelling the wheelchair part of that?

201

00:24:59.500 --> 00:25:03.550

PT2: But I don't know that I would consider propelling a wheelchair as part of physical activity.

202

00:25:03.600 --> 00:25:06.919

PT2: As you know, it is part of physical activity, but it's not

203

00:25:07.020 --> 00:25:12.310

PT2: so like I said. That's why i'm. I would put it at the low scale as a one or 2.

204

00:25:12.540 --> 00:25:16.299

PT2: Let's go with 2, because I don't really think that

205

00:25:16.380 --> 00:25:18.680

PT2: that that's appropriate for that level.

206

00:25:19.230 --> 00:25:21.880

NM: And so so you still think it's

207

00:25:22.060 --> 00:25:23.699

NM: it's somewhat appropriate.

208

00:25:24.720 --> 00:25:28.269

PT2: There might be there might be one.

209

00:25:28.470 --> 00:25:33.340

PT2: really. I'm going with one, because I don't know I don't. I don't know of any

210

00:25:34.920 --> 00:25:42.420

PT2: any of those kids that with that are in the 4 and the 5 I don't think I have to have any of those that we're running.

211

00:25:45.610 --> 00:25:46.260

PT2: So.

212

00:25:46.910 --> 00:25:48.240

thanks to.

213

00:25:51.040 --> 00:26:02.650

NM: And that concludes the questions about the survey. Is there anything else you would like to add just about your experience as a physical therapist and measuring physical activity

214

00:26:02.700 --> 00:26:05.870

in this population

215

00:26:05.930 --> 00:26:08.030

NM: before we complete our interview.

216

00:26:08.520 --> 00:26:10.499

PT2: I think I measure

217

00:26:10.630 --> 00:26:12.510

PT2: physical activity

218

00:26:12.860 --> 00:26:20.970

PT2: by observing the child. I know I have access to all types of machines that can help me

219

00:26:21.080 --> 00:26:32.649

PT2: do that. But I don't think I I always measure it based on the child. So I look at the child's facial expressions. I look how the child is

220

00:26:32.680 --> 00:26:35.909

PT2: is interacting with me. Are they interacting with me

221

00:26:35.920 --> 00:27:03.920

PT2: their normal interaction, or are they a little more? You know we need today, or they a little more, you know. Are they not themselves? Are they not participating in therapy as much as they normally would? Are they not their happy selves. You know those are the things that I look at that gauge how a treatment session, especially a treatment session goes. But that's how I know you know. I'll even say if they come in on Monday.

222

00:27:03.930 --> 00:27:15.249

PT2: Did you have you a little tied today? Did you have a busy weekend? And then they'll say, oh, yeah, we did this, this, this this and I'm like all right. No problem. And then i'll gauge my therapy sessions

223

00:27:15.270 --> 00:27:16.370

PT2: upon that

224

00:27:17.090 --> 00:27:21.370

NM: you mentioned equipment. You have machines to help you measure. What would those be

225

00:27:22.020 --> 00:27:39.079

PT2: like? We have a pulse, oscimeter where I can look at your heart rate and your oxygen saturation rate for some of those kids. That would that have some respiratory issues. So those are the things that we would we that I would that are there available to me.

226

00:27:41.270 --> 00:27:42.070

Okay.

227

00:27:42.990 --> 00:27:46.599

NM: alright, I am going to stop share

228

00:27:46.710 --> 00:27:49.970

NM: and thank you for your time. We're going to stop our recording.
