# Supplementary material for: Validity of PROMIS® Pediatric Physical Activity Parent Proxy Short Form Scale as a Physical Activity Measure for Children with Cerebral Palsy Who Are Non-Ambulatory
Source: Behav Sci (Basel). 2025 Jul 31;15(8):1042. doi: 10.3390/bs15081042 (PMC12382615; doi:10.3390/bs15081042)
Supplement: Supplementary file 1 [file behavsci-15-01042-s001.zip › Transcripts copy/PT transcripts - deidentified/PT3.docx]

WEBVTT

1

00:00:00.980 --> 00:00:02.020

PT3: 40 in

2

00:00:03.090 --> 00:00:17.029

NM: alright. Great. So now we can start. Okay. So our first question to you, PT3, Thank you for joining us today is, how do you define physical activity for children with physical, with cerebral palsy who are not full time walkers?

3

00:00:18.180 --> 00:00:32.629

PT3: I would say physical activity is them moving in in their environment, and so sometimes, and that might be moving their arms. Or if they're sitting in a chair, and they can catch a ball or go after bubbles, or feed themselves or feed a doll.

4

00:00:32.659 --> 00:00:39.040

PT3: You have some physical activity there, or just some physical activity. If

5

00:00:39.060 --> 00:00:43.780

PT3: we can get them into some sort of wheeled mobility, or just right mobility.

6

00:00:43.860 --> 00:00:50.000

PT3: whether it's a little go cart or it's a ‘go baby go’ car or it's a ‘standing Danny’.

7

00:00:50.070 --> 00:01:01.800

PT3: So think just because they can't. Kids can't independently walk. They should still be able to move and help experience that movement. I think it really rests on cognitive abilities.

8

00:01:04.080 --> 00:01:14.130

NM: great. So, as you know, the Department of Health defines physical activity, is in the activity that encompasses energy, expenditure, or activation of skeletal muscle.

9

00:01:14.290 --> 00:01:18.220

Does this definition change your mind on how you define physical activity.

10

00:01:19.070 --> 00:01:19.949

PT3: No

11

00:01:20.140 --> 00:01:20.809

NM: Okay.

12

00:01:21.080 --> 00:01:27.090

NM: How do you think physical activity differs from other types of fitness activity in this population?

13

00:01:28.030 --> 00:01:41.130

PT3: Well, I think it. It revolves around heart rate and cardio stuff. I think kids who don't have disabilities or have more mild disabilities. We can look at increasing their cardiac endurance and Cardio pulm endurance

14

00:01:41.140 --> 00:01:50.460

PT3: active. You know, endurance children who have lower independent mobility where they're using power, for instance, or they're

15

00:01:50.520 --> 00:01:51.850

PT3: just

16

00:01:52.100 --> 00:02:02.309

PT3: even on a treadmill, or they're moving with assistance. I don't think we ever see them really get up into a cardiac state where they're really working on cardiac endurance.

17

00:02:02.380 --> 00:02:07.080

PT3: And so I think that's where the biggest thing is this: You can have physical activity.

18

00:02:07.160 --> 00:02:13.259

PT3: and I think that's important for lots of different reasons. But the differences are we getting a cardiac

19

00:02:15.080 --> 00:02:19.750

PT3: Are we getting cardiac variables in there, or are we just getting movement variables?

20

00:02:22.550 --> 00:02:26.240

NM: Would one differ from the other since you brought up cardiac versus movement.

21

00:02:27.020 --> 00:02:29.980

PT3: Yeah, I think you can move without raising your heart rate.

22

00:02:30.280 --> 00:02:39.070

PT3: You could probably raise your heart rate without moving. If you scared somebody to death, or did something like that. But I think healthy, wise.

23

00:02:39.240 --> 00:02:43.099

PT3: I think you really have to be moving hard to get your cardiac.

24

00:02:43.150 --> 00:02:50.280

PT3: especially children. They have to move. They have to move with intention and a lot of movement to get their heart rate going.

25

00:02:54.040 --> 00:02:58.769

NM: When do you witness your students participate in most of physical activity during the school day.

26

00:03:00.660 --> 00:03:15.609

PT3: Well, I work in 0 to 3, and so, but we do a little preschool classroom setting, and I would say that they get most of their physical activity when we do a free play, or we have a gross motor

27

00:03:15.620 --> 00:03:29.830

PT3: play activities sometimes that involves jumping on the trampoline, sometimes that involves, and again, that could be with great assistance. Sometimes it involves walking on the balance, beam with great assistance, or doing an obstacle course.

28

00:03:29.900 --> 00:03:31.290

and then

29

00:03:31.660 --> 00:03:41.469

PT3: sometimes the movement within the music session. So we do like a 10 min music session with them, and both intentionally

30

00:03:41.500 --> 00:03:46.220

PT3: put in some music, like some dancing movement and music

31

00:03:46.310 --> 00:04:00.709

PT3: opportunities for the kids, so they can actually kind of get up and jump around a little bit, or if their parents or caregivers are holding them. They can bounce around a little bit to the beat of the music, and that really helps the kids like to move.

32

00:04:02.240 --> 00:04:14.229

NM: All right. Next group of questions. How do you measure physical activity, frequency, intensity, time and type, and children with Cp. Who may not be at full time? Walkers. This is specific to the fit principle.

33

00:04:15.170 --> 00:04:16.000

PT3: Yeah.

34

00:04:16.060 --> 00:04:35.769

PT3: I think it's difficult, I think, with the advent of wearable technology, which is actually the technology is increasing and improving so much that there's potential that we can really look at using some wearable technology type of

35

00:04:35.780 --> 00:04:53.069

PT3: equipment to measure those those things. Otherwise, I think it's a little bit difficult. You can. You know you can measure how far a child walks, or how I, however many minutes he walks, or she walks during the school day, you know, if they're moving in between classes or in between stations.

36

00:04:54.200 --> 00:05:00.269

PT3: so I think. But for me in 0 to 3, it's more just. It's kind of like a

37

00:05:00.340 --> 00:05:07.760

PT3: I all or nothing. Did they get up and move today, you know, or did they come in in their stroller and sit on somebody's lap.

38

00:05:07.960 --> 00:05:10.700

PT3: and then just kind of get carried from place to place. So

39

00:05:12.240 --> 00:05:13.420

NM: thank you so much

40

00:05:13.530 --> 00:05:25.399

NM: Do these kiddos need assistant and completing their everyday activities, and during which activities do you see them doing physical activity in this, in the population that you serve?

41

00:05:26.160 --> 00:05:31.130

PT3: Some need assistance, including to complete their You know

42

00:05:31.260 --> 00:05:32.789

PT3: their activities in their

43

00:05:33.010 --> 00:05:50.159

PT3: personal activities. We try to really build an intention. And so, you know, every kid kind of has a job. So you have to go get your own crayons. If you're able to do that, you have to hold the bubbles you have to pass out the scarfs and the little Maracas, or whatever we're playing with that day.

44

00:05:50.170 --> 00:06:05.070

PT3: But we do like a lot of sometimes, you know, clean up and set up activities. So we try to embed and in model for the parents and caregivers how to embed movement within stuff they're doing in the everyday.

45

00:06:05.080 --> 00:06:17.690

PT3: and so that helps because we can say, you know parents can say. Oh, he helps at the table. Now I work with kids who are live in Inner City. And so those kids walk and take the bus a lot.

46

00:06:17.940 --> 00:06:31.249

PT3: And so it's the rare kid that's in a car and in a car seat. So sometimes we build in their activity instead of riding in a stroller to the bus. Could he walk? Sometimes the parents are like Well, that'll add an hour to my trip.

47

00:06:31.290 --> 00:06:48.080

PT3: and so it really depends on what part if they're trying to get the therapy? No, if they're trying to get home. So then they have some time to do that. But I think it's really important, especially with the young children, to embed things into what families already doing. The family routine is already occurring, and so

48

00:06:48.090 --> 00:07:02.059

PT3: lot of times again. We've introduced some of those movements and music things that you find on Youtube, and really ask the parents to have the children watch those things where they get up and jump around and turn around and do the silly dance contests, and all of that

49

00:07:02.880 --> 00:07:07.729

PT3: to try to build that into their play instead of just sitting and passively watching screens

50

00:07:09.260 --> 00:07:16.579

NM: sounds great. Lastly, in this segment. Do you think they should participate in more or less of these activities? And why.

51

00:07:17.710 --> 00:07:30.409

PT3: I think the more movement you can get in a child in Chicago we we have. We really push swimming as much as we can. There's several park districts that have indoor pools. There's a hospital that has an indoor.

52

00:07:30.820 --> 00:07:42.950

PT3: therapy pool that the kids can sign up for in 10 week blocks, and they just keep you sign up, and then you do your 10 weeks, and then you go to the back of the line. And so we really try to get kids movement

53

00:07:43.100 --> 00:08:05.920

PT3: kids who live in low income neighborhoods where it's dangerous. The challenge is, you can't go out and walk around the block. You. It's dangerous. And so then we think about. That's why we have them come into the center, so can we. We have tricycles there. We have scooters there. We have a basketball net there like I said the trampling. And so the kids come for 2 hour sessions, and they can come once a week or twice a week.

54

00:08:05.930 --> 00:08:06.820

PT3: and so

55

00:08:07.370 --> 00:08:19.320

PT3: can we provide that opportunity of just fun moving around and being created. We also have a couple of ‘go baby go’ cars and some of the bumper cars. And so, even if they don't have ambulation

56

00:08:19.420 --> 00:08:26.700

PT3: potential, can we get them in some sort of moving kid, appropriate vehicle, or a ‘just right’ mobility tool

57

00:08:26.860 --> 00:08:45.529

PT3: that they can at least experience some of that movement cognitively again, even though it's just doing a joystick versus getting their arms and minds going and alot of the kids. Their lives are just it's very passive. Their lives are very passive, and so we try to just build in the

58

00:08:45.540 --> 00:08:48.599

PT3: the joy of moving and dancing and jumping around.

59

00:08:50.510 --> 00:09:04.709

NM: That's great. Thank you so much. Do you address promoting physical activity during your physical therapy sessions, and you you mentioned a bit of this. So specifically, while you're actually doing your therapy sessions, Are you actually working on components of what you talked about?

60

00:09:05.580 --> 00:09:25.000

PT3: Yes, and we're working on it with the whole family. Because, again, if the child is sedentary, the whole family is sedentary. So we really do try to get the parents and even the siblings that come along. If siblings come along involved in jumping around, and you know, moving around a little bit, and so trying to

61

00:09:25.010 --> 00:09:35.650

PT3: impress on the parents. That motion is lotion. That's what we say. A lot. Motion is lotion, and so and just they'll feel better when they're moving. And so that is helpful.

62

00:09:35.690 --> 00:09:50.739

PT3: So yeah, and we really do try to problem solve with the families. How can we embed good movements or safe places to go in the city, the museums, the Botanical gardens, different places that they can go that are free

63

00:09:50.780 --> 00:10:10.119

PT3: to walk around grocery store. Can you walk around sometimes with with the older kids the 3 year olds, and if they have older siblings the OT did a really nice job and put up like a Bingo card or a treasure hunt at the local grocery store, and so the kids pick out what they want to try to find, and then they go on a hunt so that helps.

64

00:10:10.130 --> 00:10:22.119

PT3: but trying not to add the burden. Many of our families physical movement go into the gym just isn't in the realm of possibilities. So we just try to build it into their what they're doing already

65

00:10:24.480 --> 00:10:43.399

NM: wonderful, and you gave us some nice examples of that. What components of physical activity specifically do. And you you address. You talked about getting me the movement. But here are some examples specific to cardiovascular endurance, muscle, activation, and energy expenditure. Are you working with some of these components of physical activity.

66

00:10:43.540 --> 00:10:47.060

PT3: we're definitely working on muscle activation

67

00:10:47.120 --> 00:11:05.650

PT3: and just patterns of movement that in alignment activities and and more developmental things. So within the developmental sequence, we don't really look at like emg stuff or muscle power as much or in. We don't really have a great way to measure cardiovascular

68

00:11:05.660 --> 00:11:09.069

PT3: when the kids are here. But you so

69

00:11:09.180 --> 00:11:17.109

PT3: in that realm of objective measurements, no subjective measurements, and just trying to get people off the couch. Yes.

70

00:11:19.780 --> 00:11:20.660

NM: wonderful

71

00:11:20.810 --> 00:11:32.849

NM: and okay for the last question. Segment. Do you address promoting physical activity that occurs outside of your Pt session? You talked about what you do while you're with the child. How are you addressing it outside of Pt.

72

00:11:32.860 --> 00:11:52.310

PT3: Yeah, we do actually try to get them to think about ways that they can carry over things in their neighborhood. So sometimes we'll meet at the park, because families are a little bit afraid of swings and slides, and we'll show them, or the climbing equipment, and we'll work with the child. So the parent gets comfortable that the Kid is on the climbing equipment

73

00:11:52.320 --> 00:12:10.840

PT3: again. The Botanical Gardens is is very nice, and it has a kid area, so they kind of have to climb around and through things. And then the children's museums. And again, there's free days or the Chicago Public Library. You can get a a card. If you have a library card you can get a day pass

74

00:12:10.850 --> 00:12:22.279

PT3: to any of the museums in Chicago, and so the families can go for free. We also have a free zoo that has a lot of walking paths and climbing stuff. So we try to say, You know, on a nice day.

75

00:12:22.460 --> 00:12:28.450

PT3: here are some activities that you could do outside if the neighborhood is safe. If the

76

00:12:28.510 --> 00:12:37.270

PT3: if the area is safe, and if it's not safe, well, here are some safe indoor places. You can go. And again, the Chicago Park districts are fabulous.

77

00:12:37.280 --> 00:12:50.980

PT3: and so they have lots of classes and lots of swimming pools for the kids. So we we work hard to try to get families to take advantage of the good things that Chicago offers, and

78

00:12:51.070 --> 00:12:56.810

PT3: it's kind of it works for us, because the good things they offer are things that are interesting to the kids

79

00:12:57.700 --> 00:13:11.750

NM: That's wonderful. So you already kind of answered my next prompt, which was recommended community programs or events to help increase physical activity. So my my last prompt related to this was, what type of equipment have you recommended?

80

00:13:11.760 --> 00:13:29.989

NM: Help, improve home and community engagement you talked about? Go, baby, go! Some of the things within Pt. But is there anything anything specific, as it relates to equipment that you recommended to improve the home and community engagement of physical activity outside of your your clinical setting.

81

00:13:30.280 --> 00:13:59.310

PT3: And so it really depends on the child's capacity and performance. So if, for instance, that they're starting to walk and they need a walker, we try, and they we try to get them that that equipment. We either loan it to them or we have a kids equipment network that we can get kids equipment. So mobility equipment, if they need a cane, more walkers or gate trainers, and then we can just distribute them to the families. And when the child kind of outgrows it, or doesn't need it anymore.

82

00:13:59.370 --> 00:14:21.819

PT3: They bring it back. We have a treadmill with a Lite gate harness at the clinic, and so almost every kid gets put onto the treadmill at least 4 or 5, you know a session of 2 min, 5 min during the 2 hour session. It's funny, though. The kids that don't need the treadmill are the first ones to come over and turn it on. It's a little baby treadmill, and so every kid loves to run on the treadmill.

83

00:14:21.940 --> 00:14:47.409

PT3: and we have the bicycles, too. But for to take home we don't send the bicycles home, but we'll try to get them gait trainers. We'll try to get them walkers to take home crutches if they need it, and then for children who need more positioning equipment will try to get them a kid cart or a positioning stroller. So at least they're not falling over and out of their ‘great Co’. Stroller that they can be upright and positioned well, so they can start to participate a little bit better

84

00:14:47.420 --> 00:15:06.229

PT3: instead of just laying flat in their stroller. And so it's kind of whatever. If the family is interested in that in a piece of equipment, we'll try to get it for them for them to take it home. The bigger issue we actually have is the family is like oh, no, we don't want that because we don't want our child to look disabled in the community.

85

00:15:06.290 --> 00:15:20.290

PT3: And so then we think about Well, what can they use at home inside in the secret of their own house? And and again, could they use a walker, or could they use a gait trainer in indoors? And so for the families that don't want

86

00:15:20.510 --> 00:15:28.440

PT3: to go out into the community with equipment, we try to at least talk them into having stuff at home. So the child has the opportunity to practice

87

00:15:28.490 --> 00:15:30.000

upright mobility.

88

00:15:30.070 --> 00:15:44.210

PT3: or just try to figure out well in your house. What can you hold on to? Can you practice standing standing in the corner facing outwards while you're, you know, doing some reading a storybook or something, so they at least get to make

89

00:15:44.710 --> 00:15:49.970

PT3: some sort of action or some sort of movement, even if the family is refusing equipment.

90

00:15:51.210 --> 00:16:11.440

NM: That's wonderful. So now I'm. Going to share the promis scale, which is a parent proxy, assessment of physical activity and hip intensity. This was developed for families of children that had progressive disorders by the Nih. So i'm going to ask you just to look over these these 8 questions and then

91

00:16:11.450 --> 00:16:12.369

NM: hold on 1 s.

92

00:16:30.330 --> 00:16:45.489

NM: Okay, so what I would like you to look at and answer as I go through these 8 questions is, how appropriate is this question when addressing physical activity, intensity specifically in children that are non ambulatory. So that's Gms. Is 44 and 5.

93

00:16:45.500 --> 00:16:59.819

NM: This is helping us to try to figure out a measure that can help work with our population, because, as you mentioned, not so many are out there. Alright, so i'm going to actually want to scale from 0, 0 being not related at all, and 5 being highly appropriate.

94

00:16:59.830 --> 00:17:10.579

NM: When I ask you for each question, just give me your ranking, and then why? So? The first question is, how many days did your child exercise or play so hard that his or her body got tired?

95

00:17:10.589 --> 00:17:25.009

NM: How would you rate this level or appropriateness for a child at GM message level one-five would be 0, not really at all, or any 5 highly appropriate, or any alone, any points along that

96

00:17:25.369 --> 00:17:27.880

PT3: a 0 to one how it is 0.

97

00:17:30.580 --> 00:17:31.809

NM: And why?

98

00:17:33.720 --> 00:17:50.549

PT3: Because I think that they probably don't exercise and think just if they're trying to move, their body might get tired. But i'm not sure that a parent would consider that exercise or play. And I think kids at 4 or 5,

99

00:17:50.740 --> 00:17:57.610

PT3: and my own personal opinion is they're not playing that hard that they get. Their body gets tired.

100

00:17:57.730 --> 00:18:00.479

PT3: Their mind might get tired, but

101

00:18:00.660 --> 00:18:01.620

PT3: and they might

102

00:18:02.010 --> 00:18:04.109

PT3: get tired from the effort of it.

103

00:18:04.750 --> 00:18:10.210

PT3: But I don't think they actively participate in engage in exercise or play.

104

00:18:12.640 --> 00:18:20.660

NM: Thank you. Second question. How many days did your child exercise really hard for 10 min or more, would you say 0 not related at all.

105

00:18:20.680 --> 00:18:24.870

NM: or anything along the spectrum up to 5, being highly of for you.

106

00:18:25.900 --> 00:18:41.380

PT3: I would think, from a parent point of view. This might get a 3, 4, probably a 4, and because i'm thinking, if i'm thinking of a parent is like, oh, we're going to do your exercises, or we're going to do what the physical therapist told us to do.

107

00:18:41.390 --> 00:18:47.430

PT3: and for 10 min or more, and that if they perceive the child is

108

00:18:47.740 --> 00:18:49.130

PT3: working hard.

109

00:18:49.470 --> 00:18:54.170

PT3: Then I think they would perceive that that, working hard at that exercise

110

00:18:54.360 --> 00:18:55.140

PT3: it

111

00:18:55.330 --> 00:18:56.540

PT3: that would

112

00:18:57.290 --> 00:19:13.970

PT3: yeah, working hard at that exercise, and they'd work on it for more than 10 min. So it's more again of a cognitive thing, whereas the as opposed to the child's doing 100 jumping Jacks in 10 min really hard. But I think I could see a parent saying, oh, yeah, we, my child, exercise really hard, and

113

00:19:13.980 --> 00:19:30.000

PT3: the blood pressure didn't go up the heart rate didn't go up. They, the kids were really kind of focused and trying to do something that somebody had showed the parents of the parent was doing so. It it's more of an attempt, and they probably did it for more than 10 min.

114

00:19:30.110 --> 00:19:33.530

NM: Right? Okay. So in terms of physical activity

115

00:19:33.570 --> 00:19:41.099

NM: for children at Genesis level. How appropriate is Question Number 3. How many days did your child exercise so much that he or she breathed hard

116

00:19:43.150 --> 00:19:52.569

PT3: going, and with like a two-ish, a 2, 1, 2 down in that area. Because again, I think the the kids will start breathing hard if they have to work a little bit.

117

00:19:52.970 --> 00:20:02.910

PT3: you know. And so because they're de-conditioned because they haven't been exercising that that they probably are sort of getting out of breath and breathing hard.

118

00:20:03.430 --> 00:20:07.330

PT3: And so that would be an appropriate question, I think, for the parents

119

00:20:09.490 --> 00:20:20.039

NM: great. We're right along. Number 4. How many days was your child so physically active that he or she sweated. How would you rate? How, how, how appropriate this is? And

120

00:20:20.460 --> 00:20:35.239

PT3: I don't think this is appropriate at all for the Forum 5. Because again, first of all, I'm. From a parent point of view, i'm like why you asking me a question when you know my kid doesn't move. And then so it was almost insulting to say they were so physically active that they sweated.

121

00:20:35.280 --> 00:20:47.199

PT3: And for some of these kids, actually, they might break into a sweat because they've been sitting up for 3 min, you know so. But I think it's it's almost insulting from my point of view.

122

00:20:47.360 --> 00:20:58.410

PT3: because you're implying. Or this? The question obviously is implying a lot of physical activity, and the 4 and 5 don't have a lot of physical activity, but they still might sweat with a little bit of physical activity.

123

00:20:58.730 --> 00:21:02.970

PT3: but I don't think they even get that level of

124

00:21:03.360 --> 00:21:05.310

PT3: generating muscle stuff.

125

00:21:05.780 --> 00:21:07.670

NM: Great all right, Number 5.

126

00:21:08.060 --> 00:21:14.430

NM: How many days. Did your child exercise or play so hard? They had to her muscles burned. How appropriate would you think this one is?

127

00:21:14.490 --> 00:21:19.719

PT3: I think again, this one is not appropriate at all for the kids who are level 4 and 5. Because

128

00:21:20.500 --> 00:21:27.640

PT3: if I don't think they're playing or exercising that hard just because of their limitations of being a Gmf. Cs. 4 or 5

129

00:21:27.740 --> 00:21:30.929

PT3: that their muscles firmed.

130

00:21:31.030 --> 00:21:35.959

PT3: I think, for kids especially, you have to be really rocking it

131

00:21:36.220 --> 00:21:38.790

PT3: to get to muscle, fatigue, and muscle learning.

132

00:21:38.890 --> 00:21:42.169

PT3: unless you have muscular dystrophy, and then it's a danger.

133

00:21:43.850 --> 00:21:49.539

NM: Excellent number 6. How many days is your child? Exercise her place so hard he or she felt tired.

134

00:21:50.320 --> 00:22:04.099

PT3: I think this again goes to more of a fair question for the fours and 5 one number which is upgrade it. I would say a 4 or 5, because I think I need you to pick one. Now. 5, I go 5, 5,

135

00:22:04.110 --> 00:22:26.210

PT3: I i'll go 5 with this one, because I think again, they, though they, the parents, will perceive parents who are involved, who, where ex movement is important to their family and their child that they'll try to get that child to be active exercise and play, and the kids will get tired, and parents, I think, recognize that the children do fatigue.

136

00:22:26.220 --> 00:22:27.110

PT3: and so

137

00:22:27.550 --> 00:22:31.030

PT3: I think that's a fair question. I think 5 is an appropriate one for that.

138

00:22:31.300 --> 00:22:36.609

NM: Thank you. Number 7. How many days with your child physically active for 10 min or more.

139

00:22:37.230 --> 00:22:38.080

PT3: 5

140

00:22:38.870 --> 00:22:40.110

PT3: for this one.

141

00:22:41.410 --> 00:22:44.279

PT3: I think, because again, that the the

142

00:22:44.520 --> 00:22:47.679

PT3: for the fours and 5 physically active.

143

00:22:48.790 --> 00:22:58.330

PT3: they'll define it in some interesting way that I I think it. It ends up being like how the parent defines physically active. But again, maybe if you were a level 4,

144

00:22:58.340 --> 00:23:14.079

PT3: but your level, even your level. 5 kids is physically active, maybe actually just sitting on the couch, or being, you know, sitting supported, but being involved in an activity of some nature. So pushing on a switch, doing something like that. So

145

00:23:14.980 --> 00:23:20.699

NM: some and the last question is, how many days did your child run for 10 min or more. How appropriate would you rate

146

00:23:21.370 --> 00:23:22.290

PT3: one

147

00:23:22.890 --> 00:23:26.259

PT3: your love, if your level of your level fours and 5 it's just aren't running

148

00:23:30.900 --> 00:23:36.680

NM: excellent. We are at the end of the interview. Is there anything you would like to add about physical activity as it relates to this population?

149

00:23:37.070 --> 00:23:53.630

PT3: No, I think it's great that you're looking at it, and I think the technology will help you, I mean, and I would like to see I was talking with a biomedical engineer. I would like to see some wearable technology that's embedded in shorts or a shirt that the kids could wear. They could pick up those biometrics even better than the watches.

150

00:23:53.680 --> 00:23:56.500

PT3: So it'll be interesting to see what you think with your research.

151

00:23:58.960 --> 00:24:00.809

NM: What are the names of the shorts?

152

00:24:01.090 --> 00:24:14.319

PT3: They're not invented, yet I wanted them to put some some sort of sensor in the shorts, or in a t-shirt that the kid could wear that would measure some of these things that you're talking about like

153

00:24:14.350 --> 00:24:19.170

PT3: I mean, maybe it's just sitting up and sitting there for 3 min. The kids heart rate spikes.

154

00:24:19.250 --> 00:24:31.130

PT3: you know. And again, especially with little babies when we're and we're looking at kids with Sm. A. It would be really nice to know when we're we're pushing them too much. So it's a danger to their cells.

155

00:24:31.250 --> 00:24:34.419

PT3: And the technology exists

156

00:24:34.460 --> 00:24:40.519

PT3: for adults. I mean, the technology is out there, and so it' be. Somebody just has to put the 2 and 2 together.

157

00:24:41.950 --> 00:24:43.160

PT3: I think it should be you.

158

00:24:44.870 --> 00:24:50.920

NM: Thank you so much, PT3. We are going to conclude the recording hold on 1 s

159

00:24:51.070 --> 00:24:56.749

NM: extension, 5, 1, 3 spot I from an airport courtesy phone.
