# Supplementary material for: Validity of PROMIS® Pediatric Physical Activity Parent Proxy Short Form Scale as a Physical Activity Measure for Children with Cerebral Palsy Who Are Non-Ambulatory
Source: Behav Sci (Basel). 2025 Jul 31;15(8):1042. doi: 10.3390/bs15081042 (PMC12382615; doi:10.3390/bs15081042)
Supplement: Supplementary file 1 [file behavsci-15-01042-s001.zip › Transcripts copy/PT transcripts - deidentified/PT13.docx]

WEBVTT

1

00:00:01.750 --> 00:00:07.649

NM: Hi! Thank you so much for joining us today as we ring to talk about physical activity

2

00:00:07.830 --> 00:00:15.200

NM: in children with Cp. Who are not ambulatory, so I I do have a couple of questions for you. The first half is going to be

3

00:00:15.310 --> 00:00:32.599

NM: a series of questions with some follow up prompts, and then the second half will be of a view of a Nih survey called the Promise. Parents reported a proxy survey, and so we'll talk about that survey. I'm going to ask some questions related to that that surveys appropriateness to this population.

4

00:00:32.770 --> 00:00:39.170

NM: Okay. So if I sound scripted this because I am so forgive me in that I'm trying to be as streamlined as possible.

5

00:00:39.340 --> 00:00:42.039

NM: Okay, so my first question to you.

6

00:00:42.330 --> 00:00:51.320

NM: PT13, is: how do you define physical activity for children with Cp. Who are not full time Walkers, Tmf. Cs levels 4 and 5.

7

00:00:52.980 --> 00:00:59.580

PT13: I think I define physical activity as something that they're doing that's elevating their heart rate

8

00:00:59.660 --> 00:01:11.330

PT13: that they do it for a sustain period of time. Not necessarily walking. It could be moving, their arms, crawling across a mat, scooting across the that something that they're sustaining a higher

9

00:01:11.440 --> 00:01:13.499

PT13: level than what they do at baseline

10

00:01:18.040 --> 00:01:18.920

NM: Great.

11

00:01:19.020 --> 00:01:30.790

NM: So the Department of Health defines physical activity as any activity that encompasses energy expended, and activation of skeletal muscles. Does this definition change your mind about how you define physical activity?

12

00:01:31.170 --> 00:01:31.850

PT13: No.

13

00:01:34.040 --> 00:01:38.979

NM: And how do you think physical activity differs from other types of fitness activities.

14

00:01:45.220 --> 00:01:47.909

PT13: What are, what are other types of fitness activities?

15

00:01:50.540 --> 00:01:59.980

NM: Well, great question. So what do you think about fitness when you think about, you know, even just maybe for yourself, Fitness would encompass ideally something more structured.

16

00:02:00.690 --> 00:02:04.670

NM: and maybe with a fitness goal attached to it, or some kind of

17

00:02:04.710 --> 00:02:13.169

NM: kind of the goal. So how do you think physical activity differs from other types of fitness activities?

18

00:02:14.220 --> 00:02:16.140

PT13: I would.

19

00:02:16.270 --> 00:02:20.260

PT13: I guess, I would say that physical activity is

20

00:02:20.410 --> 00:02:24.490

PT13: more of a general movement, whereas fitness is is

21

00:02:24.580 --> 00:02:27.930

PT13: now that you're thinking of it that way is more

22

00:02:27.990 --> 00:02:29.420

PT13: of a scripted

23

00:02:29.900 --> 00:02:35.279

PT13: like a scripted procedure like going to like a specific kind of a fitness class.

24

00:02:35.320 --> 00:02:39.340

PT13: So something specific, for you know, a specific type of training

25

00:02:39.980 --> 00:02:41.800

PT13: like ballet or

26

00:02:41.860 --> 00:02:48.650

PT13: Pilates. I guess those are to me, I guess, thinking about it in that sense those are more like

27

00:02:49.610 --> 00:02:51.110

PT13: fitness. We're

28

00:02:51.140 --> 00:02:58.420

PT13: not necessarily physical activity, whereas, like physical activity, could be folding so, for someone could be folding their laundry because they're exerting themselves

29

00:03:03.520 --> 00:03:11.289

NM: and kind of tying it to this population. How would you say physical activity for this population differs from fitness activities

30

00:03:12.470 --> 00:03:15.940

PT13: if it does at all. I don't know. I'm just the one that out there

31

00:03:16.040 --> 00:03:22.879

PT13: I think physical activity is more of a generalized exertion of yourself Moving

32

00:03:23.420 --> 00:03:28.810

PT13: moving in some manner versus a fitness is something that could be more prescribed

33

00:03:29.040 --> 00:03:32.380

and given to you. That's more regimented, structured.

34

00:03:37.520 --> 00:03:43.260

NM: great. And when do you witness your students participate most in physical activity during the school day?

35

00:03:45.210 --> 00:03:59.169

PT13: I actually I don't know that I do. To be honest. I don't know that for my, for my to the kids that I see Specifically, I don't know that they participate in a lot of physical activity during their school day.

36

00:04:01.640 --> 00:04:02.800

PT13: which is unfortunate.

37

00:04:03.170 --> 00:04:18.229

NM: Would you say that's because of the type of school it give me a little bit of reference.

PT13: Yeah, I think that they don't they don't have like a PE class. They don't have like a a prescribed recess most of they're all wheelchair bound so, and they're non-mobile they so

38

00:04:18.510 --> 00:04:24.620

PT13: for them. If they were more to like, get out on, they'd have to be taken out on a mat, and they don't usually get out of their seating

39

00:04:25.320 --> 00:04:26.660

PT13: when they're in the school day.

40

00:04:27.010 --> 00:04:28.309

NM: Hmm. Okay.

41

00:04:32.130 --> 00:04:34.409

NM: Thank you. All right. Next question.

42

00:04:34.550 --> 00:04:37.790

NM: How do you measure physical activity, frequency.

43

00:04:37.890 --> 00:04:55.139

NM: intensity, time and type? So i'm looking at the fitt principle here, and children with Cp. Who are not full time Walk, Walkers and I'll say that again, how do you measure physical activity, frequency, intensity, time and type in children with Cp. Who are not full Time Walkers

44

00:04:56.890 --> 00:05:00.580

PT13: frequency would probably be.

45

00:05:04.150 --> 00:05:10.880

PT13: I guess i'd have to look at their daily what they do during the day, and then seeing what things may be more physically

46

00:05:11.020 --> 00:05:22.159

PT13: taxing on their body, and then also asking them what if they're able to answer me what their level of intensity is on a scale, or like a perceived exertion Scale

47

00:05:22.520 --> 00:05:28.070

PT13: the time probably, you know, whatever time it takes them to do the test. I know that for some.

48

00:05:28.500 --> 00:05:35.719

PT13: for one actually one child that i'm thinking of in particular, she had, like for her physical activities, probably pushing

49

00:05:35.770 --> 00:05:37.670

PT13: from

50

00:05:37.780 --> 00:05:40.899

PT13: you, have 1 one classroom to another, or from like

51

00:05:40.940 --> 00:05:43.539

PT13: the residents to the school.

52

00:05:43.800 --> 00:05:49.779

PT13: and that for her is a physical activity, because she's exerting her, she's definitely exerting herself. So that would be like the time for that.

53

00:05:50.970 --> 00:05:55.060

PT13: And then how many days of a week she goes to school also based on that frequency.

54

00:05:59.790 --> 00:06:05.460

NM: and even using the examples you gave, Do they need assistance to complete these activities?

55

00:06:05.500 --> 00:06:08.290

NM: and during which activities

56

00:06:08.340 --> 00:06:10.610

NM: what they need assistance? And if they

57

00:06:10.760 --> 00:06:15.050

NM: are getting assistance, is it for part of it, or just, or the whole task?

58

00:06:15.530 --> 00:06:21.429

PT13: Typically this the one student that i'm thinking of? She does not need assistance

59

00:06:21.630 --> 00:06:33.610

PT13: for the task. There is an occasion, if she's having of like a respiratory illness of some sort, that she would, may need some assistance to complete the entire way distance

60

00:06:33.740 --> 00:06:39.859

PT13: from Point a. To Point B. I think that, and also maybe some verbal encouragement, because she's only 5 years old.

61

00:06:44.250 --> 00:06:49.819

NM: So when thinking about other physical activity tasks that you may measure.

62

00:06:50.480 --> 00:06:56.359

NM: Do you have any that you may want to? You only want to list in terms of how much assistance they may need.

63

00:06:57.590 --> 00:07:09.440

PT13: I think off like. So i'm thinking of someone else who's who does like scooting on the scooting on the floor, and some like commando crawling. He definitely needs

64

00:07:09.950 --> 00:07:21.989

PT13: some a lot. Just a lot of encouragement to get there. He, on occasion will need, you know, Mid to MoD assist. If he throws his weight too far just to come back to the midline.

65

00:07:23.430 --> 00:07:31.159

NM: Is he able to get down for himself, or do you have to give it,

PT13: and he needs, and he is completely dependent for transfers to the ground

66

00:07:31.200 --> 00:07:32.450

PT13: that, too. Thank you.

67

00:07:32.500 --> 00:07:33.230

NM: No problem.

68

00:07:38.270 --> 00:07:38.840

right.

69

00:07:42.490 --> 00:07:48.410

NM: And do you think these children should participate in more or less of these activities, and why

70

00:07:49.850 --> 00:08:00.560

PT13: I think they should participate in the more I think, is that the more you move, and the more you're able to repeat it, you improve your cardiovascular endurance. You improve your the strength in your body.

71

00:08:00.660 --> 00:08:03.240

PT13: There's no other way to improve your

72

00:08:03.410 --> 00:08:08.950

PT13: for especially for children. Repeated motions, is going to be the most important for strengthening, because they're not going to do

73

00:08:09.020 --> 00:08:18.560

PT13: reps and 3 sets of 10. So getting them on the ground. To functionally move and move throughout their space is the the best and is the only way that they're going to get there

74

00:08:20.430 --> 00:08:25.480

PT13: to really move to really move the needle in their progress and to become more functional.

75

00:08:28.890 --> 00:08:35.060

NM: Great. All right. Next question. Do you address promoting physical activity during your actual physical therapy sessions.

76

00:08:35.940 --> 00:08:36.819

PT13: Yes, I do.

77

00:08:37.980 --> 00:08:40.099

NM: And how would you do? How do you do this?

78

00:08:40.890 --> 00:08:57.259

PT13: for some kids, It's sitting on a bolster and having them, you know, march in place while singing or dancing, and then incorporating that within weight bearing and through their arms and legs, so incorporating it so that they're going from a more, maybe static sitting balance

79

00:08:57.400 --> 00:09:03.050

PT13: to then a standing. So then, and going back and forth. So you're not just staying in one static position.

80

00:09:03.480 --> 00:09:07.179

PT13: Also, you know, do it

81

00:09:07.940 --> 00:09:25.010

PT13: making it dynamic through more transition. So, coming up from the so, laying down flat on the stomach, coming up to like a modified, quadruped, tall kneell kind of a position so keeping the as the trying to incorporate. So the whole body is moving and really getting that weight, bearing through as much of

82

00:09:25.100 --> 00:09:27.800

PT13: as many of the limbs as possible.

83

00:09:32.480 --> 00:09:49.299

NM: Those are all great examples. So what what components of physical activity are you addressing in these sess these sessions. So, for example, are you doing? Are you focusing more on mobility, cardiovascular endurance, muscle activation? You know those those are just some examples. What are the components that the key components that you are working on

84

00:09:49.310 --> 00:09:52.710

NM: in the sessions. When you're working on physical activity in your Pt sessions?

85

00:09:53.100 --> 00:09:57.590

PT13: Usually it's the cardiovascular endurance. It's the

86

00:09:57.670 --> 00:10:03.040

PT13: and also, like the co contraction of like your quads and hamstrings, or your gluts and abs.

87

00:10:03.220 --> 00:10:11.609

PT13: and the the muscle endurance for them for those muscle for those components to work so that they can do it repeatedly

88

00:10:12.080 --> 00:10:15.110

PT13: and to progress, to to to to decrease fatigue.

89

00:10:24.500 --> 00:10:26.500

NM: All right, Great and

90

00:10:26.790 --> 00:10:29.160

NM: okay. So you did focus on that. Okay, next question.

91

00:10:30.110 --> 00:10:35.319

NM: Do you address promoting physical activity outside of your Pt sessions? And if so, how?

92

00:10:36.730 --> 00:10:39.969

PT13: for those who are able to.

93

00:10:40.350 --> 00:10:53.759

PT13: who are small, small enough because our school staff is not able to really, or in our recreation Staff is not actually able to lift bigger children, but for those who are able to be transferred to the floor with an assistance.

94

00:10:53.890 --> 00:11:02.920

PT13: getting them out during recreational activities, getting them onto the mat so that they can move around and have it be able to explore on the mat.

95

00:11:03.340 --> 00:11:07.349

PT13: Also i'm encouraging school staff to have

96

00:11:07.500 --> 00:11:16.229

PT13: to have the children manually push their chairs if they're able to. When you typically throughout the day, they're provided assistance for that.

97

00:11:18.390 --> 00:11:23.130

PT13: or even just incorporating their arms more and whatever activities are being done in the classroom.

98

00:11:30.880 --> 00:11:36.089

NM: Have you recommended any community programs or events to your students to help increase physical activity

99

00:11:36.660 --> 00:11:39.399

PT13: not currently in the population that i'm working in.

100

00:11:42.810 --> 00:11:50.230

PT13: Most of what I'm doing is residential.

NM: You're doing a residential. I want to ask you,

PT13: yeah, yeah, Most of it is residential. So

101

00:11:51.500 --> 00:11:54.649

PT13: our rec staff is like our is more or less our community.

102

00:11:54.710 --> 00:11:56.489

NM: Yeah, that that that definitely counts.

103

00:11:56.530 --> 00:12:07.050

NM: What type of equipment have you recommended to help improve home or community engagement out of it of physical activity outside of your clinical setting, which is where you treat.

104

00:12:07.140 --> 00:12:09.390

NM: and this situation

105

00:12:09.460 --> 00:12:14.330

PT13: when appropriate. We've I've tried to incorporate walkers and gait trainers.

106

00:12:15.980 --> 00:12:19.330

PT13: or even just some handhold. or just standing.

107

00:12:21.520 --> 00:12:26.299

PT13: I'm trying to think of any like any equipment. I think that's really for equipment. Why, that's it.

108

00:12:28.870 --> 00:12:29.660

NM: Okay.

109

00:12:29.930 --> 00:12:35.440

NM: All right. Great. So now we're going to talk a little bit more about this a survey. So i'm gonna pull it up on the screen

110

00:12:36.050 --> 00:12:37.510

NM: and

111

00:12:37.990 --> 00:12:40.209

NM: give you just a few minutes to look at it.

112

00:12:41.980 --> 00:12:51.459

NM: So you You did mention some assessments that you may have used, perceive exertion and other things of that nature. So this actually does ask some questions about. You know

113

00:12:51.560 --> 00:12:53.900

NM: how a child may feel in terms of

114

00:12:53.960 --> 00:13:13.320

NM: You know. How many days a week have they exerted themselves for the parent to answer. So this was created for children that were not typically developing, which is great. But, again, how appropriate. Is it to children that are not ambulatory? We don't know. So i'm asking Pts. And parents and i'm going to ask you to rate each question, so we'll go one by one. I'm going to ask you to rate it 0.

115

00:13:13.330 --> 00:13:16.850

NM: This this is not applicable to this population 5.

116

00:13:16.910 --> 00:13:21.310

NM: It is relatable. Okay, and give me a scale ranking. And then I'm asked you to tell me why.

117

00:13:21.550 --> 00:13:23.599

NM: Okay, so let me go ahead and

118

00:13:23.750 --> 00:13:26.729

NM: move to my next session section of

119

00:13:27.170 --> 00:13:29.850

NM: notes. Okay. So for the first question.

120

00:13:30.920 --> 00:13:41.040

NM: how many days did your child exercise a place so hard that his or her body got tired again? Remember, these are caregivers, the parents that are going to be asked, and it's a physical activity, intensity, scale.

121

00:13:41.210 --> 00:13:48.990

NM: How would you rate this question? 0? Not related at all on a scale from 0 to 5, 5 being highly appropriate. How would you rate this question, and why?

122

00:13:49.840 --> 00:13:59.099

PT13: I think a 3, especially if you're talking about kids who are not ambulatory, because then their ability, their cognitive ability.

123

00:13:59.190 --> 00:14:04.249

PT13: typical not always, but typically their cognitive ability and communication

124

00:14:04.520 --> 00:14:10.289

PT13: is generally also altered. So they may or not be able to answer the question.

125

00:14:10.630 --> 00:14:13.630

PT13: because it's. It's hard to tell in a child who's

126

00:14:14.160 --> 00:14:15.780

PT13: nonverbal

127

00:14:16.510 --> 00:14:20.310

PT13: if they are, if they were, if their body was tired.

128

00:14:20.350 --> 00:14:21.080

NM: Hmm.

129

00:14:23.340 --> 00:14:26.889

PT13: and I and it. I guess it also depends on the age of the child, because

130

00:14:26.950 --> 00:14:37.249

PT13: for some children it had teasing out the behavior versus if their body was tired, can be difficult for even a child to understand, let alone someone who may have a cognitive limitation.

131

00:14:37.900 --> 00:14:50.899

PT13: right? And the parents answer this for the child. Just so. You're clear. The the. I think I think that a. But I think a caregiver might not be able to read that easily in someone who's not and not as who's not ambulatory?

132

00:14:51.450 --> 00:14:52.460

NM: Nonverbal.

133

00:14:52.710 --> 00:14:55.519

NM: Yeah. And nonetheless. Yeah. Okay, sounds good.

134

00:14:55.660 --> 00:14:56.760

NM: Number 2.

135

00:14:56.870 --> 00:15:10.840

NM: How many days did your child exercise really hard for 10 min or more. How would you rate this for a parent to answer this related to a physical activity 0 not related all 5 or up to 5. Highly appropriate.

136

00:15:11.960 --> 00:15:13.809

I think it's highly appropriate.

137

00:15:13.890 --> 00:15:17.080

PT13: because I think you can see when you're when someone's working really hard.

138

00:15:20.900 --> 00:15:26.260

NM: Okay, and number 3. How many days is your child exercise so much that he or she breathes heart?

139

00:15:26.630 --> 00:15:31.730

NM: How would you rate this? This? This question 0 not related up to 5 highly appropriate.

140

00:15:38.350 --> 00:15:42.100

PT13: probably a 4. I think it's mostly relatable.

141

00:15:44.350 --> 00:15:44.930

Okay?

142

00:15:45.230 --> 00:15:45.960

NM: Why.

143

00:15:47.630 --> 00:15:53.109

PT13: I think that also that breathing hard is something that you can physically see it, no matter what the child's

144

00:15:53.170 --> 00:15:54.290

PT13: abilities are.

145

00:15:55.620 --> 00:15:56.430

Okay.

146

00:15:58.970 --> 00:16:00.619

NM: all right. Number 4.

147

00:16:00.880 --> 00:16:04.889

NM: How many days was your child so physically active that he or she sweated

148

00:16:05.020 --> 00:16:09.820

NM: 0, not related at all. Not appropriate for this population. 5 highly appropriate.

149

00:16:11.470 --> 00:16:20.310

PT13: I think it's probably a a 0 or a one, just because I I think that your ability to sweat is altered with kids with.

150

00:16:20.950 --> 00:16:22.779

PT13: from my experience what I've seen.

151

00:16:22.900 --> 00:16:25.279

PT13: I don't know that every kid sweats

152

00:16:25.540 --> 00:16:28.610

PT13: as an indication of being physically active.

153

00:16:29.120 --> 00:16:31.629

NM: Yeah, I I I I definitely

154

00:16:32.390 --> 00:16:35.479

NM: here you on that. So give me a number 0 or one

155

00:16:38.870 --> 00:16:42.420

PT13: for it, because it's for children who are nonambulatory, Correct?

156

00:16:43.020 --> 00:16:44.579

PT13: I'm gonna go with 0.

157

00:16:44.640 --> 00:16:45.460

NM: Okay.

158

00:16:46.940 --> 00:16:48.160

NM: Number 5.

159

00:16:50.980 --> 00:17:06.910

NM: How many days did you your child exercise or place so hard that his or her muscles burned, and I remember the parents answer this, and they would they would score. How many days in the past 7 days? So 0 not really not related 5 highly appropriate. How would you rate this question

160

00:17:10.920 --> 00:17:18.180

PT13: again? I think one. I think there's most, I think, one, because it I don't know that there is. I don't know that for that population that

161

00:17:18.300 --> 00:17:19.349

PT13: you're gonna get

162

00:17:19.609 --> 00:17:26.170

PT13: that understanding of. You know that a parent could even know if their child's muscles were burning.

163

00:17:28.730 --> 00:17:34.009

PT13: or that the child would be able to relay that to the parent that to to know. Do I ask that question?

164

00:17:34.300 --> 00:17:34.990

NM: Right?

165

00:17:36.680 --> 00:17:37.310

Okay.

166

00:17:37.350 --> 00:17:38.450

NM: Number 6.

167

00:17:38.850 --> 00:17:45.610

NM: How many days. Did your child exercise or play so hard that he or she felt tired? 0 not related at all. 5

168

00:17:45.630 --> 00:17:48.959

NM: highly appropriate. What number between 0 and 5 would you give.

169

00:17:50.960 --> 00:18:07.069

PT13: I think, a 4? I think that that's a more appropriate question. I think that you can see that a parent could easily see if their child came home, and then, you know, was was able to fall asleep faster, or they were more tired or acted more lethargic. I think that's something that apparently easily see

170

00:18:10.650 --> 00:18:16.840

NM: Number 7 is how many days was your child physically active for 10 min or more? 0. Not appropriate?

171

00:18:16.990 --> 00:18:20.129

NM: 5 highly appropriate. How would you rate this one? And why?

172

00:18:25.360 --> 00:18:33.360

PT13: I think I would read it a 4 only because a parent may not always know what their child is doing during the day

173

00:18:33.450 --> 00:18:39.299

PT13: to know whether or not they were actually physically active for 10 min or just making it a function.

174

00:18:43.970 --> 00:18:44.820

NM: Okay.

175

00:18:46.640 --> 00:18:48.230

NM: Number 8.

176

00:18:49.050 --> 00:18:56.740

NM: How many days did your child run for 10 min or more? 0 not appropriate at all? 5 highly appropriate? How would you rate this question? And why?

177

00:18:57.350 --> 00:19:01.439

PT13: I think it's 0, because we were talking about low-level kids who are not able to.

178

00:19:05.520 --> 00:19:07.520

NM: And as we wrap up.

179

00:19:07.640 --> 00:19:12.199

NM: i'm asking every therapist to give me if they have any final thoughts or any

180

00:19:12.350 --> 00:19:16.370

NM: closing remarks about physical activity in this population.

181

00:19:16.640 --> 00:19:19.020

NM: please feel free to share. Now.

182

00:19:20.400 --> 00:19:24.970

PT13: I I think that the the challenge for the population is that.

183

00:19:25.210 --> 00:19:37.650

PT13: finding different activities that are meaningful for them to be able to be physically active, and then providing that setting in the environment for them to get on the floor and to be able to roll around, or

184

00:19:37.660 --> 00:19:49.439

PT13: you know the space, the caregivers or teachers, if it's in school to be able to that, are willing and able to lift them and put them on the ground, or put them back in their chairs to give them this

185

00:19:49.530 --> 00:19:56.689

PT13: more non traditional environment to really be physically active and really think out of the box. I think there's so many demands on

186

00:19:57.060 --> 00:20:01.050

PT13: teachers and therapists in different settings that

187

00:20:01.120 --> 00:20:05.879

PT13: especially in schools that don't allow for that free thinking, or like a more

188

00:20:06.090 --> 00:20:08.029

PT13: exploratory model. I guess

189

00:20:08.250 --> 00:20:09.780

PT13: something of that nature.

190

00:20:12.890 --> 00:20:14.049

NM: Yeah, that's good.

191

00:20:14.760 --> 00:20:23.629

NM: Okay. So you you just to kind of summarize you get that? It's important for this population to have a meaningful movement.

192

00:20:23.710 --> 00:20:26.430

NM: but it requires a space the the

193

00:20:26.690 --> 00:20:29.720

NM: personnel to allow for this even to occur

194

00:20:30.080 --> 00:20:34.000

NM: to allow that that more of an exploratory models. Okay?

195

00:20:35.060 --> 00:20:41.709

NM: Well, that's very enlightening. Thank you for sharing your final thoughts. I'm going to stop our recording.

196

00:20:41.940 --> 00:20:43.600

NM: Thank you so much for your time.

197

00:20:43.900 --> 00:20:44.570

PT13: Yeah.
