# Supplementary material for: Validity of PROMIS® Pediatric Physical Activity Parent Proxy Short Form Scale as a Physical Activity Measure for Children with Cerebral Palsy Who Are Non-Ambulatory
Source: Behav Sci (Basel). 2025 Jul 31;15(8):1042. doi: 10.3390/bs15081042 (PMC12382615; doi:10.3390/bs15081042)
Supplement: Supplementary file 1 [file behavsci-15-01042-s001.zip › Transcripts copy/PT transcripts - deidentified/PT18.docx]

WEBVTT

1

00:00:00.930 --> 00:00:16.019

NM: All right. Thank you so much for joining us today. We're going to talk about physical activity for children with Cp. That are functioning at GM. Message levels 4 and 5, and on the first half I'm going to ask you a few questions. So it's totally.

2

00:00:16.030 --> 00:00:29.070

NM: We welcome all of your thoughts and all your ideas, because we're learning from you there's no right or wrong Answer. The second half is going to be more so on me showing you a survey of the promise develop developed by the Nih.

3

00:00:29.120 --> 00:00:50.730

NM: and so i'll ask you about that as we get to the end. And I did want to say, I do have a bit of a script, so forgive me if I sound scripted, because i'm trying to be as consistent as possible. Okay, so again for joining us today. The first question is, how do you define physical activity for children with Cp. Who are not full time walkers?

4

00:00:53.710 --> 00:01:01.410

PT18: I basically what we would try to do is just any physical activity that they can do.

5

00:01:01.430 --> 00:01:05.880

PT18: And typically we're gonna work them

6

00:01:06.260 --> 00:01:09.860

PT18: Hate to say alot of them. We may work them to their almost

7

00:01:10.250 --> 00:01:11.400

PT18: out of breath.

8

00:01:11.670 --> 00:01:15.679

PT18: But as far as how would I define physical activity, any

9

00:01:15.740 --> 00:01:17.910

PT18: any movement that they have.

10

00:01:18.100 --> 00:01:24.479

PT18: any ability that they can assist, participate during transfers or transitions.

11

00:01:24.610 --> 00:01:28.739

PT18: and we may do some like therapeutic walking

12

00:01:28.910 --> 00:01:34.800

PT18: in a gait trainer, or riding an adapted bike. But it may also be assisted.

13

00:01:38.100 --> 00:01:47.970

NM: Great. Thank you. The department of Health defines physical activity as any activity that encompasses energy expended, and activation of skeletal muscle.

14

00:01:48.000 --> 00:01:51.999

NM: Does this definition. Change your mind about how you define physical activity.

15

00:01:52.210 --> 00:01:54.610

PT18: No, I should have been more biological.

16

00:01:54.880 --> 00:01:59.300

PT18: Yeah, that that that sounds fine.

17

00:01:59.660 --> 00:02:03.679

NM: You did great. And how do you think physical activity

18

00:02:04.800 --> 00:02:08.360

NM: differs from other types of fitness activities?

19

00:02:14.390 --> 00:02:15.350

PT18: Hmm.

20

00:02:16.270 --> 00:02:16.910

PT18: Yeah.

21

00:02:18.500 --> 00:02:25.970

PT18: You know, you know it's funny. I I I actually I don't. I just think we need to have our kids

22

00:02:26.590 --> 00:02:29.659

PT18: do whatever aerobic activity they can.

23

00:02:29.900 --> 00:02:31.080

PT18: and I I wouldn't

24

00:02:31.240 --> 00:02:40.019

PT18: discern a physical activity from a fitness activity, I think, for some of the guys that are working with

25

00:02:40.740 --> 00:02:47.189

PT18: even like after a summer they may come back, and they may not have the energy to even be upright in a stander.

26

00:02:48.060 --> 00:02:49.329

PT18: So I

27

00:02:49.380 --> 00:02:58.299

PT18: it's kind of a I don't. I don't know I I don't know if I would just start diagnosis this as 2 different things, physical activity, physical fitness.

28

00:02:58.550 --> 00:03:02.190

PT18: because I look at it as they need to to be

29

00:03:02.540 --> 00:03:09.180

PT18: enough to do the activities that are important to them and their family.

30

00:03:10.180 --> 00:03:18.909

NM: That makes sense. Yes, thank you. It's great. When do you witness Your students participate most in physical activity during the school day?

31

00:03:21.810 --> 00:03:25.900

PT18: Probably mostly, unfortunately, during physical therapy.

32

00:03:27.370 --> 00:03:28.020

Okay.

33

00:03:30.020 --> 00:03:37.149

we really do try and encourage the classrooms to do some of it in addition.

34

00:03:37.280 --> 00:03:40.639

PT18: But i'll say the most that I see is.

35

00:03:41.380 --> 00:03:45.140

PT18: you know. In the classroom they don't a lot of out of chair positioning.

36

00:03:46.790 --> 00:03:50.960

PT18: but as far as direct physical activity, I would say it's during a

37

00:03:51.770 --> 00:03:53.679

PT18: there are treatment Sessions.

38

00:03:54.280 --> 00:03:56.460

NM: Okay, great. Thank you. Next question.

39

00:03:56.620 --> 00:04:02.800

NM: How do you measure physical activity, frequency, intensity, time and type

40

00:04:02.920 --> 00:04:14.180

NM: and children with Cp. Who are not full time. Walker. So that's like the fitt principal kind of quoting that. But it can. You can describe it how you like so frequency, intensity, time, and type.

41

00:04:15.390 --> 00:04:16.690

NM: How would you measure that

42

00:04:16.740 --> 00:04:17.929

NM: in these kids?

43

00:04:19.820 --> 00:04:24.930

PT18: I? You know what I hate to say. It's going to be so individualized like. There are

44

00:04:25.250 --> 00:04:28.990

PT18: very few, though, in our school, that we may even do things like

45

00:04:29.510 --> 00:04:33.659

how many steps or how far they move.

46

00:04:33.810 --> 00:04:37.470

PT18: and like a 4 min time trial.

47

00:04:40.030 --> 00:04:40.850

PT18: but.

48

00:04:41.860 --> 00:04:55.940

PT18: because of our all goals are individualized. I think i'm actually doing them according to their IEP goals, and that's the physical activity that i'm looking for, and they for each child. It's going to be different.

49

00:04:56.690 --> 00:05:06.550

PT18: It may even just be a physical activity that they're going to. Weight bear on their legs for a an assisted stand pivot transfer. We're going to increase the duration.

50

00:05:07.100 --> 00:05:09.699

PT18: You know what I mean. I may. It may be that they're starting at

51

00:05:09.870 --> 00:05:14.540

PT18: 2 seconds. We need them to sustain consistently for 10 s.

52

00:05:15.080 --> 00:05:17.220

PT18: or maybe

53

00:05:17.240 --> 00:05:27.399

PT18: how many steps are taken in the gait trainer may go consecutively, make a total. They go how far individually they can advance the gait trainer on their own.

54

00:05:27.490 --> 00:05:32.459

PT18: You know what I mean, but I feel like each child is going to be different

55

00:05:32.880 --> 00:05:34.189

PT18: as far as that

56

00:05:34.330 --> 00:05:37.689

PT18: that goes, we may use some standardized testing.

57

00:05:38.030 --> 00:05:40.810

PT18: but when I'm actually measuring more.

58

00:05:40.940 --> 00:05:44.539

PT18: I would say, we are doing it more through the

59

00:05:45.540 --> 00:05:55.489

PT18: the student goals and these goals we have to work on, save, the data for each market period. So there's going to be 4 marketing periods during school year.

60

00:05:55.550 --> 00:05:56.320

Hmm.

61

00:05:56.410 --> 00:05:58.680

PT18: And that is the

62

00:05:59.810 --> 00:06:10.109

PT18: and for any in the physical therapy thing it's always going to be an active home. It may, it may be just that, keeping the head up right.

63

00:06:10.710 --> 00:06:13.009

PT18: It may be participating.

64

00:06:13.940 --> 00:06:16.709

PT18: And a righting reaction [postural control].

65

00:06:17.190 --> 00:06:21.040

PT18: I mean it's it's always going to be kind of different now, I think.

66

00:06:23.280 --> 00:06:27.530

PT18: probably giving you tough answers.

67

00:06:28.090 --> 00:06:42.429

NM: and you gave some examples to you already, answered my follow up. Which was, do they need assistance completing these activities? And you gave so many great examples, so do you give them part? Is assistance, or you know total systems, or

68

00:06:42.480 --> 00:06:51.519

PT18: you know what it it it really depends. And sometimes i'm even just looking for them to initiate.

69

00:06:51.660 --> 00:07:04.619

PT18: Hmm. Initiated movement. I can. I'm just thinking like I have a child, that oh, my goodness! He doesn't follow any commands. But if I put him sideline, he will initiate rolling to his back.

70

00:07:05.340 --> 00:07:06.490

PT18: and and

71

00:07:06.850 --> 00:07:10.039

PT18: that's what i'm looking at like that. Will he initiate

72

00:07:12.180 --> 00:07:14.579

that with that movement.

73

00:07:15.160 --> 00:07:30.900

PT18: and and you're right. A lot of terms it is. I found it was challenging for me to write goals that change the the child's level of participation from mid to moderate to Max, because it seemed like it was subjective as somebody else was

74

00:07:31.910 --> 00:07:34.020

PT18: grading that

75

00:07:34.560 --> 00:07:40.699

PT18: so we may even write a goal like a child, will participate in a pull to sit transfer.

76

00:07:43.690 --> 00:07:47.550

PT18: I may put on something like that performing.

77

00:07:48.120 --> 00:07:56.970

PT18: We, you know, acquiring minimal assist They will do 75 % of the activity, or I've even had it, that they will

78

00:07:57.010 --> 00:08:05.449

PT18: participate in a pull to sit transfer throughout the whole transition, because I have some students that they initiate.

79

00:08:05.550 --> 00:08:08.560

PT18: and then they just stop. They let you complete

80

00:08:09.380 --> 00:08:10.659

PT18: the transition.

81

00:08:12.490 --> 00:08:19.690

PT18: So. But you're right. I some of these are not independent. It is being done with some manual assistance. Yes.

82

00:08:20.100 --> 00:08:20.930

okay.

83

00:08:21.010 --> 00:08:22.010

NM: thank you.

84

00:08:22.480 --> 00:08:23.230

PT18: It's great.

85

00:08:23.310 --> 00:08:28.999

NM: Do you think they should participate in more or less of the activities you talked about? And why?

86

00:08:32.970 --> 00:08:41.270

PT18: Obviously, I would like that they participate more. But the challenge is for some of these students. It's more that I

87

00:08:41.429 --> 00:08:46.159

PT18: you know. You know i'm working with all GMFCS levels.

88

00:08:46.580 --> 00:08:51.680

PT18: You know Cs is level 4 and 5, some that don’t follow commands.

89

00:08:51.800 --> 00:08:52.540

PT18: and

90

00:08:52.810 --> 00:09:01.040

PT18: well kind of just look for some more consistency, too, like I may sporadically see something that is it right?

91

00:09:01.490 --> 00:09:02.859

And it's not that.

92

00:09:03.280 --> 00:09:09.820

PT18: Yes, I would like them to do more, but if they got to be more consistently consistent at what they did.

93

00:09:09.840 --> 00:09:12.310

PT18: it would even be so helpful for the caregiver.

94

00:09:13.120 --> 00:09:14.249

PT18: Does that make sense?

95

00:09:14.530 --> 00:09:16.889

PT18: Yes.

96

00:09:18.010 --> 00:09:27.530

NM: all right. There are question. Do you address promoting physical activity during your Pt. Session, and if yes, how do you do this?

97

00:09:29.380 --> 00:09:32.630

PT18: Yes, I I would say, i'm always

98

00:09:33.000 --> 00:09:34.080

PT18: trying to get.

99

00:09:34.210 --> 00:09:41.379

PT18: not always because we do do some power mobility. So if i'm not stressing power mobility at the time.

100

00:09:41.680 --> 00:09:44.940

PT18: Yes, I am going to see whatever active.

101

00:09:46.070 --> 00:09:49.259

PT18: whatever way they can be active

102

00:09:49.780 --> 00:09:55.120

PT18: during their therapy time, and, as I said, we use a lot of swings.

103

00:09:55.250 --> 00:09:55.970

PT18: Alright.

104

00:09:56.000 --> 00:10:03.610

PT18: you know mobile Surfaces gait trainers. We do have a treadmill here. We do have adaptive bikes.

105

00:10:03.890 --> 00:10:04.710

PT18: and typically

106

00:10:05.740 --> 00:10:11.760

PT18: our sessions are 45 min. I'm not going to keep them on one activity the whole 45 min.

107

00:10:12.220 --> 00:10:15.149

PT18: But I may start with that activity.

108

00:10:17.090 --> 00:10:18.839

PT18: Try to get as much

109

00:10:18.940 --> 00:10:21.500

PT18: active participation as we can.

110

00:10:21.840 --> 00:10:25.910

PT18: I don't want to just passively push somebody on a bike.

111

00:10:26.100 --> 00:10:27.789

PT18: We don't want to

112

00:10:27.950 --> 00:10:30.909

PT18: have someone up in there at a gait trainer and work.

113

00:10:31.070 --> 00:10:33.450

PT18: you know, moving their legs on the treadmill.

114

00:10:33.560 --> 00:10:38.359

PT18: Som times We may do that then we would also put them over ground, too.

115

00:10:38.480 --> 00:10:46.250

PT18: You know what I mean, just trying to get to step in over the treadmill, and then go for over ground. Also to see if we're getting any carry-over

116

00:10:46.280 --> 00:10:47.119

PT18: like that.

117

00:10:47.530 --> 00:10:48.630

PT18: Sure.

118

00:10:52.700 --> 00:10:59.600

NM: And what components of physical activity do you feel like you're addressing? Let me give you an example Like.

119

00:10:59.640 --> 00:11:04.400

NM: Are you dressing cardiovascular endurance, mobility, muscle, activation.

120

00:11:04.440 --> 00:11:11.069

NM: energy, expenditure? What do you feel like your what component of physical activity Are you hitting it most of the time during your sessions.

121

00:11:14.040 --> 00:11:25.850

PT18: I think we're looking mainly for muscle activation. But definitely, I will say, like I do have students that you know they'll pedal in a burst

122

00:11:26.180 --> 00:11:34.530

PT18: in the on the bike, and it is definitely cardiovascular. They pedal, you know. They might go through like 20 revolutions

123

00:11:34.750 --> 00:11:36.570

PT18: have to stop and catch the breath.

124

00:11:36.710 --> 00:11:47.390

PT18: Okay, and then do it again. Do it again. The same thing with stepping in a gait trainer, some of them. That is what's limiting them. They don't really have the cardiovascular endurance

125

00:11:47.560 --> 00:11:52.680

PT18: to continue so. They will go, give it their all.

126

00:11:52.860 --> 00:12:02.210

PT18: stop, take a breather, give it their all, stop take a breather. Have a lot of students that do seem to work in that kind burst type of mode

127

00:12:04.360 --> 00:12:05.840

PT18: mode

128

00:12:05.880 --> 00:12:07.010

NM: Yeah, yeah.

129

00:12:08.700 --> 00:12:13.039

NM: Great. All right, and that that was the example by some cardiovascular

130

00:12:13.240 --> 00:12:16.070

NM: challenging. Now, right now, that's good.

131

00:12:16.100 --> 00:12:23.859

NM: all right. And fourth question. Do you address promoting physical activity that occurs outside of Pt sessions.

132

00:12:24.660 --> 00:12:28.340

PT18: Yes, and that is more. We're doing a lot of equipment based.

133

00:12:28.450 --> 00:12:34.309

PT18: We try to have. Each of the families have a stander

134

00:12:34.390 --> 00:12:35.939

PT18: at home

135

00:12:36.160 --> 00:12:38.579

PT18: and a gait trainer if possible.

136

00:12:38.840 --> 00:12:41.380

PT18: and we try to connect

137

00:12:41.470 --> 00:12:45.340

PT18: as much as we can with us funding sources for adaptive bikes.

138

00:12:45.600 --> 00:12:51.100

It it is a challenge. Some families are definitely more involved than others, and that's

139

00:12:52.350 --> 00:12:57.209

PT18: And I yeah, yeah, I understand the challenge of the time commitment.

140

00:12:57.840 --> 00:13:02.789

PT18: But out of those 3. We really try to push for the stander

141

00:13:03.440 --> 00:13:06.660

PT18: if they can. At least, you know, to the standing at home.

142

00:13:08.220 --> 00:13:10.750

PT18: You know we do do standing here.

143

00:13:11.070 --> 00:13:17.689

PT18: but it we're school, and obviously this weekend some longer holidays. Summer time.

144

00:13:19.140 --> 00:13:19.900

NM: Gotcha.

145

00:13:19.940 --> 00:13:24.489

PT18: So it's a lot of a lot of that. I feel like it's like equipment based.

146

00:13:25.150 --> 00:13:29.759

PT18: if that makes sense. But I try to make sure that the family has the equipment they need at home

147

00:13:30.480 --> 00:13:32.190

PT18: to continue with the program

148

00:13:32.790 --> 00:13:39.460

PT18: got you, and and we've been using a lot of that, hey? The rifton E pacer, or because you can use it also as transfer.

149

00:13:39.520 --> 00:13:44.620

PT18: transfer aid when you have the you know the leg slings.

150

00:13:44.640 --> 00:13:45.820

PT18: and

151

00:13:45.870 --> 00:13:56.029

PT18: you know I can just think of 2 families that we have that love putting their child in that for the stepping part, but also use it a lot for the transfers.

152

00:13:57.220 --> 00:13:58.010

PT18: So

153

00:13:58.980 --> 00:14:00.530

NM: okay, it's not.

154

00:14:00.610 --> 00:14:08.450

NM: And what have you recommended any community programs or events to your students to help increase physical activity.

155

00:14:11.510 --> 00:14:13.309

PT18: You know what I feel.

156

00:14:14.320 --> 00:14:15.480

PT18: our rec

157

00:14:15.560 --> 00:14:23.850

PT18: We're spoiled by by that. They do a lot of that, you know. We will recommend the adaptive skiing.

158

00:14:24.460 --> 00:14:33.060

PT18: The adaptive soccer is in this area. The flyers have a great adaptive

159

00:14:33.680 --> 00:14:35.170

PT18: hockey program.

160

00:14:35.850 --> 00:14:44.210

PT18: around here. But I will say, sad is i'm not doing the recommendation I will talk to. We have a rec...

161

00:14:45.040 --> 00:14:57.160

PT18: you know, a recreation therapy department that does those kind of recommendations, and we have a lot of the kids that are doing, you know, baseball soccer, hockey.

162

00:14:57.250 --> 00:15:02.069

PT18: The adaptive skiing is, you know, pretty much just like that.

163

00:15:02.090 --> 00:15:04.830

PT18: one or 2 common thing.

164

00:15:06.570 --> 00:15:13.560

PT18: and unfortunately, our local swimming program, they they haven't resummed since Covid.

165

00:15:13.790 --> 00:15:16.200

PT18: Yeah. So

166

00:15:18.810 --> 00:15:19.629

PT18: yeah.

167

00:15:20.640 --> 00:15:31.370

NM: And you already kind of answered this. They'll ask it again. What type of equipment have you recommended to help you prove home and or community engagement and physical activity outside

168

00:15:31.590 --> 00:15:44.500

PT18: of the clinical setting?

169

00:15:44.760 --> 00:15:50.940

PT18: You know what I mean, Like we, I I think, since I've been a therapist I've only got one

170

00:15:52.440 --> 00:15:58.319

PT18: and one through insurance and one through a waiver program, and it was the Waiver program did it because it was therapeutic

171

00:15:58.700 --> 00:16:00.619

conditioning.

172

00:16:00.960 --> 00:16:06.799

NM: You know. We went through the whole aerobic cardiovascular, and they did pay for it.

173

00:16:06.960 --> 00:16:09.220

PT18: typically, though

174

00:16:09.360 --> 00:16:10.489

PT18: you know what I

175

00:16:10.770 --> 00:16:14.160

PT18: It's a challenge with the funding. But if all possible.

176

00:16:14.400 --> 00:16:15.920

PT18: definitely try to get that.

177

00:16:17.460 --> 00:16:19.350

NM: Yeah.

178

00:16:19.580 --> 00:16:32.279

PT18: yeah, and and and the standers, some of them want to static we do. The mobile standards seem to take up a little less space in the home, and if they have the trunk control that they can use the mobile.

179

00:16:32.490 --> 00:16:33.810

PT18: We'll go that way

180

00:16:34.440 --> 00:16:44.330

NM: a little more space.

PT18: The the Rifton mobile standard seems to take up a little less space.

181

00:16:44.650 --> 00:16:51.720

PT18: but but unfortunately it's definitely a challenge because you don't get the trunk supports. We do a lot of the easy stands and bantams.

182

00:16:52.370 --> 00:16:55.930

PT18: you know. We can.

183

00:16:55.960 --> 00:16:58.669

NM: Yeah, the bantams are great yeah.

184

00:17:03.150 --> 00:17:07.749

NM: All right, now we are at the second half. I'll go ahead and show you this this survey

185

00:17:08.260 --> 00:17:11.889

again. This was developed by the National Institute of Hell.

186

00:17:12.339 --> 00:17:19.060

NM: and i'm gonna let you look at it for a second. So just so, you see, this is a parent proxy survey. So the parent answers, the child is not

187

00:17:19.280 --> 00:17:24.140

NM: about what they believe. Their child's level of physical activity has been

188

00:17:24.500 --> 00:17:35.070

NM: for the previous week. Okay, and they will if you. They. This was given out, but I really am looking to see what we think is Pts

189

00:17:35.300 --> 00:17:49.169

NM: about how this really is appropriate for the kids we've been talking about so far, alright, so i'm gonna i'm gonna tell you. I'm gonna read the question. And i'm gonna ask you after each question. Want to scale from 0, not related at all

190

00:17:49.460 --> 00:17:54.300

NM: up to 5 highly appropriate how you would rate this question and walk

191

00:17:54.880 --> 00:17:55.899

NM: It's not good.

192

00:17:57.090 --> 00:18:03.969

NM: Alright, First question: how many dates your child exercise or play so hard that his or her body got tired.

193

00:18:05.610 --> 00:18:09.370

NM: 5 highly appropriate, or somewhere in between.

194

00:18:09.740 --> 00:18:11.940

PT18: No, I think that's highly appropriate.

195

00:18:12.150 --> 00:18:12.870

NM: Okay.

196

00:18:12.970 --> 00:18:14.010

NM: because

197

00:18:14.780 --> 00:18:19.080

PT18: I think it's an easy one for the family to answer.

198

00:18:19.430 --> 00:18:24.010

PT18: And I like I can just think of kids that

199

00:18:24.040 --> 00:18:25.349

PT18: even if they're just

200

00:18:25.760 --> 00:18:29.490

PT18: on the floor, rolling and laughing that org

201

00:18:29.700 --> 00:18:31.010

PT18: bye.

202

00:18:31.360 --> 00:18:35.449

PT18: You know that I I I like it. I I think it's as I said.

203

00:18:36.040 --> 00:18:38.870

PT18: it's not complicated for the family to answer that.

204

00:18:39.710 --> 00:18:41.750

NM: Okay? Great. What number would that be?

205

00:18:41.850 --> 00:18:43.510

PT18: I would give that a 5.

206

00:18:43.590 --> 00:18:54.259

NM: Okay, Great number 2. How many days is your child exercise really hard for 10 min or more 0 not appropriate at all up to 5 highly appropriate, and why

207

00:18:57.880 --> 00:19:04.040

PT18: I still may give that a my concern like I know that some of the families will just even just like.

208

00:19:05.140 --> 00:19:13.359

PT18: stand a child we support like at a table or just we have a few that have to upsee.

209

00:19:13.460 --> 00:19:18.490

NM: Hmm. Oh, you know the upsey. Oh, my God, okay, i'm sorry we stayed on

210

00:19:18.620 --> 00:19:22.490

NM: all right, so

211

00:19:24.270 --> 00:19:31.430

PT18: and I I like that, because when I think about. A lot of the kids might really exercise for like 5 min.

212

00:19:31.630 --> 00:19:43.720

PT18: That was my only question, as far as time you know what I mean, and then they have to really relax. But i'm okay. With that question, too. Again, I think it's easy for the family to answer. And

213

00:19:45.540 --> 00:19:47.030

PT18: yeah, I I

214

00:19:47.210 --> 00:19:49.210

PT18: I'm: okay with that one, too.

215

00:19:49.860 --> 00:19:52.970

PT18: So whatever I I give it a font.

216

00:19:53.130 --> 00:19:53.780

NM: Okay.

217

00:19:54.130 --> 00:19:55.450

NM: Number 3.

218

00:19:56.060 --> 00:20:01.789

NM: How many days is your child exercise so much that he or she breathe hard.

219

00:20:01.930 --> 00:20:05.210

NM: 5 is highly reliable.

220

00:20:05.540 --> 00:20:20.420

PT18: I I I like it because that's the other thing. A lot of families like they may put them up in a standard. But truth for the child may not be free and hard. You know what I mean. Like it's not an aerobic type thing, and they're like. That's enough. So now I I like to.

221

00:20:20.960 --> 00:20:25.069

PT18: I I'd say, still fine that it

222

00:20:29.850 --> 00:20:31.599

NM: and number 4.

223

00:20:32.170 --> 00:20:40.950

NM: How many days was your child so physically active that he or she sweated. How would you rate that 1 0 Not appropriate at all? 5 highly

224

00:20:43.370 --> 00:20:48.939

PT18: I once more of a challenge for me. I might go more with. I think of 4.

225

00:20:49.500 --> 00:20:56.469

PT18: I I think what gets hard on this one is it's definitely going to depend obviously on the weather outside.

226

00:20:56.520 --> 00:20:58.690

And

227

00:21:02.210 --> 00:21:03.370

PT18: yeah.

228

00:21:03.640 --> 00:21:08.490

PT18: this this this one is that my little order for me that given it 3 3

229

00:21:08.820 --> 00:21:10.180

NM: and

230

00:21:10.200 --> 00:21:10.980

NM: wow.

231

00:21:14.820 --> 00:21:15.510

PT18: Hey.

232

00:21:16.230 --> 00:21:28.460

PT18: I like having that information, but I don't know if it's how as much about what is going on, because so many of the kids I see have temperature just regulation.

233

00:21:28.560 --> 00:21:29.690

PT18: anyway.

234

00:21:29.930 --> 00:21:33.979

PT18: like we have kids that get so overheated.

235

00:21:35.080 --> 00:21:39.029

you know, like air conditioning is a must in their IP.

236

00:21:39.410 --> 00:21:41.330

PT18: Hmm. So

237

00:21:41.700 --> 00:21:47.450

PT18: I I think it just gets a little confusing because I see kids that it may sweat every day.

238

00:21:47.540 --> 00:21:51.090

PT18: and sometimes, unfortunately, the sweating is, even if there are

239

00:21:51.500 --> 00:21:55.649

PT18: physically uncomfortable crying.

240

00:21:59.000 --> 00:22:03.379

I can't say it's always for my physical activity. Does that make sense?

241

00:22:03.430 --> 00:22:04.580

NM: Yes.

242

00:22:04.680 --> 00:22:06.870

NM: absolutely.

243

00:22:07.060 --> 00:22:10.299

NM: And that's great. So that's number 4 Number 5.

244

00:22:11.140 --> 00:22:18.869

NM: How many days did your child exercise or play so hard that his or her muscles burned 0. Not appropriate.

245

00:22:19.090 --> 00:22:21.539

NM: 500. And how would you rate that one?

246

00:22:21.890 --> 00:22:25.100

PT18: The reason I find that hard is

247

00:22:25.210 --> 00:22:28.200

PT18: you'd have to have a child, and communicate

248

00:22:28.220 --> 00:22:30.200

PT18: that their muscles.

249

00:22:30.220 --> 00:22:32.440

PT18: or that like.

250

00:22:32.570 --> 00:22:38.040

PT18: I can think of some kids who who exercise so much that then they are like shaking.

251

00:22:38.110 --> 00:22:41.259

PT18: You know what I mean. They go into like a spasm.

252

00:22:43.180 --> 00:22:43.780

I

253

00:22:45.140 --> 00:22:50.879

PT18: I think that one's a little harder for me because you're so. It's a subjective thing that appears so

254

00:22:51.160 --> 00:22:56.460

PT18: answering unless the child can answer that for them. So i'm gonna say it too.

255

00:22:56.700 --> 00:22:57.310

NM: Okay.

256

00:22:58.090 --> 00:22:58.950

thank you.

257

00:23:00.230 --> 00:23:03.000

NM: And Number 6.

258

00:23:03.460 --> 00:23:08.600

NM: How many days your child exercise or play so hard that he or she felt tired.

259

00:23:09.500 --> 00:23:13.219

PT18: Alright, so that's different than his body. Not child.

260

00:23:19.500 --> 00:23:23.950

Oh, i'm. Okay, with that one that that one I could still say a bond.

261

00:23:24.280 --> 00:23:25.010

Okay.

262

00:23:26.280 --> 00:23:29.450

PT18: for the same reasons as number one.

263

00:23:34.060 --> 00:23:41.530

NM: Okay, Number 7. How many days did your child? How was your child physically active for 10 min or more.

264

00:23:42.010 --> 00:23:47.949

PT18: Okay. So the difference here is this: one is just physically active. The other one was really hard.

265

00:23:48.860 --> 00:23:50.880

PT18: Yes, okay.

266

00:23:52.230 --> 00:23:56.059

NM: But this one is also physically active for 10 min. Oh, you're right.

267

00:23:56.310 --> 00:24:04.770

NM: That was the main difference. Yup. One that exercise really hard, One says, physically active. The time.

268

00:24:05.160 --> 00:24:07.600

PT18: Yeah, a 5,

269

00:24:09.450 --> 00:24:14.669

NM: because you like you like the the terminology better. What was the reason that you was?

270

00:24:15.730 --> 00:24:17.310

PT18: I do.

271

00:24:17.700 --> 00:24:22.899

PT18: I do like the terminology better that they were physically active for 10 min or more

272

00:24:23.300 --> 00:24:31.770

PT18: problem, and exercising really hard, because sometimes, when you say they're exercising for them, it was very hard

273

00:24:32.110 --> 00:24:34.730

PT18: like. I don't know how it makes it

274

00:24:35.940 --> 00:24:38.710

PT18: less subjective for the parent.

275

00:24:39.350 --> 00:24:40.260

NM: Gotcha.

276

00:24:41.540 --> 00:24:43.839

NM: All right. Number 8.

277

00:24:45.490 --> 00:24:51.820

NM: How many days i'll run for 10 min or more 0 or not. 0.

278

00:24:52.900 --> 00:24:53.930

PT18: Hello!

279

00:24:54.550 --> 00:24:59.710

PT18: I Unfortunately, the children that I say, really they

280

00:25:00.060 --> 00:25:04.840

PT18: they may run in a gate trainer, but it's going to be a

281

00:25:06.840 --> 00:25:09.180

PT18: that you know what I mean. It's it's

282

00:25:09.390 --> 00:25:11.949

PT18: it's it's not going to be the typical

283

00:25:12.450 --> 00:25:17.149

PT18: way that you would imagine someone to run, and I think the question is just confusing because of that.

284

00:25:21.380 --> 00:25:29.450

NM: All right. How last the allow at the end. I like to get last words and final thoughts about physical activity

285

00:25:29.490 --> 00:25:34.489

NM: in this population, would you? Would you do you have any final thoughts you would like to share

286

00:25:34.540 --> 00:25:36.680

for us as we wrap up?

287

00:25:37.870 --> 00:25:43.359

PT18: I do like this. I. I really think that it would be.

288

00:25:44.350 --> 00:25:49.930

PT18: You know it's funny. We've been talking about that. We should be doing some case stories at our facility.

289

00:25:49.990 --> 00:25:52.860

because we may get kids

290

00:25:52.920 --> 00:25:58.189

PT18: when we can think of 2 of them right now, who basically

291

00:25:58.230 --> 00:25:59.299

PT18: we're in it

292

00:25:59.330 --> 00:26:03.209

PT18: school where they didn't really have any equipment

293

00:26:03.550 --> 00:26:07.569

PT18: to try with them, and they basically sign the chair of the whole day.

294

00:26:08.280 --> 00:26:10.070

which is sad.

295

00:26:10.100 --> 00:26:15.309

PT18: And the one child now is getting into a gay trainer

296

00:26:15.430 --> 00:26:18.120

PT18: will take off.

297

00:26:18.170 --> 00:26:32.220

PT18: But yes, do they need help to steer? Yes, but are they moving their legs so fast? You're trying to steer? Why, they're moving it. You know what I mean. Like they don't have the capacity to

298

00:26:32.480 --> 00:26:47.249

PT18: totally steer on their own. They're moving in, and they are doing same thing, and I can't. That's probably one of my biggest choices that we've got a number of students that have never been on a bike, never.

299

00:26:47.360 --> 00:26:52.549

PT18: and they will be so excited. I can tell you this little girl right now that

300

00:26:52.630 --> 00:26:55.929

PT18: Oh, my gosh! The first couple of times I could have tears in my apps.

301

00:26:56.000 --> 00:27:10.459

PT18: you know she would puddle like again. She's not cut on the whole time. She go about like 1215 revolutions. This is she can't stop again, do it again, and be like screaming

302

00:27:10.520 --> 00:27:12.560

PT18: and and left them.

303

00:27:12.620 --> 00:27:14.910

PT18: So I just think

304

00:27:16.080 --> 00:27:28.829

PT18: it would be really nice to have something like this, even for the families to fill out to say, okay, this is the kind of activity that we're doing before now that they've been in this programming.

305

00:27:29.280 --> 00:27:31.679

PT18: This is what we're seeing.

306

00:27:32.210 --> 00:27:34.579

PT18: You know the different opportunities

307

00:27:35.610 --> 00:27:39.460

PT18: that a. And we have another. We that I think unfortunately, he's had

308

00:27:39.580 --> 00:27:41.750

PT18: is hips, or some.

309

00:27:42.660 --> 00:27:54.559

PT18: you know, not really aligned it. I think they were even nervous about even stand them, and with the right supports you can't stand. He can't get in the gate trainer, and he's just so excited to be an upfront.

310

00:27:55.100 --> 00:27:58.879

PT18: you know. He may not be stepping, but he is up right.

311

00:27:59.570 --> 00:28:01.670

PT18: and just a different

312

00:28:01.760 --> 00:28:07.639

PT18: view of our surroundings. And you know, oh, my goodness for bone today.

313

00:28:08.050 --> 00:28:08.900

you know.

314

00:28:09.440 --> 00:28:10.410

PT18: Keep in

315

00:28:10.660 --> 00:28:14.850

PT18: this pouse regular.

316

00:28:15.640 --> 00:28:22.949

PT18: so I I think we do need to have better ways to monitor what the child

317

00:28:23.110 --> 00:28:26.230

PT18: is doing in their daily life.

318

00:28:26.870 --> 00:28:29.579

PT18: If that makes sense, so that then you could say.

319

00:28:30.580 --> 00:28:36.969

But the only I wish that in this there was some kind of way that it was even talking about.

320

00:28:39.600 --> 00:28:42.570

PT18: Were they doing this with equipment?

321

00:28:44.360 --> 00:28:49.539

PT18: Just so. You have some kind of qualifier, knowing that they can do it with equipment, but

322

00:28:50.310 --> 00:28:51.899

PT18: equipment, not equipment.

323

00:28:53.140 --> 00:28:57.069

PT18: So I just just went on that one. But I I there it

324

00:28:58.600 --> 00:29:04.110

PT18: I I I like it, even if nothing else, to make the family more cognizant like.

325

00:29:04.480 --> 00:29:05.170

PT18: Yeah.

326

00:29:05.410 --> 00:29:07.960

PT18: Oh, my child, should be doing this.

327

00:29:10.290 --> 00:29:16.910

PT18: It's just like a a real realistic expectation for my child and for a lot of it really would be

328

00:29:19.680 --> 00:29:20.500

PT18: So

329

00:29:21.540 --> 00:29:29.399

NM: that's great. I do have one more follow up. You mentioned how, when you're not, you're not working on physical activity when they're in the power wheelchair.

330

00:29:30.760 --> 00:29:40.130

NM: Why, don't you consider that physical activity? I'm: just curious. Actually, I do, I. It really does a lot of next strengthening

331

00:29:40.250 --> 00:29:41.800

PT18: the focus.

332

00:29:43.490 --> 00:29:45.390

PT18: our body strengthening.

333

00:29:45.420 --> 00:29:55.779

PT18: I do actually. And you're right now. But I I I phrase that wrong, because I can even just think we the little boy who had never been in a power chair and

334

00:29:55.890 --> 00:29:57.500

PT18: a joystick driver.

335

00:29:57.590 --> 00:30:15.529

PT18: As soon as you put him in the chair he understood it. There was no doubt about if he could do it, and we colon them a chair. And what we found is, if they teach him too much to be in the power chair all day at school so literally. What we were doing initially was having him in the power chair in the morning

336

00:30:15.840 --> 00:30:18.139

PT18: is manual in the afternoon.

337

00:30:18.440 --> 00:30:22.150

PT18: He could not self propel at all.

338

00:30:22.170 --> 00:30:29.679

PT18: but it was just too physically exhausted for him, and it was interesting how much, how t when it was on his hand

339

00:30:29.960 --> 00:30:32.530

PT18: and his whole

340

00:30:32.870 --> 00:30:34.559

PT18: daddy. Actually.

341

00:30:35.010 --> 00:30:37.800

PT18: So you have a good point. I shouldn't phrase it in that way.

342

00:30:38.060 --> 00:30:44.849

PT18: And now he's a full time power wheelchair driver really independent. Yeah, I e.

343

00:30:44.920 --> 00:30:45.730

PT18: Each

344

00:30:45.860 --> 00:30:48.340

PT18: indoor She really is independent.

345

00:30:49.160 --> 00:30:50.329

PT18: Sorry about that.

346

00:30:50.590 --> 00:31:01.370

NM: Oh, no, it's fine. It is actually good. I don't want to keep you past the time I didn't. I didn't do that appropriate. It's like kind of crazy. But we actually believe it or not. We clock in our in and out here.

347

00:31:01.660 --> 00:31:02.340

Okay.

348

00:31:02.490 --> 00:31:05.719

PT18: I I need an alarm to remind me.
