# Supplementary material for: Validity of PROMIS® Pediatric Physical Activity Parent Proxy Short Form Scale as a Physical Activity Measure for Children with Cerebral Palsy Who Are Non-Ambulatory
Source: Behav Sci (Basel). 2025 Jul 31;15(8):1042. doi: 10.3390/bs15081042 (PMC12382615; doi:10.3390/bs15081042)
Supplement: Supplementary file 1 [file behavsci-15-01042-s001.zip › Transcripts copy/PT transcripts - deidentified/PT4.docx]

WEBVTT

1

00:00:00.000 --> 00:00:13.200

PT4: Hi! So let's start recording. Hello! Thank you. Hello! Well, let let me pull you back up. Do you see me or no? Did I I do see you. Did you see me? Okay, let me pull you back up. Okay.

2

00:00:13.260 --> 00:00:14.510

NM: So

3

00:00:14.670 --> 00:00:20.799

NM: Dr. PT4, Thank you for joining us today. We are going to start with looking at

4

00:00:23.130 --> 00:00:31.560

NM: Some questions I have for you. So this is a an interview, and we're going to start with some general questions, and then i'm going to actually pull up a

5

00:00:31.700 --> 00:00:40.110

NM: nih survey that. I'm going to ask you some questions about and see which your thoughts are just as your from your experience the Pt. And just get some feelings about

6

00:00:40.170 --> 00:00:48.789

NM: how you think that assessment may be beneficial to children with Cp. Who are not ambulatory. Okay. So our my first question to you is.

7

00:00:49.360 --> 00:00:53.389

NM: how do you define physical activity for children with Cp.

8

00:00:53.530 --> 00:00:56.600

NM: Who are not full time walkers.

9

00:00:58.950 --> 00:01:18.649

PT4: So I would describe. Sorry. Okay, sweetheart, you have to go eat your breakfast. Okay. So I would describe physical activity really needs to be gauged by the caretaker and or the parents in a structured type of a setting such as Whether or not they're in the standing frame.

10

00:01:18.660 --> 00:01:35.929

PT4: what what type of therapies or activities they have coming into the home. So usually, if my children, who have Cp. Who are non ambulatory, I try to give the parents a structured setting of a half hour to an hour duration 2 or 3 times of a day in their standing frame

11

00:01:36.230 --> 00:01:44.330

PT4: give them activities of rolling activities, sitting activities. you know it's 15 to 20 min intervals depending on

12

00:01:45.020 --> 00:01:50.659

PT4: who, of course, who the caretaker is in the home? If that child is at home.

13

00:01:51.080 --> 00:01:55.209

PT4: you know, throughout the day so majority of the children that I service

14

00:01:55.460 --> 00:02:01.610

PT4: They're under the age of 3, so they're usually not yet in a program, and so they are home all day.

15

00:02:01.710 --> 00:02:11.939

PT4: Many of those children also will have a home health attendant. That's there that is very helpful in if the parents is out to work helpful in having the child

16

00:02:12.030 --> 00:02:15.759

PT4: be able to do these routine type of activities.

17

00:02:16.510 --> 00:02:22.090

PT4: So I usually will give basically a home exercise program to whoever the caretaker is

18

00:02:22.270 --> 00:02:35.690

PT4: of pass range motion exercises like bicycling. you know, play activities. And then maybe a supported ring sit type of a position or a long sit type of position.

19

00:02:36.880 --> 00:02:38.110

PT4: and also

20

00:02:38.750 --> 00:02:46.999

PT4: working with other therapists on the case, you know, will when the let's say the special instructor will come in, or the speech therapist to come in

21

00:02:47.160 --> 00:02:56.160

PT4: They usually will incorporate a lot of the activities of ring sitting, of arm swiping and movements during their

22

00:02:56.320 --> 00:03:11.550

PT4: their sessions. for the children that are not able to have. You know that aren't ambulatory, and that don't have a good control over their body movements. that are more in a dependent type of state.

23

00:03:15.030 --> 00:03:16.200

NM: Awesome.

24

00:03:16.900 --> 00:03:35.289

NM: so I have a I have some follow ups. That was our first question. The first follow up question, for that is so just so you you may you? You know this. The department of Health defines physical activity as any activity that encompasses energy, expenditure and activation of skeletal muscle.

25

00:03:35.320 --> 00:03:39.570

NM: Does this change your mind about how you define physical activity in these kids.

26

00:03:41.380 --> 00:03:47.609

PT4: No, I would say no, because the activities that we just talked about incorporate

27

00:03:48.360 --> 00:03:49.090

PT4: that.

28

00:03:50.240 --> 00:03:55.520

NM: Thank you so much. How do you think physical activity differs from other types of fitness activities?

29

00:03:57.260 --> 00:03:59.810

PT4: How do I think physical activity

30

00:03:59.860 --> 00:04:06.799

PT4: differs from other type of fitness activities? Fitness activities. Hmm.

31

00:04:07.970 --> 00:04:12.010

PT4: I would kind of look at physical activity as an umbrella.

32

00:04:12.440 --> 00:04:14.860

PT4: and under that umbrella I would look at

33

00:04:16.110 --> 00:04:22.639

PT4: sports related, or exercise related activities. Running jogging is kind of subsets.

34

00:04:23.520 --> 00:04:30.339

PT4: So that's how I would do that now. I don't know if I really answered your question with that. Okay.

35

00:04:30.670 --> 00:04:44.739

NM: You said you mentioned of subsets like sports, and then of running, jogging just under…

PT4: dancing gymnastics. So I would look at physical activity, and then under that the umbrella of, and then you would have a subset of, you know, depending on what type of activity

36

00:04:44.820 --> 00:04:49.630

PT4: you know. So like, for for for instance, if we had a child.

37

00:04:49.670 --> 00:04:52.950

PT4: who is very, you know, maybe a

38

00:04:53.600 --> 00:05:08.810

PT4: a a grade 5 you know, even putting him in the pool, and having kind of move around in the pool as a swimming activity would be a subset of, you know, if I would tell the parents. Oh, you need to increase his physical activity, swimming would be a subset of increasing his physical activity.

39

00:05:09.820 --> 00:05:24.630

NM: I understood that's great, thank you PT4. And then the last follow up to. That is where I mean, I know you work typically in AI. So it's not doesn't necessarily the school day or daycare. But when do you witness? Your students participate in most of physical activity during the day?

40

00:05:25.050 --> 00:05:26.889

PT4: I would say morning.

41

00:05:26.980 --> 00:05:34.819

PT4: morning times absolutely. But this subset of 0 to 3 Morning Times is lively usually in the homes.

42

00:05:34.930 --> 00:05:49.030

PT4: the child has been rested overnight. seems to have more energy, more alertness. later on in the day. The child seems to be more tired, more lethargic. not as willing to cooperate.

43

00:05:49.040 --> 00:06:04.810

PT4: Also, you know the stresses on the family and the homes, and the caretakers of other children coming home from school of you know their own tiredness of getting dinner ready, and so I find that the mornings for these type of children seem to be better as far as getting

44

00:06:05.010 --> 00:06:09.239

PT4: activity participation.

45

00:06:09.490 --> 00:06:11.670

PT4: all around from the the child and the family.

46

00:06:14.530 --> 00:06:16.970

NM: Thank you all right. Second question.

47

00:06:17.290 --> 00:06:27.010

NM: How do you measure physical activity, frequency, intensity, time and type, in children with Cp. Who are not full Time Walkers. So that's like the fit principle.

48

00:06:28.880 --> 00:06:41.309

PT4: right? So how do I measure? Can you give me an exact? Is it? Is it okay to give me an example of

NM: yeah, I mean. So this is tough because I I honestly.

49

00:06:42.510 --> 00:06:53.409

NM: I think a lot of the time, and i'll speak for myself. I don't really think I measured it, you know, like if i'm honest, you know, I think we maybe time I maybe I look more so. It's just their endurance

50

00:06:53.470 --> 00:07:03.790

PT4: would be an example or and I hope i'm not asking you your No, no, no, I can agree that No, you could stop talking, and I can, because I just needed. I need a little bit of a lead in, because I didn't quite understand.

51

00:07:04.050 --> 00:07:11.180

PT4: But when you gave me that question that was what I was thinking. I I you know I would time it like. So let's say I had a child

52

00:07:11.550 --> 00:07:22.099

PT4: where I was doing some prone weight bearing maybe on extended arms, and you know I would time how long, maybe, he was able to extend his head or keep the head at neutral. Keep the head at 30 degrees.

53

00:07:22.260 --> 00:07:23.880

PT4: so I I I guess

54

00:07:23.900 --> 00:07:28.390

PT4: right, because you know, it would be a 30 min session we would be doing. But within that session

55

00:07:28.530 --> 00:07:35.939

PT4: I would have it broken down to various exercise, and usually I would look at the duration of how long the child could hold a position.

56

00:07:36.040 --> 00:07:36.980

NM: Yeah.

57

00:07:37.010 --> 00:07:43.549

PT4: And then I would do that multiple times, and usually my first or second time of putting that child in position with were my best times.

58

00:07:43.590 --> 00:07:58.429

PT4: And then, as I got the child more and more into the position. Either the child did not want to be into the position it would fall out of it quicker, or the child just couldn't sustain any more. They were tired. you know the the duration would be longer. So so with some of these children I would look at

59

00:07:58.620 --> 00:08:05.350

PT4: holding a position, and how long they could hold that position for what the duration was. And that's how I kind of would gauge the progress

60

00:08:07.380 --> 00:08:12.979

PT4: Another measurement I would use would be

61

00:08:15.040 --> 00:08:19.540

PT4: I I guess you know it even like sustaining. Let's say sitting balance.

62

00:08:19.740 --> 00:08:22.349

PT4: You know. How how long could they sustain?

63

00:08:22.570 --> 00:08:33.979

PT4: Maybe a modified ring sit, you know, and ha like. So I would kind of time. I would also use time to see how long they would sustain it, or how long they would sustain, maybe holding their head up

64

00:08:34.539 --> 00:08:39.250

PT4: in the midline. You know I one child in in particular

65

00:08:39.590 --> 00:08:50.160

PT4: He had liccencephaly, and microcephaly, and missing Corpus collosum in a handful of other brain abnormalities, and so for him just to be able to hold his head upright

66

00:08:50.620 --> 00:08:55.909

PT4: was an accomplishment that he wanted to, because he wanted to see what was around, so he was very well aware of what he was doing.

67

00:08:55.960 --> 00:09:08.659

PT4: But, it would be more looking at how long he would sustain, how long I could get him to sustain it, for so I would, I guess, timed repetition. So with these children I would look at time repetition positioning

68

00:09:09.530 --> 00:09:12.440

PT4: to see progress

69

00:09:13.110 --> 00:09:14.530

PT4: in their skills.

70

00:09:14.880 --> 00:09:16.020

NM: That's great.

71

00:09:16.150 --> 00:09:29.310

NM: And You said something interesting, and i'm gonna get to that in a minute. I I have my note. But let me do my prompts for a stick to my script. That's the hardest one, because I i'm like, always want to go on. Okay, do you do? They need assistance to complete

72

00:09:29.410 --> 00:09:31.910

NM: these physical activities.

73

00:09:31.950 --> 00:09:46.170

NM: and and what I you mentioned sitting so i'll like we can stick with that if you have any others you want to share. What you talk about biking is standing, so do they need assistance, and then do they need assistance for the entire thing, or are there sometimes where you they Only this is the part of it.

74

00:09:47.330 --> 00:09:52.719

PT4: So yes, usually they, for my fours and 5 they usually need assistance.

75

00:09:52.910 --> 00:09:59.869

PT4: Some Some of the children have been dependent, completely dependent in their care. So

76

00:09:59.990 --> 00:10:19.670

PT4: I would say I could wean my assistance down. For, say, if I do like a reciprocal kind of bicycle type of movement like, say it's more of like a passive range of motion they're laying to supine on their back. and i'm kind of just having them kind of hip flex and knee flex and kind of get that type of movement kind of like as a warm up activity

77

00:10:20.670 --> 00:10:26.539

PT4: quite often with routine and reputation. Once I start it, the child will then kind of show kind of

78

00:10:27.080 --> 00:10:31.190

PT4: you know, imitate and mimic the movement that i'm doing so.

79

00:10:31.570 --> 00:10:34.260

PT4: Yes, majority of children are dependent

80

00:10:34.690 --> 00:10:38.050

PT4: for a lot of the positioning and the movements, but

81

00:10:38.620 --> 00:10:45.200

PT4: there are times where they're able to do it on their own, with minimal assistance.

82

00:10:45.580 --> 00:10:47.769

PT4: with repetition of the activity

83

00:10:48.510 --> 00:10:49.599

PT4: through time.

84

00:10:50.440 --> 00:10:56.879

NM: Thank you. And then do you think they should participate in more or less of the activities you mentioned

85

00:10:57.460 --> 00:10:58.340

PT4: more

86

00:10:58.380 --> 00:10:59.160

okay.

87

00:10:59.820 --> 00:11:01.930

PT4: because I think these children.

88

00:11:02.780 --> 00:11:15.159

PT4: these children don't get enough activity. They don't have enough physical, so it is not built in enough into their routine, and especially, I think, when they go to school in their school, aged a lot of times, they're sitting in their

89

00:11:15.280 --> 00:11:19.419

PT4: wheelchair or their modified adaptive stroller.

90

00:11:19.600 --> 00:11:25.329

PT4: They may be transitioned out a few times, possibly to the standard, or to

91

00:11:25.420 --> 00:11:34.569

PT4: you know, to you know, to some therapy equipment, or out for a therapy session. But I think these children

92

00:11:34.610 --> 00:11:42.269

PT4: part of part of the problem for them is the the the static-ness of their day that they are

93

00:11:42.700 --> 00:11:45.919

PT4: in postures that really Don't contribute

94

00:11:46.400 --> 00:11:54.240

PT4: to helping them benefit, you know, over time. So I I really do feel that movement is key. It stimulates the brain.

95

00:11:54.310 --> 00:12:02.890

PT4: it, you know, increases their alertness, I mean, for all of us it gets to the endorphins running. It gets everything going, so I I do think

96

00:12:02.940 --> 00:12:14.130

PT4: that they do need more physical activity throughout the day. Part of is realistic. They They are now in a school type of program, and you know certain activities have to get done during the day.

97

00:12:14.210 --> 00:12:17.879

PT4: and it does require a lot of one on one

98

00:12:18.600 --> 00:12:20.879

PT4: one on one

99

00:12:21.000 --> 00:12:29.179

PT4: ratio of, you know, child, to caretaker, therapist teacher, you know. So it really requires a lot. But I do. I do feel it.

100

00:12:29.390 --> 00:12:34.019

PT4: That would be much better for them. Cardiovascular at everything. Pulmonary wise.

101

00:12:34.040 --> 00:12:36.730

PT4: I think these children tend to get sick a lot.

102

00:12:36.760 --> 00:12:37.589

PT4: and I think

103

00:12:37.630 --> 00:12:41.419

PT4: the lack of movements is a is a big contributor to it.

104

00:12:41.450 --> 00:12:44.040

PT4: I would just see that in the 0 to threes

105

00:12:44.590 --> 00:12:57.130

PT4: because a lot of times they, when I wasn't there the caretaker would have them, you know, in a in a supine position, a lying position maybe on a wedge where there's somewhat reclined. But these kids would get so many pulmonary

106

00:12:57.350 --> 00:13:03.969

PT4: infections. and so. Yes, I do feel long term for the general health. that they do need

107

00:13:04.060 --> 00:13:15.430

PT4: more movement and activity, whether whether you know whether it's it, it's also doing it for them. You know where it's. It's it's it's assisted type of movement.

108

00:13:15.590 --> 00:13:19.660

PT4: but I do feel that they need more physical activity within their daily routines.

109

00:13:20.310 --> 00:13:21.730

NM: Thank you. That was great.

110

00:13:21.800 --> 00:13:28.750

NM: So just just a comment on what you said earlier, because I kind of want to get a little bit more from you with this you you mentioned

111

00:13:29.760 --> 00:13:38.569

NM: holding their he holding his head. And the guy the little guy you talked about was a huge accomplishment. you said that. And I want to talk about.

112

00:13:39.110 --> 00:13:40.550

NM: How is that?

113

00:13:40.620 --> 00:13:47.150

NM: Because he's sitting, and he's holding his head? How would that? How is that physical activity for this little guy

114

00:13:48.590 --> 00:13:55.529

PT4: because he couldn’t. He had such poor.

115

00:13:56.070 --> 00:14:14.530

PT4: He had almost like an Athetosis type of movement pattern. So he had poor ability to basically sustain him in line, and and he wanted to lift his head. So he would go to the end ranges. So he would extend, or he would completely flex. And so to kind of get that muscular skeletal balance of mid range for him

116

00:14:14.580 --> 00:14:20.690

PT4: was incredible, and for him to sustain it and actually hold it and look at us and smile.

117

00:14:20.880 --> 00:14:26.300

PT4: It was. It was like the you light up, because you knew he was just trying so hard, and he got it.

118

00:14:26.680 --> 00:14:30.810

PT4: and then fatigue would take over, and he would collapse.

119

00:14:31.140 --> 00:14:46.360

PT4: so yeah, so that was how that was such a huge accomplishment for him, and it was such a huge goal for the parents, because and the family and the siblings because they I just wanted him to, you know, look up and look and partake in the activities that were going on in the home.

120

00:14:50.020 --> 00:14:51.260

NM: This is gold.

121

00:14:53.570 --> 00:14:59.299

NM: It's the little things it's the small thing, I mean, and that's the thing, you know. So that.

122

00:14:59.660 --> 00:15:09.789

NM: And this is, you know. Okay, I'm gonna get on my soap box once again I can see you're about to cry. I mean, it's so important, you know. And so I i'm glad that

123

00:15:09.980 --> 00:15:12.710

NM: you know we're gonna, you know. Have this, You know.

124

00:15:12.800 --> 00:15:18.040

NM: documented that they want me to partake in the family moments.

125

00:15:18.120 --> 00:15:20.509

NM: But just

126

00:15:21.110 --> 00:15:24.650

NM: yeah, something right? Okay. Third question.

127

00:15:24.940 --> 00:15:39.439

NM: Do you address promoting physical activity during your actual session like within your session do you promote?

PT4: I am a a strong advocate. My frustration as a therapist is the lack of carry over.

128

00:15:39.620 --> 00:15:40.520

NM: Yeah.

129

00:15:40.930 --> 00:15:49.039

PT4: And the lack of Not that the family doesn't want to do it? I I feel that you know, when you have a child that is so disabled.

130

00:15:49.320 --> 00:15:54.400

PT4: You have a lot of people coming at you with a lot of different things, you know. Gonna say, AFOs on, give them the stander. Do this, do that.

131

00:15:54.430 --> 00:16:04.899

PT4: You know Every therapist has their own regiment of what they want carried over, and I think for a family. Sometimes it can be very overwhelming, and they're going to do what they can, and the best they can within their constraints.

132

00:16:05.110 --> 00:16:10.589

PT4: and so my frustration is the the lack of carry over.

133

00:16:10.640 --> 00:16:29.179

PT4: You know I can order a set of braces, and I can see that they're not being used, you know. I know they look brand new every time I come in and so that you know that could be. And then, you know, sometimes some of these things are complicated. Some of these, you know standers as as much as you try to pick out the stander that's

134

00:16:29.760 --> 00:16:39.060

PT4: going to be the best for the family and their home right in their in their in, you know, whatever the size of the you know what they need, and the best for the child. as far as positioning.

135

00:16:39.340 --> 00:16:52.229

PT4: Sometimes they're very cumbersome and difficult to get in. You know they take. Sometimes they say they take one person, but really they take 2 and so you know, so that that's that's the hard part.

136

00:16:52.430 --> 00:16:57.549

PT4: And I think I think, and probably this is my fault in a way, too, because you.

137

00:16:57.590 --> 00:17:08.989

PT4: by interviewing me, really put, stuck it in my mind as much as I know, and I've said he needs to be set up more. He needs this, for you know, because he's getting he's getting those chronic pulmonary infections.

138

00:17:10.420 --> 00:17:15.200

PT4: You really made me put it together and think more of the actual, real, like

139

00:17:15.380 --> 00:17:33.349

PT4: physical activity for these child, you know, and I know it for the cardiac and for their overall being that digestive system being upright more. You know all the reasons why we're getting these kids up who might never have been put up in 20-30 years ago, you know, who would have kind of been more left Sedentary

140

00:17:33.880 --> 00:17:39.210

PT4: is is, you know it's, and I I need to really explain that more

141

00:17:39.310 --> 00:17:43.539

PT4: for the lifelong health of their child. And I think that's the point

142

00:17:44.270 --> 00:17:46.010

PT4: you're reinforcing to me

143

00:17:46.850 --> 00:18:00.329

PT4: through this interview. You know what my learning, what I'm taking away from this interview is. I really need to be a little bit more of an advocate for the lifelong health of these children in in emphasizing the importance of the physical activity, even though a parent may say, Listen, my child is never going to be.

144

00:18:00.340 --> 00:18:08.869

PT4: you know, kicking a ball and running around a field. But then that's okay. But we have to understand, though, for his overall general health

145

00:18:09.040 --> 00:18:16.270

PT4: to you know, to to to prevent a lot of things in his life from happening. We need, you know, we need to get him moving more.

146

00:18:18.420 --> 00:18:37.130

NM: Thank you. And so you answered this before and i'll just. I'll just bring it up again if you want to add anything else, so i'll know the follow up to this is so you do focus on physical therapy. And then how physical activity in your sessions! How do you do this? And you mentioned that. But what components of physical activity? So you mentioned endurance

147

00:18:37.310 --> 00:18:55.340

NM: before would you? I have some other examples part of that. Yeah, exactly, or anything like that. You want to. So okay, so what type of physical activity? So you address so right? So I mean…

PT4: I would look at strength. I would look at

148

00:18:55.560 --> 00:19:07.339

PT4: endurance. I would look at, you know, passive and active range of motion. so like whatever sporadic and random type of kicking arm, swiping whatever overall movements.

149

00:19:07.390 --> 00:19:10.859

I would look at

150

00:19:12.030 --> 00:19:22.609

PT4: you know, just even just you know, standing as as you know, supported as it may be standing. I would look at weight shifting.

151

00:19:22.780 --> 00:19:27.019

PT4: I would, you know I would. What else would I do with this child?

152

00:19:27.220 --> 00:19:30.659

PT4: I mean, I love to do rolling and stimulation like that.

153

00:19:30.730 --> 00:19:32.390

PT4: so

154

00:19:32.710 --> 00:19:44.450

PT4: it's not. You know those aren't really components, but I mean it definitely. Endurance strengthening passive, active range motion. a lot of positioning a lot of positioning sustaining positions

155

00:19:44.480 --> 00:19:48.860

PT4: transitions, you know. Movement transitions to with these kids.

156

00:19:49.830 --> 00:19:57.290

PT4: And I like to like play with them in those transitional movements just to get that kind of movement rotation things like that that they lack.

157

00:19:58.120 --> 00:20:01.330

PT4: But I would say, yeah. So during those activities I would. I would. You know

158

00:20:01.930 --> 00:20:05.970

PT4: I would. Those Those would be the things that I do.

159

00:20:06.210 --> 00:20:08.340

PT4: Yeah. Okay.

160

00:20:09.040 --> 00:20:13.940

NM: Do you address promoting physical activity that occurs outside of your Pt. Session.

161

00:20:15.570 --> 00:20:25.789

PT4: Do I promote with this with this, with these clients with these? Oh, yeah, absolutely. So. I'm: I'm a I love swimming

162

00:20:25.920 --> 00:20:37.010

PT4: our children, you know, because they're just free, and we all know you know we so swimming is is a huge one, you know, depending on on. I've I've always like looked for

163

00:20:37.150 --> 00:20:49.999

PT4: although they're far in a few between, you know, like a modified dance or a modified type of the yoga or gymnastics type of of an activity I do promote like a lot of the

164

00:20:50.010 --> 00:21:01.539

PT4: the videos and the songs, and you know head, shoulders, knees, and and toes like where you know they're kind of doing it with their child. so like you know that type of that type of a

165

00:21:01.550 --> 00:21:17.660

PT4: activity Karate, I mean depending on the level of the child. But I would, I mean I've recommended everything from soccer, you know, to like. Let's say level one level 2 to you know, Karate, to you. Know anything where it could be Somewhat adaptive

166

00:21:17.750 --> 00:21:26.889

PT4:, you know, sometimes hard for for families is find adaptive, and and some of these programs, you know, is

167

00:21:28.180 --> 00:21:34.900

PT4: they don't there's not a lot in the communities, and if you can find one that's willing to accept a child that might have a disability.

168

00:21:35.110 --> 00:21:45.820

PT4: you know it. It's. You know I had one swimming program that was that, as long as the father was in the pool, you know they were fine with him, kind of going along with whatever they Did you know?

169

00:21:47.980 --> 00:21:52.999

NM: right? So you answer my next follow about community programs, or that.

170

00:21:53.030 --> 00:21:55.170

NM: And so the last follow up

171

00:21:55.190 --> 00:21:59.790

NM: really would be, what type of equipment. Have you recommended for the home and community

172

00:22:00.150 --> 00:22:02.550

NM: for a community engagement?

173

00:22:02.800 --> 00:22:05.220

PT4: Okay. So for the home.

174

00:22:05.330 --> 00:22:12.420

PT4: I mean everything from bath chairs to adaptive seating systems to adaptive strollers to standers.

175

00:22:12.490 --> 00:22:16.599

PT4: to the use of tumble forms, kind of equipment for positioning

176

00:22:17.110 --> 00:22:26.439

PT4: afos all sorts of orthotics and and leg braces splints.

177

00:22:26.710 --> 00:22:37.970

PT4: for in the home therapy balls, bolsters, you know, whatever whatever the home could support, and whatever the constraints are, Sometimes it's it's this: it's this very small apartment, you know, with 5 or 6 people living in it, and one or 2 bedroom.

178

00:22:38.080 --> 00:22:40.909

PT4: and sometimes it's a larger home, where you know they can

179

00:22:41.330 --> 00:22:46.630

PT4: manage this equipment. wedges

180

00:22:47.690 --> 00:22:55.089

PT4: even like a floor kind of like a little’ payley’ guys. I mean, these kids are little, too. So, depending on like.

181

00:22:55.260 --> 00:23:00.720

PT4: you know. So in the home for that subset and that age those are the kind of like

182

00:23:01.030 --> 00:23:07.559

PT4: things. But we've had modified. I've I've modified like tricycles, you know, like little things like that where we've had them.

183

00:23:07.640 --> 00:23:09.819

PT4: you know. I've modified.

184

00:23:09.970 --> 00:23:12.400

PT4: I mean so even I mean, I know.

185

00:23:12.470 --> 00:23:15.800

PT4: a lot of therapist hate these things. But you know

186

00:23:16.510 --> 00:23:20.469

PT4: sometimes just just for activity and and for enjoyment. And with.

187

00:23:20.590 --> 00:23:26.590

PT4: explains the parent, you do not leave them. And these things are, You know, the exersoucers and the bouncers and things like that.

188

00:23:26.820 --> 00:23:37.710

PT4: because they're fun, and the child enjoys them, and and it's an activity. So for this zero to 3 subset a lot of times, I will take that those baby equipment and modify it.

189

00:23:37.910 --> 00:23:51.529

PT4: for the family, you know, so that the child can use it safely and enjoy it. And in a way they're upright, so they're not lying on their back or on their side or and and so for that subset

190

00:23:52.060 --> 00:23:56.280

PT4: So i'll do that, and then for the outside.

191

00:23:56.380 --> 00:24:04.840

PT4: So the question for the outside just repeated again, and then i'll answer it for the outside.

NM: Okay. So what types of equipment have you recommended to help improve

192

00:24:05.110 --> 00:24:12.420

NM: home? Which you answer? So this will be improve community engagement outside of the clinical setting.

193

00:24:12.520 --> 00:24:20.470

PT4: Right? So again it would be a a tricycle or a modified kind of push car again. It's a 0 to 3 subset.

194

00:24:20.510 --> 00:24:25.230

PT4: so I would. I would recommend the things like that.

195

00:24:25.260 --> 00:24:41.500

PT4: I we've had. We've recommended that they have a backyard, so we've gotten them swings adaptive swings. Yeah, for the outdoors. what else have I recommended for the outside?

196

00:24:42.410 --> 00:24:45.019

PT4: Aside from the car, tricycle.

197

00:24:45.140 --> 00:24:48.180

PT4: the swing.

198

00:24:50.850 --> 00:24:51.669

PT4: I think

199

00:24:52.440 --> 00:24:55.850

PT4: I think that's pretty much it. I think those are the things pretty much that

200

00:24:57.170 --> 00:24:58.410

PT4: I frequented.

201

00:24:58.600 --> 00:25:07.940

NM: Oh, that is great, all right. So we're at the second part. So i'm gonna go ahead and share my screen, cause I want you to take a look at this is called the Promise

202

00:25:08.350 --> 00:25:13.670

NM: Parent Proxy, physical activity Survey. So this is on created by then

203

00:25:14.000 --> 00:25:19.200

NM: National Institute of Health and Ideally, it's supposed to be for children that

204

00:25:19.740 --> 00:25:25.630

NM: are more involved. So the parent will fix it for this out, and it's a measure of physical activity intensity.

205

00:25:25.690 --> 00:25:36.330

NM: So go ahead. I'm gonna ask you about these 8 questions. I'm trying to make sure I get them all in there, so you can just kind of look at it. I mean, i'm gonna actually, one by one. So the parent would go ahead and answer in the past 7 days. You know

206

00:25:36.830 --> 00:25:40.679

NM: how many days and they click. And so basically. How intense

207

00:25:40.790 --> 00:25:46.639

NM: is their their week of activity, right? And so my questions to you are going to be geared to

208

00:25:46.720 --> 00:25:50.009

NM: thinking about the children that are not inventory. Right?

209

00:25:50.150 --> 00:25:52.559

NM: How appropriate is this tool!

210

00:25:53.740 --> 00:26:05.200

NM: And it could be for parent or for a a therapist, whoever, how appropriate, because we don't really have that many measures. That's really what i'm getting at right. So this is one of them I found. So I just want to ask my colleagues.

211

00:26:05.360 --> 00:26:09.979

NM: How do I feel about it? So I'm asked you to rate it from 0 being it's not related at all.

212

00:26:10.420 --> 00:26:13.420

NM: or 5. It's highly appropriate question to ask.

213

00:26:13.560 --> 00:26:16.409

NM: This could be beneficial. And then I'm gonna ask you Why?

214

00:26:16.480 --> 00:26:19.670

NM: Okay? The first question.

215

00:26:20.160 --> 00:26:27.560

NM: as you see. i'm going to ask you to rate it. How related is this: for physical activity, and children are non ambulatory with Cp.

216

00:26:27.800 --> 00:26:36.710

NM: How many days is your child exercise, or place so hard that his or her body got tired. So you're going to give me a rating from 0 to 5

217

00:26:37.920 --> 00:26:42.150

PT4: right. So I think that that's a good question. I think

218

00:26:42.590 --> 00:26:43.850

PT4: that that

219

00:26:43.920 --> 00:26:47.540

PT4: I would give it a 5, because I think

220

00:26:48.530 --> 00:26:53.919

PT4: I think that only is this a. You know it's. It's a tool, and it also kind of

221

00:26:53.950 --> 00:27:02.449

PT4: in my into the parent that oh, my child, should be exercising to get to get tired. So I I think that it's it's a good question.

222

00:27:02.660 --> 00:27:06.460

PT4: because sometimes these children, what I find also is they're they're left home

223

00:27:06.850 --> 00:27:12.029

PT4: like be able to take the rest of the family out, and sometimes these kids will be left home when they do an outing.

224

00:27:12.330 --> 00:27:16.829

PT4: and I know it's hard sometimes to bring these kids to some outings.

225

00:27:16.910 --> 00:27:23.269

PT4: but it it it it it. It stimulates in the mind that they need to be incorporated into

226

00:27:23.440 --> 00:27:26.889

PT4: physical activities, just as the other children in the family are.

227

00:27:28.170 --> 00:27:32.770

PT4: so no, I think it's a good question. I think it's a good question.

228

00:27:32.920 --> 00:27:37.309

NM: all right. So next question is, how many days

229

00:27:37.940 --> 00:27:47.749

NM: did your child exercise really hard for 10 min or more. What what would you give that from a scale from 0, not related at all to 5? Being highly appropriate for this population.

230

00:27:48.680 --> 00:27:51.350

PT4: I would say 5

231

00:27:52.410 --> 00:27:56.230

PT4: for the same reason before same reason, Same reason.

232

00:27:58.370 --> 00:28:00.410

NM: Okay. And number 3.

233

00:28:02.000 --> 00:28:06.430

NM: How many days your child exhausted so much that he or she breathed her.

234

00:28:11.310 --> 00:28:17.810

PT4: That I don't know. It's so appropriate because some of these kids have respiratory problems to begin with.

235

00:28:19.180 --> 00:28:25.719

PT4: So how would you? So that make it, I would give it a 3, because it's a To me it's a neutral question in the fact

236

00:28:25.860 --> 00:28:28.699

PT4: that that's kind of more of a clinical question.

237

00:28:28.950 --> 00:28:37.190

PT4: because the parent might not be able to differentiate, you know they might say, Well, my child, you know, breathes hard all the time, or you know my

238

00:28:37.610 --> 00:28:41.530

PT4: so. you know, and some of these kids may have

239

00:28:41.620 --> 00:28:55.309

PT4: ribcage, you know. Deformations, or you know they have might have, you know, from being in that wind, so sweat, position, or whatever it is. That so? I would say I would get like the neutral kind of a 3, because I think that would confuse a parent.

240

00:28:55.320 --> 00:29:02.569

PT4: in a way. and I think a lot of these kids sometimes do have breathing problems that they parent might not be able to differentiate

241

00:29:03.450 --> 00:29:05.769

NM: Gotcha All right. Number 4.

242

00:29:05.930 --> 00:29:10.619

NM: How many days was your child so physically at active that he or she sweated

243

00:29:11.250 --> 00:29:15.249

NM: 0? Not it related all 5 highly, really highly appropriate.

244

00:29:19.250 --> 00:29:20.309

PT4: How many days?

245

00:29:23.370 --> 00:29:28.480

PT4: again, I think I would give that a 3, because

246

00:29:29.520 --> 00:29:31.540

PT4: the child probably

247

00:29:32.120 --> 00:29:36.080

PT4: so. I mean, I find with some of these kids Level 5 is they have

248

00:29:36.680 --> 00:29:45.910

PT4: auto regulation issues right? So you know, if it's if it's very hot out, yeah, they'll they. They, they'll swept up aspire. They

249

00:29:45.960 --> 00:29:50.430

PT4: you know, even like when they get fevers and things like that I find with the younger population.

250

00:29:52.460 --> 00:29:58.019

PT4: you know that I find that their auto regulation is a a little different than ours. So

251

00:29:58.560 --> 00:30:01.280

PT4: and I mean honestly.

252

00:30:01.380 --> 00:30:04.110

PT4: I've never had a child sweat on me

253

00:30:04.670 --> 00:30:09.410

during like a 30 min. Pretty intensive therapy session. So

254

00:30:09.720 --> 00:30:14.240

PT4: again I would give that kind of a neutral 3

255

00:30:15.180 --> 00:30:17.570

PT4: you know, or maybe even a 2.

256

00:30:17.610 --> 00:30:19.930

NM: Okay, you. You want me to change it to it, too.

257

00:30:20.220 --> 00:30:23.200

PT4: We could give it a 2. We could give it a 2. Okay.

258

00:30:23.780 --> 00:30:25.370

NM: all right. Number 5.

259

00:30:25.450 --> 00:30:30.419

NM: How many days your child exercise or play so hard that his or her muscles burn

260

00:30:31.480 --> 00:30:33.079

PT4: right? So I would.

261

00:30:34.420 --> 00:30:40.170

PT4: I would probably give that a 2, because

262

00:30:40.570 --> 00:30:44.739

PT4: and again i'm, I'm thinking from my subset. Most of my kids are not verbal.

263

00:30:47.410 --> 00:30:50.609

PT4: So I don't know if they can explain

264

00:30:51.380 --> 00:30:55.139

PT4: what muscles burning feel like. So I think that's subjective.

265

00:30:55.480 --> 00:30:57.180

PT4: Hmm. That's why.

266

00:30:57.570 --> 00:30:58.140

Okay.

267

00:31:00.420 --> 00:31:03.319

PT4: So I think it would be too subjective to assess

268

00:31:03.460 --> 00:31:04.550

PT4: accurately

269

00:31:06.600 --> 00:31:08.529

PT4: if that makes sense.

270

00:31:10.010 --> 00:31:10.970

NM: All right.

271

00:31:13.440 --> 00:31:15.000

Oh, good thanks.

272

00:31:15.300 --> 00:31:16.689

NM: all right. Number 6.

273

00:31:16.980 --> 00:31:21.589

NM: How many days did your child exercise or play so hard that he or she felt tired.

274

00:31:21.840 --> 00:31:27.769

PT4: so that I would give a 5, because I think that is much more easier to assess.

275

00:31:27.920 --> 00:31:32.609

PT4: I think you know parents know very quickly what this, what you know, what

276

00:31:32.740 --> 00:31:38.119

PT4: what what their child is when they get tired. What you know, what what what the

277

00:31:38.470 --> 00:31:48.579

PT4: queues are, so that I would give a 5 because a parent can quickly. We know, you know, when your kids tired it's like immediate like. You can see the signs

278

00:31:48.740 --> 00:31:50.410

PT4: so that I would give a 5,

279

00:31:52.750 --> 00:31:53.680

NM: all right.

280

00:31:53.710 --> 00:31:59.449

NM: And then number 7 is how many days with your child physically active for 10 min or more.

281

00:31:59.880 --> 00:32:01.280

PT4: Yeah. Great question.

282

00:32:01.370 --> 00:32:02.679

PT4: That's easy to measure.

283

00:32:02.840 --> 00:32:03.650

NM: Okay.

284

00:32:06.030 --> 00:32:08.779

NM: And then the last one

285

00:32:09.790 --> 00:32:13.219

NM: 8. How many days your child run for 10 min or more?

286

00:32:14.850 --> 00:32:16.220

PT4: Right? So

287

00:32:16.310 --> 00:32:19.440

PT4: when we, when we're talking about the fours and fives like they.

288

00:32:19.500 --> 00:32:25.760

PT4: They're they're not running right there. So I would give that probably a one, because it's

289

00:32:26.040 --> 00:32:29.640

PT4: It's not a practical question for that subpopulation.

290

00:32:39.330 --> 00:32:46.990

NM: All right, and before we end always ask, is there anything else you want to add about? You know your understanding, your feelings of all physical activity in this population.

291

00:32:49.560 --> 00:32:55.320

PT4: no, I think this was a great I I think this is a great study to be doing

292

00:32:56.760 --> 00:33:03.240

PT4: it. It's very eye-opening. I think even a lot of these questions who are like so this this to be presented the parents is, is

293

00:33:03.510 --> 00:33:07.610

PT4: it's it's a good kind of self-learning activity for them as well.

294

00:33:07.960 --> 00:33:11.829

PT4: you know so no overall, I think I think this was

295

00:33:12.100 --> 00:33:14.090

PT4: excellent, because you know

296

00:33:14.150 --> 00:33:20.579

PT4: as long as I've been practicing a therapist. You know it. It all of these questions that open my eyes up again

297

00:33:20.910 --> 00:33:24.310

PT4: to the specific needs of this population, the subset.

298

00:33:27.190 --> 00:33:29.790

NM: Well, let me stop our Corey. Thank you.

299

00:33:29.820 --> 00:33:33.009

PT4: Thank you.

300

00:33:33.900 --> 00:33:34.829

PT4: Am I off camera?
