# Supplementary material for: Validity of PROMIS® Pediatric Physical Activity Parent Proxy Short Form Scale as a Physical Activity Measure for Children with Cerebral Palsy Who Are Non-Ambulatory
Source: Behav Sci (Basel). 2025 Jul 31;15(8):1042. doi: 10.3390/bs15081042 (PMC12382615; doi:10.3390/bs15081042)
Supplement: Supplementary file 1 [file behavsci-15-01042-s001.zip › Transcripts copy/PT transcripts - deidentified/PT22.docx]

WEBVTT

1

00:00:00.960 --> 00:00:07.099

NM: Okay, Thank you so much for joining me today. We are going to spend some time

2

00:00:07.330 --> 00:00:17.250

NM: talking about physical activity and children that have Cp. Who are not ambulatory. So if it sounds like I'm scripted, is because I am.

3

00:00:17.260 --> 00:00:37.129

NM: I have a couple of questions for you, and a couple of prompts. The first half will be questions about your critical expertise, and there's no right or wrong. Answer. I'm learning from the clinicians that work with this population every day and day and day out, and then the second half is going to be me sharing a survey with you. Develop on the national health.

4

00:00:37.360 --> 00:00:47.819

NM: looking at how you will perceive this survey to be valid for this population in terms of assessing physical activity, intensity. So we'll do that in the second half any questions.

5

00:00:48.830 --> 00:00:49.900

NM: Okay? Great.

6

00:00:50.100 --> 00:00:52.070

NM: So the first question I have for you

7

00:00:52.180 --> 00:00:57.719

NM: is, how do you define physical activity for children with Cp who are not full-time Walkers

8

00:01:00.750 --> 00:01:02.839

PT22: activities that

9

00:01:03.560 --> 00:01:12.320

PT22: would help to facilitate, increase heart rate and increase motor performance, increase engagement.

10

00:01:13.620 --> 00:01:14.860

PT22: increase fun.

11

00:01:16.620 --> 00:01:17.360

PT22: Hmm.

12

00:01:18.870 --> 00:01:19.429

Okay.

13

00:01:19.600 --> 00:01:22.019

PT22: I think that's it. I mean, I know there's more.

14

00:01:22.710 --> 00:01:28.420

NM: No, that's great, thank you. All right. So my first prompt is the Department of Health.

15

00:01:28.560 --> 00:01:37.059

NM: The find physical activity of any activity it encompasses energy expended in activation of skeletal muscle. Does this change your definition

16

00:01:37.640 --> 00:01:39.220

NM: of how you do find PA?

17

00:01:40.690 --> 00:01:42.280

PT22: No, I don't think so.

18

00:01:43.570 --> 00:01:49.140

NM: And how do you think physical activity differs from other types of fitness activity.

19

00:01:54.820 --> 00:01:58.000

PT22: But I guess with fitness, there's always the thought that you're going to be

20

00:01:58.170 --> 00:02:01.869

PT22: really kind of building on and getting your endurance up.

21

00:02:01.910 --> 00:02:06.250

PT22: I think that's probably the biggest thing is to work on and skill

22

00:02:09.310 --> 00:02:10.080

NM: great.

23

00:02:10.190 --> 00:02:15.130

NM: When do you witness? Your students participate most in physical activity during the school day.

24

00:02:17.280 --> 00:02:19.850

PT22: probably when they are in physical therapy.

25

00:02:22.040 --> 00:02:24.520

PT22: Otherwise they're pretty much in the wheelchairs

26

00:02:25.020 --> 00:02:26.240

NM: all right.

27

00:02:27.980 --> 00:02:29.970

NM: And next question.

28

00:02:30.160 --> 00:02:38.530

NM: How do you measure physical activity, frequency, intensity, time and type, and children with Cp. Who are not full time Walkers.

29

00:02:39.270 --> 00:02:47.319

PT22: Well, we do have a treadmill. I did not write that down as one of the things that we use. But we do have a treadmill, so we would sometimes be

30

00:02:47.530 --> 00:02:53.930

PT22: document how how long they're on the treadmill more commonly. What we'll do is

31

00:02:54.280 --> 00:03:10.809

PT22: when they're in a partial weight, bearing system or gait trainer, Rifton pacer or tram. Then we will indicate how far along the hallway. They can travel without taking a rest. That's probably one of the more frequent months we also do when I put this one in there, either adaptive bikes

32

00:03:10.820 --> 00:03:15.300

PT22: we do, adaptive bikes as well, our kids most typically.

33

00:03:16.410 --> 00:03:18.729

PT22: and we have a few that can actually

34

00:03:18.780 --> 00:03:34.760

PT22: output the propelling. Most of the kids can only do a partial or no propelling, but if they can, then we'll be looking at that if it's. Sometimes we're just looking at. Can they hold their head up and stay engaged in the world around them while they're on their bike.

35

00:03:34.770 --> 00:03:40.440

PT22: So it's very variable, depending upon the device or equipment we're using

36

00:03:42.070 --> 00:03:42.830

NM: right

37

00:03:43.080 --> 00:03:56.699

NM: and you are you actually answer my next prompt? Do they need assistance to complete these activities, and during which tasks do they need assistance, and do they need assistance for the whole task or just part of it. So you kind of you kind of answered that. But do you have anything else to add.

38

00:03:56.730 --> 00:03:58.500

PT22: Yeah, no. I mean our kids

39

00:03:58.800 --> 00:04:01.339

PT22: pretty much always require some.

40

00:04:01.560 --> 00:04:15.459

PT22: And the bikes they're pretty almost. We have one or 2 kids that can steer the most part they do not. We have one or 2 kids that can steer even a walker, but most cannot. So yeah.

41

00:04:15.640 --> 00:04:18.630

PT22: physical systems always pretty well required.

42

00:04:19.160 --> 00:04:19.950

NM: Okay.

43

00:04:22.130 --> 00:04:27.129

NM: And do you think they should participate in more or less of each of these activities? And why?

44

00:04:27.820 --> 00:04:44.379

PT22: Well, there's been so many studies out that show that the kids alertness and awareness definitely increases with physical activity. We have been trying to promote that in our school, and so we would like to see more of it. Some of the teachers are a little bit more

45

00:04:44.690 --> 00:04:48.559

PT22: buying into that, and do actually

46

00:04:48.700 --> 00:05:00.600

PT22: use those techniques. We have some teachers that will come down and say, hey, we're ready to do a bike group, and they'll come and get the bikes from us, and they'll put the kids all on bikes, and and they'll do that first thing in the morning.

47

00:05:00.670 --> 00:05:02.380

NM: And okay, that's great.

48

00:05:04.210 --> 00:05:06.490

NM: The teachers are helping out. That's awesome.

49

00:05:08.480 --> 00:05:12.950

NM: Alright, so your your answer is more more of these activities.

50

00:05:13.340 --> 00:05:14.830

NM: All right. Next question.

51

00:05:14.870 --> 00:05:19.239

Do you address promoting physical activity during your physical therapy sessions?

52

00:05:20.710 --> 00:05:27.650

PT22: Yes, so is the question pertaining to the child or to the environment. I guess it's the question.

53

00:05:28.010 --> 00:05:40.049

NM: It's to your session, so it you can include different environments or or not. And then so the next question is, how how do you do this? How do you promote physical activity during your Pt session, since you you answered Yes.

54

00:05:40.060 --> 00:05:48.150

PT22: right right. So we do try to get the kids up and moving into either a gait trainer or a bike, or

55

00:05:48.160 --> 00:06:08.099

PT22: partial weight bearing system of some sort, if the child can. Sometimes it could just be that the child is actually working on sit to stand stand transition. So we would start him off on the bench and have them facilitate coming up to stand. That's very common and very typical. So. But it is engaging mobility so

56

00:06:08.410 --> 00:06:09.740

PT22: pretty much.

57

00:06:09.770 --> 00:06:23.600

PT22: I think we see the kids 2 to 3 times a week, and sometimes we'll put them into a power wheelchair. More of a driving to learn activity, but they're just learning switch stuff, but that's still getting mobility and encouraging head

58

00:06:23.610 --> 00:06:34.060

PT22: holding up and lifting. So, even though it's maybe not as visual as being in a gait trainer. It's still for our kids facilitating movement and physical activity.

59

00:06:38.120 --> 00:06:38.970

NM: It's great.

60

00:06:39.350 --> 00:06:53.389

NM: And are there ever I am awesome? I'm sorry. So you answered. How you do this. But what component of physical activity. Do you address, for example, Are you focusing on cardiovascular endurance, muscle, activation, mobility, energy, expenditure.

61

00:06:56.760 --> 00:07:13.429

NM: so that it can be? And there's so many more options. But what are the components of physical activity that that do you primarily address? Is it, for example, cardiovascular endurance, muscle, activation, mobility, energy, expenditure, just to name a few.

62

00:07:13.620 --> 00:07:20.819

PT22: Okay. So muscle activation is probably the key one for our level of kits just to keep them going. I wouldn't say

63

00:07:21.380 --> 00:07:25.910

PT22: we get very heavily into the cardiovascular, just because

64

00:07:27.320 --> 00:07:32.099

PT22: it is so difficult for our kids to actually get up and get moving with any

65

00:07:33.180 --> 00:07:38.000

PT22: to enough to really kind of promote that.

66

00:07:38.410 --> 00:07:43.570

PT22: but just in mobility is big for us. So, however, we can get the kids movement.

67

00:07:43.690 --> 00:07:56.339

PT22: and that does actually, I know, work some of the cardiovascular, but it is such a wax, and, Wayne, with our kids, it's very hard to keep them moving. They have to have frequent downtime, so

68

00:07:57.470 --> 00:08:02.440

NM: and if you are not working on any of these areas, what would be the reason why you don't focus on it?

69

00:08:03.520 --> 00:08:17.500

PT22: Adaptive equipment needs to having to fix something on their wheelchair, having to do something with their braces pretty much. We're always working at some component on it, unless there's something or testing

70

00:08:17.700 --> 00:08:21.570

PT22: testing, would be another reason why we wouldn't be doing it. That session

71

00:08:21.760 --> 00:08:23.619

PT22: range of motion assessment.

72

00:08:23.640 --> 00:08:25.780

PT22: Gmfms: Pedi-Cats

73

00:08:26.260 --> 00:08:27.190

NM: Gotcha

74

00:08:28.330 --> 00:08:35.509

NM: awesome. All right. Next question. Do you address promoting physical activity that occurs outside of your physical therapy session.

75

00:08:36.000 --> 00:08:52.230

PT22: Yes, we do so again. We try to encourage it in the the school wide. We will jump in and do co-treates with whoever we can. Actually so is the most frequent. We have a dance movement therapist who is outstanding, and

76

00:08:52.240 --> 00:09:07.680

PT22: we do try and jump in for some of her sessions. We do go in and jump in when we can in the classroom, and we do communicate with the families, and especially during Covid. It was

77

00:09:07.830 --> 00:09:11.570

like the primary way is doing it through the family.

78

00:09:15.810 --> 00:09:20.469

NM: And have you recommended any community programs for events to

79

00:09:21.690 --> 00:09:24.810

NM: events, to your students to help increase physical activity.

80

00:09:24.930 --> 00:09:40.780

PT22: Yeah. Some we have through the years. And again, with things just coming up, Covid. Probably not as much of least recent year, but in the past we have found some. Y [YmcA] programs that have been good for our kids. We found

81

00:09:40.790 --> 00:09:52.350

PT22: some Karate studios that have been accepting of our kids some horse back riding programs that we know of, that we advocate to families. Hey, go, check this out

82

00:09:52.500 --> 00:09:57.669

PT22: some of our family, so do it on their own. We have quite a few families that

83

00:09:57.860 --> 00:10:11.520

PT22: I just got a picture this week from one of my kids family, and it's like, oh, we just went adaptive skating. Yeah. So so we do have quite a few families that are very into finding resources for their kids to do.

84

00:10:12.700 --> 00:10:13.560

NM: Wonderful!

85

00:10:13.710 --> 00:10:19.899

NM: What type of equipment have you recommended to help improve home and or community engagement?

86

00:10:22.840 --> 00:10:27.059

PT22: Well, strollers, believe it or not, just so that they're more

87

00:10:27.840 --> 00:10:38.410

PT22: the the world is more accessible than what they would be in power or manual wheelchair. That's a hard one, because then the kids are somewhat more dependent.

88

00:10:38.430 --> 00:10:39.580

PT22: Yeah

89

00:10:39.740 --> 00:10:40.300

it.

90

00:10:40.400 --> 00:10:45.209

PT22: you know. Sometimes it's what's needed in order to get them around. But

91

00:10:45.270 --> 00:11:00.779

PT22: sometimes families have come to us and said, hey, if you know any way to do like it, the big pool ones that you can use for the beach, so we'll get resources for them that way. Big time advocates of having the families for any of that stuff or for adaptive bikes.

92

00:11:00.840 --> 00:11:06.790

PT22: We'll help with that as well. But to go up to the abilities expo, which is in New Jersey.

93

00:11:06.940 --> 00:11:09.929

PT22: so that the families can go and try things out there.

94

00:11:10.000 --> 00:11:10.830

PT22: So

95

00:11:11.120 --> 00:11:24.899

PT22: But if a family has an idea or a thought of something that they really want to do, or we have something that we think, hey, this might work for Johnny or or Sally, or whoever. Then we'll be checking about the families and saying, hey, can we be interested in checking this out

96

00:11:25.000 --> 00:11:25.850

PT22: so.

97

00:11:26.670 --> 00:11:34.179

PT22: and also for resources for the funding for that, too. There's a couple of different resources, this up especially for the adaptive bikes. Now

98

00:11:34.210 --> 00:11:35.660

PT22: that we kind of try and

99

00:11:35.730 --> 00:11:37.169

PT22: hook the families up with

100

00:11:41.230 --> 00:11:42.140

NM: that's great.

101

00:11:44.040 --> 00:11:51.960

NM: so we're already at the second part. So let me go ahead and show you the promise survey which was developed

102

00:11:52.000 --> 00:11:54.599

NM: for children, that we're going through

103

00:11:55.500 --> 00:11:57.710

NM: some level of

104

00:11:59.110 --> 00:12:17.229

NM: a treatment, or like, maybe a neural, progressive, or really oncology is what I believe. This prompted the development of the survey. So it is a survey that is geared to parents to answer for their child. And the parent answers 8 questions. This is the short form survey.

105

00:12:17.240 --> 00:12:22.120

NM: and they record how many days in the past 7 days that they, basically

106

00:12:22.430 --> 00:12:36.879

NM: their child, participate in one of these a criteria. So what I'm going to do is i'm going to go through this question and ask you how appropriate is this question as it relates to the children, we're talking about. What is children with Cp. Functioning at levels? Gms: Yes. Levels 4 and 5.

107

00:12:37.280 --> 00:12:41.510

PT22: Okay. So take a moment to look at that over, and i'll go through each question.

108

00:12:43.270 --> 00:12:45.100

NM: So the first question

109

00:12:46.240 --> 00:12:58.170

NM: i'm going to ask you how appropriate is the question, addressing physical activity, intensity with these children at in Cp. With Cp. Who are not full time Walkers. So the first question is.

110

00:12:58.670 --> 00:13:03.509

NM: How many days did your child exercise a place so hard that his or her body got tired.

111

00:13:03.690 --> 00:13:07.209

NM: So please give me a rating from 0 to 5

112

00:13:07.350 --> 00:13:13.940

NM: 0, not related all 5 highly appropriate, or in between. How would you rate this question and why?

113

00:13:15.840 --> 00:13:32.070

PT22: I I guess I go between 4 and 5, and that I guess i'd have to say 5, I guess my big concern about it is that so many of our kids don't sleep well at night, and so the whole tired part of it is hard to be objective, because

114

00:13:32.340 --> 00:13:36.529

but I would assume that the families would have a general idea of like.

115

00:13:36.760 --> 00:13:40.039

PT22: Oh, it's the middle of the day, and Johnny's usually

116

00:13:40.340 --> 00:13:49.069

PT22: sleepy because he didn't sleep that night. So because of that. But it is one concern i'd have for our kids the sleeping good point.

117

00:13:49.110 --> 00:13:49.990

NM: Good point.

118

00:13:50.140 --> 00:13:51.919

NM: Okay. Thank you. Number 2.

119

00:13:52.000 --> 00:14:00.529

NM: How many days your child exercise really hard for 10 min or more. Would you rate that a 0 not related at all 5 highly appropriate, or somewhere between. And why

120

00:14:01.340 --> 00:14:04.740

I probably give that a 5 as well.

121

00:14:04.940 --> 00:14:10.770

PT22: Again, I think the families typically know their kids well enough to know that in activity

122

00:14:11.160 --> 00:14:12.150

PT22: was

123

00:14:13.170 --> 00:14:15.270

PT22: requiring energy expenditure.

124

00:14:15.630 --> 00:14:17.550

PT22: So I think I would give that 1 5

125

00:14:18.580 --> 00:14:20.949

NM: number 3.

126

00:14:21.300 --> 00:14:23.970

NM: How many days did your child

127

00:14:24.470 --> 00:14:28.859

NM: exercise so much that he or she breathed hard. How would you rate that question?

128

00:14:29.200 --> 00:14:34.659

NM: 0 not related at all? 5 highly appropriate, or somewhere between. And Why.

129

00:14:35.090 --> 00:14:43.599

PT22: yeah, I give this one somewhere between I. My experience is not been that most of our parents get their kids moving to the point where they breathe are

130

00:14:45.390 --> 00:14:50.830

PT22: I don't. I I think it's hard for our kids to get to that point unless they have

131

00:14:51.320 --> 00:14:58.670

PT22: an illness or respiratory issue coming on. And Obviously that would not be what we would be looking for.

132

00:14:58.780 --> 00:15:01.689

NM: So what number would you give it? Give it? Finally? Answer.

133

00:15:02.050 --> 00:15:02.960

PT22: 3.

134

00:15:03.410 --> 00:15:04.270

NM: Okay.

135

00:15:06.430 --> 00:15:10.110

PT22: And for the same reason I give the next 1 3.

136

00:15:10.650 --> 00:15:16.020

NM: Okay. So this one is the sweating, I think. Yes, Number 4. How many days did your child

137

00:15:16.250 --> 00:15:27.399

NM: exercise so? No. How many days was your child so physically after that he or she sweat it. So you said you give it to

138

00:15:27.440 --> 00:15:32.869

PT22: yeah, I would drop it down even to the 2, because I don't think

139

00:15:35.530 --> 00:15:39.119

PT22: I can't Even I can't even begin to think of time I've seen.

140

00:15:39.220 --> 00:15:47.189

PT22: except for when they're crying any of our kids wet really much. I don't. I don't tend to see that as being

141

00:15:47.920 --> 00:15:52.080

PT22: something I even commonly seen. There are people with them up and walk walking.

142

00:15:52.520 --> 00:15:53.839

PT22: They don't tend this way

143

00:15:53.980 --> 00:16:01.169

PT22: so, and I think that's just because they're not challenged like their systems can't handle it. So I don't think that's a valid

144

00:16:03.070 --> 00:16:06.370

PT22: assessment.

145

00:16:06.570 --> 00:16:08.180

NM: Gotcha. Thank you.

146

00:16:08.490 --> 00:16:18.509

NM: Number 5. How many days did your child exercise or play so hard that his or her muscles burned? How would you rate that question 0 not related at all. 5 highly appropriate. And why

147

00:16:18.790 --> 00:16:21.749

PT22: I put this in that same category, I to give it a 2

148

00:16:21.790 --> 00:16:22.859

PT22: again. Okay.

149

00:16:22.880 --> 00:16:29.280

PT22: I see our kids having cramps which are not the same things as having their muscle burn from, you know.

150

00:16:29.960 --> 00:16:36.520

PT22: really doing an activity. Yeah, our kids end up cramping more, which is again not the outcome you want.

151

00:16:38.580 --> 00:16:39.830

PT22: So I give it a 2

152

00:16:42.410 --> 00:16:44.530

NM: number 6.

153

00:16:46.010 --> 00:16:48.280

PT22: So i'm not saying.

154

00:16:48.620 --> 00:16:56.739

NM: Yeah, the first one in this one. Yeah, this one is more. So how many days is your chat? I' to play so hard that he or she felt tired.

155

00:16:58.110 --> 00:17:00.300

PT22: So they actually have to say.

156

00:17:00.580 --> 00:17:01.950

PT22: Hey, i'm tired.

157

00:17:02.060 --> 00:17:11.490

NM: They don't have to say that I think the parent makes that this termination, because this could be for a child that is not verbal. So you still have to interpret.

158

00:17:11.530 --> 00:17:14.609

NM: If the child felt tied versus their body. Got tired.

159

00:17:15.180 --> 00:17:18.670

PT22: That's hard.

160

00:17:19.260 --> 00:17:22.400

NM: How would you rate that 1 0 9?

161

00:17:22.710 --> 00:17:24.490

PT22: I probably give that a 4.

162

00:17:24.579 --> 00:17:33.029

PT22: I think that I I think it's a hard difference between body gotten tight or felt tied. But I still think that's more appropriate.

163

00:17:35.850 --> 00:17:36.600

NM: Okay.

164

00:17:36.860 --> 00:17:37.650

NM: Got it?

165

00:17:38.010 --> 00:17:39.549

NM: This to number one, right?

166

00:17:39.650 --> 00:17:40.370

PT22: Yeah.

167

00:17:41.800 --> 00:17:45.230

NM: All right. And then number 7 is how many days

168

00:17:45.990 --> 00:17:49.590

was your child physically active for 10 min or more. How would you like that one

169

00:17:49.670 --> 00:17:51.330

PT22: that I give a 5?

170

00:17:51.400 --> 00:17:52.730

PT22: I think that's it.

171

00:17:53.110 --> 00:17:55.480

PT22: That's a a good thing to be.

172

00:17:56.950 --> 00:17:59.739

PT22: At least it's a little bit more objectifiable.

173

00:18:06.360 --> 00:18:07.040

NM: Okay.

174

00:18:08.260 --> 00:18:09.879

NM: And number 8.

175

00:18:10.000 --> 00:18:16.059

NM: How many days is your child run for 10 min or more? 0 not related at all up to 5 highly appropriate, and why

176

00:18:19.080 --> 00:18:20.450

PT22: I have

177

00:18:21.120 --> 00:18:24.569

PT22: one child that can run in a gay trainer.

178

00:18:24.710 --> 00:18:30.769

PT22: It's not pretty, and she can't do it for 10 min, so i'm gonna give it

179

00:18:31.850 --> 00:18:34.740

PT22: a 3, because I think

180

00:18:37.310 --> 00:18:41.020

PT22: I think for the level 4 and 5 kids

181

00:18:41.080 --> 00:18:44.189

PT22: running for 10 min even adaptively.

182

00:18:45.040 --> 00:18:47.769

PT22: It's hard for most of our kids, so

183

00:18:48.380 --> 00:18:52.090

PT22: I don't know how practical it is to use that so much in an assessment.

184

00:18:53.810 --> 00:18:58.320

PT22: and there are a few kids that can do it. But i'd say the majority of my case, though right now

185

00:18:58.380 --> 00:18:59.150

PT22: cannot.

186

00:18:59.750 --> 00:19:02.250

NM: Yeah, and they wouldn't need the that's right.

187

00:19:02.420 --> 00:19:03.040

PT22: right?

188

00:19:04.960 --> 00:19:06.120

NM: Okay.

189

00:19:06.330 --> 00:19:17.999

NM: All right. So we are at the end. I ask every therapist to give me some final comments and thoughts about physical activity in this population. Any closing remarks as we wrap up.

190

00:19:20.350 --> 00:19:24.040

PT22: I love that you're doing this. I think I just was at.

191

00:19:24.280 --> 00:19:28.269

PT22: of course, this summer, and it talked an awful lot about

192

00:19:28.410 --> 00:19:31.819

PT22: fun and how to keep fun

193

00:19:31.990 --> 00:19:33.990

PT22: involved for our kids.

194

00:19:35.050 --> 00:19:41.910

PT22: I guess as you move forward, we'd love to stay involved with you as you come up with more studies, and

195

00:19:41.930 --> 00:19:42.930

PT22: and

196

00:19:43.060 --> 00:19:49.620

PT22: if you end up trying any of these to do an assessment on. I know I would love to be part of it

197

00:19:49.710 --> 00:19:55.349

PT22: because it's not actually something that's looked at Very well. So

198

00:19:55.770 --> 00:19:59.429

PT22: yeah, there's more. But we'd love to stay involved with you.

199

00:19:59.510 --> 00:20:01.670

because this is really cool

200

00:20:03.050 --> 00:20:04.639

and it's much needed.

201

00:20:05.840 --> 00:20:07.609

PT22: I think those are my finals.

202

00:20:08.250 --> 00:20:20.340

NM: Well, I really appreciate that. Thank you. I am humbled at that because it is important, and I agree with you, and I just want to thank you for your involvement. I'm going to stop the recording right now. Hold on a second.
