# Supplementary material for: Validity of PROMIS® Pediatric Physical Activity Parent Proxy Short Form Scale as a Physical Activity Measure for Children with Cerebral Palsy Who Are Non-Ambulatory
Source: Behav Sci (Basel). 2025 Jul 31;15(8):1042. doi: 10.3390/bs15081042 (PMC12382615; doi:10.3390/bs15081042)
Supplement: Supplementary file 1 [file behavsci-15-01042-s001.zip › Transcripts copy/PT transcripts - deidentified/PT14.docx]

WEBVTT

1

00:00:02.890 --> 00:00:28.489

NM: All right, awesome, Dr. PT14. Thank you for joining us today. Today. We're going to talk a little bit about physical activity in children that have Cp. That are not ambulatory. There is no right or wrong answer. I do have a set of questions, and so, if I sound scripted this because I am so that I could be consistent with a questioning. I do have some follow ups and prompts after each question, and and again the second half. We will talk about a survey for children

2

00:00:28.500 --> 00:00:37.360

NM: with how we would rate his collectivity, intensity, virtual name? Cp: okay. So first part, how do you define

3

00:00:37.630 --> 00:00:42.280

NM: physical activity for children with Cp Who are not full time walkers.

4

00:00:45.240 --> 00:00:53.359

PT14: So I would think of this as any type of movement that they can do. You know

5

00:00:53.480 --> 00:01:06.710

PT14: whether it's assisted by when they're well it. I guess it could be upright, or, you know, in a a supported position, laying down. But you know we need it

6

00:01:07.260 --> 00:01:12.089

PT14: whenever there's movement. You know where they are

7

00:01:12.190 --> 00:01:17.770

PT14: moving, and perhaps the respiratory rate is increasing. You know that they are

8

00:01:17.800 --> 00:01:22.179

PT14: being physically active, you know.

9

00:01:22.610 --> 00:01:31.729

PT14: So yeah. So I, you know, increase in respiratory rate and with the combination of of body movement.

10

00:01:34.110 --> 00:01:43.149

NM: Great. Thank you. So my first Prompt. The Department of health defines physical activities. Any activity that compasses if you expand it, and activation of skeletal muscle.

11

00:01:43.240 --> 00:01:48.660

PT14: Is this definition: Change your mind about how you define their activity. So you one more time. What do they say?

12

00:01:48.850 --> 00:02:00.330

NM: The department of Health defines physical activity as any activity that encompasses energy expended, and activation of skeletal muscle. Does this definition change your mind about how you define physical activity?

13

00:02:00.640 --> 00:02:01.320

PT14: No.

14

00:02:01.420 --> 00:02:05.450

NM: And how do you think physical activity

15

00:02:06.080 --> 00:02:09.830

NM: differs from other types of fitness activities.

16

00:02:13.890 --> 00:02:30.240

PT14: other types of fitness activities like, I guess sometimes we think of other fitness activities, something being organized, you know, going to an aquatic session or going to going, biking, or whatever it might be so.

17

00:02:30.340 --> 00:02:32.719

PT14: And how does that compare is that

18

00:02:34.670 --> 00:02:39.339

PT14: it may not be as social.

19

00:02:39.660 --> 00:02:48.379

PT14: you know, as other fitness activities, Unfortunately. It may.

20

00:02:49.650 --> 00:03:02.180

PT14: It's it's it's it's still, you know, energy expenditure, regardless of of If i'm thinking of, you know fitness activities being something more organized.

21

00:03:03.960 --> 00:03:14.240

PT14: And yeah, you know, they're not. Just. These children are aren;’t walking. And so we do think of a lot of the things that we need to to be able to walk.

22

00:03:14.410 --> 00:03:15.410

NM: Hmm.

23

00:03:15.750 --> 00:03:16.580

NM: Yeah.

24

00:03:19.100 --> 00:03:26.809

NM: And when do you witness your students? The patients participate most in physical activity during the school day or during the the

25

00:03:27.240 --> 00:03:28.540

NM: the day hours.

26

00:03:29.930 --> 00:03:30.860

PT14: Well.

27

00:03:30.870 --> 00:03:58.080

PT14: so i'm not in the school system, but I I am in outpatient. So I whenever they come outpatient, they they, you know. I I definitely think they are being challenged in physical therapy sessions, and potentially, you know, when they are have play time, you know, recess and hopefully physical education, You know, gym class. You know whether we're being more physically active.

28

00:03:58.690 --> 00:04:18.370

PT14: you know it depends on you know how they train or how they get to school, you know it could be in the transportation to you know that they may be more physically active if they have to be upright, and so so that's possible. As well. But yeah, I would say, you know those, those

29

00:04:18.380 --> 00:04:19.320

PT14: there would be therapy,

30

00:04:19.510 --> 00:04:27.689

PT14: physical education, class recess, and possibly in the modest transportation to and from different places.

31

00:04:28.770 --> 00:04:30.129

NM: Thank you so much.

32

00:04:30.200 --> 00:04:31.670

Next question.

33

00:04:31.880 --> 00:04:37.649

NM: How do you measure physical activity, frequency, intensity, time and type?

34

00:04:38.030 --> 00:04:45.430

NM: And that's like the fitt principle there in children with Cp. Who are not full time walkers, and i'll say it again. How would you measure

35

00:04:46.020 --> 00:04:53.000

NM: physical activity, frequency, intensity, time and type, and children with Cp. Who are not full time, walkers.

36

00:04:55.940 --> 00:05:00.199

PT14: so i'll start with

37

00:05:00.210 --> 00:05:24.200

PT14: intensity in that, you know. That would be. You know how how exerted they show me that they are, you know. Are they able to talk to me, you know? Are they able to look at me, you know? Look at me, and you know so so visual eye contact and and and and you know, verbal skills if they have them, you know.

38

00:05:24.210 --> 00:05:30.630

PT14: as along with the level of their respiration, you know, this is how I would really their intensity

39

00:05:32.030 --> 00:05:46.319

PT14: frequency I looked at, as you know. So you know, we like to think of, you know, 30 to 60 min, and of of you know.

40

00:05:47.180 --> 00:05:54.449

PT14: I, I say, like it extremely extreme, but more significant

41

00:05:54.460 --> 00:06:10.390

PT14: exertion, you know. You know, however, many times per week should be, you know, 4 or 5 times per week. So you want these kids to be, you know, really active, for you know, 30 to 60 min, 5 times a week.

42

00:06:10.400 --> 00:06:20.839

PT14: So i'm looking at frequency, as I guess, times per week and time and type of time is how much throughout the day. You know how many you know.

43

00:06:20.960 --> 00:06:27.529

PT14: If it's minutes, or if it's hours per day and type

44

00:06:27.580 --> 00:06:28.740

PT14: and

45

00:06:29.290 --> 00:06:30.460

PT14: type.

46

00:06:31.040 --> 00:06:48.399

PT14: I guess I think of type, as you know, depending on the activity that they're doing. Is it part of their, you know, ADLs, you know. Is it part of their getting dressed in the morning? And

47

00:06:48.480 --> 00:07:04.410

PT14: you know just you know again that that the whole mode of transportation, you know, interacting with other people. You know what we're doing during feeding times, you know. So that's sort of like could be potentially.

48

00:07:04.720 --> 00:07:11.029

PT14: We would think of as a lower level. But some could be for these kids more physically active, you know it

49

00:07:11.140 --> 00:07:14.759

PT14: to really be a higher level. So he.

50

00:07:15.250 --> 00:07:23.350

PT14: even though that I guess the type that's that's tough. I don't know. If you know.

51

00:07:24.270 --> 00:07:26.859

PT14: I don't I I

52

00:07:26.870 --> 00:07:54.539

PT14: having a hard time with that one.

NM: That's okay. That's okay. This is just this is just kind of brainstorming chatting like that's that's perfect. Yeah, I think you did a great explanation. If you come up with something else, feel free to share later.

PT14: That's I would just break it up again. And so, you know, is it causing them less or more visible exertion. Is it causing them to be very much, you know, because it's something that they are able to at all.

53

00:07:55.300 --> 00:07:57.049

NM: perfect? Thank you so much.

54

00:07:57.110 --> 00:08:08.060

NM: Do these children, and you get some nice examples. Do they need exam assistance to complete complete these activities? And if so, which activity do they need a assistance

55

00:08:09.260 --> 00:08:28.650

PT14: with. Yes, they need assistance with it's like if we're talking about, you know, acting like that activities of daily living in terms of Yes, they need to help with dressing they may need help with the feeding. They need help. You know. like with a helper, you know.

56

00:08:28.680 --> 00:08:43.849

PT14: to sit with them. If they're how they're being transported, you know, to in some, you know. And then, of course, not playing around in a recess. And yes, I mean, I would say it assistance with all activities

57

00:08:46.180 --> 00:08:54.480

NM: and with all of the activities they need assistance. Do they need it for the entire task, or sometimes part of the task? And if you have an example of each, that would be great.

58

00:08:54.630 --> 00:09:03.840

PT14: Yeah. So part of the task. So, for instance, they may need help with you know the

59

00:09:04.740 --> 00:09:30.609

PT14: it. I guess it depends on on the patient, but but in terms of dressing they may be able to, you know, with their arms to get them through the shirt, but need help with, you know. Actually, you know, stabilizing the trunk, to pull it over, you know. So over the same thing, you know, with the socks and shoes, you know they may need

60

00:09:30.850 --> 00:09:37.679

PT14: help in terms of positioning them to get. You know things in the right spot, but then they can, you know.

61

00:09:37.800 --> 00:09:40.099

finish finish the task.

62

00:09:40.750 --> 00:09:43.719

PT14: So yes, not not necessarily the whole task.

63

00:09:43.780 --> 00:09:44.380

No.

64

00:09:45.890 --> 00:09:52.160

NM: thank you. And do you think they should participate more or less in each of these activities. And why?

65

00:09:52.740 --> 00:10:10.340

PT14: Yeah, I think for as much as they can do on their own. We want them to. It's difficult, obviously, because it takes more time and patience for the caretaker, and you know, like just the biggest thing is usually time, you know. You have to get ready so much earlier to go places.

66

00:10:10.350 --> 00:10:28.970

PT14: But I definitely think that this helps children, you know, in the long run, because then they are being, you know, more enabled to be able to compute those tasks by themselves, and then, hopefully, you know, continue that on, you know, in their lifetime.

67

00:10:31.340 --> 00:10:32.910

NM: Great. Thank you

68

00:10:33.490 --> 00:10:35.329

NM: all right next question.

69

00:10:35.550 --> 00:10:39.849

NM: Do you address promoting physical activity during your PT Sessions?

70

00:10:40.520 --> 00:10:46.139

PT14: Yes, yes, definitely. You know as much as possible. Get up

71

00:10:46.330 --> 00:11:04.979

PT14: out of whatever device we're in, you know. Or maybe you know. Yeah, we try and get up. We'd be we upright, and whatever it is that we can do, you know, I used to be.

72

00:11:04.990 --> 00:11:08.700

PT14: Gait trainers, to help them walk

73

00:11:08.760 --> 00:11:10.060

PT14: you know.

74

00:11:10.330 --> 00:11:19.059

PT14: Yeah, just going back to the question. Yes, but definitely trying to promote them to to be upright, to to to get up and do activity during during a session

75

00:11:19.430 --> 00:11:40.039

NM: great. And so you already gave me how you do this, so that's great out my next question. So you mentioned the gate trainers. What components of physical activity do you think you address? And I can give you some examples? Are you mostly addressing cardiovascular endurance or or muscle activation, mobility. You know there's so many things you can work on. But like, what are the things you feel like? You mostly address.

76

00:11:40.050 --> 00:12:00.320

PT14: Yeah, I would say cardiovascular is is is probably number one on the list. And you know, I do think that that's super important because they we want them, To be strong. You know these are kids who, when they do get sick they can get very sick. You know it's harder.

77

00:12:00.330 --> 00:12:06.820

PT14: They don't have that, you know the fitness to be able to to get up and and and and you know.

78

00:12:07.990 --> 00:12:35.770

PT14: you know, use their their muscles and and and and and and and and stay strong. So when they do get sick and you know it, I think it does hit them harder, and they, their their cardiovascular is is is weak also, you know, they all this respiratory muscles so definitely no cardiovascular, but also, yes, you know, in terms of muscular skeletal making sure that we're not getting any contractures, that we

79

00:12:35.790 --> 00:12:48.279

PT14: you might already be present, you know, trying to keep whatever range of motion we have, and strength that we have, and continuing to strengthen to make stronger as much as we can.

80

00:12:49.540 --> 00:12:51.000

PT14: with Mobility Yes.

81

00:12:51.190 --> 00:12:53.180

NM: right, right, great great.

82

00:12:53.320 --> 00:12:54.710

NM: And

83

00:12:55.460 --> 00:13:04.189

NM: do you address promoting physical activity that occurs outside of physical therapy sessions.

84

00:13:04.200 --> 00:13:27.539

PT14: The parents are generally so good with wanting to do things to so so so yes, you know, trying to get them involved in whatever you know the child may show interested and just with the family, you know. Whatever the family activities are, one child to participate in them as well. You know, to the extent that they're able, involving them in all you know.

85

00:13:27.550 --> 00:13:40.930

PT14: the family activities is super important, you know. And so yeah, so, and I definitely do that, You know, Throughout their sessions I would say, that's one of another big part is family education.

86

00:13:41.050 --> 00:13:51.179

PT14: you know. Just making sure that that um. You know they are doing things outside of PT sessions and and and being involved with the family as much as possible.

87

00:13:52.530 --> 00:13:53.510

NM: That's great.

88

00:13:53.570 --> 00:13:59.029

NM: And Have you recommended any community programs or events to your students to help increase? PA:

89

00:13:59.040 --> 00:14:26.689

PT14: Yeah. So we have some like like a buddy... There was a buddy program at one time that I, you know, recommended for a child. You know where another you know more physically able body-Child, will assist another child who is not, you know, as abled body, to to to, you know, participate in in order to organize activity, Whether it's, you know.

90

00:14:27.100 --> 00:14:40.979

PT14: basketball, or or what what have you so so that, and you know, depending on the child, and and and if they show interest in an activity I can just

91

00:14:40.990 --> 00:15:00.459

PT14: recommended. Parent and and I have also recommended aquatic therapy for some children. But yeah, if they're interested in somebody, they show interest in something that they like, You know, I I I encourage the parent to then, you know, pursue that as a possibility for this, for this you know.

92

00:15:00.470 --> 00:15:01.270

PT14: child

93

00:15:03.110 --> 00:15:15.850

NM: perfect. Thank you. And have you rec..in terms of equipment. What type of equipment have you recommended to helping improve home and or community engagement of physical activity outside of the clinical setting

94

00:15:16.460 --> 00:15:26.899

PT14 : you're you're not looking for like specific types, are you? Or brands any?

NM: No, it doesn't matter whatever you've used, and you can give me a brand. You Can you just tell me what you typically recommend it for

95

00:15:26.910 --> 00:15:38.819

NM: to help improve home and or community engagement outside of the clinical setting?

96

00:15:38.960 --> 00:15:52.359

PT14: So I mean, Wheelchairs, adaptive wheelchairs, Custom, Not just your standard hospital wheelchair, but you know I do recommend those for for children who are not ambulatory, when they go out to be as upright as much as possible, and with the environment.

97

00:15:52.370 --> 00:16:02.840

PT14: And then also, when we're able to, you know, get into a like a gait trainer or like Rifton pacer, I use that. You know where they're supported. They can be supported

98

00:16:02.990 --> 00:16:20.450

PT14: as much as you really need, you know. And the trunk, then you know that's nice, because then the parent can kind of help to to, you know. Push that along as well. So so those are probably the bigger ones that I recommended for community use

99

00:16:20.460 --> 00:16:35.220

PT14: parents do often look for. You know some type of bathing, you know, system like something they can use in bathroom, and that's more individualized, you know. But yeah, they need help with that, too. You know it's been to be safe

100

00:16:35.230 --> 00:16:45.740

PT14: and comfortable in the bathtub, you know. So that is something that I don't remember the specific type that we I looked at with other things as well.

101

00:16:46.720 --> 00:16:50.900

NM: Thank you so much. All right. Now. We're trying to look at the survey. We've got to the second

102

00:16:51.120 --> 00:16:56.000

NM: part, where I will show you. This is called the Promise

103

00:16:57.130 --> 00:17:11.549

NM: surveys the parent Proxy, physical activity, survey, and this was created by the the National Institute of Health, and, as you can see, this was created for children that we're not typically developing, or had going through some kind of

104

00:17:11.560 --> 00:17:35.359

NM: progressive disorder, so the parent will fill this out for caregiver, and it's a physical activity survey. So about how they're starting themselves throughout the week. And so i'm asking therapists and eventually parents, how they would rate each question about how appropriate it would be for this population, Specifically, children with Cp. And Levels and 5. So how appropriate valid?

105

00:17:35.370 --> 00:17:47.230

NM: Would you say this assessment is so? I'm going to ask you about each questions. We'll take a second to look at that. So based on each question, i'm going to ask you how appropriate is a question to addressing physical activity, intensity

106

00:17:47.240 --> 00:18:00.429

NM: in the population we're talking about, and 0 is not related at all. 5 is highly appropriate, and I'm going to ask you to give me a range along that scale which, including 0 to 5. But how would you rate the question?

107

00:18:00.470 --> 00:18:02.019

NM: So for the first question.

108

00:18:02.420 --> 00:18:13.829

NM: How many days did your child exercise a place so hard that his or her body got tired? Would you rate this not related at all? 0, 5 highly appropriate, and somewhere between. And why?

109

00:18:16.030 --> 00:18:28.199

PT14: So I would read it in between. I it. I would put it at a 3. Don't like, yeah, the the the exercise. Your play so hard is the tricky

110

00:18:28.230 --> 00:18:34.610

PT14: to be part. You know, that that might be confusing for parents to that with where our end of the point.

111

00:18:34.860 --> 00:18:35.580

NM: Okay

112

00:18:37.170 --> 00:18:38.620

NM: and tricky, because.

113

00:18:39.470 --> 00:18:50.629

PT14: like, yeah, we want to know if they got tired, but we might freeze it differently. How many does your child move so so much that because when our body got tired.

114

00:18:51.760 --> 00:19:07.820

PT14: okay, like it's a it's a good question. This is the the exercise you can so hard. Maybe it may not thank you, but you don't it's hard to say right parents. But but yeah, it's like that's where I think some some parents, maybe

115

00:19:07.930 --> 00:19:11.180

PT14: I think that that question is geared towards more

116

00:19:11.400 --> 00:19:13.870

NM: Gotcha.

117

00:19:13.910 --> 00:19:19.010

NM: All right, Number 2. How many days did your child exercise really hard for 10 min or more?

118

00:19:19.100 --> 00:19:29.289

NM: How appropriate. Would you say this 0 not related at all? 5 highly appropriate in this population? Somewhere between. And why again, 3 is the exercise part

119

00:19:33.030 --> 00:19:34.800

PT14: I would change the exercise.

120

00:19:34.910 --> 00:19:35.850

NM: We Gotcha

121

00:19:35.920 --> 00:19:37.399

NM: all right. Number 3.

122

00:19:37.460 --> 00:19:41.570

NM: How many days did your child exercise so much that he or she breathed hard

123

00:19:42.180 --> 00:19:46.100

PT14: again. 3 great change the exercise.

124

00:19:50.900 --> 00:20:03.309

NM: Do you feel like parents would take that word wrong like in a sensitive? Could it be something insensitive? Okay, I don't want for words. Your mouth. But I I I kind of got that just from the

125

00:20:14.540 --> 00:20:16.809

NM: all right. Number 4.

126

00:20:16.980 --> 00:20:27.159

NM: How many days was your child so physically active that he or she sweated. How appropriate would you think that one is for this population? Would you say 0 not related at all?

127

00:20:27.710 --> 00:20:29.710

5 highly appropriate.

128

00:20:29.740 --> 00:20:32.720

PT14: or somewhere between? And Why, I would say 5.

129

00:20:32.820 --> 00:20:33.910

NM: Okay? Why.

130

00:20:35.000 --> 00:20:37.169

PT14: the physically active makes it

131

00:20:37.490 --> 00:20:40.470

PT14: sound more. Possibly.

132

00:20:40.500 --> 00:20:49.080

PT14: you know, appropriate for this population. And really they sweat. And you know.

133

00:20:56.590 --> 00:20:58.279

NM: and for the sweating component.

134

00:20:58.550 --> 00:20:59.470

PT14: Yes.

135

00:20:59.490 --> 00:21:00.180

NM: okay.

136

00:21:00.370 --> 00:21:04.339

NM: Number 5. How many days did your child

137

00:21:04.440 --> 00:21:08.099

NM: exercise of our place so hard that his or her muscles?

138

00:21:08.180 --> 00:21:11.360

NM: How appropriate Would you think that one would be 0 to 5?

139

00:21:11.480 --> 00:21:12.340

NM: And why

140

00:21:14.480 --> 00:21:20.859

PT14: that I would put down sort of to the if

141

00:21:21.410 --> 00:21:32.890

PT14: the child may not be able to describe. You know that their muscles burn. They may say that they have pain, you know, and I think it would be just be changing the exercise and also changing the muscles. And

142

00:21:45.510 --> 00:21:53.709

NM: right Number 6. How many days did your child exercise or play so hard that he or she felt tired?

143

00:21:54.490 --> 00:21:58.630

PT14: How many?

144

00:21:59.020 --> 00:22:03.849

PT14: And he felt tired, so

145

00:22:05.920 --> 00:22:08.750

PT14: that that's the one

146

00:22:08.930 --> 00:22:22.660

PT14: you know. I I put it in a 3, the exercise your place so hard, but also the felt tired and dependent upon how they can describe their feelings, how they can describe

147

00:22:22.670 --> 00:22:37.309

PT14: how they feel more. So you know i'm sure they're tired, you know, with with most physical activity. So being able to, you know, differentiate that they feel more tired. Or you know that that's difficult.

148

00:22:37.650 --> 00:22:47.650

NM: Yeah. And the parent would be the one assessing this like apparently determine it. So yeah, these are the questions asked to a parent. So a child wouldn't necessarily be answering. But

149

00:22:48.060 --> 00:22:57.780

PT14: so, looking at it from a parent perspective. Okay. So yeah. So I see what you mean, right? Right? So for the parents to be able to.

150

00:22:58.960 --> 00:23:02.779

I put it in 3. It you know the

151

00:23:02.970 --> 00:23:12.470

PT14: I do think that parents are very perceptive, you know, and so they would be able to tell their child. Tired, you know they?

152

00:23:12.600 --> 00:23:15.619

PT14: I think so. I think so. They they'd be able to know that.

153

00:23:15.880 --> 00:23:16.620

NM: Okay.

154

00:23:16.640 --> 00:23:20.080

NM: All right. Great Number 7,

155

00:23:20.390 --> 00:23:32.289

NM: And you still score that into 3, right Number 7. How many days was your child physically active for 10 min or more? How appropriate would that be? 0? Not at all for this population 5 highly appropriate.

156

00:23:33.120 --> 00:23:36.430

PT14: I put it at a at a 5.

157

00:23:36.630 --> 00:23:38.110

NM: Okay? Why.

158

00:23:38.450 --> 00:23:53.109

PT14: I think it's a good question. Yeah, I think parents would be able to to be able to answer that, and you know easily know that they would be able to say, yeah, they were.

159

00:23:55.140 --> 00:23:59.999

PT14: you know.

160

00:24:00.180 --> 00:24:02.940

PT14: Well, every day, or whatever it might be

161

00:24:04.080 --> 00:24:07.280

NM: okay. And then number 8

162

00:24:07.560 --> 00:24:14.179

NM: last one. How many days did you travel on for 10 min or more? How appropriate with this question be for this population 0. Not at all.

163

00:24:14.330 --> 00:24:17.849

PT14: 5 highly appropriate.

164

00:24:18.260 --> 00:24:20.910

NM: since they're not running.

165

00:24:21.310 --> 00:24:24.800

PT14: If I anything anything else on that one.

166

00:24:24.840 --> 00:24:38.319

PT14: Nope. If I could go back 1, 2, 3, 4 to number 4, where they sweated. I I think about with the children in this population, I guess what i'm thinking of is they get hot, and they they do do

167

00:24:38.330 --> 00:24:48.979

PT14: their clothes do get wet, you know, and I, and it's not necessarily that they're necessarily perspiring. But so that's why I thought that that was appropriate. But I can see how

168

00:24:49.460 --> 00:24:51.060

PT14: it you know.

169

00:24:51.100 --> 00:25:18.430

PT14: You know this sweating aspect is, we is, you know, correlated with higher levels of physical, and then maybe running. But it that's why I kind of. I thought that it was appropriate. But then now i'm thinking of, maybe because of the population, right? Because sometimes yeah, I see what you're saying. Yeah. Yeah. Yeah. So would you like to change your question? Your answer, Change it back to the let's get. I think I put it on a forward.

170

00:25:18.440 --> 00:25:19.499

NM: You gave it a 5.

171

00:25:19.560 --> 00:25:35.759

PT14: I'll give you the 5. Let me go down to my to 3, because you don't think, because sometimes they may not proceed. I think of sweating. As you know, the clothes are well like they're hot, you know. They've been in this chair for how long? And you know.

172

00:25:35.770 --> 00:25:41.280

PT14: but they mean parents may not think of whether it is that way.

173

00:25:41.810 --> 00:25:42.880

NM: So

174

00:25:43.310 --> 00:25:49.960

NM: because these kids sweat that because whether they're physical at you or not, is that what you're kind of leaning towards?

175

00:25:50.500 --> 00:25:54.160

PT14: Yeah, They right? Exactly. Exactly. Okay.

176

00:25:57.610 --> 00:26:00.260

NM: So I put that down to 3

177

00:26:00.380 --> 00:26:01.420

PT14: Right?

178

00:26:05.020 --> 00:26:05.880

Hmm.

179

00:26:11.850 --> 00:26:26.960

NM: Alright, now we're at the end. So what I typically ask is we, we, we conclude, is for any final thoughts that you may have about this population and any polling thoughts you want to say about. You know your just thoughts about physical activity in this population.

180

00:26:28.780 --> 00:26:46.610

PT14: So I think this is great this is, I mean, a great. It seems like a great project, you know. Just it seems. It is really good information, you know, to be able to use this especially for parents benefit, you know, to be able to feel included. I think that's the biggest problem is that

181

00:26:47.040 --> 00:27:05.860

PT14: you know their lives change so much when you have a child with disability, and it's just so hard. You know you. I think parents just always want to be, you know, really a part of whatever it is that every other child is doing right. It makes me emotional. So I think about that.

182

00:27:06.660 --> 00:27:09.530

PT14: And so I think it's great. You know this would be such good information.

183

00:27:09.680 --> 00:27:16.319

NM: Thank you so much, that girl Cool! Stop it, don't make me cry. I I really appreciate your time. So i'm going to stop the recording. Thank you.
