# Supplementary material for: Validity of PROMIS® Pediatric Physical Activity Parent Proxy Short Form Scale as a Physical Activity Measure for Children with Cerebral Palsy Who Are Non-Ambulatory
Source: Behav Sci (Basel). 2025 Jul 31;15(8):1042. doi: 10.3390/bs15081042 (PMC12382615; doi:10.3390/bs15081042)
Supplement: Supplementary file 1 [file behavsci-15-01042-s001.zip › Transcripts copy/PT transcripts - deidentified/PT8.docx]

WEBVTT

1

00:00:03.480 --> 00:00:04.440

PT8: Okay.

2

00:00:04.450 --> 00:00:30.770

NM: all right. Thank you so much for joining us today. this interview is specifically about physical activity. and children are not full time. Walkers who have some little calls, and so understand that there are some times where we don't even have a diagnosis of a cure poly depending on the age so ideally just working with individuals that have brain based disorders that are not ambulatory. So my first question

3

00:00:31.310 --> 00:00:36.679

NM: is, how do you define physical activity for children with Cp. Who are not full time walkers.

4

00:00:38.490 --> 00:00:39.780

PT8: Do I define it?

5

00:00:40.760 --> 00:00:45.620

PT8: Any kind of active movement that they are doing

6

00:00:51.670 --> 00:00:56.319

NM: great. So anytime of active move, mo mo movement. You mentioned

7

00:00:57.180 --> 00:00:59.320

NM: anything additional you want to add.

8

00:01:03.150 --> 00:01:09.139

PT8: I mean No, I I I I I guess I don't understand fully the question. That's you know, like

9

00:01:09.590 --> 00:01:27.150

NM: that's totally fine. Let me give you I have a couple of prompts for each question, and i'll I know you saw. I try to stick to the script. But the first prompt is the Department of Health defines physical activity as any activity that encompasses energy expended, and activation of skeletal muscle. Does it

10

00:01:27.160 --> 00:01:33.889

NM: definitely? If you change your mind about PA, it's kind of what you already said.

PT8: No, because it's any active movement that the child is doing

11

00:01:33.930 --> 00:01:42.089

PT8: so. I'm. not including movement that I am doing to that child, but active movement that they are doing.

12

00:01:44.750 --> 00:01:50.020

NM: Thank you. And then how do you think physical activity differs from other types of fitness activities?

13

00:01:50.770 --> 00:01:53.610

PT8: I I don't think it doesn't it. Does

14

00:01:54.080 --> 00:01:58.800

PT8: you? You think that's the just only in this population, or just in general in general?

15

00:02:03.990 --> 00:02:22.300

NM: And what when do you witness your students or your children that you treat participating most during physical activity during the school day. Like, if you have a child, that you see that maybe in school, when do they have the most physical activity.

PT8: Okay, so we don't have a a a PE session

16

00:02:22.310 --> 00:02:31.680

PT8: so that is considered the physical therapy. So I would say. Probably they are most active in our physical therapy sessions.

17

00:02:31.700 --> 00:02:35.949

PT8: And then and then going to and from school.

18

00:02:36.830 --> 00:02:47.100

PT8: Okay, interesting. Can you give me a little bit more of an example you said, going to and from school. I currently don't have any children at the

19

00:02:48.220 --> 00:02:54.220

PT8: levels 4 and 5 so my children are all

20

00:02:54.400 --> 00:02:57.290

PT8: ambulatory during the school day.

21

00:02:58.140 --> 00:03:10.160

NM: but from your experience, when you say going to and from school, do you mean just getting on the bus or just getting ready the preparation back back from the therapy area to the school. Oh, okay, that's what I was trying to understand.

22

00:03:12.510 --> 00:03:13.310

NM: Got it.

23

00:03:14.410 --> 00:03:15.649

NM: Okay. Next question.

24

00:03:15.900 --> 00:03:26.179

NM: How do you measure physical activity, frequency, intensity, time and type and children with Cp. Who are not full time walkers. And that's like i'm citing them fit principle there.

25

00:03:29.460 --> 00:03:33.770

PT8: It would depend on the activity that the child is doing

26

00:03:33.840 --> 00:03:36.670

PT8: so. and then

27

00:03:36.700 --> 00:03:40.250

PT8: I would if I needed to.

28

00:03:42.050 --> 00:03:44.390

PT8: If I was looking at.

29

00:03:44.480 --> 00:03:58.609

PT8: say, for example, if I wanted to see endurance and what they were able to do, I might look at how fast they could roll from one side of their living room to the other side.

30

00:03:58.700 --> 00:04:02.690

PT8: and if they could do it continually throughout the day.

31

00:04:04.920 --> 00:04:09.139

NM: Okay, Great. So you're looking at just to help me kind of

32

00:04:09.320 --> 00:04:10.020

NM: Oh.

33

00:04:10.140 --> 00:04:17.130

NM: organize this. You mean, like more like the frequency of it a task that you're working on, and your piece frequency the time.

34

00:04:17.220 --> 00:04:31.389

PT8: and how often they the not just like they did it 5 times in a row. But how many times during the day, so the duration, frequency, duration and so i'm getting an intensity

35

00:04:31.580 --> 00:04:33.430

PT8: of their activity.

36

00:04:34.570 --> 00:04:36.040

NM: Thank you. That's great.

37

00:04:36.540 --> 00:04:55.309

PT8: And do they typically need assistance to complete these type of activities? So you mentioned rolling? That would be an example of one of the activities you may for some chat children, if they needed to initiate or complete the movement. But here I wouldn't count it for physical activity.

38

00:04:55.320 --> 00:05:00.870

PT8: if every if it was the child being moved by another person who space

39

00:05:06.080 --> 00:05:07.550

NM: and

40

00:05:07.580 --> 00:05:17.029

NM: So we talked about rolling. Is it any of the activities where they may need some assistance to get started, and then do they need assistance for the whole task with some of these kids, or just part of it?

41

00:05:18.390 --> 00:05:37.090

PT8: it it could be either or so pushing up and lifting their head up against pro against gravity. I would not think of driving a powered chair as physical activity because they are moving through space. But it's not really They wouldn't be a real

42

00:05:37.110 --> 00:05:39.229

PT8: looking at

43

00:05:40.020 --> 00:05:44.899

PT8: you know. I would think of that as more of a transportation.

44

00:05:48.770 --> 00:05:55.989

NM: And do you think they should participate more or less in each of the activities that you you deemed physical activity? And why

45

00:05:56.720 --> 00:05:59.870

PT8: it depends on

46

00:06:00.300 --> 00:06:06.290

PT8: If if if it's something that will not

47

00:06:06.970 --> 00:06:13.940

PT8: be deleterious in the long run. So if they, for example, If their rolling was incredibly asymmetrical.

48

00:06:14.100 --> 00:06:28.489

PT8: and doing that more on their own would cause them to have an orthopedic deformity, then I would want to have make sure that whatever we encourage the family and the child to be doing

49

00:06:28.530 --> 00:06:33.309

PT8: would be something that didn't end up being deleterious in the long run.

50

00:06:34.330 --> 00:06:44.050

NM: Thank you. All right. Third Question: Moving right along. Do you address promoting physical activity during your actual physical therapy sessions? And it's so. How

51

00:06:45.660 --> 00:06:47.700

PT8: I believe that.

52

00:06:47.930 --> 00:06:56.760

PT8: Any movement that the child does actively helps to help with with motor learning, and helps them to

53

00:06:56.920 --> 00:07:07.400

PT8: to engage and to try to do more. So. I definitely do so, even if the person is only able to move

54

00:07:07.450 --> 00:07:17.299

PT8: a partial range through on their own. I will have them do that, and then I might take them through the rest of that range

55

00:07:17.920 --> 00:07:21.319

PT8: to make sure that they remain that mobility.

56

00:07:27.330 --> 00:07:44.509

PT8: I hope i'm understanding your questions correct.

NM: No, you doing. You're doing great, I think. what I wanted to in terms of a follow up. I was hoping to get a little bit more examples like is it, during actually just like passive or active range of motion, to do a task, or is it to engage in some in some sort of…

57

00:07:44.800 --> 00:07:59.820

PT8: trying to find what is motivating to them for play. That's something that you know that they can interact with, or that I can give the the family to to do, because basically I find that

58

00:07:59.920 --> 00:08:03.999

PT8: a lot of the children who are more significantly involved.

59

00:08:04.060 --> 00:08:09.860

PT8: you know, building on some of the beginning movements or beginning interests.

60

00:08:09.910 --> 00:08:11.939

PT8: pays off in the long run.

61

00:08:15.630 --> 00:08:34.919

NM: That's great, Thank you. And so what components of the physical act of physical activity do you address? So let me give you an example. You've mentioned motor control? Some examples I have listed are cardiovascular endurance, muscle, activation, and energy expenditure. What are some of the components that you feel like you address

62

00:08:35.080 --> 00:08:47.869

PT8: now? Always Cardio pulm, because, if you can't breathe. He can't move. so anything that has to to to do with that is, very important.

63

00:08:49.320 --> 00:08:57.859

PT8: you know, activating whatever muscle groups we can in a way that that doesn't causes deleterious effect down the road.

64

00:09:00.650 --> 00:09:02.190

NM: Great and

65

00:09:02.260 --> 00:09:04.609

PT8: endurance it depends on.

66

00:09:05.590 --> 00:09:12.439

PT8: you know. If if the child has enough function to really, you know, carry out something.

67

00:09:12.490 --> 00:09:23.930

PT8: you know I have not really thought about measuring endurance, so it's tricky to measure endurance for all of our patients, anyway, so I usually try to do it as

68

00:09:24.140 --> 00:09:30.730

PT8: like. How many times somebody can complete something. So if this was somebody who could

69

00:09:30.750 --> 00:09:33.249

PT8: assistant, if its to Stand

70

00:09:33.280 --> 00:09:34.940

PT8: I might

71

00:09:35.100 --> 00:09:37.979

PT8: describe exactly how they were doing that

72

00:09:38.030 --> 00:09:39.079

PT8: And then

73

00:09:39.340 --> 00:09:41.729

PT8: how many times they could

74

00:09:41.770 --> 00:09:45.770

PT8: do it within a certain timeframe to measure kind of endurance.

75

00:09:48.810 --> 00:09:49.750

NM: Thank you.

76

00:09:49.980 --> 00:09:56.109

NM: all right. Fourth question. And then we're going to look at a survey that was created by the National Institute of Health.

77

00:09:56.290 --> 00:10:01.639

NM: Do you address promoting physical activity that occurs now outside of your Pt sessions?

78

00:10:03.670 --> 00:10:08.550

PT8: yes, absolutely. I I always try to find

79

00:10:08.600 --> 00:10:14.369

PT8: activities that first of all, I i'm looking more for it not

80

00:10:14.410 --> 00:10:16.329

PT8: to be exercise

81

00:10:16.610 --> 00:10:21.399

PT8: not to be something that their person is thinking about homework.

82

00:10:21.440 --> 00:10:22.940

PT8: but part of life.

83

00:10:23.000 --> 00:10:27.340

PT8: So for, and that's something. Maybe they can do all together as a family.

84

00:10:27.420 --> 00:10:32.390

PT8: So for me, I frequently talk about doing

85

00:10:32.560 --> 00:10:36.429

PT8: a bilateral coordination activities like climbing

86

00:10:36.530 --> 00:10:50.109

PT8: like anything that the family can be involved in together that doesn't make it like an extra separate time, because I feel like that's so important.

87

00:10:50.220 --> 00:10:52.580

PT8: for consistency, and carry over

88

00:10:54.310 --> 00:11:11.969

NM: excellent. Have you recommended any community programs or events to families. I have found that difficult in our area. the one contact, and it's it's funny, because that's one of the things that

89

00:11:12.060 --> 00:11:24.810

PT8: I had said after I finished. my degree, that i'm gonna do is I want to join it looked like there weren't any requirements to join the Wellness Council for the APTA.

90

00:11:24.840 --> 00:11:33.350

PT8: Also, I wanted to get more involved with that, and also thinking about community programs. There is one contact I had

91

00:11:33.400 --> 00:11:37.390

PT8: for ice hockey for New York City kids.

92

00:11:38.510 --> 00:11:57.590

PT8: But I have not been able to like like that would have been something like I would like to get involved in fine there was also another project that I don't know if it still exists. that it was all the way downtown that children could participate in for general movement.

93

00:11:57.600 --> 00:12:08.579

PT8: there have also been one or 2 programs in the westchester area that I don't have their names. but any time, if somebody tells me of something I try to

94

00:12:08.780 --> 00:12:14.599

PT8: jot it down but a lot of that now, since Covid I don't know what has resumed

95

00:12:15.870 --> 00:12:25.750

NM: absolutely and what types of equipment have you recommended to help improve home and or community engagement and physical activity outside of the

96

00:12:25.920 --> 00:12:34.009

PT8: well like since. If if there is a if the child has the potential to ride a bike

97

00:12:34.030 --> 00:12:46.040

PT8: like I we were like, or if we get an adapted bike donated that we can't use at our facility trying to see if there's a family who will use it.

98

00:12:46.080 --> 00:12:52.090

PT8: and get that that have that active, you know. Have it as a

99

00:12:53.200 --> 00:12:57.459

PT8: activity for them, so that I've worked on for bicycles

100

00:12:57.560 --> 00:13:01.539

PT8: both for upper extremity bikes and

101

00:13:01.630 --> 00:13:04.910

PT8: also lower extremities bikes.

102

00:13:06.140 --> 00:13:06.980

PT8: Then

103

00:13:07.750 --> 00:13:14.929

PT8: I would say that with it, as far as equipment goes, I I you know some of the

104

00:13:16.160 --> 00:13:26.970

PT8: other more. yeah, I I mean, other things would have been just simple things like if it was somebody who could do something with theraband that kind of stuff, you know, but other bigger items now

105

00:13:30.960 --> 00:13:35.300

NM: Great, All right. We're towards the second half. I'm going to pull up

106

00:13:35.430 --> 00:13:45.640

NM: a survey called the Promise Parent Oxy. Oh, okay. So you're familiar with this survey. Okay, great. So let me pull it up, and just so you can have a chance to look

107

00:13:45.740 --> 00:13:49.509

NM: at the survey. And so this was created

108

00:13:50.540 --> 00:14:03.899

NM: out of the desire to try to create a physical activity survey for children that may have a progressive disorder. I think it was oncology and some other

109

00:14:04.010 --> 00:14:22.079

NM: disorders. This has been looked at. so we're looking to see what you think and other pts. And then we're going to also look at interview parents about how appropriate this is for children that are not ambulatory. specifically 4 and 5 Gms: this level. So i'm going to ask you for each question Here.

110

00:14:22.420 --> 00:14:28.160

NM: i'm gonna ask you how appropriate is the question to addressing physical activity, intensity.

111

00:14:28.340 --> 00:14:43.409

NM: and children with Cp. At 4 and 5 like the Fourth of Fifth Level. And then i'm gonna ask you Why? All right. So the first question is, if you see how many days did your child exercise a place so hard that his or her body got tired, would you rate it 0 not appropriate at all.

112

00:14:43.680 --> 00:15:00.990

PT8: or 5 highly appropriate it could be anywhere along the next I I scan through, I think, for this survey. that it would really need to have a lot of education to the family before using it, because I could see a family getting really upset

113

00:15:01.080 --> 00:15:16.199

PT8: like Don't. You have an idea of what like child can or cannot do so either. I think the survey would need to be adapted, or there needs to be a lot of education, you know. so that

114

00:15:16.290 --> 00:15:21.669

PT8: you know, like that. No, and that the therapist would really need to know the child.

115

00:15:21.780 --> 00:15:27.460

PT8: because of this I was doing this as a in a bell on somebody. I

116

00:15:28.010 --> 00:15:31.820

PT8: I think this would not go better.

117

00:15:31.890 --> 00:15:34.600

PT8: there is another

118

00:15:34.830 --> 00:15:38.700

PT8: chart that that I another endurance

119

00:15:38.790 --> 00:15:44.099

PT8: Of that of that we have used in the past for children.

120

00:15:44.610 --> 00:15:47.180

NM: do you have the name of that to you.

121

00:15:47.380 --> 00:15:48.020

PT8: What

122

00:15:48.090 --> 00:15:55.340

PT8: you have the name of the tool you use before. Yeah it I would. I have to think of it offhand, it is

123

00:15:55.390 --> 00:16:02.709

PT8: from the child in place. Study up in that Mcmaster University. I had

124

00:16:02.890 --> 00:16:10.890

PT8: Some of our Pt. Students presented it, and we gave it to a couple of the kids. And here it was interesting because

125

00:16:11.540 --> 00:16:22.539

PT8: The family saw the child as active doing things, so then rated them actually a little bit higher, you know. Then we might separated them. So it was

126

00:16:22.950 --> 00:16:24.909

NM: interesting.

127

00:16:25.460 --> 00:16:30.039

NM: that's great. Okay, so but so this is why

128

00:16:30.190 --> 00:16:36.069

NM: i'm doing this study. Because, there are a few out there. And so, looking at

129

00:16:36.510 --> 00:16:50.490

NM: what we can maybe get from any of these questions and see what Pts feel like, maybe even relatable. because this is a national I mean, I think this though second the last, not the last question but the one before that.

130

00:16:50.520 --> 00:17:04.509

PT8: you know. If it with a definite definition of what is physically active, that was consistent. I think that would be something that could be worked. and I

131

00:17:06.069 --> 00:17:17.540

PT8: can I get a score for each question, though? Just to kind of, I would say for the question, I would say, that I like what you want. I don't think

132

00:17:17.550 --> 00:17:27.240

PT8: 0 is not applicable at all, and then 5 is highly appropriate. So somewhere, anywhere alone at scale 0. So I would say, 0, 0

133

00:17:29.220 --> 00:17:30.290

PT8: 0

134

00:17:31.100 --> 00:17:34.590

PT8: 3 0. I need that. Yeah 0.

135

00:17:35.080 --> 00:17:39.160

PT8: For the same reasons you stayed in the beginning.

136

00:17:39.270 --> 00:17:42.939

PT8: 0 for all of them. There's none better than you.

137

00:17:43.040 --> 00:17:50.770

PT8: I I think the only one that I would do is that one. How many days would your child physically active for 10 min or more?

138

00:17:51.880 --> 00:17:57.749

PT8: And defining what physically active would be in relationship to that child.

139

00:17:58.390 --> 00:18:04.460

NM: Okay, so that's number 7. What's what you get it? What good would you give it? A 5? I would give that a

140

00:18:04.630 --> 00:18:10.820

PT8: before like with with that with a good explanation. You know what they good. Okay.

141

00:18:12.130 --> 00:18:13.710

PT8: The others I don't think.

142

00:18:13.880 --> 00:18:29.299

PT8: because at all like no level like, you know, even though one about breathing hard Number 3 sweating because they might have. I'm. Because typically when i'm thinking about a lot of the children who you know, I don't know if

143

00:18:29.350 --> 00:18:30.800

PT8: if somebody would

144

00:18:31.120 --> 00:18:33.490

PT8: feel comfortable having a child

145

00:18:33.510 --> 00:18:43.969

PT8: at that level, breathing hard because a lot of the kids would also have respiratory compromise. So like finding that balance there. So I think i'd be nervous about that one.

146

00:18:45.180 --> 00:18:53.450

NM: And this is just to have a parent grade what they pay from the they're not going to put anything they're not gonna have I? I I think it would just be number 7 for me.

147

00:18:53.700 --> 00:18:55.110

NM: Got it. Okay.

148

00:18:58.770 --> 00:19:07.120

NM: And any any any rationale for the ones about muscle burning. Because I got some interesting answers about why that was the name of why that's inappropriate.

149

00:19:07.500 --> 00:19:12.320

PT8: I I because again, like, I think, how

150

00:19:12.750 --> 00:19:15.159

PT8: it's i'm thinking about

151

00:19:15.270 --> 00:19:20.570

PT8: this. We didn't really then say that it was a child that cognitively

152

00:19:20.670 --> 00:19:32.240

PT8: have an understanding of what it feels like to have their muscles burning, and so you'd have to make sure that it was somebody that was cognitively intact.

153

00:19:32.490 --> 00:19:51.430

PT8: It couldn't be somebody who just had the grading of activity for the level of gross motor function 4 or 5. But you'd also have to really read out the cognitive level, and I don't think I would want a parent to interpret that my child muscles were burning, you know.

154

00:19:51.440 --> 00:19:54.270

PT8: because then that makes it. Then you're getting into.

155

00:19:54.290 --> 00:19:56.079

PT8: or is the child in pain?

156

00:19:56.530 --> 00:19:57.810

NM: Hmm. Yep.

157

00:19:57.850 --> 00:19:59.439

NM: Okay, Very good.

158

00:19:59.510 --> 00:20:03.379

NM: Any comment about Number 6, Which is he? Or she felt tired.

159

00:20:03.490 --> 00:20:12.979

PT8: Do you think a parent will be able to assess that for the week. No, because I I I think that I don't think that it is

160

00:20:13.790 --> 00:20:17.979

PT8: yeah, no, I I i'd be I I'd be cautious about that.

161

00:20:18.370 --> 00:20:19.190

NM: Okay.

162

00:20:20.560 --> 00:20:28.989

PT8: I feel if I really want to get to physical activity. 7 is the only one you know like. Could I be then really saying, okay.

163

00:20:29.020 --> 00:20:33.480

PT8: the was your child active for more than 10 min throughout the whole day?

164

00:20:33.520 --> 00:20:39.289

PT8: and that would be a very minimal small amount of of movement, you know.

165

00:20:39.390 --> 00:20:40.140

NM: Yeah.

166

00:20:42.800 --> 00:20:48.319

NM: all right. Any last comments that you want to add about physical activity. This has been very helpful.

167

00:20:48.840 --> 00:20:50.760

NM: final.

168

00:20:51.270 --> 00:20:59.170

PT8: No, I mean, I think it's an interesting area to study, and I think it's like, I think, like looking at

169

00:20:59.690 --> 00:21:02.749

PT8: I think what's hard is that?

170

00:21:02.810 --> 00:21:08.479

PT8: that the focus a lot of times now is for children.

171

00:21:08.870 --> 00:21:10.979

PT8: that i'm finding

172

00:21:11.110 --> 00:21:21.470

PT8: that a lot of the programs focused like anything that is adaptive or after school. there is such a focus on children with autism.

173

00:21:21.750 --> 00:21:25.950

PT8: and I feel like that, or that

174

00:21:25.990 --> 00:21:42.030

PT8: this this, that children would serve a policy, get neglected in that sense, or also Seeing, then how to push into regular physical activities for children with cerebral quality, and how to adapt it.

175

00:21:45.690 --> 00:21:46.749

NM: That's great.

176

00:21:50.320 --> 00:21:56.379

NM: awesome, All right. I'm going to stop the recording. Thank you for your time. 1 s.
