# Supplementary material for: Validity of PROMIS® Pediatric Physical Activity Parent Proxy Short Form Scale as a Physical Activity Measure for Children with Cerebral Palsy Who Are Non-Ambulatory
Source: Behav Sci (Basel). 2025 Jul 31;15(8):1042. doi: 10.3390/bs15081042 (PMC12382615; doi:10.3390/bs15081042)
Supplement: Supplementary file 1 [file behavsci-15-01042-s001.zip › Transcripts copy/PT transcripts - deidentified/PT9.docx]

WEBVTT

1

00:00:01.680 --> 00:00:19.820

NM: Good afternoon. Thank you so much for meeting with me today to discuss physical activity and children with Cp. Who are not an ambulatory. So I do have a few questions that i'm going to ask you. And now forgive me because I had a little bit of a script, so there'll be some prompts for each question. So if I sound a little scripted that it is because I am

2

00:00:19.920 --> 00:00:23.769

NM: so. The first question I will ask you is.

3

00:00:24.000 --> 00:00:34.840

NM: How do you define physical activity for children with through quality who are not full time? Walker. Specifically, children within the GMFCS levels 4 and 5.

4

00:00:37.000 --> 00:00:41.930

PT9: I think you know Recently I've gone into the

5

00:00:42.000 --> 00:00:58.690

PT9: like. The efforts from Canchild in terms of, you know, promoting participation and using the ICF model. So I think you know anything that promotes participation, fun, family, and friends, even if it's not ambulation, would be a form of physical activity

6

00:01:00.430 --> 00:01:13.469

NM: great. So the department of health defines physical activity as any activity that encompasses energy expended, and activation of skeleton muscles. Does this definition change your mind about how you define physical activity?

7

00:01:14.340 --> 00:01:15.780

PT9: No, I don't think so.

8

00:01:17.800 --> 00:01:23.499

NM: And how do you think physical activity differs from other types of fitness activities

9

00:01:25.640 --> 00:01:30.570

PT9: when you say fitness activities? Do you mean like ambulation. Or do you mean what? What do you mean?

10

00:01:31.070 --> 00:01:42.800

NM: Well, when I think of the definition of fitness, I think more so like something more structured that has like a a goal that's related to some of the form of like, maybe structured exercise, or something like that.

11

00:01:42.950 --> 00:01:43.750

NM: But

12

00:01:44.380 --> 00:01:50.969

NM: you know i'm not saying it's different, different or not. I'm just saying in terms of how you think of physical activity. Does it differ

13

00:01:51.200 --> 00:01:55.189

from other types of fitness activities, and specifically in this population.

14

00:01:58.520 --> 00:02:07.710

PT9: I guess. Does it different? I don't know if it should differ. I mean, I think it's challenging to achieve fitness in this population because of the limitations in.

15

00:02:07.820 --> 00:02:10.190

PT9: you know, with lack of ambulation.

16

00:02:10.210 --> 00:02:14.009

PT9: So I think the goal should be to achieve a state of fitness. But

17

00:02:14.050 --> 00:02:15.399

PT9: it, I think it's harder.

18

00:02:20.600 --> 00:02:21.680

NM: Thank you

19

00:02:21.770 --> 00:02:38.429

NM: all right. And when do you witness your students or clients participate most in physical activity during the school day, or do you what what activities? Or if you don't witness it, do you know we'll have in terms of your students how they participate most in physical activity during school day

20

00:02:40.910 --> 00:02:53.040

PT9: during the school day. I'm not sure as being like on the outpatient side, I would say, probably in like their assisted stepping devices, or, you know, standing, you know, standing like in their equipment in different positioners.

21

00:02:53.090 --> 00:02:59.860

PT9: you know. Probably I would say the most. And then also, unfortunately, I think the most during therapy sessions also

22

00:03:00.670 --> 00:03:01.989

PT9: is what I would say

23

00:03:04.020 --> 00:03:14.789

NM: right And we'll go into more of the things you may do in therapy in a moment. Okay, that was the first question. Second question. Now, how do you measure physical activity, frequency.

24

00:03:14.950 --> 00:03:22.550

NM: intensity, time, and type? So that's the fitt principle, and children with Cp. Who are not full Time Walkers.

25

00:03:23.530 --> 00:03:31.000

NM: I'll say it again. How do you measure? Yeah, it's it's kind of tough. How do you measure physical activity, frequency.

26

00:03:31.040 --> 00:03:37.269

NM: intensity, time and type in children with Cp who are not full-time walkers.

27

00:03:38.740 --> 00:03:47.499

PT9: I think things a lot of that would be based on parent report if i'm looking at it that way. I think i'm looking at it in session. Then I'm looking at in session

28

00:03:48.820 --> 00:04:07.469

PT9: you know signs of fatigue whether that could be verbal, or you know other non-verbal signs of fatigue in terms of intensity. You know frequency in terms of how much i'm recommending from like a home exercise program, perspective, and then obviously choosing. You know the type of physical activity, and then

29

00:04:07.510 --> 00:04:11.430

PT9: I I think I hit all of your by answering that question

30

00:04:15.390 --> 00:04:16.190

NM: great.

31

00:04:16.459 --> 00:04:33.570

NM: How do you assist? How do they need assistance to complete these activities. So you mentioned just time and type. It would depend. So if you would select a type of physical activity right for this population? Do they need? Give me a type of activity, and then tell me what they need assistance to complete

32

00:04:33.650 --> 00:04:34.820

NM: the activity.

33

00:04:36.990 --> 00:04:42.389

PT9: So what i'm thinking, what comes to mind is like assisted stepping, so

34

00:04:42.650 --> 00:05:03.829

PT9: it depends on the patient. But some of them yes, and then some of them know, like some of them, can be in their gait training device, and you know, even though it's not necessarily like independent ambulation, they can get some sort of propulsion on their own, and then others do need that like assist for propulsion, even if it's i'm not talking like reciprocal gait, or anything like that. But even if it's just moving forward, for with some sort of

35

00:05:04.060 --> 00:05:06.490

PT9: like assistance from the therapist or family.

36

00:05:08.640 --> 00:05:20.440

NM: and kind of to what? To the stepping activity you gave a perfect example. Do they in in so? Yes, may they may be assistance for the stepping or not. But do they need assistance? For

37

00:05:20.680 --> 00:05:25.050

NM: to for the part of this, for the part of the task they need assistance in.

38

00:05:25.320 --> 00:05:31.300

NM: That would include, maybe prepare getting them in the device. Would that be fair? Okay, cause I can't. Okay.

39

00:05:31.380 --> 00:05:32.150

PT9: yeah.[they need assist with being put into the device]

40

00:05:34.910 --> 00:05:37.719

NM: And that's for most of this population. Would you say.

41

00:05:37.790 --> 00:05:39.609

PT9: Yeah, I would say so.

42

00:05:43.750 --> 00:05:54.720

NM: And do you think these kid all should participate in more or less of each of the activities you mentioned. What you mentioned stepping. If you have any others you would like to share, Would you? Do you think they should participate in more. And why.

43

00:05:55.740 --> 00:05:59.240

PT9: yeah, I definitely think there should be more. I mean, I think there's a lack of

44

00:05:59.390 --> 00:06:19.310

PT9: literature which is, I'm sure you're going in this route of You know, physical activity in this population because of the you know the movement difficulties. So I I that there has to be more, and it is challenging to figure out how to create more. I think, in a lot of circumstances. So that's just, you know 1 one example.

45

00:06:19.340 --> 00:06:22.710

PT9: you know. But yeah, I would love to include more

46

00:06:22.930 --> 00:06:24.360

PT9: somehow.

47

00:06:25.250 --> 00:06:35.229

NM: All right. So now let's talk a little bit about your Pt sessions. Do you address promoting physical activity during Pt. You kind of yes, answered that. And then how do you do that in your actual sessions?

48

00:06:36.540 --> 00:06:52.399

PT9: So I try to. I mean, I think I I can definitely do more of it. I try to incorporate as much like this assistant stepping. You know. If a family is not using a standing device like getting some sort of trial and then incorporating that into the home program. You know.

49

00:06:52.410 --> 00:07:04.749

PT9: I think something I need to focus more on is figure out more ways as opposed to just in sessions, or in a certain device of getting some sort of cardiovascular fitness. But I think that's the challenge.

50

00:07:05.340 --> 00:07:22.849

PT9: So I would say, those are. The main things is a lot of like equipment use. And then, or, if you know, if I can, depending on the size of the patient, obviously work on like a repetitive functional task that requires the patient to participate. Then i'm doing that too, so like sit to stand, or, you know, supreme, to sit transfers or things like that, if appropriate.

51

00:07:27.290 --> 00:07:42.869

NM: awesome. And so you gave gave some examples which are great. What components of physical activity do you address? So you You mentioned functional tasks like sit to stand so I can give you some examples. But what components of physical activity? What is it muscle activation.

52

00:07:42.880 --> 00:07:52.799

NM: Is it mobility like? What things do you feel like when you do the the the physical activity, work in your sessions? What are you targeting, and in terms of the components of physical activity.

53

00:07:53.500 --> 00:07:58.040

PT9: I think probably in general more so Mobility, I mean, yes.

54

00:07:58.310 --> 00:08:14.200

PT9: muscle activation. But you know again, depending on the age of the patient. If I know that we're going to have a tough time changing like selective motor control or certain activation patterns. And we're looking at that general mobility for participation as opposed to like specific muscle activation, even though

55

00:08:14.530 --> 00:08:16.930

PT9: you're gonna get that. Naturally.

56

00:08:17.360 --> 00:08:18.669

PT9: if that makes sense.

57

00:08:19.210 --> 00:08:25.789

NM: and there's so many others to those cardiovascular endurance, energy, expenditure. And are you targeting some of that, or

58

00:08:25.970 --> 00:08:43.249

NM: or more, mostly mobility and participation,

PT9: like I would say, mostly mobility and participation. I think it's hard within the time constraints of the clinic environment to truly achieve endurance, training and like energy, and you know, managing and improving, you know, like

59

00:08:43.570 --> 00:08:45.500

PT9: efficiency of energy expenditure.

60

00:08:51.570 --> 00:09:03.550

NM: And so I that was a perfect example, like if you're not able to work on any of those the time, the the time constraints can be a challenge. Yeah. And okay. So next and final question before we get to our survey.

61

00:09:03.580 --> 00:09:08.759

NM: do you address promoting physical activity that occurs now outside of your Pt session

62

00:09:10.670 --> 00:09:22.870

PT9: I try to. So in we try to encourage as much like adaptive extracurricular participation as possible, and as appropriate. But I find resources for that are low.

63

00:09:24.450 --> 00:09:32.099

PT9: you know. I think that would probably be the biggest way right now outside of Carry-over of what we do in sessions into the home environment or community environment.

64

00:09:35.040 --> 00:09:39.090

NM: Have you recommended any community programs specifically, or events

65

00:09:39.120 --> 00:09:41.450

NM: to your students to help increase physical activity?

66

00:09:42.330 --> 00:09:54.980

PT9: Yes, there's some, I mean honestly, a lot of times the families find them on their own, and then we talk about them. But there's some like adaptive like dance and sports programs that can be appropriate based on the child.

67

00:09:58.980 --> 00:10:06.870

NM: What type of equipment have you recommended to help improve home and or community engagement in physical activity outside of the clinic

68

00:10:08.200 --> 00:10:17.829

PT9: standers, powered mobility gait trainers. I typically use the pacer a lot just because that's what's available to me.

69

00:10:19.990 --> 00:10:26.420

PT9: You know, we've used at like adaptive adaptive strollers, depending on the age of the patient, you know, adaptive seating.

70

00:10:26.510 --> 00:10:42.480

PT9: There is, I will throughout there that we do at my clinic have a equipment specialist. So I am a little spoiled, and that I I have a general idea of what could be helpful. And then they refer to the equipment specialist who kind of helps me out from there. So I will say that

71

00:10:43.190 --> 00:10:44.339

NM: that's great.

72

00:10:44.490 --> 00:10:56.170

NM: Okay, so I have standers, payers, power, mobility. Yup. But I got a good amount of that's great, and all right. So now we're going to go into the survey. I'm going to share my screen. Give you a second to look at it and

73

00:10:58.240 --> 00:11:00.300

NM: wait a minute. I lost my

74

00:11:11.250 --> 00:11:12.770

NM: I'll show you

75

00:11:12.930 --> 00:11:31.939

NM: the 8 questions. The 8 questions survey. Okay. So take a moment to look at it. It's usually given to a parent or caregiver to determine a bit about their physical activity, intensity over the week, the past week. So it's a scale looking at no way that you know a parent can answer a child caregiver can answer about

76

00:11:31.960 --> 00:11:43.429

NM: how much their child has done. Let's say in the past 7 days. Okay. So now that you have getting a chance to look at it, what i'm going to ask you what i'm asking therapists and parents about this scale

77

00:11:43.500 --> 00:12:02.260

NM: is to see how valid is. Is it measuring physical activity in these kittles? Okay and specific visual activity intensity? So i'm going to ask you to write each question on how appropriate you feel. It is for this population, so 0 not appropriate all 5 highly appropriate. You think it's good to ask a parent these questions.

78

00:12:02.270 --> 00:12:06.879

NM: and then I'm going to ask you why, and i'm trying to get some. You know more

79

00:12:07.410 --> 00:12:25.980

NM: quantitative data. As I look at this at this scale. Okay. So so 5 5 is highly appropriate. No, ha. 5 is highly appropriate. 5 is highly appropriate. Okay, yeah. 0 and you can go as low as 0. Not at all, not at all appropriate. Okay. So the first question is, and these are children that are not inventory. Gms.

80

00:12:26.010 --> 00:12:34.969

NM: How many days did your child exercise a place so hard that his or her body got tired? How would you rate that 0? Not at all related 5 highly.

81

00:12:39.090 --> 00:12:44.319

PT9: I think that's highly appropriate, because, you know, play is defined in so many different ways. So

82

00:12:44.430 --> 00:12:48.540

PT9: yeah, I think that's appropriate, and you can tell when it When a child gets tired.

83

00:12:52.940 --> 00:12:54.320

NM: Great number 2.

84

00:12:54.490 --> 00:13:04.749

NM: How many days is your child exercise really hard for 10 min or more. How appropriate would you say this question is 0 not at all related 5 highly appropriate anywhere along the spectrum.

85

00:13:05.260 --> 00:13:07.229

PT9: Now, this would be given to parents right.

86

00:13:12.020 --> 00:13:21.719

PT9: I would maybe go with like a 3 on this one, because I feel like when you start using the word exercise, particularly if you're looking at a a patient that's not an ambulatory. Families may

87

00:13:22.310 --> 00:13:26.309

PT9: like, have a harder time figuring out what exercise means for their child.

88

00:13:30.530 --> 00:13:38.870

PT9: Right? What number did you give it again? And like a 3. Okay, neutral. Got it? So just the term exercise. Yeah, yeah.

89

00:13:44.120 --> 00:13:53.900

NM: hmm. Number 3. How many days is your child exercise so much that he or she breathed hard. How would you rate this question? 0 not related at all. 5 highly applied

90

00:13:54.280 --> 00:13:56.070

NM: somewhere along that spectrum.

91

00:13:56.840 --> 00:14:03.830

PT9: I would say the same as the other one, I mean, I think it's an appropriate question. But I just don't know if the term like exercises would throw them all

92

00:14:03.930 --> 00:14:05.770

PT9: like coming from a parent perspective.

93

00:14:05.810 --> 00:14:06.650

NM: Hmm.

94

00:14:08.660 --> 00:14:09.880

NM: My taking

95

00:14:14.390 --> 00:14:23.479

NM: now Number 4. How many days was your child so physically active that he or she sweated so a parent being able to answer that For how many days in the week

96

00:14:23.840 --> 00:14:29.070

NM: did they physically sweat? Is it highly related with the 5 or somewhere?

97

00:14:29.200 --> 00:14:39.600

PT9: Yeah, because I think it'd be easy for them to tell, like sweat or no sweat. And that's you know. Parents have that connection to physical activity, no matter what type of activity it is

98

00:14:49.670 --> 00:14:52.280

NM: okay, Number 5 right along.

99

00:14:52.320 --> 00:14:57.139

NM: How many days is your child exercise or play so hard that his or her muscles burned?

100

00:14:58.220 --> 00:15:04.809

NM: So how are 0 not related at all anywhere from a scale 0 to 5 5 being highly appropriate.

101

00:15:05.580 --> 00:15:11.610

PT9: I think that one's appropriate, too. I mean, I think, that one might be harder to answer for parents, but appropriate.

102

00:15:11.740 --> 00:15:14.260

PT9: So how would you rate it? So maybe like a 4.

103

00:15:14.360 --> 00:15:15.120

NM: Okay.

104

00:15:16.520 --> 00:15:20.700

NM: a little, you said, because it was a little, maybe a little difficult.

105

00:15:21.130 --> 00:15:23.539

PT9: I think. Just like muscles burned.

106

00:15:23.720 --> 00:15:32.449

PT9: and just thinking that might be hard for parents to know like it's not as visible right, you know, as sweating, or like visible signs of fatigue.

107

00:15:32.630 --> 00:15:40.839

PT9: but I think it's appropriate as a as like a measure of fatigue. I think it's appropriate. I think it's just harder to define, I guess, or or actually measure.

108

00:15:41.390 --> 00:15:42.210

NM: Okay.

109

00:15:44.920 --> 00:15:45.700

Got it.

110

00:15:46.810 --> 00:15:48.329

NM: And number 6.

111

00:15:49.190 --> 00:15:55.819

NM: How many days did your child exercise or play so play or play so hard that he or she felt tired.

112

00:15:56.020 --> 00:15:58.909

PT9: How is that? I give that a 5? Okay.

113

00:15:58.990 --> 00:16:00.040

NM: Yeah. And why?

114

00:16:00.860 --> 00:16:03.570

PT9: Because I think, like, I think that's

115

00:16:05.210 --> 00:16:10.070

PT9: almost similar to like the first question, you know.

116

00:16:10.780 --> 00:16:13.540

PT9: Yeah, honestly, yeah, they they seem similar to me.

117

00:16:14.060 --> 00:16:16.509

PT9: Okay. So for the same reasons. Yeah.

118

00:16:16.540 --> 00:16:17.160

okay.

119

00:16:21.370 --> 00:16:22.700

NM: Number 7.

120

00:16:23.490 --> 00:16:27.749

NM: How many days was your child physically active for 10 min or more?

121

00:16:28.250 --> 00:16:32.399

NM: How would you rate this 1? 0 not related at all? 5 highly appropriate.

122

00:16:32.810 --> 00:16:36.230

PT9: I would say, highly appropriate for this, too. Okay? And why?

123

00:16:36.750 --> 00:16:39.590

PT9: Because I think again, that's like an easy.

124

00:16:39.900 --> 00:16:42.670

PT9: easier, easy one for parents to

125

00:16:42.870 --> 00:16:45.290

PT9: measure like they can think about. Okay.

126

00:16:45.660 --> 00:16:52.430

PT9: you know, Did they do 10 min or more of some sort of activity? And I think that physically active can range and what that activity

127

00:16:52.780 --> 00:16:53.630

PT9: would be.

128

00:16:55.950 --> 00:16:58.190

NM: Okay and last one.

129

00:16:58.270 --> 00:17:03.640

NM: How many days is your child run for 10 min or more? 0 not related at all? Fine, highly appropriate.

130

00:17:04.740 --> 00:17:08.790

PT9: Well, for a non ambulatory kid, as I would say, 0

131

00:17:10.050 --> 00:17:11.020

NM: Gotcha.

132

00:17:11.690 --> 00:17:12.680

NM: Thank you

133

00:17:12.849 --> 00:17:20.180

NM: all right, and I've asked everyone to if they have any final comments about physical activity in this population. Anything that you wanna

134

00:17:20.339 --> 00:17:23.440

NM: you want to share just from your experience, or like a take home message?

135

00:17:25.520 --> 00:17:30.100

PT9: No, I mean really looking forward to seeing what you come up with. Because I think this is

136

00:17:30.810 --> 00:17:36.530

PT9: this we need. We need a so much more for our 4 and 5. So I think this is this is great.

137

00:17:39.700 --> 00:17:43.449

NM: Well, thank you. I'm going to stop the recording now.
