# Supplementary material for: Validity of PROMIS® Pediatric Physical Activity Parent Proxy Short Form Scale as a Physical Activity Measure for Children with Cerebral Palsy Who Are Non-Ambulatory
Source: Behav Sci (Basel). 2025 Jul 31;15(8):1042. doi: 10.3390/bs15081042 (PMC12382615; doi:10.3390/bs15081042)
Supplement: Supplementary file 1 [file behavsci-15-01042-s001.zip › Transcripts copy/PT transcripts - deidentified/PT15.docx]

WEBVTT

1

00:00:01.350 --> 00:00:16.489

NM: All right. Good morning. Thank you so much for joining me today as we discussed physical activity for children with Cp. Who are not full time. Walkers, Gmfc. Is levels 4 and 5. I'm. Going to ask you a few questions about your clinical expertise and how you view physical activity in this population.

2

00:00:16.500 --> 00:00:32.050

NM: and then the second half of this interview. I'm going to share a survey where i'll have to look at it, and we'll ask you some questions about how related or validated the scale may be for children in this population. If I sound scripted, it's because I am so. I have a little bit of a a

3

00:00:32.060 --> 00:00:40.109

NM: a script to follow. Just so i'm as consistent as possible. All right, so, and I have prompts for each question as well. All right. So the first question

4

00:00:40.140 --> 00:00:46.279

NM: is, how do you define physical activity for children with Cp. Who are not full time Walkers.

5

00:00:47.290 --> 00:00:59.549

PT15: Oh, that's a great question. Their physical activity is what they're able to do to the maximal capacity that they have, that their body allows them to move.

6

00:00:59.560 --> 00:01:14.870

PT15: and also it includes what the parents are doing, or what the therapist is instructing the parents to increase that activity because their physical activity tends to be less than your typical child.

7

00:01:16.900 --> 00:01:29.679

NM: Great. Thank you. The Department of Health defines physical activity as any activity that encompasses energy expended and activation of skeletal muscle. Does this definition change your mind about how you define physical activity?

8

00:01:30.460 --> 00:01:31.380

PT15: No.

9

00:01:32.290 --> 00:01:35.789

NM: And how do you think physical activity differs

10

00:01:36.270 --> 00:01:39.590

NM: from other types of fitness activities?

11

00:01:40.270 --> 00:01:56.550

PT15: So when I think of fitness, I think of the repetitive motions and activities, using the full range that your body should be able to do versus what the children that I see.

12

00:01:57.130 --> 00:02:05.849

PT15: You know their repetitive repetitive motions are very limited. They don't have a wide repertoire of movement.

13

00:02:10.020 --> 00:02:16.010

NM: and when do you witness your students participate most in physical activity during the day or in the school day.

14

00:02:17.270 --> 00:02:29.470

PT15: when they are included, when they actually have an aid helping them or their parents are helping them, or the therapist comes in and doing some treatment

15

00:02:33.920 --> 00:02:35.130

NM: great. Next question.

16

00:02:35.620 --> 00:02:46.250

NM: How do you measure physical activity, frequency, intensity, time and type, and children with Cp. Who are not full time work walkers. So here i'm quoting in a fitt principle.

17

00:02:46.450 --> 00:02:53.149

NM: So again, that's frequency, intensity, time and type for children with Cp. And levels 4 and 5.

18

00:02:54.440 --> 00:03:06.319

PT15: So the question was, how do I set it?

NM: How do you measure it?

PT15: Oh, how do I measure it so? I mean you get through your examination process and just looking at

19

00:03:06.700 --> 00:03:07.950

PT15: to mobility.

20

00:03:08.140 --> 00:03:26.869

PT15: How often are they moving? You do an interview process with the parents and asking, okay, so what does a typical day look like for that child, also asking the teachers what does it? You know? What are they doing during class time? What do you expect that child to be doing, and if they have that

21

00:03:26.880 --> 00:03:30.989

PT15: that aid or not to help.

22

00:03:31.440 --> 00:03:43.720

PT15: and then, as far as like the outcome issues, it'd be, you know, things like the GMFM. And just seeing whether at depending on the age of that child also.

23

00:03:44.100 --> 00:03:50.329

PT15: like I said just in the general of what are they exhibiting during that time that I see them.

24

00:03:50.360 --> 00:03:54.899

PT15: How active are they? And then just kind of do a analysis that way?

25

00:03:56.840 --> 00:03:57.900

NM: Thank you.

26

00:03:58.020 --> 00:04:01.299

NM: Do they need assistance to complete these activities.

27

00:04:01.420 --> 00:04:08.030

NM: and during which activities would they be assistance? And is it for just part of the task or the entire task?

28

00:04:10.180 --> 00:04:19.710

PT15: Now, that's a big question, right? It depends on the level of the child how much spasticity is is.

29

00:04:19.970 --> 00:04:39.289

PT15: You know they're exhibiting during some of those activities. So a lot of a lot of the times it has to do with. Okay, Can we use a type of equipment to help support the rest of the body? So maybe it free’s up the arms so they could do some some activation on switches, or you know

30

00:04:40.590 --> 00:04:58.319

PT15: well I have one client who the parents still would like for them to keep walking, but now he's like 13-14, and so we have to use a mobility device with the realization that he's not propelling in a typical way that you would expect.

31

00:04:58.330 --> 00:05:13.160

PT15: and that he actually needs a lot more assist. So it's a lot more on the caregiver on the therapist, on on the aid to actually get them on that type of equipment. So try to be realistic about okay.

32

00:05:13.330 --> 00:05:17.710

PT15: How are they functioning at this point? How can we get them to function

33

00:05:17.750 --> 00:05:27.019

PT15: appropriately and not like you said expend so much energy, and then their fatigue for the rest of the day. So it's kind of like that balancing act

34

00:05:27.550 --> 00:05:30.800

PT15: that we had we have to do. I don't know if that answered it. But

35

00:05:30.960 --> 00:05:45.109

NM: no, I do. I was curious about some other like. Can you give me some another examples of activities where they may need assistance, and how how much assistance they may need like During the during the task. We can give you an example of.

36

00:05:45.200 --> 00:06:00.280

PT15: So another one was where we were in the classroom, and all the children Small child. I think he was in kindergarten, or it could be in first grade where they were still doing circle time. And this child

37

00:06:00.430 --> 00:06:03.060

PT15: cannot sit on their own.

38

00:06:03.280 --> 00:06:05.180

or if they did

39

00:06:05.310 --> 00:06:13.669

PT15: it. So it's another. So it's 2 different kids that I I have pictures of right in my head or the other child that could props it, but it was so

40

00:06:14.200 --> 00:06:17.780

PT15: like rounded in the back, and he has to hyper extend the head

41

00:06:17.800 --> 00:06:23.549

PT15: again. Energy expenditure. So just being realistic about that. And how much is this? So really it's. It's

42

00:06:23.570 --> 00:06:31.479

PT15: using equipment to the best of the ability to help support, and still not spend so much energy

43

00:06:31.490 --> 00:06:47.429

PT15: during just even circle time on the floor. Is it realistic to get that child on the floor with the rest of the classmates, you know. So again balancing act between the 2? Do we keep them in the equipment, do we, or do we get some form of equipment that

44

00:06:47.440 --> 00:06:56.219

PT15: you know I have to fight for with the school district to try and get so they can be low to the ground. That balancing act.

45

00:06:56.760 --> 00:07:06.499

NM: So it sounds like from what you share to that they may need assistance for most of the task, if not all for this population. I'm just. I don't want to put words in the out, but from the examples you've given.

46

00:07:06.590 --> 00:07:13.509

PT15: Yeah, all read at this at this point. From what I see, they all need equipment

47

00:07:18.040 --> 00:07:23.469

NM: great. And do you think they should participate in more or less of these activities. And why

48

00:07:24.410 --> 00:07:34.970

PT15: definitely, definitely more activities Because it's that participation we're we're looking at that ICF model, right? So we really want them to engage in

49

00:07:34.980 --> 00:07:46.050

PT15: not be segregated out from the peers and have the peers say, oh, okay, you know the part of the class. It's a typical

50

00:07:46.100 --> 00:07:57.119

PT15: engagement. It's not going to be that separation. Oh, my gosh, okay. They have a disability. They're over there. They're out in the corner. So really it's it's it's all about that engagement

51

00:07:57.190 --> 00:08:01.969

PT15: in in participation. Get belonging into that classroom.

52

00:08:03.280 --> 00:08:10.960

NM: That's great. Thank you. All right. Next question. Do you address promoting physical activity during your your physical therapy session

53

00:08:11.890 --> 00:08:41.800

PT15: all the time, all the time as much physical activity and the way it's. So when we talk when you talk about the FITT, because I put that in my evaluations right? I always have FITT. My evaluations always has a fitt, you know. I'm. I'm always discussing. Hey, what does that look like? We may say 3 times a day, but when I break it down to the families or break it down to the instructions, it's as much as possible, as much engagement and participation as I can

54

00:08:41.809 --> 00:08:51.540

PT15: throughout all day, so that way. But to make it easier if if it's easy for you to remember to these activities. Do it at breakfast. Do it at lunch, do it at dinner.

55

00:08:52.930 --> 00:08:55.110

PT15: That's how I kind of break it down for them.

56

00:08:55.640 --> 00:09:00.249

NM: And how do you? How do you address this in your physical therapy session specifically?

57

00:09:00.580 --> 00:09:02.449

NM: How do you use the activity

58

00:09:02.630 --> 00:09:07.320

PT15: so based on the activities that we've worked on.

59

00:09:07.550 --> 00:09:28.220

PT15: I usually so I discuss it with the child, regardless of the child, is cognitive enough to understand, but I make it where it's understandable, for also the parents when they're listening, because they're so in engaged, or you know, usually the teachers pretty busy. So with teachers. I have to kind of separate myself out and and

60

00:09:28.230 --> 00:09:43.529

PT15: discuss with them with the Aids- They're usually right there with you, too, unless they have, like 5 other kids with them. So it's the same. It just depends on how engaged that person is with me. And then that's that's how I so

61

00:09:43.550 --> 00:10:02.419

PT15: if they're not as engaged because they have other children that they have to take care of. Then I will set aside, you know. Oh, can we talk for at least 5 min and talk about this, but if they're right there with me, they they're engaging. They're listening, and I talk with the child while the parents are listening at the same time. So they hear the same information.

62

00:10:04.270 --> 00:10:23.090

NM: Okay, that's great. Now, what components of physical activity are you addressing during a physical therapy session, for example, Are you addressing cardiovascular endurance, muscle, activation, energy, expansion, mobility there so many things you could work on. But what are the components of physical activity? Do you address in a Pt. Session.

63

00:10:23.550 --> 00:10:28.990

PT15: So it's it's like you said there's so many components to it. And so

64

00:10:29.880 --> 00:10:42.600

PT15: you know I always let my parents know my job isn't to really teach those skills. It's more of let's introduce these skills. So maybe they start to take over some of these skills.

65

00:10:42.610 --> 00:11:01.079

PT15: Teach them that there's different ways to move, and so I talk about different speeds of movement, you know, rolling. It could be slow. But then, you know, you try to get the muscle activation, and you know you're trying to get that dissociation between the limbs. But then you can also speed it up, and sometimes

66

00:11:01.140 --> 00:11:12.979

PT15: they like that speed. They like the momentum, and then they kick in all their spasticity, and for them that might be functional. And so it might be a repetitive. That's all the way that they know how to do it.

67

00:11:12.990 --> 00:11:24.609

PT15: and that's a different speed. But you're also going to help them realize that. Oh, I can even do it slow, and when I do it, though it may look a little different than if I sped it up so

68

00:11:24.960 --> 00:11:28.429

PT15: really it's just teaching, and there's different ways to move.

69

00:11:28.440 --> 00:11:47.150

PT15: and that encompasses everything that you just mentioned. Mobility, range dissociation, strengthening endurance. So then, we also talk about, like, you know, repetition. If it's throughout the day, you know minimum, if we could do it 3 times a day. Fantastic. But imagine, if you just do it all the time.

70

00:11:47.590 --> 00:11:51.220

PT15: then we're we're increasing that that endurance. And yeah

71

00:11:53.290 --> 00:11:59.999

NM: great. So in your actual session, when your hands, or with with your child.

72

00:12:00.360 --> 00:12:04.800

NM: what do you feel like you most target like in a session, whether the parents, they or not.

73

00:12:05.010 --> 00:12:10.239

NM: What do you really in terms of like your go to when you're actually with the child?

74

00:12:10.580 --> 00:12:14.290

NM: What do you feel like you're targeting the most as it relates to physical activity?

75

00:12:14.740 --> 00:12:15.950

PT15: Oh, gosh.

76

00:12:16.140 --> 00:12:27.970

PT15: definitely, the dissociation. Yeah. Dissociation of the body to be able to actually do that type of mobility and the repetition.

77

00:12:28.700 --> 00:12:38.369

NM: Okay, Is That kind of… is that tied to like muscle activation. Is that muscle activation

PT15: muscle activiation, strengthening

78

00:12:38.450 --> 00:12:57.009

PT15: I try to get them moving at different speeds because I try and get vestibular because they know, and so trying to get them different vestibular. Input, Get those canals moving the the fluids in the ear, moving, you know. I’m there for an hour…But again, when i'm not there, i'm i'm don’t know

79

00:12:57.020 --> 00:13:02.649

PT15: when i'm not there. What what difference did I make. I don't know

80

00:13:02.850 --> 00:13:12.439

NM: you're leading me right into my next question, PT15. So that's coming right. Now you're you're so good before I get there, though, when when you don't want to work on, let's say

81

00:13:12.660 --> 00:13:18.049

NM: muscle activation in your energy expenditure. Would there be reasons why you wouldn't focus on this? And why

82

00:13:18.470 --> 00:13:29.590

PT15: you know, it just depends on the day and the presentation the mood. There's so many other yeah, external factors that kind of kick in. And sometimes, you know

83

00:13:31.860 --> 00:13:39.400

PT15: I mean, okay. So I had one that was very highly cognitive with me there, so they'll let me know.

84

00:13:39.500 --> 00:13:58.970

PT15: You know. I give them choices, and sometimes they'll let me know. Look, i'm so tired! It was such a long day. Can we do something different? Whereas my non cognitive - They may not have as many choices, and I just go through it, you know with what my plan was to begin with. But then I listen to their body.

85

00:13:58.980 --> 00:14:10.899

PT15: and I listen to them and their responses. So, even though they may not be able to verbally let me know, they definitely will tell me otherwise.

86

00:14:11.810 --> 00:14:12.710

NM: Great.

87

00:14:12.730 --> 00:14:22.990

NM: All right. Next question do you address promoting physical activity that occurs outside of your Pt. Session? And you already answered that pretty good. So how do you do that?

88

00:14:23.140 --> 00:14:36.779

PT15: So it's. It's definitely a discussion of hey, what does you know, You want your life not to revolve around therapy. Life should not be about physical therapy, and so you know

89

00:14:36.800 --> 00:14:39.060

PT15: me, seeing you guys forever.

90

00:14:39.260 --> 00:14:42.409

PT15: We need to start including

91

00:14:42.440 --> 00:14:48.409

PT15: activities that you guys all enjoy. So one of the the kiddos that I had they love swimming

92

00:14:48.520 --> 00:14:54.489

PT15: They love it. The family loves swimming their older child, the twin, I should say

93

00:14:54.830 --> 00:15:00.490

PT15: they're a competitive swimmer, you know. So how can we include that child

94

00:15:00.530 --> 00:15:01.490

PT15: with

95

00:15:01.540 --> 00:15:11.599

PT15: the one that i'm seeing. So then we do outside of stuff. Hey, You're in the pool that's fantastic. While you're in the pool. You can kind of do some some of these things, but otherwise

96

00:15:11.640 --> 00:15:22.120

PT15: move around. Go. Go, do those things. How else can we include them? You know you love going to the park? All right. It's not. You know it's not.

97

00:15:22.350 --> 00:15:41.989

PT15: It's not feasible for them to actually walk and use that gait trainer, especially with the, you know, when they have Timber on the floor and stuff. And so what equipment can we use instead, You know these, the some of these children get bigger and older, and it's not as realistic, but they still enjoy going. So how

98

00:15:42.000 --> 00:15:54.589

PT15: so It's just kind of coming up with different ways, but allowing the parents to know that, hey, you have a life, you have a family life, and it's beyond therapy, and therapy should not be about you know, their life.

99

00:15:54.690 --> 00:15:58.110

PT15: but it seems like it to a lot of them. But it shouldn't be that way.

100

00:16:01.700 --> 00:16:04.319

NM: That's good, thank you.

101

00:16:04.440 --> 00:16:11.119

NM: What have you recommended? Any community programs or specific events to your students self increase physical activity.

102

00:16:11.670 --> 00:16:17.150

PT15: Yeah. So the special Olympics for sure

103

00:16:17.330 --> 00:16:23.270

PT15: I know in the school district here. Usually they have that one day where they do the

104

00:16:23.370 --> 00:16:34.599

PT15: similar similar things. And so the therapist comes in, and they, you know they help the child, and then they go through the whole obstacle course, and they make it a day out of that.

105

00:16:35.400 --> 00:16:38.130

PT15: as oh, so I have

106

00:16:38.380 --> 00:16:55.770

PT15: said, you know, even just going to like a ballet studio, you know, and just having them watch. But, hey, instead of just watching, why, aren't they in the middle of it, and just kind of allowing the other dances. And you know you you know the parent participate.

107

00:16:55.780 --> 00:16:59.639

PT15: But that's kind of outside the the box for a lot of them.

108

00:16:59.790 --> 00:17:00.950

NM: Yeah.

109

00:17:01.270 --> 00:17:19.710

PT15: because they, the parents definitely see the difference between their child and the group. And so you know, and it's time constraints, too. It's. It's trying to be creative, but yet realistic about some of these things when when recommending outside groups. If I find

110

00:17:19.720 --> 00:17:35.210

PT15: through, you know, because I had a child with down syndrome and Cp. And autism. So when I was doing like, you know, those groups with different activities, I share it straight away. Hey, this is, you know this is what's going on.

111

00:17:36.380 --> 00:17:37.959

PT15: and then I go. Thanks, PT15.

112

00:17:38.370 --> 00:17:41.579

PT15: I don't follow up whether they attend or not.

113

00:17:41.740 --> 00:17:45.370

PT15: providing them that information with them.

114

00:17:47.440 --> 00:17:48.500

Lastly.

115

00:17:48.740 --> 00:17:55.769

NM: what type of equipment. Have you recommended to improve home and or community engagement of physical activity outside of the clinic?

116

00:17:56.640 --> 00:17:58.660

so

117

00:17:58.940 --> 00:18:10.579

PT15: so definitely There's both the wheelchair depending on the level, you know, cognitive level, the level of the child mobility. We've adjusted power wheel chairs.

118

00:18:10.590 --> 00:18:20.089

PT15: The it might be like a I don't want to say stroller base wheelchair, but you know a wheel chair that The caregiver or the aid can manually push.

119

00:18:21.270 --> 00:18:29.850

PT15: We've done those that still want to do the gait trainers depending on the age 2 we'll do gait trainers.

120

00:18:30.180 --> 00:18:31.290

PT15: Oh, gosh!

121

00:18:32.730 --> 00:18:42.189

PT15: Oh, I mean we've even done, you know, standers with wheels, and just having them, like you know, be in the stander and and push them around.

122

00:18:42.320 --> 00:18:43.210

PT15: So yeah.

123

00:18:45.960 --> 00:18:51.020

NM: all right, we're at the second half where i'll share the screen so you can look at this promise scale.

124

00:18:51.380 --> 00:18:58.049

NM: And so this promise scale again was developed by the National Institute of Health. There are 8 questions.

125

00:18:58.080 --> 00:19:06.850

NM: and this is given to a parent. A parent will answer these questions related to what they believe their child's physical activity. Intensity was the week prior to answering.

126

00:19:07.030 --> 00:19:16.120

NM: and so they would say, You know in the past 7 days have they done this and that? And what I'm going to ask you as we go through this is how you would rage, question

127

00:19:16.200 --> 00:19:24.669

NM: how you know how valid it would be in this population, and i'll, I'll ask each question, and i'll ask you why you answered it the way you did it.

128

00:19:24.730 --> 00:19:29.639

NM: and so let's go ahead and get right into it. So for the first question

129

00:19:30.050 --> 00:19:31.620

NM: as we read through it.

130

00:19:31.950 --> 00:19:45.229

NM: i'm gonna ask you to how to answer. How appropriate the question is addressing physical activity, intensity, and children at Gmf. Is levels 4 and 5, and out of the options I'm. Going to ask you to rate it on it scale from 0,

131

00:19:45.300 --> 00:19:51.179

NM: not related at all up to 5. It's highly appropriate. And then why? Okay?

132

00:19:51.200 --> 00:19:54.020

NM: So again, these questions questions will be asked to a parent.

133

00:19:54.500 --> 00:20:03.099

NM: How many days did your child exercise or play so hard that his or her body got tired? How would you rate that question? And why 0 not related at all

134

00:20:03.170 --> 00:20:05.970

NM: up to 5, which is highly appropriate?

135

00:20:06.530 --> 00:20:11.920

PT15: I think. 5 cause that's a that's a great question to us

136

00:20:12.430 --> 00:20:20.450

PT15: because it gives them it gives that parent. Okay, what do you mean by exercise at play? And then they qualified it that you know

137

00:20:20.510 --> 00:20:26.710

PT15: that the child's body got so tired from it. So that that's actually a good question to ask.

138

00:20:26.920 --> 00:20:27.550

NM: Okay.

139

00:20:27.710 --> 00:20:28.620

NM: thank you.

140

00:20:28.680 --> 00:20:29.710

NM: Number 2.

141

00:20:30.140 --> 00:20:34.290

NM: How many days did your child exercise really hard for 10 min or more

142

00:20:34.740 --> 00:20:41.230

NM: like that 0 not related at all for children that are not inventory up to 5 highly appropriate.

143

00:20:41.470 --> 00:20:42.310

NM: Why.

144

00:20:46.070 --> 00:20:47.880

PT15: I would say.

145

00:20:48.460 --> 00:20:50.929

PT15: definitely appropriate.

146

00:20:53.510 --> 00:21:02.969

PT15: I think the hip hop might be the 10 min like not like they really. And and how do you qualify? How do you qualify that really had

147

00:21:03.730 --> 00:21:10.599

PT15: 10 min, some more when they don't really have it, you know. I would say 4 on that one.

148

00:21:10.860 --> 00:21:11.710

NM: Okay.

149

00:21:13.230 --> 00:21:16.019

PT15: But it's still a great question. Now you know

150

00:21:16.200 --> 00:21:34.680

PT15: why. Why is this still a great question? Because it's still saying, hey, do have you really thought about it? Have you really exercise, because a lot of these kids like, I said, if it's me once a week, you know the parents are they really doing 10 min, or they so busy in their life

151

00:21:34.730 --> 00:21:41.209

PT15: that it might give him pause to wait and say, Whoa, did I? Did they really exercise?

152

00:21:41.490 --> 00:21:42.630

PT15: So

153

00:21:43.070 --> 00:21:43.930

PT15: yeah.

154

00:21:44.500 --> 00:21:46.249

NM: okay, Number 3,

155

00:21:46.400 --> 00:21:50.850

NM: how many days is your child exercise so much that he or she breathe hard?

156

00:21:51.130 --> 00:21:57.119

NM: That's 0, not related at all. 5 highly appropriate, or somewhere between? How would you rate that question?

157

00:21:57.480 --> 00:21:59.480

PT15: I would say

158

00:21:59.680 --> 00:22:00.860

PT15: a 3.

159

00:22:01.220 --> 00:22:05.879

PT15: Okay, because breathing hard.

160

00:22:05.910 --> 00:22:06.960

PT15: you know.

161

00:22:07.310 --> 00:22:11.279

PT15: they may not necessarily be a tune to that breath.

162

00:22:11.690 --> 00:22:22.750

PT15: It might be more of the the body getting tired right, or they got so tired they slumped, but they may not be necessarily focus on that breathing

163

00:22:22.970 --> 00:22:24.580

so much.

164

00:22:29.080 --> 00:22:30.940

PT15: Now you're making me think

165

00:22:31.240 --> 00:22:35.139

PT15: when I when I do the exercises.

166

00:22:36.490 --> 00:22:41.010

PT15: my focus may not really be on the breath. More of the responses

167

00:22:41.500 --> 00:22:46.850

PT15: to. You know the fatigue of the muscle, because we're separating

168

00:22:46.960 --> 00:22:51.470

PT15: body parts out and concentrating, and it may not necessarily be

169

00:22:52.480 --> 00:22:55.160

PT15: the whole body for some of those kids

170

00:22:58.030 --> 00:22:58.800

alright.

171

00:22:58.990 --> 00:23:00.770

NM: all right. Number 4.

172

00:23:01.830 --> 00:23:10.579

NM: How many days was your child so physically active that he or she sweated, how would you rate this question? 0 not related at all up to a 5 highly appropriate.

173

00:23:10.610 --> 00:23:11.640

NM: And why?

174

00:23:12.900 --> 00:23:28.610

PT15: So? I would say 3 as well, same as the last question, because some sometimes some of these kids they might have sweat issues. Some may not, but a lot. So that that's kind of hard to gauge.

175

00:23:30.040 --> 00:23:32.269

PT15: I think the parents will have a had time

176

00:23:32.850 --> 00:23:34.130

PT15: seeing this one.

177

00:23:34.650 --> 00:23:35.510

Yeah.

178

00:23:36.230 --> 00:23:37.170

okay.

179

00:23:39.670 --> 00:23:40.939

NM: Number 5.

180

00:23:41.070 --> 00:23:44.979

NM: How many days did your child exercise up to so hard that

181

00:23:45.580 --> 00:23:49.620

NM: his or her muscles 9 0 not related at all 5

182

00:23:50.460 --> 00:23:52.730

NM: highly appropriate. How would you write this question?

183

00:23:53.320 --> 00:23:57.780

PT15: A 3 as well? Because at least that child is cognitive.

184

00:23:59.440 --> 00:24:02.620

PT15: and you explain what muscle burning means?

185

00:24:03.130 --> 00:24:09.220

PT15: I don't think parents understand that. I I mean that that's the hard one for the parents to understand.

186

00:24:13.120 --> 00:24:14.839

NM: All right, Number 6.

187

00:24:15.460 --> 00:24:24.049

NM: How many days did your child exercise a place so hard that he or she felt tired. 0 not related at all up to a 5 highly appropriate. And why

188

00:24:24.420 --> 00:24:27.940

PT15: 5, cause they'll understand that that question.

189

00:24:28.230 --> 00:24:31.429

PT15: because they'll see a change in the

190

00:24:31.670 --> 00:24:35.339

PT15: sleep, or you know.

191

00:24:35.410 --> 00:24:42.729

PT15: if there's every stimulus not stimulated enough that They' they they'll see the tired. They'll see that description attired.

192

00:24:43.950 --> 00:24:44.830

NM: Gotcha

193

00:24:45.120 --> 00:24:46.360

NM: Number 7.

194

00:24:46.430 --> 00:24:50.600

NM: How many days was your child physically active for 10 min or more?

195

00:24:50.840 --> 00:24:52.290

0?

196

00:24:52.340 --> 00:24:57.360

NM: Not an applicable at all. Number up to 5 highly appropriate. How would you rate that question? And why?

197

00:24:57.510 --> 00:24:58.840

For

198

00:24:59.040 --> 00:25:00.820

PT15: okay

199

00:25:01.030 --> 00:25:04.979

PT15: again, physically active for 10 min or more?

200

00:25:05.670 --> 00:25:10.139

PT15: Is it independent? Is it with this.

201

00:25:11.860 --> 00:25:19.299

PT15: And what did they mean by physically active? Maybe they qualified some of those activities or what they're looking for?

202

00:25:26.070 --> 00:25:26.830

Hmm.

203

00:25:29.310 --> 00:25:31.190

NM: All right. Number 8 last one.

204

00:25:31.260 --> 00:25:37.509

NM: How many days did your child run for 10 min for more? How appropriate would you you rate that 1 0? Not at all.

205

00:25:37.610 --> 00:25:41.370

NM: 5 highly appropriate, or somewhere between, and why

206

00:25:43.970 --> 00:25:47.359

PT15: we saw my face right.

207

00:25:47.450 --> 00:25:50.939

PT15: But kids that are 4 or 5. Are they running?

208

00:25:54.130 --> 00:25:58.640

PT15: They're not running. I i'm trying to think of which kids I have that run

209

00:26:02.500 --> 00:26:04.090

PT15: again. It's so much.

210

00:26:04.590 --> 00:26:06.980

PT15: And no, I don't think it's appropriate.

211

00:26:07.160 --> 00:26:08.609

NM: Okay, give me a number

212

00:26:10.200 --> 00:26:24.819

PT15: 0. I gotta think, like you know. What do they qualify by running me going in there and repetitively doing this? And i'm i'm getting exhausted. But it's so passive for the child, you know.

213

00:26:25.660 --> 00:26:28.330

NM: All right. Okay. So that was

214

00:26:28.650 --> 00:26:33.890

NM: the last question. And what I do before we close is, I ask the the therapist to

215

00:26:34.260 --> 00:26:37.470

NM: share any final thoughts that they want to share about

216

00:26:37.490 --> 00:26:44.000

NM: how what they believe about physical activity in this population. He's kind of closing statements we like to say

217

00:26:44.430 --> 00:26:45.529

NM: as we.

218

00:26:45.750 --> 00:26:57.699

PT15: you know I I I think it's really important to qualify what that physical activity means especially, and and how it works for that for the population.

219

00:26:57.760 --> 00:27:13.500

PT15: because right now a lot of them are getting that physical activity that we would like to see right that more intensive. But how feasible is it and realistic and cost-wise for families to be able to do that.

220

00:27:13.540 --> 00:27:14.509

PT15: You know it.

221

00:27:15.620 --> 00:27:17.179

PT15: It's it's like

222

00:27:18.500 --> 00:27:20.620

PT15: what happens after

223

00:27:21.350 --> 00:27:32.519

PT15: you know after doing intensive what happens after that, you know they get the home program from those intensive. But are they really doing it at home to continue there?

224

00:27:32.560 --> 00:27:36.410

PT15: You'll see improvements. But is that child

225

00:27:36.430 --> 00:27:51.920

PT15: really able to do that physical activity to the point of fatigue and working out. And you know it's still a child that they're going to do what the body needs to also do. So we may be pushing for all these things.

226

00:27:52.240 --> 00:27:57.159

but it sometimes we just have to also step back, and really really look at the whole picture

227

00:27:57.350 --> 00:27:58.140

PT15: right?

228

00:27:59.380 --> 00:28:00.059

PT15: And it

229

00:28:00.480 --> 00:28:04.070

PT15: it should be on a case by case basis, as far as like

230

00:28:05.620 --> 00:28:07.229

PT15: the expectations

231

00:28:07.270 --> 00:28:16.460

PT15: and change when it needs to and be dynamic in that sense versus hey? You really got to do this, you know, 3 times a day.

232

00:28:16.970 --> 00:28:18.560

PT15: 5 days a week.

233

00:28:18.690 --> 00:28:32.570

PT15: which would be ideal. But but realistically, can they really do this? So I think the more active that child can get, the more engaged. Yeah, the more they interact with with the rest participation.

234

00:28:32.740 --> 00:28:33.860

NM: Hmm.

235

00:28:36.640 --> 00:28:37.970

PT15: Hopefully, it makes sense.

236

00:28:38.360 --> 00:28:44.340

NM: It did. It did. Okay, Thank you so much. Hold on, let me stop the recording. We are included.

237

00:28:44.480 --> 00:28:45.619

NM: All right to you.
