# Supplementary material for: Validity of PROMIS® Pediatric Physical Activity Parent Proxy Short Form Scale as a Physical Activity Measure for Children with Cerebral Palsy Who Are Non-Ambulatory
Source: Behav Sci (Basel). 2025 Jul 31;15(8):1042. doi: 10.3390/bs15081042 (PMC12382615; doi:10.3390/bs15081042)
Supplement: Supplementary file 1 [file behavsci-15-01042-s001.zip › Transcripts copy/PT transcripts - deidentified/PT5.docx]

WEBVTT

1

00:00:01.460 --> 00:00:16.870

NM: All right. Good afternoon, PT5 Thank you for joining us, Dr.PT5 and today we're going to talk a little bit about physical activity, and I really appreciate your time. So I have a few questions and have some follow ups, and then we'll look at a specific tool. So the first question is.

2

00:00:17.000 --> 00:00:23.030

NM: How do you define physical activity for children with Cp. Who are not full time Walkers.

3

00:00:23.860 --> 00:00:28.710

PT5: I define physical activity. for

4

00:00:28.880 --> 00:00:37.510

PT5: children who are not ambulatory as just really anything that gets their heart rate up anything that is activating their muscles. And

5

00:00:38.120 --> 00:00:47.409

PT5: really, anyway, because if they're not walking, they, You know they're not going to have that ability, but anything that's going to activate their muscles is going to, cause you know them some stress, and it's going to

6

00:00:48.770 --> 00:00:55.789

PT5: They have to work hard. So it's gonna it's gonna do a lot to strengthen their muscles if they're even if they're just moving against gravity really in any way

7

00:00:56.630 --> 00:01:11.880

NM: all right, perfect. And so my first follow up is the department of Health defines physical activity as any activity that encompasses energy expended, and activation of skeletal muscles. Does this definition change your mind about how you define physical activity?

8

00:01:12.480 --> 00:01:14.350

PT5: I mean, I feel like that's

9

00:01:14.660 --> 00:01:21.899

PT5: pretty much what I said. I I just wanted to.

NM: Yeah, I agree. got to follow my script. Excellent and

10

00:01:22.470 --> 00:01:24.940

NM: so no, that didn't change your mind.

11

00:01:25.080 --> 00:01:30.340

NM: I'm like wait, literally said the same thing. I'm: very.

12

00:01:30.930 --> 00:01:37.390

NM: So. Next follow up. How do you think physical activity differs from other types of like fitness activities?

13

00:01:39.870 --> 00:01:42.350

PT5: like people who are like into fitness.

14

00:01:42.550 --> 00:01:45.490

NM: Yeah, like so fitness is

15

00:01:45.670 --> 00:01:54.579

NM: by definition, like more structured like sports kind of you know. How would that differ from it? Doesn't have to be sports. It can be just any kind of structured

16

00:01:55.600 --> 00:01:59.490

NM: exercise, for example. how do you think it differs?

17

00:01:59.600 --> 00:02:02.430

NM: How do you think physical activity differs from fitness.

18

00:02:03.090 --> 00:02:04.440

PT5: Oh,

19

00:02:04.790 --> 00:02:07.810

PT5: So fitness, I would say, is more

20

00:02:08.570 --> 00:02:09.220

PT5: so.

21

00:02:09.610 --> 00:02:10.740

PT5: Wow!

22

00:02:11.060 --> 00:02:23.210

PT5: fitness is really it was. It's just more of a challenge. It's just adding, taking your physical activity and adding more of a challenge to it like me sitting here moving my arms like I'm physically moving. But this isn't fitness. I'm not.

23

00:02:23.270 --> 00:02:31.099

PT5: I'm not expending a significant amount of energy, you know, as opposed to. If i'm coming back from a zumba class and

24

00:02:31.890 --> 00:02:41.250

PT5: barely breathing. It's just it's expending more a significant amount of more energy, more energy than it takes to sit like stationary.

25

00:02:41.580 --> 00:02:44.109

PT5: or just like move in everyday movements.

26

00:02:44.910 --> 00:02:51.190

NM: Got it all right. And when do you witness? Your students participate most in physical activity during the school day?

27

00:02:54.940 --> 00:02:57.920

PT5: So i'm gonna go based off of

28

00:02:58.140 --> 00:03:06.539

PT5: like previous. Right? Okay, I would say, just mostly in mostly in therapy, I mean throughout the day they were all, you know.

29

00:03:06.620 --> 00:03:17.230

PT5: sitting in their wheelchairs, and I mean, but even that, like depending on what they're doing, if they're propelling their own wheelchairs, if they have that ability, even if with the the students that could use.

30

00:03:18.590 --> 00:03:25.790

PT5: Oh, my gosh! That's my brain not working right now. Like the power mobility like even that, for some of those kids is such a struggle, you know, if you're using

31

00:03:25.970 --> 00:03:40.589

PT5: the head array is that's a huge struggle, and then a lot of times like the whole body will come into play just to kind of, you know. Get the head working, and even like with the joysticks, I think, for some of the kids that's still a huge amount of energy, because they have to really.

32

00:03:41.030 --> 00:03:42.990

PT5: you know, work hard to

33

00:03:43.170 --> 00:03:46.219

PT5: to make sure that they're going in the right direction and moving.

34

00:03:48.070 --> 00:03:48.880

PT5: So

35

00:03:49.410 --> 00:03:50.620

PT5: yeah, I would say

36

00:03:50.980 --> 00:03:58.219

PT5: that. And if they are ambulatory at a gait trainer, or if they had a walker, obviously that, and then definitely obviously therapy.

37

00:03:59.630 --> 00:04:02.029

NM: Great Next question.

38

00:04:02.200 --> 00:04:05.049

NM: How do you measure physical activity.

39

00:04:05.200 --> 00:04:13.760

NM: frequency, physical activity, intensity, physical activity, time and type in children at Gmfcs level 4- 5, the non walkers.

40

00:04:16.700 --> 00:04:32.250

NM: yeah. How do you measure physical activity, frequency, intensity, time and type? You remember the fit principle? How would you measure physical activity as it relates to that in children with Cp Who are not full time Walkers

41

00:04:34.130 --> 00:04:35.480

PT5: frequency.

42

00:04:37.170 --> 00:04:40.729

PT5: I would just say, you know, anytime they're really

43

00:04:41.210 --> 00:04:52.750

PT5: moving, and you can see that they're they're stressing out, and it's not just like a simple, you know, blinking, or you know, turning my head to look again. Sometimes that's hard to. So really, just anytime that you can see

44

00:04:52.920 --> 00:04:57.599

PT5: that they're expending energy, and that it's not just something that's easy and like

45

00:04:59.610 --> 00:05:00.829

PT5: it's like breathing

46

00:05:00.880 --> 00:05:11.439

PT5: Well, again, that can also sometimes be depending on the student. Really, it's hard to say, and I would think, for each kid, it's really it's really based on per child based on what their ability

47

00:05:11.630 --> 00:05:21.109

PT5: is, and I think that goes for all of it, frequency, intensity, you know. It might be hard for John John, Jr. Over here to breathe, and because he has so much

48

00:05:21.140 --> 00:05:26.330

PT5: so many more complications and muscle weakness as opposed to

49

00:05:26.950 --> 00:05:39.310

PT5: Kathy, who you know it's harder for her to walk, but she can breathe with no problem. So I think really all of that is based per student, because it everyone has different in Gmfcs level, but not walkers like

50

00:05:39.520 --> 00:05:41.049

PT5: they all have their different.

51

00:05:41.740 --> 00:05:45.960

PT5: You know there are different abilities where some things are harder for others than

52

00:05:46.190 --> 00:05:48.140

PT5: something easier, for you know

53

00:05:49.060 --> 00:05:51.429

PT5: it's like very individualized, I would say

54

00:05:52.160 --> 00:05:57.619

NM: Thank you all right. First prompt. Do they need assistance to complete

55

00:05:57.800 --> 00:06:03.750

NM: Some of the activities you mentioned and and during and which which activities

56

00:06:03.820 --> 00:06:17.609

NM: would they need assistance? And would it be for the whole activity for the parted activity like you mentioned earlier propelling or biking, or you even mentioned like looking looking and sitting, you know. So

57

00:06:17.620 --> 00:06:26.400

NM: what how much do they need assistance for some of the things you mentioned, and during which activities do they need the most assistance, and then do they need assistance for her, all of it, or.

58

00:06:26.790 --> 00:06:28.829

NM: you know, maybe part of it.

59

00:06:28.920 --> 00:06:33.810

PT5: Yeah, I mean it's I would say they need assistance for maybe some of it, like I've probably worked with

60

00:06:34.010 --> 00:06:52.590

PT5: students on the bike, for example, who can propel them? Who can propel and move once they have like that initial momentum. But they might not be able to initiate getting the bike going, because that takes so much more energy to start the bike versus when the bike is already moving. So sometimes I might have to help, you know. Get them going, and then they can kind of pedal, and then

61

00:06:52.600 --> 00:07:10.209

PT5: they might need help steering because they can. All you know. That's a hard that's a really hard thing to coordinate steering upper, extremely hard, I mean. Look where you're going, keeping your head up, steering the bike so even if they need some assistance, you know. Same thing. If I'm sitting with a student on like a on a bolster, and i'm like holding them

62

00:07:10.220 --> 00:07:18.089

PT5: because they they don't have the core strength, and i'm holding them. But they're still, you know, reaching looking, grabbing things with their upper extremities, keeping their head up. That's still.

63

00:07:18.230 --> 00:07:23.880

PT5: you know they're still. They're still actively working. But i'm still helping. So they're not falling off the bolster

64

00:07:24.110 --> 00:07:25.929

PT5: or whatever it is that you're working on.

65

00:07:28.310 --> 00:07:34.250

NM: Perfect. And do you think they should participate in more or less of these activities? And why.

66

00:07:35.730 --> 00:07:44.270

PT5: I mean, I think it's always good. I mean more is not always better. I think we should be mindful of just how much we're, you know, putting stress on them. But I think

67

00:07:44.480 --> 00:07:46.789

PT5: if we should be putting just the right amount of

68

00:07:46.810 --> 00:07:48.440

PT5: stress.

69

00:07:48.620 --> 00:08:01.779

PT5: on each child again, individually based on what their needs are and what their capabilities are. you gotta find that like right balance. You don't want to do too little where they're not really getting anything out of. But you don't want to like over Fatigue them with them now, like they're you know

70

00:08:02.140 --> 00:08:06.329

PT5: they can't participate in the rest of the day, because they are too tired. You have to find that nice

71

00:08:06.430 --> 00:08:09.080

PT5: right balance where it's just right.

72

00:08:10.540 --> 00:08:16.700

NM: Excellent. Okay. Third question. Do you address promoting physical activity during your physical therapy sessions

73

00:08:18.700 --> 00:08:24.310

PT5: to promote addressing physical activity during I mean, yeah, every every session is for the most part has to have

74

00:08:24.420 --> 00:08:28.009

PT5: some component of physical activity, even if it's a child that

75

00:08:29.180 --> 00:08:30.210

PT5: really just.

76

00:08:30.260 --> 00:08:41.300

PT5: we're working on the mat, and we're stretching, and then I still try to work on rolling, even if it's just getting them to initiate rolling, or if i'm initiating, rolling, and then they're finishing the role. I'm still trying to get them moving. We're not going to just be

77

00:08:41.860 --> 00:08:43.650

PT5: laying around the whole time

78

00:08:44.110 --> 00:08:45.670

PT5: stretching

79

00:08:46.300 --> 00:09:03.589

NM: and okay. But if you say yes, how do you do this? You mentioned the rolling? What components of physical activity do you? Do you think you address, for example, cardiovascular endurance, muscle activation, energy, expenditure. What things do you really like? You're mostly addressing when you're working on this in therapy?

80

00:09:03.860 --> 00:09:10.360

PT5: I would say definitely muscle activation for sure. definitely cardiovascular

81

00:09:10.840 --> 00:09:12.050

PT5: depending, you know.

82

00:09:12.260 --> 00:09:17.360

PT5: because it's no matter what. If you're if you're having a child, move that isn't really

83

00:09:17.410 --> 00:09:24.370

PT5: moving that isn't able to move so much, no matter what you're gonna be that's gonna affect our cardiovascular system because they have to work so hard.

84

00:09:24.410 --> 00:09:26.730

PT5: even just if it's rolling sometimes.

85

00:09:27.040 --> 00:09:29.420

PT5: and then what was the third one?

86

00:09:29.630 --> 00:09:43.499

NM: Oh, I mentioned, and it could be more than this. So i'm just. This is now cardiovascular endurance, muscle, animation, energy, expenditure to 3. I gave me samples of yeah.

PT5: Oh, and definitely any, or to expenditure there again. Same thing, like if they're working against gravity to try to

87

00:09:43.510 --> 00:09:54.040

PT5: role, pick their head up as move their arms or sit up. Just sit up, and some is just sitting up to. They're always expending. If they're moving and they're physical, and they're like physically moving.

88

00:09:54.440 --> 00:09:58.389

PT5: doing things that are tough for them. It's they're going to be expending energy. Of course.

89

00:09:59.590 --> 00:10:04.620

NM: this is just a question I was thinking, so Does it have to be tough for them to expand energy?

90

00:10:04.840 --> 00:10:06.559

NM: and with the kind of task?

91

00:10:07.860 --> 00:10:14.340

PT5: No, it doesn't have to be tough, like. If you're moving, you're going to spend energy regardless, but just in terms of

92

00:10:14.870 --> 00:10:18.549

PT5: strengthening and stressing out a little bit

93

00:10:18.870 --> 00:10:20.430

PT5: stressing their whole body.

94

00:10:23.100 --> 00:10:23.970

NM: Yeah.

95

00:10:29.060 --> 00:10:40.560

NM: and last question before I get to the the scale is, do you address promoting physical activity now, outside of your therapy session you mentioned in your session how you do it outside.

96

00:10:41.380 --> 00:10:45.090

PT5: so there always has to always, always has to be carry over

97

00:10:45.170 --> 00:11:00.650

PT5: either in the classroom or at home with the parents. because really, however long you're seeing that student, if i'm seeing a student for an hour twice a week, and they're not. There's no carry over in the classroom at home. It's not there's you're not going to get anything accomplished because

98

00:11:00.800 --> 00:11:02.480

PT5: an hour or 2

99

00:11:03.280 --> 00:11:04.930

PT5: twice a week.

100

00:11:05.620 --> 00:11:09.429

PT5: and then you have all the other hours of the day, like your has to be carry over

101

00:11:09.680 --> 00:11:10.590

PT5: for sure.

102

00:11:13.740 --> 00:11:19.830

NM: Have you recommended any community programs or events to your students to help increase physical activity.

103

00:11:20.540 --> 00:11:21.640

PT5: yeah.

104

00:11:22.000 --> 00:11:23.849

PT5: I would say. You know, there's always

105

00:11:24.190 --> 00:11:28.389

PT5: taking taking kids into the pool is always a good one.

106

00:11:29.170 --> 00:11:36.770

PT5: hippo therapy is a really good one, especially for this population. There's lots of good things, and I definitely would always recommend

107

00:11:37.090 --> 00:11:49.350

PT5: any of those activities, whatever it is, as long as it gets the kids moving. It's not easier for the parents to if they are in an activity that's more directed other than besides the parents just having to like kinda come up with stuff and figure it out on their own

108

00:11:50.510 --> 00:11:51.670

NM: that's good.

109

00:11:53.790 --> 00:12:01.599

NM: so it's better when it's structured like it's like a something already going on like they should bring the kids?

110

00:12:02.830 --> 00:12:04.120

PT5: It doesn't have to be. But

111

00:12:04.160 --> 00:12:06.029

PT5: yeah, it could be more helpful.

112

00:12:08.480 --> 00:12:18.490

NM: Okay? And then what types of equipment have you recommended to help improve home or community engagement and physical activity outside of the clinical setting?

113

00:12:18.850 --> 00:12:26.510

PT5: I definitely really love those. The bikes, the adaptive bikes, are probably my favorite

114

00:12:26.920 --> 00:12:32.579

PT5: gait trainers, even standers, even, you know you, if the kid is supported in a stander, and then they can still do

115

00:12:32.630 --> 00:12:40.479

PT5: and move and work on head control. And, you know, using upper extremities. Those are probably, I would say, definitely, my favorites for sure.

116

00:12:43.020 --> 00:12:43.740

NM: Excellent.

117

00:12:44.060 --> 00:12:48.940

NM: All right, Part 2. So now pull up this the the survey

118

00:12:49.250 --> 00:12:52.150

NM: that I I mentioned and

119

00:12:52.640 --> 00:12:54.040

NM: let me share my screen.

120

00:12:58.010 --> 00:12:59.680

NM: Okay? So

121

00:13:00.940 --> 00:13:04.709

NM: i'm gonna let you look at this for a second. what? I'm gonna ask you in a moment.

122

00:13:04.800 --> 00:13:15.909

NM: It's a great these questions. So this is a physical activity survey, so the parent will fill this out for their child on what they think they did in the last 7 days.

123

00:13:15.960 --> 00:13:30.779

NM: and what I need what i'm interviewing therapist about, and i'm going to do. Parents as well is to find out how appropriate these questions. But this population the non. And then i'm asked you to rate it so 0 would be not appropriate at all.

124

00:13:31.150 --> 00:13:35.799

NM: 5 is highly appropriate. Okay? And then i'm gonna ask you why

125

00:13:35.870 --> 00:13:37.990

NM: So hold on. Let me get back to my

126

00:13:42.920 --> 00:13:45.339

NM: Okay. So the first question.

127

00:13:46.330 --> 00:13:54.060

NM: How many days did your child exercise a place so hard that his or her body got tired. E. 0 is not appropriate.

128

00:13:54.080 --> 00:13:57.779

NM: 5 is highly appropriate. What do you? What would you write this question.

129

00:14:11.060 --> 00:14:12.499

NM: Jack? When they lose you?

130

00:14:13.650 --> 00:14:15.000

NM: I think I did.

131

00:14:29.080 --> 00:14:30.020

NM: Jacqueline.

132

00:15:21.660 --> 00:15:32.810

NM: Okay, Glad you made it back. All right, let's continue so. The first question was, how many days did your child exercise a play so hard that his or her body got tired.

133

00:15:33.560 --> 00:15:40.349

NM: How would you rate that question? 0 is not related on a scale from 0 to 5 5 is highly appropriate. How would you rate that question?

134

00:15:44.760 --> 00:15:52.150

PT5: I don't know. I don't really like the wording of it so much. I don't think

135

00:15:54.400 --> 00:15:57.000

PT5: I don't know

136

00:15:57.810 --> 00:16:00.150

PT5: because it depends on what the parents gonna be like.

137

00:16:00.400 --> 00:16:11.940

PT5: considering, you know, like what we talked about like just simpler rolling, reaching, sitting. They're going to consider that exercise, you know, or if they're not going to, I think is really what the issue is going to be.

138

00:16:12.170 --> 00:16:14.989

PT5: because, like you were talking like exercise, like.

139

00:16:15.410 --> 00:16:22.960

PT5: you know, running a mile, or, you know, doing sit ups or push ups like these kids. Aren't going to do that. So the parents really have to like. Have a better understanding of.

140

00:16:24.210 --> 00:16:27.470

PT5: you know, for their child what's going to

141

00:16:28.030 --> 00:16:29.560

PT5: the exercise?

142

00:16:30.800 --> 00:16:35.520

NM: 0 not related at all. 5 highly appropriate. Give me a number.

143

00:16:35.600 --> 00:16:37.840

PT5: Oh, i'm gonna go with

144

00:16:38.430 --> 00:16:39.860

PT5: one. It's not

145

00:16:40.070 --> 00:16:44.110

NM: yeah one. We'll go with one all right next

146

00:16:44.960 --> 00:16:46.100

NM: Number 2.

147

00:16:47.290 --> 00:16:53.689

NM: How many days your child child exercise really hard for 10 min or more. How would you rate this one? And why?

148

00:16:54.100 --> 00:16:59.859

PT5: Yeah, I don't like that either. I think that's even worse. I'm gonna go that one to 0. I don't like it

149

00:17:00.270 --> 00:17:04.999

PT5: this like really hard again, like it's just gonna

150

00:17:05.800 --> 00:17:06.370

I like it

151

00:17:06.770 --> 00:17:09.160

really hard. It's just so like

152

00:17:10.150 --> 00:17:14.249

PT5: again that's going to be like. Oh, my child, ran 2 laps on the track. You know it's

153

00:17:14.480 --> 00:17:15.339

PT5: It's just

154

00:17:16.210 --> 00:17:19.590

PT5: for these kids. It's not. I don't think the wording is really appropriate.

155

00:17:21.730 --> 00:17:23.190

NM: All right, Number 3.

156

00:17:24.260 --> 00:17:29.900

NM: How many days did your child exercise so much that he or she breathed breathe hard

157

00:17:31.750 --> 00:17:34.730

PT5: alright, that one might be a little better, but

158

00:17:36.020 --> 00:17:38.749

PT5: that one's a little better, I think.

159

00:17:39.460 --> 00:17:40.939

PT5: i'm gonna give it.

160

00:17:44.560 --> 00:17:51.519

NM: because there's so many other factors that can be causing them to breathe hard. Let's talk about that like it's tough

161

00:17:51.790 --> 00:17:58.769

PT5: If you're a kid that has a hard time breathing To begin with, you know what I mean, that's gonna be all day long, cause like, how are you gonna differentiate between?

162

00:17:59.180 --> 00:18:09.220

PT5: They exercise the hard that they're not breathing, or they're just not breathing, because there's something like internal going on in their body that they can't express to you, because they probably maybe can't talk.

163

00:18:09.350 --> 00:18:12.729

PT5: How are you differentiating? What's causing them to breed so hard.

164

00:18:13.440 --> 00:18:17.659

PT5: Maybe i'm thinking too hard into this. I don't know all good points.

165

00:18:18.060 --> 00:18:19.839

PT5: So what number would you give it?

166

00:18:20.800 --> 00:18:22.320

PT5: I'm gonna give this one up.

167

00:18:23.370 --> 00:18:24.630

PT5: I guess. One of 2.

168

00:18:24.790 --> 00:18:25.540

NM: Okay.

169

00:18:27.130 --> 00:18:28.309

NM: Number 4.

170

00:18:30.890 --> 00:18:33.490

PT5: I definitely this is a 0.

171

00:18:34.460 --> 00:18:36.979

PT5: I don't like it

172

00:18:37.050 --> 00:18:50.370

PT5: because it's a sweating piece. Yeah, not all like If there's like neurologically stuff going on right? Obviously, if these gets a Cp. They have neurological issues. Some of them might be sweating too much all day. Some of them might just not sweat at all.

173

00:18:50.400 --> 00:18:54.910

PT5: That's definitely. I feel like something I've seen so no, definitely not. I don't like it.

174

00:18:55.910 --> 00:18:56.660

PT5: 0.

175

00:18:56.840 --> 00:19:00.730

NM: I got it all that. All good points, all right. Number 5.

176

00:19:01.570 --> 00:19:06.589

NM: How many days your child exercise or play so hard to his or her muscles learned

177

00:19:07.320 --> 00:19:09.459

PT5: also 0. How are you gonna know that?

178

00:19:10.180 --> 00:19:12.629

PT5: How was it? How is the child going to know that, like

179

00:19:12.890 --> 00:19:14.190

PT5: hey? Express it?

180

00:19:14.610 --> 00:19:16.130

PT5: All of these children

181

00:19:16.370 --> 00:19:19.999

PT5: probably maybe can't express that. And then, like that's a hard.

182

00:19:20.260 --> 00:19:31.330

PT5: It's a hard concept for a child to even understand. Like all my muscle burns. Nope. Kind of like it. So the parent is the one filling this out right. But how is the parent going to know that their child, that their that their kids muscles are burning.

183

00:19:31.540 --> 00:19:34.280

PT5: If a kid can't express that, or if a kid can't

184

00:19:34.590 --> 00:19:35.270

PT5: like

185

00:19:35.530 --> 00:19:37.170

PT5: understand, like

186

00:19:37.490 --> 00:19:42.680

PT5: what that means is what I is what i'm saying like if a child can't express that back to their parents, how the heck are they gonna know?

187

00:19:42.750 --> 00:19:43.550

NM: Yeah.

188

00:19:43.640 --> 00:19:45.099

PT5: Yeah, no, I don't like it.

189

00:19:45.500 --> 00:19:46.990

NM: Got it. Yeah.

190

00:19:48.670 --> 00:19:56.960

NM: That's good. All good points. Number 6. How many days did he did your child exercise or play so hard that he or she felt tired.

191

00:19:58.400 --> 00:19:59.830

PT5: It's like the same as

192

00:19:59.950 --> 00:20:01.600

PT5: it's like the same as the first one.

193

00:20:01.790 --> 00:20:07.989

NM: But the difference is this: one is felt tired, and the first one is got tired.

194

00:20:08.230 --> 00:20:11.210

PT5: Yeah, that's yeah, that's I one.

195

00:20:12.300 --> 00:20:13.940

NM: And why would you say one?

196

00:20:14.980 --> 00:20:19.860

PT5: Because it's just it's the same thing like there's other. There could be so many other things going on that

197

00:20:20.180 --> 00:20:23.530

PT5: you're not going to know if it's necessarily contributed to the exercise or not.

198

00:20:24.310 --> 00:20:27.100

PT5: And the child again has to be able to like that.

199

00:20:29.620 --> 00:20:31.280

PT5: Yeah, it's hard. It's hard.

200

00:20:32.550 --> 00:20:34.120

NM: Yeah, I really do.

201

00:20:35.570 --> 00:20:37.339

NM: And number 7.

202

00:20:38.590 --> 00:20:42.430

NM: How many days was your child physically active for 10 min or more.

203

00:20:42.560 --> 00:20:43.939

PT5: Oh, that sounds a little better.

204

00:20:43.980 --> 00:20:49.299

PT5: I like this one. I'm going to give this one a 5. This is probably the only one that I like.

205

00:20:49.600 --> 00:20:56.360

PT5: Well, because that's a little easier to see like if You' if the child is moving around at all like that's a little easier to

206

00:20:57.390 --> 00:20:58.690

PT5: see and understand

207

00:20:59.110 --> 00:21:01.560

PT5: versus all these other crazy ones.

208

00:21:01.970 --> 00:21:03.400

PT5: Their muscles are burning

209

00:21:03.560 --> 00:21:04.410

NM: right

210

00:21:04.460 --> 00:21:05.479

crazy.

211

00:21:05.780 --> 00:21:08.650

NM: all right. I got the last one for you. I already know.

212

00:21:09.200 --> 00:21:13.720

PT5: How many days did your child run for 10 min or more.

213

00:21:15.090 --> 00:21:17.989

PT5: I didn't want to run for 10 min or more. Forget it.

214

00:21:19.190 --> 00:21:21.770

PT5: so it's not applicable.

215

00:21:21.810 --> 00:21:22.949

PT5: No, this is terrible.

216

00:21:23.220 --> 00:21:25.339

PT5: I hope your Research isn't

217

00:21:25.660 --> 00:21:35.080

PT5: promoting this because I just ripped it apart. I'm sorry

218

00:21:35.590 --> 00:21:40.220

NM: it's it's hard. It's hard to discern, and trying to figure out how to

219

00:21:40.320 --> 00:21:55.430

NM: is why i'm asking a lot of pts, you know, trying to get some trying to get a good understanding of what people are doing and what ideas people have. So we're at the end. I usually like to give you a chance to ask some final words about how you, I think maybe

220

00:21:55.440 --> 00:22:00.669

NM: physical activity can be addressed in this population, or any ideas, any comments you want to say for the end.

221

00:22:01.340 --> 00:22:02.750

PT5: Yeah, it's. It's

222

00:22:02.950 --> 00:22:06.229

PT5: like we like, I said. And all these it's just it's so hard to

223

00:22:07.730 --> 00:22:13.240

PT5: measure it. I guess I mean it's easier to see like you can tell if you're working with a child and like

224

00:22:13.620 --> 00:22:18.309

PT5: you're putting stress on their muscles. It's just on their body, and if they're act physically, acting like they might.

225

00:22:18.330 --> 00:22:20.610

PT5: you know they might turn red, or they might.

226

00:22:20.970 --> 00:22:23.150

PT5: Their breathing might change, but like.

227

00:22:25.500 --> 00:22:27.470

PT5: and I think it's. But I think it's important.

228

00:22:27.520 --> 00:22:30.939

PT5: that we do keep them physically active, and they're not just kinda

229

00:22:32.700 --> 00:22:36.649

PT5: not doing anything. But again, a lot of

230

00:22:37.150 --> 00:22:41.240

PT5: oh, most most most movements are going to stress them out

231

00:22:41.260 --> 00:22:43.540

PT5: cause some physical activity.

232

00:22:43.830 --> 00:22:47.709

PT5: You say most movements do you include the passive movements to?

233

00:22:48.390 --> 00:22:56.170

PT5: Okay. No most active movements, Thank you. I mean, because sometimes. But sometimes, if you're stretching them that's going to stress them right out, too.

234

00:22:56.500 --> 00:22:59.789

NM: I'll i'll. I'll make sure. I clear our cleaning. Active.

235

00:22:59.920 --> 00:23:01.190

PT5: Okay, Thank you.

236

00:23:01.300 --> 00:23:02.180

Okay.

237

00:23:04.480 --> 00:23:06.580

Some

238

00:23:07.380 --> 00:23:13.639

NM: and okay. I'll wrap up now, and I'll stop the recording, but we can definitely continue to

239

00:23:14.880 --> 00:23:16.110

NM: thank you for your time.
