# Supplementary material for: Validity of PROMIS® Pediatric Physical Activity Parent Proxy Short Form Scale as a Physical Activity Measure for Children with Cerebral Palsy Who Are Non-Ambulatory
Source: Behav Sci (Basel). 2025 Jul 31;15(8):1042. doi: 10.3390/bs15081042 (PMC12382615; doi:10.3390/bs15081042)
Supplement: Supplementary file 1 [file behavsci-15-01042-s001.zip › Transcripts copy/PT transcripts - deidentified/PT19.docx]

WEBVTT

1

00:00:00.580 --> 00:00:01.929

NM: Just now. All right.

2

00:00:02.500 --> 00:00:20.959

NM: Good morning again. Thank you so much for joining us today. So I am going to be asking you a few questions about physical activity and children with Cp. They are levels GM: message level 4 and 5. If it sounds like I am reading a script is because I am I'm just trying to be, as

3

00:00:21.000 --> 00:00:37.160

NM: you know, because consistent as possible. So bear with me in that. And then on the second half of the interview. We're going to look at a scale called the promise scale that was developed by the Nih for children with disabilities. So parent proxy scale. So the parent would

4

00:00:37.240 --> 00:00:44.539

NM: basically answer the questions for their child, and we'll look at the second half. So again, thank you for your time. And here our first. My first question.

5

00:00:46.010 --> 00:00:52.289

NM: How do you define physical activity for children with Cp. Who are not full time? Walkers

6

00:00:53.370 --> 00:01:02.630

PT19: physical activity is some sort of active movement. So if it's rolling at MoD assist, or even

7

00:01:03.520 --> 00:01:07.850

PT19: sitting and testing core balance, it's

8

00:01:08.390 --> 00:01:19.379

PT19: for the yeah for kids who aren't active walkers. It's some sort of contribution, some some sort of participation in pretty much any activity that is at their level.

9

00:01:22.330 --> 00:01:23.460

NM: Thank you.

10

00:01:23.550 --> 00:01:37.169

NM: So related to that. The Department of Health defines physical activity as any activity that encompasses energy expended, and activation of skeletal muscle. Does this definition change your mind about how you define physical activity?

11

00:01:38.150 --> 00:01:41.030

PT19: I think it's pretty similar. So

12

00:01:41.340 --> 00:01:47.449

PT19: because for these kids I mean even just moving a muscle or 2 is energy expenditure.

13

00:01:49.470 --> 00:01:55.779

NM: So you're basic, no cause it's kind of what you Guys

14

00:01:56.440 --> 00:01:57.410

great. Thank you.

15

00:01:57.450 --> 00:02:03.050

NM: And how do you think physical activity differs from other types of fitness activities?

16

00:02:03.960 --> 00:02:07.839

PT19: I mean, fitness. Activity is thought about, you know thing.

17

00:02:07.940 --> 00:02:14.120

PT19: maybe a higher level of energy expenditure. So people usually think about a heart rate going up

18

00:02:14.200 --> 00:02:20.890

PT19: and sweating, and maybe more movement for kids who like

19

00:02:21.160 --> 00:02:23.680

PT19: who aren't in these particular settings.

20

00:02:25.420 --> 00:02:30.799

NM: and when do you witness your students participate in most in physical activity during the school day?

21

00:02:32.890 --> 00:02:33.640

PT19: I mean

22

00:02:34.080 --> 00:02:41.050

PT19: I I see them for an hour a day. So in my sessions they're doing more than in any other sessions.

23

00:02:41.210 --> 00:02:45.250

PT19: and then it depends on the kids. Some kids are

24

00:02:45.830 --> 00:02:54.979

PT19: aroused, and awake, and alert every day, and then other kids. I'm lucky if I get half a session once a week where they want to open their eyes.

25

00:02:59.420 --> 00:03:00.580

NM: Thank you.

26

00:03:00.690 --> 00:03:02.510

NM: All right. So second question.

27

00:03:02.530 --> 00:03:11.300

NM: How do you measure physical activity, frequency, intensity, time and type and children with Cp. Who are not full time walkers.

28

00:03:11.460 --> 00:03:22.469

PT19: Okay, so frequency, I mean, every time i'm thinking of one or 2 of my particular kids to a very little active movement. So for them, anytime.

29

00:03:23.040 --> 00:03:28.599

PT19: they turn, turn their head anytime. They open their eyes

30

00:03:29.900 --> 00:03:35.329

PT19: then for other kids. It's it depends on where you meet them, so to

31

00:03:35.360 --> 00:03:41.369

PT19: I guess, at the lowest level of the scale for a level 5 who's low aroused

32

00:03:42.110 --> 00:03:51.810

PT19: arousal and some movement. But the kids at 5 who are like a Gmfcs., 5 5. But who are active, Who can move.

33

00:03:51.950 --> 00:03:57.090

PT19: Then, however much time they're moving, that's not an off... that’s not an official rest break.

34

00:03:58.640 --> 00:04:13.509

NM: Does that make sense? I don't know if I you You listed a lot of things. Let me know if I didn't hit one of the No, that's great. So like, and you know, I'm i'm quoting like the fitt principle, right? So frequency, intensity, time and tight. I believe you you did that, you? I'll just

35

00:04:13.610 --> 00:04:23.360

NM: kind of reiterate what you said, basically anytime they have any movement would be. The frequency is what I have here. It also depends on where you meet them.

36

00:04:23.510 --> 00:04:25.370

NM: and a level of arousal.

37

00:04:25.560 --> 00:04:35.469

NM: and for how much time they are moving, based and compared to.

PT19: And then type is hard because I am not. All of the kids have different types of activity.

38

00:04:35.550 --> 00:04:47.739

PT19: So I mean a kid who can move more. I guess I could do static and dynamic like if he's holding a position, or if he's actively like rolling or something. But they don't all have that much

39

00:04:48.220 --> 00:04:51.129

NM: differentiation.

40

00:04:54.490 --> 00:05:00.770

NM: Okay, that's great. Is it related to that? Do they need assistance to complete

41

00:05:00.910 --> 00:05:02.320

NM: these activities?

42

00:05:03.690 --> 00:05:04.520

PT19: Yes.

43

00:05:04.650 --> 00:05:05.860

PT19: most

44

00:05:06.070 --> 00:05:08.619

PT19: the vast majority. Yes.

45

00:05:08.720 --> 00:05:14.660

PT19: I mean it could be Max assist it could be men. But there's usually an assistance level.

46

00:05:18.090 --> 00:05:21.360

NM: And is it for part of the task, the entire task?

47

00:05:23.930 --> 00:05:29.850

PT19: It depends on the kid. Sometimes it's the entire task. Sometimes it's just initiation.

48

00:05:30.230 --> 00:05:32.280

PT19: Sometimes it's completion.

49

00:05:37.740 --> 00:05:46.599

NM: And do you think they should participate in more or less? And each these activities you mentioned, and you mentioned a lot of activity as well? You talked about.

50

00:05:46.730 --> 00:06:06.599

PT19: Should feels weird because I try to. I try to push kids as much as I think they can tolerate. Would I like them to have more activity, maybe interspersed throughout different times of their day? Sure would I like there to be more activity absolutely, but sometimes

51

00:06:06.610 --> 00:06:23.089

PT19: half an hour, once a day is all it can can really handle. If they have the capacity, then absolutely, I feel like the more you can promote movement. It usually contributes to an arousal level, and then they're going to be more interactive in other sessions and other activities.

52

00:06:29.510 --> 00:06:35.470

NM: Okay, next question, do you address promoting physical activity during your PT sessions.

53

00:06:36.510 --> 00:06:51.669

PT19: Yeah, definitely, either directly with the kid or explaining what the power can do. And but there are professional activities they can do in downtime or helping the nurses so either directly to the Kid or their

54

00:06:51.780 --> 00:06:53.100

PT19: surrounding staff.

55

00:06:54.900 --> 00:06:57.929

NM: And in your therapy session.

56

00:06:58.370 --> 00:07:02.510

NM: how do you do this Promoting of physical activity?

57

00:07:03.060 --> 00:07:18.570

PT19: So either it'll usually start verbally. I'll explain what I'm doing or what I want to do. Will Demo, act it out. We will demonstrate activities depending on how difficult it is, it could be hand over hand.

58

00:07:18.840 --> 00:07:31.860

PT19: Excuse me with either the Kid or the Para, or both, depending on what you're trying to do so as many ways as possible, verbal, visual, tactile, as many as I can incorporate.

59

00:07:34.650 --> 00:07:50.860

NM: And what components of physical activity do you feel like you address most of these sessions. So, for example, cardiovascular endurance, muscle and activation, energy, expenditure, mobility, what what things do you feel like? You mostly address? So everyone's muscle activation.

60

00:07:50.870 --> 00:08:05.979

PT19: I I definitely that that's the the basic like. If all I can do is get a kid to rotate his head. Then we got muscle activation and rotating His head a tiny bit isn't it. It probably isn't going to activate him terribly much cardiovascularly, but, like.

61

00:08:06.130 --> 00:08:17.619

PT19: take a kid that you can put in a gait trainer, and you can have them walk. So that's starting mobility. And then, if they're a kid who can move a who can move a little bit more

62

00:08:17.930 --> 00:08:25.940

PT19: if you can get them to go fast enough. You can bring in Cardio cardio exercise like I feel like they kind of scale up

63

00:08:26.850 --> 00:08:28.820

PT19: if that makes sense

64

00:08:32.400 --> 00:08:33.190

NM: great.

65

00:08:33.490 --> 00:08:34.120

NM: Hmm.

66

00:08:34.210 --> 00:08:39.719

NM: And if if there's a reason why you wouldn't focus on any of these, why wouldn't you

67

00:08:40.220 --> 00:08:43.710

PT19: would there be a reason?

68

00:08:43.799 --> 00:08:49.489

PT19: Contraindications, If there is a fracture I mean basic red flag stuff, or

69

00:08:50.250 --> 00:09:01.960

PT19: if they are not at a physical level to it, to expend cardiovascularly, I'm. Always into independence and mobility and muscle activation, unless there's one like an explicit

70

00:09:02.640 --> 00:09:07.529

PT19: red flag or like contraindication. I can't do that. I'm always going to do that.

71

00:09:14.790 --> 00:09:15.700

NM: Okay.

72

00:09:17.050 --> 00:09:24.649

NM: Thank you. All right next question. Do you address promoting physical activity that occurs outside of your Pt session?

73

00:09:25.190 --> 00:09:26.480

PT19: Absolutely

74

00:09:27.950 --> 00:09:44.939

PT19: as much as I can, especially if kids have free time. I'll like, you know. Okay. So they're in academics. You can sit them on a bench in this way. They have some free time. Let's put them in the bike definitely. I always as as much as a kid can tolerate as much as they have time for. Definitely.

75

00:09:47.350 --> 00:09:52.950

NM: Have you recommended any community programs or events, your students to help increase physical activity.

76

00:09:56.100 --> 00:10:03.049

PT19: Let's see. I I guess I do work. I have another side job. I do hippotherapy.

77

00:10:03.260 --> 00:10:09.100

PT19: so sometimes i'll recommend that for parents. I think it could be good for them.

78

00:10:09.800 --> 00:10:11.760

PT19: I know there are.

79

00:10:11.950 --> 00:10:18.440

PT19: or like, try to put parents in touch with you. Some of the kids do like it's active swing, adaptive, a surfing, and things like that.

80

00:10:18.520 --> 00:10:22.089

PT19: So those kind of programs as much as i'm aware of.

81

00:10:22.270 --> 00:10:24.450

PT19: I'm sure there's plenty I don't know.

82

00:10:28.000 --> 00:10:36.559

NM: And what type of equipment have you recommended to help improve home and or community engagement and physical activity outside of the clinical setting.

83

00:10:36.700 --> 00:10:37.820

PT19: So

84

00:10:37.990 --> 00:10:54.489

PT19: standers I've recommended standers, gait trainers more difficult to achieve, but with families with financial assistance, totally into adaptive bicycles, I think that's great to bring everywhere.

85

00:10:57.570 --> 00:11:00.549

PT19: Yeah, I think that that's about that's about it.

86

00:11:03.050 --> 00:11:05.909

NM: Awesome. Okay. So now i'm going to share

87

00:11:06.440 --> 00:11:09.510

PT19: the survey I mentioned earlier. Cool.

88

00:11:10.370 --> 00:11:11.840

NM: Wendy, how are you?

89

00:11:13.540 --> 00:11:22.500

PT19: I put the baby down, should I? I can turn the video back on if you want? Oh, no, I don't need the video. You're good. Okay, no problem. Here we go. Let me show you. Can you see that?

90

00:11:23.240 --> 00:11:31.610

PT19: And I see I don't see anything

91

00:11:31.840 --> 00:11:34.029

NM: all right. Great. So okay, great.

92

00:11:34.070 --> 00:11:43.339

NM: And you can take your time and look at this is called the parent proxy. Physical activity Survey. So this has 8 questions, and this is usually geared to a parent or caregiver.

93

00:11:43.370 --> 00:11:51.909

NM: and they have to answer. How many days in the past week have their child, you know, done one of these things. So what i'm going to ask you to do is you just take a moment to look at this

94

00:11:51.970 --> 00:11:54.200

NM: is to give me a rating

95

00:11:54.260 --> 00:12:03.230

NM: for each question. So i'm going to ask you on a scale from 0, not related at all to 5 being highly appropriate.

96

00:12:03.940 --> 00:12:09.250

NM: How does appropriate as a question to addressing physical activity, intensity.

97

00:12:09.270 --> 00:12:11.749

NM: and children with Cp. At levels 4 and 5.

98

00:12:12.130 --> 00:12:16.379

NM: Okay. So i'll. I'll start. We'll go one by one.

99

00:12:16.680 --> 00:12:28.399

NM: Okay. So for the first question, i'll read it. I'll read each one. How many days did your child exercise or play so hard that his or her body got tired? How appropriate would you think of parent

100

00:12:28.730 --> 00:12:32.619

NM: for a parent with a child or a caregiver.

101

00:12:32.830 --> 00:12:37.449

NM: Would this be really in just in terms of assessing physical activity, intensity

102

00:12:37.640 --> 00:12:42.709

NM: for a child at levels 4 and 5 0 not related at all. 5 highly appropriate.

103

00:12:43.430 --> 00:12:48.820

PT19: I don't know, maybe of 3, cause there I don't know half

104

00:12:48.980 --> 00:12:54.029

PT19: half the kids this could be appropriate for, and then there's half that. It's not

105

00:12:54.340 --> 00:12:59.970

PT19: to a a 3. I guess

106

00:13:00.130 --> 00:13:08.780

PT19: there are kids that can move their bodies enough to get so first it's hard to tell if the kids tired, but you can assume that a parent probably knows that knows how to read the queues. But

107

00:13:08.910 --> 00:13:13.289

PT19: there are some kids who their physical activity or what they're doing.

108

00:13:13.970 --> 00:13:17.980

PT19: You know isn't so hard that their body gets tired, but it's still meaningful work.

109

00:13:20.770 --> 00:13:26.740

PT19: So like, even if a kid is just working on reaching midline or holding their head up.

110

00:13:27.210 --> 00:13:32.840

PT19: Is their body? Is their their head physically tired? Maybe. But maybe not.

111

00:13:33.690 --> 00:13:36.569

PT19: And does that make sense. Yeah. Okay.

112

00:13:37.900 --> 00:13:39.469

NM: Okay. So number 2.

113

00:13:40.620 --> 00:13:53.440

NM: How many days did your child exercise really hard for 10 min or more? How appropriate is this for a child at levels 4 and 5. Would you rate it? 0? Not at all related, or 5 highly appropriate, or somewhere between, and why

114

00:13:54.070 --> 00:13:57.539

PT19: it's so subjective. What is really hard, mean.

115

00:13:58.020 --> 00:13:58.940

PT19: like

116

00:13:59.620 --> 00:14:00.540

PT19: I just

117

00:14:00.860 --> 00:14:19.209

PT19: it. It seems I don't like it, because whose definition of really hard, because they're apparent to like my kids not sweating. They're not working really hard, but this kid is doing everything they can just to participate, and they would consider it really hard. I don't like this one at all. I guess a 0. I don't think it's appropriate.

118

00:14:19.300 --> 00:14:20.930

NM: Okay, thank you.

119

00:14:21.880 --> 00:14:28.010

NM: And this is this is why I asked. Because it just taking it on its face. How valid is it?

120

00:14:28.330 --> 00:14:33.649

NM: Questions? So thank you for your your answers. Okay. So number 3.

121

00:14:33.750 --> 00:14:44.800

NM: How many days did your child exercise so? Ha! So much that he or she breathed hard. 0 not related at all. I highly appropriate somewhere between how and why.

122

00:14:45.350 --> 00:14:57.950

PT19: Okay, so I guess, similar similar to one. I mean, there are. There are some kids where this is absolutely a measure of how hard they worked. You'll see nasal flaring. You'll see breath movement.

123

00:14:58.020 --> 00:15:00.600

PT19: But there are lots of kids that

124

00:15:01.150 --> 00:15:06.370

PT19: don't show this particular measure of exertion.

125

00:15:06.900 --> 00:15:09.909

PT19: I don't know a 2 or 3, I guess.

126

00:15:09.990 --> 00:15:11.490

NM: Okay. Final answer.

127

00:15:11.600 --> 00:15:12.860

PT19: Oh.

128

00:15:14.050 --> 00:15:15.550

PT19: 2,

129

00:15:20.410 --> 00:15:32.940

NM: all right. Number 4. How many days was your child so physically active that he or she sweated 0? Not related at all. 5 highly appropriate, or any somewhere in between. How would you rate this question, and why?

130

00:15:33.230 --> 00:15:44.549

PT19: Maybe my cas load is biased because most of the kids I see can't sweat like, even if they were working super super hard, they don't sweat for whatever is going on with their body.

131

00:15:44.920 --> 00:15:55.369

PT19: I don't know a one like maybe there is a kid who this is appropriate for. But I again, I don't think it's an accurate in this population. An accurate descript descript

132

00:15:55.560 --> 00:15:58.599

PT19: measure of these kids working hard.

133

00:16:06.360 --> 00:16:11.300

NM: Just a follow up. Why, why would you say they're not sweating, or you don't sweat?

134

00:16:11.740 --> 00:16:13.200

PT19: I think

135

00:16:13.760 --> 00:16:15.740

PT19: the kids and i'm thinking of

136

00:16:16.800 --> 00:16:19.069

PT19: trying to think there's one kid who like

137

00:16:19.360 --> 00:16:25.399

PT19: neurologic like he doesn't, or a few kids. You don't sweat. They don't have that.

138

00:16:25.540 --> 00:16:29.080

PT19: I'm not sure if it's part of their genetic disorder or

139

00:16:29.530 --> 00:16:40.219

PT19: a neurological sign of hypothesia at some point. But it's something neurological. They never sweat, even if they were in a room that was a 1,000 degrees they don't sweat. It's just not something their body does.

140

00:16:48.540 --> 00:16:49.839

NM: Number 5.

141

00:16:49.860 --> 00:16:57.889

NM: Thank you. How many days did your child exercise a place for that? His or her muscles burn 0 not related at all.

142

00:16:58.020 --> 00:17:00.140

PT19: I'm highly appropriate.

143

00:17:01.550 --> 00:17:03.659

PT19: Well, it's again

144

00:17:03.820 --> 00:17:09.800

PT19: how you need a kid who can tell like a parent can't tell a kid, if their muscles are burning.

145

00:17:10.030 --> 00:17:17.240

PT19: So you need a kid who can communicate. You need a kid who has a has a Toby or has an assistant device, or even can hit a switch.

146

00:17:17.710 --> 00:17:20.859

PT19: And then you need a kid who can understand

147

00:17:20.980 --> 00:17:23.289

PT19: what muscles burning means.

148

00:17:23.369 --> 00:17:32.250

PT19: I don't know. I can think of maybe one or 2 kids, I see who can actually answer that question accurately. So a one.

149

00:17:32.360 --> 00:17:41.259

NM: Okay, Thank you. And thank you very much. Also, I' to confirm you can be a one for the last one and the one about breathing hard.

150

00:17:41.290 --> 00:17:43.960

PT19: Yes, okay. I want to make sure we have a number. Yes.

151

00:17:44.200 --> 00:17:45.850

NM: Number 6.

152

00:17:48.150 --> 00:17:57.149

NM: How many days did your child exercise or play so hard that he or she felt tired? 0 not related at all. I probably appropriate. How would you rate this one.

153

00:17:58.540 --> 00:18:09.329

PT19: So i'm thinking about that in the context of the last one where it's, you need a child who can tell you that they felt tired, but that also, like the first question.

154

00:18:09.690 --> 00:18:17.470

PT19: it's not that dissimilar. So they felt tired, or their body got tired. It seems kind of the same thing

155

00:18:19.750 --> 00:18:28.749

PT19: except this question. They have to tell you so. It's a they felt tired like. Is it? Does it count that

156

00:18:28.810 --> 00:18:43.330

PT19: the parents sees tired queues, or does they? Okay. So if it's so, the parents, judging if it's a parent, is judging, then it seems really similar to the first question, how would you rate it. So

157

00:18:43.850 --> 00:18:50.200

PT19: I don't. I guess I like tired better than I like some of the other ones. So maybe a 3.

158

00:18:50.320 --> 00:18:51.250

NM: Okay.

159

00:18:51.630 --> 00:18:53.370

NM: gotcha. Thank you.

160

00:18:55.950 --> 00:18:57.540

NM: And Number 7.

161

00:18:58.520 --> 00:19:05.700

NM: How many days was your child physically active for 10 min or more? 0 not related at all? 5 highly appropriate

162

00:19:05.710 --> 00:19:19.219

NM: somewhere along the spectrum from between 0 and 5 and one. I guess I like this one better because it's objective. It's easily measured as long as there was a I guess a definition of physically active.

163

00:19:19.770 --> 00:19:26.109

PT19: I don't know a 5. I like this one. It's something that you can measure it's something that you can't. Really.

164

00:19:26.720 --> 00:19:29.680

PT19: I don't know it's less ambiguous. Okay.

165

00:19:32.340 --> 00:19:33.540

NM: Number 8.

166

00:19:34.140 --> 00:19:37.499

NM: How many days is your child run for 10 min or more?

167

00:19:38.100 --> 00:19:49.979

PT19: Hero not related at all by definition of the the PET the population. You said, this is kids who aren't primary walkers. So if they're not primary walkers, there's no

168

00:19:50.100 --> 00:20:00.940

PT19: there is a very small chance that they're going to ever run at this until you change until they become more more common. Walker. 0. Absolutely not.

169

00:20:02.560 --> 00:20:17.130

NM: And then thank you. And the way I've been asking everyone to end is, I like to get your final thoughts on physical activity in this population. Anything you want to share. As your final falls comments.

170

00:20:18.280 --> 00:20:25.430

PT19: you need to remember who you're dealing with. And first physical activity is different for every kid

171

00:20:25.690 --> 00:20:37.779

PT19: you need to meet them where they're at in their ability, level. Physical activity. Isn't what you want, what you think it's going to be, or what you want it to be. You might think this kid can do one thing, but this is where they're at.

172

00:20:38.290 --> 00:20:39.220

PT19: so

173

00:20:39.270 --> 00:20:43.309

PT19: it's it's absolutely important. As long as you're not imposing.

174

00:20:45.710 --> 00:20:48.079

PT19: I don't know unrealistic expectations

175

00:20:48.260 --> 00:20:52.229

PT19: or pushing them farther than they can realistically attain

176

00:20:55.410 --> 00:20:57.100

PT19: all efforts. Valid?

177

00:20:57.350 --> 00:20:58.540

NM: Hmm.

178

00:21:06.140 --> 00:21:11.800

NM: That was great. Thank you so much for your answers. I'm: gonna stop recording and hold on. Okay, Thank you.
